# Dataset on SPT-based seismic soil liquefaction

**DOI:** 10.1016/j.dib.2018.08.043

**Published:** 2018-08-22

**Authors:** K. Onder Cetin, Raymond B. Seed, Robert E. Kayen, Robb E.S. Moss, H. Tolga Bilge, Makbule Ilgac, Khaled Chowdhury

**Affiliations:** a Dept. of Civil Engineering, Middle East Technical University, Ankara, Turkey; b Dept. of Civil and Environmental Engineering, University of California, Berkeley, CA, USA; c California Polytechnic State University, San Luis Obispo, CA, USA; d GeoDestek Ltd. Sti., Ankara, Turkey; e US Army Corps of Engineers, South Pacific Division Dam Safety Production Center, Sacramento, CA, USA

## Abstract

This data article provides a summary of seismic soil liquefaction triggering and non-triggering case histories, which were compiled, screened for data completeness and quality, and then processed for the development of triggering relationships proposed in “SPT-based probabilistic and deterministic assessment of seismic soil liquefaction triggering hazard” [1]. The database is composed of 113 liquefaction, 95 non-liquefaction, and 2 marginal liquefaction case histories, from seismic events with moment magnitude M_w_ values varying in the range of 5.9 to 8.3. A spreadsheet summary of these case histories are included along with a separate spreadsheet, by which maximum likelihood assessment was performed. These data transparently enable researchers to access case history input parameters and processing details, and to compare the case history processing protocols with the ones of different researchers (e.g.: “The influence of SPT procedures in soil liquefaction resistance evaluations.” [2], “SPT-based liquefaction triggering procedures.” [3]).

**Specifications Table**

**Table:** 

Subject area	• *Earth and Planetary Sciences* • *Engineering*
More specific subject area	*Civil Engineering, Geotechnical Engineering, Earthquake Engineering*
Type of data	*Table*
How data was acquired	*Case history data was acquired from technical publications in the form of articles, engineering and/or site reconnaissance reports, etc.*
Data format	*Raw, filtered, analyzed*
Experimental factors	*Potentially liquefiable soil layers located at varying depths with varying standard penetration test resistances and fines contents were shaken by different intensity and duration earthquakes. The performance of these sites were classified as liquefied or non-liquefied.*
Experimental features	*The dependency of liquefaction triggering performance on standard penetration test blowcounts, vertical effective stress, cyclic stress ratio, moment magnitude of the earthquake event, and fines content is examined.*
Data source location	*USA, Japan, Argentina, China, Philippines*
Data accessibility	*Data is included in this article*
	Supplementary A, B, C
Related research article	Cetin KO, Seed RB, Kayen RE, Moss RES, Bilge HT, Ilgac M, Chowdhury K. SPT-based probabilistic and deterministic assessment of seismic soil liquefaction triggering hazard. Soil Dynamics and Earthquake Engineering, 2018, Manuscript Number SOILDYN_2017_506, accepted for publication in April, 2018 [1].

**Value of the data**

• This database presents a summary of 210 liquefaction and non-liquefaction case histories compiled from a number of earthquake events in *USA, Japan, Argentina, China, Philippines.*
• This database can be used for the development of new probabilistic and deterministic seismic soil liquefaction triggering relationships.
• Additionally, a comparison among case histories and protocol details of similar databases (e.g.: Seed et al. [2], Cetin et al. [1], [4] and Boulanger and Idriss [5] is possible.

## 1 Data

This paper provides details about the collection of the data, their interpretation and analyses for the seismic soil liquefaction triggering performance of soil sites shaken by different intensity and magnitude earthquake events. The data consists of a summary spreadsheet, where mean and standard deviation of model input parameters are listed. Additionally, a separate spreadsheet is provided, which defines the maximum likelihood assessment performed on this database. Each spread sheet contains data required to reproduce the outlined assessments in the subject manuscript independently and transparently.

## 2 Experimental design, materials and methods

Seismic soil liquefaction of soil layers is defined as significant reduction in shear strength and stiffness due to increase in excess pore pressure. After major earthquakes, as a result of soil liquefaction, ground failures and deformations in the form of excessive settlement, lateral spreading, sand boils, landslides, etc. were discussed in the literature. This data article provides documentation of the field performance of these case history soil sites and the probabilistic maximum likelihood assessments performed for the development of Cetin et al. [1] seismic soil liquefaction triggering relationships. It is intended as a concise summary of a vast amount of data. The interpretations presented are those of the research team. A more detailed description of some of the details of the methods and procedures used to evaluate and analyze these field performance case histories is also presented in Cetin [6] and Cetin et al. [4], though the final evaluations presented in this article are the most recent interpretations undertaken as part of Cetin et al. [1] studies.

A summary of the case histories and a list of mean and standard deviation of input parameters is given in Supplementary A.

The output table in each spreadsheet contains the following columns:

• Case Number
• Earthquake
• Site
• Liquefied?
• Data Class
• Critical Depth Upper Bound (ft)
• Critical Depth Lower Bound (ft)
• Critical Depth (ft) =Critical depth for liquefaction
• Critical Depth (m) =Critical depth for liquefaction
• σ_critical depth_ (ft) =Standard deviation of critical depth
• Water Depth (ft)
• σ_water table_ (ft) =Standard deviation of water table
• γ_1_(pcf) =Unit weight above ground water table
• σ_γ1_ (pcf) =Standard deviation of γ_1_
• γ_2_ (pcf) =Unit weight below ground water table
• σ_γ2_ (pcf) =Standard deviation of γ_2_
• σ_v_ (psf) =Vertical total stress
• σ_σv_ (psf) =Standard deviation of σ_v_
• σ_v_׳ (psf) =Vertical effective stress
• σ_σv_׳ (psf) =Standard deviation of σ_σv_׳
• ρ_σσ_׳= Correlation coefficient between σ_v_׳ and σ_v_
• r_d_=Shear mass modal participation factor, commonly referred to as cyclic shear stress reduction factor
• σ_rd_=Standard deviation of
• a_max_ (g)= Peak horizontal acceleration
• σ_amax_=Standard deviation of a_max_
• Magnitude for K_Mw_
• Magnitude Scaling Factor (MSF)
• σ_KMw_=Standard deviation of K_Mw_
• CSR_σ׳v,Mw_=Actual CSR, not normalized to a reference state of σ׳_v_ = 1 atm, M_w_ =7.5
• σ_CSR_=Standard deviation of CSR_σ׳v,Mw_
• CSR_σ׳v=100kPa,Mw=7.5_**=** CSR normalized to σ׳_v_ =1 atm, M_w_=7.5
• σ_CSRN_=Standard deviation of CSR_σ׳v=100kPa,Mw=7._
• D_50_ (mm)=Median grain size
• σ_D50_=Standard deviation of D_50_
• FC %=Fines content
• σ_FC %_=Standard deviation of FC
• Sampler (SS: standard sampler or SWL: sampler without liners)
• C_S_= SPT correction for sampler configuration details
• Rod length
• C_R_= SPT correction factor for the rod length
• Borehole diameter (mm)
• C_B_= SPT correction for borehole diameter
• Energy Ratio (SH: Safety Hammer; DH: Donut Hammer or the percentage)
• C_E_= SPT correction for hammer energy ratio (ER)
• Number of N values
• N (mean) =Field measured standard penetration resistance (blows/30 cm)
• σ_N_ =Standard deviation of N
• C_N_=Overburden correction factor
• SPT Correction (Energy + Rod length)
• (N_1_)_60_= Standard penetration test blowcount value corrected for overburden, energy, equipment and procedural factors.
• σ_(N1)60_ =Standard deviation of (N_1_)_60_
• Reference
• V*_s,40ft_ (fps) = Shear wave velocity for the upper 40 ft
• K_σ_=Correction for overburden stress
• K_Mw_=Correction for magnitude (duration) effects
• CSR_Mw=7.5,σ׳v=100kPA_**=** CSR normalized to σ׳_v_ =100 kPa, M_w_=7.5
• N_1,60,CS_=Fines-corrected N_1,60_ value

Additionally, for each case history, a detailed presentation of the borehole data, maps, laboratory data, corrected N_1,60_ and CSR graphs, a summary page including surface manifestation, location of the site and expertise comments, are given in Supplementary B. On the summary page, the interpretations of Seed et al. [2] and Idriss and Boulanger [3] are also summarized, if available.

On the basis of the compiled resulting database, the maximum likelihood assessment was performed by using the Microsoft Excel Software Tool: Solver. The spreadsheet, which presents MLE assessments, is presented in Supplementary C.
