# Supplementary material for: Dataset on SPT-based seismic soil liquefaction
Source: Data Brief. 2018 Aug 22;20:544–8. doi: 10.1016/j.dib.2018.08.043 (PMC6126194; doi:10.1016/j.dib.2018.08.043)
Supplement: Supplementary file 3 — Supplementary material [file mmc3.pdf]

SUPPLEMENTARY B

A SUMMARY OF SEISMIC SOIL LIQUEFACTION FIELD CASE HISTORY DATA

**Case number:**	1
**Earthquake:**	1944 Tohnankai
**Magnitude:**	8.3 (M_R)
**Location:**	Ienaga
**References:**	Kishida (1969)
**Nature of Failure:**	…differential settlements occurred in addition to eruption of water.
**Comments:**	The epicenter of the earthquake is located 165 kms south-southwest of Nagoya city.<br>The seismic intensity of shaking was estimated as JMAIS V-VI.<br>In the source document, the liquefied zone was predicted at depth range of 11.5-12.5 m based on the criterias as follow :<br>Effective overburden pressure<2.0 kg/cm^2<br>D_r<75%<br>Saturated coarse grained soil with U_c<10 and 0.074mm<D_{50}<2.0 mm

**Summary of Data**	SPT						
	Cetin et al. (2016)	Idriss&Boulanger (2010)	Seed et.al. (1984)		Cetin et al. (2016)	Idriss&Boulanger (2010)	Seed et.al. (1984)
Liquefied?	Yes	Yes	Yes	D_{50}	0.150 ± 0.050		0.15
Data Class	B	-		% Fines	72.5 ± 36.9	30.0	30
Critical Depth Range (ft)	8.0 - 20.0	14.1	10.0	% PI			
Depth to GWT (ft)	8.0 ± 0.4	7.9	8.0				
σ_v (psf)	1460.0 ± 222.1	1670.8	1120.0	N	1.5 ± 0.5	2.3	2
σ_v' (psf)	1085.6 ± 101.5	1274.0	1000.0	C_R	0.90	0.95	
a_{max} (g)	0.200 ± 0.060	0.20	0.200	C_S	1.00	1.00	
r_d	0.87 ± 0.068	0.99	0.980	C_B	1.00	1.00	
CSR	0.153 ± 0.048	0.144	0.145	C_E	1.17	1.17	1.17
Equivalent Magnitude	8.1	8.1		C_N	1.40	1.32	1.37
MSF		0.85	0.94	$(N_1)_{60}$	2.2 ± 0.8	3.4	3
CSRN		0.159	0.16				

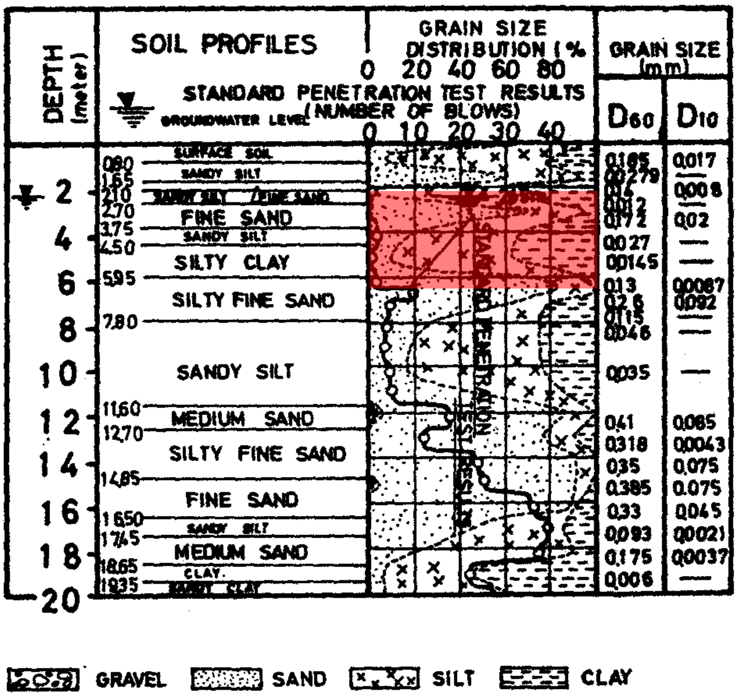

γ_m=100±3 pcf

γ_s=110±3 pcf

(III) IENAGA SHINDEN

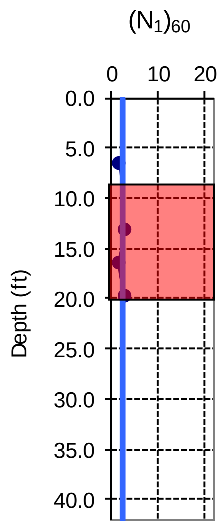

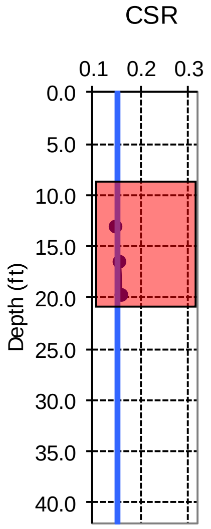

Depth (m)	Depth (ft)	CSR	N	C_N	C_R	$(N1)_{60}$	FC	clay	silt	Depth (m)	D60	D10
2.0	6.6	NA	1	1.80	0.80	***1.7***	***99.0***	40.0	59.0	2.0	0.140	0.008
4.0	13.1	0.150	2	1.42	0.89	***2.9***	***83.0***	34.0	49.0	2.1	0.012	-
5.0	16.4	0.158	1	1.33	0.92	***1.4***	***90.0***	37.0	53.0	2.7	0.172	0.020
6.0	19.7	0.160	2	1.25	0.95	***2.8***	***18.0***	4.0	14.0	4.0	0.027	-
					**Mean:**	2.2	72.5			5.0	0.015	-
					**St. Dev.**	0.8	36.9			6.0	0.130	0.0087

**Case number:**	2
**Earthquake:**	1944 Tohnankai
**Magnitude:**	8.3 (M_R)
**Location:**	Komei
**References:**	Kishida (1969)
**Nature of Failure:**	The subsidence of ground and extensive damage to houses. Eruption of water during the earthquake.
**Comments:**	The epicenter of the earthquake is located 165 kms south-southwest of Nagoya city. Borelog was obtained from a site where subsidence of the ground and extensive damage to houses occurred as a result of liquefaction of the sandy layer. A budhist temple supported on piles did not show any settlement, but the ground around the temple subsided ~40 cm and water erupted during the earthquake.<br>The seismic intensity of shaking was estimated as JMAIS V-VI.<br>In the source document,the liquefied zone was predicted in the depth range of 2-7 m based on criterias as follow :<br>Effective overburden pressure < 2.0 kg/cm2<br>D_r<75%<br>Saturated coarse grained soil with U_c<10 and 0.074mm<D_{50}<2.0 mm<br>Tip of the piles were at 8 m below ground surface.<br>SPT energy was estimated as 70 % by Seed et al (1984).

Summary of Data SPT

	Cetin et al. (2016)	Idriss&Boulanger (2010)	Seed et.al. (1984)		Cetin et al. (2016)	Idriss&Boulanger (2010)	Seed et.al. (1984)
Liquefied?	Yes	Yes	Yes	D_{50}	0.400 ± 0.050		0.4
Data Class	B	-		% Fines	9.7 ± 1.5	10.0	10
Critical Depth Range	6.4 - 16.4	17.1	17.0	% PI			
Depth to GWT (ft)	6.4 ± 0.4	6.9	7.0				
σ_v (psf)	1214.8 ± 193.4	2046.8	2040.0	N	5.8 ± 1.7	5.9	8
σ_v' (psf)	904.6 ± 92.5	1420.2	1420.0	C_R	0.87	0.95	
a_{max} (g)	0.200 ± 0.060	0.20	0.200	C_S	1.00	1.00	
r_d	0.97 ± 0.057	0.98	0.960	C_B	1.00	1.00	
CSR	0.169 ± 0.053	0.182	0.180	C_E	1.17	1.17	1.17
Equivalent Magnitude	8.1	8.1		C_N	1.53	1.25	1.17
MSF		0.85	0.92	$(N_1)_{60}$	8.9 ± 2.7	8.2	11
CSRN		0.207	0.20				

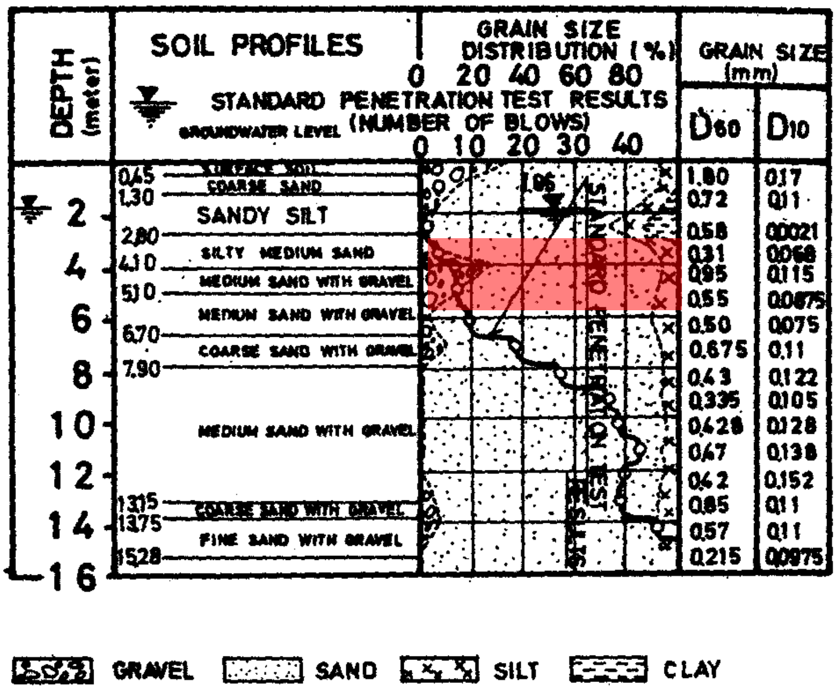

(I) KOMEI TOWN

γ_m=100±3 pcf

γ_s=115±3 pcf

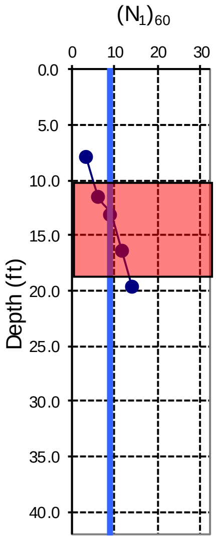

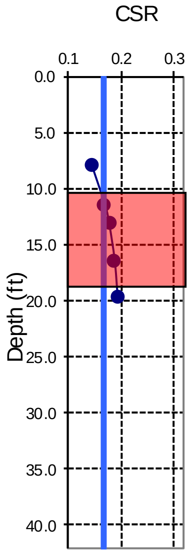

Depth (m)	Depth (ft)	CSR	N	C_N	C_R	$(N1)_{60}$	FC	clay	silt	D60	D10
2.4	7.9	0.144	2	1.72	0.82	3.3	26.0	13.0	13.0	0.580	0.002
3.5	11.5	0.170	4	1.53	0.87	***6.2***	***10.0***	0.0	10.0	0.310	0.068
4.0	13.1	0.177	6	1.46	0.89	***9.1***	***8.0***	0.0	7.0	0.950	0.115
5.0	16.4	0.188	8	1.35	0.92	***11.6***	***11.0***	0.0	8.0	0.550	0.088
6.0	19.7	0.194	10	1.26	0.95	13.9	0.0	0.0	11.0	0.500	0.075
					**Mean:**	8.9	9.7				
					**St. Dev.**	2.7	1.5				

**Case number:**	3
**Earthquake:**	1944 Tohnankai
**Magnitude:**	8.3 (M_R)
**Location:**	Meiko
**References:**	Kishida (1969)
**Nature of Failure:**	" …very fine soil came out of the ground, and houses settled as much as about 1 meter"
**Comments:**	The epicenter of the earthquake is located 165 kms south-southwest of Nagoya city.<br>The seismic intensity of sahking is estimated as JMAIS V-VI<br>Kishida (1969) predicted the liquefied zone to be from 0.5-5 m based on following criteria :<br>Effective overburden pressure<2.0 kg/cm^2<br>D_r<75%<br>Saturated coarse grained soil with U_c<10 and 0.074mm<D_{50}<2.0 mm.
**Summary of Data**	SPT

	Cetin et al. (2016)	Idriss&Boulanger (2010)	Seed et.al. (1984)		Cetin et al. (2016)	Idriss&Boulanger (2010)	Seed et.al. (1984)
Liquefied?	Yes	Yes	Yes	D_{50}	0.200 ± 0.050		0.2
Data Class	B			% Fines	19.3 ± 10.8	27.0	27
Critical Depth Range	1.6 - 11.5	12.1	12.0	% PI			
Depth to GWT (ft)	1.6 ± 0.3	6.9	7.0				
σ_v (psf)	703.2 ± 181.9	1441.1	1440.0	N	2.0 ± 0.9	1.0	1
σ_v' (psf)	397.3 ± 81.5	814.5	820.0	C_R	0.80	0.85	
a_{max} (g)	0.200 ± 0.060	0.20	0.200	C_S	1.00	1.00	
r_d	0.92 ± 0.036	0.99	0.980	C_B	1.00	1.00	
CSR	0.212 ± 0.066	0.225	0.225	C_E	1.17	1.17	1.17
Equivalent Magnitude	8.1	8.1		C_N	2.00	1.70	1.49
MSF		0.85	0.94	$(N_1)_{60}$	3.6 ± 1.6	1.7	1.5
CSRN		0.245	0.24				

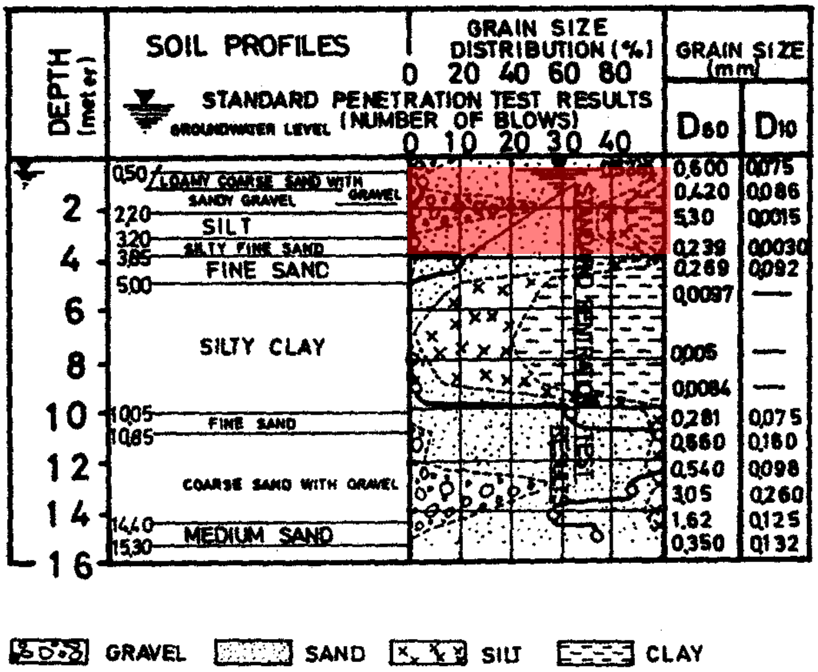

γ_m=100±3 pcf

γ_s=110±3 pcf

(II) MEIKO STREET

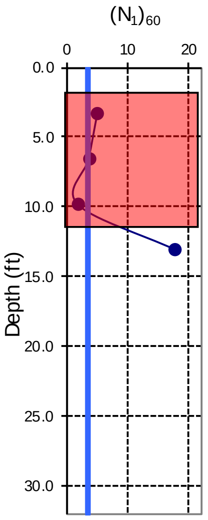

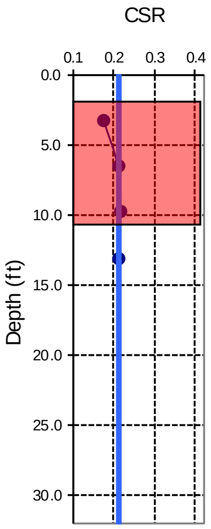

Depth (m)	Depth (ft)	CSR	N	C_N	C_R	$(N1)_{60}$	FC	clay	silt	D60	D10
1.0	3.3	0.178	3	2.00	0.73	***5.1***	***7.0***	0	7	0.420	0.085
2.0	6.6	0.212	2	2.00	0.80	***3.7***	***27.0***	15	12	5.300	0.002
3.0	9.8	0.218	1	1.95	0.85	***1.9***	***24.0***	10	14	0.238	0.003
4.0	13.1	0.214	10	1.73	0.89	17.9	18.0	6	12	0.269	0.082
					**Mean:**	3.6	19.3				
					**St. Dev.**	1.6	10.8				

**Case number:**	4
**Earthquake:**	1948 Fukui
**Magnitude:**	7.0 (Mw) USGS Centennial Earthquake Catalog
**Location:**	Shonenji Temple
**References:**	Kishida (1969)<br>Hamada et al (1989)
**Nature of Failure:**	"… eruption of water and sand volcanoes were quite promonent, and the main building of the temple settled 0.30 m"
**Comments:**	The epicenter of the earthquake is located 5 kms east of Fukui City.<br>The seismic intensity of shaking was estimated as JMAIS V-VI. A PGA value of 0.4 g was adopted by Seed et al (84)<br>Shonenji Temple and Agricultural Union sites are 500 m apart.<br>Kishida (1969) predicted the critical zone to be from 1-4 m based on:<br>Effective overburden pressure<2.0 kg/cm2<br>D_r<75%<br>Saturated coarse grained soil with U_c<10 and 0.074mm<D_{50}<2.0 mm<br>SPT energy was estimated as 70 % by Seed et al. (84)
**Summary of Data**	SPT

	Cetin et al. (2016)	Idriss&Boulanger (2010)	Seed et.al. (1984)		Cetin et al. (2016)	Idriss&Boulanger (2010)	Seed et.al. (1984)
Liquefied?	Yes	Yes	Yes	D_{50}	0.400 ± 0.050		0.45
Data Class	B			% Fines	0.0 ± 2.0	0.0	0
Critical Depth Range	3.9 - 13.0	13.1	13.0	% PI			
Depth to GWT (ft)	3.9 ± 0.3	3.9	4.0				
σ_v (psf)	912.7 ± 175.4	1566.4	1560.0	N	3.7 ± 1.3	8.0	8
σ_v' (psf)	631.1 ± 83.0	1002.5	1000.0	C_R	0.83	0.85	
a_{max} (g)	0.400 ± 0.120	0.40	0.400	C_S	1.00	1.00	
r_d	0.97 ± 0.044	0.96	0.970	C_B	1.00	1.00	
CSR	0.366 ± 0.114	0.390	0.395	C_E	1.17	1.17	1.17
Equivalent Magnitude	7.0	7.0		C_N	1.83	1.48	1.37
MSF		1.14	1.04	$(N_1)_{60}$	6.5 ± 2.3	11.8	13
CSRN		0.318	0.38				

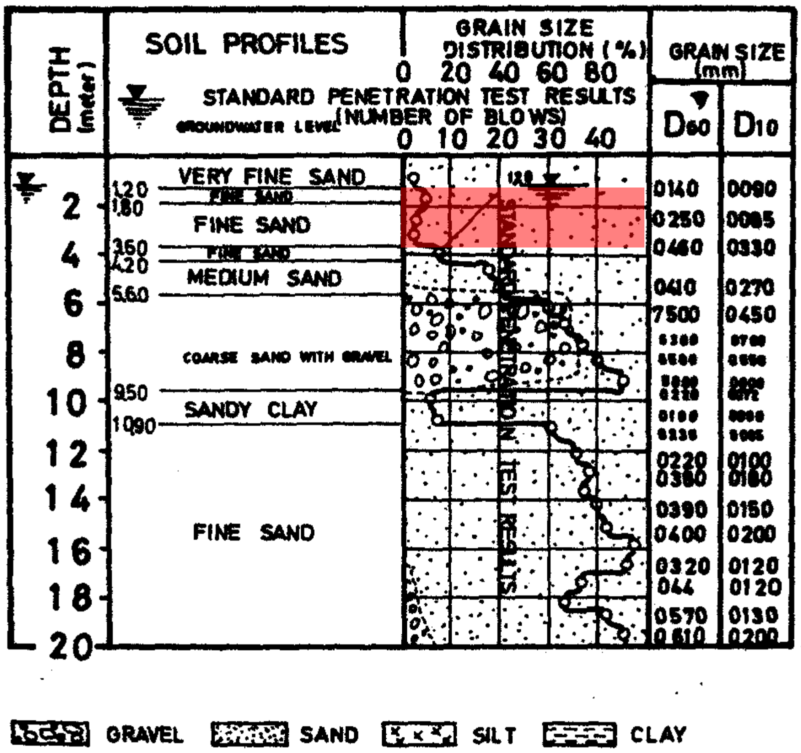

γ_m=100±3 pcf

γ_s=115±3 pcf

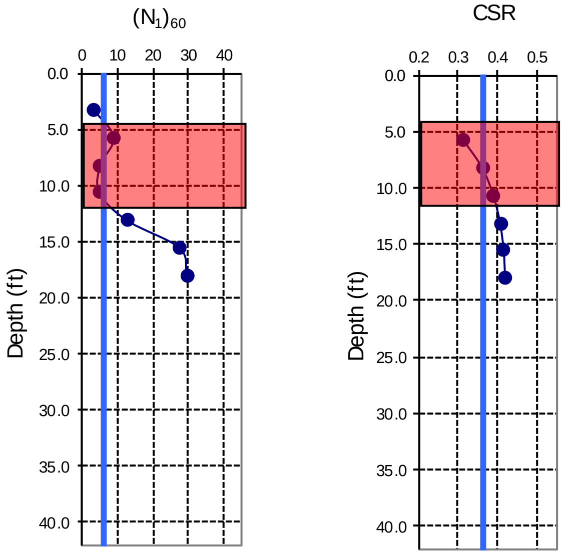

Depth (m)	Depth (ft)	CSR	N	C_N	C_R	$(N1)_{60}$	D60	D10
1.0	3.3	NA	2	2.00	0.73	3.4	0.14	0.01
1.8	5.7	0.315	5	2.00	0.78	***9.1***	0.23	0.07
2.5	8.2	0.363	3	1.85	0.82	***5.3***	0.46	0.33
3.3	10.7	0.391	3	1.68	0.86	***5.0***	0.41	0.27
4.0	13.1	0.408	8	1.55	0.89	12.9	7.50	0.45
4.8	15.6	0.418	18	1.45	0.91	27.8	-	-
5.5	18.0	0.422	20	1.37	0.94	29.8	-	-
					**Mean:**	6.5		
					**St. Dev.**	2.3		

**Case number:**	5
**Earthquake:**	1948 Fukui
**Magnitude:**	7.0 (Mw) USGS Centennial Earthquake Catalog
**Location:**	Takaya 45
**References:**	Kishida (1969)<br>Hamada et al (1989)
**Nature of Failure:**	Sand volcanoes were observed
**Comments:**	The epicenter of the earthquake is located 5 kms east of Fukui City.<br>The seismic intensity of shaking is estimated as JMAIS V-VI. A PGA value<br>0.35 g was adopted by Seed et al (84)<br>A paddy place, quickly draining.<br>Takaya 2 and 45 are approximately parallel to Kuzuryu river.<br>Kishida (1969) predicted the critical zone to be from 2-13 m based on:<br>Effective overburden pressure<2.0 kg/cm2<br>D_r<75%<br>Saturated coarse grained soil with U_c<10 and 0.074mm<D_{50}<2.0 mm<br>SPT energy was estimated as 78 % by Seed et al. (84)

Summary of Data

	Cetin et al. (2016)	Idriss&Boulanger (2010)	Seed et.al. (1984)		Cetin et al. (2016)	Idriss&Boulanger (2010)	Seed et.al. (1984)
Liquefied?	Yes	Yes	Yes	D_{50}	0.500 ± 0.050		0.65
Data Class	B			% Fines	3.3 ± 1.3	4.0	4
Critical Depth Range	12.3 - 40.0	24.6	23.0	% PI			
Depth to GWT (ft)	12.3 ± 0.3	12.1	12.0				
σ_v (psf)	3084.3 ± 579.8	2944.8	2760.0	N	16.2 ± 2.7	17.3	19
σ_v' (psf)	2220.0 ± 294.6	2172.1	2070.0	C_R	1.00	0.95	
a_{max} (g)	0.350 ± 0.105	0.35	0.350	C_S	1.00	1.00	
r_d	0.85 ± 0.115	0.90	0.950	C_B	1.00	1.00	
CSR	0.270 ± 0.090	0.283	0.290	C_E	1.30	1.30	1.30
Equivalent Magnitude	7.0	7.0		C_N	0.98	0.99	0.98
MSF		1.14	1.04	$(N_1)_{60}$	20.6 ± 3.4	21.1	24
CSRN		0.251	0.28				

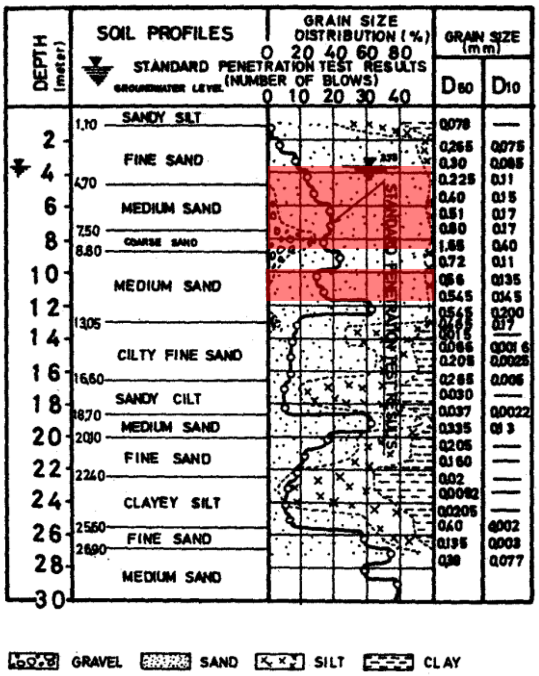

110±3 pcf

125±3 pcf

(1) TAKAYA TOWN 45-35

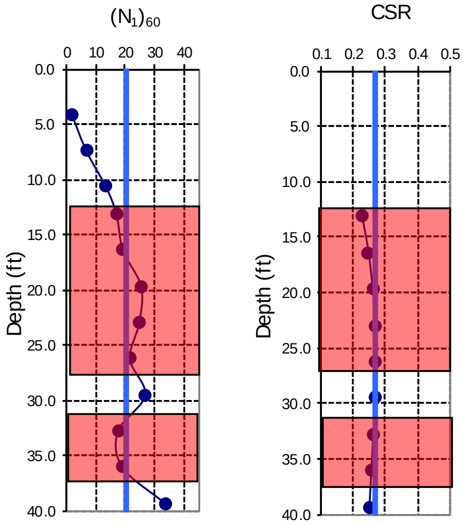

Depth (m)	Depth (ft)	CSR	N	C_N	C_R	$(N1)_{60}$	FC	silt	clay	gravel	D60	D10
1.3	4.1	NA	1	2.00	0.75	2.0	52.0	36	16	0	0.078	-
2.3	7.4	NA	4	1.61	0.81	6.8	8.0	8	0	0	0.265	0.075
3.3	10.7	NA	9	1.34	0.86	13.5	7.0	7	0	0	0.300	0.085
4.0	13.1	0.226	12	1.23	0.89	***17.0***	3.0	3	0	0	0.225	0.510
5.0	16.4	0.248	14	1.15	0.92	***19.2***	3.0	3	0	4	0.400	0.150
6.0	19.7	0.261	19	1.08	0.95	***25.4***	5.0	5	0	6	0.510	0.170
7.0	23.0	0.268	19	1.02	0.98	***24.7***	2.0	2	0	9	0.600	0.170
8.0	26.2	0.270	17	0.98	1.00	***21.5***	2.0	2	0	27	1.650	0.400
9.0	29.5	0.268	22	0.93	1.00	26.7	5.0	5	0	12	0.720	0.310
10.0	32.8	0.263	15	0.90	1.00	***17.5***	5.0	5	0	0	0.560	0.135
11.0	36.1	0.257	17	0.86	1.00	***19.1***	3.0	3	0	0	0.545	0.145
12.0	39.4	0.250	31	0.83	1.00	33.6	2.0	2	0	0	0.545	0.200
					**Mean:**	20.6	3.3					
					**St. Dev.**	3.4	1.3					

**Case number:**	6	
**Earthquake:**	1964 Niigata	
**Magnitude:**	7.6 (Mw)	http://ds.iris.edu/seismo-archives/quakes/1964niigata/
**Location:**	Aki City (Arayamotomachi)	
**References:**	Yasuda and Tohno (1988)	
**Nature of Failure:**	Sand boils	
**Comments:**	The epicenter of the earthquake is located some 35 miles north of the city.<br>At Arayamotomachi Town in Akita City, liquefaction was caused<br>in a long and narrow area by Niigata Earthquake.<br>The damage was concentrated in a small zone about 100 m in width and 500 m in<br>length, in an area of land recently reclaimed over an old river by filling with sand<br>from adjacent sand dunes. At a point near the center of the liquefied area, a<br>layer of very loose fine sand, with SPT N values of 0-6, forms a deposit<br>extending from the ground surface to about 6 m in depth. The water table is<br>at 1 m depth.<br>The maximum surface acceleration was assumed to be 0.09 g based on the<br>value recorded at the Akita Prefectural Government building 4 km from<br>Arayamotomachi Town.	
**Summary of Data**	SPT	

	Cetin et al. (2016)	Idriss&Boulanger (2010)	Seed et.al. (1984)		Cetin et al. (2016)	Idriss&Boulanger (2010)	Seed et.al. (1984)
Liquefied?	Yes	Yes		D_{50}	0.150 ± 0.050		
Data Class	A			% Fines	5.0 ± 2.0	5.0	
Critical Depth Range	3.3 - 18.0	10.8		% PI			
Depth to GWT (ft)	3.3 ± 0.3	3.3					
σ_v (psf)	1177.0 ± 284.0	1315.8		N	4.4 ± 2.7	2.6	
σ_v' (psf)	716.4 ± 132.4	856.3		C_R	0.86	0.85	
a_{max} (g)	0.090 ± 0.014	0.09		C_S	1.00	1.00	
r_d	0.94 ± 0.054	0.98		C_B	1.00	1.00	
CSR	0.090 ± 0.016	0.089		C_E	1.22	1.22	
Equivalent Magnitude	7.6	7.6		C_N	1.72	1.70	
MSF		0.97		$(N_1)_{60}$	7.9 ± 4.8	4.7	
CSRN		0.086					

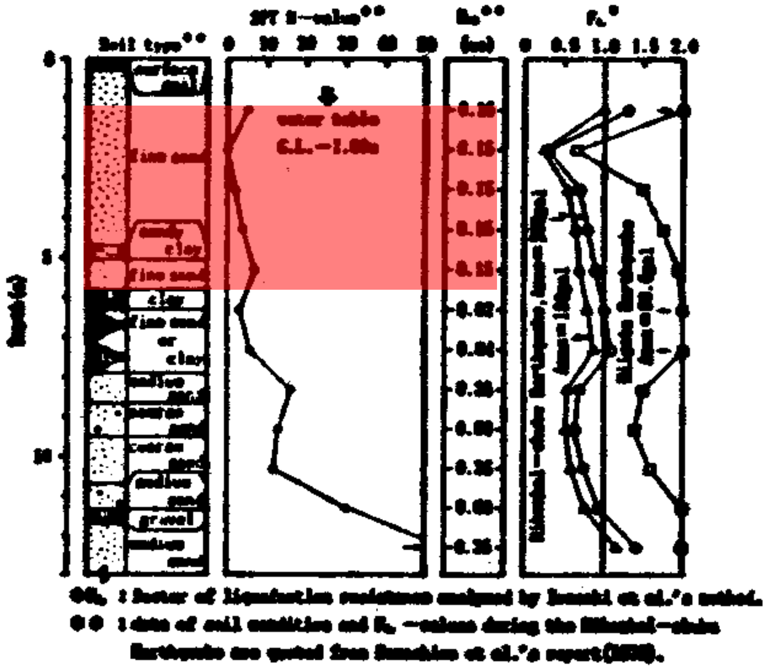

γ_m=100±3 pcf

γ_s=115±3 pcf

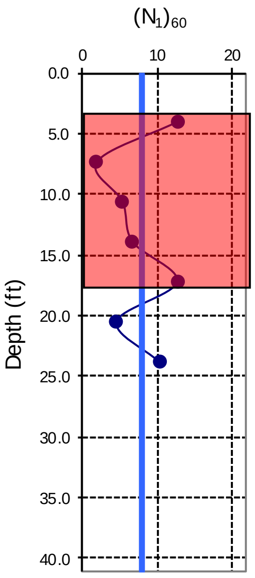

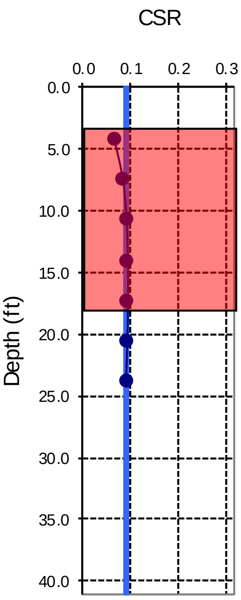

Depth (m)	Depth (ft)	CSR	N	C_N	C_R	$(N1)_{60}$	
1.3	4.1	0.065	7.0	2.00	0.75	***12.8***	fine sand
2.3	7.4	0.083	1.0	1.97	0.81	***1.9***	fine sand
3.3	10.7	0.090	3.0	1.72	0.86	***5.4***	fine sand
4.3	13.9	0.093	4.0	1.54	0.90	***6.7***	fine sand
5.3	17.2	0.093	8.0	1.41	0.93	***12.8***	fine sand
6.3	20.5	0.092	3.0	1.31	0.96	4.6	clay seam
7.3	23.8	0.089	7.0	1.23	0.98	10.3	fine sand or clay
					**Mean:**	7.9	
					**St. Dev.**	4.8	

**Case number:**	7
**Earthquake:**	1964 Niigata
**Magnitude:**	7.6 (Mw) http://ds.iris.edu/seismo-archives/quakes/1964niigata/
**Location:**	Niigata Cc17-1
**References:**	Kishida (1966)<br>Seed and Idriss (1966), "An analysis of soil liquefaction in the Niigata Eq."
**Nature of Failure:**	Along Shinano River many buildings tilted or subsided constructed on reclaimed land. Sand particles at ~ 5-7 m depth came out; sand volcanoes at various places.
**Comments:**	Niigata is located on the west coast of Japan where Shinano River enters the sea The river has bulit up a considerable thickness of alluvial sand deposits, which along the coast have been overlain by deposits of dune sand. In general the sand deposits underlying the city are relatively loose near the ground surface, but gets denser with increasing depths. The sand extents to a depth of ~100' and alluvial deposits to 200-300'. In the vicinity of river the ground is relatively flat.<br>The epicenter of the earthquake is located 35 miles north of the city.<br>From the seismograph, located in the basement of an apartment building it was estimated that the PGA at Niigata was in the order of 0.16 g. The soil below seismograph liquefied during the earthquake. It is interesting to note the marked change in form of record from a predominantly short period motion to a long period motion which occurred after about 8 seconds.<br>SPT values were taken both before and after the earthquake. N values before the earthquake were used for the analysis.<br>SPT energy was estimated as 65 % by Seed et al. (84)

**Summary of Data**	SPT						
	Cetin et al. (2016)	Idriss&Boulanger (2010)	Seed et.al. (1984)		Cetin et al. (2016)	Idriss&Boulanger (2010)	Seed et.al. (1984)
Liquefied?	Yes	Yes	Yes	D_{50}	0.200 ± 0.050		0.3
Data Class	B			% Fines	2.0 ± 2.0	2.0	2
Critical Depth Range	16.4 - 36.1	23.0	23.0	% PI			
Depth to GWT (ft)	3.0 ± 0.3	3.0	3.0				
σ_v (psf)	3089.6 ± 400.0	2756.9	2760.0	N	8.8 ± 2.3	8.0	8
σ_v' (psf)	1639.0 ± 202.0	1503.8	1510.0	C_R	1.00	0.95	
a_{max} (g)	0.160 ± 0.024	0.16	0.160	C_S	1.00	1.00	
r_d	0.80 ± 0.116	0.94	0.950	C_B	1.00	1.00	
CSR	0.157 ± 0.033	0.179	0.180	C_E	1.09	1.09	1.09
Equivalent Magnitude	7.6	7.6		C_N	1.14	1.20	1.14
MSF		0.97	1.00	$(N_1)_{60}$	10.9 ± 2.9	9.9	10
CSRN		0.178	0.18				

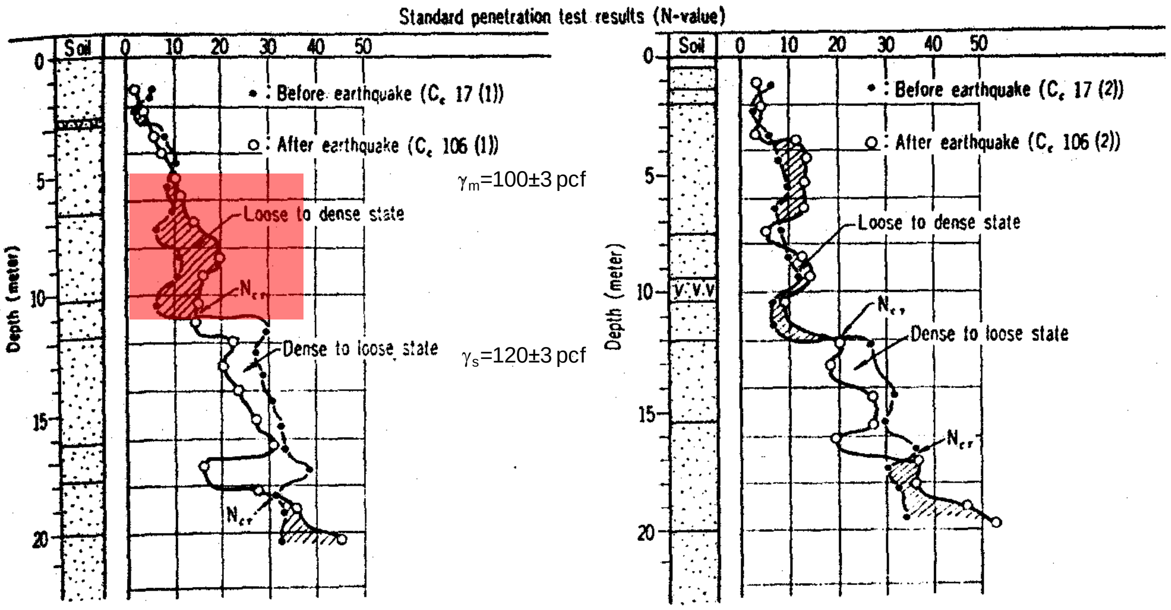

Fig. 5. Typical examples of standard penetration test results before and after Niigata earthquake (Location of boring is shown in Fig. 3)

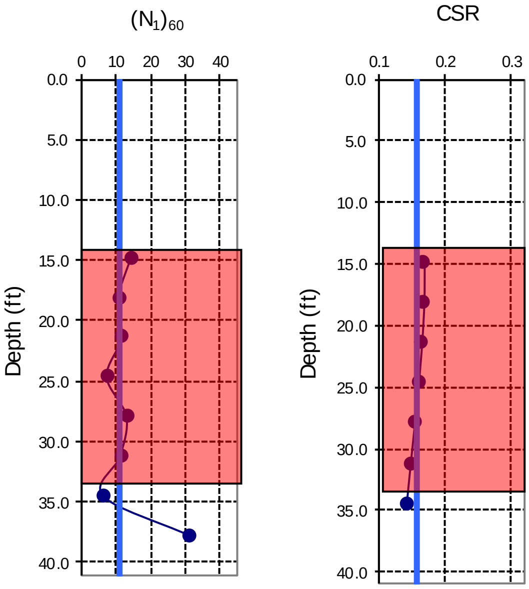

Depth (m)	Depth (ft)	CSR	N	C_N	C_R	$(N1)_{60}$
4.5	14.8	0.168	10	1.47	0.90	***14.5***
5.5	18.0	0.167	8	1.35	0.94	***11.0***
6.5	21.3	0.164	9	1.25	0.96	***11.8***
7.5	24.6	0.160	6	1.17	0.99	***7.6***
8.5	27.9	0.154	11	1.10	1.00	***13.3***
9.5	31.2	0.148	10	1.05	1.00	***11.5***
10.5	34.4	0.143	6	1.00	1.00	***6.6***
11.5	37.7	0.138	30	0.96	1.00	31.4
					**Mean:**	10.9
					**St. Dev.**	2.9

**Case number:**	8
**Earthquake:**	1964 Niigata
**Magnitude:**	7.6 (Mw) http://ds.iris.edu/seismo-archives/quakes/1964niigata/
**Location:**	Niigata Cc17-2
**References:**	Kishida (1966)<br>Seed and Idriss (1966), "An analysis of soil liquefaction in the Niigata Eq."
**Nature of Failure:**	In the Shinano River reclaimed land, many buildings tilted or subsided.<br>The sand particles at ~ 5-7 m depth came out; sand volcanoes at various places.
**Comments:**	Niigata is located on the west coast of Japan where Shinano River enters the sea.<br>The river has built up a considerable thickness of alluvial sand deposits, which<br>along the coast have been overlain by deposits of dune sand. In general the<br>sand deposits underlying the city are relatively loose near the ground surface,<br>but gets denser with increasing depth. The sand extents to a depth of ~100'<br>and alluvial deposits to 200-300'. In the vicinity of river the ground is relatively<br>flat.<br>The epicenter of the earthquake is located some 35 miles north of the city.<br>From the seismograph, located in the basement of an apartment building<br>it was estimated that the PGA at Niigata was in the order of 0.16 g. The soil below<br>seismograph liquefied during the earthquake. It is interesting to note the marked<br>change in form of record from a predominantly short period motion to a long<br>period motion which occurred after about 8 seconds.<br>SPT values were taken both before and after the earthquake. N values before<br>the earthquake were used for the analysis.<br>SPT energy was estimated as 65 % by Seed et al. (84)

**Summary of Data**	SPT						
	Cetin et al. (2016)	Idriss&Boulanger (2010)	Seed et.al. (1984)		Cetin et al. (2016)	Idriss&Boulanger (2010)	Seed et.al. (1984)
Liquefied?	Yes	Yes		D_{50}	0.200 ± 0.050		
Data Class	A			% Fines	2.0 ± 2.0	8.0	
Critical Depth Range	11.5 - 23.0	17.4		% PI			
Depth to GWT (ft)	3.0 ± 0.3	3.0					
σ_v (psf)	2006.9 ± 233.8	1775.3		N	8.0 ± 1.4	7.9	
σ_v' (psf)	1119.3 ± 119.2	898.1		C_R	0.93	0.95	
a_{max} (g)	0.160 ± 0.024	0.16		C_S	1.00	1.00	
r_d	0.90 ± 0.081	0.96		C_B	1.00	1.00	
CSR	0.168 ± 0.030	0.199		C_E	1.09	1.09	
Equivalent Magnitude	7.6	7.6		C_N	1.37	1.55	
MSF		0.97		$(N_1)_{60}$	11.1 ± 1.9	12.7	
CSRN		0.188					

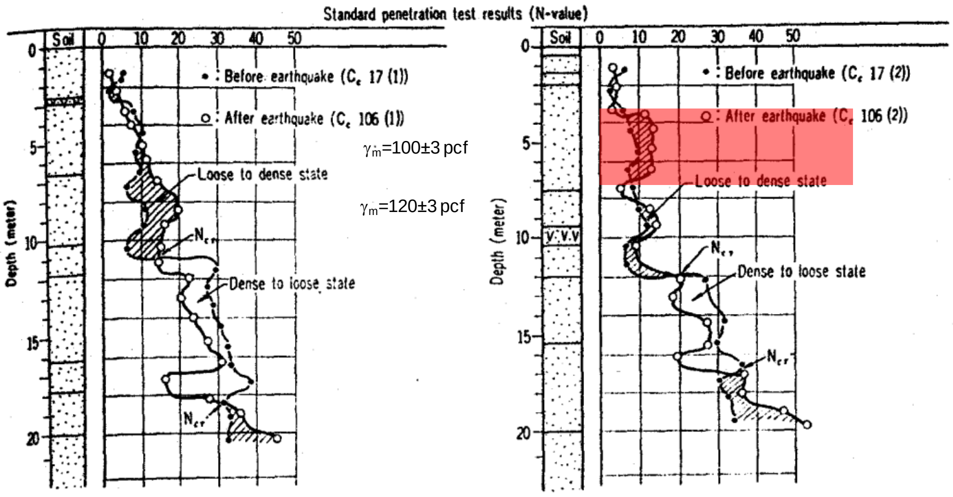

Fig. 5. Typical examples of standard penetration test results before and after Niigata earthquake (Location of boring is shown in Fig. 3)

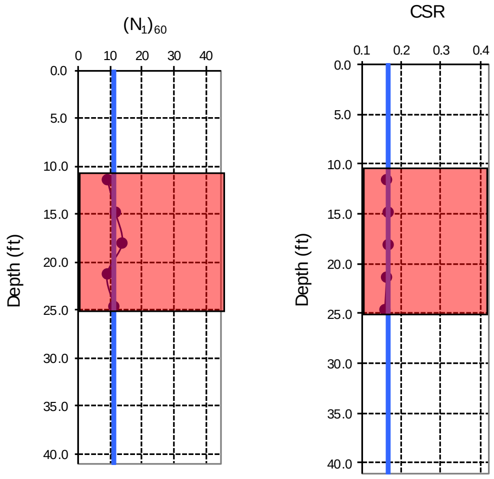

Depth (m)	Depth (ft)	CSR	N	C_N	C_R	$(N1)_{60}$
3.5	11.5	0.165	6	1.64	0.87	***9.3***
4.5	14.8	0.168	8	1.47	0.90	***11.6***
5.5	18.0	0.167	10	1.35	0.94	***13.8***
6.5	21.3	0.164	7	1.25	0.96	***9.2***
7.5	24.6	0.160	9	1.17	0.99	***11.4***
					**Mean:**	11.1
					**St. Dev.**	1.9

Despite the fact that it is sligthly below the bottom of the critical depth range, due to consistent trend in N values, N=9 is used to characterize the critical layer.

**Case number:**	9
**Earthquake:**	1964 Niigata
**Magnitude:**	7.6 (Mw) http://ds.iris.edu/seismo-archives/quakes/1964niigata/
**Location:**	Old Town-1
**References:**	Koizumi (1964)<br>Seed and Idriss (1966), "An analysis of soil liquefaction in the Niigata Eq."
**Nature of Failure:**	No surface evidence of liquefaction has been observed at Old Town section.
**Comments:**	Niigata is located on the west coast of Japan where Shinano River enters the sea<br>The river has built up a considerable thickness of alluvial sand deposits, which<br>along the coast have been overlain by deposits of dune sand. In general the<br>sand deposits underlying the city are relatively loose near the ground surface,<br>but gets denser with increasing depth. The sand extents to a depth of ~100'<br>and alluvial deposits to 200-300'. In the vicinity of river the ground is relatively<br>flat. Niigata city was below sea level about thousand years ago and the bay<br>was ditted with several small islands. The EQ was the first large vibration the<br>recent deposits had experienced.<br>The epicenter of the earthquake was located some 35 miles north of the city.<br>From the seismograph, located in the basement of an apartment building<br>it was estimated that the PGA at Niigata was in the order of 0.16 g. The soil below<br>seismograph liquefied during the earthquake. It is interesting to note the marked<br>change in form of record from a predominantly short period motion to a long<br>period motion which occurred after about 8 seconds.<br>Seed et al (84) estimated PGA not as 0.16 but 0.18 g probably due to the fact<br>the site did not liquefy so no de-amplification.<br>Seed et al (84) adopted a rod energy of 73 % (average Japanese energy value)<br>for non-liquefied sites and 65 % (Japanese lower bound) for liquefied sites.<br>Water table was at 1-2 m except dune sand.<br>SPT values were taken before the earthquake.<br>SPT energy was estimated as 73 % by Seed et al. (84)

**Summary of Data**	SPT						
	Cetin et al. (2016)	Idriss&Boulanger (2010)	Seed et.al. (1984)		Cetin et al. (2016)	Idriss&Boulanger (2010)	Seed et.al. (1984)
Liquefied?	No	No	No	D_{50}	0.200 ± 0.050		0.3
Data Class	A			% Fines	2.0 ± 2.0	2.0	2
Critical Depth Range	16.4 - 32.8	23.0	23.0	% PI			
Depth to GWT (ft)	6.0 ± 0.3	5.9	6.0				
σ_v (psf)	2985.8 ± 346.8	2756.9	2760.0	N	17.4 ± 0.5	18.0	18
σ_v' (psf)	1824.8 ± 181.5	1691.7	1700.0	C_R	0.99	0.95	
a_{max} (g)	0.180 ± 0.027	0.18	0.180	C_S	1.00	1.00	
r_d	0.87 ± 0.110	0.94	0.950	C_B	1.00	1.00	
CSR	0.167 ± 0.033	0.179	0.180	C_E	1.21	1.21	1.21
Equivalent Magnitude	7.6	7.6		C_N	1.08	1.10	1.08
MSF		0.97	1.00	$(N_1)_{60}$	22.3 ± 0.7	22.7	23.5
CSRN		0.178	0.18				

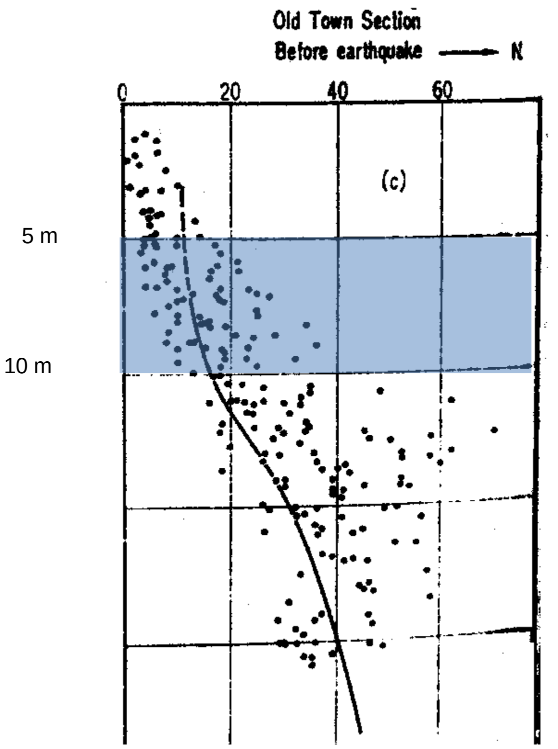

γ_m=110±3 pcf

γ_s=125±3 pcf

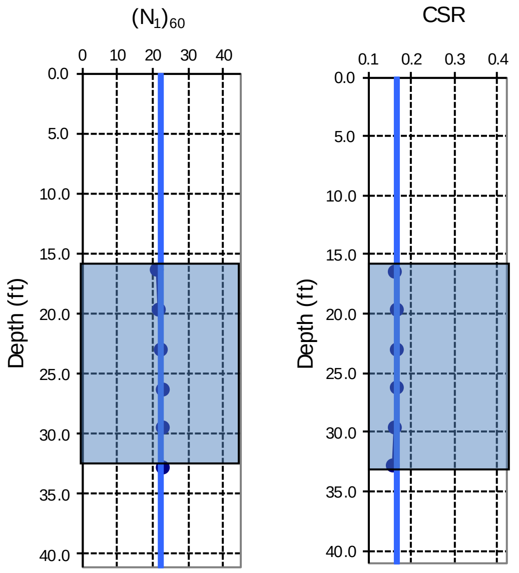

Depth (m)	Depth (ft)	CSR	N	C_N	C_R	$(N1)_{60}$
5.0	16.4	0.164	15	1.27	0.92	*21.2*
6.0	19.7	0.167	16	1.18	0.95	*21.7*
7.0	23.0	0.168	17	1.11	0.98	*22.2*
8.0	26.2	0.166	18	1.05	1.00	*22.8*
9.0	29.5	0.163	19	1.00	1.00	*22.9*
10.0	32.8	0.159	20	0.95	1.00	*23.0*
					**Mean:**	22.3
					**St. Dev.**	0.7

**Case number:**	10
**Earthquake:**	1964 Niigata
**Magnitude:**	7.6 (Mw) http://ds.iris.edu/seismo-archives/quakes/1964niigata/
**Location:**	Old Town-2
**References:**	Koizumi (1964)<br>Seed and Idriss (1966), "An analysis of soil liquefaction in the Niigata Eq."
**Nature of Failure:**	No surface evidence of liquefaction has been observed at Old Town section.
**Comments:**	Niigata is located on the west coast of Japan where Shinano River enters the sea<br>The river has built up a considerable thickness of alluvial sand deposits, which<br>along the coast have been overlain by deposits of dune sand. In general the<br>sand deposits underlying the city are relatively loose near the ground surface,<br>but gets denser with increasing depth. The sand extents to a depth of ~100'<br>and alluvial deposits to 200-300'. In the vicinity of river the ground is relatively<br>flat. Niigata city was below sea level about thousand years ago and the bay<br>was dotted with several small islands. The EQ was the first large vibration the<br>recent deposits had experienced.<br>The epicenter of the earthquake was located some 35 miles north of the city.<br>From the seismograph, located in the basement of an apartment building<br>it was estimated that the PGA at Niigata was in the order of 0.16 g. The soil below<br>seismograph liquefied during the earthquake. It is interesting to note the marked<br>change in form of record from a predominantly short period motion to a long<br>period motion which occurred after about 8 seconds.<br>Seed et al (84) estimated PGA not as 0.16 but 0.18 g probably due to the fact<br>the site did not liquefy so no de-amplification.<br>Seed et al (84) adopted a rod energy of 73 % (average Japanese energy value)<br>for non-liquefied sites and 65 % (Japanese lower bound) for liquefied sites.<br>Water table was at 1-2 m except dune sand.<br>SPT values were taken before the earthquake.<br>SPT energy was estimated as 73 % by Seed et al. (84)
**Summary of Data**	SPT

	Cetin et al. (2016)	Idriss&Boulanger (2010)	Seed et.al. (1984)		Cetin et al. (2016)	Idriss&Boulanger (2010)	Seed et.al. (1984)
Liquefied?	No	No	No	D_{50}	0.200 ± 0.050		0.3
Data Class	B			% Fines	2.0 ± 2.0	2.0	2
Critical Depth Range	32.8 - 42.7	33.1	33.0	% PI			
Depth to GWT (ft)	6.0 ± 0.3	5.9	6.0				
σ_v (psf)	4626.2 ± 226.8	3968.2	3960.0	N	24.7 ± 3.0	20.0	20
σ_v' (psf)	2646.3 ± 141.9	2276.5	2270.0	C_R	1.00	1.00	
a_{max} (g)	0.180 ± 0.027	0.18	0.180	C_S	1.00	1.00	
r_d	0.74 ± 0.158	0.90	0.910	C_B	1.00	1.00	
CSR	0.152 ± 0.040	0.184	0.185	C_E	1.21	1.21	1.21
Equivalent Magnitude	7.6	7.6		C_N	0.89	0.97	0.94
MSF		0.97	1.00	$(N_1)_{60}$	26.7 ± 3.2	23.5	22.5
CSRN		0.191	0.19				

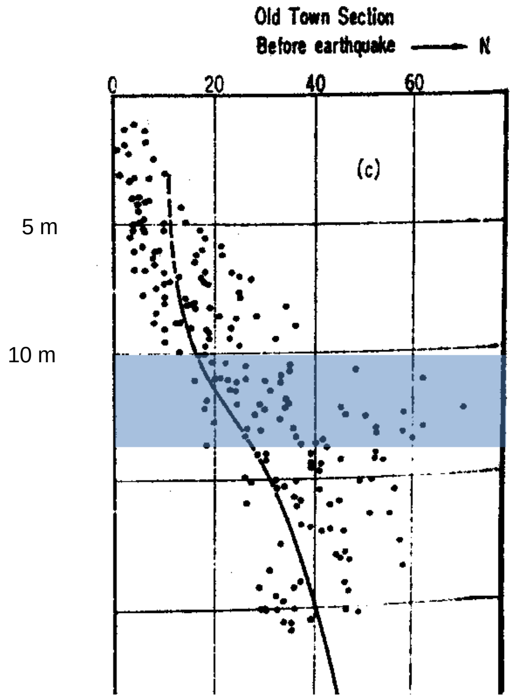

γ_m=110±3 pcf

γ_s=125±3 pcf

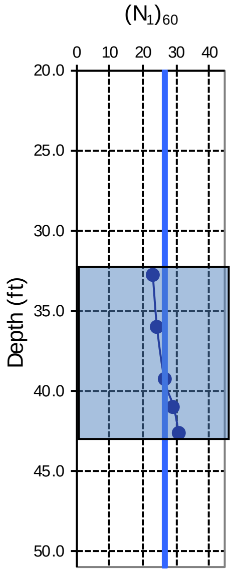

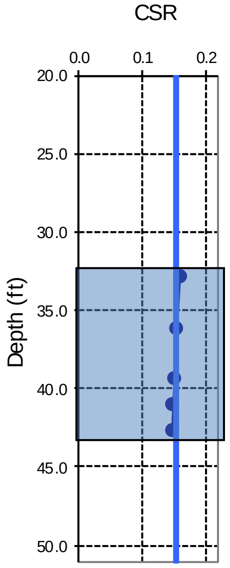

Depth (m)	Depth (ft)	CSR	N	C_N	C_R	$(N1)_{60}$
10.0	32.8	0.159	20	0.95	1.00	***23.0***
11.0	36.1	0.154	22	0.91	1.00	***24.2***
12.0	39.4	0.150	25	0.88	1.00	***26.5***
12.5	41.0	0.148	28	0.86	1.00	***29.1***
13.0	42.7	0.146	30	0.85	1.00	***30.7***
					**Mean:**	26.7
					**St. Dev.**	3.2

**Case number:**	11
**Earthquake:**	1964 Niigata
**Magnitude:**	7.6 (Mw) http://ds.iris.edu/seismo-archives/quakes/1964niigata/
**Location:**	Rail Road-1
**References:**	Koizumi (1964)<br>Seed and Idriss (1966), "An analysis of soil liquefaction in the Niigata Eq."
**Nature of Failure:**	The SPT measurements before and after the earthquake are used for predicting liquefaction in conjunction with the surface evidence of it.
**Comments:**	Niigata is located on the west coast of Japan where Shinano River enters the sea The river has built up a considerable thickness of alluvial sand deposits, which along the coast have been overlain by deposits of dune sand. In general the sand deposits underlying the city are relatively loose near the ground surface, but gets denser with increasing depth. The sand extents to a depth of ~100' and alluvial deposits to 200-300'. In the vicinity of river the ground is relatively flat. Niigata city was below sea level about thousand years ago and the bay was dotted with several small islands. The EQ was the first large vibration the recent deposits had experienced.<br>The epicenter of the earthquake is located some 35 miles north of the city.<br>From the seismograph, located in the basement of an apartment building it was estimated that the PGA at Niigata was of the order of 0.16 g. The soil below seismograph liquefied during the earthquake. It is interesting to note the marked change in form of record from a predominantly short period motion to a long period motion which occurred after about 8 seconds.<br>Seed et al (84) adopted a rod energy of 73 % (average Japanese energy value) for non-liquefied sites and 65 % (Japanese lower bound) for liquefied sites.<br>Water table was at 1-2 m except dune sand.<br>SPT values were taken before and after the earthquake.<br>SPT energy % was estimated as 65 % by Seed et al. (84)

Summary of Data SPT

	Cetin et al. (2016)	Idriss&Boulanger (2010)	Seed et.al. (1984)		Cetin et al. (2016)	Idriss&Boulanger (2010)	Seed et.al. (1984)
Liquefied?	Yes	Yes	Yes	D_{50}	0.200 ± 0.050		0.3
Data Class	A			% Fines	2.0 ± 2.0	2.0	2
Critical Depth Range	16.4 - 32.8	33.1	33.0	% PI			
Depth to GWT (ft)	3.0 ± 0.3	3.0	3.0				
σ_v (psf)	3030.8 ± 348.0	3968.2	3960.0	N	9.4 ± 1.1	10.0	10
σ_v' (psf)	1682.6 ± 183.8	2088.5	2090.0	C_R	0.99	1.00	
a_{max} (g)	0.160 ± 0.024	0.16	0.160	C_S	1.00	1.00	
r_d	0.85 ± 0.110	0.90	0.910	C_B	1.00	1.00	
CSR	0.160 ± 0.032	0.178	0.180	C_E	1.09	1.09	1.09
Equivalent Magnitude	7.6	7.6		C_N	1.12	1.01	0.98
MSF		0.97	1.00	$(N_1)_{60}$	11.3 ± 1.3	11.0	10.5
CSRN		0.182	0.18				

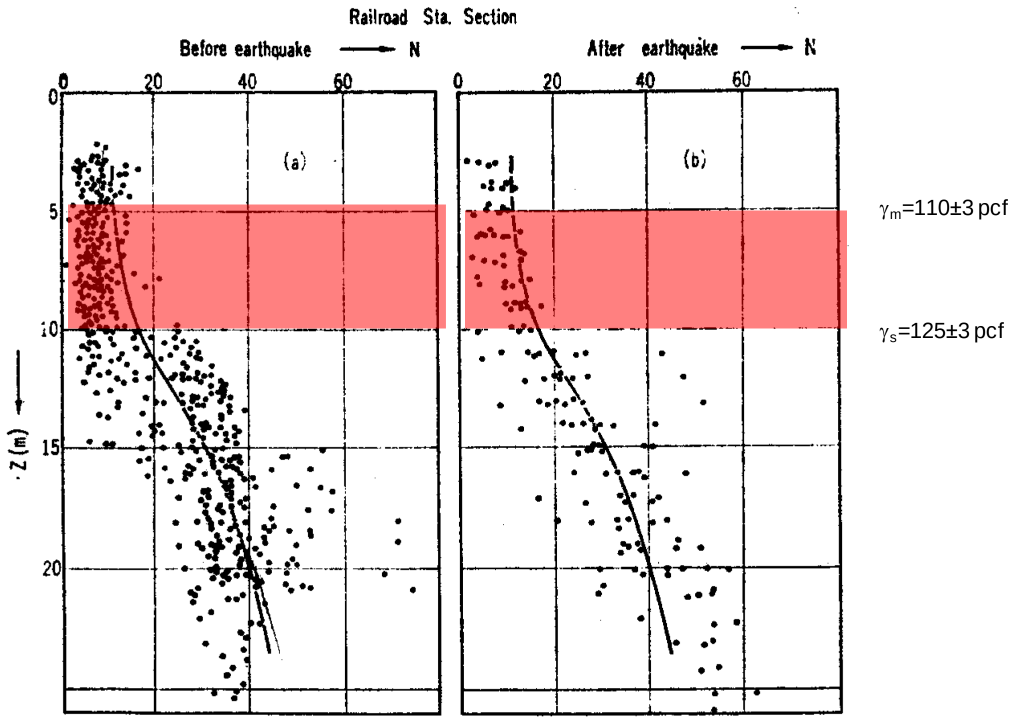

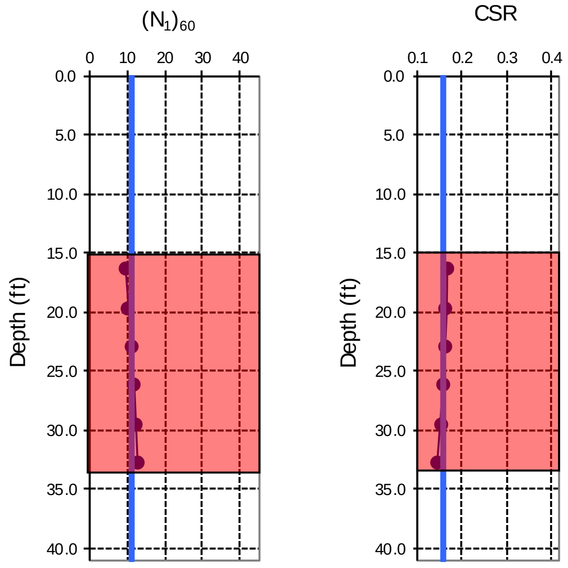

Depth (m)	Depth (ft)	CSR	N	C_N	C_R	$(N1)_{60}$
5.0	16.4	0.166	7	1.35	0.92	***9.5***
6.0	19.7	0.165	8	1.24	0.95	***10.3***
7.0	23.0	0.162	9	1.16	0.98	***11.1***
8.0	26.2	0.158	10	1.09	1.00	***11.9***
9.0	29.5	0.153	11	1.03	1.00	***12.4***
10.0	32.8	0.147	12	0.98	1.00	***12.9***
					**Mean:**	11.3
					**St. Dev.**	1.3

**Case number:**	12
**Earthquake:**	1964 Niigata
**Magnitude:**	7.6 (Mw) http://ds.iris.edu/seismo-archives/quakes/1964niigata/
**Location:**	Rail Road-2
**References:**	Koizumi (1964)<br>Seed and Idriss (1966), "An analysis of soil liquefaction in the Niigata Eq."
**Nature of Failure:**	The SPT measurements before and after the earthquake were used for predicting liquefaction in conjunction with the surface evidence of it.
**Comments:**	Niigata is located on the west coast of Japan where Shinano River enters the sea The river has built up a considerable thickness of alluvial sand deposits, which along the coast have been overlain by deposits of dune sand. In general the sand deposits underlying the city are relatively loose near the ground surface, but gets denser with increasing depth. The sand extents to a depth of ~100' and alluvial deposits to 200-300'. In the vicinity of river the ground is relatively flat. Niigata city was below sea level about thousand years ago and the bay was ditted with several small islands. The EQ was the first large vibration the recent deposits had experienced.<br>The epicenter of the earthquake was located some 35 miles north of the city. From the seismograph, located in the basement of an apartment building it was estimated that the PGA at Niigata was of the order of 0.16 g. The soil below seismograph liquefied during the earthquake. It is interesting to note the marked change in form of record from a predominantly short period motion to a long period motion which occurred after about 8 seconds.<br>Seed et al (84) adopted a rod energy of 73 % (average Japanese energy value) for non-liquefied sites and 65 % (Japanese lower bound) for liquefied sites.<br>Water table was at 1-2 m except dune sand.<br>SPT values were taken before and after the earthquake.<br>SPT energy was estimated as 65 % by Seed et al. (84)

Summary of Data

	Cetin et al. (2016)	Idriss&Boulanger (2010)	Seed et.al. (1984)		Cetin et al. (2016)	Idriss&Boulanger (2010)	Seed et.al. (1984)
Liquefied?	No/Yes	Marginal	No/Yes	D_{50}	0.200 ± 0.050		0.3
Data Class	B		C	% Fines	2.0 ± 2.0	2.0	2
Critical Depth Range	29.5 - 36.1	33.1	33.0	% PI			
Depth to GWT (ft)	3.0 ± 1.0	3.0	3.0				
σ_v (psf)	4056.0 ± 164.3	3968.2	3960.0	N	16.0 ± 1.9	16.0	16
σ_v' (psf)	2196.0 ± 122.5	2088.5	2090.0	C_R	1.00	1.00	
a_{max} (g)	0.160 ± 0.024	0.16	0.160	C_S	1.00	1.00	
r_d	0.77 ± 0.140	0.90	0.910	C_B	1.00	1.00	
CSR	0.147 ± 0.035	0.178	0.180	C_E	1.09	1.09	1.09
Equivalent Magnitude	7.6	7.6		C_N	0.98	1.01	0.98
MSF		0.97	1.00	$(N_1)_{60}$	17.1 ± 2.0	17.5	17
CSRN		0.182	0.18				

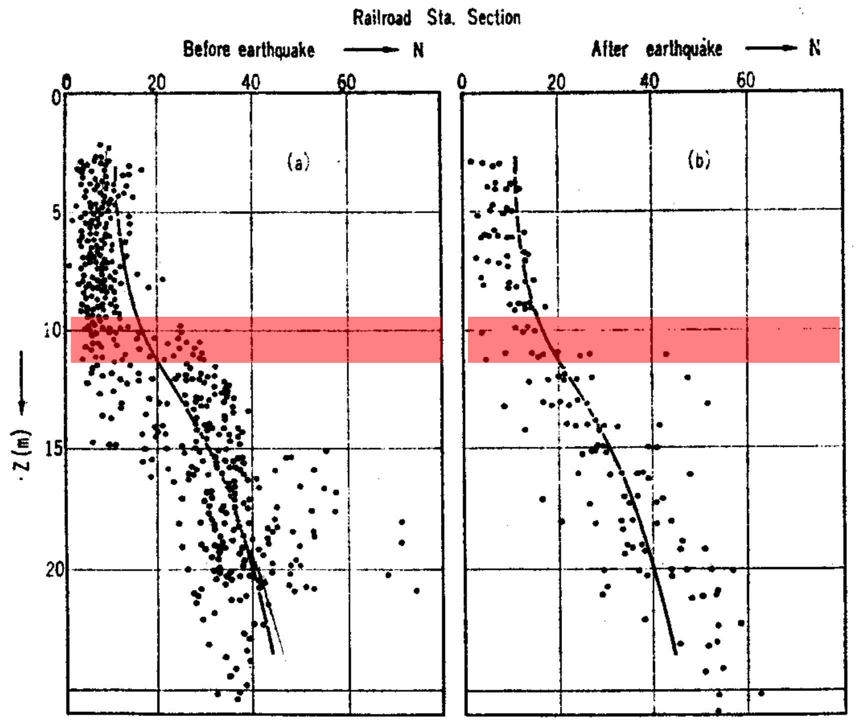

γ_m=110±3 pcf

γ_s=125±3 pcf

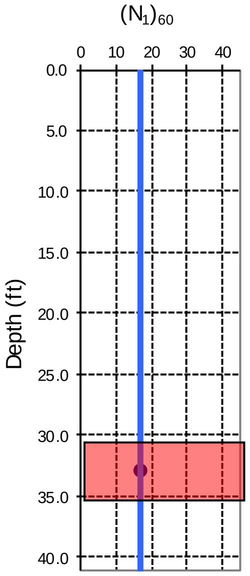

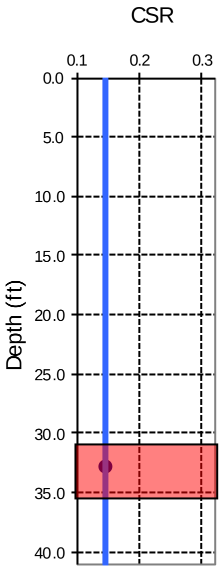

Depth (m)	Depth (ft)	CSR	N	C_N	C_R	$(N1)_{60}$
10.0	32.8	0.147	16	0.98	1.00	***17.1***
					**Mean:**	17.1
					**St. Dev.**	2.0

**Case number:**	13
**Earthquake:**	1964 Niigata
**Magnitude:**	7.6 (Mw) http://ds.iris.edu/seismo-archives/quakes/1964niigata/
**Location:**	River Site
**References:**	Ishihara et. al. (1979)<br>Seed and Idriss (1966), "An analysis of soil liquefaction in the Niigata Eq."
**Nature of Failure:**	Surface cracking and ejection of sand and water; flooding of sand and water over the ground surface.
**Comments:**	Niigata is located on the west coast of Japan where Shinano River enters the sea The river has built up a considerable thickness of alluvial sand deposits, which along the coast have been overlain by deposits of dune sand. In general the sand deposits underlying the city are relatively loose near the ground surface, but gets denser with increasing depth. The sand extents to a depth of ~100' and alluvial deposits to 200-300'. In the vicinity of river the ground is relatively flat. Niigata city was below sea level about thousand years ago and the bay was ditted with several small islands. The EQ was the first large vibration the recent deposits had experienced.<br>The River Site was located ~ 10 m from the bank of the Shinano river. The site was reclaimed by in 1955 by constructing a dike along the river channel.<br>The top 1 m consists of uncompacted fill dumped in air. Undensified fine sand dumped through water between depth of 1-3m. From 3-5m overconsolidated medium sand deposits. Fluvial river deposits consisting of medium sand with occasional silt lenses. Below 15 m a silt layer underlain by uniform coarse sand. The epicenter of the earthquake was located some 35 miles north of the city.<br>From the seismograph, located in the basement of an apartment building it was estimated that the PGA at Niigata was of the order of 0.16 g. The soil below seismograph liquefied during the earthquake. It is interesting to note the marked change in form of record from a predominantly short period motion to a long period motion which occurred after about 8 seconds.<br>SPT values were taken before and after the earthquake.<br>SPT energy was estimated as 65 % by Seed et al. (84)

Summary of Data

	Cetin et al. (2016)	Idriss&Boulanger (2010)	Seed et.al. (1984)		Cetin et al. (2016)	Idriss&Boulanger (2010)	Seed et.al. (1984)
Liquefied?	Yes	Yes	Yes	D_{50}	0.400 ± 0.050		0.4
Data Class	A			% Fines	0.0 ± 2.0	0.0	0
Critical Depth Range	6.6 - 19.7	15.1	15.0	% PI			
Depth to GWT (ft)	2.0 ± 0.3	2.0	2.0				
σ_v (psf)	1423.9 ± 243.0	1796.1	1800.0	N	4.0 ± 1.2	6.0	6
σ_v' (psf)	727.8 ± 110.6	981.6	990.0	C_R	0.89	0.95	
a_{max} (g)	0.160 ± 0.024	0.16	0.160	C_S	1.00	1.00	
r_d	0.93 ± 0.064	0.97	0.970	C_B	1.00	1.00	
CSR	0.189 ± 0.032	0.183	0.185	C_E	1.09	1.09	1.09
Equivalent Magnitude	7.6	7.6		C_N	1.71	1.52	1.38
MSF		0.97	1.00	$(N_1)_{60}$	6.6 ± 1.9	9.4	9
CSRN		0.176	0.19				

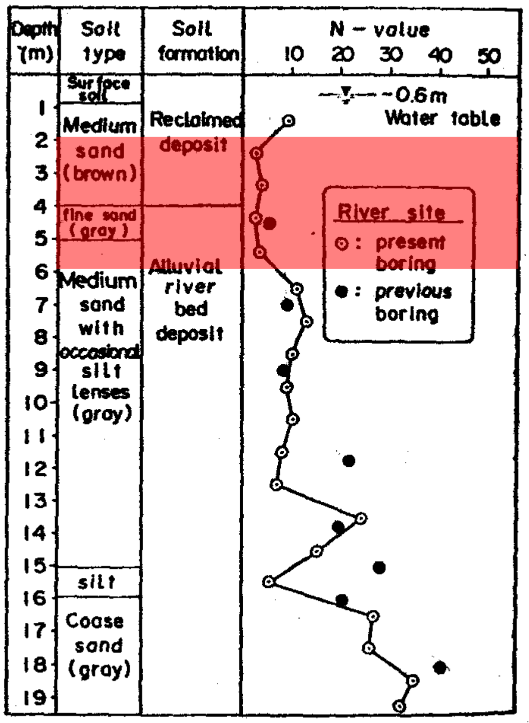

γ_m=100±3 pcf

γ_s=110±3 pcf

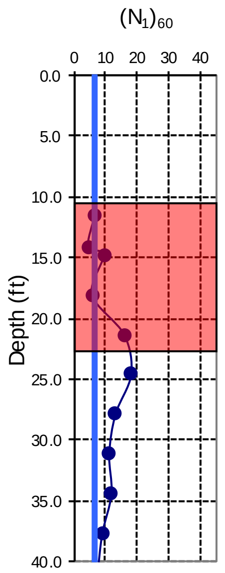

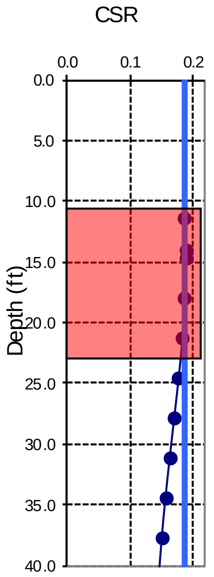

Depth (m)	Depth (ft)	CSR	N	C_N	C_R	$(N1)_{60}$
2.5	8.2	0.179	3	2.00	0.82	***5.4***
3.5	11.5	0.188	4	1.80	0.87	***6.8***
4.3	14.1	0.190	3	1.65	0.90	***4.9***
4.5	14.8	0.190	6	1.62	0.90	***9.6***
5.5	18.0	0.188	4	1.48	0.94	***6.1***
6.5	21.3	0.183	11	1.38	0.96	*15.9*
7.5	24.6	0.177	13	1.29	0.99	*18.1*
8.5	27.9	0.171	10	1.22	1.00	*13.3*
9.5	31.2	0.164	9	1.15	1.00	*11.3*
10.5	34.4	0.157	10	1.10	1.00	*12.0*
11.5	37.7	0.152	8	1.06	1.00	*9.2*
12.5	41.0	0.147	7	1.01	1.00	*7.8*
					**Mean:**	6.6
					**St. Dev.**	1.9

**Case number:**	14
**Earthquake:**	1964 Niigata
**Magnitude:**	7.6 (Mw) http://ds.iris.edu/seismo-archives/quakes/1964niigata/
**Location:**	Road Site
**References:**	Ishihara et. al. (1979)<br>Seed and Idriss (1966), "An analysis of soil liquefaction in the Niigata Eq."
**Nature of Failure:**	No surface evidence of liquefaction
**Comments:**	Niigata is located on the west coast of Japan where Shinano River enters the sea The river has built up a considerable thickness of alluvial sand deposits, which along the coast have been overlain by deposits of dune sand. In general the sand deposits underlying the city are relatively loose near the ground surface, but gets denser with increasing depth. The sand extents to a depth of ~100' and alluvial deposits to 200-300'. In the vicinity of river the ground is relatively flat. Niigata city was below sea level about thousand years ago and the bay was ditted with several small islands. The EQ was the first large vibration the recent deposits had experienced.<br>The Road Site was located on level ground adjacent to the road ~ 350 m from the Shinano River bank. The top 1.5 m consists of surface soil which is underlain to a depth of 4 m by silt and sandy silt. Below 4 m the soil profile consists of alternate layers of medium sand and silt to a depth of 16 m.<br>The epicenter of the earthquake was located some 35 miles north of the city.<br>From the seismograph, located in the basement of an apartment building it was estimated that the PGA at Niigata was of the order of 0.16 g. The soil below seismograph liquefied during the earthquake. It is interesting to note the marked change in form of record from a predominantly short period motion to a long period motion which occurred after about 8 seconds.<br>Seed et al (84) estimated PGA for the site a little higher (0.18 g) than the recorded one probably due to no de-amplification at non-liquefied sites.<br>Seed et al (84) adopted a rod energy of 73 % (average Japanese energy value) for non-liquefied sites and 65 % (Japanese lower bound) for liquefied sites.<br>SPT values were taken before the earthquake.<br>SPT energy was estimated as 65 % by Seed et al. (84)

Summary of Data

	Cetin et al. (2016)	Idriss&Boulanger (2010)	Seed et.al. (1984)		Cetin et al. (2016)	Idriss&Boulanger (2010)	Seed et.al. (1984)
Liquefied?	No	No	No	D_{50}	0.360 ± 0.129		0.36
Data Class	A			% Fines	0.0 ± 2.0	0.0	0
Critical Depth Range	13.1 - 29.5	20.0	20.0	% PI			
Depth to GWT (ft)	8.2 ± 0.4	7.9	8.0				
σ_v (psf)	2460.6 ± 345.0	2401.8	2400.0	N	12.1 ± 3.2	12.0	12
σ_v' (psf)	1641.7 ± 177.8	1649.9	1650.0	C_R	0.96	0.95	
a_{max} (g)	0.180 ± 0.027	0.18	0.180	C_S	1.00	1.00	
r_d	0.88 ± 0.097	0.95	0.960	C_B	1.00	1.00	
CSR	0.154 ± 0.029	0.162	0.165	C_E	1.09	1.09	1.09
Equivalent Magnitude	7.6	7.6		C_N	1.14	1.13	1.09
MSF		0.97	1.00	$(N_1)_{60}$	14.4 ± 3.8	14.1	14.5
CSRN		0.162	0.17				

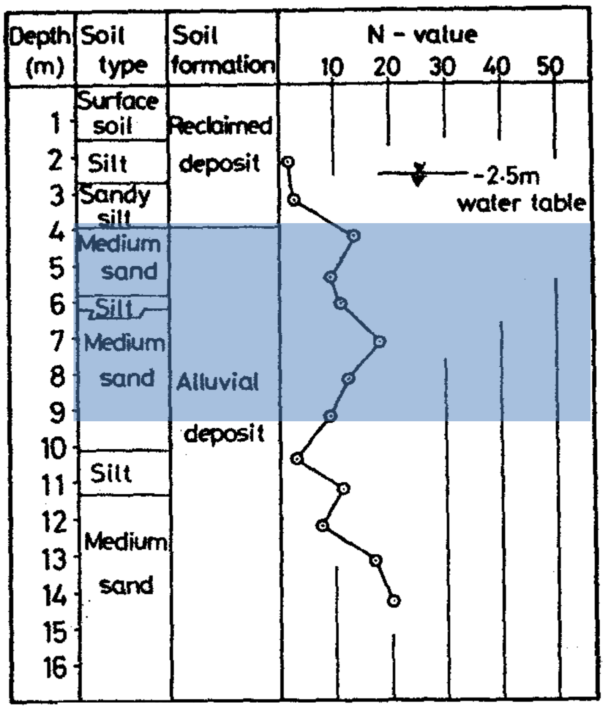

γ_m=100±3 pcf

γ_s=125±3 pcf

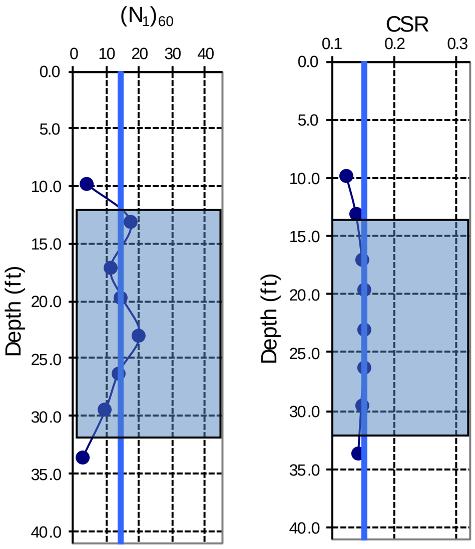

Depth (m)	Depth (ft)	CSR	N	C_N	C_R	$(N1)_{60}$
3.0	9.8	0.125	3	1.51	0.85	4.2
4.0	13.1	0.141	13	1.37	0.89	***17.2***
5.2	17.1	0.150	9	1.24	0.93	***11.3***
6.0	19.7	0.153	12	1.17	0.95	***14.6***
7.0	23.0	0.154	17	1.10	0.98	***20.0***
8.0	26.2	0.152	12	1.04	1.00	***13.6***
9.0	29.5	0.149	9	0.99	1.00	***9.7***
10.3	33.6	0.144	3	0.94	1.00	3.1
					**Mean:**	14.4
					**St. Dev.**	3.8

**Case number:**	15
**Earthquake:**	1964 Niigata
**Magnitude:**	7.6 (Mw) http://ds.iris.edu/seismo-archives/quakes/1964niigata/
**Location:**	Showa Br 2
**References:**	Takada et al (1965) in Japanese Ishihara et. al. (1979) Fear et al. (1995)<br>Seed and Idriss (1966), "An analysis of soil liquefaction in the Niigata Eq."
**Nature of Failure:**	The deck spans G3 to G7 collapsed.The left end of the bridge was separated from the bank; piles in the piers deformed.
**Comments:**	Niigata is located on the west coast of Japan where Shinano River enters the sea The river has built up a considerable thickness of alluvial sand deposits, which along the coast have been overlain by deposits of dune sand. In general the sand deposits underlying the city are relatively loose near the ground surface, but gets denser with increasing depth. The sand extents to a depth of ~100' and alluvial deposits to 200-300'. In the vicinity of river the ground is relatively flat. Niigata city was below sea level about thousand years ago and the bay was ditted with several small islands. The EQ was the first large vibration the recent deposits had experienced.<br>Showa Bridge experienced damage during Niigata Earthquake.<br>Coarse Sand Layer : the most recent strata; approximately 13 m thick at the left bank, decreasing to ~ 5 m at the right bank; a mixture of silt, loam, small gravel and organic material.<br>Medium Sand Layer: 15 m thick at the right bank (top of strta just more than 5m deep) increasing in depth and decreasing in thickness from the right to left bank; approximately 3 m thick at the left bank; some organic material, coarse sand, fine sand and iron sand, causing variations in N.<br>Fine Sand Layer: Deeper than 16-18 m; dark blue gray color; some parts contain silt and iron sand; N>30<br>The epicenter of the earthquake was located some 35 miles north of the city.<br>From the seismograph, located in the basement of an apartment building it was estimated that the PGA at Niigata was of the order of 0.16 g. The soil below seismograph liquefied during the earthquake. It is interesting to note the marked change in form of record from a predominantly short period motion to a long period motion which occurred after about 8 seconds.<br>SPT values were taken before and after the earthquake.<br>SPT energy was estimated as 65 % by Seed et al. (84)

Summary of Data

	Cetin et al. (2016)	Idriss&Boulanger (2010)	Seed et.al. (1984)		Cetin et al. (2016)	Idriss&Boulanger (2010)	Seed et.al. (1984)
Liquefied?	Yes	Yes	Yes	D_{50}	0.400 ± 0.050		0.4
Data Class	A			% Fines	10.0 ± 2.0	10.0	10
Critical Depth Range	4.5 - 20.0	14.1	14.0	% PI			
Depth to GWT (ft)	0.0 ± 0.3	0.0	0.0				
σ_v (psf)	1470.0 ± 312.2	1670.8	1680.0	N	4.1 ± 0.2	4.0	4
σ_v' (psf)	705.6 ± 154.4	814.5	810.0	C_R	0.88	0.95	
a_{max} (g)	0.160 ± 0.024	0.16	0.160	C_S	1.00	1.00	
r_d	0.90 ± 0.061	0.97	0.970	C_B	1.00	1.00	
CSR	0.195 ± 0.033	0.210	0.210	C_E	1.09	1.09	1.09
Equivalent Magnitude	7.6	7.6		C_N	1.73	1.70	1.50
MSF		0.97	1.00	$(N_1)_{60}$	6.8 ± 0.3	7.0	6.5
CSRN		0.199	0.21				

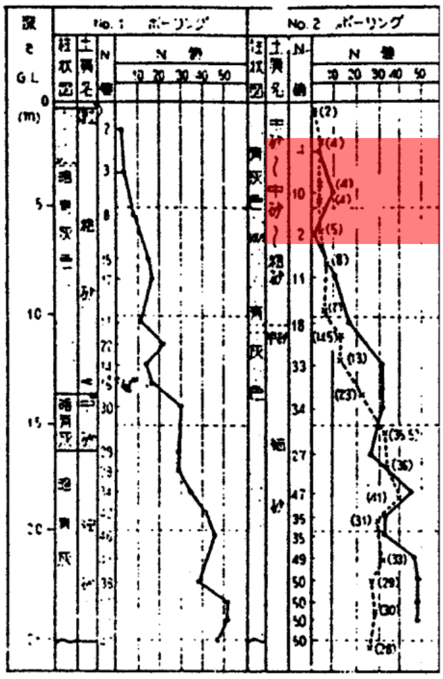

γ_m=120±3 pcf

γ_s=120±3 pcf

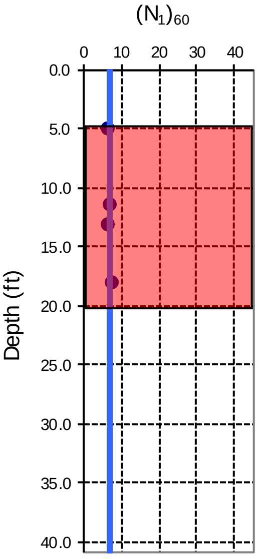

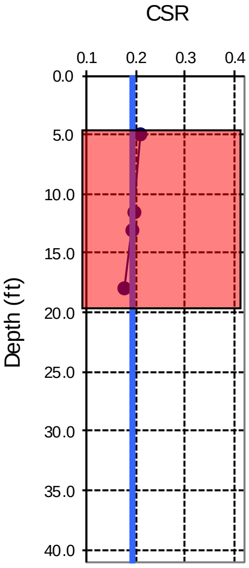

Depth (m)	Depth (ft)	CSR	N	C_N	C_R	$(N1)_{60}$
1.5	4.9	0.210	4	2.00	0.77	***6.7***
3.5	11.5	0.196	4	1.79	0.87	***6.8***
4.0	13.1	0.192	4	1.67	0.89	***6.5***
5.5	18.0	0.179	5	1.43	0.94	***7.3***
					**Mean:**	6.8
					**St. Dev.**	0.3

**Case number:**	16
**Earthquake:**	1964 Niigata
**Magnitude:**	7.6 (Mw) http://ds.iris.edu/seismo-archives/quakes/1964niigata/
**Location:**	Showa Br 4
**References:**	Takada et al (1965) in Japanese Fear et al. (1995)<br>Seed and Idriss (1966), "An analysis of soil liquefaction in the Niigata Eq."
**Nature of Failure:**	No damage to little damage.
**Comments:**	Niigata is located on the west coast of Japan where Shinano River enters the sea<br>The river has built up a considerable thickness of alluvial sand deposits, which<br>along the coast have been overlain by deposits of dune sand. In general the<br>sand deposits underlying the city are relatively loose near the ground surface,<br>but gets denser with increasing depth. The sand extents to a depth of ~100'<br>and alluvial deposits to 200-300'. In the vicinity of river the ground is relatively<br>flat. Niigata city was below sea level about thousand years ago and the bay<br>was dotted with several small islands. The EQ was the first large vibration the<br>recent deposits had experienced.<br>Showa Bridge experienced damage during The Niigata Earthquake.<br>Coarse Sand Layer : the most recent strata; approximately 13 m thick at the<br>left bank, decreasing to ~ 5 m at the right bank; a mixture of silt, loam, small<br>gravel and organic material.<br>Medium Sand Layer: 15 m thick at the right bank (top of strta just more than 5m<br>deep) increasing in depth and decreasing in thickness from the right to left<br>bank; approximately 3 m thick at the left bank; some organic material, coarse<br>sand, fine sand and iron sand, causing variations in N.<br>Fine Sand Layer: Deeper than 16-18 m; dark blue gray color; some parts<br>contain silt and iron sand; N>30<br>The epicenter of the earthquake was located some 35 miles north of the city.<br>From the seismograph, located in the basement of an apartment building<br>it was estimated that the PGA at Niigata was of the order of 0.16 g. The soil below<br>seismograph liquefied during the earthquake. It is interesting to note the marked<br>change in form of record from a predominantly short period motion to a long<br>period motion which occurred after about 8 seconds.<br>SPT values were taken after the earthquake.<br>SPT energy was estimated as 73 % by Seed et al. (84)

Summary of Data

	Cetin et al. (2016)	Idriss&Boulanger (2010)	Seed et.al. (1984)		Cetin et al. (2016)	Idriss&Boulanger (2010)	Seed et.al. (1984)
Liquefied?	No	No	No	D_{50}	0.300 ± 0.050		0.3
Data Class	B			% Fines	0.0 ± 2.0	0.0	0
Critical Depth Range	16.4 - 23.0	20.0	20.0	% PI			
Depth to GWT (ft)	4.0 ± 0.3	3.9	4.0				
σ_v (psf)	2400.6 ± 145.1	2401.8	2400.0	N	30.0 ± 2.4	27.0	27
σ_v' (psf)	1421.9 ± 85.1	1399.3	1400.0	C_R	0.95	0.95	
a_{max} (g)	0.180 ± 0.027	0.18	0.180	C_S	1.00	1.00	
r_d	0.99 ± 0.091	0.95	0.960	C_B	1.00	1.00	
CSR	0.197 ± 0.035	0.191	0.195	C_E	1.21	1.21	1.21
Equivalent Magnitude	7.6	7.6		C_N	1.22	1.14	1.18
MSF		0.97	1.00	$(N_1)_{60}$	42.0 ± 3.4	35.5	38.5
CSRN		0.178	0.20				

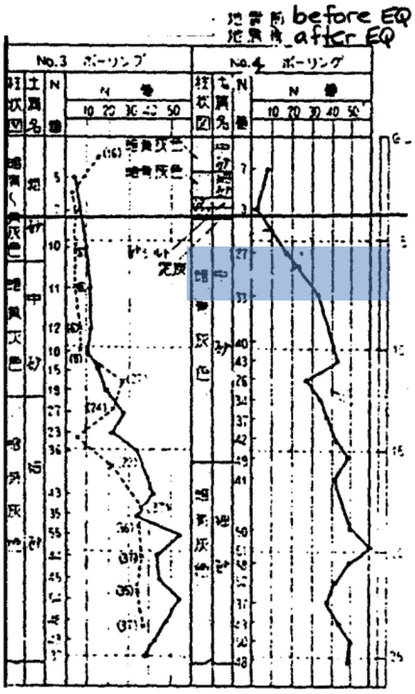

γ_m=110±3 pcf

γ_s=125±3 pcf

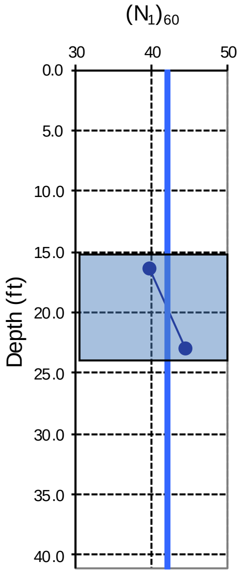

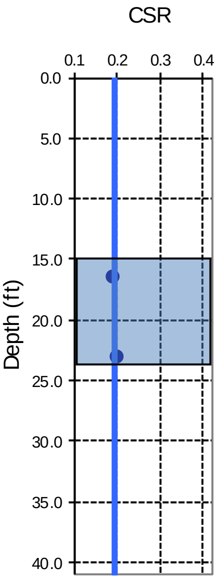

Depth (m)	Depth (ft)	CSR	N	C_N	C_R	$(N1)_{60}$
5	16.4	0.191	27	1.32	0.92	***39.6***
7.0	23.0	0.201	33	1.14	0.98	***44.4***
					**Mean:**	42.0
					**St. Dev.**	3.4

**Case number:**	17
**Earthquake:**	1968 Tokachi-Oki
**Magnitude:**	7.8 (MR)
**Location:**	Hachinohe-2
**References:**	Ohsaki (1970)
**Nature of Failure:**	No surface evidence of liquefaction. Moderate densification after earthquake.
**Comments:**	The sites are located in the city of Hachinohe ~560 kms north of Tokyo.<br>The site is a flat, sandy beach fronting on the Pacific Ocean.<br>The sites consist of sands almost entirely down to a depth of more than 20 m<br>from the ground surface containing large quantity of iron sand. However, the<br>iron sand had been excavated and backfilled with waste sand except at P2 &5<br>The ground water table is not deeper than 1.5 m everywhere in the site.<br>Site has been improved by vibrofloatation.<br>It seems like average Japanese estimated rod energy 72.5 % has been used<br>by Seed et al (1984). Same values will be adopted<br>The epicenter was located ~180 kms form the city of Hachinohe. The PGA was<br>recorded as 0.225 g at the harbor of Hachinohe.

**Summary of Data**	Cetin et al. (2016)	Idriss&Boulanger (2010)	Seed et.al. (1984)		Cetin et al. (2016)	Idriss&Boulanger (2010)	Seed et.al. (1984)
Liquefied?	No	No	No	D_{50}	0.250 ± 0.050		0.25
Data Class	B			% Fines	5.0 ± 2.0	5.0	5
Critical Depth Range	10.0 - 26.0	20.0	20.0	% PI			
Depth to GWT (ft)	7.0 ± 0.3	6.9	7.0				
σ_v (psf)	2180.0 ± 335.6	2401.8	2400.0	N	28.5 ± 2.2	28.0	28
σ_v' (psf)	1493.6 ± 172.2	1587.3	1590.0	C_R	0.94	0.95	
a_{max} (g)	0.225 ± 0.068	0.23	0.230	C_S	1.00	1.00	
r_d	0.99 ± 0.084	0.98	0.960	C_B	1.00	1.00	
CSR	0.211 ± 0.066	0.221	0.215	C_E	1.21	1.21	1.21
Equivalent Magnitude	8.3	8.3		C_N	1.19	1.10	1.10
MSF		0.81	0.93	$(N_1)_{60}$	38.4 ± 2.9	35.3	37.5
CSRN		0.254	0.23				

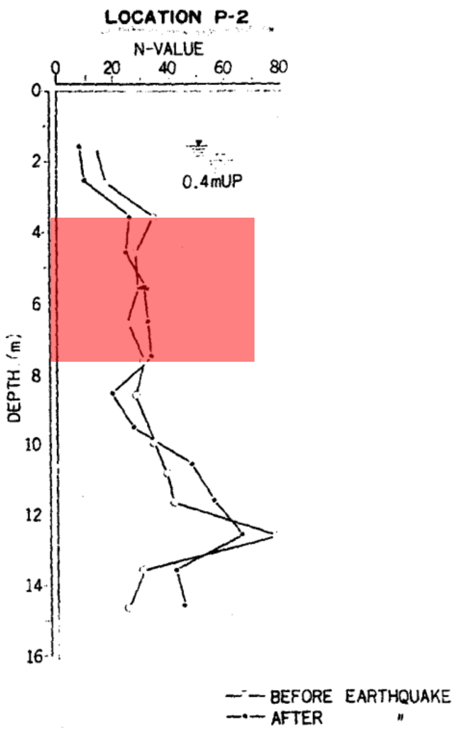

γ_m=115±3 pcf

γ_s=125±3 pcf

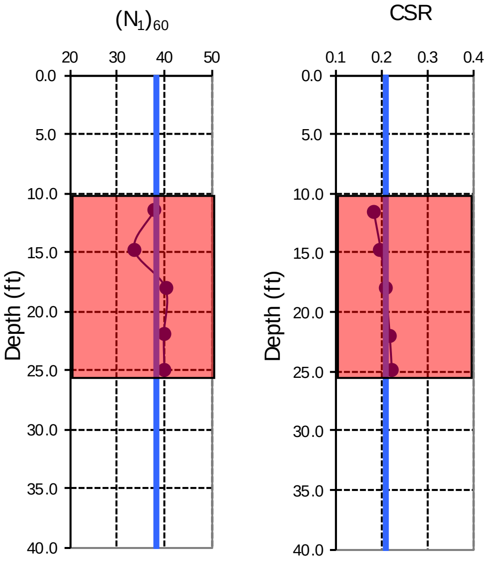

Depth (m)	Depth (ft)	CSR	N	C_N	C_R	$(N1)_{60}$
3.5	11.5	0.183	26	1.40	0.87	***38.1***
4.5	14.8	0.200	24	1.28	0.90	***33.6***
5.5	18.0	0.211	30	1.19	0.94	***40.4***
6.7	22.0	0.221	31	1.10	0.97	***40.0***
7.6	24.9	0.225	32	1.05	0.99	***40.2***
					**Mean:**	38.4
					**St. Dev.**	2.9

**Case number:**	18
**Earthquake:**	1968 Tokachi-Oki
**Magnitude:**	7.8 (MR)
**Location:**	Hachinohe-4
**References:**	Ohsaki (1970)
**Nature of Failure:**	No surface manifestation of soil liquefaction.
**Comments:**	The sites are located in the city of Hachinohe ~560 kms north of Tokyo.<br>The site is a flat, sandy beach fronting on the Pacific Ocean.<br>The sites consist of sands almost entirely down to a depth of more than 20 m from the ground surface containing large quantity of iron sand. However, the iron sand had been excavated and backfilled with waste sand except at P2 &5<br>The ground water table is not deeper than 1.5 m everywhere in the site.<br>Site has been improved by vibrofloatation.<br>It seems like average Japanese estimated rod energy 72.5 % has been used by Seed et al (1984). Same values will be adopted.<br>The epicenter was located ~180 kms form the city of Hachinohe. The PGA was recorded as 0.225 g at the harbor of Hachinohe.

**Summary of Data**	Cetin et al. (2016)	Idriss&Boulanger (2010)	Seed et.al. (1984)		Cetin et al. (2016)	Idriss&Boulanger (2010)	Seed et.al. (1984)
Liquefied?	No	No	No	D_{50}	0.250 ± 0.050		0.25
Data Class	B			% Fines	5.0 ± 2.0	5.0	5
Critical Depth Range	3.0 - 13.0	13.1	13.0	% PI			
Depth to GWT (ft)	3.0 ± 0.3	3.0	3.0				
σ_v (psf)	955.0 ± 209.1	1566.4	1560.0	N	14.0 ± 1.2	16.0	16
σ_v' (psf)	643.0 ± 106.7	939.8	940.0	C_R	0.82	0.85	
a_{max} (g)	0.225 ± 0.068	0.23	0.230	C_S	1.00	1.00	
r_d	1.00 ± 0.042	0.99	0.970	C_B	1.00	1.00	
CSR	0.217 ± 0.067	0.246	0.240	C_E	1.21	1.21	1.21
Equivalent Magnitude	8.3	8.3		C_N	1.81	1.40	1.41
MSF		0.81	0.94	$(N_1)_{60}$	25.1 ± 2.2	23.0	27.5
CSRN		0.276	0.26				

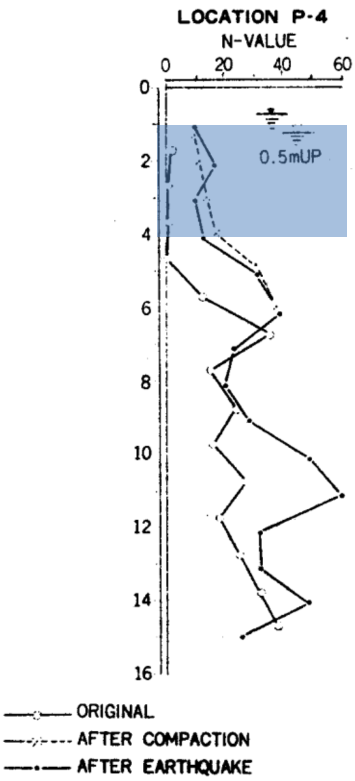

γ_m=110±3 pcf

γ_s=125±3 pcf

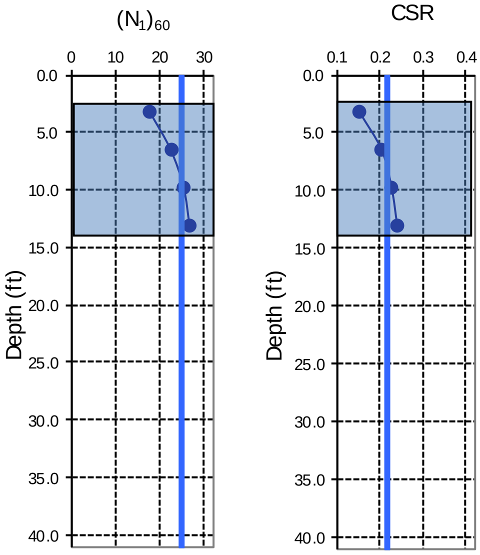

Depth (m)	Depth (ft)	CSR	N	C_N	C_R	$(N1)_{60}$
1.0	3.3	0.153	10	2.00	0.73	17.8
2.0	6.6	0.205	12	1.96	0.80	***22.6***
3.0	9.8	0.228	15	1.67	0.85	***25.6***
4.0	13.1	0.241	17	1.48	0.89	***27.0***
					**Mean:**	25.1
					**St. Dev.**	2.2

**Case number:**	19
**Earthquake:**	1968 Tokachi-Oki
**Magnitude:**	7.8 (MR)
**Location:**	Hachinohe-6
**References:**	Ohsaki (1970)
**Nature of Failure:**	Eruption of sand and water. Significant densification after earthquake
**Comments:**	The sites are located in the city of Hachinohe ~560 kms north of Tokyo.<br>The site is a flat, sandy beach fronting on the Pacific Ocean.<br>The sites consist of almost entirely sandy deposits down to a depth of 20 m from the ground surface containing large quantity of iron sand. However, the iron sand had been excavated and backfilled with waste sand except at P2 &5<br>The ground water table is not deeper than 1.5 m everywhere in the site.<br>Site has been improved by vibrofloatation.<br>Estimated rod energy 65 % has been used by Seed et al. Same value will be adopted for this study.<br>The epicenter was located ~180 kms form the city of Hachinohe. The PGA was recorded as 0.225 g at the harbor of Hachinohe.

**Summary of Data**	Cetin et al. (2016)	Idriss&Boulanger (2010)	Seed et.al. (1984)		Cetin et al. (2016)	Idriss&Boulanger (2010)	Seed et.al. (1984)
Liquefied?	Yes	Yes	Yes	D_{50}	0.250 ± 0.050		0.25
Data Class	B			% Fines	5.0 ± 2.0	5.0	5
Critical Depth Range	6.6 - 20.0	13.1	13.0	% PI			
Depth to GWT (ft)	2.0	2.0	2.0				
σ_v (psf)	1632.5 ± 281.3	1566.4	1560.0	N	4.7 ± 0.5	6.0	6
σ_v' (psf)	927.4 ± 144.7	877.2	870.0	C_R	0.89	0.85	
a_{max} (g)	0.225 ± 0.068	0.23	0.230	C_S	1.00	1.00	
r_d	0.99 ± 0.065	0.99	0.970	C_B	1.00	1.00	
CSR	0.254 ± 0.079	0.265	0.260	C_E	1.09	1.09	1.09
Equivalent Magnitude	8.3	8.3		C_N	1.51	1.63	1.47
MSF		0.81	0.95	$(N_1)_{60}$	6.8 ± 0.7	9.1	9.5
CSRN		0.304	0.28				

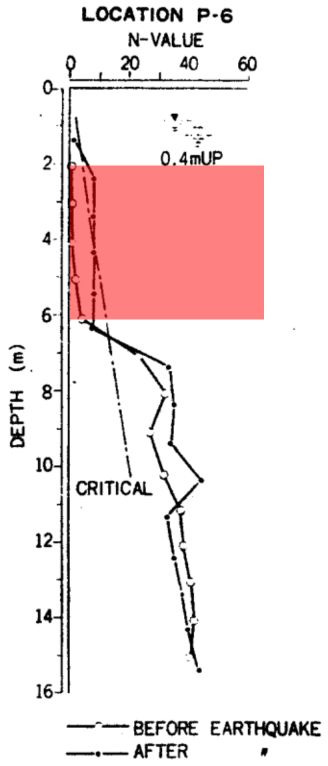

γ_m=110±3 pcf

γ_s=125±3 pcf

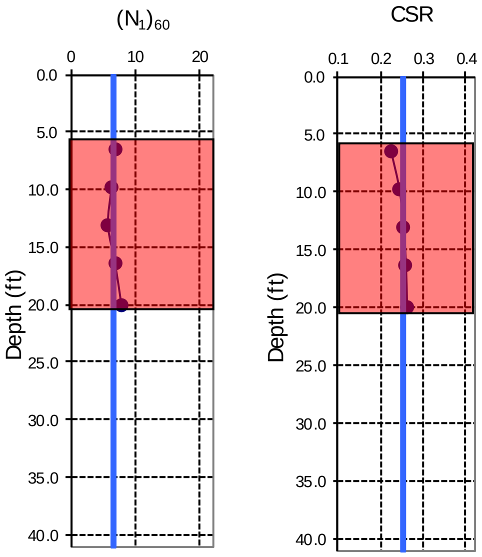

Depth (m)	Depth (ft)	CSR	N	C_N	C_R	$(N1)_{60}$
2.0	6.6	0.228	4	2.00	0.80	***7.0***
3.0	9.8	0.245	4	1.73	0.85	***6.4***
4.0	13.1	0.254	4	1.52	0.89	***5.9***
5.0	16.4	0.259	5	1.37	0.92	***6.9***
6.1	20.0	0.261	6	1.25	0.95	***7.8***
					**Mean:**	6.8
					**St. Dev.**	0.7

**Case number:**	20
**Earthquake:**	1968 Tokachi-Oki
**Magnitude:**	7.8 (MR)
**Location:**	Nanaehama
**References:**	Kishida (1970)
**Nature of Failure:**	"complete liquefaction of level ground from the ground surface to some depth was observed"<br>"water and soil began to spout out from the ground surface soon after the beginning of the earthquake and continued for about an hour"<br>So soft, hard to walk on even a week after the earthquake
**Comments:**	The site is located at Nanaehama beach in Kamiiso Town in Hokkaido Island<br>Reclamation was made at the site by hydarulic fill at about three years before the earthquake. The soil was coarse grained sand in accordance with the unified classification system, having the 50% size of less than 2.0 mm and the uniformity coefficient of less than 10. The relative density was less than 75%<br>No fine grained strata above the saturated sand.<br>3 borings before the earthquake and 3 additional borings after the earthquake<br>SPT energy was estimated as 70 % by Seed et al (1984)

Summary of Data

	Cetin et al. (2016)	Idriss&Boulanger (2010)	Seed et.al. (1984)		Cetin et al. (2016)	Idriss&Boulanger (2010)	Seed et.al. (1984)
Liquefied?	Yes	Yes	Yes	D_{50}	0.121 ± 0.034		0.12
Data Class	B			% Fines	21.7 ± 4.8	20.0	20
Critical Depth Range	3.0 - 16.4	13.1	13.0	% PI			
Depth to GWT (ft)	2.5 ± 0.6	3.0	3.0				
σ_v (psf)	1139.2 ± 269.1	1566.4	1560.0	N	5.6 ± 0.9	5.0	5
σ_v' (psf)	690.1 ± 134.0	939.8	940.0	C_R	0.84	0.85	
a_{max} (g)	0.200 ± 0.060	0.20	0.200	C_S	1.00	1.00	
r_d	0.97 ± 0.050	0.99	0.970	C_B	1.00	1.00	
CSR	0.207 ± 0.065	0.213	0.210	C_E	1.17	1.17	1.17
Equivalent Magnitude	8.3	8.3		C_N	1.75	1.52	1.41
MSF		0.81	0.95	$(N_1)_{60}$	9.6 ± 1.5	7.6	8
CSRN		0.244	0.22				

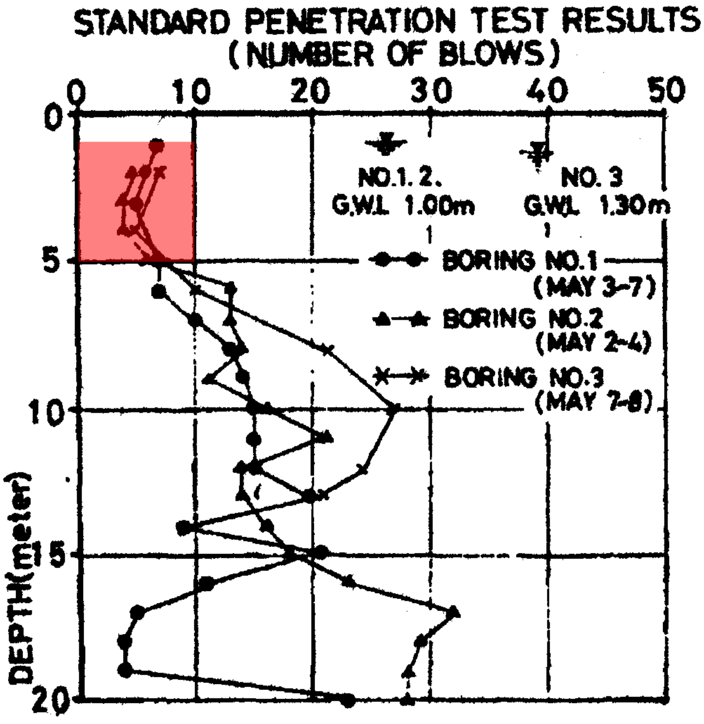

(A) Before the earthquake

γ_m=110±3 pcf

γ_s=120±3 pcf

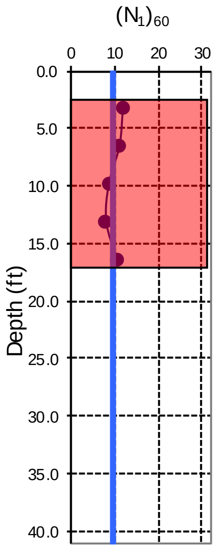

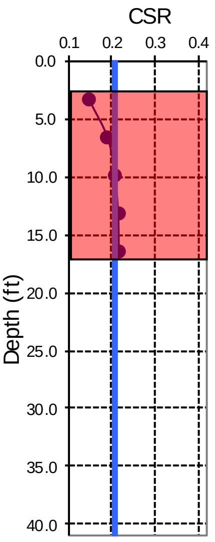

Depth (m)	Depth (ft)	CSR	N	C_N	C_R	$(N1)_{60}$
1.0	3.3	0.148	7	2.00	0.73	12.0
2.0	6.6	0.191	6	2.00	0.80	***11.2***
3.0	9.8	0.208	5	1.74	0.85	***8.6***
4.0	13.1	0.215	5	1.54	0.89	***8.0***
5.0	16.4	0.217	7	1.40	0.92	***10.5***
					**Mean:**	9.6
					**St. Dev.**	1.5

**Case number:**	21
**Earthquake:**	1968 Tokachi-Oki
**Magnitude:**	7.8 (MR)
**Location:**	Aomori City (Aomori Station)
**References:**	Yasuda and Tohno (1988)
**Nature of Failure:**	Sand boils, settled structures.
**Comments:**	The station faces the sea and the north part of the station is used as a landing place for ferryboats. The north part was built on reclaimed land constructed around 1945, but the sout part was built on natural land. Liquefaction was induced at the north part of the station during the Tokachi-Oki and the main shock of Nihonakai-chubu EQ's. Many sand volcanoes, settlement of rails and buildings and uplift of buried tanks were observed. The maximum surface acceleration at Aomari Port, about 1 km north of Aomori Station was recorded as 0.213 g.

Summary of Data

	Cetin et al. (2016)	Idriss&Boulanger (2010)	Seed et.al. (1984)		Cetin et al. (2016)	Idriss&Boulanger (2010)	Seed et.al. (1984)
Liquefied?	Yes	No		D_{50}	0.250 ± 0.050		
Data Class	A			% Fines	3.0 ± 2.0	3.0	
Critical Depth Range	13.1 - 24.6	18.7		% PI			
Depth to GWT (ft)	0.0 ± 0.4	0.0					
σ_v (psf)	2263.8 ± 236.6	1984.1		N	9.0 ± 0.9	9.0	
σ_v' (psf)	1086.6 ± 124.8	793.6		C_R	0.94	0.95	
a_{max} (g)	0.213 ± 0.011	0.21		C_S	1.00	1.00	
r_d	0.87 ± 0.087	0.98		C_B	1.00	1.00	
CSR	0.250 ± 0.029	0.335		C_E	1.22	1.22	
Equivalent Magnitude	8.3	8.3		C_N	1.40	1.58	
MSF		0.81		$(N_1)_{60}$	14.4 ± 1.4	16.5	
CSRN		0.376					

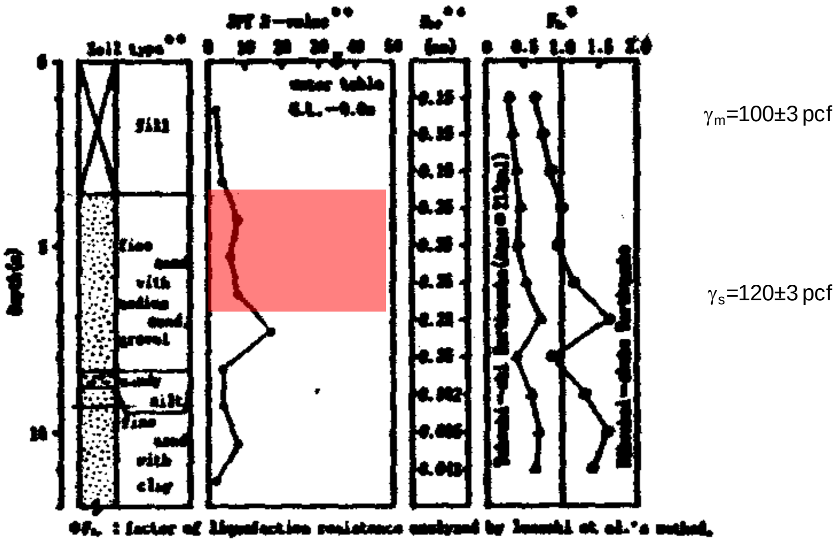

*FL : factor of liquefaction resistance analyzed by Iwasaki et al.'s method.
** : data of soil condition and FL —values during the Nihonkai—chubu Earthquake are quoted from the Railway Technical Research Institute, JNR (1984)

γ_m=100±3 pcf

γ_s=120±3 pcf

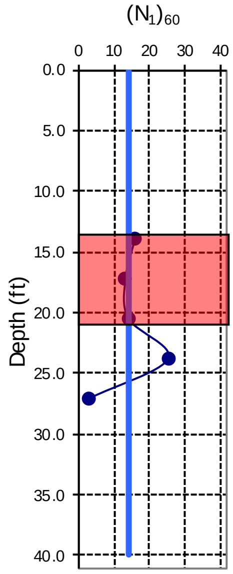

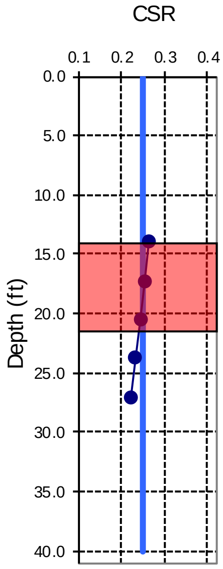

Depth (m)	Depth (ft)	CSR	N	C_N	C_R	$(N1)_{60}$	
4.3	13.9	0.264	9.0	1.62	0.90	***15.9***	
5.3	17.2	0.255	8.0	1.46	0.93	***13.2***	
6.3	20.5	0.245	9.0	1.34	0.96	***14.0***	
7.3	23.8	0.234	17.0	1.24	0.98	25.3	Gravel?
8.3	27.1	0.223	2.0	1.17	1.00	2.8	Clay?
					**Mean:**	14.4	
					**St. Dev.**	1.4	

**Case number:**	22
**Earthquake:**	1971 San Fernando
**Magnitude:**	6.4 (ML) (Mw=6.6)
**Location:**	Juvenile Hall
**References:**	Bennett (1989)
**Nature of Failure:**	Lateral Spreading, sand boils
**Comments:**	On February 9, 1971, a magnitude ML 6.4 earthquake caused major damage in the San Fernando Valley, CA. One facility that received heavy damage was San Fernando Valley Juvenile Hall. The site is located north of the main trace of surface faulting on the upthrust block. About 50 sand boils occurred in the field south of the Juvenile Hall near boring 10. Sand boils also occurred in the northwestern and northeastern parts of Upper Van Norman Lake. The lateral spread lies in the north-central part of San Fernando Valley within a small synclinal basin bounded on the north and south by hills composed of clastic sedimentary rocks. The basin contains several alluvial fans that originate from Welden, Grapevine, and Sombrero canyons.<br>On the basis of sediment properties and penetration data, four units have been defined; From top to bottom the units are A, poorly sorted, loose to medium-dense silty sand; B, very loose to loose poorly sorted silty sand to silt; C, medium-dense to dense, poorly sorted silty sand; and D, stiff clayey silt. Unit B is divided into two subunits, B1 and B2 on the basis of grain size and plasticity. Subunit B1 consists of poorly sorted, loose sandy silt and silty sand, it has an average sand and gravel content of 38 %, an average clay content of 10 %. Subunit B2 consists of very loose, poorly sorted sandy silt and silt. It has an average clay content of 19 %. Unit B2 is probably not susceptible to liquefaction due to large clay fraction.Unit B has a PI of 3-10 %.<br>PGA was estimated at the crest and the abutments of Lower San Fernando Dam as 0.55 and 0.48 g. A value of 0.45 is adopted as used by Seed et al (84)<br>SPT energy was given as 68 %<br>THE BORINGS MAY NOT BE IDENTICAL WITH THE ONE USED BY SEED ET AL. (84).

Summary of Data

	Cetin et al. (2016)	Idriss&Boulanger (2010)	Seed et.al. (1984)		Cetin et al. (2016)	Idriss&Boulanger (2010)	Seed et.al. (1984)
Liquefied?	Yes	Yes	Yes	D_{50}	0.047 ± 0.011		Silt
Data Class	A			% Fines	65.3 ± 8.0	55.0	>50
Critical Depth Range	14.4 - 20.7	20.0	20.0	% PI	3-10		
Depth to GWT (ft)	14.0 ± 0.2	15.1	15.0				
σ_v (psf)	1966.3 ± 132.0	2339.2	2330.0	N	3.3 ± 0.9	3.5	2
σ_v' (psf)	1744.6 ± 74.3	2005.0	2000.0	C_R	0.90	0.95	0.75
a_{max} (g)	0.450 ± 0.068	0.45	0.450	C_S	1.00	1.00	
r_d	0.86 ± 0.082	0.92	0.950	C_B	1.00	1.00	
CSR	0.283 ± 0.051	0.312	0.325	C_E	1.13	1.13	1.00
Equivalent Magnitude	6.6	6.6		C_N	1.10	1.03	1.00
MSF		1.26	1.16	$(N_1)_{60}$	3.7 ± 1.0	3.9	1.5
CSRN		0.246	0.28				

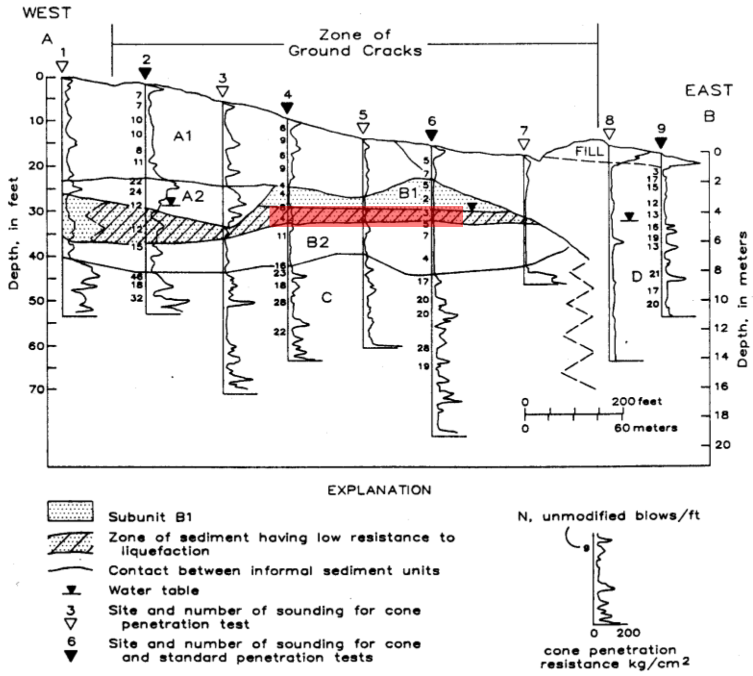

γ_m=110±3 pcf

γ_s=120±3 pcf

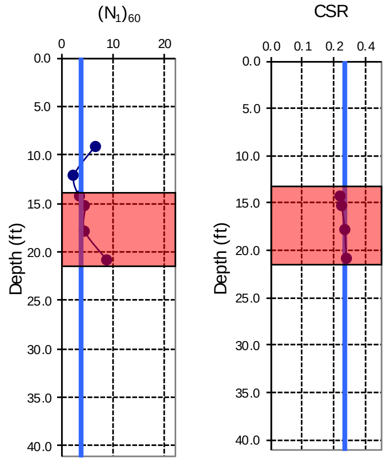

Depth (m)	Depth (ft)	CSR	N	C_N	C_R	$(N1)_{60}$	FC (%)	Silt	Clay	D_{50}	
2.8	9.1	NA	5.0	1.45	0.79	6.5	83	72	11	0.042	BH6
3.7	12.1	NA	2.0	1.26	0.84	*2.4*	64	48	16	0.037	BH6
4.4	14.3	0.266	3.0	1.17	0.87	*3.4*	74	62	12	0.042	BH6
4.6	15.3	0.272	4.0	1.15	0.88	*4.6*	68	56	12	0.045	BH4
5.4	17.8	0.284	4.0	1.10	0.91	*4.5*	55	46	9	0.062	BH4
6.3	20.8	0.291	8.0	1.05	0.93	8.9	66	58	8	0.052	BH4
					**Mean:**	3.7	65.3			0.047	
					**St. Dev.**	1.0	8.0			0.011	

**Case number:**	23
**Earthquake:**	1971 San Fernando
**Magnitude:**	6.4 (M_L) (Mw=6.6)
**Location:**	Van Norman
**References:**	Bennett (1989)
**Nature of Failure:**	Lateral Spreading, sand boils
**Comments:**	On February 9, 1971, a magnitude M_L 6.4 earthquake caused major damage in the San Fernando Valley, CA. One facility that received heavy damage was the San Fernando Valley Juvenile Hall. The site is located north of the main trace of surface faulting on the upthrust block. About 50 sand boils occurred in the field south of the Juvenile Hall near boring 10. Sand boils also occurred in the northwestern and northeastern parts of Upper Van Norman Lake. The lateral spread lies in the north-central part of San Fernando Valley within a small synclinal basin bounded on the north and south by hills composed of clastic sedimentary rocks. The basin contains several alluvial fans that originate from Welden, Grapevine, and Sombrero canyons.<br>On the basis of sediment properties and penetration data, four informal units have been defined; From top to bottom the units are A, poorly sorted, loose to medium-dense silty sand; B, very loose to loose poorly sorted silty sand to silt; C, medium-dense to dense, poorly sorted silty sand; and D, stiff clayey silt. Unit B is divided into two subunits, B1 and B2 on the basis of grain size and plasticity. Subunit B1 consists of poorly sorted, loose sandy silt and silty sand, it has an average sand and gravel content of 38 %, an average clay content of 10 %. Subunit B2 consists of very loose, poorly sorted sandy silt and silt. It has an average clay content of 19 %. Unit B2 is probably not susceptible to liquefaction due to large clay fraction.Unit B has a PI of 3-10 %.<br>PGA was estimated at the crest and the abutments of Lower San Fernando Dam as 0.55 and 0.48 g. A value of 0.45 is adopted as used by Seed et al (84)<br>SPT energy was given as 68 %<br>THE BORINGS MAY NOT BE IDENTICAL WITH THE ONE USED BY SEED ET AL. (84).

Summary of Data

	Cetin et al. (2016)	Idriss&Boulanger (2010)	Seed et.al. (1984)		Cetin et al. (2016)	Idriss&Boulanger (2010)	Seed et.al. (1984)
Liquefied?	Yes	Yes	Yes	D_{50}	0.067 ± 0.034		0.1
Data Class	A			% Fines	59.3 ± 14.2	50.0	20
Critical Depth Range	17.0 - 24.0	20.0	20.0	% PI	3-10		
Depth to GWT (ft)	16.3 ± 0.9	15.1	10.0				
σ_v (psf)	2296.9 ± 149.1	2339.2	2250.0	N	7.4 ± 2.5	7.3	9
σ_v' (psf)	2035.2 ± 96.2	2005.0	1800.0	C_R	0.93	0.95	0.75
a_{max} (g)	0.450 ± 0.068	0.45	0.450	C_S	1.00	1.00	
r_d	0.90 ± 0.094	0.92	0.950	C_B	1.00	1.00	
CSR	0.297 ± 0.055	0.312	0.345	C_E	1.13	1.13	1.00
Equivalent Magnitude	6.6	6.6		C_N	1.02	1.03	1.05
MSF		1.26	1.15	$(N_1)_{60}$	7.9 ± 2.7	8.1	7
CSRN		0.245	0.30				

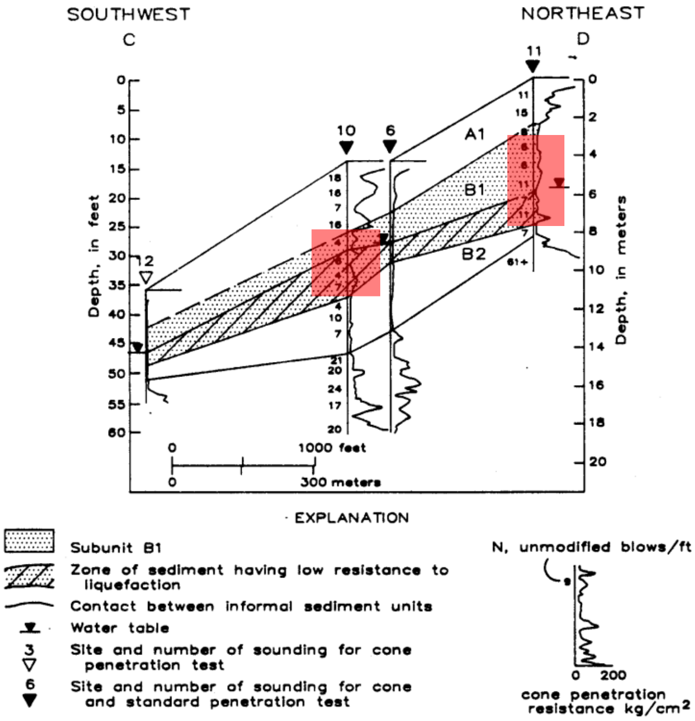

γ_m=110±3 pcf

γ_s=120±3 pcf

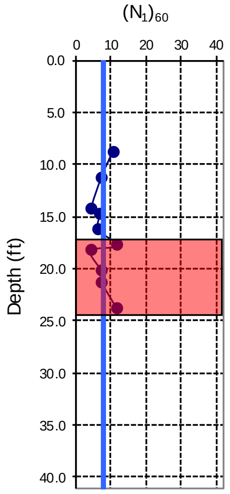

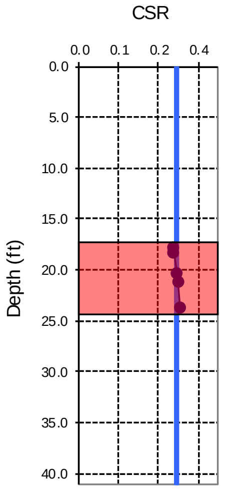

Depth (m)	Depth (ft)	CSR	N	C_N	C_R	$(N1)_{60}$	FC (%)	Silt	Clay Content	D50	
2.7	8.8	NA	8.0	1.48	0.78	***10.5***	***56.0***	42	14	0.059	BH11
3.4	11.3	NA	6.0	1.31	0.82	***7.3***	***65.0***	46	19	0.043	BH11
4.3	14.3	NA	4.0	1.16	0.87	***4.6***	***78.0***	58	20	0.031	BH10
4.5	14.8	NA	6.0	1.14	0.87	***6.8***	***52.0***	40	12	0.067	BH11
5.0	16.3	NA	6.0	1.09	0.89	***6.6***	***52.0***	45	7	0.072	BH10
5.4	17.8	0.283	11.0	1.06	0.90	***12.0***	***38.0***	30	8	0.14	BH11
5.6	18.3	0.286	4.0	1.05	0.91	***4.3***	***65.0***	58	7	0.055	BH10
6.2	20.3	0.296	7.0	1.02	0.93	***7.5***	***61.0***	47	14	0.051	BH11
6.5	21.3	0.300	7.0	1.01	0.94	***7.5***	***83.0***	70	13	0.038	BH10
7.2	23.8	0.307	11.0	0.98	0.96	***11.7***	***43.0***	33	10	0.11	BH11
					**Mean:**	7.9	59.3			0.1	
					**St. Dev.**	2.7	14.2			0.0	

**Case number:**	24
**Earthquake:**	1975 Haicheng
**Magnitude:**	7.0 (Mw) USGS Centennial Earthquake Catalog
**Location:**	Panjin Chemical Fertilizer Plant
**References:**	Shengcong et al (1983)<br>Fear et al. (1995)
**Nature of Failure:**	Liquefied site<br>Extensive sand boils and sand volcanoes were seen in the NW part of the region of 7th grade seismic intensity.
**Comments:**	This site was located in the NW part of the region and shaken by 7th grade on Chinese intensity scale. (corresponds to JMA scale of 4-5). It is located mainly in flood plain; top clayey layer ~2.6 m thick and underlain by liquefaction susceptible silty sand or fine sand > 2m thick.<br>At the moment of Haicheng EQ, the surface clayey soil layers were frozen at many sites.<br>A 65 m high reinforced concrete tower on 17 m reinforced concrete piles was not damaged- its walls cracked due to differential settlement of the ground.<br>There was almost no liquefaction near the epicenter of he EQ.<br>PGA was estimated as >0.1 g<br>SPT energy was estimated as 50 % by Seed et al. (84)

**Summary of Data**	Cetin et al. (2016)	Idriss&Boulanger (2010)	Seed et.al. (1984)		Cetin et al. (2016)	Idriss&Boulanger (2010)	Seed et.al. (1984)
Liquefied?	Yes	Yes	Yes	D_{50}	0.064 ± 0.050		0.064
Data Class	B			% Fines	67.0 ± 2.0	67.0	67
Critical Depth Range	11.5 - 41.0	26.9	30.0	% PI			
Depth to GWT (ft)	5.0 ± 0.3	4.9	5.0				
σ_v (psf)	3099.7 ± 594.2	3237.2	3675.0	N	8.1 ± 1.2	9.1	8
σ_v' (psf)	1773.1 ± 291.2	1858.8	2115.0	C_R	1.00	0.95	
a_{max} (g)	0.130 ± 0.039	0.20	0.130	C_S	1.00	1.00	
r_d	0.86 ± 0.116	0.89	0.920	C_B	1.00	1.00	
CSR	0.127 ± 0.042	0.203	0.135	C_E	0.83	0.83	0.83
Equivalent Magnitude	7.0	7.0		C_N	1.09	1.07	0.98
MSF		1.14	1.04	$(N_1)_{60}$	7.4 ± 1.1	7.6	6.5
CSRN		0.175	0.13				

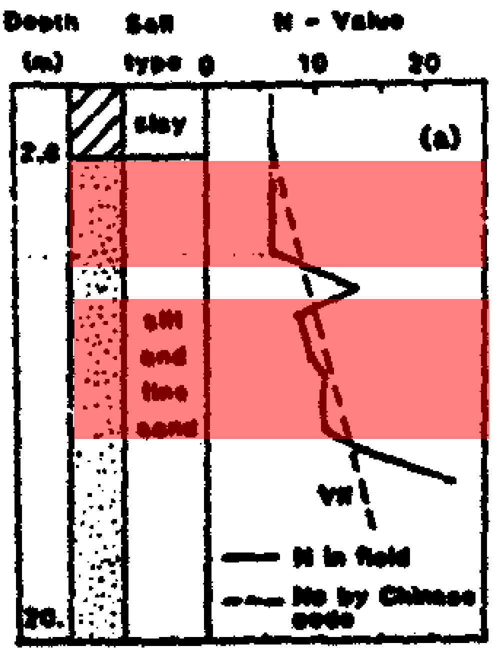

γ_m=110±3 pcf

γ_s=120±3 pcf

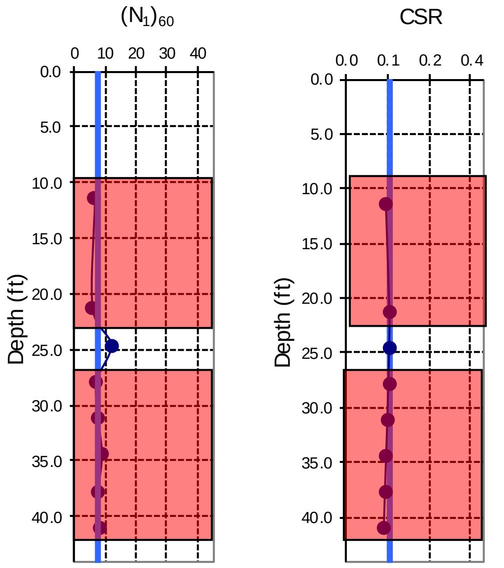

Depth (m)	Depth (ft)	CSR	N	C_N	C_R	$(N1)_{60}$	
3.5	11.5	0.118	6.0	1.51	0.87	***6.6***	
6.5	21.3	0.129	6.0	1.19	0.96	***5.7***	
7.5	24.6	0.128	13.0	1.12	0.99	12.0	gravel?
8.5	27.9	0.125	8.0	1.06	1.00	***7.1***	
9.5	31.2	0.122	9.0	1.01	1.00	***7.6***	
10.5	34.4	0.118	11.0	0.97	1.00	***8.9***	
11.5	37.7	0.115	10.0	0.93	1.00	***7.8***	
12.5	41.0	0.111	11.0	0.90	1.00	***8.2***	
					**Mean:**	7.4	
					**St. Dev.**	1.1	

**Case number:**	25
**Earthquake:**	1975 Haicheng
**Magnitude:**	7.0 (Mw) USGS Centennial Earthquake Catalog
**Location:**	Yingkou Glass Fibre Plant
**References:**	Shengcong et al (1983)<br>Fear et al. (1995)
**Nature of Failure:**	Liquefied site
**Comments:**	Yingkou Glass Fibre Plant site is located in the west of epicenter, in the 9th grade on the Chinese intensity scale, in the zone of slight liquefaction.<br>Thin top clayey layers were underlain by greater than 2 m thick potentially liquefiable silty fine sand layers.<br>At the moment of the EQ, the surface clayey soil layers were frozen at many sites.<br>PGA was estimated as >0.1 g<br>SPT energy was estimated as 60 % by Seed et al. (84)

Summary of Data

	Cetin et al. (2016)	Idriss&Boulanger (2010)	Seed et.al. (1984)		Cetin et al. (2016)	Idriss&Boulanger (2010)	Seed et.al. (1984)
Liquefied?	Yes	Yes	Yes	D_{50}	0.078 ± 0.050		0.078
Data Class	B			% Fines	48.0 ± 2.0	48.0	48
Critical Depth Range	16.4 - 29.5	25.6	27.0	% PI			
Depth to GWT (ft)	5.0 ± 0.2	4.9	5.0				
σ_v (psf)	2706.0 ± 268.4	3070.2	3300.0	N	12.4 ± 1.0	13.0	13
σ_v' (psf)	1584.1 ± 138.3	1775.3	1930.0	C_R	0.98	0.95	
a_{max} (g)	0.200 ± 0.060	0.30	0.200	C_S	1.00	1.00	
r_d	0.89 ± 0.103	0.90	0.930	C_B	1.00	1.00	
CSR	0.197 ± 0.063	0.304	0.210	C_E	1.00	1.00	1.00
Equivalent Magnitude	7.0	7.0		C_N	1.16	1.08	1.02
MSF		1.14	1.05	$(N_1)_{60}$	14.0 ± 1.1	13.3	13.5
CSRN		0.260	0.20				

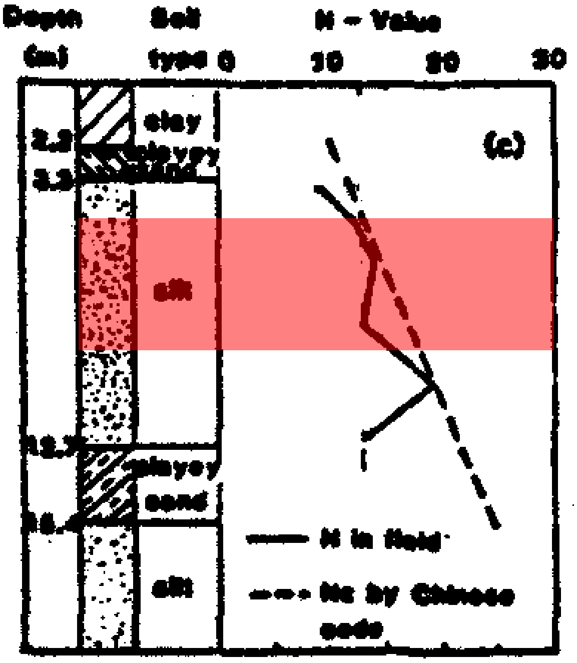

γ_m=110±3 pcf

γ_s=120±3 pcf

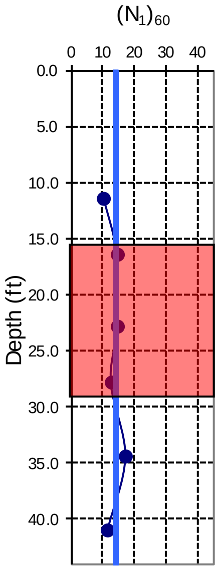

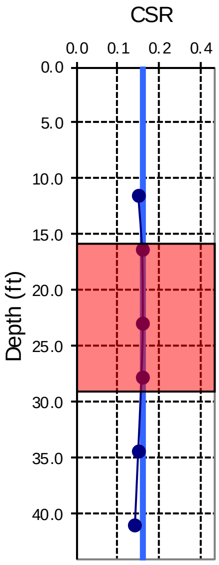

Depth (m)	Depth (ft)	CSR	N	C_N	C_R	$N_{1,60}$	
3.5	11.5	0.181	8.0	1.51	0.87	10.5	clayey sand?
5.0	16.4	0.194	12.0	1.32	0.92	***14.6***	
7.0	23.0	0.197	13.0	1.16	0.98	***14.7***	
8.5	27.9	0.192	12.0	1.06	1.00	***12.8***	
10.5	34.4	0.180	18.0	0.97	1.00	17.5	
12.5	41.0	0.169	13.0	0.90	1.00	11.7	
					**Mean:**	14.0	
					**St. Dev.**	1.1	

**Case number:**	26
**Earthquake:**	1975 Haicheng
**Magnitude:**	7.0 (Mw) USGS Centennial Earthquake Catalog
**Location:**	Yingkou Paper Plant
**References:**	Shengcong et al (1983)<br>Fear et al. (1995)
**Nature of Failure:**	Liquefied site
**Comments:**	Yingkou Paper Plant site is located in the west of epicenter, in the 9th grade on the Chinese intensity scale, in the zone of slight liquefaction.<br>Thin top clayey layers were underlain by greater than 2 m thick potentially liquefiable silty fine sand layers.<br>At the moment of the EQ, the surface clayey soil layers were frozen at many sites.<br>PGA was estimated as >0.1 g<br>SPT energy was estimated as 60 % by Seed et al. (84)

Summary of Data

	Cetin et al. (2016)	Idriss&Boulanger (2010)	Seed et.al. (1984)		Cetin et al. (2016)	Idriss&Boulanger (2010)	Seed et.al. (1984)
Liquefied?	Yes	Yes	Yes	D_{50}	0.100 ± 0.050		silt and sand
Data Class	B			% Fines	20.0 ± 2.0	5.0	silt and sand
Critical Depth Range	14.8 - 34.4	26.9	27.0	% PI			
Depth to GWT (ft)	5.0 ± 0.2	4.9	5.0				
σ_v (psf)	2902.9 ± 398.4	3299.9	3300.0	N	10.3 ± 3.2	11.0	11
σ_v' (psf)	1678.6 ± 198.8	1921.5	1930.0	C_R	0.99	0.95	
a_{max} (g)	0.200 ± 0.060	0.30	0.200	C_S	1.00	1.00	
r_d	0.81 ± 0.110	0.89	0.930	C_B	1.00	1.00	
CSR	0.183 ± 0.060	0.298	0.210	C_E	1.00	1.00	1.00
Equivalent Magnitude	7.0	7.0		C_N	1.12	1.05	1.02
MSF		1.14	1.05	$(N_1)_{60}$	11.4 ± 3.6	11.0	11
CSRN		0.259	0.20				

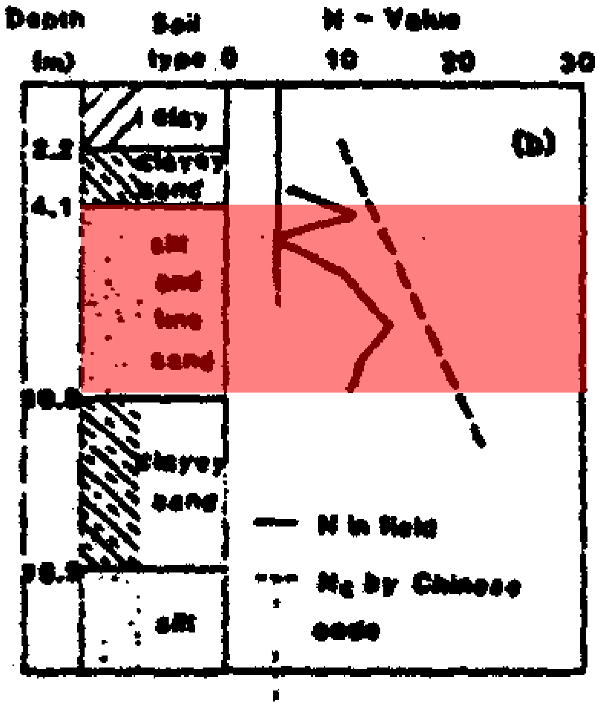

γ_m=110±3 pcf

γ_s=120±3 pcf

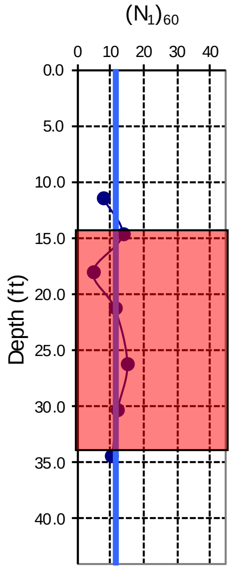

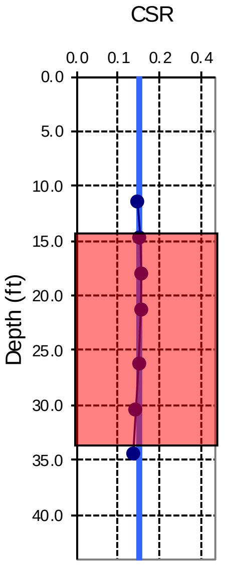

Depth (m)	Depth (ft)	CSR	N	C_N	C_R	$(N1)_{60}$	
3.5	11.5	0.177	6.0	1.51	0.87	7.9	clayey sand?
4.5	14.8	0.185	11.0	1.38	0.90	***13.7***	
5.5	18.0	0.188	4.0	1.28	0.94	***4.8***	
6.5	21.3	0.187	10.0	1.19	0.96	***11.5***	
8.0	26.2	0.180	14.0	1.09	1.00	***15.3***	
9.3	30.3	0.173	12.0	1.03	1.00	***12.3***	
10.5	34.4	0.164	11.0	0.97	1.00	***10.7***	
					**Mean:**	11.4	
					**St. Dev.**	3.6	

**Case number:**	27
**Earthquake:**	1976 Guatemala
**Magnitude:**	7.5 (M_L)
**Location:**	Amatitlan B-1
**References:**	Seed et al. (1979)
**Nature of Failure:**	Subsidence and flooding of beach areas, severe ground cracking sizes up to several meters. Severe damage to houses, numerous sand boils and lateral spreading-induced structural damages. Expulsion of occasional pieces of of pumice, ranging from small gravel to cobble sizes together with sand boil.
**Comments:**	The sites are located on deltaic deposit near the mouth of a small river, Rio Villalobos at the settlement of La Playa on the north-east shore of Lake Amatitlan.The general area behind the shoreline is quite flat with the ground sloping gently up about 2 degs. away from the lake.<br>Borehole is located in the midst of the highly liquefied zone.<br>Two test pits were dug to a depth of ~ 5' on the level ground 40' from the beach These showed the upper 4' of soil to be pumice sand underlain by a thin layer of dense fibrous peats and organic silt. Two field density test in the pumice sand revealed that it had wet and dry densities ranging from 55.5-61.5 and 35.5-39.4 pcf. respectively. Light-weight pumice sand at all boring locations was located above water table and was not itself the layer of liquefaction.<br>The epicenter was located ~170 kms north-east from Lake Amatitlan However, north terminus of the fault is only 40 kms away from the lake.<br>The Modified Mercalli Intensity of shaking at the site is high VI or low VII. Based on correlations, PGA was estimated as 0.12-0.15 g. This estimation was checked by the level of damage at nearby structures. Also attenuation relationships confirmed the range. Based on seismoscope records, USGS predicted the PGA at a site ~ 25 km from the causative fault as 0.25 g. Calibrating the attenuation relationships with this information, PGA at the site is predicted as 0.15 g.<br>Local Magnitude saturates at magnitude 7. Thus, it is hard to estimate the moment magnitude for the earthquake. However based on the recorded motion and attenuation relationships, moment magnitude is back calculated as 7-8.2.<br>Rope and pulley system (2 turns of the rope around the pulley) was used.<br>Estimated rod energy is 45%. SPT values were taken after the earthquake.

Summary of Data

	Cetin et al. (2016)	Idriss&Boulanger (2010)	Seed et.al. (1984)		Cetin et al. (2016)	Idriss&Boulanger (2010)	Seed et.al. (1984)
Liquefied?	Yes	Yes	Yes	D_{50}	0.800 ± 0.050		0.8
Data Class	C			% Fines	3.0 ± 2.0	3.0	3
Critical Depth Range	10.0 - 50.0	34.1	34.0	% PI			
Depth to GWT (ft)	5.0	4.9	5.0				
σ_v (psf)	2550.0 ± 600.6	2903.1	2910.0	N	4.3 ± 1.4	6.0	6
σ_v' (psf)	990.0 ± 186.0	1796.1	1800.0	C_R	1.00	1.00	
a_{max} (g)	0.135 ± 0.041	0.14	0.135	C_S	1.00	1.00	
r_d	0.57 ± 0.117	0.89	0.890	C_B	1.00	1.00	
CSR	0.129 ± 0.047	0.126	0.190	C_E	0.75	0.75	0.75
Equivalent Magnitude	7.5	7.5		C_N	1.46	1.10	1.05
MSF		1.00	1.00	$(N_1)_{60}$	4.7 ± 1.5	5.0	5
CSRN		0.125	0.19				

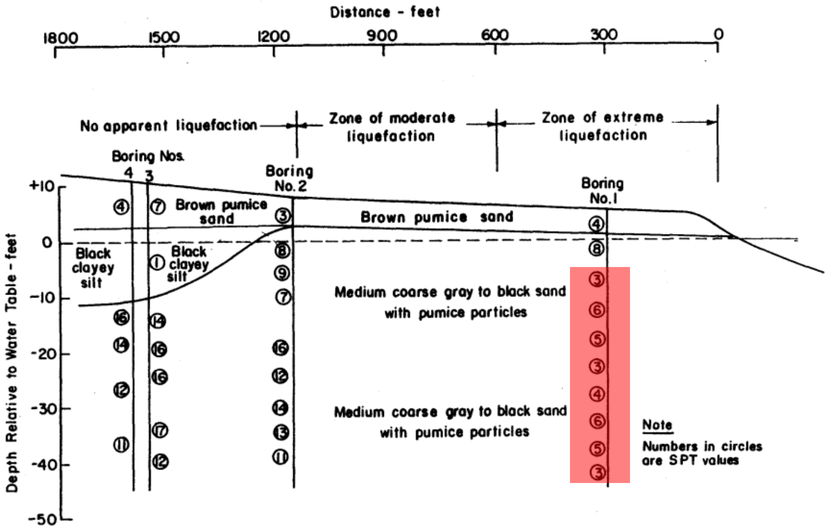

γ_m=60±1 pcf

γ_s=90±1 pcf

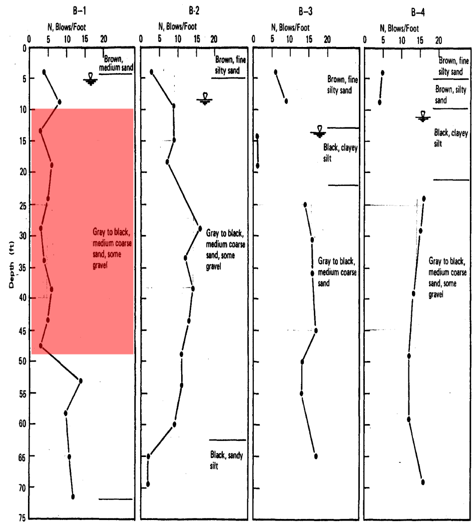

FIG. 10 BORING LOGS AT LA PLAYA SITE

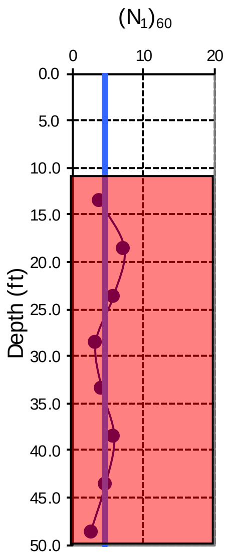

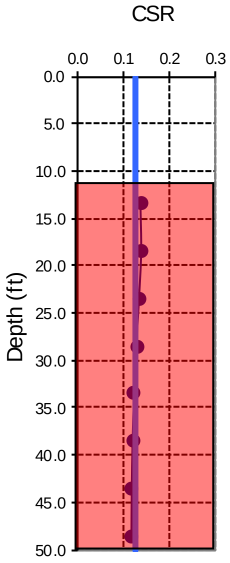

Depth (m)	Depth (ft)	CSR	N	C_N	C_R	$(N1)_{60}$
4.1	13.5	0.141	3	1.99	0.86	***3.8***
5.65	18.5	0.143	6	1.77	0.91	***7.3***
7.2	23.6	0.137	5	1.61	0.96	***5.8***
8.7	28.5	0.131	3	1.49	1.00	***3.3***
10.2	33.5	0.126	4	1.40	1.00	***4.2***
11.75	38.5	0.122	6	1.31	1.00	***5.9***
13.3	43.6	0.120	5	1.24	1.00	***4.7***
14.8	48.6	0.119	3	1.19	1.00	***2.7***
					**Mean:**	4.7
					**St. Dev.**	1.5

**Case number:**	28
**Earthquake:**	1976 Guatemala
**Magnitude:**	7.5 (ML)
**Location:**	Amatitlan B-2
**References:**	Seed et al. (1979)
**Nature of Failure:**	Right at the boundary of zone of moderate liquefaction to nonliquefaction
**Comments:**	The sites are located on deltaic deposits near the mouth of a small river, Rio Villalobos at the settlement of La Playa on the north-east shore of Lake Amatitlan.The general area behind the shoreline is quite flat with the ground sloping gently up about 2 degs away from the lake.<br>Two test pits were dug to a depth of ~ 5' on the level ground 40' from the beach These showed the upper 4' of soil to be pumice sand underlain by a thin layer of dense fibrous peats and organic silt. Two field density test in the pumice sand revealed that it had wet and dry densities ranging from 55.5-61.5 and 35.5-39.4 pcf. respectively. Light-weight pumice sand at all boring locations was located above water table and was not itself the layer of liquefaction.<br>The epicenter was located ~170 kms north-east from Lake Amatitlan<br>However, north terminus of the fault is only 40 kms away from the lake.<br>The Modified Mercalli Intensity of shaking at the site is high VI or low VII. Based on correlations, PGA was estimated as 0.12-0.15 g. This estimation was checked by the level of damage at the nearby structures. Also attenuation relationships confirmed the range. Based on seismoscope records, USGS predicted the PGA at a site ~ 25 km from the causative fault as 0.25 g. Calibrating the attenuation relationships with this information, PGA at the site was predicted as 0.15 g.<br>Local Magnitude saturates at magnitude 7. Thus, it is hard to estimate the moment magnitude for the earthquake. However based on the recorded motion and attenuation relationships, moment magnitude is back calculated as 7-8.2.<br>Rope and pulley system (2 turns of the rope around the pulley) was used.<br>Estimated rod energy is 45%. SPT values were taken after the earthquake.

**Summary of Data**	Cetin et al. (2016)	Idriss&Boulanger (2010)	Seed et.al. (1984)		Cetin et al. (2016)	Idriss&Boulanger (2010)	Seed et.al. (1984)
Liquefied?	No/Yes	Marginal	Yes/No	D_{50}	0.800 ± 0.050		0.8
Data Class	B			% Fines	3.0 ± 2.0	3.0	3
Critical Depth Range	8.0 - 20.0	15.1	15.0	% PI			
Depth to GWT (ft)	8.0	7.9	8.0				
σ_v (psf)	1020.0 ± 180.5	1148.7	1150.0	N	8.0 ± 1.1	8.0	8
σ_v' (psf)	645.6 ± 56.9	710.1	705.0	C_R	0.86	0.95	
a_{max} (g)	0.135 ± 0.041	0.14	0.135	C_S	1.00	1.00	
r_d	0.82 ± 0.061	0.97	0.950	C_B	1.00	1.00	
CSR	0.113 ± 0.037	0.138	0.135	C_E	0.75	0.75	0.75
Equivalent Magnitude	7.5	7.5		C_N	1.81	1.70	1.60
MSF		1.00	1.00	$(N_1)_{60}$	9.3 ± 1.3	9.7	9
CSRN		0.126	0.14				

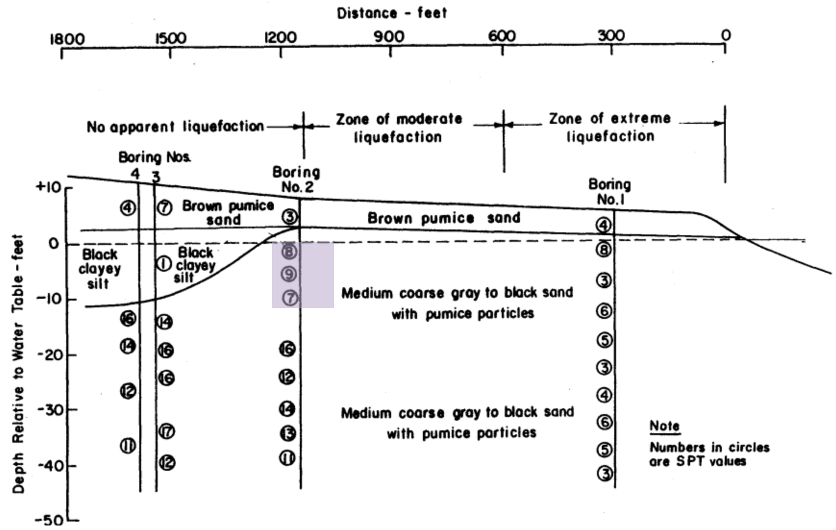

γ_m=60±1 pcf

γ_s=90±1 pcf

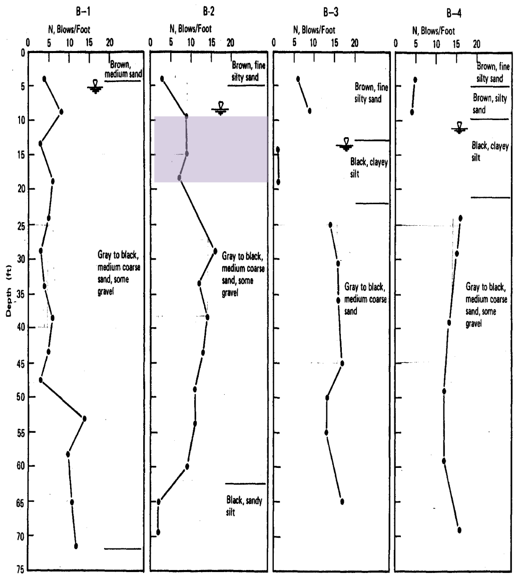

FIG. 10 BORING LOGS AT LA PLAYA SITE

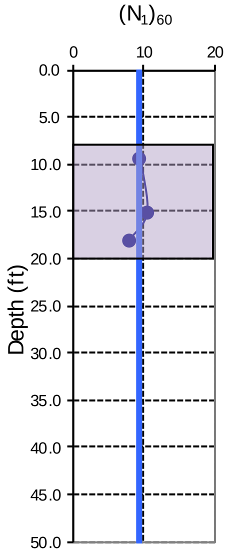

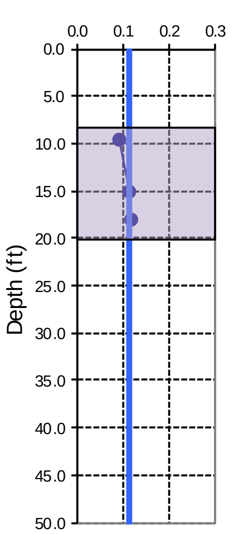

Depth (m)	Depth (ft)	CSR	N	C_N	C_R	$(N1)_{60}$
2.9	9.5	0.092	8	2.00	0.80	***9.6***
4.6	15.1	0.116	9	1.77	0.88	***10.5***
5.5	18.0	0.121	7	1.67	0.91	***8.0***
					**Mean:**	9.3
					**St. Dev.**	1.3

**Case number:**	29
**Earthquake:**	1976 Guatemala
**Magnitude:**	7.5 (ML)
**Location:**	Amatitlan B-3&4
**References:**	Seed et al. (1979)
**Nature of Failure:**	Located at the "no apparent liquefaction" zone
**Comments:**	The sites are located on deltaic deposit near the mouth of a small river, Rio Villalobos at the settlement of La Playa on the north-east shore of Lake Amatitlan.The general area behind the shoreline is quite flat with the ground sloping gently up about 2 degs away from the lake.<br>Two test pits were dug to a depth of ~ 5' on the level ground 40' from the beach These showed the upper 4' of soil to be pumice sand underlain by a thin layer of dense fibrous peats and organic silt. Two field density test in the pumice sand showed that it had wet and dry densities ranging from 55.5-61.5 and 35.5-39.4 pcf respectively. Light-weight pumice sand at all boring locations was located above water table and was not itself the layer of liquefaction.<br>The epicenter was located ~170 kms north-east from Lake Amatitlan<br>However, north terminus of the fault is only 40 kms away from the lake.<br>The Modified Mercalli Intensity of shaking at the site is high VI or low VII. Based on the correlations, PGA was estimated as 0.12-0.15 g. This estimation was checked by the level of damage at the nearby structures. Also attenuation relationships confirmed the range. Based on seismoscope records, USGS predicted the PGA at a site ~ 25 km from the causative fault as 0.25 g. Calibrating the attenuation relationships with this information, PGA at the site is predicted as 0.15 g.<br>Local Magnitude saturates at magnitude 7. Thus, it is hard to estimate the moment magnitude for the earthquake. However based on the recorded motion and attenuation relationships, moment magnitude is back calculated as 7-8.2.<br>Rope and pulley system (2 turns of the rope around the pulley) was used.<br>Estimated rod energy is 45%. SPT values were taken after the earthquake.<br>Amatitlan B-3 & 4 are ~40' apart.

Summary of Data

	Cetin et al. (2016)	Idriss&Boulanger (2010)	Seed et.al. (1984)		Cetin et al. (2016)	Idriss&Boulanger (2010)	Seed et.al. (1984)
Liquefied?	No	No	No	D_{50}	0.800 ± 0.050		0.8
Data Class	C			% Fines	3.0 ± 2.0	3.0	3
Critical Depth Range	22.0 - 45.0	35.1	35.0	% PI			
Depth to GWT (ft)	12.5	11.2	11.0				
σ_v (psf)	2640.3 ± 349.2	2861.3	2860.0	N	15.1 ± 1.9	16.0	16
σ_v' (psf)	1329.3 ± 120.5	1482.9	1490.0	C_R	1.00	1.00	
a_{max} (g)	0.135 ± 0.041	0.14	0.135	C_S	1.00	1.00	
r_d	0.68 ± 0.128	0.89	0.890	C_B	1.00	1.00	
CSR	0.119 ± 0.043	0.149	0.150	C_E	0.75	0.75	0.75
Equivalent Magnitude	7.5	7.5		C_N	1.26	1.19	1.14
MSF		1.00	1.00	$(N_1)_{60}$	14.3 ± 1.8	14.3	14
CSRN		0.144	0.15				

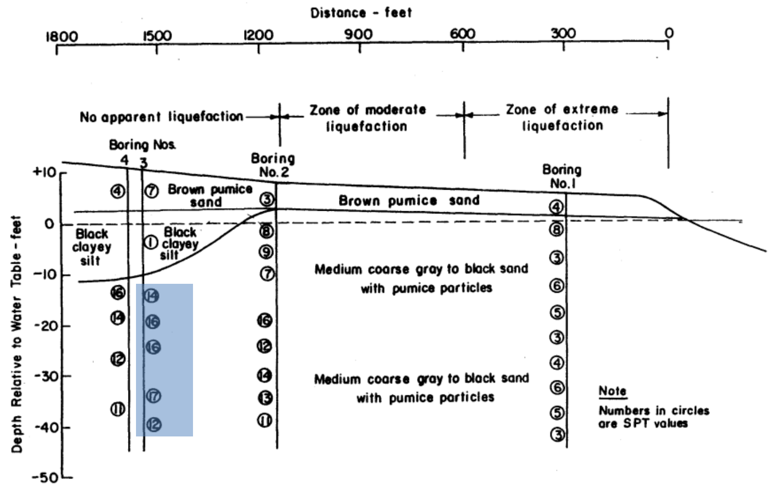

γm=60±1 pcf

γs=90±1 pcf

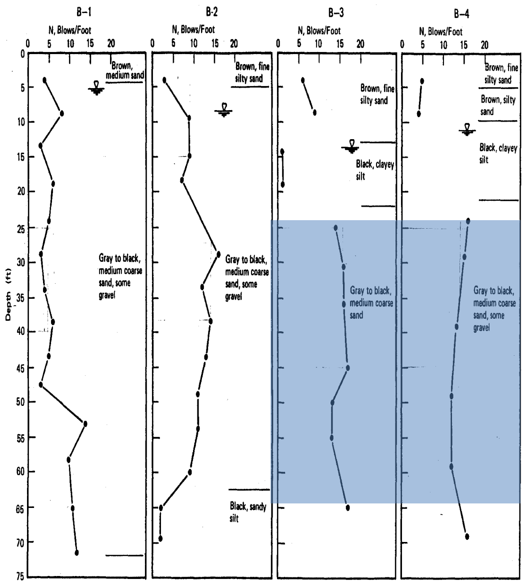

FIG. 10 BORING LOGS AT LA PLAYA SITE

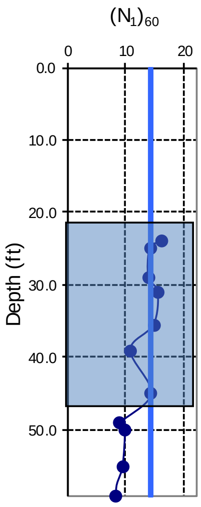

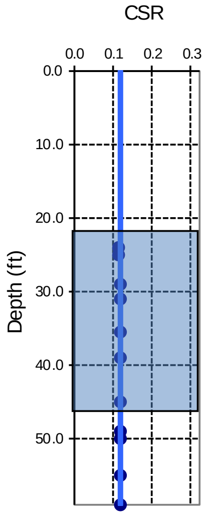

Depth (m)	Depth (ft)	CSR	N	C_N	C_R	$(N1)_{60}$	
7.3	24.0	0.116	16	1.41	0.96	***16.3***	B-4
7.6	25.0	0.117	14	1.39	0.97	***14.2***	B-3
8.8	29.0	0.119	14	1.33	1.00	***13.9***	B-4
9.4	31.0	0.119	16	1.30	1.00	***15.5***	B-3
10.8	35.5	0.119	16	1.24	1.00	***14.8***	B-3
11.9	39.0	0.118	12	1.20	1.00	***10.8***	B-4
13.7	45.0	0.118	17	1.13	1.00	***14.5***	B-3
14.9	49.0	0.118	11	1.10	1.00	9.1	B-4
15.2	50.0	0.118	12	1.09	1.00	9.8	B-3
16.8	55.0	0.120	12	1.05	1.00	9.4	B-3
18.0	59.0	0.121	11	1.02	1.00	8.4	B-4
19.8	65.0	0.123	17	0.98	1.00	12.5	B-3
21.0	69.0	0.124	16	0.96	1.00	11.5	B-4
					**Mean:**	14.3	
					**St. Dev.**	1.8	

**Case number:**	30
**Earthquake:**	1976 Tangshan
**Magnitude:**	7.6 (Mw) USGS Centennial Earthquake Catalog
**Location:**	Coastal Region
**References:**	Shengcong et al (1983)<br>Fear et al. (1995)
**Nature of Failure:**	Liquefied
**Comments:**	This site is located in the south-east of Tangshan City, south of Jing Shan Railway, in zone of liquefaction (Source document, Fig. 11, Qian Jian Yeng?).<br>The site was identified as in the area of 7th grade on the Chinese intensity scale.<br>Consisted essentially of flood plains formed by flooding from Luan and Dou Rivers. Luan river changed its river course frequently. Therefore, loose sand deposits were very common in the region.<br>The Tangshan Earthquake had a focal depth of 12-16 km and epicenter in Tangshan City. The area based on seismic intensity may be divided into two regions by the Jing-Shan railway. The north of the railway is mainly of hill areas, and experienced relatively lower seismic intensity. The south of the railway is mainly of flood plains including plains of marine and continental sediments.<br>PGA was estimated as >0.1 g<br>SPT energy was estimated as 60 % by Seed et al. (84)

Summary of Data

	Cetin et al. (2016)	Idriss&Boulanger (2010)	Seed et.al. (1984)		Cetin et al. (2016)	Idriss&Boulanger (2010)	Seed et.al. (1984)
Liquefied?	Yes	Yes	Yes	D_{50}	0.140 ± 0.050		0.14
Data Class	B			% Fines	12.0 ± 2.0	12.0	12
Critical Depth Range	9.8 - 19.7	14.8	20.0	% PI			
Depth to GWT (ft)	3.6 ± 0.4	3.6	4.0				
σ_v (psf)	1735.6 ± 200.0	1817.0	2440.0	N	9.5 ± 2.2	9.0	10
σ_v' (psf)	1039.5 ± 103.0	1127.8	1440.0	C_R	0.90	0.95	
a_{max} (g)	0.130 ± 0.039	0.13	0.130	C_S	1.00	1.00	
r_d	0.95 ± 0.064	0.97	0.960	C_B	1.00	1.00	
CSR	0.133 ± 0.041	0.130	0.140	C_E	1.00	1.00	1.00
Equivalent Magnitude	7.6	7.6		C_N	1.43	1.37	1.16
MSF		0.97	0.97	$(N_1)_{60}$	12.2 ± 2.9	11.7	11.5
CSRN		0.125	0.15				

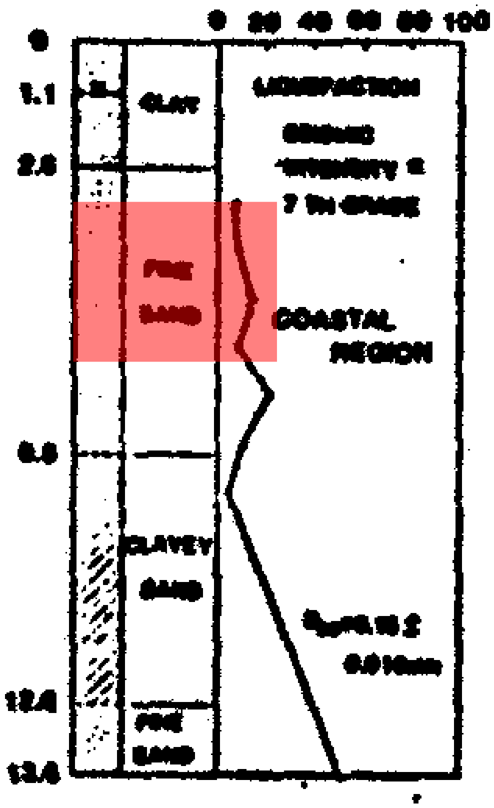

γ_m=110±3 pcf

γ_s=120±3 pcf

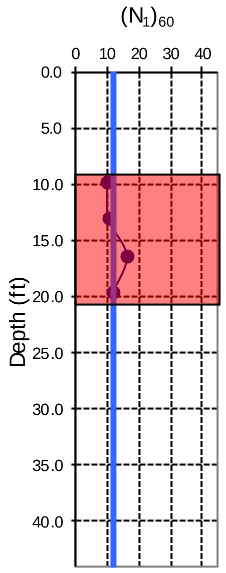

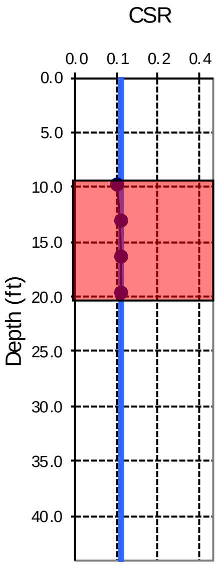

Depth (m)	Depth (ft)	CSR	N	C_N	C_R	$(N1)_{60}$
3.0	9.8	0.124	7.0	1.67	0.85	***9.9***
4.0	13.1	0.131	8.0	1.50	0.89	***10.6***
5.0	16.4	0.135	13.0	1.37	0.92	***16.4***
6.0	19.7	0.135	10.0	1.26	0.95	***12.0***
					**Mean:**	12.2
					**St. Dev.**	2.9

**Case number:**	31
**Earthquake:**	1976 Tangshan
**Magnitude:**	7.6 (Mw) USGS Centennial Earthquake Catalog
**Location:**	Le Ting L8-14
**References:**	Shengcong et al (1983)<br>Fear et al. (1995)
**Nature of Failure:**	Liquefied
**Comments:**	This site is located in the south-east of Tangshan City, south of Jing Shan<br>Railway, in the zone of severe liquefaction (Source doc., Fig. 11, Qian Jian Yeng?).<br>The site was identified as in the area of 7th grade on the Chinese intensity scale.<br>Alluvial (Holocene) deposits.<br>Manifestations of liquefaction were observed in the coastal region, south of<br>Le Ting County.<br>The Tangshan Earthquake had a focal depth of 12-16 km and epicenter<br>in Tangshan City. The area based on seismic intensity may be divided<br>into two regions by the Jing-Shan railway. The north of the railway is mainly<br>of hill areas, and experienced relatively lower seismic intensity. The<br>south of the railway is mainly of flood plains including plains of marine and<br>continental sediments.<br>New borelogs were obtained in addition to the typical borelog (Fear et al, 1995)<br>PGA was estimated as >0.1 g<br>SPT energy was estimated as 60 % by Seed et al. (84)

Summary of Data

	Cetin et al. (2016)	Idriss&Boulanger (2010)	Seed et.al. (1984)		Cetin et al. (2016)	Idriss&Boulanger (2010)	Seed et.al. (1984)
Liquefied?	Yes	Yes	Yes	D_{50}	0.185 ± 0.040		0.22
Data Class	B			% Fines	12.0 ± 2.0	12.0	12
Critical Depth Range	11.5 - 19.7	14.4	6.7	% PI			
Depth to GWT (ft)	3.3 ± 0.6	4.9	5.0				
σ_v (psf)	1837.3 ± 168.5	1691.7	760.0	N	9.9 ± 2.0	9.7	10
σ_v' (psf)	1069.3 ± 92.4	1106.9	655.0	C_R	0.91	0.85	0.75
a_{max} (g)	0.200 ± 0.060	0.20	0.200	C_S	1.00	1.00	
r_d	0.98 ± 0.067	0.97	0.990	C_B	1.00	1.00	
CSR	0.219 ± 0.068	0.194	0.155	C_E	1.00	1.00	1.00
Equivalent Magnitude	7.6	7.6		C_N	1.41	1.39	1.65
MSF		0.97	0.97	$(N_1)_{60}$	12.7 ± 2.6	11.5	12.5
CSRN		0.186	0.16				

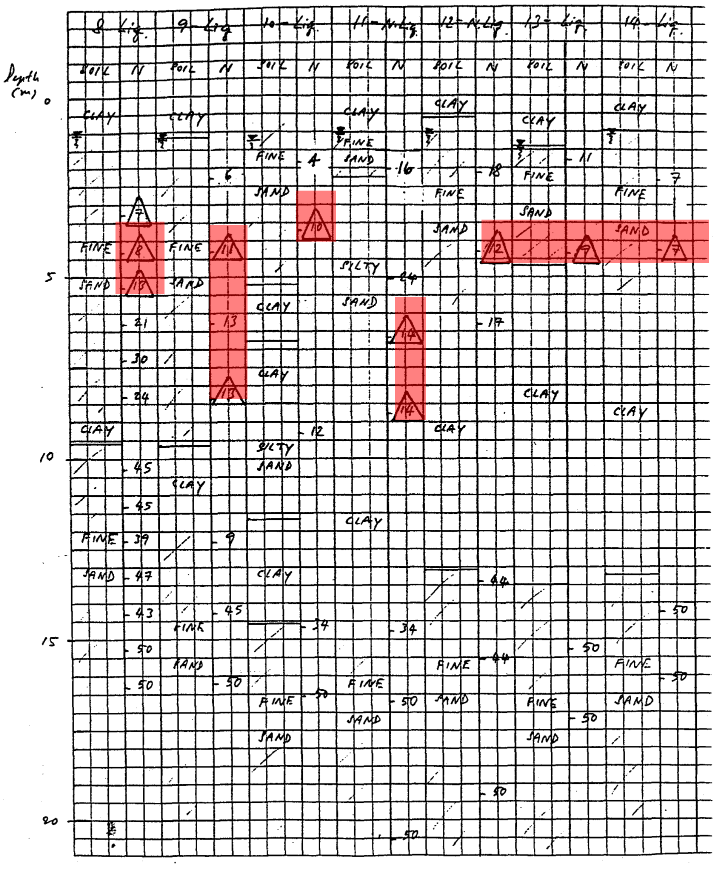

γ_m=110±3 pcf

γ_s=120±3 pcf

137

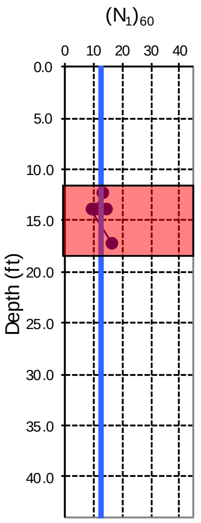

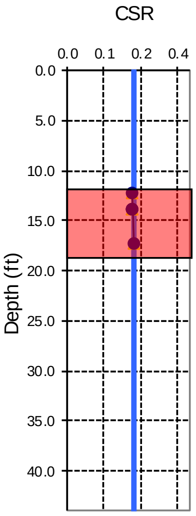

Depth (m)	Depth (ft)	CSR	N	C_N	C_R	$(N1)_{60}$	
3.8	12.3	0.210	10.0	1.55	0.88	***13.6***	L10
4.3	13.9	0.215	8.0	1.47	0.90	***10.6***	L8
4.3	13.9	0.215	11.0	1.47	0.90	***14.5***	L9
4.3	13.9	0.215	9.0	1.47	0.90	***11.9***	L13
4.3	13.9	0.215	7.0	1.47	0.90	***9.2***	L14
5.3	17.2	0.221	13.0	1.35	0.93	***16.3***	L8
					**Mean:**	12.7	
					**St. Dev.**	2.6	

**Case number:**	32
**Earthquake:**	1976 Tangshan
**Magnitude:**	7.6 (Mw) USGS Centennial Earthquake Catalog
**Location:**	Luan Nan-L1
**References:**	Shengcong et al (1983)<br>Fear et al. (1995)
**Nature of Failure:**	No surface evidence of liquefaction
**Comments:**	This site was classified as in the area of 8th grade on the Chinese intensity scale.<br>It is located just south of the Jing Shan Railway, and south-east of Tangshan City, in the zone of liquefaction (Source document, fig.11).<br>The Tangshan Earthquake had a focal depth of 12-16 km, the epicenter was located in Tangshan City. The area based on seismic intensity may be divided into two regions by the Jing-Shan railway. The north of the railway is mainly of hill areas, and experienced relatively lower seismic intensity. The south of the railway is mainly of flood plains including plains of marine and continental sediments.<br>Additional borelogs for this site were obtained from a report by the Beijing Municipal Bureau of City Planning (1982) (Fear et al., 1995). Based on newly available borelogs, it became apparent that representative N value selection by Seed et al (84) can be further improved. Borelog L2 is thought to be a better representative borelog. Probably the medium-fine sand layer liquefied at L2 liquefied first and excess pore pressure dissipated to the neighbouring layers causing them to liquefy as well.<br>Seed et al (84) used borelog L3 (probably the rest were not available)<br>PGA was estimated as >0.1 g<br>SPT energy was estimated as 60 % by Seed et al. (84)

Summary of Data

	Cetin et al. (2016)	Idriss&Boulanger (2010)	Seed et.al. (1984)		Cetin et al. (2016)	Idriss&Boulanger (2010)	Seed et.al. (1984)
Liquefied?	No	No		D_{50}	0.170 ± 0.050		
Data Class	B			% Fines	5.0 ± 2.0	5.0	
Critical Depth Range	4.9 - 18.0	11.5		% PI			
Depth to GWT (ft)	9.4 ± 0.3	3.6					
σ_v (psf)	1201.6 ± 275.0	1294.9		N	19.6 ± 2.9	19.3	
σ_v' (psf)	1068.5 ± 140.4	793.6		C_R	0.87	0.85	
a_{max} (g)	0.220 ± 0.066	0.22		C_S	1.00	1.00	
r_d	0.99 ± 0.052	0.98		C_B	1.00	1.00	
CSR	0.159 ± 0.051	0.226		C_E	1.00	1.00	
Equivalent Magnitude	7.6	7.6		C_N	1.41	1.49	
MSF		0.97		$(N_1)_{60}$	23.9 ± 3.6	24.4	
CSRN		0.211					

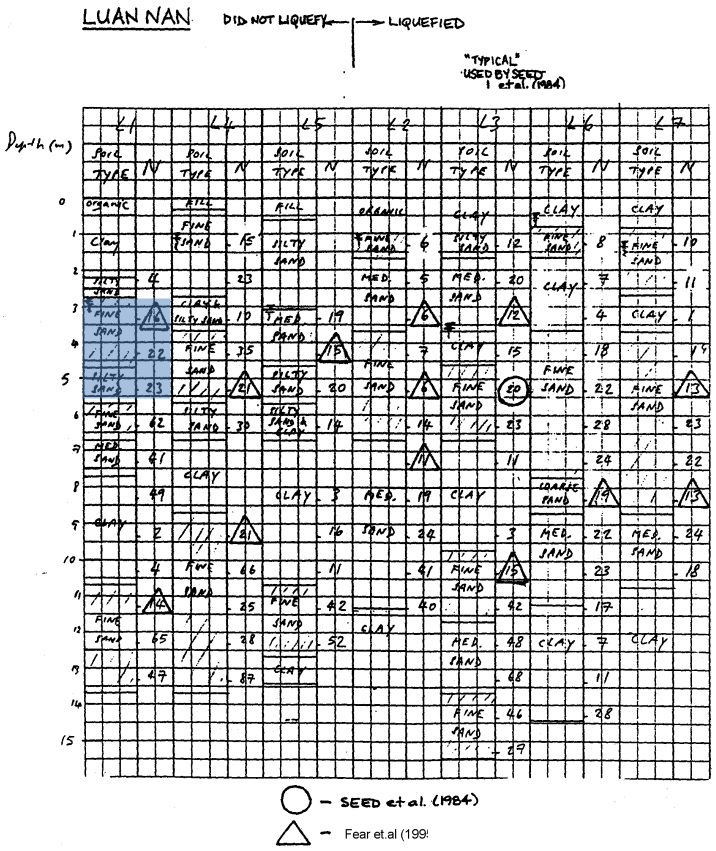

γ_m=100±3 pcf

γ_s=125±3 pcf

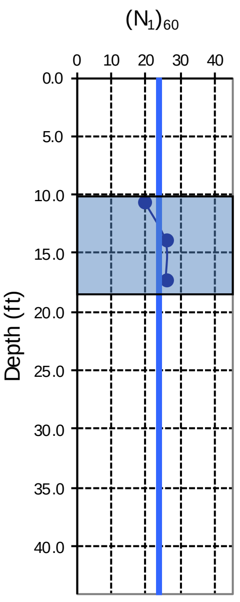

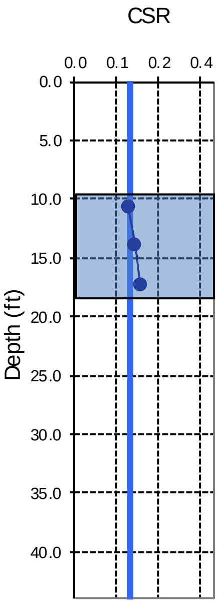

Depth (m)	Depth (ft)	CSR	N	C_N	C_R	$(N1)_{60}$
3.3	10.7	0.153	16.0	1.44	0.86	***19.8***
4.3	13.9	0.174	22.0	1.32	0.90	***25.9***
5.3	17.2	0.188	23.0	1.22	0.93	***26.0***
					**Mean:**	23.9
					**St. Dev.**	3.6

**Case number:**	33
**Earthquake:**	1976 Tangshan
**Magnitude:**	7.6 (Mw) USGS Centennial Earthquake Catalog
**Location:**	Luan Nan-L2
**References:**	Shengcong et al (1983)<br>Fear et al. (1995)
**Nature of Failure:**	Liquefied
**Comments:**	This site was classified as in the area of 8th grade on the Chinese intensity scale.<br>It is located just south of the Jing Shan Railway, and south-east of Tangshan<br>City in the zone of liquefaction (Source document, fig.11)<br>The Tangshan Earthquake had a focal depth of 12-16 km and the epicenter<br>was located in Tangshan City. The area based on seismic intensity may be divided<br>into two regions by the Jing-Shan railway. The north of the railway is mainly<br>of hill areas, and experienced relatively lower seismic intensity. The<br>south of the railway is mainly of flood plains including plains of marine and<br>continental sediments.<br>Additional borelogs for this site were obtained from a report by the Beijing<br>Municipal Bureau of City Planning (1982) (Fear et al., 1995). Based on newly<br>available borelogs, it became apparent that representative N value selection<br>by Seed et al (84) can be further improved. Borelog L2 is thought to be a<br>better representative borelog. Probably the medium-fine sand layer liquefied<br>at L2 liquefied first and excess pore pressure dissipated to the neighbouring<br>layers causing them to liquefy as well.<br>Seed et al (84) used borelog L3 (probably the rest were not available)<br>PGA was estimated as >0.1 g<br>SPT energy was estimated as 60 % by Seed et al. (84)

Summary of Data

	Cetin et al. (2016)	Idriss&Boulanger (2010)	Seed et.al. (1984)		Cetin et al. (2016)	Idriss&Boulanger (2010)	Seed et.al. (1984)
Liquefied?	Yes	Yes		D_{50}	0.170 ± 0.050		
Data Class	B			% Fines	3.0 ± 2.0	3.0	
Critical Depth Range	4.9 - 18.0	11.5		% PI			
Depth to GWT (ft)	3.6 ± 0.3	3.6					
σ_v (psf)	1381.2 ± 274.7	1169.6		N	5.9 ± 0.6	5.9	
σ_v' (psf)	889.9 ± 140.1	668.3		C_R	0.87	0.85	
a_{max} (g)	0.220 ± 0.066	0.22		C_S	1.00	1.00	
r_d	0.98 ± 0.052	0.98		C_B	1.00	1.00	
CSR	0.217 ± 0.067	0.241		C_E	1.00	1.00	
Equivalent Magnitude	7.6	7.6		C_N	1.54	1.70	
MSF		0.97		$(N_1)_{60}$	7.9 ± 0.8	8.5	
CSRN		0.225					

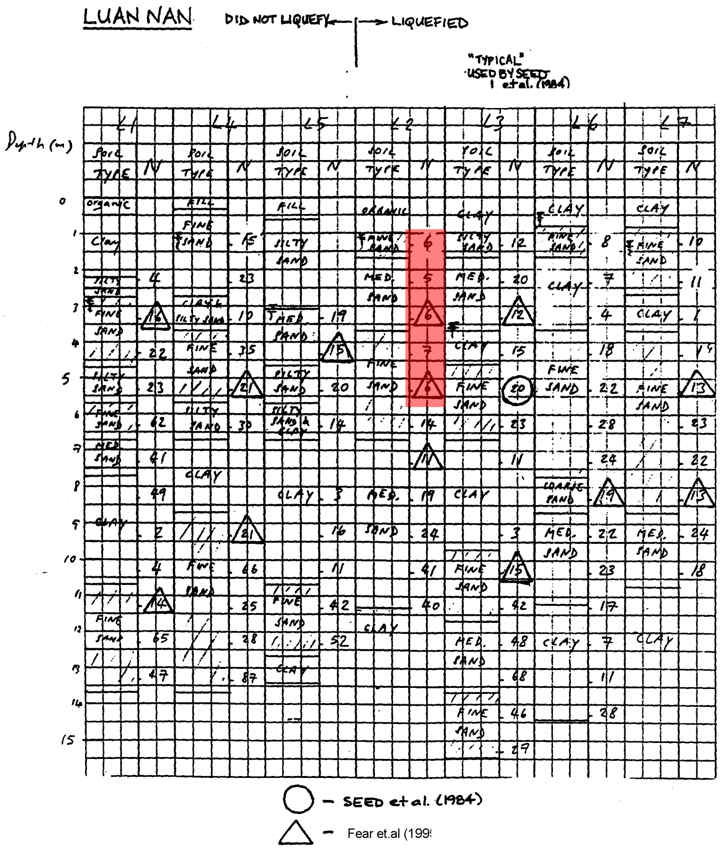

γ_m=100±3 pcf

γ_s=125±3 pcf

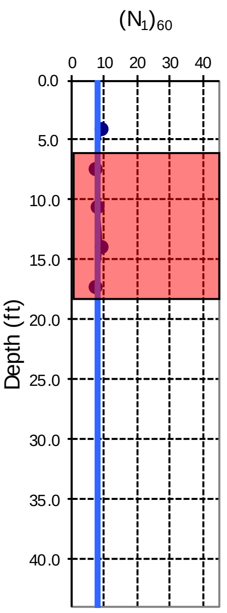

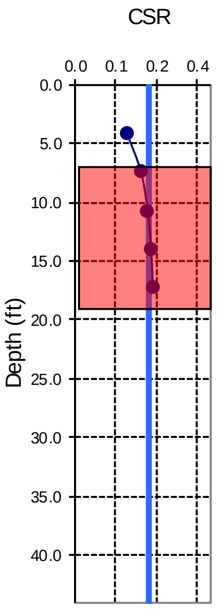

Depth (m)	Depth (ft)	CSR	N	C_N	C_R	$(N1)_{60}$	
1.3	4.1	0.152	6.0	2.00	0.75	9.0	too close to surface
2.3	7.4	0.194	5.0	1.83	0.81	***7.4***	
3.3	10.7	0.214	6.0	1.59	0.86	***8.2***	
4.3	13.9	0.224	7.0	1.42	0.90	***8.9***	
5.3	17.2	0.229	6.0	1.30	0.93	***7.3***	
					**Mean:**	7.9	
					**St. Dev.**	0.8	

**Case number:**	34
**Earthquake:**	1976 Tangshan
**Magnitude:**	7.6 (Mw) USGS Centennial Earthquake Catalog
**Location:**	Qing Jia Ying
**References:**	Shengcong et al (1983)<br>Fear et al. (1995)
**Nature of Failure:**	Liquefied
**Comments:**	This site was classified as in the area of 9th grade on the Chinese intensity scale.<br>It is located in just south of the Jing Shan Railway, and south-east of Tangshan<br>City in the zone of liquefaction (Source document, fig.11., Qian Jia Yeng?)<br>The Tangshan Earthquake had a focal depth of 12-16 km and the epicenter<br>was located in Tangshan City. The area based on seismic intensity may be divided<br>into two regions by the Jing-Shan railway. The north of the railway is mainly<br>of hill areas, and experienced relatively lower seismic intensity. The<br>south of the railway is mainly of flood plains including plains of marine and<br>continental sediments.<br>PGA was estimated as >0.1 g<br>SPT energy was estimated as 60 % by Seed et al. (84)

Summary of Data

	Cetin et al. (2016)	Idriss&Boulanger (2010)	Seed et.al. (1984)		Cetin et al. (2016)	Idriss&Boulanger (2010)	Seed et.al. (1984)
Liquefied?	Yes	Yes	Yes	D_{50}	0.137 ± 0.050		0.137
Data Class	B			% Fines	20.0 ± 2.0	20.0	20
Critical Depth Range	14.8 - 21.3	17.4	17.4	% PI			
Depth to GWT (ft)	3.0 ± 0.4	3.0	3.0				
σ_v (psf)	2211.3 ± 144.4	2130.3	2130.0	N	18.3 ± 2.1	17.0	17.0
σ_v' (psf)	1269.6 ± 84.7	1232.2	1230.0	C_R	0.94	0.95	
a_{max} (g)	0.350 ± 0.105	0.35	0.350	C_S	1.00	1.00	
r_d	0.97 ± 0.076	0.96	0.960	C_B	1.00	1.00	
CSR	0.385 ± 0.120	0.378	0.380	C_E	1.00	1.00	1.000
Equivalent Magnitude	7.6	7.6		C_N	1.29	1.24	1.3
MSF		0.97	0.96	$(N_1)_{60}$	22.1 ± 2.5	20.1	21.50
CSRN		0.357	0.40				

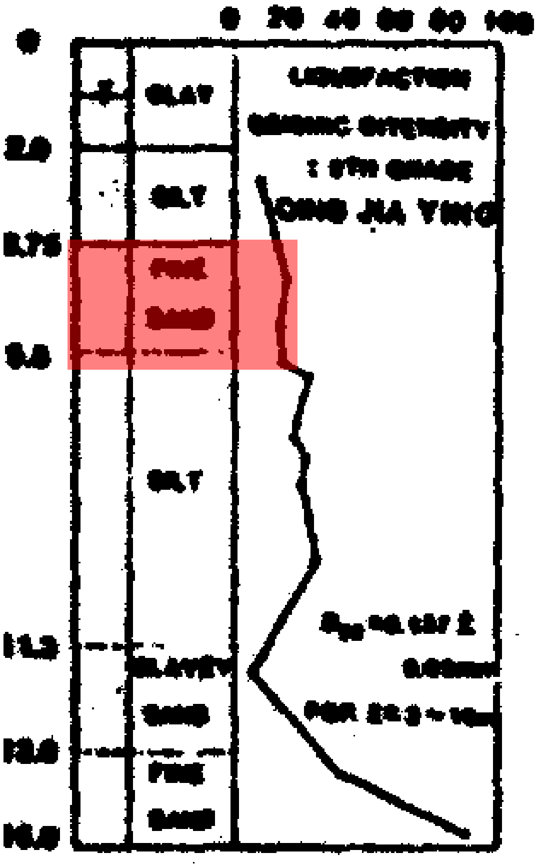

γ_m=110±3 pcf

γ_s=125±3 pcf

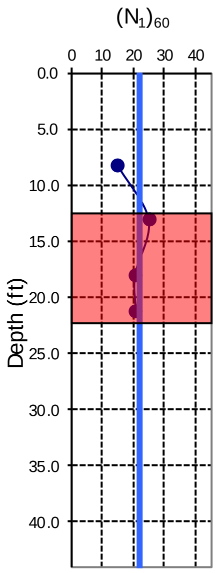

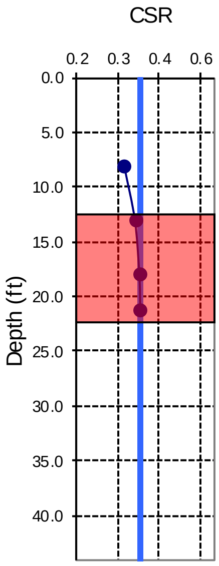

Depth (m)	Depth (ft)	CSR	N	C_N	C_R	$(N1)_{60}$	
2.5	8.2	0.339	10.0	1.80	0.82	14.8	silt-clay contact
4.0	13.1	0.372	19.0	1.48	0.89	***25.0***	
5.5	18.0	0.385	17.0	1.29	0.94	***20.6***	
6.5	21.3	0.389	18.0	1.20	0.96	***20.8***	
					**Mean:**	22.1	
					**St. Dev.**	2.5	

**Case number:**	35
**Earthquake:**	1976 Tangshan
**Magnitude:**	7.6 (Mw) USGS Centennial Earthquake Catalog
**Location:**	Tangshan City
**References:**	Shengcong et al (1983)<br>Fear et al. (1995)
**Nature of Failure:**	Non-liquefied site
**Comments:**	This site was classified as in the area of 11th grade on the Chinese intensity scale.<br>It is located in just north of the Jing Shan Railway, and north-west of the<br>the epicentral area.<br>Sandy layers are generally dense with the relative density values of<br>80 to 90 % and the ground water level was low, at ~ 5-10 m. below the ground<br>surface.<br>The Tangshan Earthquake had a focal depth of 12-16 km and the epicenter<br>was located in Tangshan City. The area based on seismic intensity may be divided<br>into two regions by the Jing-Shan railway. The north of the railway is mainly<br>of hill areas, and experienced relatively lower seismic intensity. The<br>south of the railway is mainly of flood plains including plains of marine and<br>continental sediments.<br>PGA was estimated as >0.1 g<br>SPT energy was estimated as 60 % by Seed et al. (84)

**Summary of Data**	Cetin et al. (2016)	Idriss&Boulanger (2010)	Seed et.al. (1984)		Cetin et al. (2016)	Idriss&Boulanger (2010)	Seed et.al. (1984)
Liquefied?	No	No	No	D_{50}	0.196 ± 0.050		0.196
Data Class	B			% Fines	10.0 ± 2.0	10.0	10
Critical Depth Range	11.5 - 18.0	17.4	17.5	% PI			
Depth to GWT (ft)	9.8 ± 0.3	10.2	10.0				
σ_v (psf)	1697.8 ± 140.7	2046.8	2040.0	N	29.7 ± 5.0	30.0	30.0
σ_v' (psf)	1390.7 ± 77.3	1566.4	1570.0	C_R	0.90	0.95	
a_{max} (g)	0.500 ± 0.150	0.50	0.500	C_S	1.00	1.00	
r_d	1.00 ± 0.064	0.96	0.960	C_B	1.00	1.00	
CSR	0.395 ± 0.122	0.405	0.405	C_E	1.00	1.00	1.000
Equivalent Magnitude	7.6	7.6		C_N	1.23	1.11	1.1
MSF		0.97	0.96	$(N_1)_{60}$	33.1 ± 5.6	31.6	33.50
CSRN		0.389	0.42				

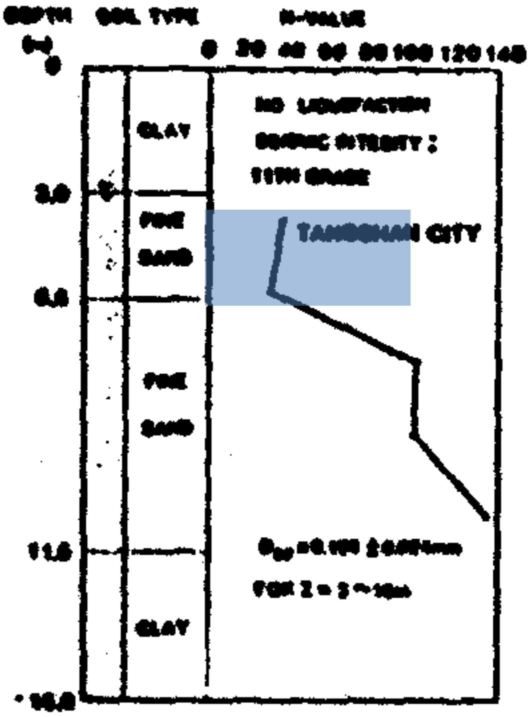

γ_m=110±3 pcf

γ_s=125±3 pcf

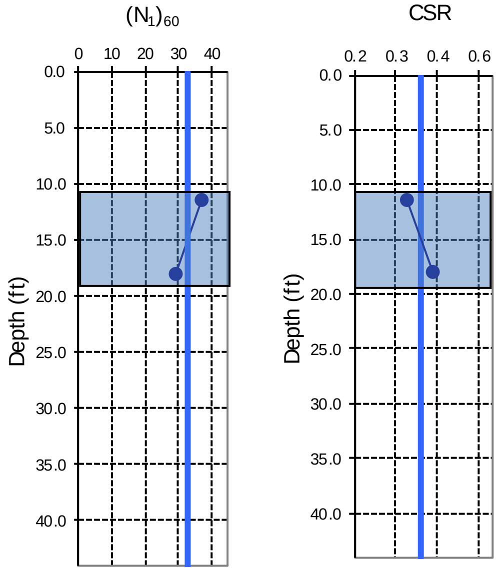

Depth (m)	Depth (ft)	CSR	N	C_N	C_R	$(N1)_{60}$
3.5	11.5	0.352	32.0	1.34	0.87	*37.1*
5.5	18.0	0.427	27.0	1.15	0.94	*29.1*
					**Mean:**	33.1
					**St. Dev.**	5.6

**Case number:**	36
**Earthquake:**	1976 Tangshan
**Magnitude:**	7.6 (Mw) USGS Centennial Earthquake Catalog
**Location:**	Yao Yuan Village
**References:**	Shengcong et al (1983)<br>Fear et al. (1995)
**Nature of Failure:**	Sand swept in the canal; depression due to liquefaction.
**Comments:**	An extraordinary level of damage to one-story house due to soil liquefaction was observed in Yao Yuan Village, near Wang Tan, Le Ting County. A big sand boil into a canal due to soil liquefaction was observed. Correspondingly, a big depression appeared. The length of the depression was around 30m, the width and depth were around 10 m and 4m respectively. Assuming that the groundwater level was the same with the water level in the canal (at 1 m depth from the ground surface), the maximum pore pressure in the liquefied sand layer at depth of 2 m was estimated as ~ 30kN/m2 which was 3 times as large as the water pressure in the canal. It seems that this pressure gradient induced the horizontal migration of pore water, which was easier to occur than the vertical migration due to "impermeable" top clay layer.<br>PGA was estimated as >0.1 g<br>SPT energy was estimated as 60 % by Seed et al. (84)

Summary of Data

	Cetin et al. (2016)	Idriss&Boulanger (2010)	Seed et.al. (1984)		Cetin et al. (2016)	Idriss&Boulanger (2010)	Seed et.al. (1984)
Liquefied?	Yes	Yes	Yes	D_{50}	0.150 ± 0.050		Silt and sand
Data Class	B			% Fines	20.0 ± 2.0	20.0	Silt and sand
Critical Depth Range	11.5 - 16.4	20.0	20.0	% PI			
Depth to GWT (ft)	3.3 ± 0.4	3.0	3.0				
σ_v (psf)	1693.7 ± 108.0	2464.5	2455.0	N	8.7 ± 3.8	9.0	9.0
σ_v' (psf)	1028.4 ± 63.5	1399.3	1395.0	C_R	0.90	0.95	
a_{max} (g)	0.200 ± 0.060	0.20	0.200	C_S	1.00	1.00	
r_d	0.97 ± 0.061	0.95	0.960	C_B	1.00	1.00	
CSR	0.208 ± 0.064	0.218	0.220	C_E	1.00	1.00	1.000
Equivalent Magnitude	7.6	7.6		C_N	1.43	1.22	1.2
MSF		0.97	0.96	$(N_1)_{60}$	11.1 ± 4.9	10.5	11.00
CSRN		0.214	0.23				

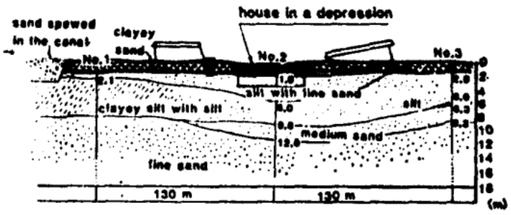

Fig. 19. House sunk into ground, Yao Yuan village, Le Ting county, induced during Tangshan earthquake (reproduced from Fang et al, 1981)

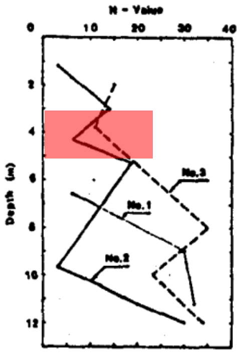

γ_m=110±3 pcf

γ_s=125±3 pcf

Fig. 20. *N*-values measured at Yao Yuan village, Le Ting county (reproduced from Fang et al, 1981)

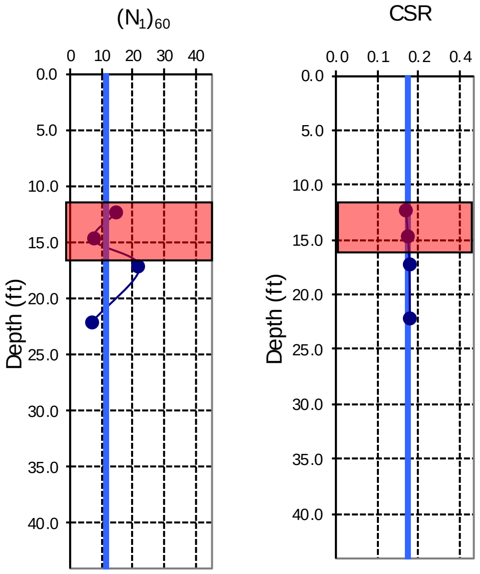

Depth (m)	Depth (ft)	CSR	N	C_N	C_R	$(N1)_{60}$	
3.8	12.3	0.204	11.0	1.51	0.88	***14.6***	No:3 Silt
4.5	14.8	0.209	6.0	1.40	0.90	***7.6***	No:2 Silt with fine sand
5.3	17.2	0.212	18.0	1.31	0.93	21.9	No:2 Silt with fine sand
6.8	22.1	0.213	6.0	1.17	0.97	6.8	No:1 Clayey silt
					**Mean:**	11.1	
					**St. Dev.**	4.9	

**Case number:**	37
**Earthquake:**	1977 Argentina
**Magnitude:**	7.5 (Mw) USGS Centennial Earthquake Catalog
**Location:**	San Juan B-1
**References:**	Idriss et al. (1979)<br>Fear et al. (1995)
**Nature of Failure:**	At several locations, the surface manifestation of liquefaction has been observed in association with abandoned fluvial deposits
**Comments:**	The boreholes were drilled at Dept. of Caucete in the Barrio Justo P. Castro, newly constructed single family stucco homes. The borehole is located northeast of Crevasse.<br>Ground response analyses were performed based on generalized soil profile:<br>0-700 ft : sand with lenses of silt and clay<br>700-1400 ft : gravel<br>>1400 ft : limestone, claystone and sand stone<br>Shear moduli values were estimated from SPT values; Borehole1,3&6 and 2,4&5 were grouped for the analyses.<br>The drainage was prevented at the surface by the clay layer.<br>PGA record was made available by INPRES in San Juan and corrected by USGS.<br>A PGA of 0.187 g was recorded at the seismographic station which was located within 10 km of the study area.<br>Average unit weight of soil was estimated as 110 pcf.<br>Dry Unit weight was reported in the range of 86-93 pcf for silty clay (CL) layer in Table B-1.<br>Estimated rod energy was 45%.<br>N values were back calculated from N_1 plots by using above summarized info.

**Summary of Data**	Cetin et al. (2016)	Idriss&Boulanger (2010)	Seed et.al. (1984)		Cetin et al. (2016)	Idriss&Boulanger (2010)	Seed et.al. (1984)
Liquefied?	Yes	Yes	Yes	D_{50}	0.140 ± 0.050		0.15
Data Class	B			% Fines	20.0 ± 2.0	20.0	20
Critical Depth Range	26.0 - 28.0	26.9	27.0	% PI			
Depth to GWT (ft)	15.0	15.1	15.0				
σ_v (psf)	3150.0 ± 71.4	2965.7	2970.0	N	9.0 ± 2.9	9.0	9.0
σ_v' (psf)	2401.2 ± 64.2	2213.9	2220.0	C_R	0.98	0.95	↑
a_{max} (g)	0.200 ± 0.030	0.20	0.200	C_S	1.00	1.00	
r_d	0.86 ± 0.107	0.92	0.940	C_B	1.00	1.00	
CSR	0.146 ± 0.029	0.160	0.165	C_E	0.75	0.75	↓ 0.750
Equivalent Magnitude	7.5	7.5		C_N	0.94	0.97	1.0
MSF		1.00	0.97	$(N_1)_{60}$	6.2 ± 2.0	6.3	6.00
CSRN		0.161	0.16				

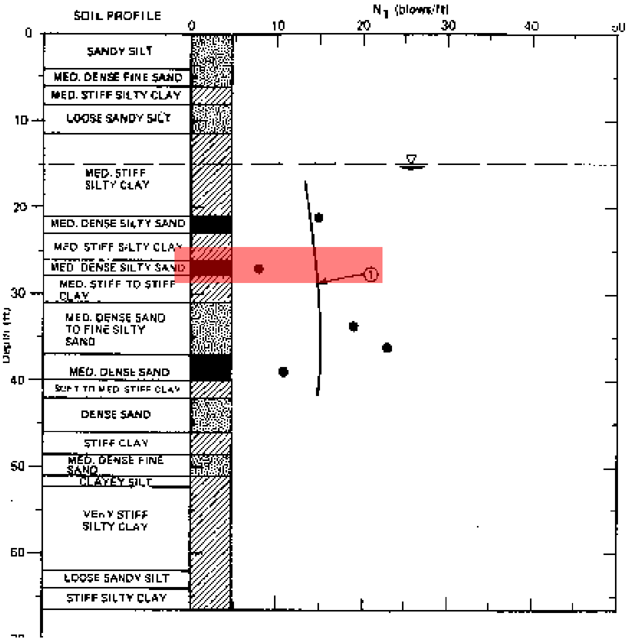

γ_m=110±3 pcf

γ_s=125±3 pcf

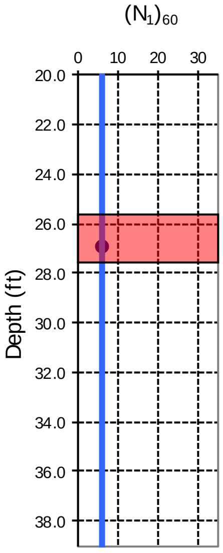

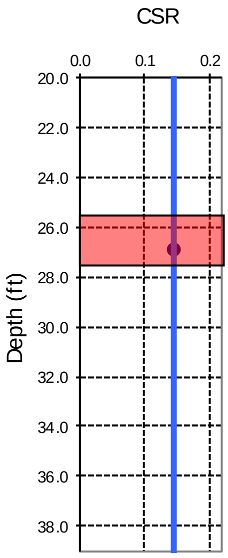

Depth (m)	Depth (ft)	CSR	N	C_N	C_R	$(N1)_{60}$
8.2	26.9	0.146	9	0.94	0.98	***6.2***
				**Mean:**	6.2	
				**St. Dev.**	2.0	assigned

**Case number:**	38
**Earthquake:**	1977 Argentina
**Magnitude:**	7.5 (Mw) USGS Centennial Earthquake Catalog
**Location:**	San Juan B-3
**References:**	Idriss et al. (1979)<br>Fear et al. (1995)
**Nature of Failure:**	At several locations, the surface manifestation of liquefaction has been observed in association with abandoned fluvial deposits.
**Comments:**	The borehole was located in north of downtown Caucete on private property which suffered substantial damage during the earthquake.<br>Ground response analyses were performed based on generalized soil profile:<br>0-700 ft : sand with lenses of silt and clay<br>700-1400 ft : gravel<br>>1400 ft : limestone, claystone and sand stone<br>Shear moduli values were estimated from SPT values; Borehole1,3&6 and 2,4&5 were grouped for the analyses.<br>The drainage was prevented at the surface by the clay layer.<br>PGA record was made available by INPRES in San Juan and corrected by USGS.<br>A PGA of 0.187 g was recorded at the subject seismographic station which was located within 10 km of the study area.<br>Average unit weight of soil was estimated as 110 pcf.<br>Dry Unit weight was reported in the range of 86-93 pcf in Table B-1.<br>Estimated rod energy was 45%<br>N values are back calculated from N1 plots by using above summarized info.

**Summary of Data**	Cetin et al. (2016)	Idriss&Boulanger (2010)	Seed et.al. (1984)		Cetin et al. (2016)	Idriss&Boulanger (2010)	Seed et.al. (1984)
Liquefied?	Yes	Yes	Yes	D_{50}	0.140 ± 0.050		SP
Data Class	B			% Fines	20.0 ± 2.0	5.0	
Critical Depth Range	33.5 - 43.0	36.4	39.0	% PI			
Depth to GWT (ft)	22.0	22.0	22.0				
σ_v (psf)	4370.0 ± 207.0	4156.2	3190.0	N	14.1 ± 2.5	13.0	12.0
σ_v' (psf)	3356.0 ± 123.7	3258.1	2130.0	C_R	1.00	1.00	
a_{max} (g)	0.200 ± 0.030	0.20	0.200	C_S	1.00	1.00	
r_d	0.71 ± 0.144	0.87	0.860	C_B	1.00	1.00	
CSR	0.120 ± 0.030	0.169	0.165	C_E	0.75	0.75	0.750
Equivalent Magnitude	7.5	7.5		C_N	0.79	0.78	1.0
MSF		1.00	0.97	$(N_1)_{60}$	8.4 ± 1.5	7.6	8.50
CSRN		0.169	0.16				

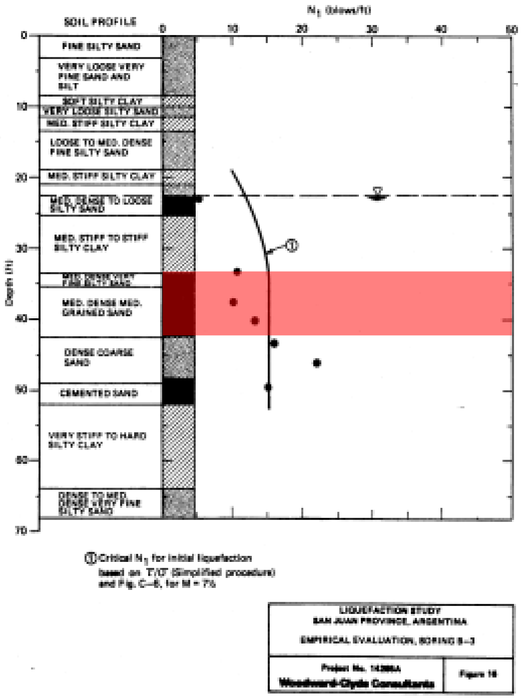

γ_m=110±3 pcf

γ_s=120±3 pcf

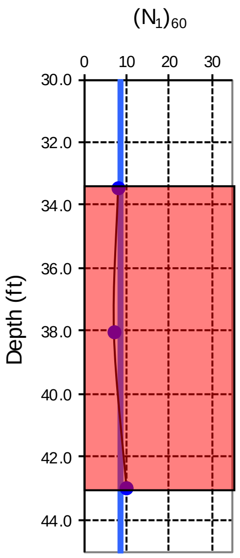

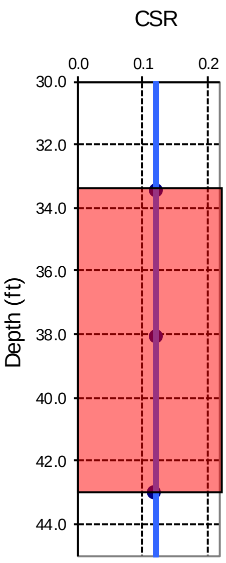

Depth (m)	Depth (ft)	CSR	N	C_N	C_R	$(N1)_{60}$
10.2	33.5	0.120	13	0.83	1.00	***8.1***
11.6	38.1	0.120	12	0.80	1.00	***7.2***
13.1	43.0	0.119	17.5	0.76	1.00	***10.0***
					**Mean:**	8.4
					**St. Dev.**	1.5

**Case number:**	39
**Earthquake:**	1977 Argentina
**Magnitude:**	7.5 (Mw) USGS Centennial Earthquake Catalog
**Location:**	San Juan B-4
**References:**	Idriss et al. (1979)<br>Fear et al. (1995)
**Nature of Failure:**	There is no surface evidence of liquefaction
**Comments:**	The borehole is located in the Dept. of 9 de Julio in the Finca Delta; It is at the corner of vineyard.<br>Ground response analyses were performed based on generalized soil profile:<br>0-700 ft : sand with lenses of silt and clay<br>700-1400 ft : gravel<br>>1400 ft : limestone, claystone and sand stone<br>Shear moduli values were estimated from SPT values; Borehole1,3&6 and 2,4&5 were grouped for the analyses.<br>PGA record was made available by INPRES in San Juan and corrected by USGS.<br>A PGA of 0.187 g was recorded at the seismographic station which was located within 10 km of the study area.<br>Average unit weight of soil was estimated as 110 pcf.<br>Dry Unit weight was reported in the range of 86-93 pcf in Table B-1.<br>Estimated rod energy was 45%<br>N values were back calculated from N_1 plots by using above summarized info.

**Summary of Data**	Cetin et al. (2016)	Idriss&Boulanger (2010)	Seed et.al. (1984)		Cetin et al. (2016)	Idriss&Boulanger (2010)	Seed et.al. (1984)
Liquefied?	No	No	No	D_{50}	0.290 ± 0.050		0.29
Data Class	B			% Fines	4.0 ± 2.0	4.0	4
Critical Depth Range	4.0 - 12.0	12.1	12.0	% PI			
Depth to GWT (ft)	4.0	3.9	4.0				
σ_v (psf)	940.0 ± 167.6	1315.8	1320.0	N	13.6 ± 0.7	14.0	14.0
σ_v' (psf)	690.4 ± 86.4	814.5	820.0	C_R	0.77	0.85	
a_{max} (g)	0.200 ± 0.030	0.20	0.200	C_S	1.00	1.00	
r_d	0.98 ± 0.038	0.98	0.970	C_B	1.00	1.00	
CSR	0.173 ± 0.029	0.204	0.200	C_E	0.75	0.75	0.750
Equivalent Magnitude	7.5	7.5		C_N	1.75	1.60	1.5
MSF		1.00	0.98	$(N_1)_{60}$	13.8 ± 0.7	14.3	16.00
CSRN		0.186	0.20				

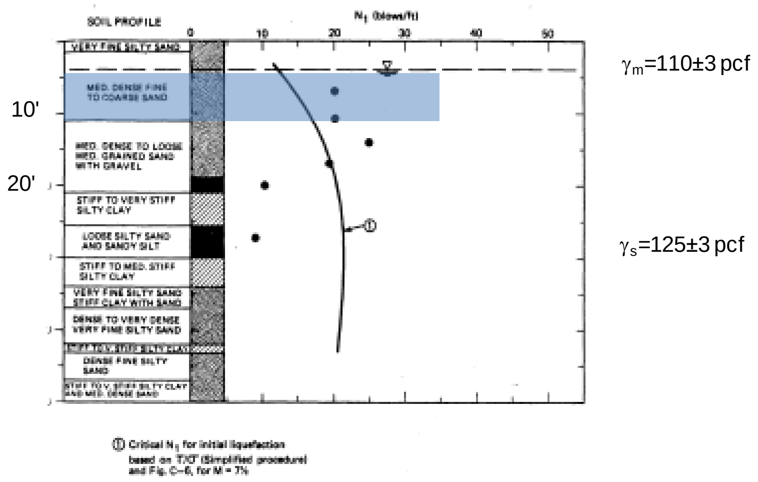

LIQUEFACTION STUDY
SAN JUAN PROVINCE, ARGENTINA

EMPIRICAL EVALUATION, BORING B-4

Woodward-Clyde Consultants | Figure 17

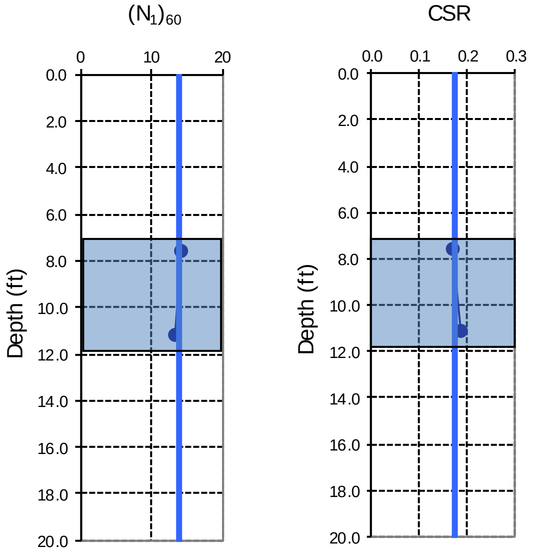

Depth (m)	Depth (ft)	CSR	N	C_N	C_R	$(N1)_{60}$
2.3	7.5	0.170	14	1.79	0.76	***14.3***
3.4	11.2	0.188	14	1.54	0.82	***13.3***
					**Mean:**	13.8
					**St. Dev.**	0.7

**Case number:**	40
**Earthquake:**	1977 Argentina
**Magnitude:**	7.5 (Mw) USGS Centennial Earthquake Catalog
**Location:**	San Juan B-5
**References:**	Idriss et al. (1979)<br>Fear et al. (1995)
**Nature of Failure:**	No apparent damage at nearby structures; no sand boils or other surface evidence of liquefaction.
**Comments:**	The borehole was located in the Dept. of 9 de Julio in the Finca La Porfia about 1.5 km east of route 20.<br>It was drilled near the center of an old filled-in tributary channel.<br>Ground response analyses were performed based on generalized soil profile:<br>0-700 ft : sand with lenses of silt and clay<br>700-1400 ft : gravel<br>>1400 ft : limestone, claystone and sand stone<br>Shear moduli values were estimated from SPT values; Borehole1,3&6 and 2,4&5 were grouped for the analyses.<br>PGA record was made available by INPRES in San Juan and corrected by USGS.<br>A PGA of 0.187 g was recorded at the subject seismographic station which was located within 10 km of the study area.<br>Average unit weight of soil was estimated as 110 pcf.<br>Dry Unit weight was reported in the range of 86-93 pcf in Table B-1.<br>Estimated rod energy was 45%<br>N values were back calculated from N_1 plots by using above summarized info.

Summary of Data

	Cetin et al. (2016)	Idriss&Boulanger (2010)	Seed et.al. (1984)		Cetin et al. (2016)	Idriss&Boulanger (2010)	Seed et.al. (1984)
Liquefied?	No	No	No	D_{50}	0.240 ± 0.050		0.24
Data Class	B			% Fines	3.0 ± 2.0	3.0	3
Critical Depth Range	7.0 - 12.0	10.2	10.0	% PI			
Depth to GWT (ft)	7.0	6.9	7.0				
σ_v (psf)	1082.5 ± 106.6	1106.9	1100.0	N	15.3 ± 0.1	14.0	14.0
σ_v' (psf)	926.5 ± 58.5	919.0	910.0	C_R	0.80	0.85	
a_{max} (g)	0.200 ± 0.030	0.20	0.200	C_S	1.00	1.00	
r_d	0.99 ± 0.044	0.98	0.980	C_B	1.00	1.00	
CSR	0.150 ± 0.024	0.154	0.155	C_E	0.75	0.75	0.750
Equivalent Magnitude	7.5	7.5		C_N	1.51	1.53	1.4
MSF		1.00	0.97	$(N_1)_{60}$	13.8 ± 0.1	13.6	14.50
CSRN		0.142	0.15				

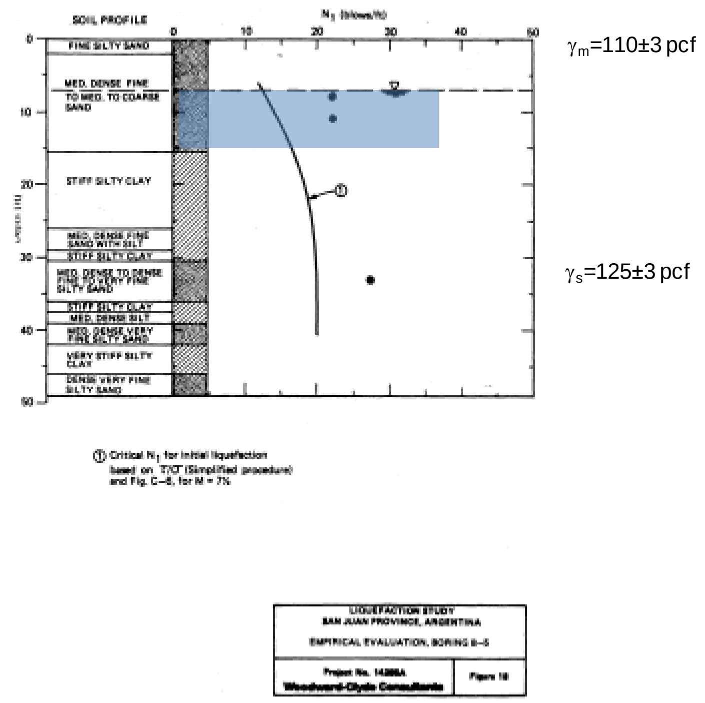

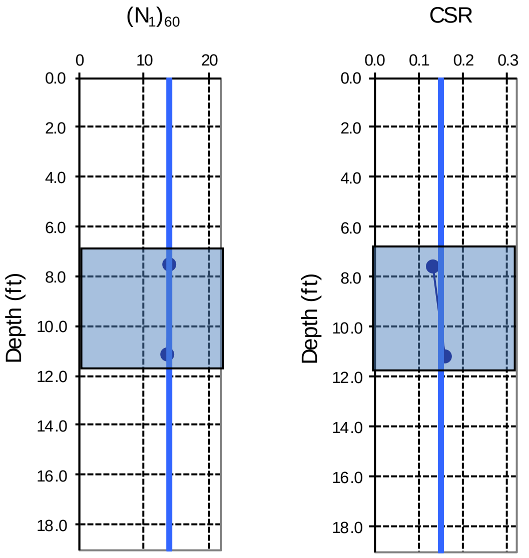

Depth (m)	Depth (ft)	CSR	N	C_N	C_R	$(N1)_{60}$
2.3	7.5	0.134	15	1.62	0.76	***13.9***
3.4	11.2	0.160	15.5	1.43	0.82	***13.7***
					**Mean:**	13.8
					**St. Dev.**	0.1

**Case number:**	41
**Earthquake:**	1977 Argentina
**Magnitude:**	7.5 (Mw) USGS Centennial Earthquake Catalog
**Location:**	San Juan B-6
**References:**	Idriss et al. (1979)<br>Fear et al. (1995)
**Nature of Failure:**	Nearby structures were damaged; sand boils; stairway collapsed, concrete posts were sheared off.
**Comments:**	The borehole is located in the Dept. of 25 de Mayo at the Finca Santiago adjacent to La Plata Highway.<br>Ground response analyses were performed based on generalized soil profile:<br>0-700 ft : sand with lenses of silt and clay<br>700-1400 ft : gravel<br>>1400 ft : limestone, claystone and sand stone<br>Shear moduli values were estimated from SPT values; Borehole1,3&6 and 2,4&5 were grouped for the anayses.<br>PGA record was made available by INPRES in San Juan and corrected by USGS.<br>A PGA of 0.187 g was recorded at the seismographic station which was located within 10 km of the study area.<br>Average unit weight of soil was estimated as 110 pcf.<br>Dry Unit weight was reported in the range of 86-93 pcf in Table B-1.<br>Estimated rod energy was 45%<br>N values were back calculated from N_1 plots by using above summarized info.

Summary of Data

	Cetin et al. (2016)	Idriss&Boulanger (2010)	Seed et.al. (1984)		Cetin et al. (2016)	Idriss&Boulanger (2010)	Seed et.al. (1984)
Liquefied?	Yes	Yes	Yes	D_{50}	0.100 ± 0.050		SM-ML
Data Class	B			% Fines	50.0 ± 2.0	50.0	50
Critical Depth Range	12.0 - 18.0	17.1	17.0	% PI			
Depth to GWT (ft)	6.0	5.9	6.0				
σ_v (psf)	1740.0 ± 124.3	1879.7	1870.0	N	6.0 ± 0.2	6.0	6.0
σ_v' (psf)	1178.4 ± 68.0	1169.6	1180.0	C_R	0.87	0.95	
a_{max} (g)	0.200 ± 0.030	0.20	0.200	C_S	1.00	1.00	
r_d	0.96 ± 0.065	0.96	0.960	C_B	1.00	1.00	
CSR	0.184 ± 0.031	0.198	0.195	C_E	0.75	0.75	0.750
Equivalent Magnitude	7.5	7.5		C_N	1.34	1.36	1.3
MSF		1.00	0.97	$(N_1)_{60}$	5.3 ± 0.2	5.8	6.00
CSRN		0.187	0.19				

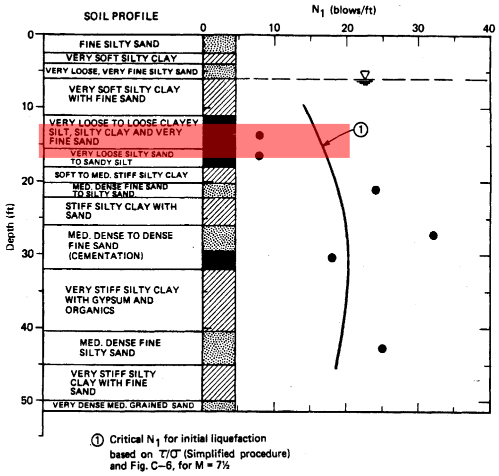

γ_m=110±3 pcf

γ_s=120±3 pcf

LIQUEFACTION STUDY<br>SAN JUAN PROVINCE, ARGENTINA<br>EMPIRICAL EVALUATION, BORING B-6	
Project No. 14395A<br>Woodward-Clyde Consultants	Figure 19

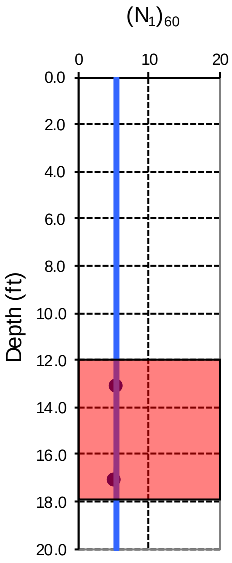

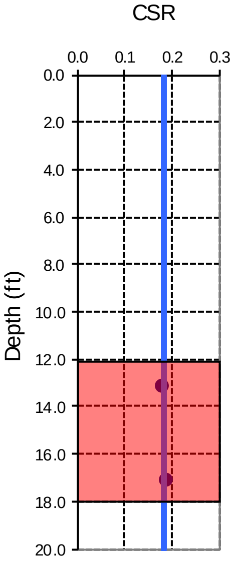

Depth (m)	Depth (ft)	CSR	N	C_N	C_R	$(N1)_{60}$
4	13.1	0.178	6	1.41	0.85	***5.4***
5.2	17.1	0.189	6	1.28	0.90	***5.2***
					**Mean:**	5.3
					**St. Dev.**	0.2

**Case number:**	42
**Earthquake:**	1978 Miyagiken-Oki
**Magnitude:**	6.5
**Location:**	Arahama
**References:**	Tohno et al. (1981)<br>Fear et al. (1995)
**Nature of Failure:**	No apparent evidence of liquefaction
**Comments:**	The site is located in Watari Town, district of Arahama.<br>Prior to the main shock of M=7.4, an earthquake of magnitude 6.7 occurred.<br>Only the Nakamura site liquefied during this earthquake.<br>The subject zone is seismically very active and had been shaken in 1897 and<br>1936 by earthquakes of Magnitudes 7.3 and 7.7<br>Arahama area is covered with very soft sandy deposits; about 9 m in thickness,<br>N values generally less than 10.<br>SPT values were taken before main earthquake (M=7.4) in 1974<br>SPT energy is estimated as 65 % by Seed et al. (84)

**Summary of Data**	Cetin et al. (2016)	Idriss&Boulanger (2010)	Seed et.al. (1984)		Cetin et al. (2016)	Idriss&Boulanger (2010)	Seed et.al. (1984)
Liquefied?	No	No	No	D_{50}	0.450 ± 0.050		0.6
Data Class	B			% Fines	0.0 ± 2.0	0.0	0
Critical Depth Range	6.6 - 26.2	21.0	21.0	% PI			
Depth to GWT (ft)	3.0 ± 0.3	3.0	3.0				
σ_v (psf)	1938.5 ± 395.9	2527.1	2520.0	N	8.9 ± 2.4	10.0	10.0
σ_v' (psf)	1102.1 ± 194.1	1399.3	1400.0	C_R	0.92	0.95	
a_{max} (g)	0.100 ± 0.030	0.10	0.100	C_S	1.00	1.00	
r_d	0.94 ± 0.070	0.90	0.950	C_B	1.00	1.00	
CSR	0.107 ± 0.033	0.105	0.110	C_E	1.09	1.09	1.090
Equivalent Magnitude	6.5	6.5		C_N	1.39	1.23	1.2
MSF		1.34	1.16	$(N_1)_{60}$	12.4 ± 3.3	12.8	13.00
CSRN		0.076	0.10				

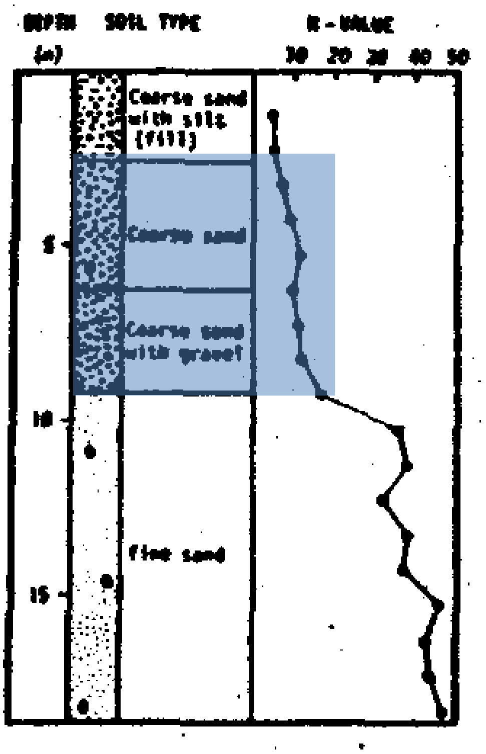

γ_m=110±3 pcf

γ_s=120±3 pcf

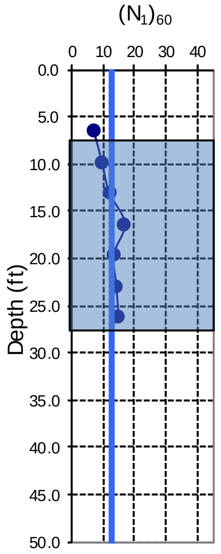

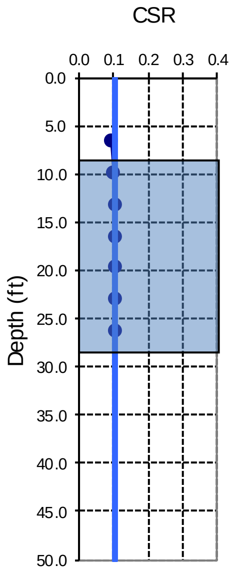

Depth (m)	Depth (ft)	CSR	N	C_N	C_R	$(N1)_{60}$
2.0	6.6	0.091	4	1.99	0.80	***6.9***
3.0	9.8	0.100	6	1.71	0.85	***9.5***
4.0	13.1	0.105	8	1.52	0.89	***11.8***
5.0	16.4	0.107	12	1.39	0.92	***16.7***
6.0	19.7	0.107	10	1.28	0.95	***13.3***
7.0	23.0	0.106	11	1.20	0.98	***14.0***
8.0	26.2	0.103	12	1.13	1.00	***14.8***
					**Mean:**	12.4
					**St. Dev.**	3.3

**Case number:**	43
**Earthquake:**	1978 Miyagiken-Oki
**Magnitude:**	6.5
**Location:**	Hiyori-18
**References:**	Tsuchida et al (1979, 1980) Tohno et al. (1981)<br>Fear et al. (1995)
**Nature of Failure:**	No surface evidence of liquefaction
**Comments:**	Prior to the main shock of M=7.4, an earthquake of magnitude 6.7 occurred.<br>Only the Nakamura site liquefied during this earthquake.<br>The subject zone is seismically very active and had been shaken in 1897 and<br>1936 by earthquakes of Magnitudes 7.3 and 7.7<br>The original Tsuchida (1979) paper (in Japanese) could not be accessed.<br>However the same author's paper of 1980 in English was used to obtain<br>borelogs. Hiyori-18 site was claimed by Tokimatsu (1993) to be by Site C.<br>SPT energy was estimated as 65 % by Seed et al. (84)

**Summary of Data**	Cetin et al. (2016)	Idriss&Boulanger (2010)	Seed et.al. (1984)		Cetin et al. (2016)	Idriss&Boulanger (2010)	Seed et.al. (1984)
Liquefied?	No	No	No	D_{50}	0.150 ± 0.050		0.15
Data Class	B			% Fines	20.0 ± 2.0	20.0	20
Critical Depth Range	8.2 - 13.1	17.1	17.0	% PI			
Depth to GWT (ft)	8.0 ± 0.3	7.9	8.0	D N fines			
σ_v (psf)	1199.5 ± 101.7	2046.8	2040.0	N	9.1 ± 1.5	9.0	9.0
σ_v' (psf)	1033.4 ± 55.8	1482.9	1480.0	C_R	0.86	0.95	
a_{max} (g)	0.140 ± 0.042	0.14	0.140	C_S	1.00	1.00	
r_d	0.98 ± 0.048	0.93	0.960	C_B	1.00	1.00	
CSR	0.103 ± 0.032	0.116	0.120	C_E	1.09	1.09	1.090
Equivalent Magnitude	6.5	6.5		C_N	1.43	1.19	1.2
MSF		1.34	1.14	$(N_1)_{60}$	12.2 ± 2.0	11.1	11.50
CSRN		0.084	0.11				

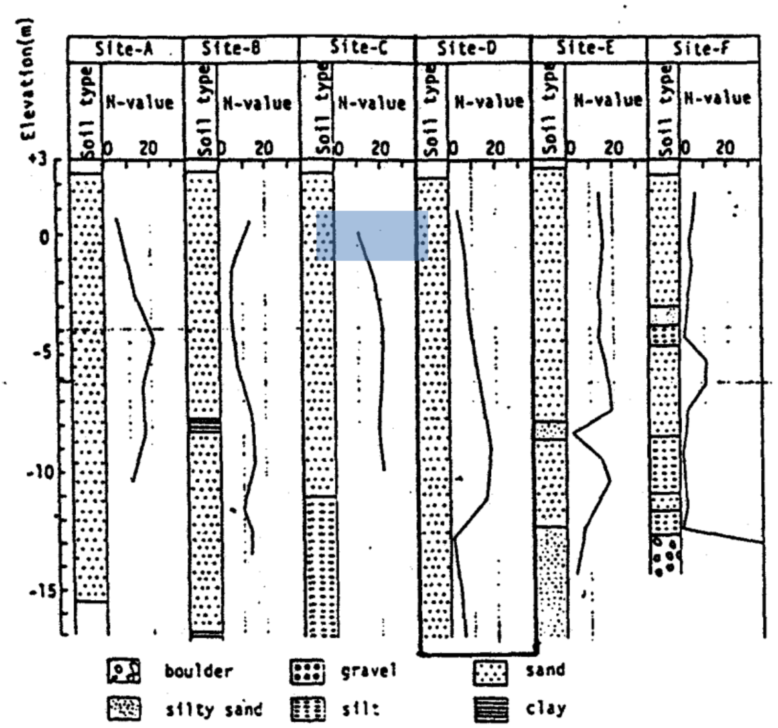

γ_m=110±3 pcf

γ_s=120±3 pcf

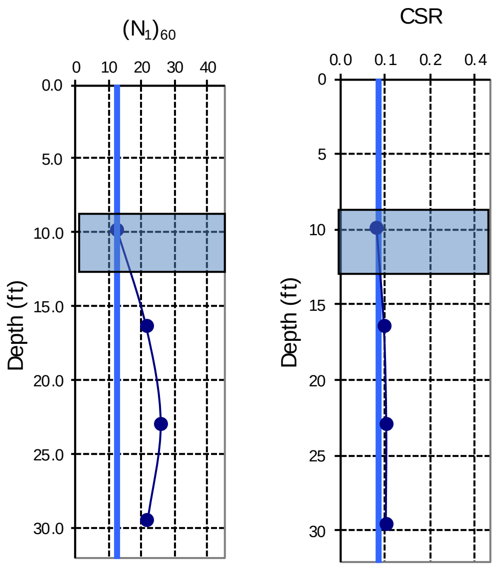

Depth (m)	Depth (ft)	CSR	N	C_N	C_R	$(N1)_{60}$
3.0	9.8	0.099	9.0	1.46	0.85	***12.2***
5.0	16.4	0.120	17.0	1.25	0.92	21.3
7.0	23.0	0.127	22.0	1.10	0.98	25.9
9.0	29.5	0.125	20.0	1.00	1.00	21.8
11.0	36.1	0.120	18.0	0.92	1.00	18.1
					**Mean:**	12.2
					**St. Dev.**	2.0

assigned

**Case number:**	44
**Earthquake:**	1978 Miyagiken-Oki
**Magnitude:**	6.5
**Location:**	Ishinomaki-2
**References:**	Ishihara et al. (1980)<br>Fear et al. (1995)
**Nature of Failure:**	No surface evidence of liquefaction
**Comments:**	Prior to the main shock of M=7.4, an earthquake of magnitude 6.7 occurred.<br>Only the Nakamura site liquefied during this earthquake.<br>The subject zone is seismically very active and had been shaken in 1897 and<br>1936 by the earthquakes of Magnitudes 7.3 and 7.7<br>The site is near the fishery port in the city of Ishinomaki. Loose sand<br>deposit to a depth of 12 m has blow count values on the order of 5. The site<br>has been improved by vibroflotation and compaction piles in 1975.<br>No significant change in SPT values before and after the earthquake.<br>SPT energy was estimated as 65 % by Seed et al. (84)

Summary of Data

	Cetin et al. (2016)	Idriss&Boulanger (2010)	Seed et.al. (1984)		Cetin et al. (2016)	Idriss&Boulanger (2010)	Seed et.al. (1984)
Liquefied?	No	No	No	D_{50}	0.150 ± 0.050		0.15
Data Class	B			% Fines	10.0 ± 2.0	10.0	10
Critical Depth Range	4.6 - 19.7	11.5	13.0	% PI			
Depth to GWT (ft)	4.6 ± 2.0	4.6	5.0				
σ_v (psf)	1410.8 ± 303.6	1378.4	1560.0	N	4.0 ± 0.3	3.7	4.0
σ_v' (psf)	939.9 ± 179.9	939.8	1060.0	C_R	0.88	0.85	
a_{max} (g)	0.120 ± 0.036	0.12	0.120	C_S	1.00	1.00	
r_d	0.92 ± 0.054	0.96	0.970	C_B	1.00	1.00	
CSR	0.107 ± 0.036	0.109	0.110	C_E	1.09	1.09	1.090
Equivalent Magnitude	6.5	6.5		C_N	1.50	1.61	1.3
MSF		1.34	1.10	$(N_1)_{60}$	5.7 ± 0.5	5.5	6.00
CSRN		0.076	0.10				

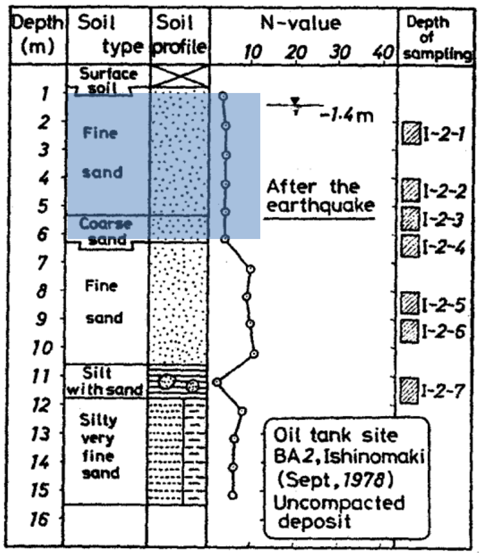

Fig. 14. Standard penetration resistance and depths of Osterberg sampling at the uncompacted site after the earthquake

γ_m=110±3 pcf

γ_s=120±3 pcf

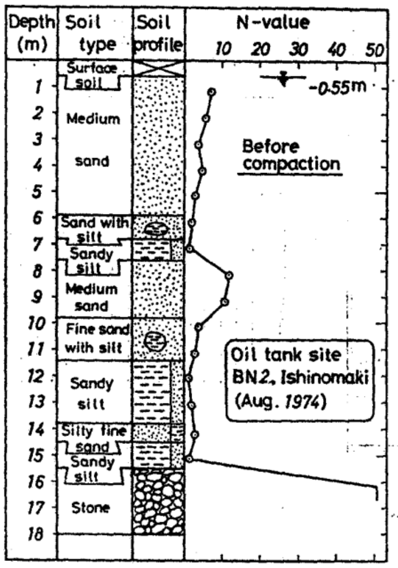

Fig. 11. A soil profile and standard penetration resistance before compaction

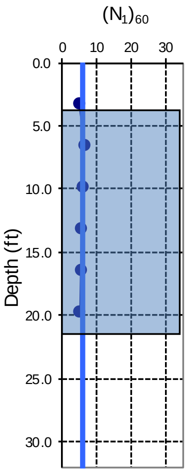

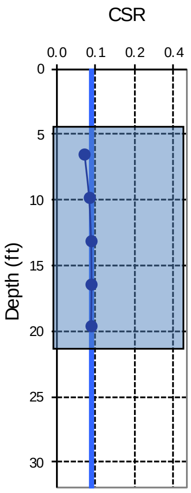

Depth (m)	Depth (ft)	CSR	N	C_N	C_R	$(N1)_{60}$	
1.0	3.3	NA	3.0	2.00	0.73	4.8	too close to surf.
2.0	6.6	0.090	4.0	1.85	0.80	***6.4***	
3.0	9.8	0.103	4.0	1.62	0.85	***6.0***	
4.0	13.1	0.108	4.0	1.46	0.89	***5.6***	
5.0	16.4	0.110	4.0	1.34	0.92	***5.4***	
6.0	19.7	0.108	4.0	1.24	0.95	***5.2***	
					**Mean:**	5.7	
					**St. Dev.**	0.5	

**Case number:**	45
**Earthquake:**	1978 Miyagiken-Oki
**Magnitude:**	6.5
**Location:**	Kitawabuchi 2
**References:**	Iwasaki et al. (1981) Tohno et al. (1981)<br>Fear et al. (1995)
**Nature of Failure:**	No surface evidence of liquefaction
**Comments:**	Prior to the main shock of M=7.4, an earthquake of magnitude 6.7 occurred.<br>Only the Nakamura site liquefied during this earthquake.<br>The subject zone is seismically very active and had been shaken in 1897 and<br>1936 by the earthquakes of Magnitudes 7.3 and 7.7<br>SPT values were taken after main earthquake (M=7.4)<br>SPT energy was estimated as 60 % by Seed et al. (84)

**Summary of Data**	Cetin et al. (2016)	Idriss&Boulanger (2010)	Seed et.al. (1984)		Cetin et al. (2016)	Idriss&Boulanger (2010)	Seed et.al. (1984)
Liquefied?	No	No	No	D_{50}	0.530 ± 0.050		0.53
Data Class	B			% Fines	5.0 ± 2.0	5.0	5
Critical Depth Range	9.8 - 13.1	11.2	11.0	% PI			
Depth to GWT (ft)	9.8 ± 0.3	10.2	10.0				
σ_v (psf)	1181.1 ± 72.4	1294.9	1300.0	N	11.1 ± 1.6	11.0	11.0
σ_v' (psf)	1078.7 ± 45.3	1232.2	1240.0	C_R	0.87	0.85	
a_{max} (g)	0.140 ± 0.042	0.14	0.140	C_S	1.00	1.00	
r_d	0.88 ± 0.052	0.96	0.980	C_B	1.00	1.00	
CSR	0.087 ± 0.027	0.092	0.095	C_E	1.00	1.00	1.000
Equivalent Magnitude	6.5	6.5		C_N	1.40	1.32	1.2
MSF		1.34	1.19	$(N_1)_{60}$	13.5 ± 2.0	12.3	13.50
CSRN		0.065	0.08				

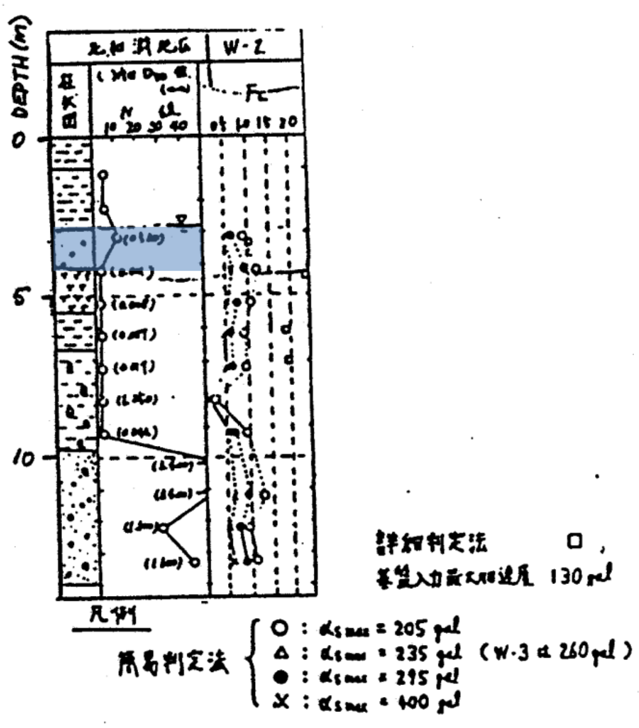

γ_m=100±3 pcf

γ_s=120±3 pcf

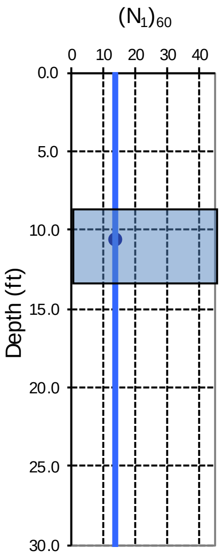

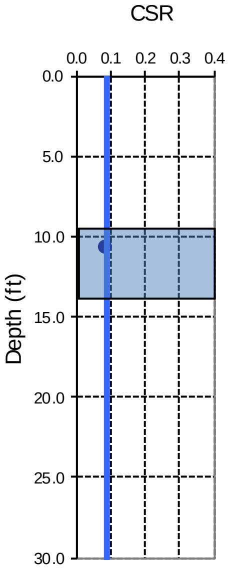

Depth (m)	Depth (ft)	CSR	N	C_N	C_R	$(N1)_{60}$
1.0	3.3	NA	4.0	2.0	0.73	5.9
2.0	6.6	NA	4.0	1.8	0.80	5.7
3.3	10.7	0.085	***11.0***	***1.4***	***0.9***	***13.5***
4.3	13.9	0.093	3.0	1.3	0.90	3.5
5.3	17.2	0.095	2.0	1.2	0.93	2.3
					**Mean:**	13.5
					**St. Dev.**	2.0

assigned

**Case number:**	46
**Earthquake:**	1978 Miyagiken-Oki
**Magnitude:**	6.5
**Location:**	Nakajima-18
**References:**	Tsuchida et al (1979, 1980) Tohno et al. (1981)<br>Fear et al. (1995)
**Nature of Failure:**	No surface evidence of liquefaction
**Comments:**	Prior to the main shock of M=7.4, an earthquake of magnitude 6.7 occurred.<br>Only the Nakamura site liquefied during this earthquake.<br>The subject zone is seismically very active and had been shaken in 1897 and<br>1936 by the earthquakes of Magnitudes 7.3 and 7.7<br>The original Tsuchida (1979) paper (in Japanese) could not be accessed.<br>However the same author's paper of 1980 in English was used to obtain<br>borelogs. Nakajima 18 site was claimed by Tokimatsu (1993) to be by Site A.<br>SPT energy was estimated as 65 % by Seed et al. (84)

Summary of Data

	Cetin et al. (2016)	Idriss&Boulanger (2010)	Seed et.al. (1984)		Cetin et al. (2016)	Idriss&Boulanger (2010)	Seed et.al. (1984)
Liquefied?	No	No	No	D_{50}	0.350 ± 0.050		0.35
Data Class	B			% Fines	3.0 ± 2.0	3.0	3
Critical Depth Range	8.0 - 20.0	20.0	20.0	% PI			
Depth to GWT (ft)	8.0 ± 0.3	7.9	8.0				
σ_v (psf)	1630.0 ± 251.8	2401.8	2400.0	N	9.6 ± 3.9	12.0	12.0
σ_v' (psf)	1255.6 ± 129.5	1649.9	1650.0	C_R	0.90	0.95	
a_{max} (g)	0.140 ± 0.042	0.14	0.140	C_S	1.00	1.00	
r_d	0.97 ± 0.061	0.91	0.960	C_B	1.00	1.00	
CSR	0.114 ± 0.036	0.120	0.125	C_E	1.09	1.09	1.090
Equivalent Magnitude	6.5	6.5		C_N	1.30	1.13	1.1
MSF		1.34	1.14	$(N_1)_{60}$	12.2 ± 5.0	14.1	14.50
CSRN		0.088	0.11				

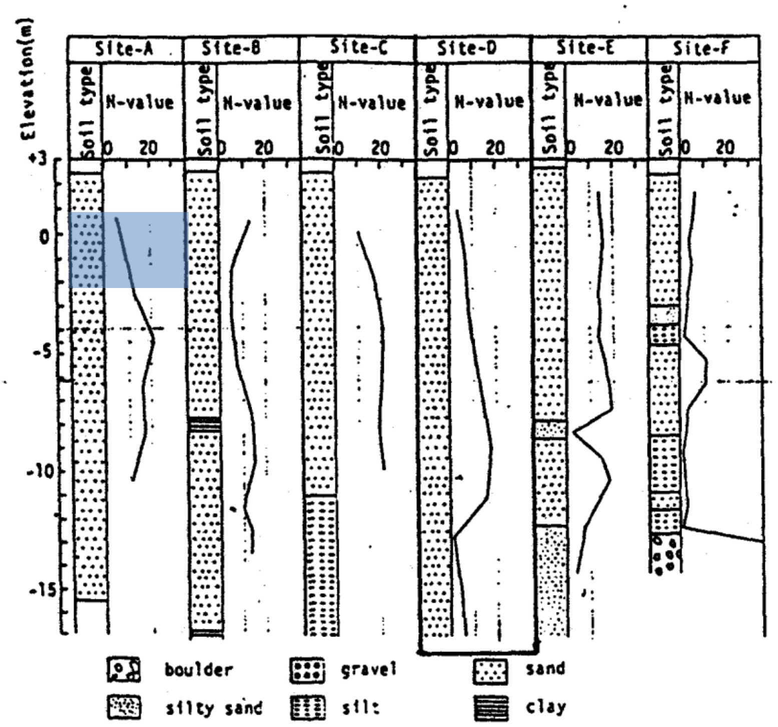

γ_m=110±3 pcf

γ_s=125±3 pcf

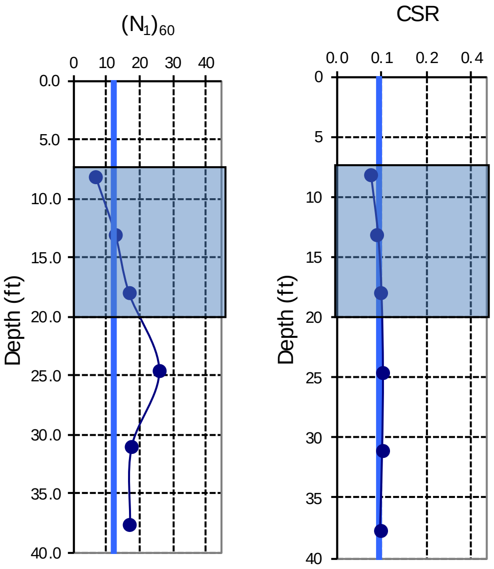

Depth (m)	Depth (ft)	CSR	N	C_N	C_R	$(N1)_{60}$
2.5	8.2	0.091	5.0	1.54	0.82	***6.9***
4.0	13.1	0.112	10.0	1.33	0.89	***12.9***
5.5	18.0	0.122	14.0	1.18	0.94	***16.9***
7.5	24.6	0.126	23.0	1.05	0.99	26.1
9.5	31.2	0.124	17.0	0.95	1.00	17.7
11.5	37.7	0.117	18.0	0.88	1.00	17.3
					**Mean:**	12.2
					**St. Dev.**	5.0

**Case number:**	47
**Earthquake:**	1978 Miyagiken-Oki
**Magnitude:**	6.5
**Location:**	Nakamura-4
**References:**	Iwasaki et al. (1978) Tohno et al. (1981)<br>Fear et al. (1995)
**Nature of Failure:**	Sand boils, cracking
**Comments:**	The site is located in the inside base towards the north end of an embankment along the left bank of the Natori River.<br>Prior to the main shock of M=7.4, an earthquake of magnitude 6.7 occurred.<br>Only the Nakamura site liquefied during this earthquake.<br>The subject zone is seismically very active and had been shaken in 1897 and 1936 by the earthquakes of Magnitudes 7.3 and 7.7<br>SPT values were taken after main earthquake (M=7.4)<br>SPT energy was estimated as 60 % by Seed et al. (84)

Summary of Data

	Cetin et al. (2016)	Idriss&Boulanger (2010)	Seed et.al. (1984)		Cetin et al. (2016)	Idriss&Boulanger (2010)	Seed et.al. (1984)
Liquefied?	Yes	Yes	Yes	D_{50}	0.700 ± 0.050		0.7
Data Class	B			% Fines	5.0 ± 2.0	5.0	5
Critical Depth Range	9.8 - 16.4	9.2	11.0	% PI			
Depth to GWT (ft)	1.6 ± 0.3	1.6	2.0				
σ_v (psf)	1558.4 ± 135.8	1106.9	1320.0	N	5.6 ± 0.4	4.7	5.0
σ_v' (psf)	841.9 ± 73.7	626.6	760.0	C_R	0.89	0.85	
a_{max} (g)	0.120 ± 0.036	0.12	0.120	C_S	1.00	1.00	
r_d	0.98 ± 0.058	0.97	0.980	C_B	1.00	1.00	
CSR	0.142 ± 0.044	0.128	0.135	C_E	1.00	1.00	1.000
Equivalent Magnitude	6.5	6.5		C_N	1.59	1.70	1.6
MSF		1.34	1.17	$(N_1)_{60}$	7.9 ± 0.6	6.9	8.00
CSRN		0.087	0.12				

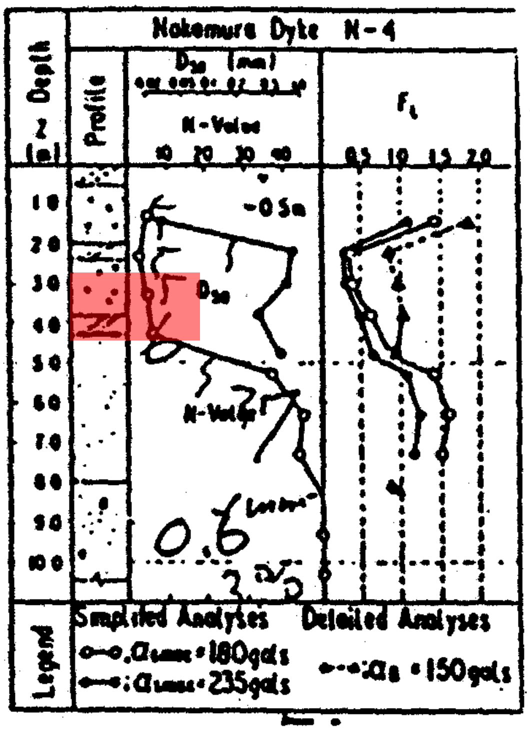

γ_m=110±3 pcf

γ_s=120±3 pcf

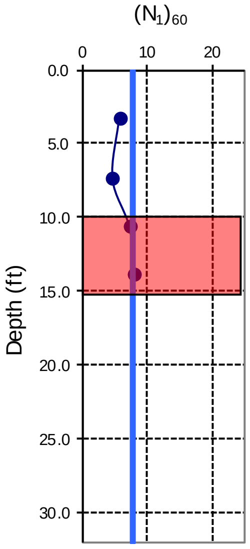

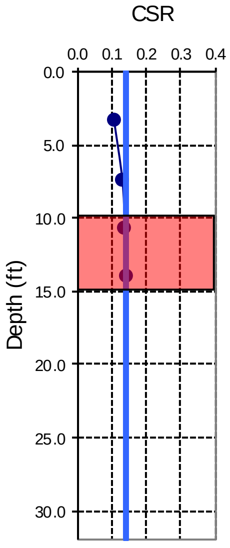

Depth (m)	Depth (ft)	CSR	N	C_N	C_R	$(N1)_{60}$	
1.0	3.3	0.107	4	2.00	0.73	5.9	close to surf.
2.3	7.4	0.132	3	2.00	0.81	4.9	
3.3	10.7	0.139	5	1.74	0.86	***7.5***	
4.3	13.9	0.142	6	1.54	0.90	***8.3***	
					**Mean:**	7.9	
					**St. Dev.**	0.6	

**Case number:**	48
**Earthquake:**	1978 Miyagiken-Oki
**Magnitude:**	6.5
**Location:**	Nakamura-5
**References:**	Iwasaki et al. (1978) Tohno et al. (1981)<br>Fear et al. (1995)
**Nature of Failure:**	No surface evidence of liquefaction
**Comments:**	The site is located in the outside base at the north end of an embankment along the left bank of the Natori River.<br>Prior to the main shock of M=7.4, an earthquake of magnitude 6.7 occurred.<br>Only the Nakamura site liquefied during this earthquake.<br>The subject zone is seismically very active and had been shaken in 1897 and 1936 by the earthquakes of Magnitudes 7.3 and 7.7<br>SPT values were taken after main earthquake (M=7.4)<br>SPT energy was estimated as 60 % by Seed et al. (84)

Summary of Data

	Cetin et al. (2016)	Idriss&Boulanger (2010)	Seed et.al. (1984)		Cetin et al. (2016)	Idriss&Boulanger (2010)	Seed et.al. (1984)
Liquefied?	No	No	No	D_{50}	0.280 ± 0.050		0.28
Data Class	B			% Fines	4.0 ± 2.0	4.0	4
Critical Depth Range	9.0 - 13.1	11.2	11.0	% PI			
Depth to GWT (ft)	4.3 ± 0.3	4.3	4.0				
σ_v (psf)	1284.8 ± 86.0	1315.8	1320.0	N	7.1 ± 1.5	7.0	7.0
σ_v' (psf)	860.6 ± 48.9	877.2	880.0	C_R	0.86	0.85	
a_{max} (g)	0.120 ± 0.036	0.12	0.120	C_S	1.00	1.00	
r_d	0.97 ± 0.050	0.96	0.980	C_B	1.00	1.00	
CSR	0.113 ± 0.035	0.112	0.115	C_E	1.00	1.00	1.000
Equivalent Magnitude	6.5	6.5		C_N	1.57	1.61	1.5
MSF		1.34	1.15	$(N_1)_{60}$	9.6 ± 2.0	9.6	10.00
CSRN		0.078	0.10				

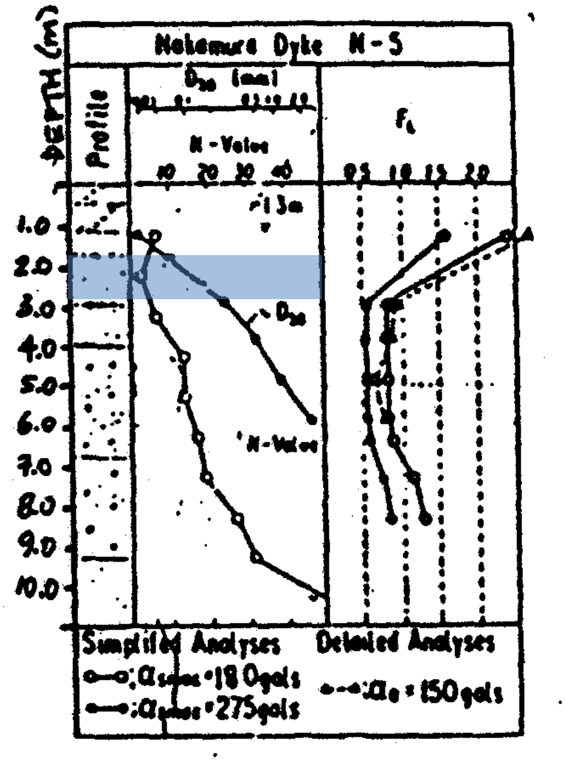

γ_m=110±3 pcf

γ_s=120±3 pcf

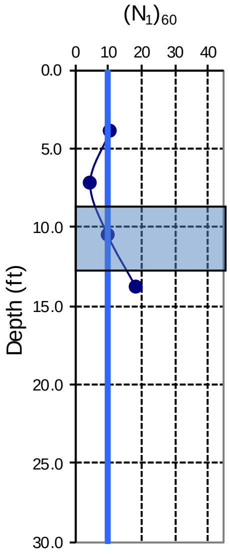

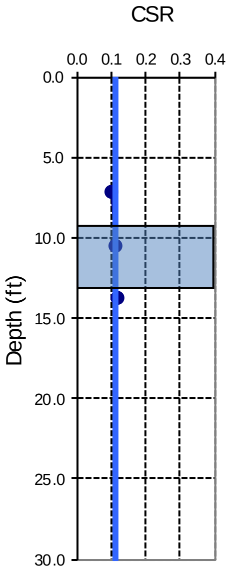

Depth (m)	Depth (ft)	CSR	N	C_N	C_R	$(N1)_{60}$
1.2	3.9	NA	7	2.00	0.75	10.5
2.2	7.2	0.099	3	1.82	0.81	4.4
3.2	10.5	0.111	7	1.60	0.86	***9.6***
4.2	13.8	0.118	14	1.44	0.89	18.1
					**Mean:**	9.6
					**St. Dev.**	2.0

assigned

**Case number:**	49
**Earthquake:**	1978 Miyagiken-Oki
**Magnitude:**	6.5
**Location:**	Oiiri 1
**References:**	Iwasaki et al. (1978) Tohno et al. (1981)<br>Fear et al. (1995)<br>A.S. Cakmak (1986) "Soil Liquefaction Studies in Japan", Vol. 5 No.1 Jan 1986,<br>Soil Dynamics & Earthquake Engineering.
**Nature of Failure:**	No surface evidence of liquefaction
**Comments:**	Prior to the main shock of M=7.4, an earthquake of magnitude 6.7 occurred.<br>Only the Nakamura site liquefied during this earthquake.<br>The subject zone is seismically very active and had been shaken in 1897 and<br>1936 by earthquakes of Magnitudes 7.3 and 7.7<br>Iwasaki et al (1978) paper did not mention Oiiri sites. However Cakmak et al<br>gave a representative depth vs. N profile for Oiiri 1<br>SPT values were taken after main earthquake (M=7.4)<br>SPT energy is estimated as 60 % by Seed et al. (84)

**Summary of Data**	Cetin et al. (2016)	Idriss&Boulanger (2010)	Seed et.al. (1984)		Cetin et al. (2016)	Idriss&Boulanger (2010)	Seed et.al. (1984)
Liquefied?	No	No	No	D_{50}	0.340 ± 0.050		0.34
Data Class	B			% Fines	5.0 ± 2.0	5.0	5
Critical Depth Range	14.0 - 25.0	21.0	21.0	% PI			
Depth to GWT (ft)	14.0 ± 0.3	14.1	14.0				
σ_v (psf)	2200.0 ± 224.6	2213.9	2220.0	N	9.2 ± 2.0	9.0	9.0
σ_v' (psf)	1856.8 ± 115.9	1775.3	1780.0	C_R	0.95	0.95	
a_{max} (g)	0.140 ± 0.042	0.14	0.140	C_S	1.00	1.00	
r_d	0.83 ± 0.081	0.90	0.950	C_B	1.00	1.00	
CSR	0.090 ± 0.029	0.102	0.110	C_E	1.00	1.00	1.000
Equivalent Magnitude	6.5	6.5		C_N	1.07	1.10	1.1
MSF		1.34	1.16	$(N_1)_{60}$	9.3 ± 2.0	9.4	9.50
CSRN		0.075	0.10				

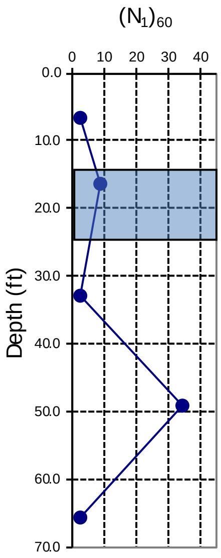

γ_m=110±3 pcf

γ_s=120±3 pcf

By Cakmak(1986)

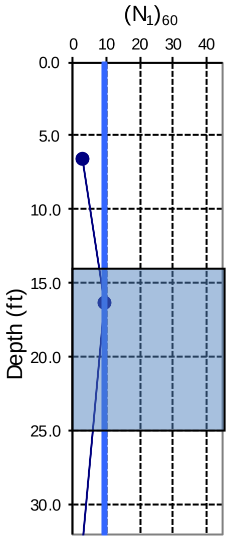

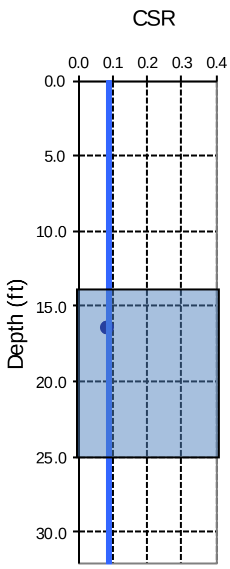

Depth (m)	Depth (ft)	CSR	N	C_N	C_R	$(N1)_{60}$
2.0	6.6	NA	2	1.71	0.80	2.7
5.0	16.4	0.086	9	1.12	0.92	***9.3***
10.0	32.8	0.086	3	0.90	1.00	2.7
15.0	49.2	0.078	45	0.77	1.00	34.7
20.0	65.6	0.079	4	0.68	1.00	2.7
					**Mean:**	9.3
					**St. Dev.**	2.0

assigned

**Case number:**	50
**Earthquake:**	1978 Miyagiken-Oki
**Magnitude:**	6.5
**Location:**	Shiomi 6
**References:**	Tsuchida et al (1979, 1980) Tohno et al. (1981)<br>Fear et al. (1995) Tokimatsu (1983)
**Nature of Failure:**	No surface evidence of liquefaction
**Comments:**	Prior to the main shock of M=7.4, an earthquake of magnitude 6.7 occurred.<br>Only Nakamura site liquefied during this earthquake.<br>The subject zone is seismically very active and had been shaken in 1897 and<br>1936 by the earthquakes of Magnitudes 7.3 and 7.7<br>The original Tsuchida (1979) paper (in Japanese) could not be accessed.<br>However the same author's paper of 1980 in English was used to obtain<br>borelogs. Shiomi 6 site was identified as Site D by Tokimatsu (1983).<br>SPT energy was estimated as 65 % by Seed et al. (84)

Summary of Data

	Cetin et al. (2016)	Idriss&Boulanger (2010)	Seed et.al. (1984)		Cetin et al. (2016)	Idriss&Boulanger (2010)	Seed et.al. (1984)
Liquefied?	No	No	No	D_{50}	0.250 ± 0.050		0.25
Data Class	B			% Fines	10.0 ± 2.0	10.0	10
Critical Depth Range	9.8 - 19.7	13.1	13.0	% PI			
Depth to GWT (ft)	8.0 ± 0.3	7.9	8.0				
σ_v (psf)	1691.7 ± 199.4	1566.4	1560.0	N	7.3 ± 1.7	6.0	6.0
σ_v' (psf)	1269.6 ± 100.8	1253.1	1250.0	C_R	0.90	0.85	
a_{max} (g)	0.140 ± 0.042	0.14	0.140	C_S	1.00	1.00	
r_d	0.94 ± 0.064	0.95	0.970	C_B	1.00	1.00	
CSR	0.114 ± 0.035	0.108	0.110	C_E	1.09	1.09	1.090
Equivalent Magnitude	6.5	6.5		C_N	1.29	1.34	1.2
MSF		1.34	1.16	$(N_1)_{60}$	9.3 ± 2.2	7.5	8.00
CSRN		0.077	0.10				

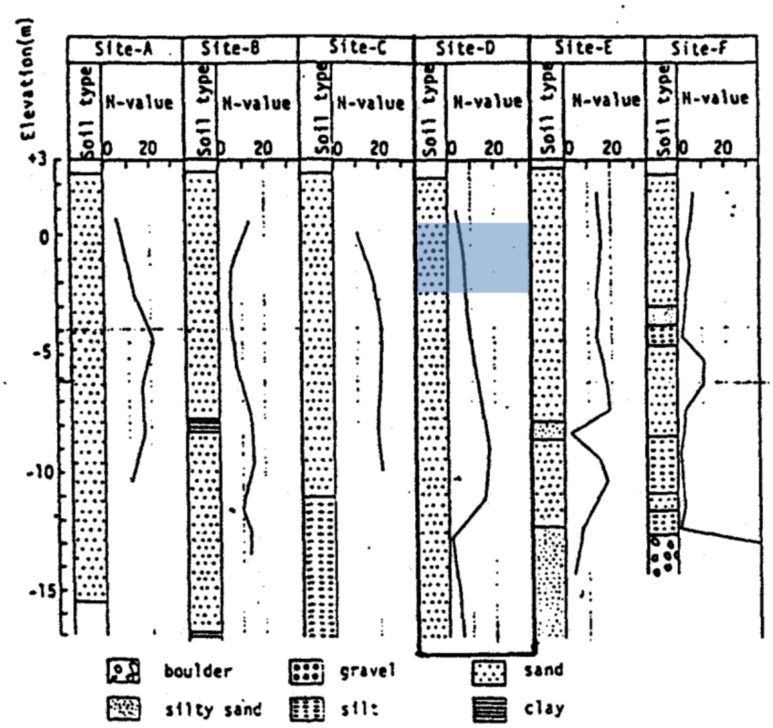

γ_m=110±3 pcf

γ_s=120±3 pcf

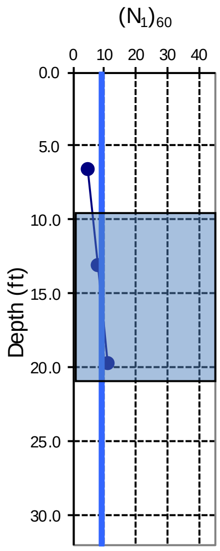

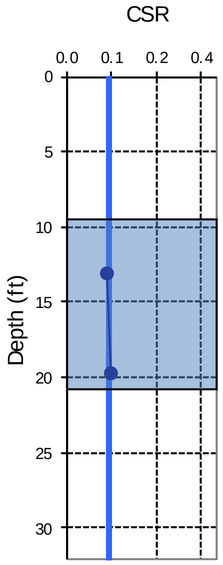

Depth (m)	Depth (ft)	CSR	N	C_N	C_R	$(N1)_{60}$
2.0	6.6	NA	3.0	1.71	0.80	4.5
4.0	13.1	0.110	6.0	1.34	0.89	***7.8***
6.0	19.7	0.121	9.0	1.17	0.95	***10.9***
					**Mean:**	9.3
					**St. Dev.**	2.2

**Case number:**	51
**Earthquake:**	1978 Miyagiken-Oki
**Magnitude:**	6.5
**Location:**	Yuriage Bridge 1
**References:**	Iwasaki et al. (1978) Tohno et al. (1981)<br>Fear et al. (1995)
**Nature of Failure:**	No surface evidence of liquefaction
**Comments:**	Prior to the main shock of M=7.4, an earthquake of magnitude 6.7 occurred.<br>Only the Nakamura site liquefied during this earthquake.<br>The subject zone is seismically very active and had been shaken in 1897 and<br>1936 by the earthquakes of Magnitudes 7.3 and 7.7<br>SPT values were taken after main earthquake (M=7.4)<br>SPT energy was estimated as 60 % by Seed et al. (84)

**Summary of Data**	Cetin et al. (2016)	Idriss&Boulanger (2010)	Seed et.al. (1984)		Cetin et al. (2016)	Idriss&Boulanger (2010)	Seed et.al. (1984)
Liquefied?	No	No	No	D_{50}	0.400 ± 0.050		0.4
Data Class	B			% Fines	10.0 ± 2.0	10.0	10
Critical Depth Range	9.8 - 13.1	14.1	14.0	% PI			
Depth to GWT (ft)	5.6 ± 0.3	5.9	6.0				
σ_v (psf)	1233.1 ± 67.9	1670.8	1680.0	N	3.0 ± 1.3	4.0	4.0
σ_v' (psf)	866.6 ± 40.4	1169.6	1180.0	C_R	0.87	0.95	
a_{max} (g)	0.120 ± 0.036	0.12	0.120	C_S	1.00	1.00	
r_d	0.98 ± 0.051	0.94	0.970	C_B	1.00	1.00	
CSR	0.109 ± 0.033	0.105	0.110	C_E	1.00	1.00	1.000
Equivalent Magnitude	6.5	6.5		C_N	1.56	1.41	1.3
MSF		1.34	1.16	$(N_1)_{60}$	4.0 ± 1.8	5.4	5.00
CSRN		0.075	0.10				

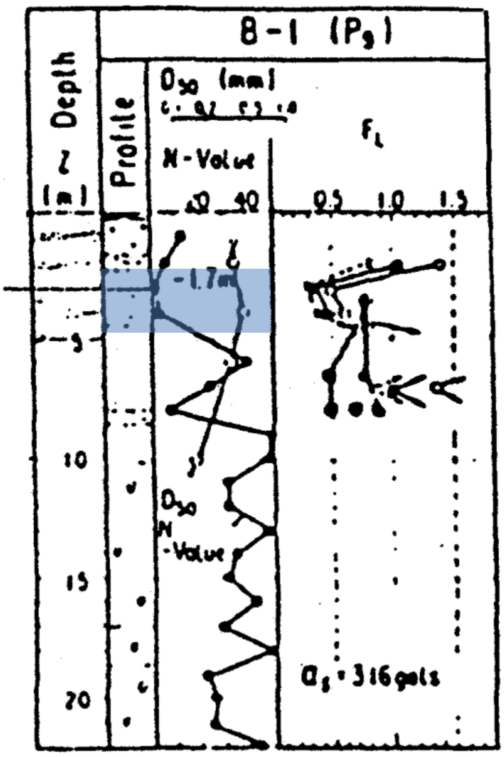

γ_m=100±3 pcf

γ_s=115±3 pcf

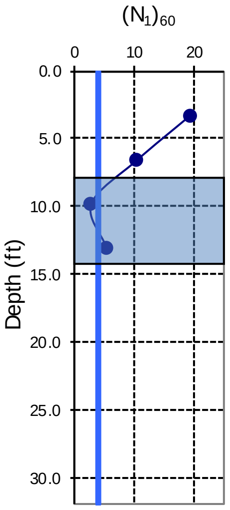

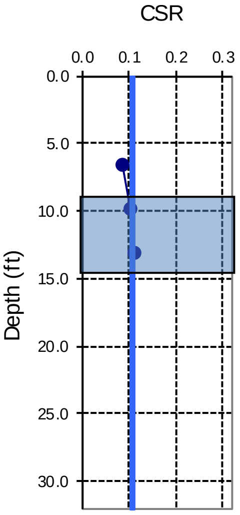

Depth (m)	Depth (ft)	CSR	N	C_N	C_R	$(N1)_{60}$	
1.0	3.3	NA	13	2.00	0.73	19.1	close to surf.
2.0	6.6	0.085	7	1.86	0.80	10.4	fines transition
3.0	9.8	0.103	2	1.64	0.85	***2.8***	
4.0	13.1	0.114	4	1.49	0.89	***5.3***	
					**Mean:**	4.0	
					**St. Dev.**	1.8	

**Case number:**	52
**Earthquake:**	1978 Miyagiken-Oki
**Magnitude:**	6.5
**Location:**	Yuriage Bridge 2
**References:**	Iwasaki et al. (1978) Tohno et al. (1981)<br>Fear et al. (1995)
**Nature of Failure:**	No surface evidence of liquefaction
**Comments:**	Prior to the main shock of M=7.4, an earthquake of magnitude 6.7 occurred.<br>Only the Nakamura site liquefied during this earthquake.<br>The subject zone is seismically very active and had been shaken in 1897 and<br>1936 by earthquakes of Magnitudes 7.3 and 7.7<br>Gravel percent exceeds 20 % by weight. (Seed et al, 84)<br>SPT values were taken after main earthquake (M=7.4)<br>SPT energy was estimated as 67 % by Seed et al. (84)

Summary of Data

	Cetin et al. (2016)	Idriss&Boulanger (2010)	Seed et.al. (1984)		Cetin et al. (2016)	Idriss&Boulanger (2010)	Seed et.al. (1984)
Liquefied?	No	No	No	D_{50}	1.600 ± 0.050		1.6
Data Class	B			% Fines	7.0 ± 2.0	7.0	7
Critical Depth Range	6.0 - 10.0	8.2	11.0	% PI			
Depth to GWT (ft)	4.3 ± 0.3	3.9	4.0				
σ_v (psf)	893.4 ± 85.4	960.7	1320.0	N	11.4 ± 1.3	10.1	13.0
σ_v' (psf)	660.3 ± 46.4	710.1	880.0	C_R	0.82	0.85	
a_{max} (g)	0.120 ± 0.036	0.12	0.120	C_S	1.00	1.00	
r_d	1.00 ± 0.038	0.96	0.980	C_B	1.00	1.00	
CSR	0.105 ± 0.032	0.112	0.115	C_E	1.12	1.12	1.120
Equivalent Magnitude	6.5	6.5		C_N	1.79	1.68	1.5
MSF		1.34	1.15	$(N_1)_{60}$	18.7 ± 2.2	16.2	21.50
CSRN		0.076	0.10				

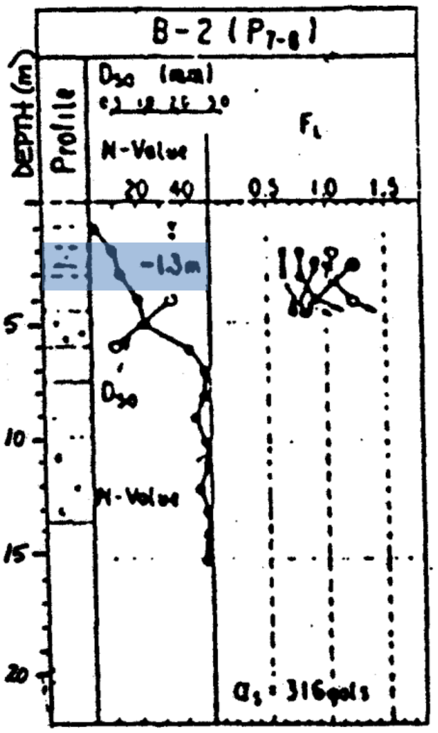

γ_m=100±3 pcf

γ_s=125±3 pcf

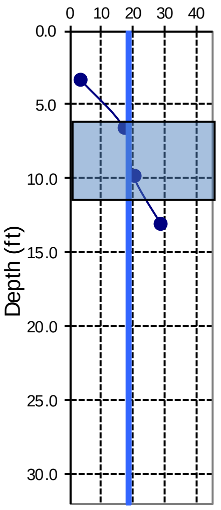

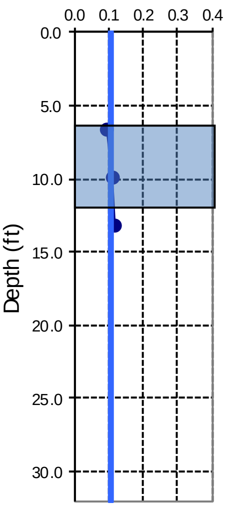

Depth (m)	Depth (ft)	CSR	N	C_N	C_R	$(N1)_{60}$	
1.0	3.3	NA	2	2.00	0.73	3.3	close to surf.
2.0	6.6	0.097	10	1.93	0.80	***17.2***	
3.0	9.8	0.112	13	1.65	0.85	***20.3***	
4.0	13.1	0.121	20	1.47	0.89	29.1	
					**Mean:**	18.7	
					**St. Dev.**	2.2	

**Case number:**	53
**Earthquake:**	1978 Miyagiken-Oki
**Magnitude:**	6.5
**Location:**	Yuriage Bridge 3
**References:**	Iwasaki et al. (1978) Tohno et al. (1981)<br>Fear et al. (1995)
**Nature of Failure:**	No surface evidence of liquefaction
**Comments:**	Prior to the main shock of M=7.4, an earthquake of magnitude 6.7 occurred.<br>Only the Nakamura site liquefied during this earthquake.<br>The subject zone is seismically very active and had been shaken in 1897 and<br>1936 by the earthquakes of Magnitudes 7.3 and 7.7<br>SPT values were taken after main earthquake (M=7.4)<br>SPT energy was estimated as 60 % by Seed et al. (84)

**Summary of Data**	Cetin et al. (2016)	Idriss&Boulanger (2010)	Seed et.al. (1984)		Cetin et al. (2016)	Idriss&Boulanger (2010)	Seed et.al. (1984)
Liquefied?	No	No	No	D_{50}	1.200 ± 0.050		1.2
Data Class	B			% Fines	12.0 ± 2.0	12.0	12
Critical Depth Range	6.6 - 13.1	14.1	14.0	% PI			
Depth to GWT (ft)	0.9 ± 0.3	1.0	1.0				
σ_v (psf)	1172.6 ± 134.0	1670.8	1680.0	N	7.1 ± 1.0	8.0	8.0
σ_v' (psf)	611.6 ± 70.3	877.2	870.0	C_R	0.85	0.95	
a_{max} (g)	0.120 ± 0.036	0.12	0.120	C_S	1.00	1.00	
r_d	0.98 ± 0.045	0.94	0.970	C_B	1.00	1.00	
CSR	0.147 ± 0.045	0.142	0.145	C_E	1.00	1.00	1.000
Equivalent Magnitude	6.5	6.5		C_N	1.86	1.56	1.5
MSF		1.34	1.12	$(N_1)_{60}$	11.2 ± 1.5	11.8	12.00
CSRN		0.097	0.13				

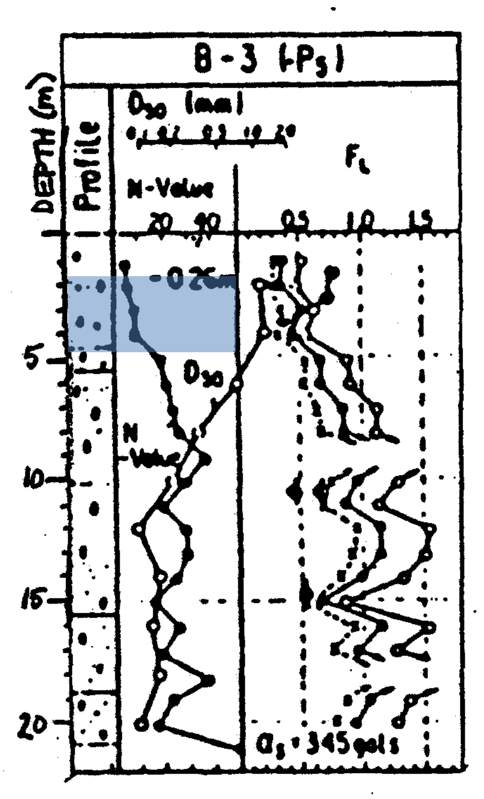

γ_m=110±3 pcf

γ_s=120±3 pcf

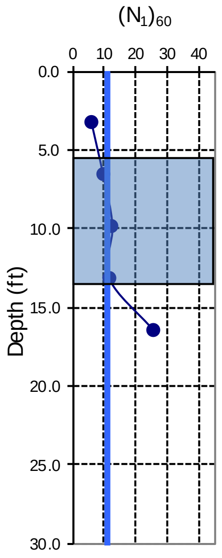

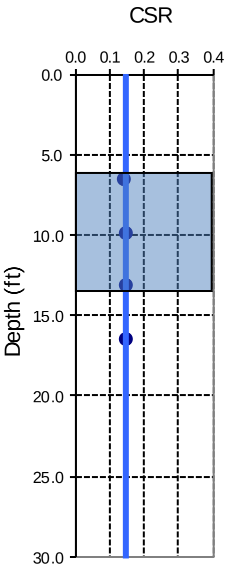

Depth (m)	Depth (ft)	CSR	N	C_N	C_R	$(N1)_{60}$	
1.0	3.3	0.128	4	2.00	0.73	5.9	close to surf.
2.0	6.6	0.142	6	2.00	0.80	***9.6***	
3.0	9.8	0.147	8	1.86	0.85	***12.6***	
4.0	13.1	0.148	8	1.63	0.89	***11.5***	
5.0	16.4	0.148	19	1.46	0.92	25.6	
					**Mean:**	11.2	
					**St. Dev.**	1.5	

**Case number:**	54
**Earthquake:**	1978 Miyagiken-Oki
**Magnitude:**	6.5
**Location:**	Yuriagekami-1
**References:**	Iwasaki et al. (1978) Tohno et al. (1981)<br>Fear et al. (1995)
**Nature of Failure:**	No surface evidence of liquefaction
**Comments:**	Prior to the main shock of M=7.4, an earthquake of magnitude 6.7 occurred.<br>Only the Nakamura site liquefied during this earthquake.<br>The subject zone is seismically very active and had been shaken in 1897 and<br>1936 by the earthquakes of Magnitudes 7.3 and 7.7<br>SPT values were taken after main earthquake (M=7.4)<br>SPT energy was estimated as 60 % by Seed et al. (84)

**Summary of Data**	Cetin et al. (2016)	Idriss&Boulanger (2010)	Seed et.al. (1984)		Cetin et al. (2016)	Idriss&Boulanger (2010)	Seed et.al. (1984)
Liquefied?	No	No	No	D_{50}	0.040 ± 0.050		0.04
Data Class	B			% Fines	60.0 ± 2.0	60.0	60
Critical Depth Range	5.9 - 18.0	18.0	18.0	% PI			
Depth to GWT (ft)	6.0 ± 0.3	5.9	6.0				
σ_v (psf)	1257.5 ± 224.0	2067.7	2060.0	N	2.0 ± 0.9	2.0	2.0
σ_v' (psf)	882.9 ± 100.8	1315.8	1310.0	C_R	0.87	0.95	
a_{max} (g)	0.120 ± 0.036	0.12	0.120	C_S	1.00	1.00	
r_d	0.97 ± 0.053	0.92	0.960	C_B	1.00	1.00	
CSR	0.108 ± 0.034	0.113	0.120	C_E	1.00	1.00	1.000
Equivalent Magnitude	6.5	6.5		C_N	1.55	1.31	1.2
MSF		1.34	1.14	$(N_1)_{60}$	2.7 ± 1.2	2.5	2.50
CSRN		0.081	0.11				

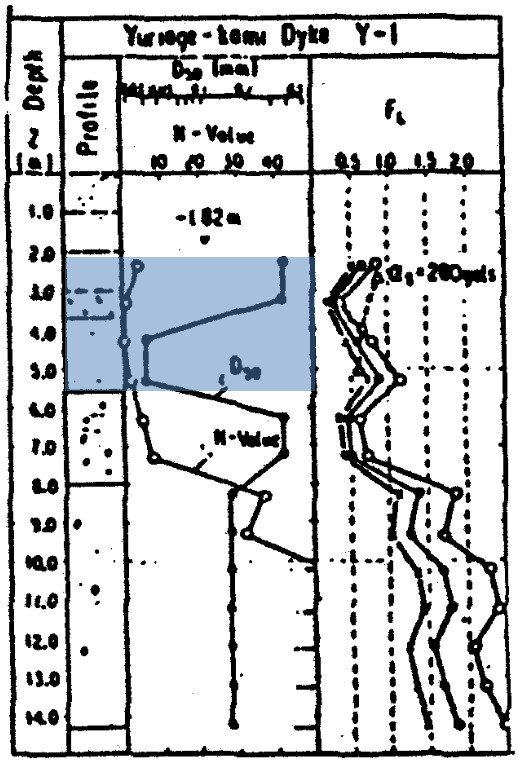

γ_m=100±3 pcf

γ_s=110±3 pcf

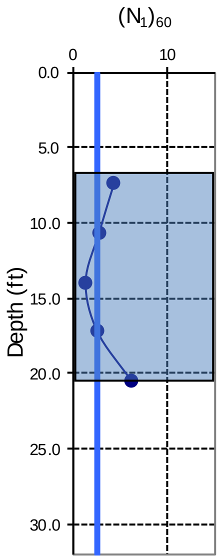

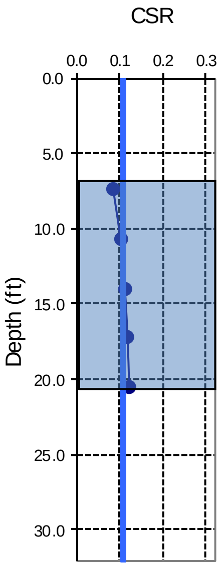

Depth (m)	Depth (ft)	CSR	N	C_N	C_R	$(N1)_{60}$
2.3	7.4	0.087	3	1.78	0.81	***4.3***
3.3	10.7	0.104	2	1.61	0.86	***2.8***
4.3	13.9	0.114	1	1.47	0.90	***1.3***
5.3	17.2	0.120	2	1.37	0.93	***2.5***
6.3	20.5	0.124	5	1.28	0.96	6.1
					**Mean:**	2.7
					**St. Dev.**	1.2

**Case number:**	55
**Earthquake:**	1978 Miyagiken-Oki
**Magnitude:**	6.5
**Location:**	Yuriagekami-2
**References:**	Iwasaki et al. (1978) Tohno et al. (1981)<br>Fear et al. (1995)
**Nature of Failure:**	No surface evidence of liquefaction
**Comments:**	Prior to the main shock of M=7.4, an earthquake of magnitude 6.7 occurred.<br>Only the Nakamura site liquefied during this earthquake.<br>The subject zone is seismically very active and had been shaken in 1897 and<br>1936 by the earthquakes of Magnitudes 7.3 and 7.7<br>SPT values were taken after main earthquake (M=7.4)<br>SPT energy was estimated as 60 % by Seed et al. (84)

Summary of Data

	Cetin et al. (2016)	Idriss&Boulanger (2010)	Seed et.al. (1984)		Cetin et al. (2016)	Idriss&Boulanger (2010)	Seed et.al. (1984)
Liquefied?	No	No	No	D_{50}	0.400 ± 0.050		0.4
Data Class	B			% Fines	0.0 ± 2.0	0.0	0
Critical Depth Range	6.6 - 18.0	14.1	14.0	% PI			
Depth to GWT (ft)	2.8 ± 0.3	3.0	3.0				
σ_v (psf)	1420.6 ± 231.7	1670.8	1680.0	N	8.8 ± 3.3	11.0	11.0
σ_v' (psf)	826.9 ± 114.9	981.6	990.0	C_R	0.88	0.95	
a_{max} (g)	0.120 ± 0.036	0.12	0.120	C_S	1.00	1.00	
r_d	0.96 ± 0.055	0.94	0.970	C_B	1.00	1.00	
CSR	0.129 ± 0.040	0.125	0.130	C_E	1.00	1.00	1.000
Equivalent Magnitude	6.5	6.5		C_N	1.60	1.45	1.4
MSF		1.34	1.13	$(N_1)_{60}$	12.3 ± 4.7	15.1	15.00
CSRN		0.086	0.12				

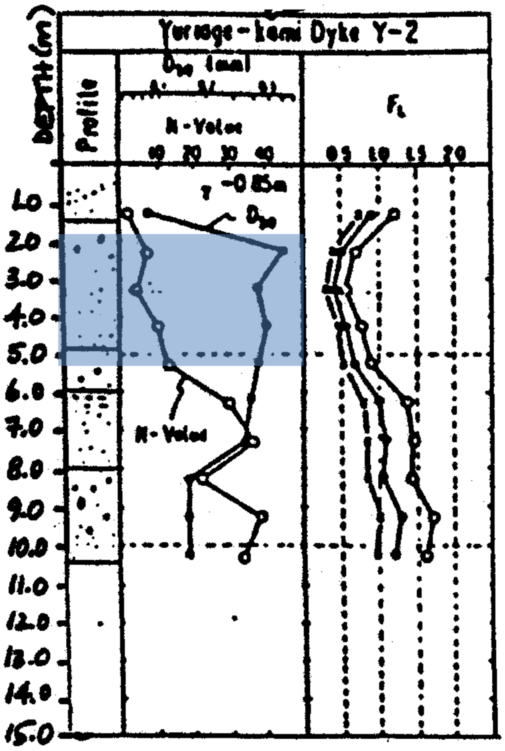

γ_m=100±3 pcf

γ_s=120±3 pcf

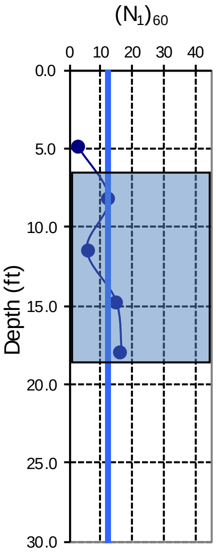

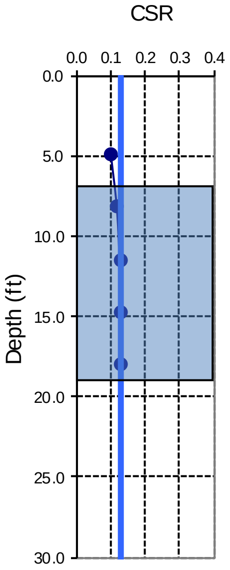

Depth (m)	Depth (ft)	CSR	N	C_N	C_R	$(N1)_{60}$	
1.5	4.9	0.103	2	2.00	0.77	3.1	close to surf.
2.5	8.2	0.120	8	1.89	0.82	***12.5***	
3.5	11.5	0.128	4	1.65	0.87	***5.7***	
4.5	14.8	0.131	11	1.48	0.90	***14.7***	
5.5	18.0	0.132	13	1.35	0.94	***16.5***	
					**Mean:**	12.3	
					**St. Dev.**	4.7	

**Case number:**	56
**Earthquake:**	1978 Miyagiken-Oki
**Magnitude:**	7.7 (Mw) USGS Centennial Earthquake Catalog
**Location:**	Nakajima-18
**References:**	Tsuchida et al (1979, 1980) Tohno et al. (1981)<br>Fear et al. (1995)
**Nature of Failure:**	Liquefied
**Comments:**	Prior to the main shock of M=7.4, an earthquake of magnitude 6.7 occurred.<br>Only the Nakamura site liquefied during this earthquake.<br>The subject zone is seismically very active and had been shaken in 1897 and<br>1936 by earthquakes of Magnitudes 7.3 and 7.7<br>The original Tsuchida (1979) paper (in Japanese) could not be accessed.<br>However the same author's paper of 1980 in English was used to obtain<br>borelogs. Nakajima 18 site was identified as Site A by Tokimatsu (1983).<br>SPT energy is estimated as 65 % by Seed et al. (84)

Summary of Data

	Cetin et al. (2016)	Idriss&Boulanger (2010)	Seed et.al. (1984)		Cetin et al. (2016)	Idriss&Boulanger (2010)	Seed et.al. (1984)
Liquefied?	Yes	Yes	Yes	D_{50}	0.350 ± 0.050		0.35
Data Class	B			% Fines	3.0 ± 2.0	3.0	3
Critical Depth Range	8.0 - 20.0	20.0	20.0	% PI			
Depth to GWT (ft)	8.0 ± 0.3	7.9	8.0				
σ_v (psf)	1630.0 ± 251.8	2401.8	2400.0	N	9.6 ± 3.9	12.0	12.0
σ_v' (psf)	1255.6 ± 129.5	1649.9	1650.0	C_R	0.90	0.95	
a_{max} (g)	0.240 ± 0.072	0.24	0.240	C_S	1.00	1.00	
r_d	0.97 ± 0.061	0.96	0.960	C_B	1.00	1.00	
CSR	0.197 ± 0.061	0.217	0.220	C_E	1.09	1.09	1.090
Equivalent Magnitude	7.7	7.7		C_N	1.30	1.13	1.1
MSF		0.95	1.02	$(N_1)_{60}$	12.2 ± 5.0	14.1	14.50
CSRN		0.223	0.22				

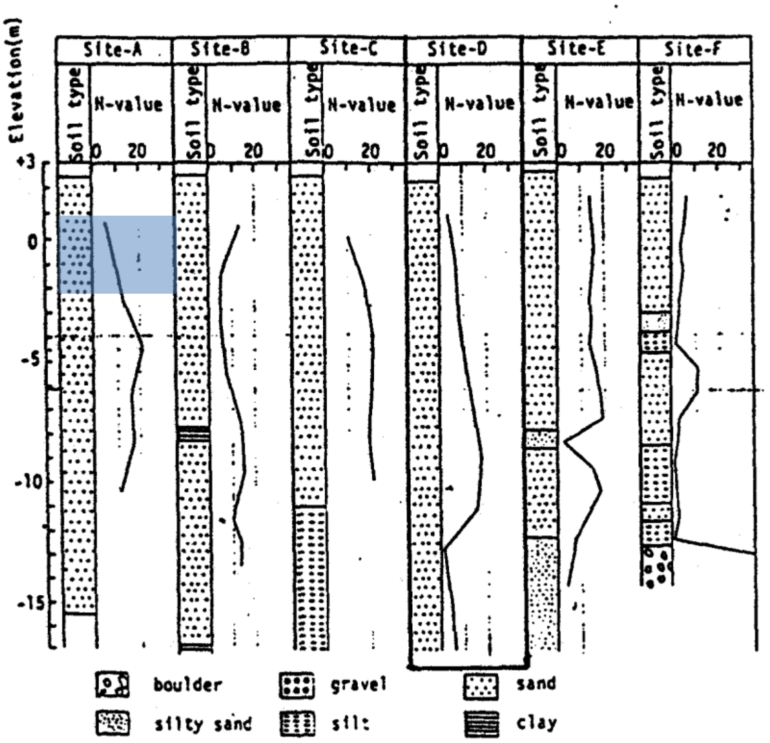

γ_m=110±3 pcf

γ_s=125±3 pcf

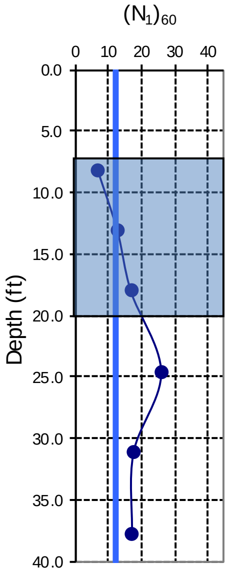

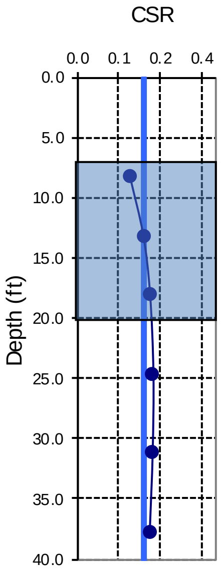

Depth (m)	Depth (ft)	CSR	N	C_N	C_R	$(N1)_{60}$
2.5	8.2	0.156	5.0	1.54	0.82	***6.9***
4.0	13.1	0.192	10.0	1.33	0.89	***12.9***
5.5	18.0	0.211	14.0	1.18	0.94	***16.9***
7.5	24.6	0.220	23.0	1.05	0.99	26.1
9.5	31.2	0.217	17.0	0.95	1.00	17.7
11.5	37.7	0.210	18.0	0.88	1.00	17.3
					**Mean:**	12.2
					**St. Dev.**	5.0

**Case number:**	57
**Earthquake:**	1978 Miyagiken-Oki
**Magnitude:**	7.7 (Mw) USGS Centennial Earthquake Catalog
**Location:**	Arahama
**References:**	Tohno et al. (1981)<br>Fear et al. (1995)
**Nature of Failure:**	Sand volcanoes, houses tilted, ground settlement of ~20 cm. Uplifted buried sewage treatment tank.
**Comments:**	The site is located in Watari Town, district of Arahama.<br>Prior to the main shock of M=7.4, an earthquake of magnitude 6.7 occurred.<br>The subject zone is seismically very active and had been shaken in 1897 and 1936 by earthquakes of Magnitudes 7.3 and 7.7<br>Arahama area is covered with very soft sandy deposits; about 9 m in thickness, N values generally less than 10.<br>SPT values were taken before main earthquake (M=7.4) in 1974.<br>SPT energy was estimated as 65 % by Seed et al. (84)

**Summary of Data**	Cetin et al. (2016)	Idriss&Boulanger (2010)	Seed et.al. (1984)		Cetin et al. (2016)	Idriss&Boulanger (2010)	Seed et.al. (1984)
Liquefied?	Yes	Yes	Yes	D_{50}	0.450 ± 0.050		0.6
Data Class	B			% Fines	0.0 ± 2.0	0.0	0
Critical Depth Range	6.6 - 26.2	21.0	21.0	% PI			
Depth to GWT (ft)	3.0 ± 0.3	3.0	3.0				
σ_v (psf)	1938.5 ± 395.9	2527.1	2520.0	N	8.9 ± 2.4	10.0	10.0
σ_v' (psf)	1102.1 ± 194.1	1399.3	1400.0	C_R	0.92	0.95	
a_{max} (g)	0.200 ± 0.060	0.20	0.200	C_S	1.00	1.00	
r_d	0.94 ± 0.070	0.95	0.950	C_B	1.00	1.00	
CSR	0.216 ± 0.067	0.223	0.220	C_E	1.09	1.09	1.090
Equivalent Magnitude	7.7	7.7		C_N	1.39	1.23	1.2
MSF		0.95	1.00	$(N_1)_{60}$	12.4 ± 3.3	12.8	13.00
CSRN		0.225	0.22				

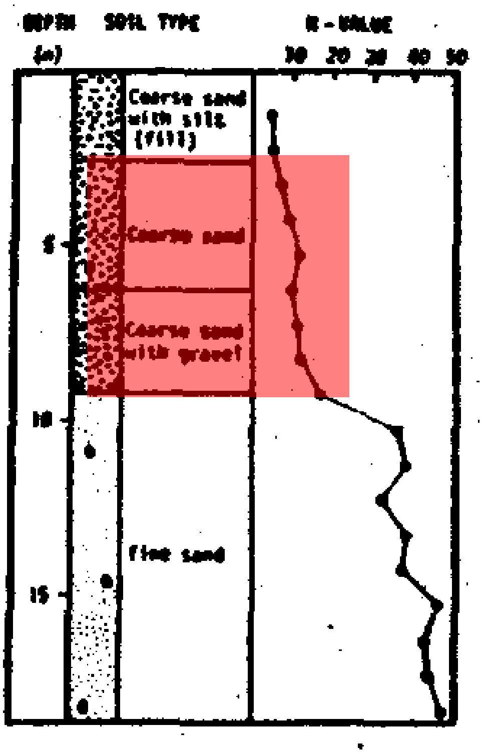

γ_m=110±3 pcf

γ_s=120±3 pcf

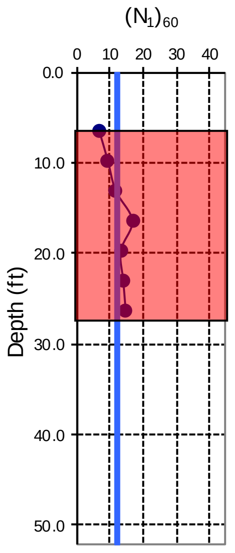

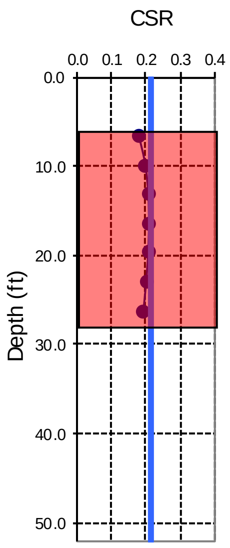

Depth (m)	Depth (ft)	CSR	N	C_N	C_R	$(N1)_{60}$
2.0	6.6	0.180	4	1.99	0.80	***6.9***
3.0	9.8	0.199	6	1.71	0.85	***9.5***
4.0	13.1	0.207	8	1.52	0.89	***11.8***
5.0	16.4	0.209	12	1.39	0.92	***16.7***
6.0	19.7	0.207	10	1.28	0.95	***13.3***
7.0	23.0	0.201	11	1.20	0.98	***14.0***
8.0	26.2	0.193	12	1.13	1.00	***14.8***
					**Mean:**	12.4
					**St. Dev.**	3.3

**Case number:**	58
**Earthquake:**	1978 Miyagiken-Oki
**Magnitude:**	7.7 (Mw) USGS Centennial Earthquake Catalog
**Location:**	Hiyori-18
**References:**	Tsuchida et al (1979, 1980) Tohno et al. (1981)<br>Fear et al. (1995)
**Nature of Failure:**	Not specifically given, but Seed et al (1984) defined the site as "liquefied."
**Comments:**	Prior to the main shock of M=7.4, an earthquake of magnitude 6.7 occurred.<br>The subject zone is seismically very active and had been shaken in 1897 and<br>1936 by earthquakes of Magnitudes 7.3 and 7.7<br>The original Tsuchida (1979) paper (in Japanese) could not be accessed.<br>However the same author's paper of 1980 in English was used to obtain<br>borelogs. Hiyori-18 site was claimed by Tokimatsu (1993) to be by Site C.<br>SPT energy is estimated as 65 % by Seed et al. (84)

Summary of Data

	Cetin et al. (2016)	Idriss&Boulanger (2010)	Seed et.al. (1984)		Cetin et al. (2016)	Idriss&Boulanger (2010)	Seed et.al. (1984)
Liquefied?	Yes	Yes	Yes	D_{50}	0.150 ± 0.050		0.15
Data Class	B			% Fines	20.0 ± 2.0	20.0	20
Critical Depth Range	8.2 - 13.1	17.1	17.0	% PI			
Depth to GWT (ft)	8.0 ± 0.3	7.9	8.0				
σ_v (psf)	1199.5 ± 101.7	2046.8	2040.0	N	9.1 ± 1.5	9.0	9.0
σ_v' (psf)	1033.4 ± 55.8	1482.9	1480.0	C_R	0.86	0.95	
a_{max} (g)	0.240 ± 0.072	0.24	0.240	C_S	1.00	1.00	
r_d	0.98 ± 0.048	0.96	0.960	C_B	1.00	1.00	
CSR	0.177 ± 0.054	0.207	0.205	C_E	1.09	1.09	1.090
Equivalent Magnitude	7.7	7.7		C_N	1.43	1.19	1.2
MSF		0.95	1.00	$(N_1)_{60}$	12.2 ± 2.0	11.1	11.50
CSRN		0.210	0.21				

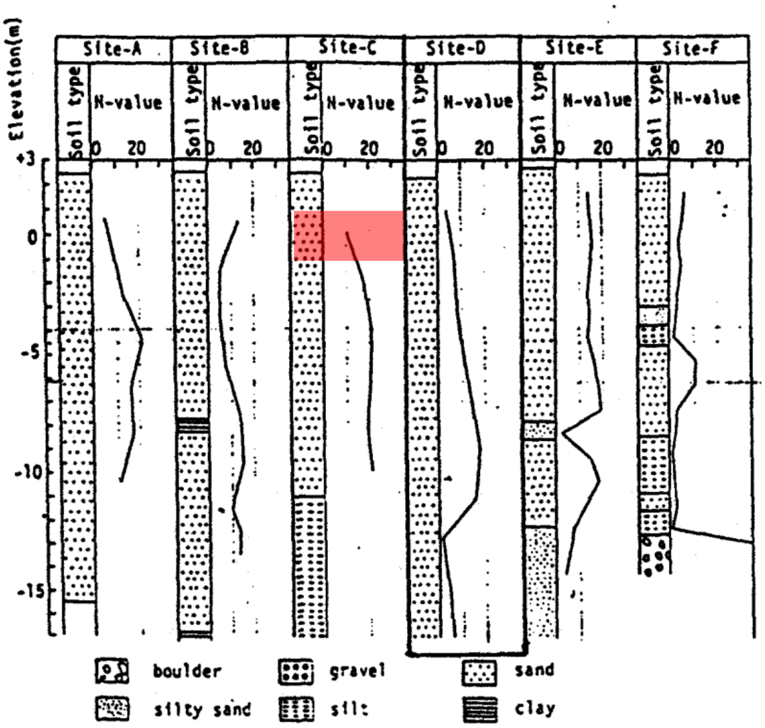

γ_m=110±3 pcf

γ_s=120±3 pcf

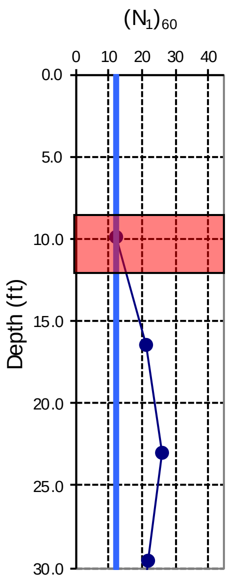

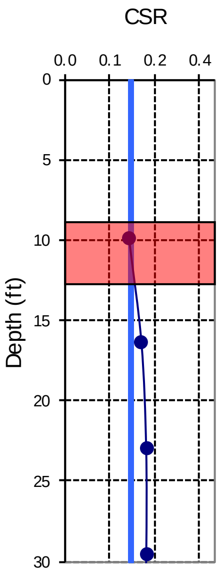

Depth (m)	Depth (ft)	CSR	N	C_N	C_R	$(N1)_{60}$
3.0	9.8	0.171	9.0	1.46	0.85	***12.2***
5.0	16.4	0.207	17.0	1.25	0.92	21.3
7.0	23.0	0.220	22.0	1.10	0.98	25.9
9.0	29.5	0.220	20.0	1.00	1.00	21.8
11.0	36.1	0.214	18.0	0.92	1.00	18.1
					**Mean:**	12.2
					**St. Dev.**	2.0

assigned

**Case number:**	59
**Earthquake:**	1978 Miyagiken-Oki
**Magnitude:**	7.7 (Mw) USGS Centennial Earthquake Catalog
**Location:**	Ishinomaki-2
**References:**	Ishihara et al. (1980)<br>Fear et al. (1995)
**Nature of Failure:**	"Many sand boils showed up about 10 minutes after the main shock. The sand spouts reached a height of approximately 1 m. above the ground surface." Surface fissuring and cracks were also observed near the tank yard.
**Comments:**	Prior to the main shock of M=7.4, an earthquake of magnitude 6.7 occurred. The subject zone is seismically very active and had been shaken in 1897 and 1936 by earthquakes of Magnitudes 7.3 and 7.7<br>The site is near the fishery port in the city of Ishinomaki. Loose sand deposit to a depth of 12 m has blow count values on the order of 5. The site has been improved by vibroflotation and compaction piles in 1975. Unimproved sand deposits in the vicinity were liquefied. The nearest strong ground motion on rock outcrop, a PGA of 289g, was recorded at Kaihoku bridge site which is located 5 km north of the oil tank site. Ishihara et al. Adopted a PGA value of 0.185g for his liquefaction analysis; considering the site response of soft soil deposits. Triaxial dynamic tests on frozen samples revealed that the FS in the depth range of 3-9 m is less than 1.(5% double amplitude axial strain in 20 cycles.)<br>The site experienced an intensity of shaking of V on JMAMS.<br>SPT energy is estimated as 65 % by Seed et al. (84)

Summary of Data

	Cetin et al. (2016)	Idriss&Boulanger (2010)	Seed et.al. (1984)		Cetin et al. (2016)	Idriss&Boulanger (2010)	Seed et.al. (1984)
Liquefied?	Yes	Yes	Yes	D_{50}	0.150 ± 0.050		0.15
Data Class	B			% Fines	10.0 ± 2.0	10.0	10
Critical Depth Range	4.6 - 19.7	11.5	13.0	% PI			
Depth to GWT (ft)	4.6 ± 2.0	4.6	5.0				
σ_v (psf)	1410.8 ± 303.6	1378.4	1560.0	N	4.0 ± 0.3	3.7	4.0
σ_v' (psf)	939.9 ± 179.9	939.8	1060.0	C_R	0.88	0.85	
a_{max} (g)	0.200 ± 0.060	0.20	0.200	C_S	1.00	1.00	
r_d	0.93 ± 0.054	0.98	0.970	C_B	1.00	1.00	
CSR	0.181 ± 0.061	0.186	0.185	C_E	1.09	1.09	1.090
Equivalent Magnitude	7.7	7.7		C_N	1.50	1.61	1.3
MSF		0.95	1.03	$(N_1)_{60}$	5.7 ± 0.5	5.5	6.00
CSRN		0.184	0.18				

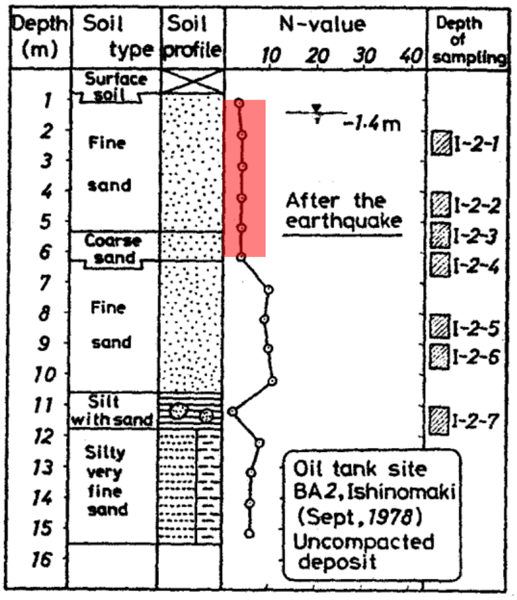

Fig. 14. Standard penetration resistance and depths of Osterberg sampling at the uncompacted site after the earthquake

γ_m=110±3 pcf

γ_s=120±3 pcf

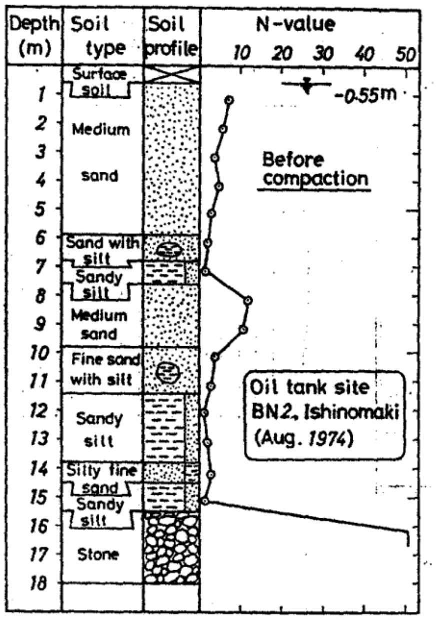

Fig. 11. A soil profile and standard penetration resistance before compaction

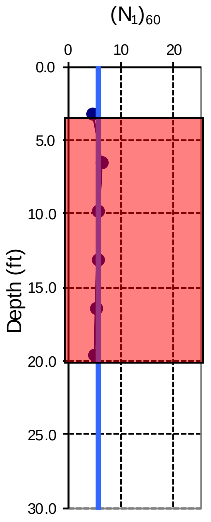

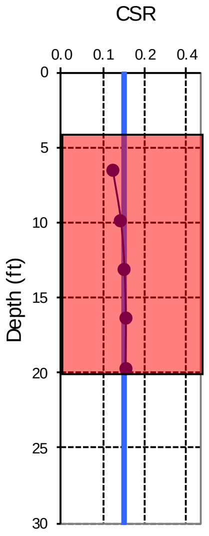

Depth (m)	Depth (ft)	CSR	N	C_N	C_R	$(N1)_{60}$	
1.0	3.3	NA	3.0	2.00	0.73	4.8	too close to surface
2.0	6.6	0.151	4.0	1.85	0.80	***6.4***	
3.0	9.8	0.173	4.0	1.62	0.85	***6.0***	
4.0	13.1	0.183	4.0	1.46	0.89	***5.6***	
5.0	16.4	0.186	4.0	1.34	0.92	***5.4***	
6.0	19.7	0.185	4.0	1.24	0.95	***5.2***	
					**Mean:**	5.7	
					**St. Dev.**	0.5	

**Case number:**	60
**Earthquake:**	1978 Miyagiken-Oki
**Magnitude:**	7.7 (Mw) USGS Centennial Earthquake Catalog
**Location:**	Ishinomaki-4
**References:**	Ishihara et al. (1980)<br>Fear et al. (1995)
**Nature of Failure:**	Nonliquefied
**Comments:**	Prior to the main shock of M=7.4, an earthquake of magnitude 6.7 occurred.<br>The subject zone is seismically very active and had been shaken in 1897 and 1936 by earthquakes of Magnitudes 7.3 and 7.7<br>The site is near the fishery port in the city of Ishinomaki. Loose sand deposit to a depth of 12 m has blow count values on the order of 5. The site has been improved by vibro-flotation and compaction piles in 1975. Unimproved sand deposits in the vicinity were liquefied. The nearest strong ground motion on rock outcrop, a PGA of 289g, was recorded at Kaihoku bridge site which is located 5 km north of the oil tank site. Ishihara et al. Adopted a PGA value of 0.185g for his liquefaction analysis; considering the site response of soft soil deposits. Triaxial dynamic tests on frozen samples revealed that the FS in the depth range of 3-9 m is less than 1.(5% double amplitude axial strain in 20 cycles.)<br>65 kN/m^3 (1316 psf) surcharge due to oil storage at the time of the earthquake<br>SPT energy is estimated as 73 % by Seed et al. (84)

Summary of Data

	Cetin et al. (2016)	Idriss&Boulanger (2010)	Seed et.al. (1984)		Cetin et al. (2016)	Idriss&Boulanger (2010)	Seed et.al. (1984)
Liquefied?	No	No	No	D_{50}	0.180 ± 0.050		0.18
Data Class	B			% Fines	10.0 ± 2.0	10.0	10
Critical Depth Range	4.6 - 23.0	14.8	20.0	% PI			
Depth to GWT (ft)	4.6 ± 0.3	4.6	5.0				
σ_v (psf)	3015.5 ± 384.0	1817.0	2400.0	N	23.4 ± 2.7	14.2	15.0
σ_v' (psf)	2442.2 ± 194.9	1190.5	1460.0	C_R	0.89	0.95	
a_{max} (g)	0.200 ± 0.060	0.20	0.200	C_S	1.00	1.00	
r_d	0.97 ± 0.060	0.97	0.960	C_B	1.00	1.00	
CSR	0.156 ± 0.048	0.188	0.205	C_E	1.21	1.21	1.210
Equivalent Magnitude	7.7	7.7		C_N	0.93	1.28	1.2
MSF		0.95	1.03	$(N_1)_{60}$	23.5 ± 2.7	20.9	21.00
CSRN		0.183	0.20				

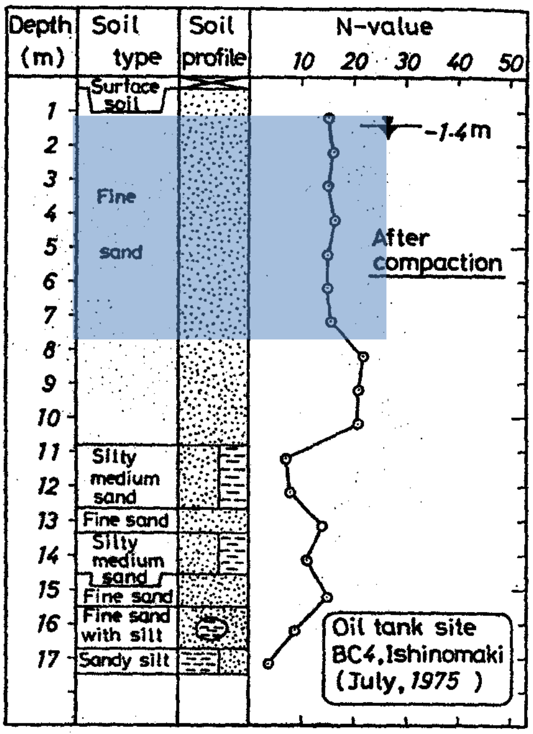

γ_m=120±3 pcf

γ_s=125±3 pcf

Fig. 12. Standard penetration resistance after compaction

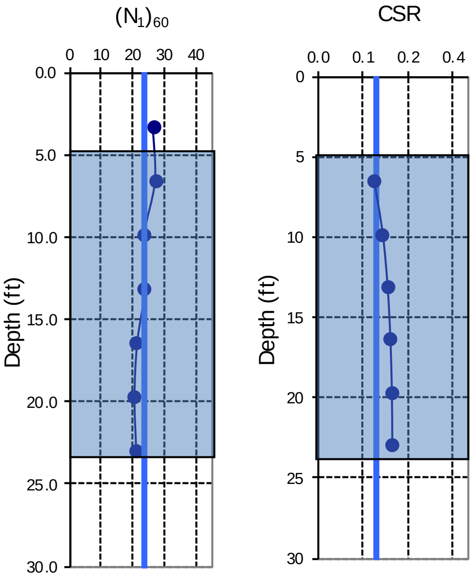

Depth (m)	Depth (ft)	CSR	N	C_N	C_R	$(N1)_{60}$
1.0	3.3	NA	15.0	2.00	0.73	***26.6***
2.0	6.6	0.152	16.0	1.77	0.80	***27.3***
3.0	9.8	0.176	15.0	1.55	0.85	***23.8***
4.0	13.1	0.189	16.0	1.40	0.89	***23.9***
5.0	16.4	0.197	15.0	1.28	0.92	***21.4***
6.0	19.7	0.201	15.0	1.19	0.95	***20.5***
7.0	23.0	0.202	16.0	1.12	0.98	***21.1***
					**Mean:**	23.5
					**St. Dev.**	2.7

**Case number:**	61
**Earthquake:**	1978 Miyagiken-Oki
**Magnitude:**	7.7 (Mw) USGS Centennial Earthquake Catalog
**Location:**	Kitawabuchi 2
**References:**	Iwasaki et al. (: Fear et al. (1995) Tohno et al. (1981)
**Nature of Failure:**	sand boils<br>fissures on the bank
**Comments:**	Kitawabuchi 2 is located on the inside of the right bank of the Eai River.<br>The ground generally consists of sand type soil, over interbedded clay and<br>hard clay, overlying sand. 0-10 m consists of thin sand layers and sandy silt<br>layers with shells where N<10; below 10 m is sand<br>Iwasaki et al. (1981) predicted the liquefied layer at depth of 3-4 m.<br>Tohno et al (1981) :<br>Prior to the main shock of M=7.4, an earthquake of magnitude 6.7 occurred.<br>The subject zone is seismically very active and had been shaken in 1897 and<br>1936 by earthquakes of Magnitudes 7.3 and 7.7<br>liqeufaction occurred at the dike of Eai River; sand volcanoes were located on<br>the old river bed near the right bank of the river and cracks were formed on the<br>surface of the dike. The surface and the slope of the bank cracked at the site<br>of the entrance to the old river bed; however the surface of the bed didn't subside<br>The sand boil characteristics matched the characteristics of the sand layer at<br>3.5-5 m depth.<br>SPT values were taken after main earthquake (M=7.4)<br>SPT energy is estimated as 60 % by Seed et al. (84)

**Summary of Data**	Cetin et al. (2016)	Idriss&Boulanger (2010)	Seed et.al. (1984)		Cetin et al. (2016)	Idriss&Boulanger (2010)	Seed et.al. (1984)
Liquefied?	Yes	Yes	Yes	D_{50}	0.530 ± 0.050		0.53
Data Class	B			% Fines	5.0 ± 2.0	5.0	5
Critical Depth Range	9.8 - 13.1	11.2	11.0	% PI			
Depth to GWT (ft)	9.8 ± 0.3	10.2	10.0				
σ_v (psf)	1181.1 ± 72.4	1294.9	1300.0	N	11.1 ± 1.6	11.0	11.0
σ_v' (psf)	1078.7 ± 45.3	1232.2	1240.0	C_R	0.87	0.85	
a_{max} (g)	0.280 ± 0.084	0.28	0.280	C_S	1.00	1.00	
r_d	0.89 ± 0.052	0.98	0.980	C_B	1.00	1.00	
CSR	0.177 ± 0.054	0.187	0.185	C_E	1.00	1.00	1.000
Equivalent Magnitude	7.7	7.7		C_N	1.40	1.32	1.2
MSF		0.95	1.00	$(N_1)_{60}$	13.5 ± 2.0	12.3	13.50
CSRN		0.187	0.19				

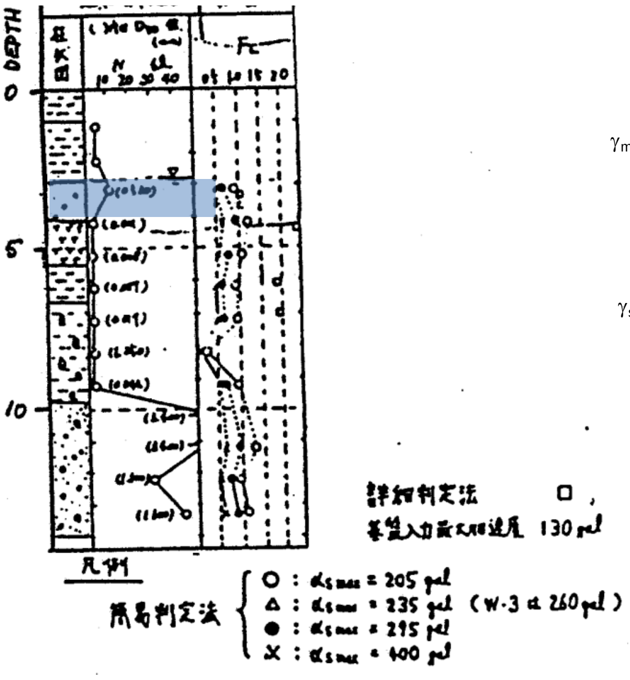

γ_m=100±3 pcf

γ_s=120±3 pcf

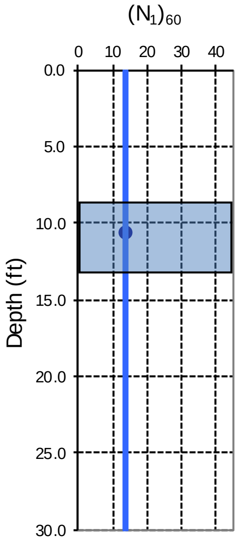

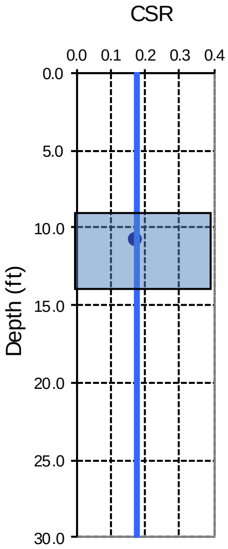

Depth (m)	Depth (ft)	CSR	N	C_N	C_R	$(N1)_{60}$
1.0	3.3	NA	4.0	2.0	0.7	5.9
2.0	6.6	NA	4.0	1.8	0.8	5.7
3.3	10.7	0.172	11.0	1.4	0.9	***13.5***
4.3	13.9	0.188	3.0	1.3	0.9	3.5
5.3	17.2	0.195	2.0	1.2	0.9	2.3
					**Mean:**	13.5
					**St. Dev.**	2.0

assigned

**Case number:**	62
**Earthquake:**	1978 Miyagiken-Oki
**Magnitude:**	7.7 (Mw) USGS Centennial Earthquake Catalog
**Location:**	Kitawabuchi-3
**References:**	Iwasaki et al. (1981) Tohno et al. (1981)<br>Fear et al. (1995)
**Nature of Failure:**	No surface evidence of liquefaction.
**Comments:**	Kitawabuchi 3 is located on the inside of the right bank of the Eai River<br>upstream from Kitawabuchi 2.<br>The ground generally consists of sand type soil, over interbedded clay and<br>Sand layer at depths of 3-10+ m.<br>Tohno et al (1981) :<br>Prior to the main shock of M=7.4, an earthquake of magnitude 6.7 occurred.<br>The subject zone is seismically very active and had been shaken in 1897 and<br>1936 by earthquakes of Magnitudes 7.3 and 7.7<br>Liqeufaction occurred at the dike of Eai River; sand volcanoes were located on<br>the old river bed near the right bank of the river and cracks were formed on the<br>surface of the dike. The surface and the slope of the bank cracked at the site<br>of the entrance to the old river bed; however the surface of the bed didn't subside<br>The sand boil characteristics matched the characteristics of the sand layer at<br>3.5-5 m depth.<br>SPT values were taken after main earthquake (M=7.4)<br>SPT energy is estimated as 73 % by Seed et al. (84)

Summary of Data

	Cetin et al. (2016)	Idriss&Boulanger (2010)	Seed et.al. (1984)		Cetin et al. (2016)	Idriss&Boulanger (2010)	Seed et.al. (1984)
Liquefied?	No	No	No	D_{50}	0.410 ± 0.050		0.41
Data Class	B			% Fines	0.0 ± 2.0	0.0	0
Critical Depth Range	10.0 - 18.0	15.7	20.0	% PI			
Depth to GWT (ft)	10.0 ± 3.0	10.2	10.0				
σ_v (psf)	1582.7 ± 166.8	1879.7	2400.0	N	13.2 ± 5.1	13.2	23.0
σ_v' (psf)	1331.7 ± 178.1	1524.6	1780.0	C_R	0.90	0.95	
a_{max} (g)	0.280 ± 0.084	0.28	0.320	C_S	1.00	1.00	
r_d	0.99 ± 0.061	0.97	0.960	C_B	1.00	1.00	
CSR	0.214 ± 0.073	0.216	0.235	C_E	1.21	1.21	1.210
Equivalent Magnitude	7.7	7.7		C_N	1.26	1.16	1.1
MSF		0.95	1.02	$(N_1)_{60}$	18.0 ± 6.9	17.6	29.00
CSRN		0.220	0.23				

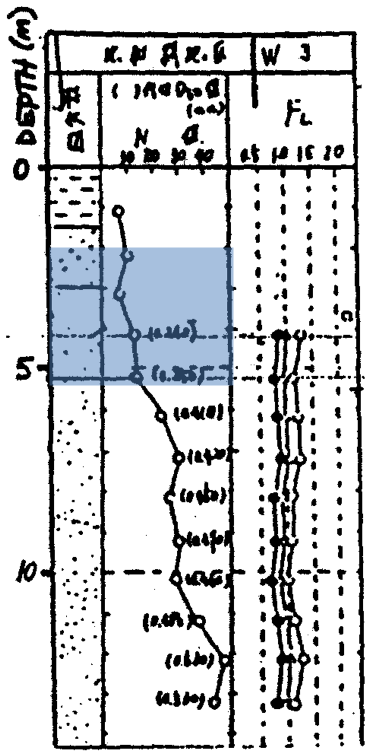

γ_m=110±3 pcf

γ_s=120±3 pcf

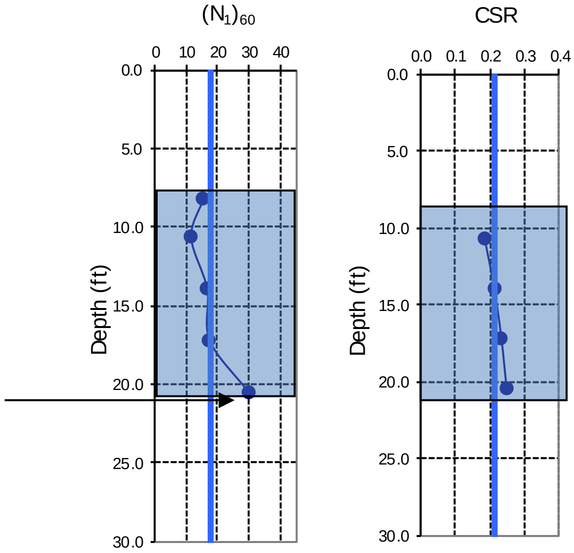

Seed et al. (1984)

Depth (m)	Depth (ft)	CSR	N	C_N	C_R	$(N1)_{60}$
2.5	8.2	NA	10	1.53	0.82	***15.2***
3.3	10.7	0.188	8	1.36	0.86	***11.3***
4.3	13.9	0.214	12	1.26	0.90	***16.4***
5.3	17.2	0.233	13	1.18	0.93	***17.2***
6.3	20.5	0.247	23	1.11	0.96	***29.6***
					**Mean:**	18.0
					**St. Dev.**	6.9

Water table info is not accurate.

**Case number:**	63
**Earthquake:**	1978 Miyagiken-Oki
**Magnitude:**	7.7 (Mw) USGS Centennial Earthquake Catalog
**Location:**	Nakajima-2
**References:**	Tsuchida et al (1979, 1980) Tohno et al. (1981)<br>Fear et al. (1995)
**Nature of Failure:**	No surface evidence of liquefaction.
**Comments:**	Prior to the main shock of M=7.4, an earthquake of magnitude 6.7 occurred.<br>Only the Nakamura site liquefied during this earthquake.<br>The subject zone is seismically very active and had been shaken in 1897 and<br>1936 by earthquakes of Magnitudes 7.3 and 7.7<br>The original Tsuchida (1979) paper (in Japanese) could not be accessed.<br>However the same author's paper of 1980 in English was used to obtain<br>borelogs. Nakajima 18 site was claimed by Tokimatsu (1993) to be by Site A.<br>SPT energy was estimated as 65 % by Seed et al. (84)

**Summary of Data**	Cetin et al. (2016)	Idriss&Boulanger (2010)	Seed et.al. (1984)		Cetin et al. (2016)	Idriss&Boulanger (2010)	Seed et.al. (1984)
Liquefied?	No	No	No	D_{50}	0.120 ± 0.050		0.12
Data Class	B			% Fines	26.0 ± 2.0	26.0	26
Critical Depth Range	10.0 - 20.0	15.1	15.0	% PI			
Depth to GWT (ft)	8.0 ± 0.3	7.9	8.0				
σ_v (psf)	1755.0 ± 210.8	1796.1	1800.0	N	11.9 ± 2.3	10.0	10.0
σ_v' (psf)	1318.2 ± 110.0	1357.6	1360.0	C_R	0.91	0.95	
a_{max} (g)	0.240 ± 0.072	0.24	0.240	C_S	1.00	1.00	
r_d	0.97 ± 0.065	0.97	0.970	C_B	1.00	1.00	
CSR	0.201 ± 0.062	0.200	0.200	C_E	1.09	1.09	1.090
Equivalent Magnitude	7.7	7.7		C_N	1.27	1.23	1.2
MSF		0.95	1.03	$(N_1)_{60}$	14.9 ± 2.9	12.7	13.00
CSRN		0.200	0.195				

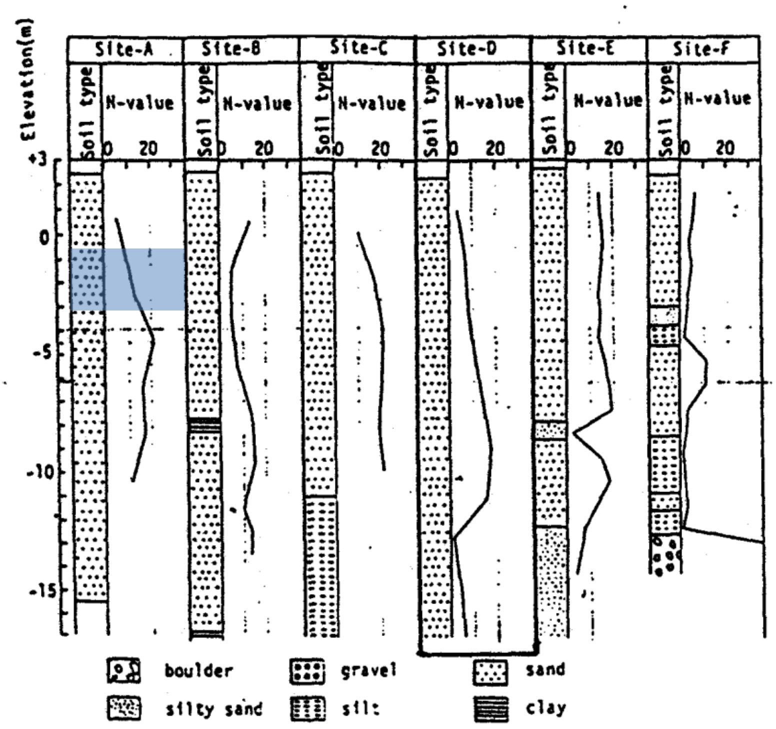

γ_m=110±3 pcf

γ_s=125±3 pcf

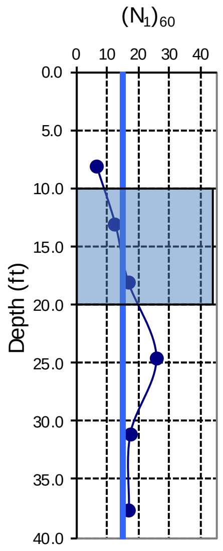

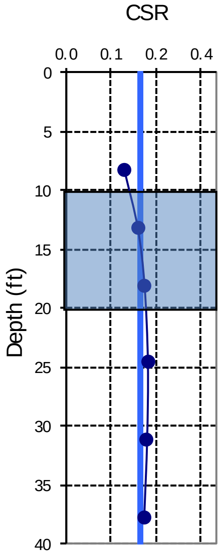

Depth (m)	Depth (ft)	CSR	N	C_N	C_R	$(N1)_{60}$
2.5	8.2	0.156	5.0	1.54	0.82	6.9
4.0	13.1	0.192	10.0	1.33	0.89	***12.9***
5.5	18.0	0.211	14.0	1.18	0.94	***16.9***
7.5	24.6	0.220	23.0	1.05	0.99	26.1
9.5	31.2	0.217	17.0	0.95	1.00	17.7
11.5	37.7	0.210	18.0	0.88	1.00	17.3
					**Mean:**	14.9
					**St. Dev.**	2.9

**Case number:**	64
**Earthquake:**	1978 Miyagiken-Oki
**Magnitude:**	7.7 (Mw) USGS Centennial Earthquake Catalog
**Location:**	Nakamura-1
**References:**	Iwasaki et al. (1978) Tohno et al. (1981)<br>Fear et al. (1995)
**Nature of Failure:**	No surface evidence of liquefaction.
**Comments:**	The site is located in the inside base towards the south end of an embankment along the left bank of the Natori River.<br>Prior to the main shock of M=7.4, an earthquake of magnitude 6.7 occurred.<br>The subject zone is seismically very active and had been shaken in 1897 and 1936 by earthquakes of Magnitudes 7.3 and 7.7<br>PGA is estimated as 0.18 g in Iwasaki (1978). Based on the results of cyclic triaxial test, the FS against liquefaction is calculated as less than 1 in the sand layer between the depths of 2-7 m.<br>From Tohno et al. (1981)<br>The river embankment was partially collapsed and cracked in both sides at Nakamura located ~3 km upstream from the mouth of Natori River. Sand boils were also observed after the February, 1978 (M=6.4) earthquake. The textural parameters of the liquefied soil revealed that the possibly liquefied layer is sandy deposits at 1.4-3 m.<br>SPT values were taken after main earthquake (M=7.4)<br>SPT energy is estimated as 67 % by Seed et al. (84)

Summary of Data

	Cetin et al. (2016)	Idriss&Boulanger (2010)	Seed et.al. (1984)		Cetin et al. (2016)	Idriss&Boulanger (2010)	Seed et.al. (1984)
Liquefied?	No	No	No	D_{50}	0.280 ± 0.050		0.28
Data Class	B			% Fines	4.0 ± 2.0	4.0	4
Critical Depth Range	6.6 - 13.1	11.2	11.0	% PI			
Depth to GWT (ft)	3.0 ± 0.3	3.0	3.0				
σ_v (psf)	1186.0 ± 138.6	1315.8	1320.0	N	15.8 ± 4.0	19.0	19.0
σ_v' (psf)	756.1 ± 73.4	814.5	820.0	C_R	0.85	0.85	
a_{max} (g)	0.320 ± 0.096	0.32	0.320	C_S	1.00	1.00	
r_d	0.99 ± 0.045	0.98	0.980	C_B	1.00	1.00	
CSR	0.324 ± 0.099	0.329	0.330	C_E	1.12	1.12	1.120
Equivalent Magnitude	7.7	7.7		C_N	1.67	1.45	1.5
MSF		0.95	1.02	$(N_1)_{60}$	24.9 ± 6.4	26.2	31.50
CSRN		0.315	0.33				

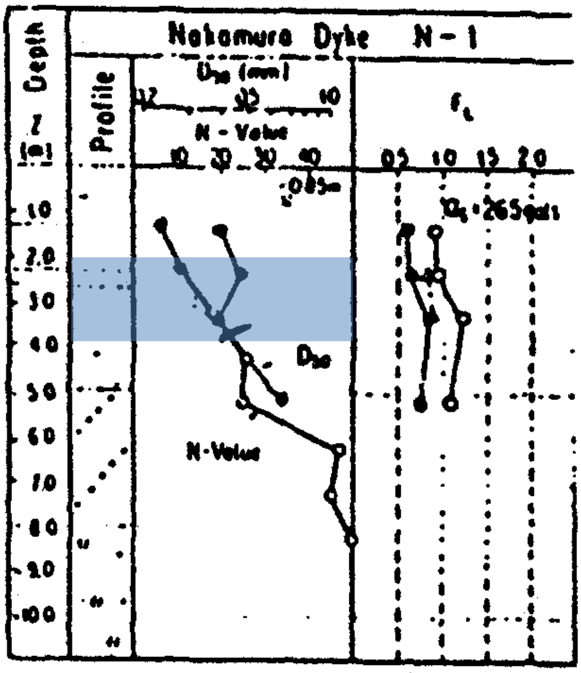

γ_m=110±3 pcf

γ_s=125±3 pcf

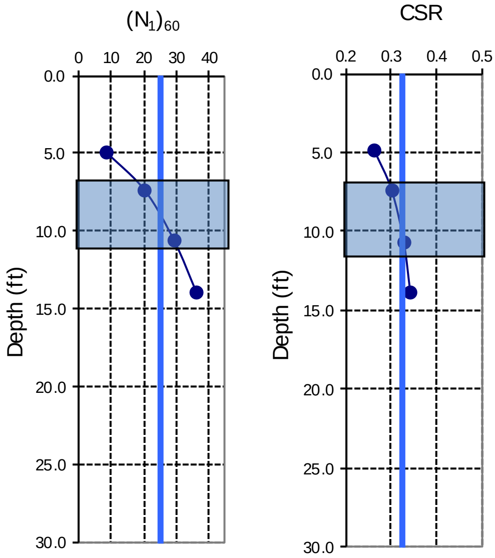

Depth (m)	Depth (ft)	CSR	N	C_N	C_R	$(N1)_{60}$
1.5	4.9	0.264	5	2.00	0.77	8.6
2.3	7.4	0.302	12	1.87	0.81	***20.4***
3.3	10.7	0.329	19	1.62	0.86	***29.4***
4.3	13.9	0.344	25	1.45	0.90	36.2
					**Mean:**	24.9
					**St. Dev.**	6.4

**Case number:**	65
**Earthquake:**	1978 Miyagiken-Oki
**Magnitude:**	7.7 (Mw) USGS Centennial Earthquake Catalog
**Location:**	Nakamura-4
**References:**	Iwasaki et al. (1978) Tohno et al. (1981)<br>Fear et al. (1995)
**Nature of Failure:**	Sand boils, cracking.
**Comments:**	The site is located in the inside base towards the north end of an embankment along the left bank of Natori River<br>Prior to the main shock of M=7.4, an earthquake of magnitude 6.7 occurred.<br>The subject zone is seismically very active and had been shaken in 1897 and 1936 by earthquakes of Magnitudes 7.3 and 7.7<br>PGA is estimated as 0.18 g in the paper. Based on the results of cyclic triaxial test, the FS against liquefaction is calculated as less than 1 in the sand layer between the depths of 2-7 m.<br>From Tohno et al. (1981)<br>The river embankment was partially collapsed and cracked in both sides at Nakamura located ~3 km upstream from the mouth of Natori River. Sand boils were also observed after the February, 1978 (M=6.4) earthquake. The textural parameters of the liquefied soil revealed that the possibly liquefied layer is sandy deposits at 1.4-3 m.<br>SPT values were taken after main earthquake (M=7.4)<br>SPT energy was estimated as 60 % by Seed et al. (84)

Summary of Data

	Cetin et al. (2016)	Idriss&Boulanger (2010)	Seed et.al. (1984)		Cetin et al. (2016)	Idriss&Boulanger (2010)	Seed et.al. (1984)
Liquefied?	Yes	Yes	Yes	D_{50}	0.700 ± 0.050		0.7
Data Class	B			% Fines	5.0 ± 2.0	5.0	5
Critical Depth Range	9.8 - 16.4	9.2	11.0	% PI			
Depth to GWT (ft)	1.6 ± 0.3	1.6	2.0				
σ_v (psf)	1558.4 ± 135.8	1106.9	1320.0	N	5.6 ± 0.4	4.7	5.0
σ_v' (psf)	841.9 ± 73.7	626.6	760.0	C_R	0.89	0.85	
a_{max} (g)	0.320 ± 0.096	0.32	0.320	C_S	1.00	1.00	
r_d	0.98 ± 0.058	0.99	0.980	C_B	1.00	1.00	
CSR	0.379 ± 0.116	0.346	0.355	C_E	1.00	1.00	1.000
Equivalent Magnitude	7.7	7.7		C_N	1.59	1.70	1.6
MSF		0.95	1.01	$(N_1)_{60}$	7.9 ± 0.6	6.9	8.00
CSRN		0.332	0.35				

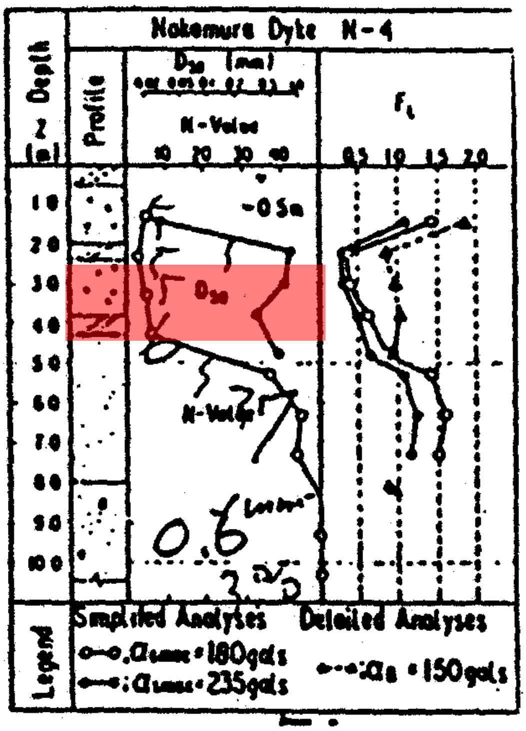

γ_m=110±3 pcf

γ_s=120±3 pcf

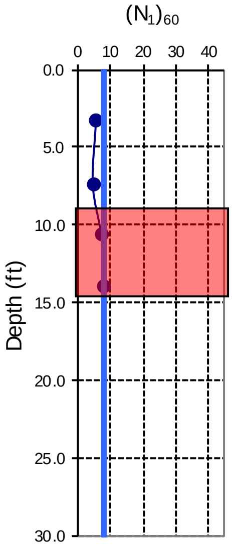

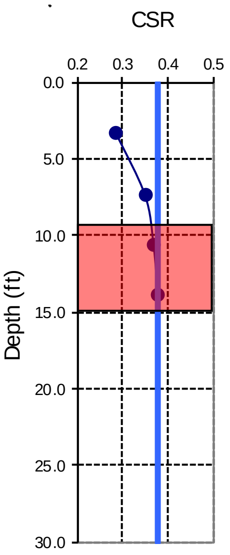

Depth (m)	Depth (ft)	CSR	N	C_N	C_R	$(N1)_{60}$
1.0	3.3	0.285	4	2.00	0.73	5.9
2.3	7.4	0.351	3	2.00	0.81	4.9
3.3	10.7	0.371	5	1.74	0.86	***7.5***
4.3	13.9	0.381	6	1.54	0.90	***8.3***
					**Mean:**	7.9
					**St. Dev.**	0.6

**Case number:**	66
**Earthquake:**	1978 Miyagiken-Oki
**Magnitude:**	7.7 (Mw) USGS Centennial Earthquake Catalog
**Location:**	Nakamura-5
**References:**	Iwasaki et al. (1978) Tohno et al. (1981)<br>Fear et al. (1995)
**Nature of Failure:**	Sand boils
**Comments:**	The site is located in the outside base at the north end of an embankment along the left bank of the Natori River.<br>PGA is estimated as 0.18 g in the paper. Based on the results of cyclic triaxial test, the FS against liquefaction is calculated as less than 1 in the sand layer between the depths of 2-7 m.<br>From Tohno et al. (1981):<br>The river embankment was partially collapsed and cracked in both sides at Nakamura located ~3 km upstream from the mouth of Natori River. Sand boils were also observed after the February, 1978 (M=6.4) earthquake. The textural parameters of the liquefied soil revealed that the possibly liquefied layer is sandy deposits at 1.4-3 m.<br>SPT values were taken after main earthquake (M=7.4)<br>SPT energy was estimated as 60 % by Seed et al. (84)

**Summary of Data**	Cetin et al. (2016)	Idriss&Boulanger (2010)	Seed et.al. (1984)		Cetin et al. (2016)	Idriss&Boulanger (2010)	Seed et.al. (1984)
Liquefied?	Yes	Yes	Yes	D_{50}	0.280 ± 0.050		0.28
Data Class	B			% Fines	4.0 ± 2.0	4.0	4
Critical Depth Range	9.0 - 13.1	11.2	11.0	% PI			
Depth to GWT (ft)	4.3 ± 0.3	4.3	4.0				
σ_v (psf)	1284.8 ± 86.0	1315.8	1320.0	N	7.1 ± 1.5	7.0	7.0
σ_v' (psf)	860.6 ± 48.9	877.2	880.0	C_R	0.86	0.85	
a_{max} (g)	0.320 ± 0.096	0.32	0.320	C_S	1.00	1.00	
r_d	0.97 ± 0.050	0.31	0.980	C_B	1.00	1.00	
CSR	0.302 ± 0.092	0.306	0.305	C_E	1.00	1.00	1.000
Equivalent Magnitude	7.7	7.7		C_N	1.57	1.61	1.5
MSF		0.95	1.02	$(N_1)_{60}$	9.6 ± 2.0	9.6	10.00
CSRN		0.299	0.30				

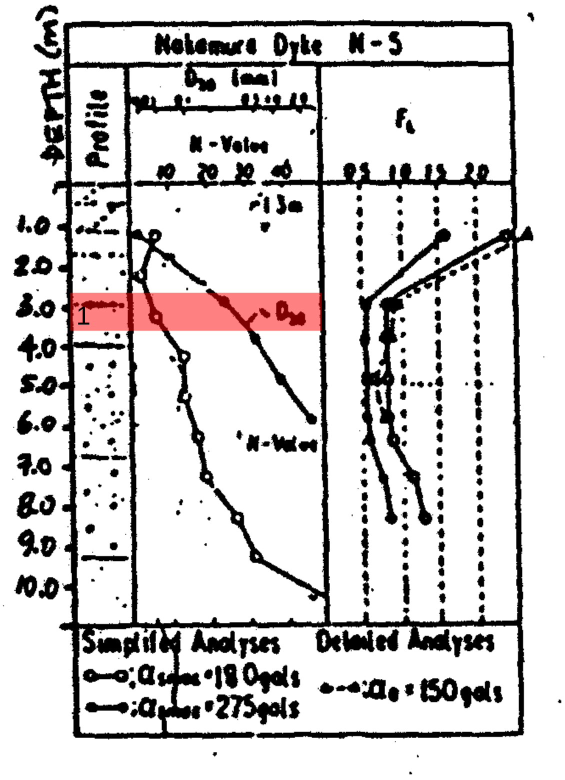

γ_m=110±3 pcf

γ_s=120±3 pcf

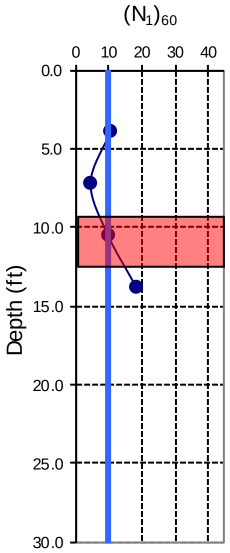

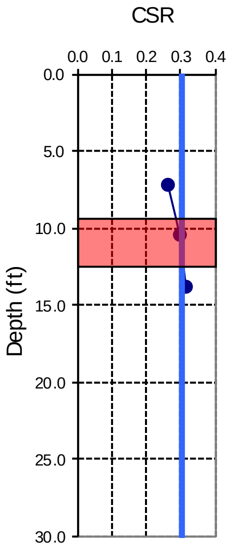

Depth (m)	Depth (ft)	CSR	N	C_N	C_R	$(N1)_{60}$
1.2	3.9	NA	7	2.00	0.75	10.5
2.2	7.2	0.264	3	1.82	0.81	4.4
3.2	10.5	0.298	7	1.60	0.86	***9.6***
4.2	13.8	0.316	14	1.44	0.89	18.1
					**Mean:**	9.6
					**St. Dev.**	2.0

assigned

**Case number:**	67
**Earthquake:**	1978 Miyagiken-Oki
**Magnitude:**	7.7 (Mw) USGS Centennial Earthquake Catalog
**Location:**	Oiiri 1
**References:**	Iwasaki et al. (1978) No mention of Oiiri-1?<br>Fear et al. (1995) Tohno et al. (1981)<br>A.S. Cakmak (1986) "Soil Liquefaction Studies in Japan", Vol. 5 No.1 Jan 1986,<br>Soil Dynamics & Earthquake Engineering.
**Nature of Failure:**	Not given but according to Seed et al (1984) "liquefied."
**Comments:**	Prior to the main shock of M=7.4, an earthquake of magnitude 6.7 occurred.<br>The subject zone is seismically very active and had been shaken in 1897 and<br>1936 by earthquakes of Magnitudes 7.3 and 7.7<br>Iwasaki et al (1978) paper did not mention Oiiri sites. However Cakmak et al.<br>gave a representative depth vs. N profile for Oiiri 1.<br>Tohno et al (1981) :<br>Liquefaction was observed at the dike of Old Kitakami River. Sand volcanoes<br>were found on both sides of the base of the dike and in the vicinity of it.<br>The boil material has the same characteristics of samd layer at depth of 4.5-7m<br>SPT values were taken after main earthquake (M=7.4)<br>SPT energy is estimated as 60 % by Seed et al. (84)

**Summary of Data**	Cetin et al. (2016)	Idriss&Boulanger (2010)	Seed et.al. (1984)		Cetin et al. (2016)	Idriss&Boulanger (2010)	Seed et.al. (1984)
Liquefied?	Yes	Yes	Yes	D_{50}	0.340 ± 0.050		0.34
Data Class	B			% Fines	5.0 ± 2.0	5.0	5
Critical Depth Range	14.0 - 25.0	21.0	21.0	% PI			
Depth to GWT (ft)	14.0 ± 0.3	14.1	14.0				
σ_v (psf)	2200.0 ± 224.6	2213.9	2220.0	N	9.2 ± 2.0	9.0	9.0
σ_v' (psf)	1856.8 ± 115.9	1775.3	1780.0	C_R	0.95	0.95	
a_{max} (g)	0.240 ± 0.072	0.24	0.240	C_S	1.00	1.00	
r_d	0.85 ± 0.081	0.95	0.950	C_B	1.00	1.00	
CSR	0.157 ± 0.050	0.185	0.185	C_E	1.00	1.00	1.000
Equivalent Magnitude	7.7	7.7		C_N	1.07	1.10	1.1
MSF		0.95	1.03	$(N_1)_{60}$	9.3 ± 2.0	9.4	9.50
CSRN		0.192	0.18				

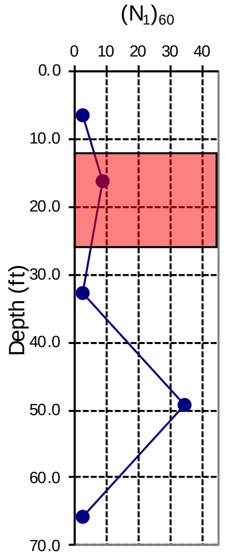

γ_m=110±3 pcf

γ_s=120±3 pcf

By Cakmak(1986)

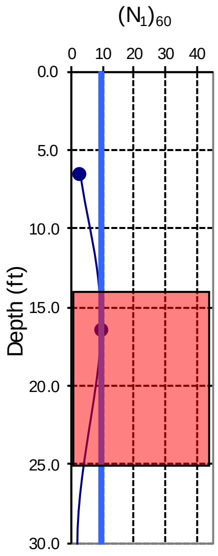

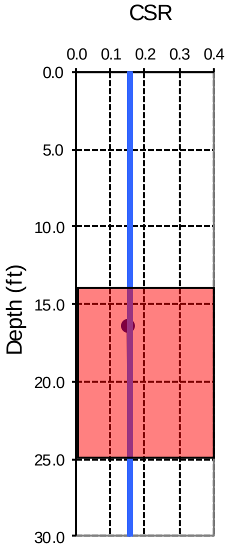

**Depth (m)**	**Depth (ft)**	**CSR**	**N**	**C_N**	**C_R**	**$(N1)_{60}$**
2.0	6.6	NA	2	1.71	0.80	2.7
5.0	16.4	0.151	9	1.12	0.92	***9.3***
10.0	32.8	0.156	3	0.90	1.00	2.7
15.0	49.2	0.147	45	0.77	1.00	34.7
20.0	65.6	0.149	4	0.68	1.00	2.7
					**Mean:**	9.3
					**St. Dev.**	2.0

assigned

**Case number:**	68
**Earthquake:**	1978 Miyagiken-Oki
**Magnitude:**	7.7 (Mw) USGS Centennial Earthquake Catalog
**Location:**	Shiomi 6
**References:**	Tsuchida et al (1979, 1980) Tohno et al. (1981)<br>Fear et al. (1995)
**Nature of Failure:**	Not specifically given but identified by Seed et al. (84) as "liquefied."
**Comments:**	Prior to the main shock of M=7.4, an earthquake of magnitude 6.7 occurred.<br>Only the Nakamura site liquefied during this earthquake.<br>The subject zone is seismically very active and had been shaken in 1897 and<br>1936 by earthquakes of Magnitudes 7.3 and 7.7<br>The original Tsuchida (1979) paper (in Japanese) could not be accessed.<br>However the same author's paper of 1980 in English was used to obtain<br>borelogs. Shiomi 6 site was claimed by Tokimatsu (1993) to be by Site D.<br>SPT energy was estimated as 65 % by Seed et al. (84)

Summary of Data

	Cetin et al. (2016)	Idriss&Boulanger (2010)	Seed et.al. (1984)		Cetin et al. (2016)	Idriss&Boulanger (2010)	Seed et.al. (1984)
Liquefied?	Yes	Yes	Yes	D_{50}	0.250 ± 0.050		0.25
Data Class	B			% Fines	10.0 ± 2.0	10.0	10
Critical Depth Range	9.8 - 19.7	13.1	13.0	% PI			
Depth to GWT (ft)	8.0 ± 0.3	7.9	8.0				
σ_v (psf)	1691.7 ± 199.4	1566.4	1560.0	N	7.3 ± 1.7	6.0	6.0
σ_v' (psf)	1269.6 ± 100.8	1253.1	1250.0	C_R	0.90	0.85	
a_{max} (g)	0.240 ± 0.072	0.24	0.240	C_S	1.00	1.00	
r_d	0.95 ± 0.064	0.98	0.970	C_B	1.00	1.00	
CSR	0.197 ± 0.061	0.190	0.190	C_E	1.09	1.09	1.090
Equivalent Magnitude	7.7	7.7		C_N	1.29	1.34	1.2
MSF		0.95	1.03	$(N_1)_{60}$	9.3 ± 2.2	7.5	8.00
CSRN		0.192	0.19				

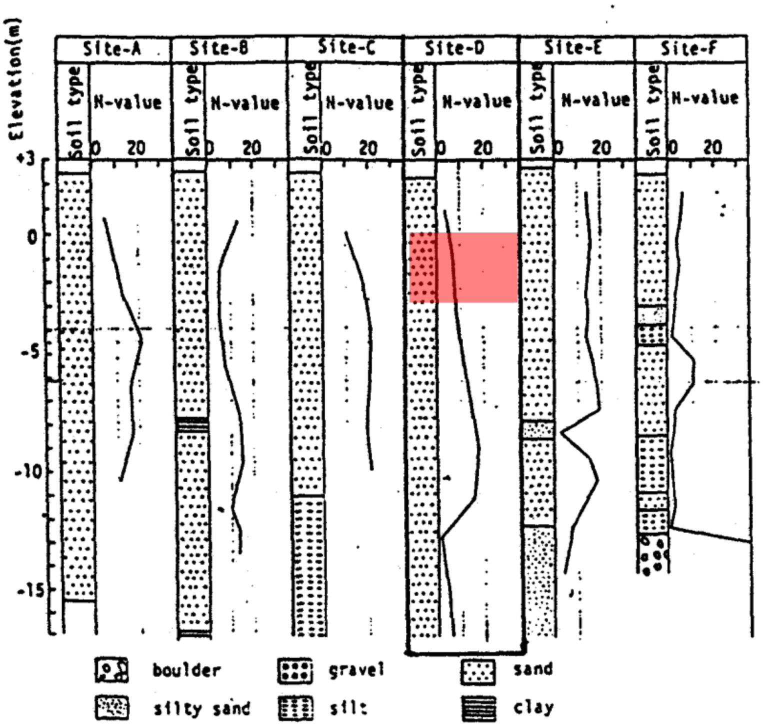

γ_m=110±3 pcf

γ_s=120±3 pcf

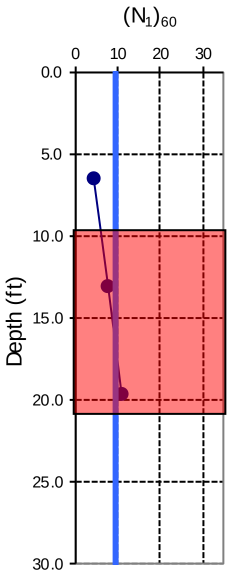

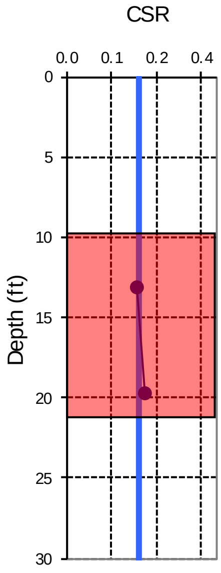

Depth (m)	Depth (ft)	CSR	N	C_N	C_R	$(N1)_{60}$
2.0	6.6	NA	3.0	1.71	0.80	4.5
4.0	13.1	0.190	6.0	1.34	0.89	***7.8***
6.0	19.7	0.210	9.0	1.17	0.95	***10.9***
					**Mean:**	9.3
					**St. Dev.**	2.2

**Case number:**	69
**Earthquake:**	1978 Miyagiken-Oki
**Magnitude:**	7.7 (Mw) USGS Centennial Earthquake Catalog
**Location:**	Yuriage Bridge 1
**References:**	Iwasaki et al. (1978) Tohno et al. (1981)<br>Fear et al. (1995)
**Nature of Failure:**	Sand boils out of surface cracks.<br>Damage to Yuriagegami Bridge, cracked piers, etc.
**Comments:**	Yuriage Br. 1 is located near pier 9 of the Yuriagegami Bridge, which crosses Natori River. Pier 9 is the closest to the right bank of the river.<br>PGA estimations of 0.24 and 0.3 g were used by Iwasaki et al (1978).<br>Based on cyclic triaxial test results, the sand layer at depth range of 2-6 m is predicted to be liquefied.<br>Tohno et al. (1981):<br>Prior to the main shock of M=7.4, an earthquake of magnitude 6.7 occurred.<br>The subject zone is seismically very active and had been shaken in 1897 and 1936 by earthquakes of Magnitudes 7.3 and 7.7<br>The dry river bed was cracked parallel to the river near Yuriage-Ohashi Bridge, and liquefied sands seeped out from the cracks. Some cracks were found near the piers of the Yuriage-Ohashi Birdge and the ground around the piers settled.<br>The boiled soil was a poorly graded coarse sand. Iwasaki and Tokida (1980) identified the liquefied layer as at depths of 2-5 m.<br>SPT values were taken after main earthquake (M=7.4)<br>SPT energy is estimated as 60 % by Seed et al. (84)

Summary of Data

	Cetin et al. (2016)	Idriss&Boulanger (2010)	Seed et.al. (1984)		Cetin et al. (2016)	Idriss&Boulanger (2010)	Seed et.al. (1984)
Liquefied?	Yes	Yes	Yes	D_{50}	0.400 ± 0.050		0.4
Data Class	B			% Fines	10.0 ± 2.0	10.0	10
Critical Depth Range	9.8 - 13.1	14.1	14.0	% PI			
Depth to GWT (ft)	5.6 ± 0.3	5.9	6.0				
σ_v (psf)	1233.1 ± 67.9	1670.8	1680.0	N	3.0 ± 1.3	4.0	4.0
σ_v' (psf)	866.6 ± 40.4	1169.6	1180.0	C_R	0.87	0.95	
a_{max} (g)	0.240 ± 0.072	0.24	0.240	C_S	1.00	1.00	
r_d	0.97 ± 0.051	0.97	0.970	C_B	1.00	1.00	
CSR	0.216 ± 0.066	0.216	0.215	C_E	1.00	1.00	1.000
Equivalent Magnitude	7.7	7.7		C_N	1.56	1.41	1.3
MSF		0.95	1.00	$(N_1)_{60}$	4.0 ± 1.8	5.4	5.00
CSRN		0.218	0.22				

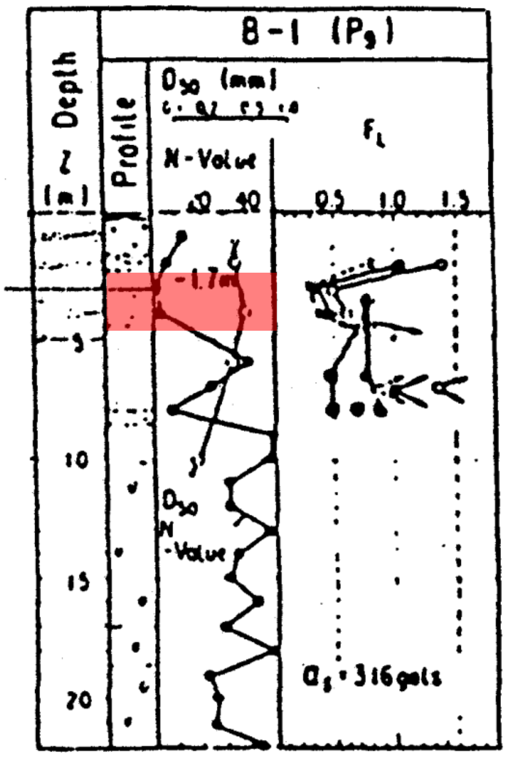

γ_m=100±3 pcf

γ_s=115±3 pcf

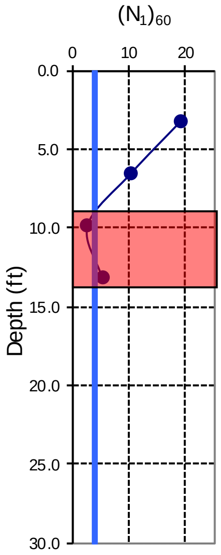

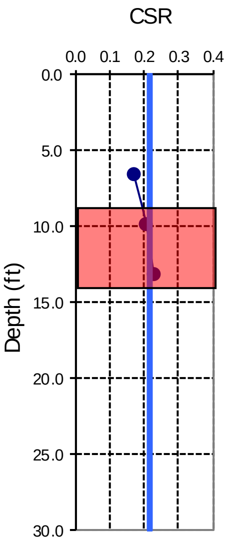

Depth (m)	Depth (ft)	CSR	N	C_N	C_R	$(N1)_{60}$	
1.0	3.3	NA	13	2.00	0.73	19.1	close to surf.
2.0	6.6	0.170	7	1.86	0.80	10.4	fines transition
3.0	9.8	0.206	2	1.64	0.85	***2.8***	
4.0	13.1	0.228	4	1.49	0.89	***5.3***	
					**Mean:**	4.0	
					**St. Dev.**	1.8	

**Case number:**	70
**Earthquake:**	1978 Miyagiken-Oki
**Magnitude:**	7.7 (Mw) USGS Centennial Earthquake Catalog
**Location:**	Yuriage Bridge 2
**References:**	Iwasaki et al. (1978) Tohno et al. (1981)<br>Fear et al. (1995)
**Nature of Failure:**	Sand boils out of surface cracks.<br>Damage to Yuriagegami Bridge, cracked piers, etc.
**Comments:**	Yuriage Br. 2 is located near pier 7 of the Yuriagegami Bridge, which crosses<br>Natori River. Pier 7 is towards the right bank of the river.<br>PGA estimations of 0.24 and 0.3 g were used by Iwasaki et al (1978).<br>Based on cyclic triaxial test results, the sand layer at depth range of 2-5 m is<br>predicted to be liquefied.<br>Tohno et al. (1981):<br>Prior to the main shock of M=7.4, an earthquake of magnitude 6.7 occurred.<br>The subject zone is seismically very active and had been shaken in 1897 and<br>1936 by earthquakes of Magnitudes 7.3 and 7.7<br>The dry river bed was cracked parallel to the river near Yuriage-Ohashi Bridge,<br>and liquefied sands seeped out from the cracks. Some cracks were found near<br>the piers of the Yuriage-Ohashi Birdge and the ground around the piers settled.<br>The boiled soil was a poorly graded coarse sand. Iwasaki and Tokida (1980)<br>identified the liquefied layer as at depths of 2-5 m.<br>Gravel percent exceeds 20 % by weight. (Seed et al, 84)<br>SPT values were taken after main earthquake (M=7.4)<br>SPT energy was estimated as 67 % by Seed et al. (84)

**Summary of Data**	Cetin et al. (2016)	Idriss&Boulanger (2010)	Seed et.al. (1984)		Cetin et al. (2016)	Idriss&Boulanger (2010)	Seed et.al. (1984)
Liquefied?	Yes	Yes	Yes	D_{50}	1.600 ± 0.050		1.6
Data Class	B			% Fines	7.0 ± 2.0	7.0	7
Critical Depth Range	6.0 - 10.0	8.2	11.0	% PI			
Depth to GWT (ft)	4.3 ± 0.3	3.9	4.0				
σ_v (psf)	893.4 ± 85.4	960.7	1320.0	N	11.4 ± 1.3	10.1	13.0
σ_v' (psf)	660.3 ± 46.4	710.1	880.0	C_R	0.82	0.85	
a_{max} (g)	0.240 ± 0.072	0.24	0.240	C_S	1.00	1.00	
r_d	1.00 ± 0.038	0.99	0.980	C_B	1.00	1.00	
CSR	0.210 ± 0.064	0.212	0.230	C_E	1.12	1.12	1.120
Equivalent Magnitude	7.7	7.7		C_N	1.79	1.68	1.5
MSF		0.95	1.02	$(N_1)_{60}$	18.7 ± 2.2	16.2	21.50
CSRN		0.203	0.23				

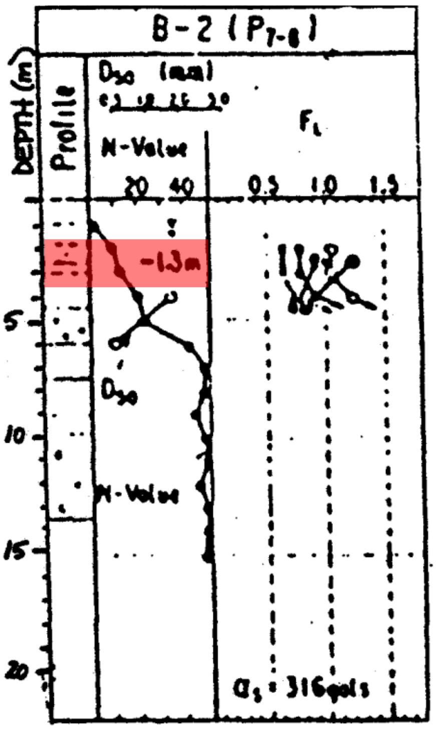

γ_m=100±3 pcf

γ_s=125±3 pcf

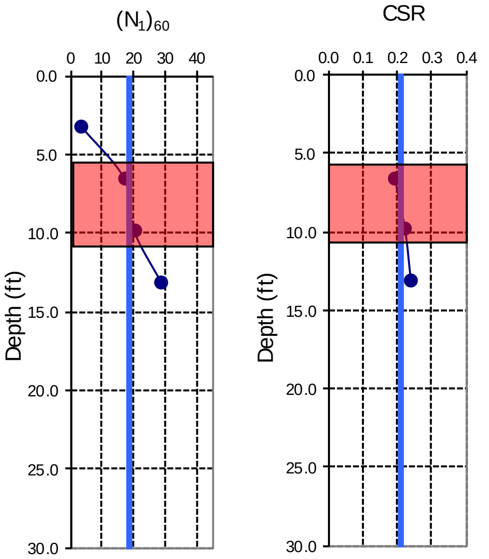

Depth (m)	Depth (ft)	CSR	N	C_N	C_R	$(N1)_{60}$	
1.0	3.3	NA	2	2.00	0.73	3.3	close to surf.
2.0	6.6	0.195	10	1.93	0.80	***17.2***	
3.0	9.8	0.225	13	1.65	0.85	***20.3***	
4.0	13.1	0.242	20	1.47	0.89	29.1	
					**Mean:**	18.7	
					**St. Dev.**	2.2	

**Case number:**	71
**Earthquake:**	1978 Miyagiken-Oki
**Magnitude:**	7.7 (Mw) USGS Centennial Earthquake Catalog
**Location:**	Yuriage Bridge 3
**References:**	Iwasaki et al. (1978) Tohno et al. (1981)<br>Fear et al. (1995)
**Nature of Failure:**	Sand boils out of surface cracks.<br>Damage to Yuriagegami Bridge, cracked piers, etc.
**Comments:**	Yuriage Br. 3 is located near pier 5 of the Yuriagegami Bridge, which crosses Natori River. Pier 5 is towards the center of the river.<br>PGA estimations of 0.24 and 0.3 g were used by Iwasaki et al (1978).<br>Based on cyclic triaxial test results, the sand layer at depth range of 1-2 m is predicted to be liquefied.<br>Tohno et al. (1981):<br>Prior to the main shock of M=7.4, an earthquake of magnitude 6.7 occurred.<br>The subject zone is seismically very active and had been shaken in 1897 and 1936 by earthquakes of Magnitudes 7.3 and 7.7<br>The dry river bed was cracked parallel to the river near Yuriage-Ohashi Bridge, and liquefied sands seeped out from the cracks. Some cracks were found near the piers of the Yuriage-Ohashi Birdge and the ground around the piers settled.<br>The boiled soil was a poorly graded coarse sand. Iwasaki and Tokida (1980) identified the liquefied layer as at depths of 2-5 m.<br>SPT values were taken after main earthquake (M=7.4)<br>SPT energy was estimated as 60 % by Seed et al. (84)

**Summary of Data**	Cetin et al. (2016)	Idriss&Boulanger (2010)	Seed et.al. (1984)		Cetin et al. (2016)	Idriss&Boulanger (2010)	Seed et.al. (1984)
Liquefied?	Yes	Yes	Yes	D_{50}	1.200 ± 0.050		1.2
Data Class	B			% Fines	12.0 ± 2.0	12.0	12
Critical Depth Range	6.6 - 13.1	14.1	14.0	% PI			
Depth to GWT (ft)	0.9 ± 0.3	1.0	1.0				
σ_v (psf)	1172.6 ± 134.0	1670.8	1680.0	N	7.1 ± 1.0	8.0	8.0
σ_v' (psf)	611.6 ± 70.3	877.2	870.0	C_R	0.85	0.95	
a_{max} (g)	0.240 ± 0.072	0.24	0.240	C_S	1.00	1.00	
r_d	0.99 ± 0.045	0.97	0.970	C_B	1.00	1.00	
CSR	0.295 ± 0.090	0.293	0.290	C_E	1.00	1.00	1.000
Equivalent Magnitude	7.7	7.7		C_N	1.86	1.56	1.5
MSF		0.95	1.00	$(N_1)_{60}$	11.2 ± 1.5	11.8	12.00
CSRN		0.282	0.29				

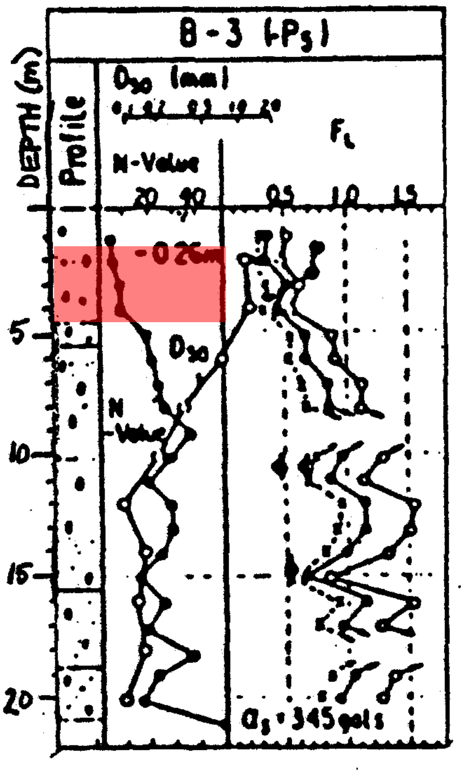

γ_m=110±3 pcf

γ_s=120±3 pcf

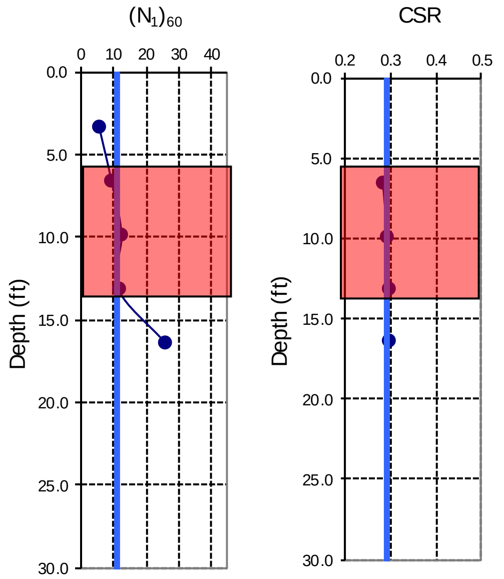

Depth (m)	Depth (ft)	CSR	N	C_N	C_R	$(N1)_{60}$	
1.0	3.3	0.256	4	2.00	0.73	5.9	close to surf.
2.0	6.6	0.285	6	2.00	0.80	***9.6***	
3.0	9.8	0.295	8	1.86	0.85	***12.6***	
4.0	13.1	0.298	8	1.63	0.89	***11.5***	
5.0	16.4	0.298	19	1.46	0.92	25.6	
					**Mean:**	11.2	
					**St. Dev.**	1.5	

**Case number:**	72
**Earthquake:**	1978 Miyagiken-Oki
**Magnitude:**	7.7 (Mw) USGS Centennial Earthquake Catalog
**Location:**	Yuriage Bridge 5
**References:**	Iwasaki et al. (1978) Tohno et al. (1981)<br>Fear et al. (1995)
**Nature of Failure:**	No surface evidence of liquefaction.
**Comments:**	Yuriage Br. 5 is located near pier 1 of the Yuriagegami Bridge, which crosses the Natori River. Pier 5 is the closest pier to the left bank of the river.<br>PGA estimations of 0.24 and 0.3 g were used by Iwasaki et al (1978).<br>Based on cyclic triaxial test results, the sand layer at depth range of 3-9 m is predicted to be liquefied. However no surface evindence of liquefaction exists.<br>Tohno et al. (1981):<br>Prior to the main shock of M=7.4, an earthquake of magnitude 6.7 occurred. The subject zone is seismically very active and had been shaken in 1897 and 1936 by earthquakes of Magnitudes 7.3 and 7.7<br>The dry river bed was cracked parallel to the river near Yuriage-Ohashi Bridge, and liquefied sands seeped out from the cracks. Some cracks were found near the piers of the Yuriage-Ohashi Birdge and the ground around the piers settled. The boiled soil was a poorly graded coarse sand. Iwasaki and Tokida (1980) identified the potentially liquefiable layer at depths of 2-5 m.<br>SPT values were taken after main earthquake (M=7.4)<br>SPT energy is estimated as 67 % by Seed et al. (84)

Summary of Data

	Cetin et al. (2016)	Idriss&Boulanger (2010)	Seed et.al. (1984)		Cetin et al. (2016)	Idriss&Boulanger (2010)	Seed et.al. (1984)
Liquefied?	No	No	No	D_{50}	0.350 ± 0.050		0.35
Data Class	B			% Fines	17.0 ± 2.0	17.0	17
Critical Depth Range	19.7 - 29.5	24.0	24.0	% PI			
Depth to GWT (ft)	4.3 ± 0.5	3.9	4.0				
σ_v (psf)	3054.5 ± 214.3	2882.2	2880.0	N	20.5 ± 6.7	17.0	17.0
σ_v' (psf)	1785.2 ± 123.5	1629.1	1630.0	C_R	0.99	0.95	
a_{max} (g)	0.240 ± 0.072	0.24	0.240	C_S	1.00	1.00	
r_d	0.98 ± 0.099	0.94	0.940	C_B	1.00	1.00	
CSR	0.261 ± 0.083	0.260	0.260	C_E	1.12	1.12	1.120
Equivalent Magnitude	7.7	7.7		C_N	1.09	1.11	1.1
MSF		0.95	1.02	$(N_1)_{60}$	24.6 ± 8.0	20.1	21.00
CSRN		0.263	0.26				

γ_m=120±3 pcf

γ_s=125±3 pcf

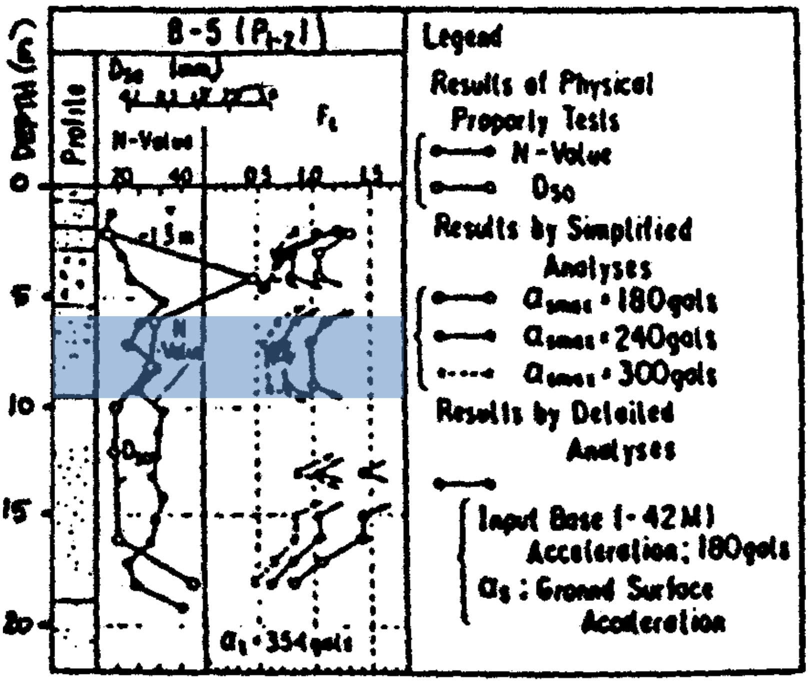

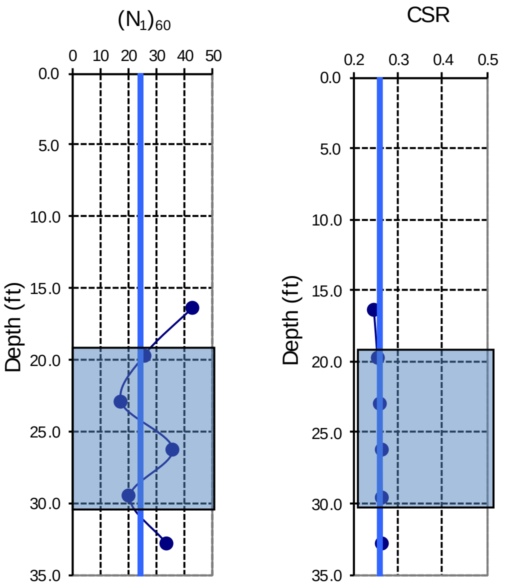

Depth (m)	Depth (ft)	CSR	N	C_N	C_R	$(N1)_{60}$
5.0	16.4	0.247	32	1.29	0.92	42.5
6.0	19.7	0.254	20	1.20	0.95	***25.4***
7.0	23.0	0.259	14	1.12	0.98	***17.1***
8.0	26.2	0.262	30	1.06	1.00	***35.5***
9.0	29.5	0.263	18	1.01	1.00	***20.2***
10.0	32.8	0.263	31	0.96	1.00	33.2
					**Mean:**	24.6
					**St. Dev.**	8.0

**Case number:**	73
**Earthquake:**	1978 Miyagiken-Oki
**Magnitude:**	7.7 (Mw) USGS Centennial Earthquake Catalog
**Location:**	Yuriagekami-1
**References:**	Iwasaki et al. (1978) Tohno et al. (1981)<br>Fear et al. (1995)
**Nature of Failure:**	Sand volcanoes, cracking and spreading of the ground towards the pond, also damage to the bank of the dike.
**Comments:**	Yuriagekami is located in between the edge of the Shin-enko numa pond just just inland from the Natori rive, and the base of the dyke on the right bank of the river.<br>PGA is estimated as 0.18 g by Iwasaki et al (1978). Based on cyclic triaxial test results, the liquefied layer is predicted to be the sand layer at a depth of 2-8 m.<br>Tohno et al. (1981)<br>Prior to the main shock of M=7.4, an earthquake of magnitude 6.7 occurred. The subject zone is seismically very active and had been shaken in 1897 and 1936 by earthquakes of Magnitudes 7.3 and 7.7.<br>The river embankment was partially collpased and cracked on both sides at Yuriagekami.<br>SPT values were taken after main earthquake (M=7.4)<br>SPT energy is estimated as 60 % by Seed et al. (84)

**Summary of Data**	Cetin et al. (2016)	Idriss&Boulanger (2010)	Seed et.al. (1984)		Cetin et al. (2016)	Idriss&Boulanger (2010)	Seed et.al. (1984)
Liquefied?	Yes	Yes	Yes	D_{50}	0.040 ± 0.050		0.04
Data Class	B			% Fines	60.0 ± 2.0	60.0	60
Critical Depth Range	5.9 - 18.0	18.0	18.0	% PI			
Depth to GWT (ft)	6.0 ± 0.3	5.9	6.0				
σ_v (psf)	1257.5 ± 224.0	2067.7	2060.0	N	2.0 ± 0.9	2.0	2.0
σ_v' (psf)	882.9 ± 100.8	1315.8	1310.0	C_R	0.87	0.95	
a_{max} (g)	0.240 ± 0.072	0.24	0.240	C_S	1.00	1.00	
r_d	0.98 ± 0.053	0.96	0.960	C_B	1.00	1.00	
CSR	0.217 ± 0.068	0.236	0.235	C_E	1.00	1.00	1.000
Equivalent Magnitude	7.7	7.7		C_N	1.55	1.31	1.2
MSF		0.95	1.02	$(N_1)_{60}$	2.7 ± 1.2	2.5	2.50
CSRN		0.239	0.23				

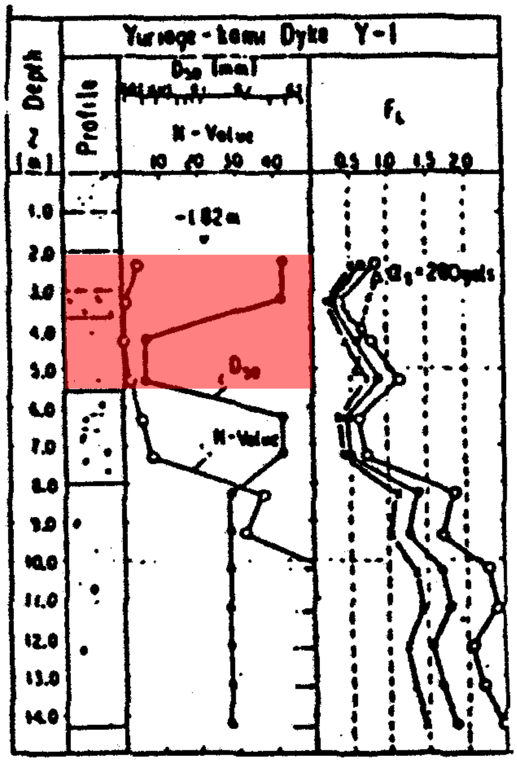

γ_m=100±3 pcf

γ_s=110±3 pcf

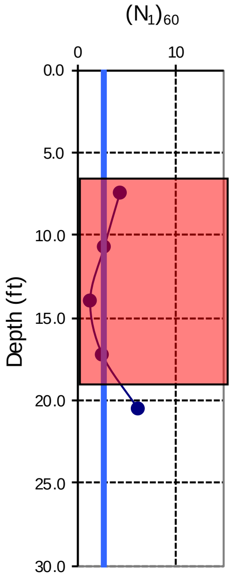

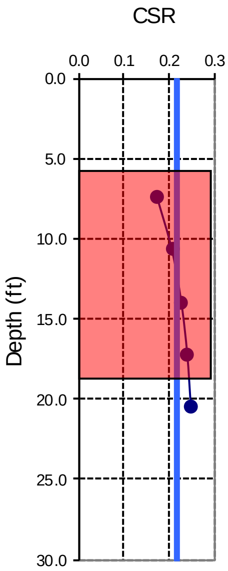

Depth (m)	Depth (ft)	CSR	N	C_N	C_R	$(N1)_{60}$
2.3	7.4	0.175	3	1.78	0.81	***4.3***
3.3	10.7	0.208	2	1.61	0.86	***2.8***
4.3	13.9	0.229	1	1.47	0.90	***1.3***
5.3	17.2	0.242	2	1.37	0.93	***2.5***
6.3	20.5	0.250	5	1.28	0.96	6.1
					**Mean:**	2.7
					**St. Dev.**	1.2

**Case number:**	74
**Earthquake:**	1978 Miyagiken-Oki
**Magnitude:**	7.7 (Mw) USGS Centennial Earthquake Catalog
**Location:**	Yuriagekami-2
**References:**	Iwasaki et al. (1978) Tohno et al. (1981)<br>Fear et al. (1995)
**Nature of Failure:**	Sand boils.
**Comments:**	Yuriagekami-2 is located by the edge of the Natori river, on the right bank of the river.<br>PGA is estimated as 0.18 g by Iwasaki et al (1978). Based on cyclic triaxial test results, the liquefied layer is peridicted to be the sand layer at depth of 1-6 m.<br>Tohno et al. (1981):<br>Prior to the main shock of M=7.4, an earthquake of magnitude 6.7 occurred.<br>The subject zone is seismically very active and had been shaken in 1897 and 1936 by earthquakes of Magnitudes 7.3 and 7.7.<br>The river embankment was partially collapsed and cracked on both sides at Yuriagekami.<br>Cracks were observed both on and at the base of the dike. Also sand volcanoes were found along the dike.<br>SPT values were taken after main earthquake (M=7.4)<br>SPT energy was estimated as 60 % by Seed et al. (84)

**Summary of Data**	Cetin et al. (2016)	Idriss&Boulanger (2010)	Seed et.al. (1984)		Cetin et al. (2016)	Idriss&Boulanger (2010)	Seed et.al. (1984)
Liquefied?	Yes	Yes	Yes	D_{50}	0.400 ± 0.050		0.4
Data Class	B			% Fines	0.0 ± 2.0	0.0	0
Critical Depth Range	6.6 - 18.0	14.1	14.0	% PI			
Depth to GWT (ft)	2.8 ± 0.3	3.0	3.0				
σ_v (psf)	1420.6 ± 231.7	1670.8	1680.0	N	8.8 ± 3.3	11.0	11.0
σ_v' (psf)	826.9 ± 114.9	981.6	990.0	C_R	0.88	0.95	
a_{max} (g)	0.240 ± 0.072	0.24	0.240	C_S	1.00	1.00	
r_d	0.97 ± 0.055	0.97	0.970	C_B	1.00	1.00	
CSR	0.260 ± 0.080	0.258	0.255	C_E	1.00	1.00	1.000
Equivalent Magnitude	7.7	7.7		C_N	1.60	1.45	1.4
MSF		0.95	1.02	$(N_1)_{60}$	12.3 ± 4.7	15.1	15.00
CSRN		0.251	0.25				

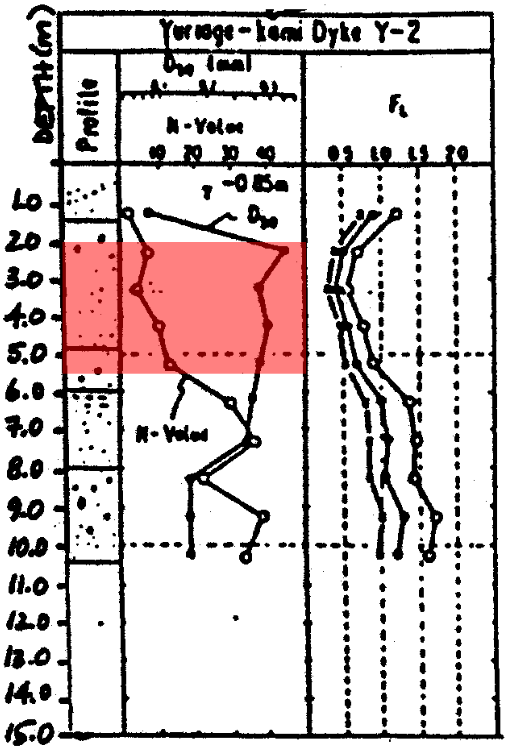

γ_m=100±3 pcf

γ_s=120±3 pcf

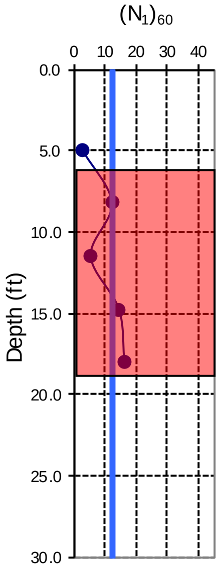

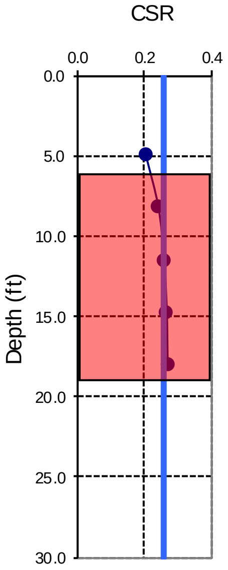

Depth (m)	Depth (ft)	CSR	N	C_N	C_R	$(N1)_{60}$	
1.5	4.9	0.206	2	2.00	0.77	3.1	close to surf.
2.5	8.2	0.241	8	1.89	0.82	***12.5***	
3.5	11.5	0.257	4	1.65	0.87	***5.7***	
4.5	14.8	0.265	11	1.48	0.90	***14.7***	
5.5	18.0	0.267	13	1.35	0.94	***16.5***	
					**Mean:**	12.3	
					**St. Dev.**	4.7	

**Case number:**	75
**Earthquake:**	1978 Miyagiken-Oki
**Magnitude:**	7.7 (Mw) USGS Centennial Earthquake Catalog
**Location:**	Yuriagekami-3
**References:**	Iwasaki et al. (1978) Tohno et al. (1981)<br>Fear et al. (1995)
**Nature of Failure:**	No surface evidence of liquefaction.
**Comments:**	Yuriagekami-3 is located at the base of the dike on the right bank of the Natori River, not far from the Shin-enko numa pond.<br>PGA is estimated as 0.18 g by Iwasaki et al (1978).<br>Tohno et al. (1981):<br>Prior to the main shock of M=7.4, an earthquake of magnitude 6.7 occurred.<br>The subject zone is seismically very active and had been shaken in 1897 and 1936 by earthquakes of Magnitudes 7.3 and 7.7<br>The river embankment was partially collapsed and cracked on both sides at Yuriagekami.<br>Cracks were observed both on and at the base of the dike. Also sand volcanoes were found along the dike.<br>SPT values were taken after main earthquake (M=7.4)<br>SPT energy was estimated as 67 % by Seed et al. (84)

**Summary of Data**	Cetin et al. (2016)	Idriss&Boulanger (2010)	Seed et.al. (1984)		Cetin et al. (2016)	Idriss&Boulanger (2010)	Seed et.al. (1984)
Liquefied?	No	No	No	D_{50}	0.600 ± 0.050		0.6
Data Class	B			% Fines	0.0 ± 2.0	0.0	0
Critical Depth Range	14.8 - 24.6	18.0	18.0	% PI			
Depth to GWT (ft)	7.1 ± 0.3	6.9	7.0				
σ_v (psf)	2354.8 ± 209.6	2151.2	2160.0	N	21.1 ± 1.9	20.0	20.0
σ_v' (psf)	1566.6 ± 112.4	1462.0	1470.0	C_R	0.95	0.95	
a_{max} (g)	0.240 ± 0.072	0.24	0.240	C_S	1.00	1.00	
r_d	0.97 ± 0.082	0.96	0.960	C_B	1.00	1.00	
CSR	0.227 ± 0.071	0.220	0.220	C_E	1.12	1.12	1.120
Equivalent Magnitude	7.7	7.7		C_N	1.16	1.16	1.2
MSF		0.95	1.02	$(N_1)_{60}$	26.0 ± 2.3	24.6	26.00
CSRN		0.220	0.22				

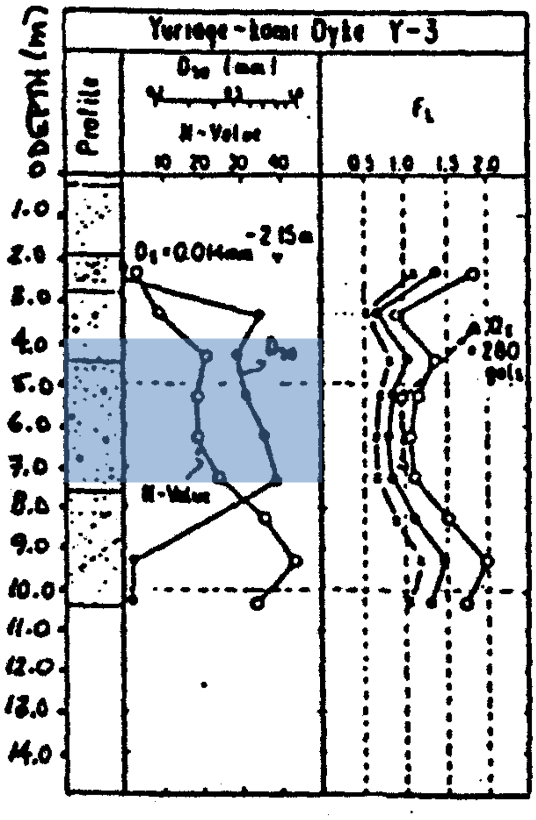

γ_m=110±3 pcf

γ_s=125±3 pcf

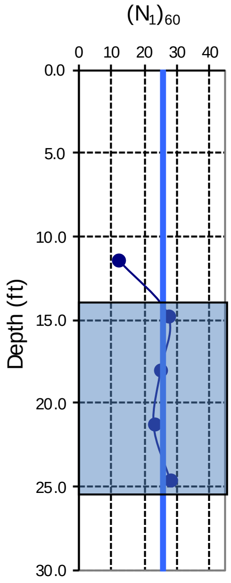

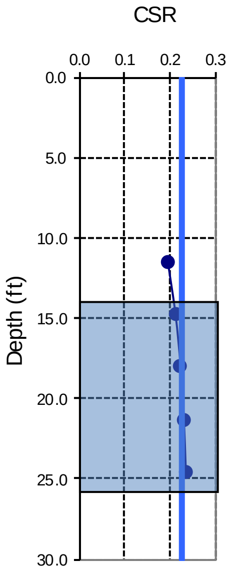

Depth (m)	Depth (ft)	CSR	N	C_N	C_R	$(N1)_{60}$	
3.5	11.5	0.195	9	1.42	0.87	12.4	clay?
4.5	14.8	0.212	21	1.30	0.90	***27.5***	
5.5	18.0	0.223	20	1.20	0.94	***25.1***	
6.5	21.3	0.231	19	1.13	0.96	***23.0***	
7.5	24.6	0.235	24	1.06	0.99	***28.2***	
					**Mean:**	26.0	
					**St. Dev.**	2.3	

**Case number:**	76
**Earthquake:**	1979 Imperial Valley
**Magnitude:**	6.53 (Mw) Pacific Earthquake Engineering Research Center: NGA Database
**Location:**	Heber Road A1
**References:**	Youd and Bennett (1983), "Liquefaction sites, Imperial Valley, CA", Benett et. al. (1979)
**Nature of Failure:**	No surface manifestation of liquefaction
**Comments:**	The Imperial Valley lies in the southern part of the Salton Basin, which was formed by northward extension of the crustal rifting that opened the Gulf of California. The basin is underlain by as much as 20,000 ft of Tertiary and Quaternary marine, continental, and lacustrine sediment. Holocene sediment is as thick as 330 ft.<br>The principal rivers are the Alamo and New Rivers along which many sand boils were developed on fluvial deposits.<br>Heber Road site is ~1.6 km to the fault rupture plane.<br>AGR and BCR are two stations that are located ~ 7 kms southeast of Heber Road site. The peak acceleration recorded at station AGR at an epicentral distance of 1.8 km is 0.35 and 0.23 g. The station is underlain by deep alluvium and the motion is free field. At Bonds Corner, 5.2 km southern terminus of the fault rupture and 6 km form the southeast of the Heber Road site, PGA was recorded as 0.76 and 0.58 g. Seed et al (1984) used a PGA value of 0.78 g.<br>It is believed that directivity effects play a major role in the intensity of shaking.<br>Higher intensity shakings (0.83 g) were recorded at the northern section of the fault.<br>Based on the recorded motion and earthquake specific PGA contour maps, PGA was estimated as in the range of 0.4-0.7 g.<br>Site response analyses based PGA estimation was in the range of ~ 0.45 to 0.50<br>ASTM D1586-67 procedures were followed for SPT tests. 10" outside diameter hollow-stem auger was used with Safe-T- Driver and safety hammer for the drillings in 1979 and 1981. Donut Hammer with rope cathead was used for the drillings in in 1982. Energy ratios are adopted as 68 and 66 % for safety and donut hammers respectively.

Summary of Data

	Cetin et al. (2016)	Idriss&Boulanger (2010)	Seed et.al. (1984)		Cetin et al. (2016)	Idriss&Boulanger (2010)	Seed et.al. (1984)
Liquefied?	No	No		D_{50}	0.111 ± 0.007		0.112
Data Class	A			% Fines	13.0 ± 3.9	12.0	25
Critical Depth Range	5.9 - 16.4	9.5	12.0	% PI			
Depth to GWT (ft)	5.9	5.9	6.0				
σ_v (psf)	1246.7 ± 220.1	1106.9	1410.0	N	32.9 ± 2.6	30.4	28.0
σ_v' (psf)	919.2 ± 112.6	877.2	1035.0	C_R	0.82	0.85	↑
a_{max} (g)	0.470 ± 0.071	0.78	0.780	C_S	1.00	1.00	
r_d	0.82 ± 0.010	0.97	0.970	C_B	1.00	1.00	1.050
CSR	0.340 ± 0.055	0.620	0.670	C_E	1.13	1.13	↓
Equivalent Magnitude	6.53	6.53		C_N	1.52	1.30	1.4
MSF		1.29	1.17	$(N_1)_{60}$	46.5 ± 3.7	37.8	39.50
CSRN		0.437	0.58				

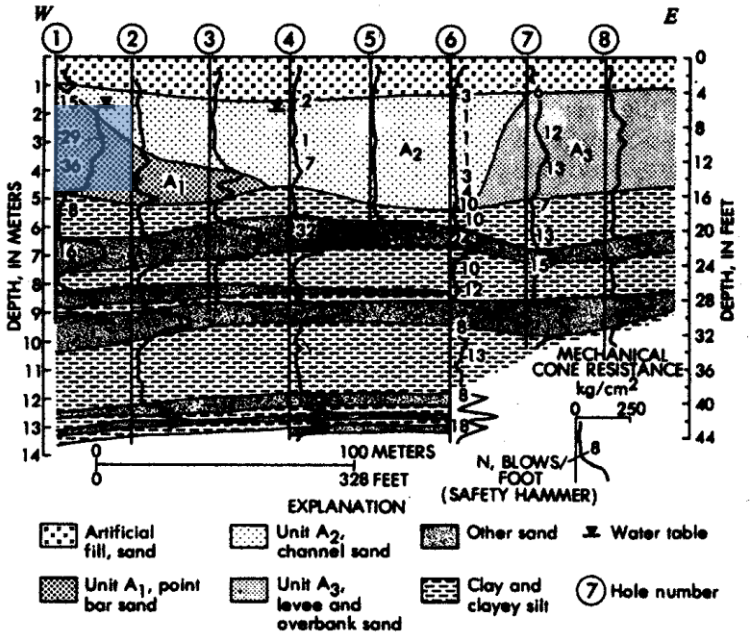

γ_m=100±3 pcf

γ_s=125±3 pcf

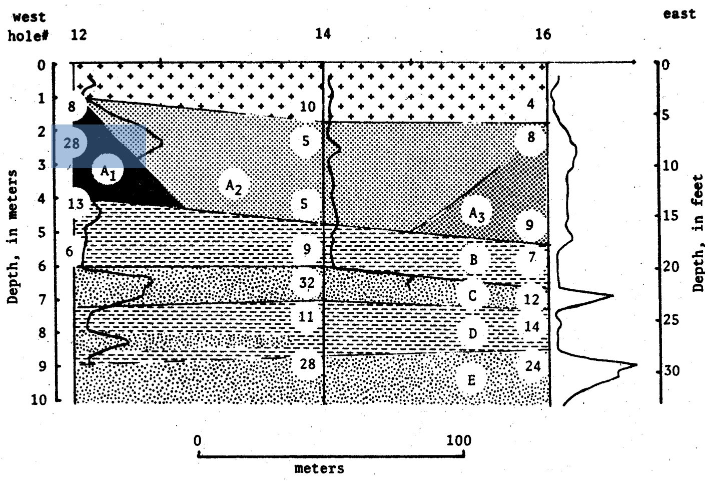

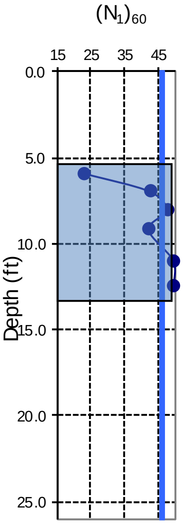

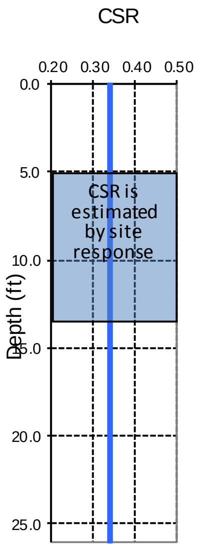

Depth (m)	Depth (ft)	CSR	N	C_N	C_R	$(N1)_{60}$	
1.8	5.9		15	1.89	0.73	23.4	BH-1
2.12	7.0		28	1.80	0.75	***42.7***	BH-12
2.45	8.0		32	1.71	0.77	***47.8***	BH-1 Donut
2.8	9.2		29	1.63	0.79	***42.4***	BH-1
3.35	11.0		35	1.53	0.82	***49.7***	BH-1 Donut
3.8	12.5		36	1.45	0.84	***49.9***	BH-1
					**Mean:**	46.5	
					**St. Dev.**	3.7	

Case number: 77

Earthquake: 1979 Imperial Valley

Magnitude: 6.53 (Mw) Pacific Earthquake Engineering Research Center: NGA Database

Location: Heber Road A2

References: Youd and Bennett (1983), "Liquefaction sites, Imperial Valley, CA", Benett et. al. (1979)

Nature of Failure: Liquefaction-induced lateral spreading shifted the road as much as 7 ft.
southward toward a 6.6 ft deep depression. Ground cracks and scaprs formed.
Sand boils erupted at several placed along the spread.

Comments: The Imperial Valley lies in the southern part of the Salton Basin, which was formed by northward extension of the crustal rifting that opened the Gulf of California. The basin is underlain by as much as 20,000 ft of Tertiary and Quaternary marine, continental, and lacustrine sediment. Holocene sediment is as thick as 330 ft.
The principal rivers are the Alamo and New Rivers along which many sand boils were developed on fluvial deposits.
Heber Road site is ~1.6 km to the fault rupture plane.
AGR and BCR are two stations that are located ~ 7 kms southeast of Heber Road site. The peak acceleration recorded at station AGR at an epicentral distance of 1.8 km is 0.35 and 0.23 g. The station is underlain by deep alluvium and the motion is free field. At Bonds Corner, 5.2 km southern terminus of the fault rupture and 6 km form the southeast of the Heber Road site, PGA was recorded as 0.76 and 0.58 g. Seed et al (1984) used a PGA value of 0.78 g.
It is believed that directivity effects play a major role in the intensity of shaking.
Higher intensity shakings (0.83 g) were recorded at the northern section of the fault.
Based on the recorded motion and earthquake specific PGA contour maps, PGA was estimated as in the range of 0.4-0.7 g.
Site response analyses based PGA estimation was in the range of ~ 0.45 to 0.50
ASTM D1586-67 procedures were followed for SPT tests. 10" outside diameter hollow-stem auger was used with Safe-T- Driver and safety hammer for the drillings in 1979 and 1981. Donut Hammer with rope cathead was used for the drillings in in 1982. Energy ratios are adopted as 68 and 66 % for safety and donut hammers respectively.

Summary of Data

	Cetin et al. (2016)	Idriss&Boulanger (2010)	Seed et.al. (1984)		Cetin et al. (2016)	Idriss&Boulanger (2010)	Seed et.al. (1984)
Liquefied?	Yes	Yes	Yes	D_{50}	0.116 ± 0.013		0.119
Data Class	A			% Fines	20.9 ± 5.0	18.0	29
Critical Depth Range	6.0 - 15.1	12.1	12.0	% PI			
Depth to GWT (ft)	5.9	5.9	6.0				
σ_v (psf)	1103.0 ± 167.6	1420.2	1410.0	N	2.4 ± 1.5	2.0	1.0
σ_v' (psf)	812.3 ± 76.9	1044.3	1035.0	C_R	0.81	0.85	
a_{max} (g)	0.470 ± 0.071	0.78	0.780	C_S	1.00	1.00	
r_d	0.78 ± 0.020	0.96	0.970	C_B	1.00	1.00	1.050
CSR	0.324 ± 0.054	0.661	0.670	C_E	1.13	1.13	
Equivalent Magnitude	6.53	6.53		C_N	1.61	1.51	1.4
MSF		1.29	1.17	$(N_1)_{60}$	3.5 ± 2.2	2.9	1.50
CSRN		0.484	0.58				

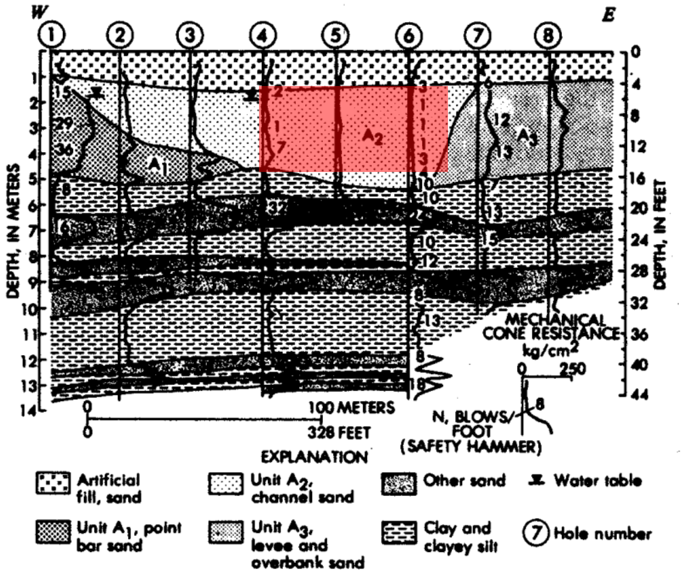

γ_m=100±3 pcf

γ_s=110±3 pcf

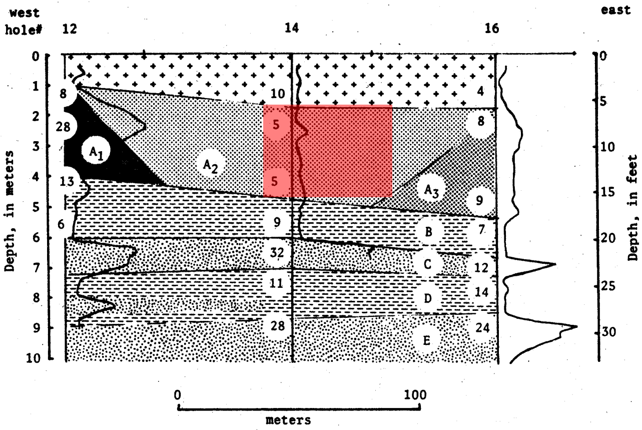

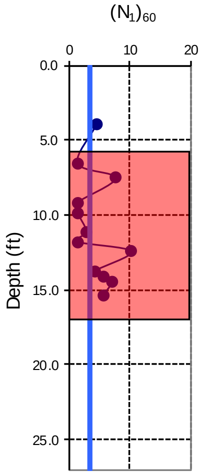

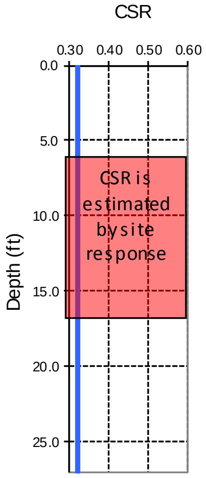

Depth (m)	Depth (ft)	CSR	N	C_N	C_R	$(N1)_{60}$	
1.2	3.9		3	2.00	0.68	4.6	BH-6
2	6.6		1	1.84	0.74	***1.6***	BH-6
2.3	7.5		5	1.78	0.76	7.7	BH-14
2.8	9.2		1	1.68	0.79	***1.5***	BH-6
3	9.8		1	1.65	0.80	***1.5***	BH-4
3.4	11.2		2	1.59	0.82	***3.0***	BH-5 Donut
3.6	11.8		1	1.56	0.83	***1.5***	BH-6
3.8	12.5		7	1.53	0.84	10.2	BH-4
4.2	13.8		3	1.48	0.86	***4.3***	BH-6
4.3	14.1		4	1.47	0.86	***5.7***	BH-5 Donut
4.4	14.4		5	1.46	0.87	***7.2***	BH-14
4.7	15.4		4	1.42	0.88	***5.7***	BH-6
					**Mean:**	3.5	
					**St. Dev.**	2.2	

**Case number:**	78
**Earthquake:**	Imperial Valley
**Magnitude:**	6.53 (Mw) Pacific Earthquake Engineering Research Center: NGA Database
**Location:**	Heber Road A3
**References:**	Youd and Bennett (1983), "Liquefaction sites, Imperial Valley, CA"
**Nature of Failure:**	No surface manifestation of liquefaction
**Comments:**	The Imperial Valley lies in the southern part of the Salton Basin, which was formed by northward extension of the crustal rifting that opened the Gulf of California. The basin is underlain by as much as 20,000 ft of Tertiary and Quaternary marine, continental, and lacustrine sediment. Holocene sediment is as thick as 330 ft.<br>The principal rivers are the Alamo and New Rivers along which many sand boils were developed on fluvial deposits.<br>Heber Road site is 1.6 km to the fault rupture plane.<br>AGR and BCR are two stations that are located ~ 7 kms southeast of Heber Road site. The peak acceleration recorded at station AGR at an epicentral distance of 1.8 km is 0.35 and 0.23 g. The station is underlain by deep alluvium and the motion is free field. At Bonds Corner, 5.2 km southern terminus of the fault rupture and 6 km form the southeast of the Heber Road site, PGA was recorded as 0.76 and 0.58 g. Seed et al (1984) used a PGA value of 0.78 g.<br>It is believed that directivity effects play a major role in the intensity of shaking.<br>Higher intensity shakings (0.83 g) were recorded at the northern section of the fault.<br>Based on the recorded motion and earthquake specific PGA contour maps, PGA was estimated as in the range of 0.4-0.7 g.<br>Site response analyses based PGA estimation was in the range of ~ 0.45 to 0.50<br>ASTM D1586-67 procedures were followed for SPT tests. 10" outside diameter hollow-stem auger was used with Safe-T- Driver and safety hammer for the drillings in 1979 and 1981. Donut Hammer with rope cathead was used for the drillings in in 1982. Energy ratios are adopted as 68 and 66 % for safety and donut hammers respectively.

Summary of Data

	Cetin et al. (2016)	Idriss&Boulanger (2010)	Seed et.al. (1984)		Cetin et al. (2016)	Idriss&Boulanger (2010)	Seed et.al. (1984)
Liquefied?	No	No	No	D_{50}	0.094 ± 0.004		0.094
Data Class	A			% Fines	25.3 ± 5.2	25.0	37
Critical Depth Range	5.9 - 16.1	13.1	14.0	% PI			
Depth to GWT (ft)	5.9	5.9	6.0				
σ_v (psf)	1200.8 ± 204.8	1649.9	1660.0	N	13.0 ± 4.2	13.0	13.0
σ_v' (psf)	883.5 ± 101.2	1169.58	1160.0	C_R	0.82	0.85	↑
a_{max} (g)	0.470 ± 0.071	0.78	0.780	C_S	1.00	1.00	
r_d	0.75 ± 0.025	0.950	0.960	C_B	1.00	1.00	1.050
CSR	0.311 ± 0.052	0.69	0.695	C_E	1.13	1.13	↓
Equivalent Magnitude	6.53	6.53		C_N	1.55	1.29	1.3
MSF		1.290	1.17	$(N_1)_{60}$	18.6 ± 6.0	16.2	17.50
CSRN			0.60				

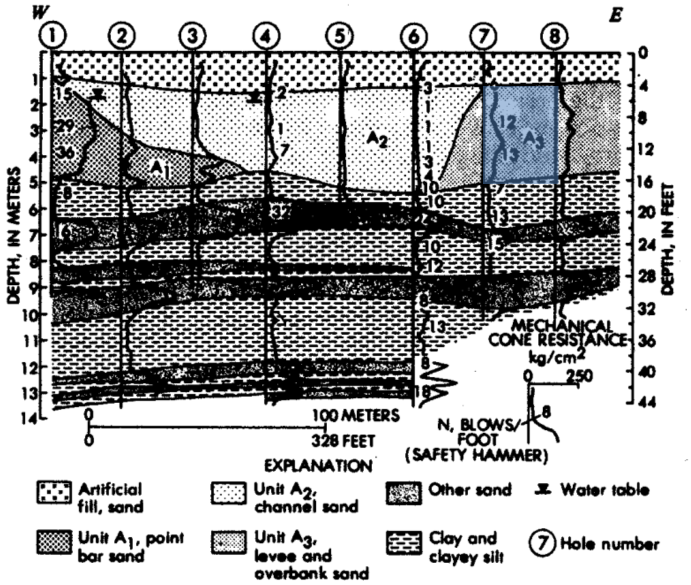

γ_m=100±3 pcf

γ_s=120±3 pcf

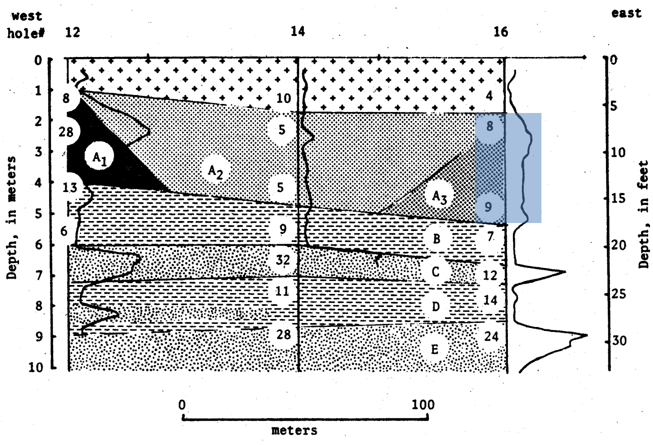

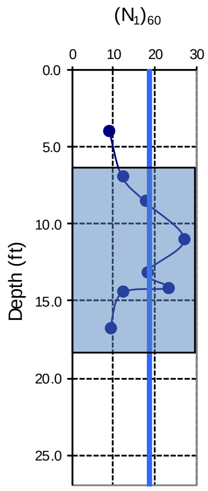

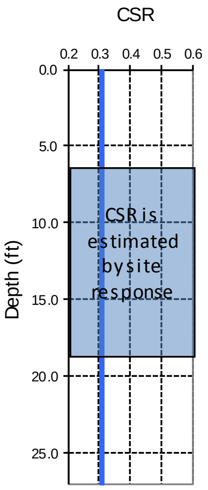

Depth (m)	Depth (ft)	CSR	N	C_N	C_R	$(N1)_{60}$	
1.2	3.9		6	2.00	0.68	9.2	BH-7
2.1	6.9		8	1.81	0.75	***12.3***	BH-16
2.6	8.5		12	1.69	0.78	***17.9***	BH-7
3.35	11.0		19	1.55	0.82	***27.4***	BH-8
4	13.1		13	1.45	0.85	***18.2***	BH-7
4.3	14.1		17	1.41	0.86	***23.5***	BH-8
4.4	14.4		9	1.40	0.87	***12.4***	BH-16
5.1	16.7		7	1.32	0.89	9.4	BH-7
					**Mean:**	18.6	
					**St. Dev.**	6.0	

Case number: 79

Earthquake: 1979 Imperial Valley Earthquake

Magnitude: 6.53 (Mw) Pacific Earthquake Engineering Research Center: NGA Database

Location: Kornbloom B

References: Bennett et al. (1984) and Fear et al. (1995)
James Gerard Bierschwale. (1984)
Youd, T.L., Wieczorek G. F., (1984), "Liquefaction during the 1981 and
Previous Earthquakes Near Westmorland, CA" Open File Rep. 84-680

Nature of Failure: No surface evidence of liquefaction

Comments:

The Imperial Valley lies in the southern part of the Salton Basin, which was formed by northward extension of the crustal rifting that opened the Gulf of California. The basin is underlain by as much ad 20,000 ft of Tertiary and Quaternary marine, continental, and lacustrine sediment. Holocene sediment is as thick as 330 ft.
The principal rivers are the Alamo and New Rivers along which many sand boils were developed on fluvial deposits.
A: silty clay, stiff-lacustrine and/or flood plain deposit
B: silty and sandy silt-fluvial and perhaps fluvial deltaic deposition
C: silty clay to clay, stiff-lacustrine and/or flood plain deposit
D: interbedded silt and sand-loose to medium dense
E: Uniform sand with some silt-medium dense to dense
F: interbedded silt and sand - loose to medium dense.
D-F :fluvial and perhaps fluvial-deltaic deposition
PGA was recorded at the ground level of a two-story building as 0.13 g (CAL) which is ~23 km from the north terminus of the fault and ~16 km north of the north terminus of the fault, PGA is recorded as 0.11 g.(Westmorland). The site is 25 km north of the north terminus of the fault and ~9 km from the westmorland seismographic station. PGA was estimated as 0.12 g by Andrus and Stoke (NCEER, 97). Due to rapid dissipation of wave energy beyond the north terminus of the fault, PGA may be lower than what is recorded at Westmorland. Based on earthquake specific attenuation relationship, PGA was estimated as 0.06-0.1 g. A value of 0.13 is adopted for the study based on site response analysis results.
Energy ratio ~68 %

Summary of Data

	Cetin et al. (2016)	Idriss&Boulanger (2010)	Seed et.al. (1984)		Cetin et al. (2016)	Idriss&Boulanger (2010)	Seed et.al. (1984)
Liquefied?	No	No		D_{50}	0.045 ± 0.008		0.045
Data Class	A			% Fines	83.0 ± 9.6	92.0	92
Critical Depth Range	9.0 - 17.5	14.1	14.0	% PI			
Depth to GWT (ft)	9.0	8.9	9.0				
σ_v (psf)	1410.0 ± 172.7	1608.2	1615.0	N	5.4 ± 3.1	5.0	5.0
σ_v' (psf)	1144.8 ± 88.1	1294.9	1305.0	C_R	0.85	0.85	
a_{max} (g)	0.130 ± 0.020	0.13		C_S	1.00	1.00	
r_d	0.83 ± 0.030	0.95	0.970	C_B	1.00	1.00	1.050
CSR	0.086 ± 0.014	0.099		C_E	1.13	1.13	
Equivalent Magnitude	6.53	6.53		C_N	1.36	1.29	1.2
MSF		1.29		$(N_1)_{60}$	7.1 ± 4.1	6.2	6.50
CSRN		0.073					

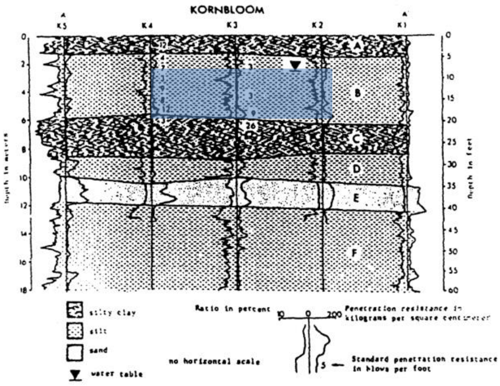

γ_m=100±3 pcf

γ_s=120±3 pcf

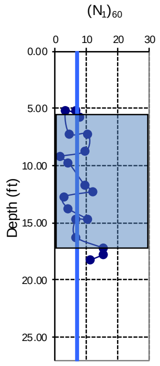

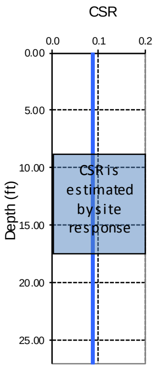

Depth (m)	Depth (ft)	CSR	N	C_N	C_R	$(N1)_{60}$		FC	sand	silt	clay	D50	WN	WL	PI	LI
1.60	5.25		4	2.00	0.71	6.5	K-4	88.0	12.0	65.0	23.0	0.019	26.0	29.0	11.0	0.7
1.75	5.75		5	1.92	0.72	7.9	K-4 b	84.0	16.0	74.0	10.0	0.038				
1.60	5.25		2	2.00	0.71	3.2	SK4-5	-	-	-	-	-				
2.21	7.25		3	1.71	0.76	4.4	K-3	80.0	21.0	73.0	7.0	0.044				
2.21	7.25		7	1.71	0.76	10.2	K-4	74.0	26.0	68.0	6.0	0.052				
2.67	8.75		7	1.56	0.78	9.7	K-4 b	86.0	14.0	83.0	3.0	0.049				
2.82	9.25		1	1.52	0.79	1.4	SK4-5	-	-	-	-	-				
2.97	9.75		3	1.50	0.80	4.1	K-4	62.0	38.0	53.0	9.0	0.056	28.0			
3.58	11.75		7	1.41	0.83	9.3	K-4 b	88.0	12.0	79.0	9.0	0.042				
3.73	12.25		9	1.40	0.84	11.9	K-4	96.0	4.0	83.0	13.0	0.028	30.0			
3.89	12.75		2	1.38	0.85	2.6	SK4-5	-	-	-	-	-				
4.19	13.75		3	1.34	0.86	3.9	K-3	91.0	9.0	79.0	12.0	0.039				
4.50	14.75		8	1.31	0.87	10.4	K-4	83.0	17.0	74.0	9.0	0.047	29.0			
4.50	14.75		5	1.31	0.87	6.5	K-4 b	81.0	19.0	71.0	10.0	0.042				
4.95	16.25		5	1.27	0.89	6.4	SK4-5	-	-	-	-	-				
5.26	17.25		12	1.24	0.90	15.2	K-4	89.0	11.0	83.0	6.0	0.048				
5.41	17.75		12	1.23	0.90	15.1	K-4 b	44.0	56.0	40.0	4.0	0.700				
5.56	18.25		9	1.22	0.91	11.3	K-3	30.0	70.0	29.0	1.0	0.082				
					**Mean:**	7.1		83.0	17.1	74.6	8.4	0.045				
					**St. Dev.**	4.1		9.6	9.7	9.3	3.0	0.008				

**Case number:**	80
**Earthquake:**	1979 Imperial Valley
**Magnitude:**	6.53 (Mw) Pacific Earthquake Engineering Research Center: NGA Database
**Location:**	McKim Ranch A
**References:**	Bennett et al. (1984) and Fear et al. (1995)<br>James Gerard Bierschwale. (1984)
**Nature of Failure:**	Fissures developed and as many as one<br>hundred sand boils erupted at this location. The sand boils occurred between<br>two continuous fissures, some 10-15 m apart, that parallel each other in an<br>arcuate north-to-south zone approximately 1.8 km long.
**Comments:**	The Imperial Valley lies in the southern part of the Salton Basin, which was<br>formed by northward extension of the crustal rifting that opened the Gulf of<br>California. The basin is underlain by as much ad 20,000 ft of Tertiary and<br>Quaternary marine, continental, and lacustrine sediment. Holocene sediment<br>is as thick as 330 ft.<br>The principal rivers are the Alamo and New Rivers along which many sand boils<br>were developed on fluvial deposits.<br>Located between the Alamo River and the Redwood Canal.<br>A: very fine sand (fining upwards)-loose sand that filled the relic channel.<br>B: stiff silty clay<br>McKim Ranch Site is 6 km from the northern terminus of the fault rupture. The<br>earthquake specific PGA contour maps indicate a PGA level of 0.5-0.8g.<br>2-3 km away from site, at the seismagraphic station EO4 located on deep<br>alluvium (>300m), PGA was recorded as 0.49g.<br>Energy ratio ~68 %

Summary of Data

	Cetin et al. (2016)	Idriss&Boulanger (2010)	Seed et.al. (1984)		Cetin et al. (2016)	Idriss&Boulanger (2010)	Seed et.al. (1984)
Liquefied?	Yes	Yes	Yes	D_{50}	0.106 ± 0.005		0.105
Data Class	B			% Fines	19.8 ± 3.5	31	31
Critical Depth Range	5.0 - 13.0	6.9	7.0	% PI			
Depth to GWT (ft)	5.0	4.9	5.0				
σ_v (psf)	1030.0 ± 161.2	793.6	800.0	N	5.4 ± 2.6	3	3.0
σ_v' (psf)	780.4 ± 80.7	668.3	675.0	C_R	0.79	0.80	0.8
a_{max} (g)	0.510 ± 0.153	0.51	0.510	C_S	1.00	1.00	↑
r_d	0.94 ± 0.042	0.98	0.990	C_B	1.00	1.00	↕
CSR	0.411 ± 0.127	0.385	0.390	C_E	1.13	1.13	↓ 1.050
Equivalent Magnitude	6.53	6.53		C_N	1.65	1.70	1.6
MSF		1.29	1.16	$(N_1)_{60}$	7.9 ± 3.8	4.6	4.00
CSRN		0.271	0.34				

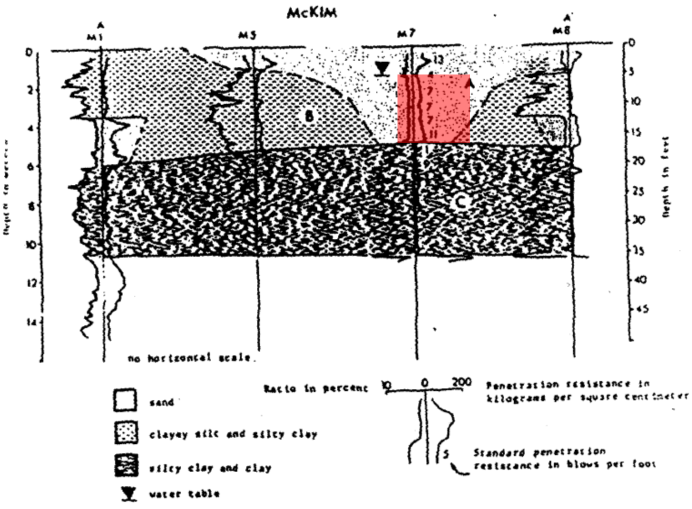

γ_m=110±3 pcf

γ_s=120±3 pcf

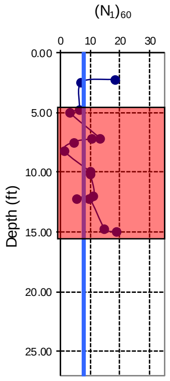

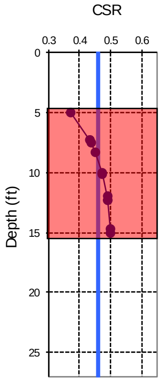

Depth (m)	Depth (ft)	CSR	N	C_N	C_R	$(N1)_{60}$	
0.69	2.25	NA	13	2.00	0.62	18.3	M-7
0.76	2.50	NA	5	2.00	0.63	7.2	SM-7
1.45	4.75	NA	4	2.00	0.70	***6.3***	***M-7***
1.52	5.00	0.322	2	1.96	0.71	***3.1***	***SM-7***
2.21	7.25	0.382	9	1.76	0.76	***13.6***	***M-2***
2.21	7.25	0.382	7	1.76	0.76	***10.6***	***M-7***
2.29	7.50	0.387	3	1.75	0.76	***4.5***	***SM-7***
2.51	8.25	0.400	1	1.69	0.78	***1.5***	***M-3***
3.05	10.00	0.423	7	1.59	0.81	***10.2***	***SM-7***
3.09	10.15	0.425	7	1.58	0.81	***10.1***	***M-7***
3.66	12.00	0.440	8	1.49	0.84	***11.3***	***SM-7***
3.73	12.25	0.441	4	1.48	0.84	***5.6***	***M-3***
3.73	12.25	0.441	7	1.48	0.84	***9.8***	***M-7***
4.50	14.75	0.450	11	1.38	0.87	15.0	M-7
4.57	15.00	0.450	14	1.37	0.87	19.0	SM-7
					**Mean:**	7.9	
					**St. Dev.**	3.8	

Depth (ft)	FC	sand	silt	clay	D50	
2.25	31.0	69.0	18.0	13.0	0.085	M-7
2.50	-	-	-	-	-	SM-7
4.75	***-***	***-***	***-***	***-***	***-***	***M-7***
5.00	***-***	***-***	***-***	***-***	***-***	***SM-7***
7.25	***-***	***-***	***-***	***-***	***-***	***M-2***
7.25	***24.0***	***76.0***	***14.0***	***10.0***	***0.100***	***M-7***
7.50	***-***	***-***	***-***	***-***	***-***	***SM-7***
8.25	***-***	***-***	***-***	***-***	***-***	***M-3***
9.40	***21.0***	***79.0***	***11.0***	***10.0***	***0.105***	***M-7***
10.00	***-***	***-***	***-***	***-***	***-***	***SM-7***
10.15	***16.0***	***84.0***	***9.0***	***7.0***	***0.110***	***M-7***
12.00	***-***	***-***	***-***	***-***	***-***	***SM-7***
12.25	***-***	***-***	***-***	***-***	***-***	***M-3***
12.25	***18.0***	***82.0***	***11.0***	***7.0***	***0.110***	***M-7***
14.75	22.0	78.0	15.0	7.0	0.093	M-7
15.00	-	-	-	-	-	SM-7
	19.8	80.3	11.3	8.5	0.106	
	3.5	3.5	2.1	1.7	0.005	

**Case number:**	81
**Earthquake:**	1979 Imperial Valley
**Magnitude:**	6.53 (Mw) Pacific Earthquake Engineering Research Center: NGA Database
**Location:**	Radio Tower B1
**References:**	James Gerard Bierschwale. (1984)<br>Bennett et al. (1984) and Fear et al. (1995)
**Nature of Failure:**	Liquefied during both the 1979 and 1981 earthquakes. Several sandboils expelled considerable volumes of water and sand and created a new pond near the radio tower at the site. Small fissures devepoed around the pond and larger fissures developed at the margin of the river flood plane locally in the adjacent bluffs.
**Comments:**	The Imperial Valley lies in the southern part of the Salton Basin, which was formed by northward extension of the crustal rifting that opened the Gulf of California. The basin is underlain by as much ad 20,000 ft of Tertiary and Quaternary marine, continental, and lacustrine sediment. Holocene sediment is as thick as 330 ft.<br>The principal rivers are the Alamo and New Rivers along which many sand boils were developed on fluvial deposits.<br>A: very poorly sorted, soft brown clay and silt-flood plain deposit<br>B: brown, loose to med.dense silt to fine sand- sediment at base is well sorted very fine sand fining upwards to poorly sorted silt-channel deposit<br>C: very stiff brown clayey silt to clay-lacustrine<br>D: med dense sand<br>Radio Tower Site is 12 km from the northern terminus of the fault rupture. 0.22g and 0.16 of PGA was recorded at BRA recording site which is 9-10 km from the northern terminus of the fault rupture. Based on the earthquake specific PGA contours, the estimated PGA at the River site is ~0.2 g. However, site response analyses results suggested a slightly lower value: 0.18 g<br>Energy ratio ~68 %

Summary of Data

	Cetin et al. (2016)	Idriss&Boulanger (2010)	Seed et.al. (1984)		Cetin et al. (2016)	Idriss&Boulanger (2010)	Seed et.al. (1984)
Liquefied?	Yes	Yes	Yes	D_{50}	0.074 ± 0.037		0.047
Data Class	B			% Fines	43.5 ± 29.0	64.0	75
Critical Depth Range	9.8 - 18.0	11.2	11.0	% PI			
Depth to GWT (ft)	6.6	6.9	7.0				
σ_v (psf)	1505.1 ± 160.1	1294.9	1270.0	N	4.5 ± 3.4	2.0	2.0
σ_v' (psf)	1044.5 ± 79.5	1044.3	1020.0	C_R	0.86	0.85	↑
a_{max} (g)	0.180 ± 0.027	0.20	0.200	C_S	1.00	1.00	1.050
r_d	0.97 ± 0.030	0.96	0.960	C_B	1.00	1.00	
CSR	0.164 ± 0.026	0.154	0.160	C_E	1.13	1.13	↓
Equivalent Magnitude	6.53	6.53		C_N	1.42	1.49	1.4
MSF		1.29	1.19	$(N_1)_{60}$	6.2 ± 4.7	2.9	3.00
CSRN		0.112	0.14				

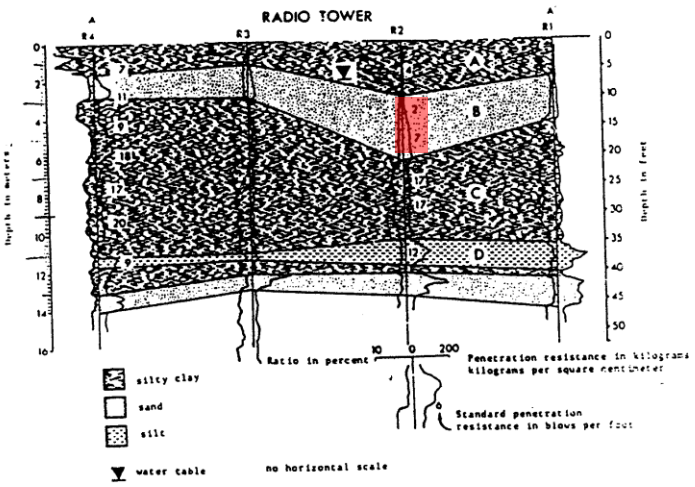

γ_m=100±3 pcf

γ_s=115±3 pcf

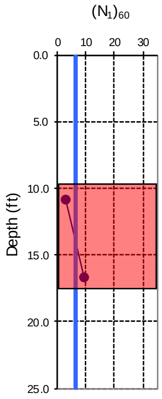

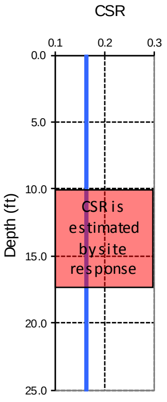

Depth (m)	Depth (ft)	CSR	N	C_N	C_R	$(N1)_{60}$
3.3	10.8		2	1.55	0.82	***2.9***
5.1	16.7		7	1.33	0.89	***9.5***
					**Mean:**	6.2
					**St. Dev.**	4.7

Depth (m)	FC	sand	silt	clay	D50
3.3	***64.0***	***36.0***	***59.0***	***5.0***	***0.047***
5.1	***23.0***	***77.0***	***22.0***	***1.0***	***0.100***
23.15	*18.0*	*82.0*	*13.0*	*5.0*	*0.125*
	43.5	56.5	40.5	3.0	0.1
	29.0	29.0	26.2	2.8	0.0

**Case number:**	82
**Earthquake:**	1979 Imperial Valley
**Magnitude:**	6.53 (Mw) Pacific Earthquake Engineering Research Center: NGA Database
**Location:**	Radio Tower B2
**References:**	James Gerard Bierschwale. (1984)<br>Bennett et al. (1984) and Fear et al. (1995)
**Nature of Failure:**	Liquefied during both the 1979 and 1981 earthquakes. Several sandboils expelled considerable volumes of water and sand and created a new pond near the radio tower at the site. Small fissures developed around the pond and larger fissures developed at the margin of the river flood plane locally in the adjacent bluffs. This borelog is from the "nonliquefied" section
**Comments:**	The Imperial Valley lies in the southern part of the Salton Basin, which was formed by northward extension of the crustal rifting that opened the Gulf of California. The basin is underlain by as much ad 20,000 ft of Tertiary and Quaternary marine, continental, and lacustrine sediment. Holocene sediment is as thick as 330 ft.<br>The principal rivers are the Alamo and New Rivers along which many sand boils were developed on fluvial deposits.<br>A: very poorly sorted, soft brown clay and silt-flood plain deposit<br>B: brown, loose to med.dense silt to fine sand- sediment at base is well sorted very fine sand fining upwards to poorly sorted silt-channel deposit<br>C: very stiff brown clayey silt to clay-lacustrine<br>D: med dense sand<br>Radio Tower Site is 12 km from the northern terminus of the fault rupture. 0.22g and 0.16 of PGA is recorded at BRA recording site which is 9-10 km from the northern terminus of the fault rupture. The earthquake specific PGA contours estimated a PGA of ~ 0.2 g at the River site. Based on site response analysis results a PGA value of 0.18 g is adopted<br>Energy ratio ~68 %

Summary of Data

	Cetin et al. (2016)	Idriss&Boulanger (2010)	Seed et.al. (1984)		Cetin et al. (2016)	Idriss&Boulanger (2010)	Seed et.al. (1984)
Liquefied?	No	No	No	D_{50}	0.100 ± 0.050		0.1
Data Class	B			% Fines	18.0 ± 2.0	30.0	30
Critical Depth Range	6.6 - 9.8	7.5	7.5	% PI			
Depth to GWT (ft)	6.6	6.9	7.0				
σ_v (psf)	852.8 ± 67.4	835.4	830.0	N	11.3 ± 1.4	11.0	11.0
σ_v' (psf)	750.5 ± 39.0	793.6	800.0	C_R	0.77	0.80	0.8
a_{max} (g)	0.180 ± 0.027	0.20	0.200	C_S	1.00	1.00	↑
r_d	0.99 ± 0.020	0.98	0.980	C_B	1.00	1.00	|
CSR	0.132 ± 0.021	0.132	0.130	C_E	1.13	1.13	↓ 1.050
Equivalent Magnitude	6.53	6.53		C_N	1.68	1.53	1.5
MSF		1.29	1.13	$(N_1)_{60}$	16.6 ± 2.0	15.2	13.00
CSRN		0.093	0.12				

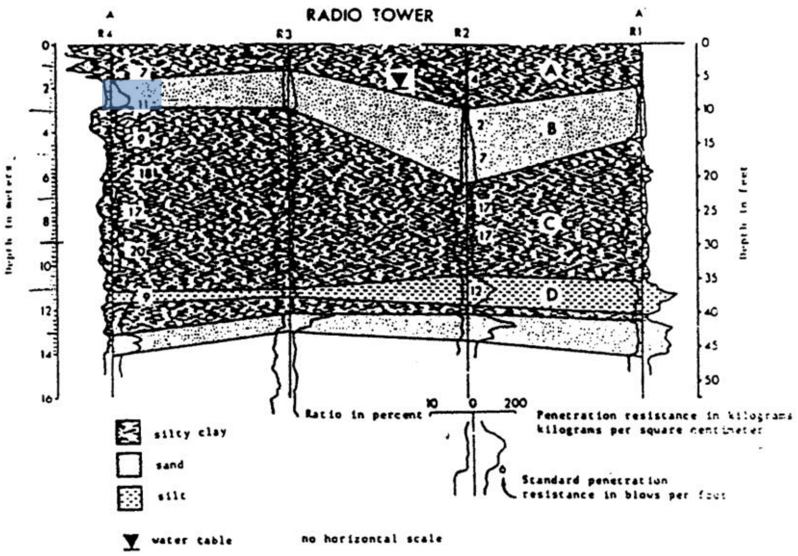

γ_m=100±3 pcf

γ_s=120±3 pcf

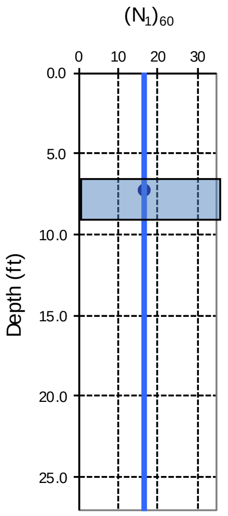

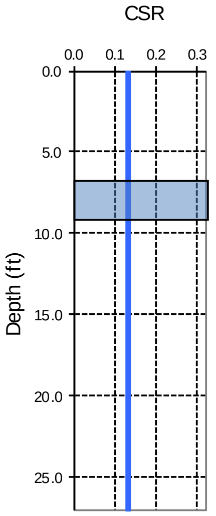

CSR is estimated by site response

Depth (m)	Depth (ft)	CSR	N	C_N	C_R	$(N1)_{60}$	FC	silt	clay	D50
2.3	7.3		11	1.74	0.76	*16.6*	*18.0*	*17.0*	*1.0*	*0.10*
					**Mean:**	16.6				
					**St. Dev.**	2.0	assigned			

**Case number:**	83
**Earthquake:**	1979 Imperial Valley
**Magnitude:**	6.53 (Mw) Pacific Earthquake Engineering Research Center: NGA Database
**Location:**	River Park A
**References:**	Youd et al. (1983), "Liquefaction Sites, Imperial Valley, CA
**Nature of Failure:**	Hundreds of sand boils, a slump, and numerous small ground cracks. Water continued to seep from a few sand boils for two weeks.
**Comments:**	The Imperial Valley lies in the southern part of the Salton Basin, which was formed by northward extension of the crustal rifting that opened the Gulf of California. The basin is underlain by as much as 20,000 ft of Tertiary and Quaternary marine, continental, and lacustrine sediment. Holocene sediment is as thick as 330 ft.<br>The principal rivers are the Alamo and New Rivers along which many sand boils were developed on fluvial deposits.<br>River Park Site is 3.4 km from the northern terminus of the fault rupture.PGA of 0.22-0.16g were recorded at Brawley Airport recording site which is 5.9 km from the northern terminus of the fault rupture. Based on the earthquake specific PGA contours, the estimated PGA at the River site is ~0.2 g. The directivity and the basin effects are significant. Contrary, site response analyses results suggest a lower value (probably due to significant strain softening) ~0.16 g<br>ASTM D1586-67 procedures were followed for SPT tests. 10" outside diameter hollow-stem auger was used with Safe-T Driver and safety hammer.<br>Energy ratio ~68 %<br>SPT blowcounts were taken in 1979 and 1981.

Summary of Data

	Cetin et al. (2016)	Idriss&Boulanger (2010)	Seed et.al. (1984)		Cetin et al. (2016)	Idriss&Boulanger (2010)	Seed et.al. (1984)
Liquefied?	Yes	Yes	Yes	D_{50}	0.010 ± 0.050		0.04
Data Class	B			% Fines	91.0 ± 2.0	80.0	80
Critical Depth Range	1.0 - 5.9	5.9	6.0	% PI			
Depth to GWT (ft)	1.0	1.0	1.0				
σ_v (psf)	369.8 ± 90.3	731.0	735.0	N	2.7 ± 2.3	3.0	3.0
σ_v' (psf)	216.8 ± 42.7	417.7	425.0	C_R	0.66	0.80	0.8
a_{max} (g)	0.160 ± 0.024	0.24	0.240	C_S	1.00	1.00	
r_d	0.99 ± 0.015	0.98	0.990	C_B	1.00	1.00	
CSR	0.176 ± 0.032	0.266	0.265	C_E	1.13	1.13	1.050
Equivalent Magnitude	6.5	6.5		C_N	2.00	1.70	1.9
MSF		1.29	1.15	$(N_1)_{60}$	4.0 ± 3.4	4.6	4.50
CSRN		0.187	0.23				

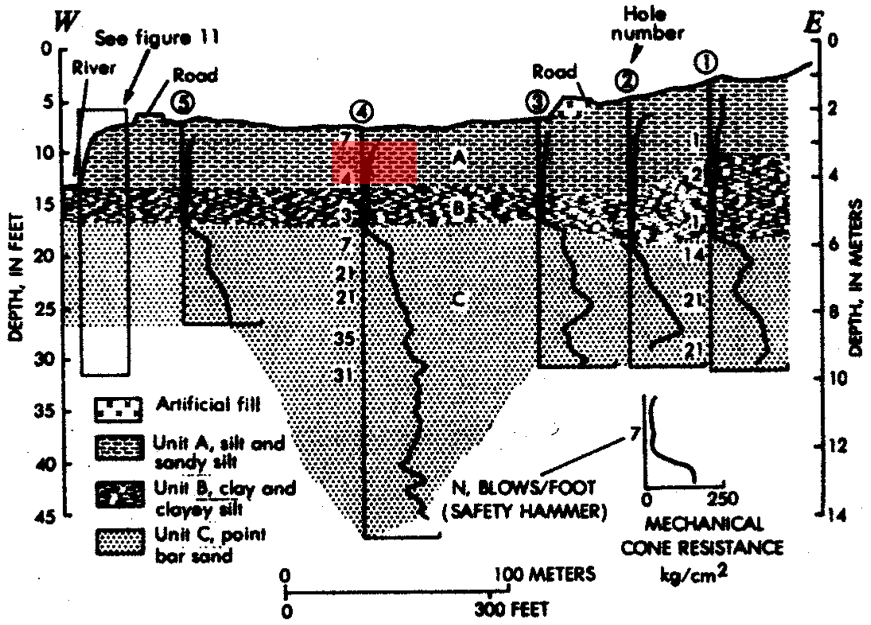

γ_m=100±3 pcf

γ_s=110±3 pcf

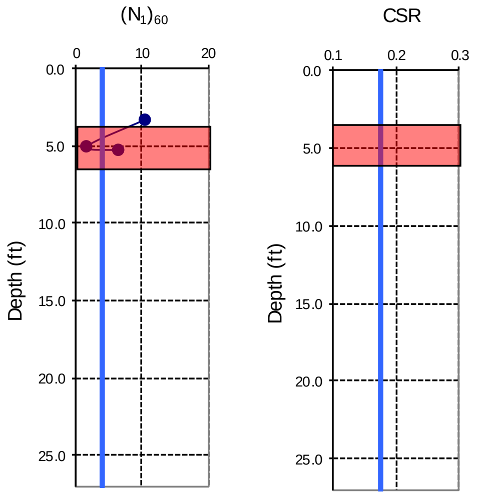

CSR is estimated by site response

Depth (m)	Depth (ft)	CSR	N	C_N	C_R	$(N1)_{60}$	
1.00	3.3		7	2.00	0.66	10.4	BH-4 (1979)
1.52	5.0		1	2.00	0.71	***1.6***	BH-15 (1981)
1.60	5.2		4	2.00	0.71	***6.5***	BH-4 (1979)
					**Mean:**	4.0	
					**St. Dev.**	3.4	

FC	sand	silt	clay	D50
-	-	-	-	0.071
91.0	***9.0***	***74.0***	***17.0***	***0.010***
-	***-***	***-***	***-***	***-***
91.0	9.0	74.0	17.0	0.010
2.0	-	-	-	0.050

**Case number:**	84
**Earthquake:**	1980 Mid-Chiba
**Magnitude:**	6.1
**Location:**	Owi-1
**References:**	Ishihara et al (1981)<br>Fear et al. (1995)
**Nature of Failure:**	No significant pore pressure generation
**Comments:**	Owi Island No 1 is located on the west side of Tokyo Bay. The island was reclaimed from 1961 to 1969 with soils dredged fom the nearby seabed to open a navigation route.Soils with different grain sizes were transported in slurry through pipes and discharged in the area. When the soil surface reached the sea level, several kinds of waste soils were dumped on top of the dredged fill. and alluvial deposits to 200-300'. In the vicinity of river the ground is relatively Two set of piezometers were embedded at two depths, I and II were installed 5m apart at depths of 6 and 13 m. III and IV were also embedded 5 m apart at depths 6 and 14 m parallel to, and 5 m from the first set. A seismograph was installed at the surface. A PGA of 45 gal due to a small magnitude EQ Mw ~4-5 has not triggered the pore pressure transducers.<br>Piezometers I and II were destroyed during the earthquake. The epicenter was located 15 km southeast of the City of Chiba, and had a focal depth of ~20 km. The intensity of shaking sorrounding the Tokyo Bay was V (JMAS).<br>PGA was recorded as 95 and 65 gal respectively in two normal directions<br>Pore pressure started to increase after the PGA exceeded 60 gal and excess pore pressure reached to the maximum values of 0.75 and 1.32 m at depths 6 and 14 m. 50 % of the excess pore pressure dissipated within 10 seconds.<br>No excess pore pressure remained at depth 14 m (FC=27) after 2 hours.<br>Seed et al estimated the CSR that will trigger the liquefaction based on laboratory test results on frozen samples. It is believed that he used number of cycles of 5-6 to represent a M=6.1 earthquake. CSR that will cause liquefaction in 5-6 cycles was predicted for depths 4.9 and 13.9 as 0.37 and 0.35 respectively. based on lab data. The CSR estimated due to earthquake was estimated as 0.232 and 0.224. So he predicted that a PGA of 0.145g (~1.5x0.95) would cause liquefaction. CSR estimates were not multiplied by 0.65<br>SPT values were taken before the earthquake.<br>SPT energy is estimated as 65 % by Seed et al. (84)

Summary of Data

	Cetin et al. (2016)	Idriss&Boulanger (2010)	Seed et.al. (1984)		Cetin et al. (2016)	Idriss&Boulanger (2010)	Seed et.al. (1984)
Liquefied?	No	No	No	D_{50}	0.120 ± 0.026		0.18
Data Class	A			% Fines	30.0 ± 16.5	13.0	13
Critical Depth Range	13.1 - 23.0	20.0	20.0	% PI			
Depth to GWT (ft)	3.3 ± 0.3	3.0	3.0				
σ_v (psf)	2165.4 ± 202.0	2255.6	2260.0	N	5.8 ± 2.3	5.0	5.0
σ_v' (psf)	1244.1 ± 106.5	1190.5	1200.0	C_R	0.94	0.95	
a_{max} (g)	0.079 ± 0.004	0.10	0.100	C_S	1.00	1.00	
r_d	0.81 ± 0.076	0.89	0.960	C_B	1.15	1.00	
CSR	0.072 ± 0.008	0.104	0.120	C_E	1.09	1.09	1.090
Equivalent Magnitude	6.1	6.0		C_N	1.30	1.36	1.3
MSF		1.48	1.26	$(N_1)_{60}$	8.9 ± 3.5	7.1	7.00
CSRN		0.067	0.10				

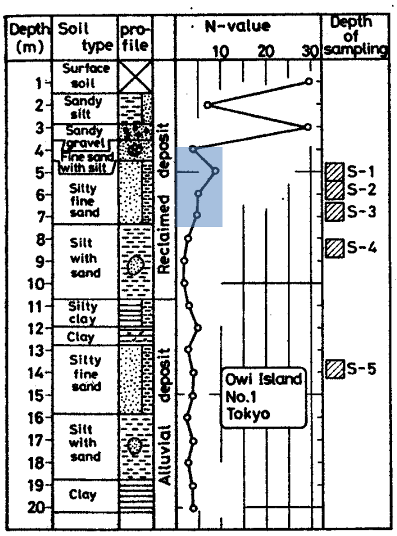

γ_m=120±3 pcf

γ_s=120±3 pcf

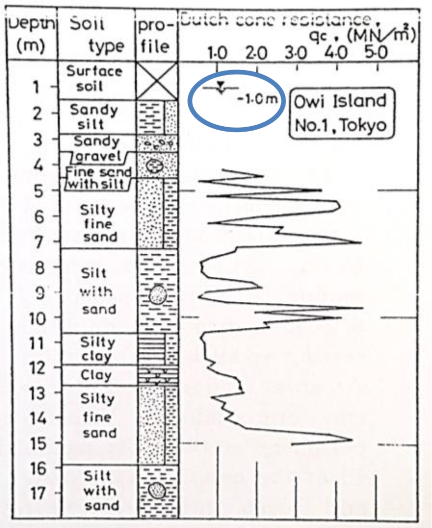

Fig. 9. Dutch cone penetration test results at the test site

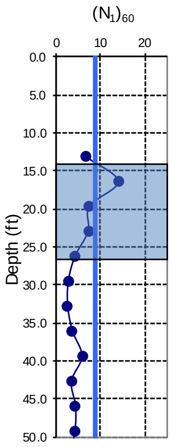

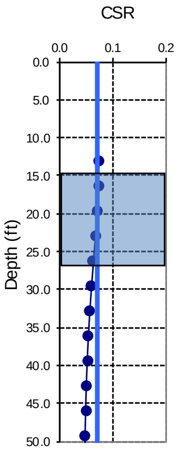

Depth (m)	Depth (ft)	CSR	N	C_N	C_R	$(N1)_{60}$
4	13.1	0.074	4	1.48	0.89	*6.6*
5	16.4	0.073	9	1.36	0.92	*14.1*
6	19.7	0.070	5	1.26	0.95	*7.5*
7	23.0	0.067	5	1.18	0.98	*7.2*
8	26.2	0.064	3	1.11	1.00	4.2
9	29.5	0.060	2	1.05	1.00	2.6
10	32.8	0.057	2	1.01	1.00	2.5
11	36.1	0.054	3	0.96	1.00	3.6
12	39.4	0.052	5	0.93	1.00	5.8
13	42.7	0.050	3	0.89	1.00	3.4
14	45.9	0.049	4	0.86	1.00	4.3
15	49.2	0.048	4	0.83	1.00	4.2
16.0	52.5	0.048	3	0.81	1.00	3.0
					**Mean:**	8.9
					**St. Dev.**	3.5

	d (m)	D_{10} (mm)	D_{50} (mm)	D_{60} (mm)	FC %
	4.9	*-*	*0.090*	*0.120*	*47.0*
	5.8	*0.045*	*0.140*	*0.160*	*14.0*
	6.7	*0.070*	*0.130*	*0.160*	*29.0*
**Mean:**	5.8	0.058	0.120	0.147	30.0
**St. Dev.**	0.9	0.035	0.026	0.023	16.5

**Case number:**	85
**Earthquake:**	1980 Mid-Chiba
**Magnitude:**	6.1
**Location:**	Owi-2
**References:**	Ishihara et al (1981)<br>Fear et al. (1995)
**Nature of Failure:**	No significant pore pressure generation
**Comments:**	Owi Island is located on the west side of Tokyo Bay. The island was<br>reclaimed from 1961 to 1969 with soils dredged fom the nearby seabed to open<br>a navigation route.Soils with different grain sizes were transported in slurry<br>through pipes and discharged in the area. When the soil surface reached the<br>sea level, several kinds of waste soils were dumped on top of the dredged fill.<br>and alluvial deposits to 200-300'. In the vicinity of river the ground is relatively<br>Two set of piezometers were embedded at two depths, I and II were installed 5m<br>apart at depths of 6 and 13 m. III and IV were also embedded 5 m apart at<br>depths 6 and 14 m parallel to, and 5 m from the first set. A seismograph<br>was installed at the surface. A PGA of 45 gal due to a small magnitude EQ<br>Mw ~4-5 has not triggered the pore pressure transducers.<br>Piezometers I and II were destroyed during the earthquake. The epicenter was<br>located 15 km southeast of the City of Chiba, and had a focal depth of ~20 km.<br>The intensity of shaking sorrounding the Tokyo Bay was V JMAS<br>PGA was recorded as 95 and 65 gal respectively in two normal directions<br>Pore pressure started to increase after the PGA exceeded 60 gal and excess<br>pore pressure reached to the maximum values of 0.75 and 1.32 m at depths<br>6 and 14 m. 50 % of the excess pore pressure dissipated within 10 seconds.<br>No excess pore pressure remained at depth 14 m (FC=27) after 2 hours.<br>Seed et al estimated the CSR that will trigger the liquefaction based on<br>laboratory test results of frozen samples. It is believed that he used number of<br>cycles of 5-6 to represent a M=6.1 earthquake. CSR that will cause liquefaction<br>in 5-6 cycles is predicted for depths 4.9 and 13.9 as 0.37 and 0.35 respectively.<br>based on lab data. The CSR estimated due to earthquake was estimated as<br>0.232 and 0.224. So he predicted that a PGA of 0.145g (~1.5x0.95) would<br>cause liquefaction. CSR estimates were not multiplied by 0.65<br>SPT values were taken before the earthquake.<br>SPT energy is estimated as 65 % by Seed et al. (84)

Summary of Data

	Cetin et al. (2016)	Idriss&Boulanger (2010)	Seed et.al. (1984)		Cetin et al. (2016)	Idriss&Boulanger (2010)	Seed et.al. (1984)
Liquefied?	No	No	No	D_{50}	0.170 ± 0.050		0.17
Data Class	B			% Fines	27.0 ± 2.0	27.0	27
Critical Depth Range	42.7 - 52.5	46.9	47.0	% PI			
Depth to GWT (ft)	3.3 ± 0.3	3.0	3.0				
σ_v (psf)	5708.7 ± 237.7	5304.9	5310.0	N	3.5 ± 0.6	4.0	4.0
σ_v' (psf)	2944.9 ± 164.4	2568.9	2560.0	C_R	1.00	1.00	
a_{max} (g)	0.079 ± 0.004	0.10	0.100	C_S	1.00	1.00	
r_d	0.49 ± 0.149	0.69	0.790	C_B	1.15	1.00	
CSR	0.049 ± 0.015	0.089	0.105	C_E	1.09	1.09	1.090
Equivalent Magnitude	6.1	6.0		C_N	0.85	0.90	0.9
MSF		1.48	1.24	$(N_1)_{60}$	3.7 ± 0.6	3.9	4.00
CSRN		0.061	0.09				

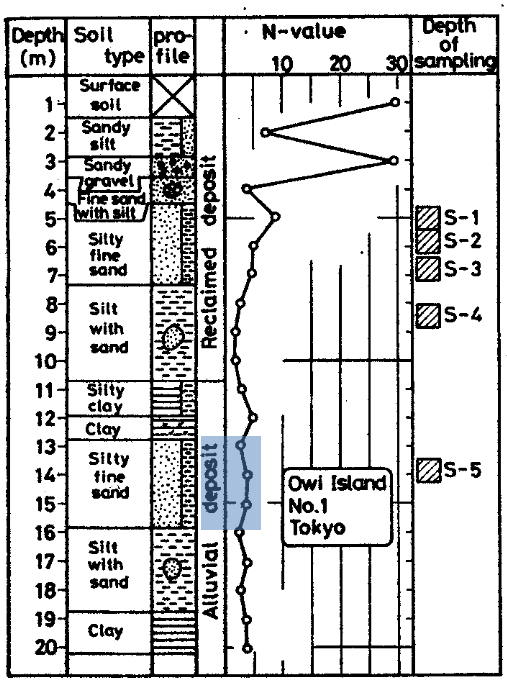

γ_m=120±3 pcf

γ_s=120±3 pcf

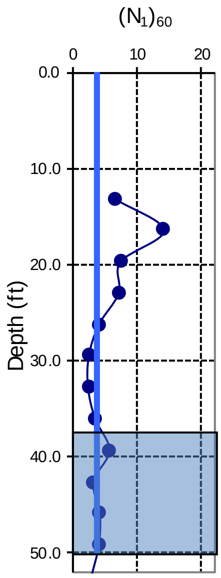

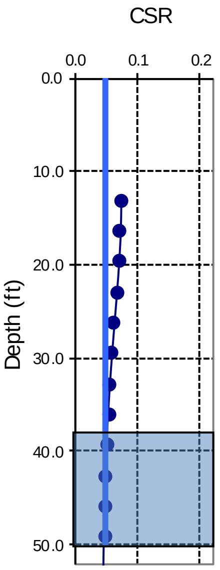

Depth (m)	Depth (ft)	CSR	N	C_N	C_R	$(N1)_{60}$
4	13.1	0.074	4	1.48	0.89	6.6
5	16.4	0.073	9	1.36	0.92	14.1
6	19.7	0.070	5	1.26	0.95	7.5
7	23.0	0.067	5	1.18	0.98	7.2
8	26.2	0.064	3	1.11	1.00	4.2
9	29.5	0.060	2	1.05	1.00	2.6
10	32.8	0.057	2	1.01	1.00	2.5
11	36.1	0.054	3	0.96	1.00	3.6
12	39.4	0.052	5	0.93	1.00	5.8
13	42.7	0.050	3	0.89	1.00	***3.4***
14	45.9	0.049	4	0.86	1.00	***4.3***
15	49.2	0.048	4	0.83	1.00	***4.2***
16.0	52.5	0.048	3	0.81	1.00	***3.0***
					**Mean:**	3.7
					**St. Dev.**	0.6

**Case number:**	86
**Earthquake:**	1981 WestMorland
**Magnitude:**	5.6 (M_L)
**Location:**	Kornbloom B
**References:**	Bennett et al. (1984) and Fear et al. (1995)<br>James Gerard Bierschwale. (1984)<br>Youd, T.L., Wieczorek G. F., (1984), "Liquefaction during the 1981 and Previous Earthquakes Near Westmorland, CA" Open File Rep. 84-680
**Nature of Failure:**	More than one hundred sand boils erupted at this site. The boils are widely scattered but generally occurred in poorly defined bands. Some of the boils erupted along short ground fissures. The unique aspect of the Kornbloom site is that the liquefied sediments were primarily silty material.<br>…the extent of the liquefiable silt layer is from 5-22'.
**Comments:**	The Imperial Valley lies in the southern part of the Salton Basin, which was formed by northward extension of the crustal rifting that opened the Gulf of California. The basin is underlain by as much as 20,000 ft of Tertiary and Quaternary marine, continental, and lacustrine sediment. Holocene sediment is as thick as 330 ft.<br>The principal rivers are the Alamo and New Rivers along which many sand boils were developed on fluvial deposits.<br>A: silty clay, stiff-lacustrine and/or flood plain deposit<br>B: silty and sandy silt-fluvial and perhaps fluvial deltaic deposition<br>C: silty clay to clay, stiff-lacustrine and/or flood plain deposit<br>D: interbedded silt and sand-loose to medium dense<br>E: Uniform sand with some silt-medium dense to dense<br>F: interbedded silt and sand - loose to medium dense.<br>D-F :fluvial and perhaps fluvial-deltaic deposition<br>PGA was estimated based on earthquake specific attenuation relationship for alluvium sites as min. 0.24, mean 0.28 and max 0.46 g.<br>at the site. Based on site specific response analyses, PGA was estimated as 0.19 g<br>Energy ratio ~68 %

**Summary of Data**	Cetin et al. (2016)	Idriss&Boulanger (2010)	Seed et.al. (1984)		Cetin et al. (2016)	Idriss&Boulanger (2010)	Seed et.al. (1984)
Liquefied?	Yes	Yes	Yes	D_{50}	0.045 ± 0.008		0.045
Data Class	A			% Fines	83.0 ± 9.6	92.0	92
Critical Depth Range	9.0 - 17.5	14.1	14.0	% PI			
Depth to GWT (ft)	9.0	8.9	9.0				
σ_v (psf)	1410.0 ± 172.7	1608.2	1615.0	N	5.4 ± 3.1	5.0	5.0
σ_v' (psf)	1144.8 ± 88.1	1294.9	1305.0	C_R	0.85	0.85	
a_{max} (g)	0.190 ± 0.029	0.32	0.320	C_S	1.00	1.00	
r_d	0.90 ± 0.012	0.93	0.970	C_B	1.00	1.00	1.050
CSR	0.137 ± 0.022	0.240	0.260	C_E	1.13	1.13	
Equivalent Magnitude	5.9	5.9		C_N	1.36	1.29	1.2
MSF		1.52	1.41	$(N_1)_{60}$	7.1 ± 4.1	6.2	6.50
CSRN		0.151	0.19				

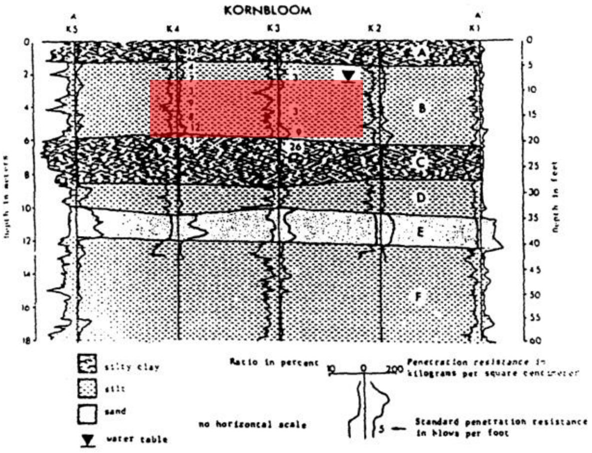

γ_m=100±3 pcf

γ_s=120±3 pcf

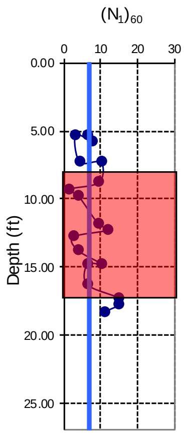

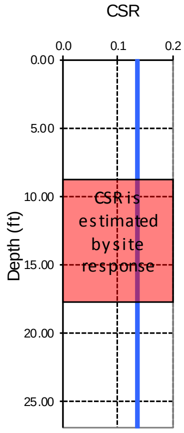

Depth (m)	Depth (ft)	CSR	N	C_N	C_R	$(N1)_{60}$		FC	sand	silt	clay	D50	WN	WL	PI	LI
1.60	5.25		4	2.00	0.71	6.5	K-4	88.0	12.0	65.0	23.0	0.019	26.0	29.0	11.0	0.7
1.75	5.75		5	1.92	0.72	7.9	K-4 b	84.0	16.0	74.0	10.0	0.038				
1.60	5.25		2	2.00	0.71	3.2	SK4-5	-	-	-	-	-				
2.21	7.25		3	1.71	0.76	4.4	K-3	80.0	21.0	73.0	7.0	0.044				
2.21	7.25		7	1.71	0.76	10.2	K-4	74.0	26.0	68.0	6.0	0.052				
2.67	8.75		7	1.56	0.78	9.7	K-4 b	86.0	14.0	83.0	3.0	0.049				
2.82	9.25		1	1.52	0.79	1.4	SK4-5	-	-	-	-	-				
2.97	9.75		3	1.50	0.80	4.1	K-4	62.0	38.0	53.0	9.0	0.056	28.0			
3.58	11.75		7	1.41	0.83	9.3	K-4 b	88.0	12.0	79.0	9.0	0.042				
3.73	12.25		9	1.40	0.84	11.9	K-4	96.0	4.0	83.0	13.0	0.028	30.0			
3.89	12.75		2	1.38	0.85	2.6	SK4-5	-	-	-	-	-				
4.19	13.75		3	1.34	0.86	3.9	K-3	91.0	9.0	79.0	12.0	0.039				
4.50	14.75		8	1.31	0.87	10.4	K-4	83.0	17.0	74.0	9.0	0.047	29.0			
4.50	14.75		5	1.31	0.87	6.5	K-4 b	81.0	19.0	71.0	10.0	0.042				
4.95	16.25		5	1.27	0.89	6.4	SK4-5	-	-	-	-	-				
5.26	17.25		12	1.24	0.90	15.2	K-4	89.0	11.0	83.0	6.0	0.048				
5.41	17.75		12	1.23	0.90	15.1	K-4 b	44.0	56.0	40.0	4.0	0.700				
5.56	18.25		9	1.22	0.91	11.3	K-3	30.0	70.0	29.0	1.0	0.082				
					**Mean:**	7.1		83.0	17.1	74.6	8.4	0.045				
					**St. Dev.**	4.1		9.6	9.7	9.3	3.0	0.008				

**Case number:**	87
**Earthquake:**	Westmorland
**Magnitude:**	5.6 (M_L)
**Location:**	Radio Tower B1
**References:**	James Gerard Bierschwale. (1984)<br>Bennett et al. (1984) and Fear et al. (1995)
**Nature of Failure:**	Liquefied during both the 1979 and 1981 earthquakes. Several sandboils expelled considerable volumes of water and sand and created a new pond near the radio tower at the site. Small fissures devepoed around the pond and larger fissures developed at the margin of the river flood plane locally in the adjacent bluffs.
**Comments:**	The Imperial Valley lies in the southern part of the Salton Basin, which was formed by northward extension of the crustal rifting that opened the Gulf of California. The basin is underlain by as much as 20,000 ft of Tertiary and Quaternary marine, continental, and lacustrine sediment. Holocene sediment is as thick as 330 ft.<br>The principal rivers are the Alamo and New Rivers along which many sand boils were developed on fluvial deposits.<br>A: very poorly sorted, soft brown clay and silt-flood plain deposit<br>B: brown, loose to med.dense silt to fine sand- sediment at base is well sorted very fine sand fining upwards to poorly sorted silt-channel deposit<br>C: very stiff brown clayey silt to clay-lacustrine<br>D: med dense sand<br>Radio Tower Site is 10 km from the epicenter of the earthquake. At Brawley seismographic station which is ~ 4 km from the River Park site and at an epicentral distance of 16 km, PGA was recorded as 0.17 and 0.15g (deep alluvium site). Based on earthquake specific attenuation relationships the mean and standard deviation of PGA were estimated as 0.18 ± 0.025<br>PGA was estimated as ~ 0.20 g by Seed et al (1984)<br>Based on site response analyses results, PGA was estimated as 0.17 g<br>Energy ratio ~68 %

Summary of Data

	Cetin et al. (2016)	Idriss&Boulanger (2010)	Seed et.al. (1984)		Cetin et al. (2016)	Idriss&Boulanger (2010)	Seed et.al. (1984)
Liquefied?	Yes	Yes	Yes	D_{50}	0.074 ± 0.037		0.047
Data Class	B			% Fines	43.5 ± 29.0	64.0	75
Critical Depth Range	9.8 - 18.0	11.2	11.0	% PI			
Depth to GWT (ft)	6.6	6.9	7.0				
σ_v (psf)	1505.1 ± 160.1	1294.9	1270.0	N	4.5 ± 3.4	2.0	2.0
σ_v' (psf)	1044.5 ± 79.5	1044.3	1020.0	C_R	0.86	0.85	↑
a_{max} (g)	0.170 ± 0.026	0.20	0.200	C_S	1.00	1.00	1.050
r_d	0.89 ± 0.012	0.95	0.960	C_B	1.00	1.00	
CSR	0.142 ± 0.022	0.152	0.160	C_E	1.13	1.13	↓
Equivalent Magnitude	5.9	5.9		C_N	1.42	1.49	1.4
MSF		1.52	1.45	$(N_1)_{60}$	6.2 ± 4.7	2.9	3.00
CSRN		0.094	0.11				

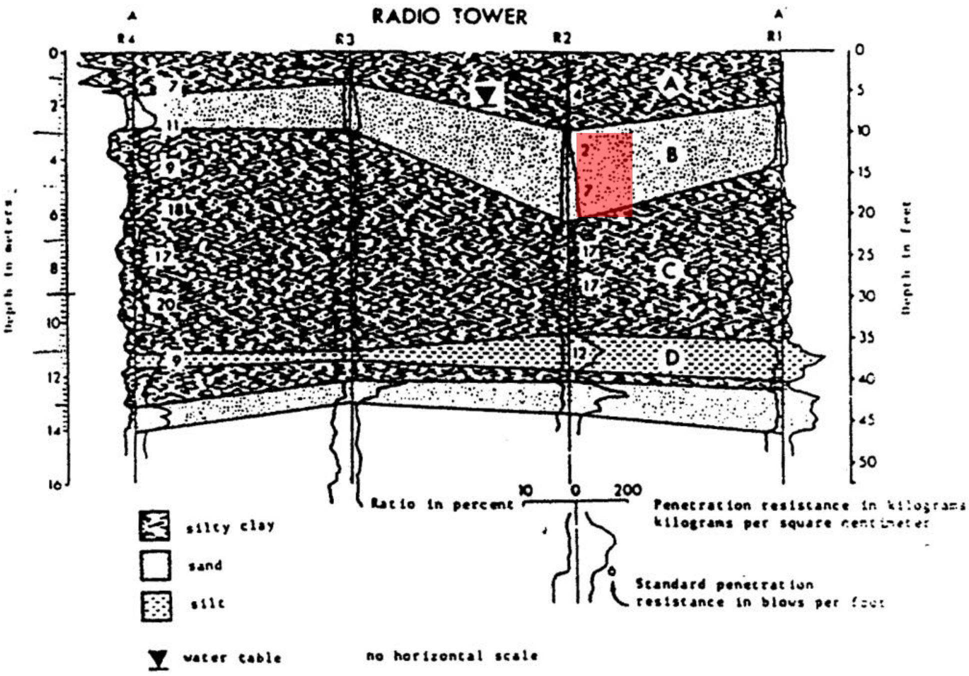

γ_m=100±3 pcf

γ_s=115±3 pcf

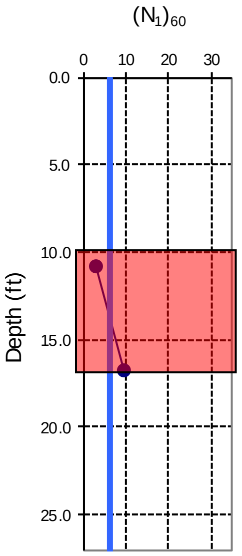

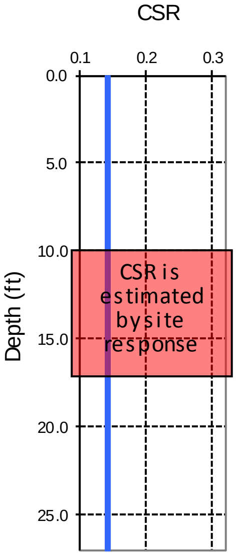

Depth (m)	Depth (ft)	CSR	N	C_N	C_R	$(N1)_{60}$
3.3	10.8		2	1.55	0.82	***2.9***
5.1	16.7		7	1.33	0.89	***9.5***
					**Mean:**	6.2
					**St. Dev.**	4.7

Depth (m)	FC	sand	silt	clay	D50
3.3	***64.0***	***36.0***	***59.0***	***5.0***	***0.047***
5.1	***23.0***	***77.0***	***22.0***	***1.0***	***0.100***
23.2	*18.0*	*82.0*	*13.0*	*5.0*	*0.125*
	43.5	56.5	40.5	3.0	0.074
	29.0	29.0	26.2	2.8	0.037

**Case number:**	88
**Earthquake:**	Westmorland
**Magnitude:**	5.6 (M_L)
**Location:**	Radio Tower B2
**References:**	James Gerard Bierschwale. (1984)<br>Bennett et al. (1984) and Fear et al. (1995)
**Nature of Failure:**	Liquefied during both the 1979 and 1981 earthquakes. Several sandboils expelled considerable volumes of water and sand and created a new pond near the radio tower at the site. Small fissures devepoed around the pond and larger fissures developed at the margin of the river flood plane locally in the adjacent bluffs. This borelog is from the "nonliquefied" section
**Comments:**	The Imperial Valley lies in the southern part of the Salton Basin, which was formed by northward extension of the crustal rifting that opened the Gulf of California. The basin is underlain by as much ad 20,000 ft of Tertiary and Quaternary marine, continental, and lacustrine sediment. Holocene sediment is as thick as 330 ft.<br>The principal rivers are the Alamo and New Rivers along which many sand boils were developed on fluvial deposits.<br>A: very poorly sorted, soft brown clay and silt-flood plain deposit<br>B: brown, loose to med.dense silt to fine sand- sediment at base is well sorted very fine sand fining upwards to poorly sorted silt-channel deposit<br>C: very stiff brown clayey silt to clay-lacustrine<br>D: med dense sand<br>Radio Tower Site is 10 km from the epicenter of the earthquake. At Brawley seismographic station which is ~ 4 km from the River Park site and at an epicentral distance of 16 km, PGA was recorded as 0.17and 0.15g (deep alluvium site). Based on earthquake specific attenuation relationships the mean and standard deviation of PGA were estimated as 0.18 ± 0.025<br>PGA was estimated by Seed et al (1984) ~0.20 g<br>Based on site response analyses results, PGA was estimated as 0.16 g.<br>Energy ratio ~68 %

**Summary of Data**	Cetin et al. (2016)	Idriss&Boulanger (2010)	Seed et.al. (1984)		Cetin et al. (2016)	Idriss&Boulanger (2010)	Seed et.al. (1984)
Liquefied?	No	No	No	D_{50}	0.100 ± 0.050		0.1
Data Class	B			% Fines	18.0 ± 2.0	30.0	30
Critical Depth Range	6.6 - 9.8	7.5	7.5	% PI			
Depth to GWT (ft)	6.6	6.9	7.0				
σ_v (psf)	852.8 ± 67.4	835.4	830.0	N	11.3 ± 1.4	11.0	11.0
σ_v' (psf)	750.5 ± 39.0	793.6	800.0	C_R	0.77	0.80	0.8
a_{max} (g)	0.160 ± 0.024	0.20	0.200	C_S	1.00	1.00	↕
r_d	0.98 ± 0.010	0.97	0.980	C_B	1.00	1.00	
CSR	0.116 ± 0.018	0.131	0.130	C_E	1.13	1.13	1.050
Equivalent Magnitude	5.9	5.9		C_N	1.68	1.53	1.5
MSF		1.52	1.37	$(N_1)_{60}$	16.6 ± 2.0	15.2	13.00
CSRN		0.078	0.10				

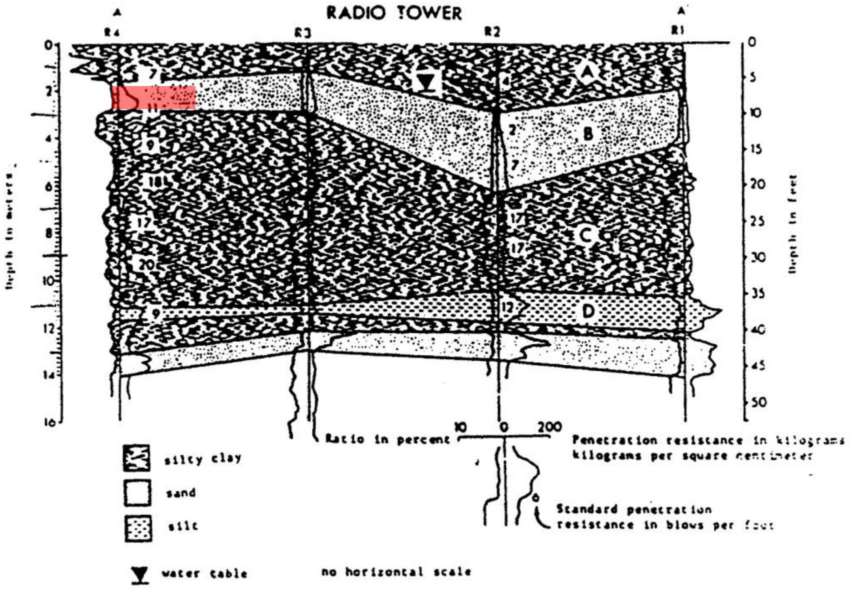

γ_m=100±3 pcf

γ_s=120±3 pcf

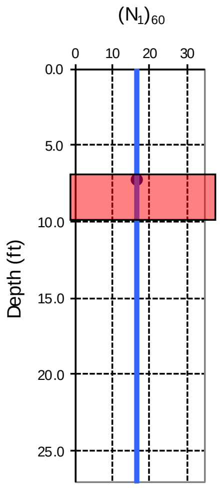

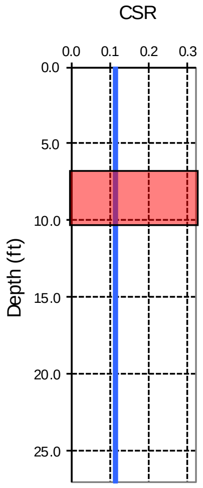

CSR is estimated by site response

Depth (m)	Depth (ft)	CSR	N	C_N	C_R	$(N1)_{60}$	FC	silt	clay	D50
2.3	7.3		11	1.74	0.76	***16.6***	***18.0***	***17.0***	***1.0***	***0.1***
					**Mean:**	16.6				
					**St. Dev.**	2.0	assigned			

**Case number:**	89
**Earthquake:**	Westmorland
**Magnitude:**	5.6 (M_L)
**Location:**	River Park A
**References:**	Youd et al. (1983), "Liquefaction sites, Imperial Valley, CA
**Nature of Failure:**	Hundreds of sand boils, a slump, and numerous small ground cracks. Water continued to seep from a few sand boils for two weeks.
**Comments:**	The Imperial Valley lies in the southern part of the Salton Basin, which was formed by northward extension of the crustal rifting that opened the Gulf of California. The basin is underlain by as much as 20,000 ft of Tertiary and Quaternary marine, continental, and lacustrine sediment. Holocene sediment is as thick as 330 ft.<br>The principal rivers are the Alamo and New Rivers along which many sand boils were developed on fluvial deposits.<br>River Park Site is 14 km from the epicenter of the earthquake. At Brawley seismographic station which is ~ 4 km from the River Park site and at an epicentral distance of 16 km, PGA was recorded as 0.17 and 0.15g (deep alluvil site). PGA was estimated by Seed et al (1984) ~0.21 g<br>Based on site response analyses results, PGA was estimated as 0.17 g.<br>ASTM D1586-67 procedures were followed for SPT tests. 10" outside diameter hollow-stem auger was used with Safe-T Driver and safety hammer.<br>Energy ratio ~68 %<br>SPT blowcounts were taken in 1979 and 1981.

Summary of Data

	Cetin et al. (2016)	Idriss&Boulanger (2010)	Seed et.al. (1984)		Cetin et al. (2016)	Idriss&Boulanger (2010)	Seed et.al. (1984)
Liquefied?	No	No	No	D_{50}	0.010 ± 0.050		0.04
Data Class	B			% Fines	91.0 ± 2.0	80.0	80
Critical Depth Range	1.0 - 5.9	5.9	6.0	% PI			
Depth to GWT (ft)	1.0	1.0	1.0				
σ_v (psf)	369.8 ± 90.3	731.0	735.0	N	2.7 ± 2.3	3.0	3.0
σ_v' (psf)	216.8 ± 42.7	417.7	425.0	C_R	0.66	0.80	0.8
a_{max} (g)	0.170 ± 0.026	0.21	0.210	C_S	1.00	1.00	
r_d	0.99 ± 0.003	0.98	0.990	C_B	1.00	1.00	
CSR	0.187 ± 0.034	0.231	0.235	C_E	1.13	1.13	1.050
Equivalent Magnitude	5.9	5.9		C_N	2.00	1.70	1.9
MSF		1.52	1.42	$(N_1)_{60}$	4.0 ± 3.4	4.6	4.50
CSRN		0.138	0.17				

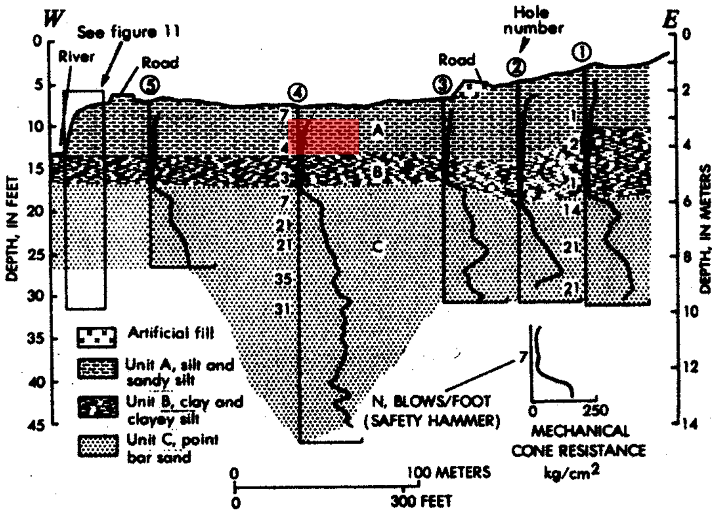

γ_m=100±3 pcf

γ_s=110±3 pcf

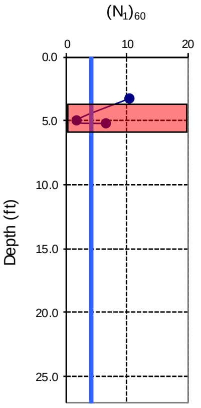

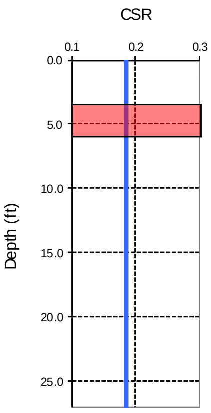

CSR is estimated by site response

Depth (m)	Depth (ft)	CSR	N	C_N	C_R	$(N1)_{60}$	
1.0	3.3		7	2.00	0.66	10.4	BH-4 (1979)
1.5	5.0		1	2.00	0.71	***1.6***	BH-15 (1981)
1.6	5.2		4	2.00	0.71	***6.5***	BH-4 (1979)
					**Mean:**	4.0	
					**St. Dev.**	3.4	

FC	sand	silt	clay	D50
-	-	-	-	0.071
91.0	***9.0***	***74.0***	***17.0***	***0.010***
-	-	-	-	-
91.0	9.0	74.0	17.0	0.010
2.0	-	-	-	0.050

**Case number:**	90
**Earthquake:**	Westmorland
**Magnitude:**	5.6 (M_L)
**Location:**	River Park C
**References:**	Youd et al. (1983), "Liquefaction sites, Imperial Valley, CA
**Nature of Failure:**	Hundreds of sand boils, a slump, and numerous small ground cracks. Water continued to seep from a few sand boils for two weeks.
**Comments:**	The Imperial Valley lies in the southern part of the Salton Basin, which was formed by northward extension of the crustal rifting that opened the Gulf of California. The basin is underlain by as much as 20,000 ft of Tertiary and Quaternary marine, continental, and lacustrine sediment. Holocene sediment is as thick as 330 ft.<br>The principal rivers are the Alamo and New Rivers along which many sand boils were developed on fluvial deposits.<br>River Park Site is 14 km from the epicenter of the earthquake. At Brawley seismographic station which is ~ 4 km from the River Park site and at an epicentral distance of 16 km, PGA was recorded as 0.17 g (deep allivual site) . PGA was estimated by Seed et al (1984) ~0.21 g.Site response analysis results suggest a PGA value of 0.17 g.<br>ASTM D1586-67 procedures were followed for SPT tests. 10" outside diameter hollow-stem auger was used with Safe-T Driver and safety hammer.<br>Energy ratio ~68 %<br>SPT blowcounts were taken in 1979 and 1981.

**Summary of Data**	Cetin et al. (2016)	Idriss&Boulanger (2010)	Seed et.al. (1984)		Cetin et al. (2016)	Idriss&Boulanger (2010)	Seed et.al. (1984)
Liquefied?	No	No	No	D_{50}	0.142 ± 0.020		0.15
Data Class	A			% Fines	13.2 ± 4.4	18.0	18
Critical Depth Range	11.0 - 17.0	14.1	14.0	% PI			
Depth to GWT (ft)	1.0	1.0	1.0				
σ_v (psf)	1595.0 ± 121.6	1733.5	1735.0	N	12.4 ± 4.9	11.0	11.0
σ_v' (psf)	783.8 ± 67.6	939.8	930.0	C_R	0.86	0.85	↑
a_{max} (g)	0.170 ± 0.026	0.21	0.210	C_S	1.00	1.00	
r_d	0.97 ± 0.010	0.93	0.960	C_B	1.00	1.00	
CSR	0.218 ± 0.034	0.237	0.245	C_E	1.13	1.13	↓ 1.050
Equivalent Magnitude	5.9	5.9		C_N	1.64	1.44	1.4
MSF		1.52	1.40	$(N_1)_{60}$	19.9 ± 7.8	15.2	16.00
CSRN		0.142	0.18				

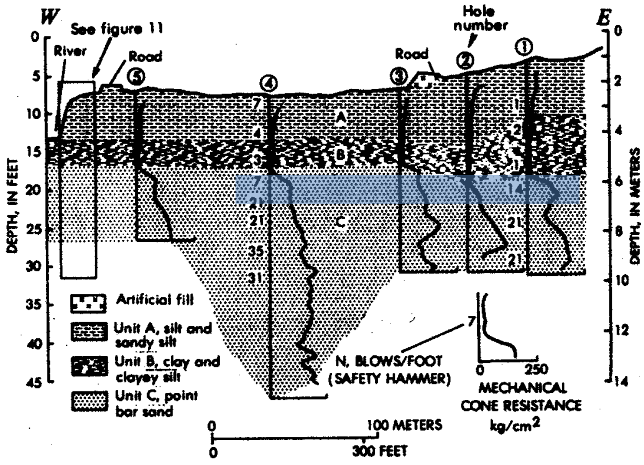

γ_m=100±3 pcf

γ_s=115±3 pcf

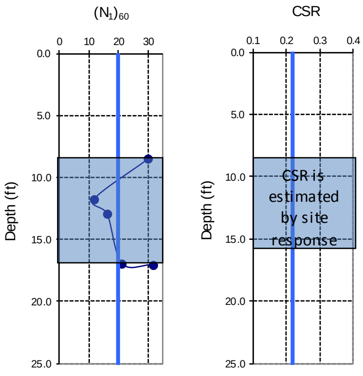

Depth (m)	Depth (ft)	CSR	N	C_N	C_R	$(N1)_{60}$	
2.6	8.5		17	2.00	0.78	*30.1*	BH-13
3.6	11.8		7	1.78	0.83	*11.8*	BH-4
3.96	13.0		10	1.70	0.85	*16.4*	BH-6
5.18	17.0		14	1.50	0.90	*21.3*	BH-1
5.2	17.1		21	1.50	0.90	32.0	BH-4
					**Mean:**	19.9	
					**St. Dev.**	7.8	

Depth (m)	FC	sand	silt	clay	D50	
**3.3**	**12**	**88**	**9**	**3**	**0.110**	BH-4
**3.5**	**13**	**87**	**11**	**2**	**0.120**	BH-4
**3.9**	**11**	**88**	**9**	**2**	**0.135**	BH-4
**4.4**	**11**	**89**	**10**	**1**	**0.140**	BH-4
**4.6**	**10**	**90**	**9**	**1**	**0.145**	BH-4
**5.1**	**10**	**90**	**10**	**0**	**0.145**	BH-4
**5.2**	**9**	**91**	**9**	**0**	**0.180**	BH-1
**5.2**	**14**	**86**	**14**	**0**	**0.170**	BH-1
**5.3**	**13**	**87**	**10**	**3**	**0.145**	BH-4
**5.7**	**18**	**82**	**14**	**4**	**0.140**	BH-4
**6.0**	**24**	**76**	**20**	**4**	**0.135**	BH-4
6.4	7	93	7	0	0.115	BH-1
7.8	6	94	6	0	0.145	BH-1
**Mean:**	13.2	86.7	11.4	1.8	0.142	
**St. Dev.**	4.4	4.3	3.4	1.5	0.020	

**Case number:**	91
**Earthquake:**	1981 WestMorland
**Magnitude:**	5.6 (M_L)
**Location:**	Wildlife B
**References:**	Bennett et al. (1984) and Fear et al. (1995)<br>James Gerard Bierschwale. (1984)<br>Youd, T.L., Wieczorek G. F., (1984), "Liquefaction during the 1981 and Previous Earthquakes Near Westmorland, CA" Open File Rep. 84-680
**Nature of Failure:**	Numerous sand boils, fissures, and slumped banks occurred along both sides of a 3 km long stretch of Alamo River within the Wildlife Management Area. Sand boils and fissures extended as far as 100 m from the river.
**Comments:**	The Imperial Valley lies in the southern part of the Salton Basin, which was formed by northward extension of the crustal rifting that opened the Gulf of California. The basin is underlain by as much as 20,000 ft of Tertiary and Quaternary marine, continental, and lacustrine sediment. Holocene sediment is as thick as 330 ft.<br>The principal rivers are the Alamo and New Rivers along which many sand boils were developed on fluvial deposits.<br>A: very loose and very soft interbedded micaceous sandy silt, silt and clayey silts (0-2.5 m) flood plain deposits<br>B: very loose to loose moderately sorted sandy silt (2.5-6.8m); point bar deposits<br>C: silty clay (medium to very stiff) and clayey silt (medium to stiff); lacustrine deposit.<br>PGA was recorded at a one-story building underlain by deep alluvium as 0.21 g at epicentral distance of 9.2 km. There is no surface faulting. The site is located ~9.5 km from the epicenter. Also at WLF station PGA was recorded as 0.19 and 0.18 g. Based on site response analyses results, PGA was estimated as as 0.23 g. Energy ratio ~68 %

Summary of Data

	Cetin et al. (2016)	Idriss&Boulanger (2010)	Seed et.al. (1984)		Cetin et al. (2016)	Idriss&Boulanger (2010)	Seed et.al. (1984)
Liquefied?	Yes	Yes	Yes	D_{50}	0.109 ± 0.038		0.09
Data Class	A			% Fines	26.2 ± 7.6	30.0	40
Critical Depth Range	9.0 - 22.0	15.1	16.0	% PI			
Depth to GWT (ft)	3.0	3.9	3.0				
σ_v (psf)	1800.0 ± 262.9	1817.0	1960.0	N	7.9 ± 3.5	7.1	9.0
σ_v' (psf)	1020.0 ± 131.5	1127.8	1150.0	C_R	0.88	0.95	↑
a_{max} (g)	0.230 ± 0.012	0.26	0.260	C_S	1.00	1.00	
r_d	0.89 ± 0.013	0.92	0.970	C_B	1.00	1.00	1.050
CSR	0.235 ± 0.014	0.267	0.280	C_E	1.13	1.13	↓
Equivalent Magnitude	5.9	5.9		C_N	1.44	1.36	1.3
MSF		1.52	1.40	$(N_1)_{60}$	11.3 ± 5.0	10.3	11.50
CSRN		0.164	0.20				

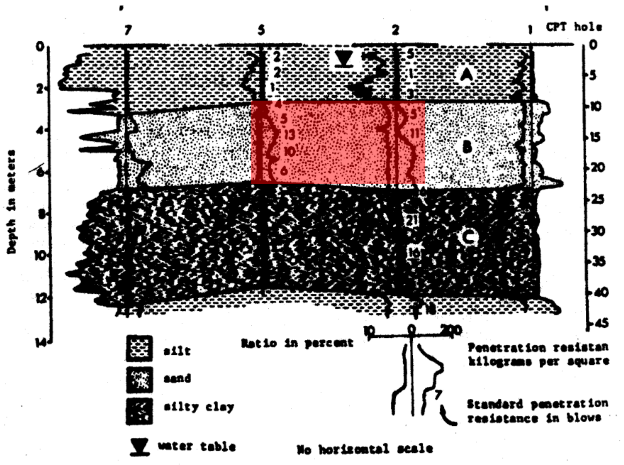

γ_m=100±3 pcf

γ_s=120±3 pcf

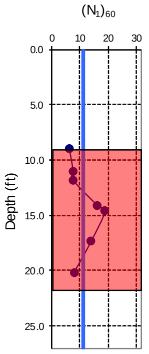

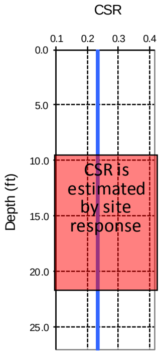

Depth (m)	Depth (ft)	CSR	N	C_N	C_R	$(N1)_{60}$	
2.75	9.0		4	1.81	0.79	***6.5***	Hole-5
3.35	11.0		5	1.67	0.82	***7.8***	Hole-2
3.6	11.8		5	1.62	0.83	***7.6***	Hole-5
4.3	14.1		11	1.50	0.86	***16.2***	Hole-2
4.45	14.6		13	1.48	0.87	***18.9***	Hole-5
5.3	17.4		10	1.37	0.90	***14.0***	Hole-5
6.15	20.2		6	1.28	0.93	***8.1***	Hole-5
					**Mean:**	11.3	
					**St. Dev.**	5.0	

Depth (ft)	FC	Sand	Silt	Clay	D50	
10.5	***34.0***	***66.0***	***34.0***	***0.0***	***0.2***	Hole-2
11.2	***17.0***	***83.0***	***17.0***	***0.0***	***0.1***	Hole-2
13.5	***27.0***	***73.0***	***26.0***	***1.0***	***0.1***	Hole-2
14.2	***20.0***	***80.0***	***20.0***	***0.0***	***0.1***	Hole-2
18.3	***33.0***	***67.0***	***29.0***	***4.0***	***0.1***	Hole-2
22.8	*18.0*	*82.0*	*17.0*	*1.0*	*0.1*	Hole-2
**Mean:**	26.2	73.8	25.2	1.0	0.109	
**St. Dev.**	7.6	7.6	6.8	1.7	0.038	

**Case number:**	92
**Earthquake:**	Westmorland
**Magnitude:**	5.6 (M_L), 5.9(Mw)
**Location:**	McKim Ranch A
**References:**	Bennett et al. (1984) and Fear et al. (1995)<br>James Gerard Bierschwale. (1984)
**Nature of Failure:**	No surface evidence of liquefaction
**Comments:**	The Imperial Valley lies in the southern part of the Salton Basin, which was formed by northward extension of the crustal rifting that opened the Gulf of California. The basin is underlain by as much as 20,000 ft of Tertiary and Quaternary marine, continental, and lacustrine sediment. Holocene sediment is as thick as 330 ft.<br>The principal rivers are the Alamo and New Rivers along which many sand boils were developed on fluvial deposits.<br>Located between the Alamo River and the Redwood Canal.<br>A: very fine sand (fining upwards)-loose sand that filled the relic channel.<br>B: stiff silty clay<br>Mc Kim Ranch is ~25 km from the epicenter of the earthquake. At SUP seismographic station which is ~ located at an epicentral distance of 24 km, PGA was recorded as 0.1 g (deep alluvial site).<br>Based on earthquake specific attenuation relationship, the mean and standard deviation were estimated as 0.12 ± 0.06 g.<br>PGA was estimated by Seed et al (1984) ~0.09 g .<br>Energy ratio ~68 %

Summary of Data

	Cetin et al. (2016)	Idriss&Boulanger (2010)	Seed et.al. (1984)		Cetin et al. (2016)	Idriss&Boulanger (2010)	Seed et.al. (1984)
Liquefied?	No	No	No	D_{50}	0.106 ± 0.005		0.105
Data Class	A			% Fines	19.8 ± 3.5	31.0	31
Critical Depth Range	5.0 - 13.0	6.9	7.0	% PI			
Depth to GWT (ft)	5.0	4.9	5.0				
σ_v (psf)	1030.0 ± 161.2	793.6	800.0	N	5.4 ± 2.6	3.0	3.0
σ_v' (psf)	780.4 ± 80.7	668.3	675.0	C_R	0.79	0.80	0.8
a_{max} (g)	0.090 ± 0.014	0.09	0.090	C_S	1.00	1.00	
r_d	0.93 ± 0.010	0.97	0.990	C_B	1.00	1.00	1.050
CSR	0.072 ± 0.012	0.068	0.070	C_E	1.13	1.13	
Equivalent Magnitude	5.9	5.9		C_N	1.65	1.70	1.6
MSF		1.52	1.40	$(N_1)_{60}$	7.9 ± 3.8	4.6	4.00
CSRN		0.040	0.05				

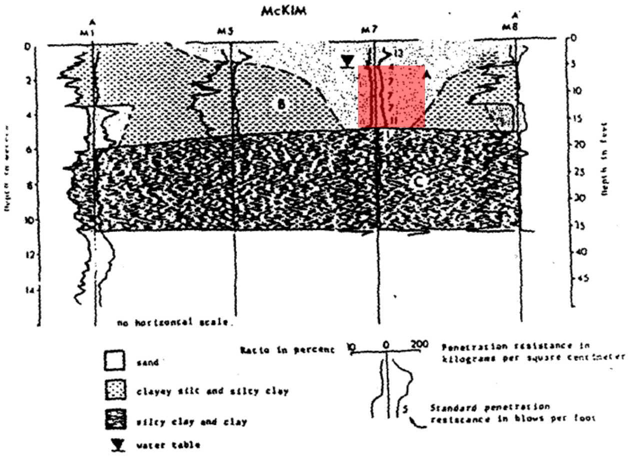

γ_m=110±3 pcf

γ_s=120±3 pcf

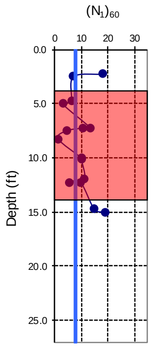

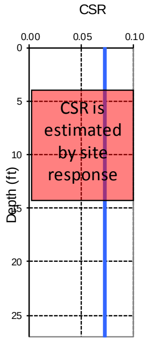

Depth (m)	Depth (ft)	CSR	N	C_N	C_R	$(N1)_{60}$	
0.7	2.3		13	2.00	0.62	18.3	M-7
0.8	2.5		5	2.00	0.63	7.2	SM-7
1.4	4.8		4	2.00	0.70	6.3	M-7
1.5	5.0		2	1.96	0.71	3.1	SM-7
2.2	7.3		9	1.76	0.76	13.6	M-2
2.2	7.3		7	1.76	0.76	10.6	M-7
2.3	7.5		3	1.75	0.76	4.5	SM-7
2.5	8.3		1	1.69	0.78	1.5	M-3
3.0	10.0		7	1.59	0.81	10.2	SM-7
3.1	10.2		7	1.58	0.81	10.1	M-7
3.7	12.0		8	1.49	0.84	11.3	SM-7
3.7	12.3		4	1.48	0.84	5.6	M-3
3.7	12.3		7	1.48	0.84	9.8	M-7
4.5	14.8		11	1.38	0.87	15.0	M-7
4.6	15.0		14	1.37	0.87	19.0	SM-7
					Mean:	7.9	
					St. Dev.	3.8	

Depth (ft)	FC	sand	silt	clay	D50	
2.3	31.0	69.0	18.0	13.0	0.085	M-7
2.5	-	-	-	-	-	SM-7
4.8	-	-	-	-	-	M-7
5.0	-	-	-	-	-	SM-7
7.3	-	-	-	-	-	M-2
7.3	24.0	76.0	14.0	10.0	0.100	M-7
7.5	-	-	-	-	-	SM-7
8.3	-	-	-	-	-	M-3
9.4	21.0	79.0	11.0	10.0	0.105	M-7
10.0	-	-	-	-	-	SM-7
10.2	16.0	84.0	9.0	7.0	0.110	M-7
12.0	-	-	-	-	-	SM-7
12.3	-	-	-	-	-	M-3
12.3	18.0	82.0	11.0	7.0	0.110	M-7
14.8	22.0	78.0	15.0	7.0	0.093	M-7
15.0	-	-	-	-	-	SM-7
	19.8	80.3	11.3	8.5	0.106	
	3.5	3.5	2.1	1.7	0.005	

**Case number:**	93
**Earthquake:**	1983 Nihonkai-Chubu
**Magnitude:**	7.1 (M)
**Location:**	Aki City (Arayamotomachi)
**References:**	Yasuda and Tohno (1988)
**Nature of Failure:**	No Surface evidence of liquefaction
**Comments:**	The Nihonkai-chubu Earthquake, with a magnitude of 7.7 occurred on May 26, 1983 in the northwest Japan and produced liquefaction sites in the coastal plain of Akita and Aomori Prefectures. About one month later, a magnitude 7.1 earthquake again hit the area and caused liquefaction at Aomori Prefecture but not at Akita. At Arayamotomachi Town in Akita City, liquefaction was caused along a narrow area by the main shock of Nihonkaichubu earthquake. The damage was concentrated in a small zone about 100 m in width and 500 m in length, in an area of land recently reclaimed over an old river by filling with sand from adjacent sand dunes. At a point near the center of the liquefied area, a layer of very loose fine sand, with SPT N values of 0-6, forms a deposit extending from the ground surface to about 6 m in depth. The water table is very shallow, only 1 m in depth.<br>The maximum surface acceleration was given as 0.2 and 0.15 g for the main and after shock respectively. The site has also liquefied during Niigata EQ. It is believed that a thin soil layer has liquefied during Niigata EQ. However, several meters of soil thickness is estimated to have been liquefied during Nihonkai-Chubu Earthquake.

**Summary of Data**	Cetin et al. (2016)	Idriss&Boulanger (2010)	Seed et.al. (1984)		Cetin et al. (2016)	Idriss&Boulanger (2010)	Seed et.al. (1984)
Liquefied?	No	No		D_{50}	0.150 ± 0.050		
Data Class	B			% Fines	5.0 ± 2.0	5.0	
Critical Depth Range	3.3 - 18.0	14.1		% PI			
Depth to GWT (ft)	3.3 ± 0.3	3.3					
σ_v (psf)	1177.0 ± 284.0	1441.1		N	4.4 ± 2.7	2.6	
σ_v' (psf)	716.4 ± 132.4	772.8		C_R	0.86	0.95	
a_{max} (g)	0.150 ± 0.045	0.15		C_S	1.00	1.00	
r_d	0.93 ± 0.048	0.95		C_B	1.00	1.00	
CSR	0.150 ± 0.047	0.172		C_E	1.22	1.22	
Equivalent Magnitude	7.1	6.8		C_N	1.72	1.70	
MSF		1.20		$(N_1)_{60}$	7.9 ± 4.8	5.1	
CSRN		0.133					

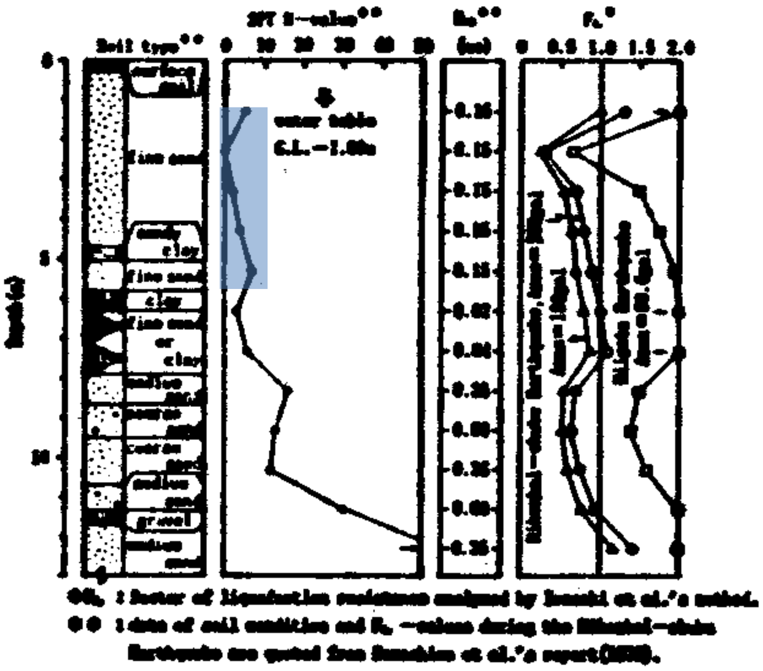

γ_m=100±3 pcf

γ_s=115±3 pcf

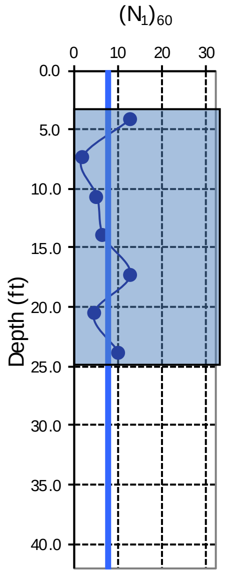

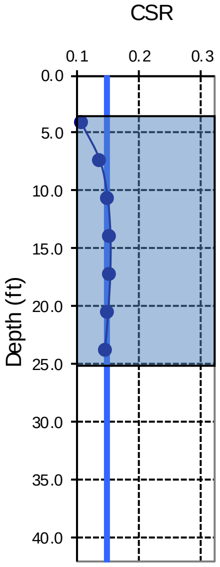

Depth (m)	Depth (ft)	CSR	N	C_N	C_R	$(N1)_{60}$	
1.3	4.1	0.109	7.0	2.00	0.75	***12.8***	fine sand
2.3	7.4	0.138	1.0	1.97	0.81	***1.9***	fine sand
3.3	10.7	0.150	3.0	1.72	0.86	***5.4***	fine sand
4.3	13.9	0.154	4.0	1.54	0.90	***6.7***	fine sand
5.3	17.2	0.154	8.0	1.41	0.93	***12.8***	fine sand
6.3	20.5	0.151	3.0	1.31	0.96	4.6	clay seam
7.3	23.8	0.146	7.0	1.23	0.98	10.3	fine sand or clay
					**Mean:**	7.9	
					**St. Dev.**	4.8	

**Case number:**	94
**Earthquake:**	1983 Nihonkai-Chubu
**Magnitude:**	7.1 (M)
**Location:**	Aki City (Arayamotomachi Coarse Sand)
**References:**	Yasuda and Tohno (1988)
**Nature of Failure:**	No surface evidence of liquefaction
**Comments:**	The Nihonkai-chubu Earthquake, with a magnitude of 7.7 occurred on May 26, 1983 in the northwest Japan and produced liquefaction sites in the coastal plain of Akita and Aomori Prefectures. About one month later, a magnitude 7.1 earthquake again hit the area and caused liquefaction at Aomori Prefecture but not at Akita. At Arayamotomachi Town in Akita City, liquefaction was caused along a narrow area by the main shock of Nihonkaichubu earthquake. The damage was concentrated in a small zone about 100 m in width and 500 m in length, in an area of land recently reclaimed over an old river by filling with sand from djacent sand dunes. At a point near the center of the liquefied area, a layer of very loose fine sand, with SPT N values of 0-6, forms a deposit extending from the ground surface to about 6 m in depth. The water table is very shallow, only 1 m in depth.<br>The maximum surface acceleration was given as 0.2 and 0.15 g for the main and after shock respectively. The site has also liquefied during Niigata EQ. It is believed that a thin soil layer has liquefied during Niigata EQ. However, several meters of soil thickness is estimated to have been liquefied during Nihonkai-Chubu Earthquake.

Summary of Data

	Cetin et al. (2016)	Idriss&Boulanger (2010)	Seed et.al. (1984)		Cetin et al. (2016)	Idriss&Boulanger (2010)	Seed et.al. (1984)
Liquefied?	No	No		D_{50}	0.420 ± 0.050		
Data Class	B			% Fines	0.0 ± 2.0	0.0	
Critical Depth Range	26.2 - 34.4	30.2		% PI			
Depth to GWT (ft)	3.3 ± 0.3	3.3					
σ_v (psf)	3576.1 ± 183.4	3299.9		N	13.1 ± 3.3	13.1	
σ_v' (psf)	1887.1 ± 114.2	1608.2		C_R	1.00	1.00	
a_{max} (g)	0.150 ± 0.045	0.15		C_S	1.00	1.00	
r_d	0.70 ± 0.118	0.86		C_B	1.00	1.00	
CSR	0.129 ± 0.044	0.172		C_E	1.22	1.22	
Equivalent Magnitude	7.1	6.8		C_N	1.06	1.13	
MSF		1.20		$(N_1)_{60}$	16.9 ± 4.3	18.1	
CSRN		0.139					

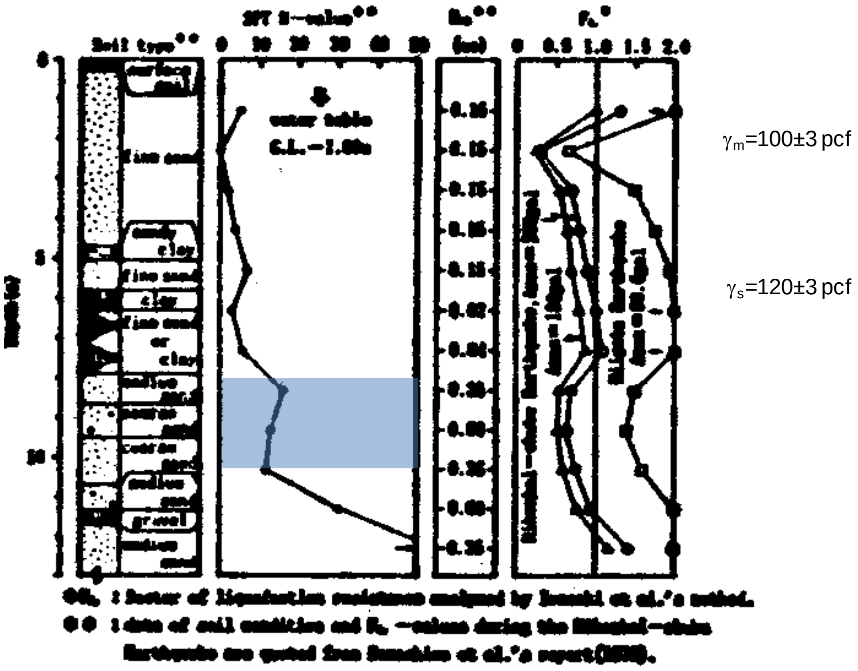

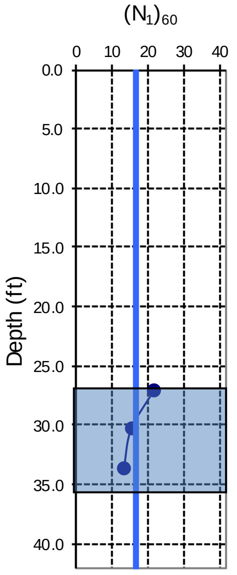

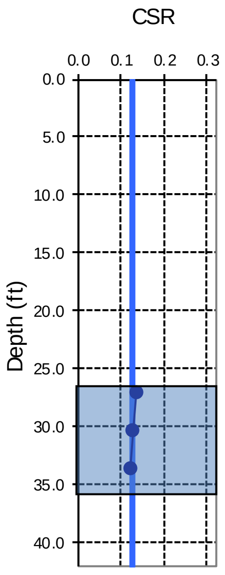

Depth (m)	Depth (ft)	CSR	N	C_N	C_R	$(N1)_{60}$
8.3	27.1	0.135	16.0	1.12	1.00	***21.7***
9.3	30.3	0.129	12.0	1.06	1.00	***15.5***
10.3	33.6	0.123	11.0	1.01	1.00	***13.5***
					**Mean:**	16.9
					**St. Dev.**	4.3

**Case number:**	95
**Earthquake:**	1983 Nihonkai-Chubu
**Magnitude:**	7.1 (M)
**Location:**	Nakazata Town (Takeda Elementary School)
**References:**	Yasuda and Tohno (1988)
**Nature of Failure:**	Sand boils, settled structures.
**Comments:**	The Nihonkai-chubu Earthquake, with a magnitude of 7.7 occurred on May 26, 1983 in the northwest Japan and produced liquefaction sites in the coastal plain of Akita and Aomori Prefectures. About one month later, a magnitude 7.1 earthquake again hit the area and caused liquefaction at the Takeda Elementary School in Nakazato Town. The school was built on the old bed of the Iwaki River. During the main shock on May 26, the ground beneath the schoolhouses (two-story wood structures) and sweeped out sand and water then was submerged. The aftershock of June 21 again caused sand and water to sweep out from the ground. According to some teachers, the area where sand volcanoes were observed almost coincided with the area where sand volcaones had been caused by the main shock. The maximum surface acceleration was suggested to be 0.28 and 0.11 g for the main and after shock respectively based on the values recorded at the Tsugaru-ohashi Bridge about 6 km from the school. Slightly higher value is ~0.12g is adopted due to possible amplification at extremely soft upper 5 ft.

Summary of Data

	Cetin et al. (2016)	Idriss&Boulanger (2010)	Seed et.al. (1984)		Cetin et al. (2016)	Idriss&Boulanger (2010)	Seed et.al. (1984)
Liquefied?	Yes	Yes		D_{50}	0.240 ± 0.050		
Data Class	B			% Fines	0.0 ± 2.0	0.0	
Critical Depth Range	8.2 - 21.3	14.1		% PI			
Depth to GWT (ft)	1.1 ± 0.4	1.1					
σ_v (psf)	1680.6 ± 254.9	1691.7		N	7.9 ± 0.9	7.4	
σ_v' (psf)	831.0 ± 123.3	877.2		C_R	0.90	0.95	
a_{max} (g)	0.120 ± 0.036	0.11		C_S	1.00	1.00	
r_d	0.85 ± 0.064	0.95		C_B	1.00	1.00	
CSR	0.134 ± 0.042	0.132		C_E	1.22	1.22	
Equivalent Magnitude	7.1	6.8		C_N	1.60	1.56	
MSF		1.20		$(N_1)_{60}$	13.8 ± 1.5	13.3	
CSRN		0.101					

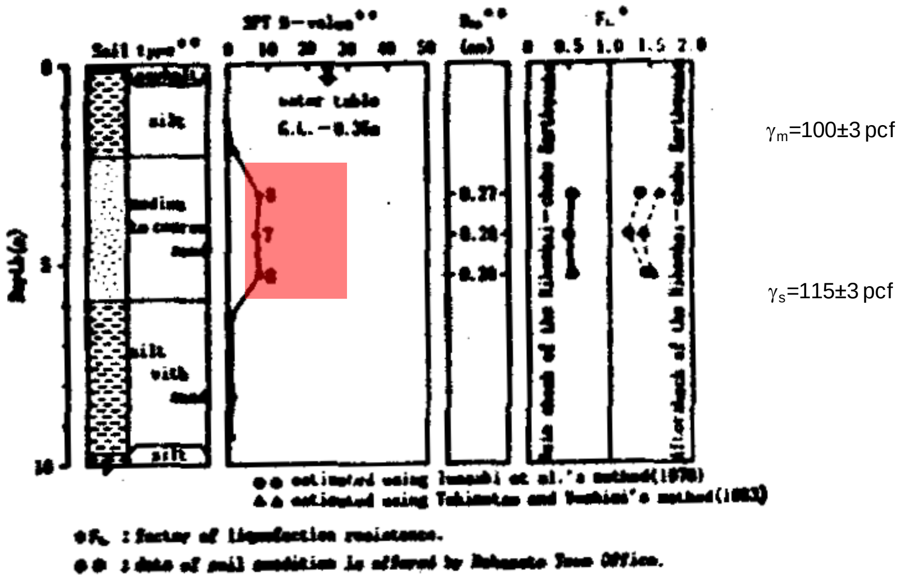

γ_m=100±3 pcf

γ_s=115±3 pcf

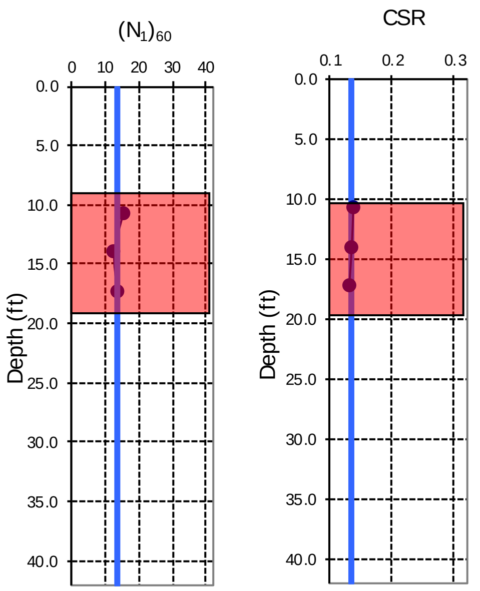

Depth (m)	Depth (ft)	CSR	N	C_N	C_R	$(N1)_{60}$
3.3	10.7	0.139	8.0	1.85	0.86	***15.5***
4.3	13.9	0.135	7.0	1.64	0.90	***12.5***
5.3	17.2	0.130	8.0	1.48	0.93	***13.4***
					**Mean:**	13.8
					**St. Dev.**	1.5

Case number: 96

Earthquake: 1983 Nihonkai-Chubu

Magnitude: 7.7 (Mw by USGS, NISEE)

Location: Akita Station

References: Iai. et. al. (1989)

Nature of Failure: Liquefied and nonliquefied areas are defined by the boundary lines between artifically filled areas and the areas of the Quaternary sediments which are shown in photos provided by Iai et. al. (1989)

Comments:

Tsuchida et. al (1985) shows the liquefied and non liquefied sites on Akita Port in Figure 8.
The Akita Station is the strong ground motion station site.
SMAC_B2 type accelerograph measured an acceleration value of 190 and 205 Gals
in NS and EW directions. Geometric mean of 190 and 205 Gals is equal to 0.204 g is assigned to the site.
SPT procedures were not clearly documented. However, on page 66 in Iai et. al. (1989) for comparison with Seed et. al. 1985,
an average Japanese SPT energy ratio of 73% was reported to be used. On the basis of this, with some uncertainty Iai et. al.
N values may be adopted as N_{73} values. Note that Seed et. al. (1985) adopted an energy ratio of 73%
for liquefied cases. The rationale behind this choice could be field data which is unknown to us or simply
a conservative engineering judgement. Soil profile data is missing just N values and FC exist.
From Technical Report of the Port and Horbour Research Institute Ministry of Transport,
Japan No. 511 Marc. 1985 the borehole data could not be obtained.
Site response by Iai et.al. exists however soil profile not known.
There is an ambiguity if the site is free field or not in the vicinty of Akita Port Channel which is 10 m in depth.
From Figure 8 the site is 75 m away from the channel.
Note that the location of the site may not be correctly located in Figure 8
since the scale is very small and the coordinates of the borehole are not available.
Additionally Quay walls are believed to be located 75 m away from site.
Presence of Quay walls and due to proximity to the Akita Port Channel which 10 m free
field stress boundary conditions may not be fulfilled. Hence cases within 20 m to the
Quay walls and channel are eliminated. In Idriss and Boulanger (2010) Akita Station
is divided into two case since there exist 2 borehole datas.
Note that for one borehole FC data is missing. In addition two boreholes at one site
is against statistically independent assumption. In Cetin 2014 Akita (1)
and (2) are composed and represented as Akita Station
by taking into account of two borehole data.

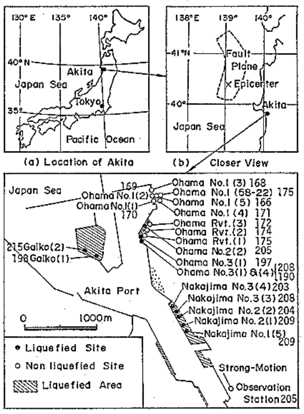

(c) Location of Investigated Sites
(three digit numbers shown at the sites are the maximum acceleration in Gals obtained by the ground response analysis)

Fig. 8. Investigated sites in Akita Port

Summary of Data

	Cetin et al. (2016)	Idriss&Boulanger (2010)	Seed et.al. (1984)		Cetin et al. (2016)	Idriss&Boulanger (2010)	Seed et.al. (1984)
Liquefied?	No			D_{50}	- ± -		
Data Class	B			% Fines	2.7 ± 1.5		
Critical Depth Range	6.6 - 13.1			% PI	-		
Depth to GWT (ft)	5.7 ± 3.0						
σ_v (psf)	1181.1 ± 132.9			N	10.3 ± 1.8		
σ_v' (psf)	925.2 ± 198.6			C_R	0.85		
a_{max} (g)	0.204 ± 0.010			C_S	1.00		
r_d	0.999 ± 0.045			C_B	1.00		
CSR	0.169 ± 0.037			C_E	1.22		
Equivalent Magnitude	7.7			C_N	1.51		
MSF	0.93			$(N_1)_{60}$	16.0 ± 2.8		
CSRN	0.18						

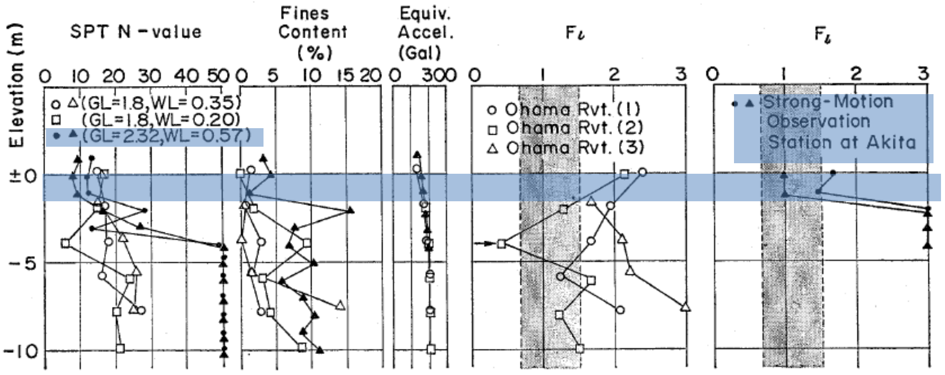

γ_m=120±3 pcf

γ_s=120±3 pcf

(e) Ohama No.2 Former Revetment and the strong-motion observation station (Nonliquefied)

Fig. 9. Ground conditions and resulted liquefaction potentials at Akita Port during 1983 Nihonkai-Chubu Earthquake

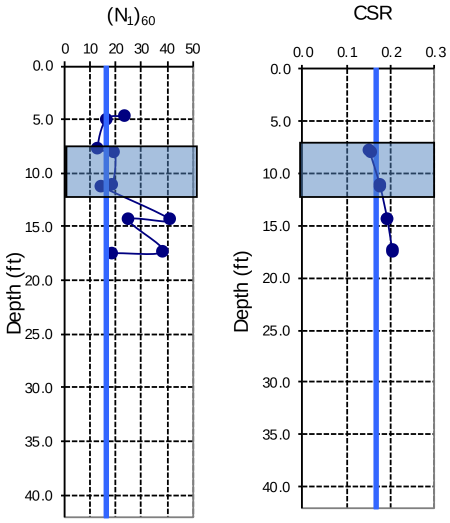

Depth (m)	Depth (ft)	CSR	N	C_N	C_R	$(N1)_{60}$	FC	
1.41	4.6	NA	13	1.95	0.76	*23.5*	-	Akita Station (1)
1.50	4.9	NA	9	1.89	0.77	***15.9***	***3.0***	Akita Station (2)
2.35	7.7	0.153	8	1.62	0.82	***12.9***	***4.0***	Akita Station (2)
2.43	8.0	0.155	12	1.61	0.82	***19.3***	**-**	Akita Station (1)
3.36	11.0	0.176	12	1.46	0.86	***18.4***	**-**	Akita Station (1)
3.40	11.2	0.177	9	1.45	0.86	***13.8***	***1.0***	Akita Station (2)
4.37	14.3	0.192	28	1.34	0.90	*41.0*	-	Akita Station (1)
4.37	14.3	0.192	17	1.34	0.90	*24.9*	*16.0*	Akita Station (2)
5.26	17.3	0.203	27	1.25	0.93	*38.2*	*8.0*	Akita Station (2)
5.32	17.5	0.203	13	1.25	0.93	*18.3*	-	Akita Station (1)
					**Mean:**	16.0	2.7	
					**St. Dev.**	2.8	1.5	

**Case number:**	97
**Earthquake:**	1983 Nihonkai-Chubu
**Magnitude:**	7.7 (M)
**Location:**	Aomori City (Aomori Station)
**References:**	Yasuda and Tohno (1988)
**Nature of Failure:**	Sand boils, settled structures.
**Comments:**	The Nihonkai-chubu Earthquake, with a magnitude of 7.7 occurred on May 26, 1983 in the northwest Japan and produced liquefaction sites in the coastal plain of Akita and Aomori Prefectures. About one month later, a magnitude 7.1 earthquake again hit the area and caused liquefaction at the Aomori Station. The station faces the sea and the north part of the station is used as a landing place for ferryboats. The north part was built on reclaimed land constructed in 1945, but the south part was built on natural land. Liquefaction was induced at the north part of the station during the main shock of the Nihonakai-chubu EQ. Many sand volcanoes, settlement of rails and buildings and uplift of buried tanks occurred. The maximum surface acceleration at Aomari Port, about 1 km north of Aomori Station was 0.116 g

Summary of Data

	Cetin et al. (2016)	Idriss&Boulanger (2010)	Seed et.al. (1984)		Cetin et al. (2016)	Idriss&Boulanger (2010)	Seed et.al. (1984)
Liquefied?	Yes	Yes		D_{50}	0.250 ± 0.050		
Data Class	A			% Fines	3.0 ± 2.0	3.0	
Critical Depth Range	13.1 - 24.6	18.7		% PI			
Depth to GWT (ft)	0.0 ± 0.4	0.0					
σ_v (psf)	2263.8 ± 236.6	1984.1		N	9.0 ± 0.9	9.0	
σ_v' (psf)	1086.6 ± 124.8	793.6		C_R	0.94	0.95	
a_{max} (g)	0.116 ± 0.006	0.12		C_S	1.00	1.00	
r_d	0.86 ± 0.079	0.96		C_B	1.00	1.00	
CSR	0.135 ± 0.015	0.178		C_E	1.22	1.22	
Equivalent Magnitude	7.7	7.7		C_N	1.40	1.58	
MSF		0.95		$(N_1)_{60}$	14.4 ± 1.4	16.5	
CSRN		0.171					

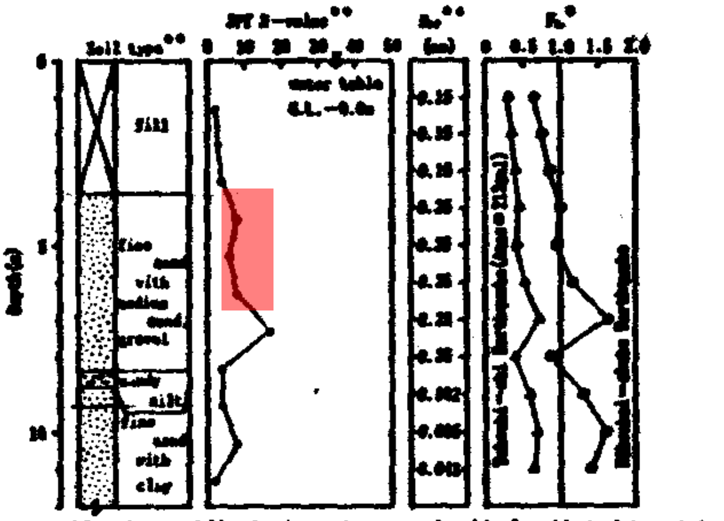

*F_L : factor of liquefaction resistance analyzed by Iwasaki et al.'s method.
** : data of soil condition and F_L -values during the Nihonkai-chubu Earthquake are quoted from the Railway Technical Research Institute, JNR(1984)

γ_m=100±3 pcf

γ_s=120±3 pcf

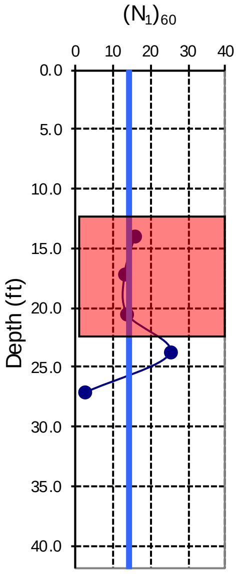

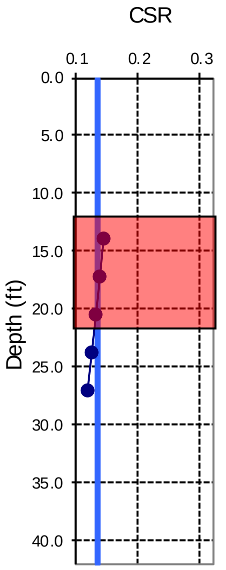

Depth (m)	Depth (ft)	CSR	N	C_N	C_R	$(N1)_{60}$	
4.3	13.9	0.143	9.0	1.62	0.90	***15.9***	
5.3	17.2	0.138	8.0	1.46	0.93	***13.2***	
6.3	20.5	0.132	9.0	1.34	0.96	***14.0***	
7.3	23.8	0.126	17.0	1.24	0.98	25.3	Gravel?
8.3	27.1	0.120	2.0	1.17	1.00	2.8	Clay?
					**Mean:**	14.4	
					**St. Dev.**	1.4	

**Case number:**	98
**Earthquake:**	1983 Nihonkai-Chubu
**Magnitude:**	7.7 (M)
**Location:**	Aki City (Arayamotomachi)
**References:**	Yasuda and Tohno (1988)
**Nature of Failure:**	Sand boils, settled structures, sewage tanks floated.
**Comments:**	The Nihonkai-chubu Earthquake, with a magnitude of 7.7 occurred on May 26, 1983 in the northwest Japan and produced liquefaction sites in the coastal plain of Akita and Aomori Prefectures. About one month later, a magnitude 7.1 earthquake again hit the area and caused liquefaction at Aomori Prefecture but not at Akita. At Arayamotomachi Town in Akita City, liquefaction was caused along a narrow area by the main shock of Nihonkaichubu earthquake. The damage was concentrated in a small zone about 100 m in width and 500 m in length, in an area of land recently reclaimed over an old river by filling with sand from djacent sand dunes. At a point near the center of the liquefied area, a layer of very loose fine sand, with SPT N values of 0-6, forms a deposit extending from the ground surface to about 6 m in depth. The water table is very shallow, only 1 m in depth.<br>The maximum surface acceleration was given as 0.2 and 0.15 g for the main and after shock respectively. The site has also liquefied during Niigata EQ. It is believed that a thin soil layer has liquefied during Niigata EQ. However, several meters of soil thickness is estimated to have been liquefied during Nihonkai-Chubu Earthquake.

Summary of Data

	Cetin et al. (2016)	Idriss&Boulanger (2010)	Seed et.al. (1984)		Cetin et al. (2016)	Idriss&Boulanger (2010)	Seed et.al. (1984)
Liquefied?	Yes	Yes		D_{50}	0.150 ± 0.050		
Data Class	B			% Fines	5.0 ± 2.0	5.0	
Critical Depth Range	3.3 - 18.0	14.1		% PI			
Depth to GWT (ft)	3.3 ± 0.3	3.3					
σ_v (psf)	1177.0 ± 284.0	1441.1		N	4.4 ± 2.7	2.6	
σ_v' (psf)	716.4 ± 132.4	772.8		C_R	0.86	0.95	
a_{max} (g)	0.200 ± 0.060	0.20		C_S	1.00	1.00	
r_d	0.94 ± 0.048	0.97		C_B	1.00	1.00	
CSR	0.200 ± 0.062	0.234		C_E	1.22	1.22	
Equivalent Magnitude	7.7	7.7		C_N	1.72	1.70	
MSF		0.95		$(N_1)_{60}$	7.9 ± 4.8	5.1	
CSRN		0.230					

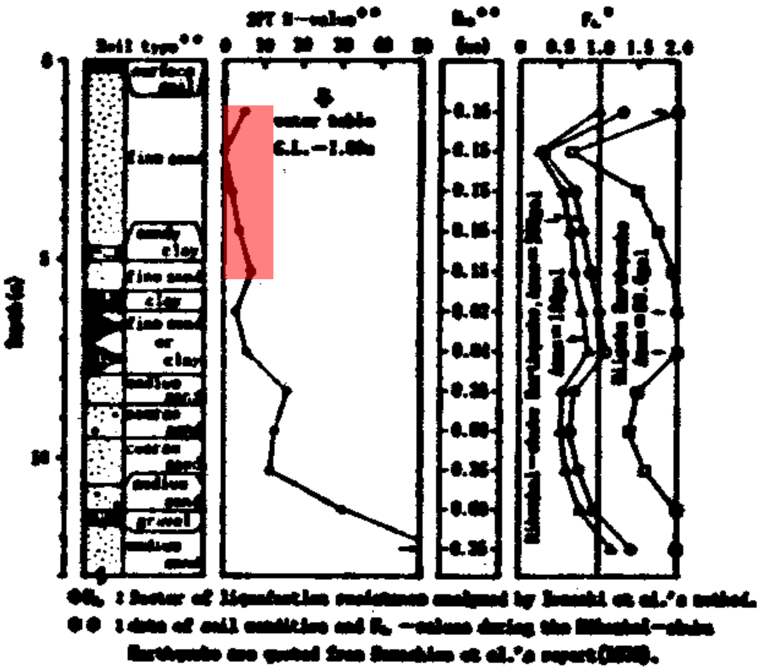

γ_m=100±3 pcf

γ_s=115±3 pcf

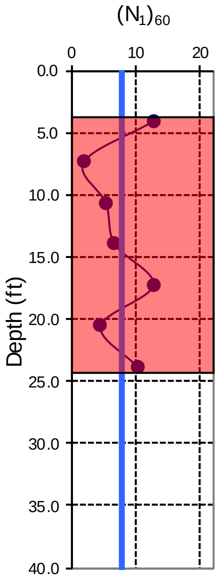

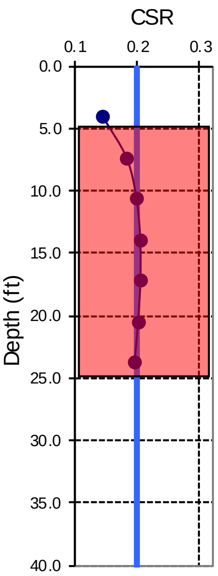

Depth (m)	Depth (ft)	CSR	N	C_N	C_R	$(N1)_{60}$	
1.3	4.1	0.145	7.0	2.00	0.75	***12.8***	fine sand
2.3	7.4	0.184	1.0	1.97	0.81	***1.9***	fine sand
3.3	10.7	0.200	3.0	1.72	0.86	***5.4***	fine sand
4.3	13.9	0.207	4.0	1.54	0.90	***6.7***	fine sand
5.3	17.2	0.207	8.0	1.41	0.93	***12.8***	fine sand
6.3	20.5	0.203	3.0	1.31	0.96	4.6	clay seam
7.3	23.8	0.197	7.0	1.23	0.98	10.3	fine sand or clay
					**Mean:**	7.9	
					**St. Dev.**	4.8	

**Case number:**	99
**Earthquake:**	1983 Nihonkai-Chubu
**Magnitude:**	7.7 (Mw by USGS, NISEE)
**Location:**	Gaiko-1&2
**References:**	Iai. et. al. (1989)
**Nature of Failure:**	Liquefied and nonliquefied areas are defined by the boundary lines between artifically filled areas and the areas of the Quaternary sediments which are shown in photos provided by Iai et. al. (1989)

Comments:

Tsuchida et. al (1985) shows the liquefied and non liquefied sites on Akita Port in Figure 8.
Gaiko-1 and Gaiko-2 is located approximatly 2.76 km away from strong ground motion station in addition the two sites are very close to each other and soil profiles are very similar. In Cetin 2015 database these two sites are decided to be combained and represetnetd as Gaiko-1&2.
SMAC_B2 type accelerograph measured an acceleration value of 190 and 205 Gals in NS and EW directions. "These two values are used for computing the rock outcrop motion. Then this rock outcrop motion is used as input for the earthquake response analysis at several sites." (Kurata et. al. 1983)
In Figure 8 three digit numbers shows the maximum acceleration values obtained from ground response analysis. However this procedure is done for one component which is 215 Gals (EW) In order ro take into account multi-directional effects site response by Iai et.al. was scaled, maximum acceleration values of 215 and 205 Gals are obtained.
Geometric mean of 215 and 204 Gals is equal to 0.214 g is assigned to site.
SPT procedures were not clearly documented. However, on page 66 in Iai et. al. for comparison with Seed et. al. 1985, an average Japanese SPT energy ratio of 73% was reported to be used. On the basis of this, with some uncertainty Iai et. al. N values may be adopted as N_{73} values. Note that Seed et. al. (1985) adopted an energy ratio of 73% for liquefied cases.
The rationale behind this choice could be field data which is unknown to us or simply a conservative engineering judgement.
From Technical Report of the Port and Horbour Research Institute Ministry of Transport, Japan No. 511 Marc. 1985 the borehole data is obtained.
Site response by Iai et.al. exists.
There is an ambiguity if the site is free field or not in the vicinty of Akita Port Channel which is 10 m in depth.
From Figure 8 the site is 37 m away from channel.
Note that the location of the site may not be correctly located in Figure 8 since the scale is very small and the coordinates of the borehole are not available.
Additionally Quay walls are believed to be located 37 m away from site.
Presence of Quay walls and due to proximity to the Akita Port Channel which 10 m free field stress boundary conditions may not be fulfilled. Hence cases within 20 m to the Quay walls and channel are eliminated.

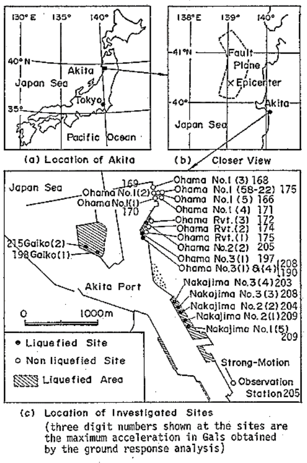

Fig. 8. Investigated sites in Akita Port

Summary of Data

	Cetin et al. (2016)	Idriss&Boulanger (2010)	Seed et.al. (1984)		Cetin et al. (2016)	Idriss&Boulanger (2010)	Seed et.al. (1984)
Liquefied?	Yes			D50	- ± -		
Data Class	B			% Fines	5.5 ± 6.9		
Critical Depth Range (m)	9.8 - 59.1			% PI	-		
Depth to GWT (ft)	4.8 ± 0.3						
σ_v (psf)	4085.6 ± 988.4			N	5.8 ± 1.8		
σ_v' (psf)	2237.0 ± 481.2			CR	1.00		
a_{max} (g)	0.214 ± 0.032			CS	1.00		
r_d	0.622 ± 0.131			CB	1.00		
CSR	0.158 ± 0.041			CE	1.22		
Equivalent Magnitude	7.7			CN	0.97		
MSF				$(N_1)_{60}$	6.9 ± 2.1		
CSRN							

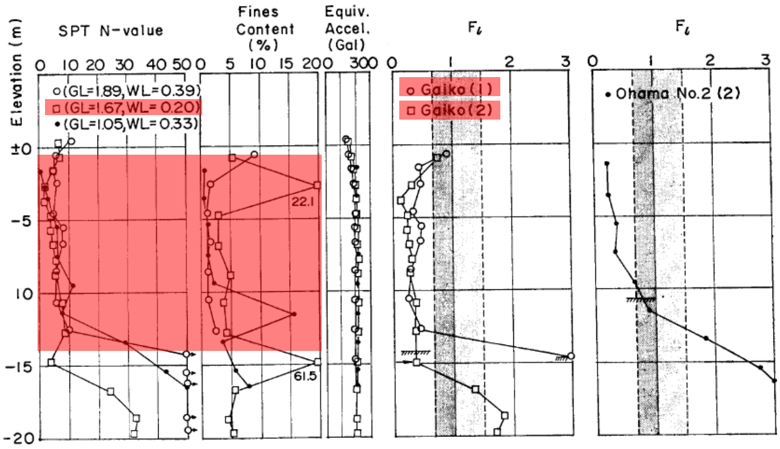

(a) Gaiko and Ohama No. 2 (Liquefied)

γ_m=110±3 pcf

γ_s=120±3 pcf

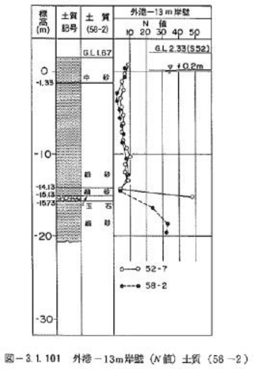

0-3	medium sand
3-15.8	sand
15.8-16.8	sand
16.8-17.4	**
17.4-22.7	sand

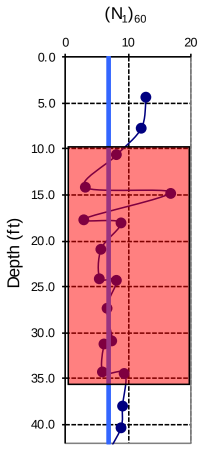

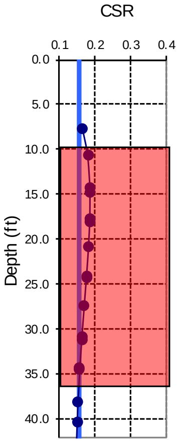

Depth (m)	Depth (ft)	CSR	N	C_N	C_R	$(N1)_{60}$	FC		
1.3	4.4	NA	7	2.00	0.76	12.9	-	medium sand	Gaiko-2
2.4	7.8	0.166	7	1.74	0.82	12.1	5.0	medium sand	Gaiko-2
3.3	10.7	0.181	5	1.56	0.86	8.1	-	sand	Gaiko-2
4.3	14.2	0.188	2	1.41	0.90	3.1	22.0	sand	Gaiko-2
4.5	14.8	0.188	11	1.39	0.90	16.8	-	-	Gaiko-1
5.4	17.6	0.188	2	1.29	0.93	2.9	-	sand	Gaiko-2
5.5	18.0	0.187	6	1.28	0.94	8.7	9.0	-	Gaiko-1
6.4	20.9	0.184	4	1.21	0.96	5.6	3.0	sand	Gaiko-2
7.4	24.1	0.178	4	1.13	0.99	5.4	-	sand	Gaiko-2
7.4	24.3	0.178	6	1.13	0.99	8.2	2.0	-	Gaiko-1
8.3	27.4	0.171	5	1.08	1.00	6.5	3.0	sand	Gaiko-2
9.4	30.9	0.164	6	1.02	1.00	7.4	-	sand	Gaiko-2
9.5	31.2	0.164	5	1.02	1.00	6.2	1.0	-	Gaiko-1
10.5	34.4	0.158	5	0.97	1.00	5.9	3.0	sand	Gaiko-2
10.5	34.4	0.158	8	0.97	1.00	9.5	-	-	Gaiko-1
11.6	38.1	0.153	8	0.93	1.00	9.1	2.0	-	Gaiko-1
12.3	40.4	0.150	8	0.91	1.00	8.8	4.0	sand	Gaiko-2
13.5	44.3	0.147	6	0.87	1.00	6.3	1.0	-	Gaiko-1
14.4	47.2	0.145	9	0.84	1.00	9.2	4.0	sand	Gaiko-2
15.7	51.5	0.143	6	0.81	1.00	5.9	1.0	-	Gaiko-1
16.4	53.8	0.143	4	0.79	1.00	3.9	20.0	sand	Gaiko-2
17.5	57.4	0.142	10	0.77	1.00	9.4	2.0	-	Gaiko-1
18.4	60.5	0.142	24	0.75	1.00	22.0	6.0	sand	Gaiko-2
20.3	66.5	0.142	33	0.72	1.00	28.9	4.0	sand	Gaiko-2
21.3	70.0	0.142	32	0.70	1.00	27.4	5.0	sand	Gaiko-2
					Mean:	6.9	5.5		
					St. Dev	2.1	6.9		

**Case number:**	100
**Earthquake:**	1983 Nihonkai-Chubu
**Magnitude:**	7.7 (M)
**Location:**	Gaiko Wharf B-2
**References:**	Hamada (1992)
**Nature of Failure:**	Lateral spreading, sand boils, ground fissures
**Comments:**	The Nihonkai-chubu Earthquake, with a magnitude of 7.7 occurred on May 26, 1983 in the northwest Japan and produced liquefaction sites in the coastal plain of Akita and Aomori Prefectures. About one month later, a magnitude 7.1 earthquake again hit the area . Akita Harbor suffered severe damage to its quay walls due to liquefaction. At Gaiko, which is an area newly reclaimed from the sea, liquefaction-induced ground displacements were observed. The quay wall, made of concrete caisson moved about 1.5 m and was tilted about 3 degrees The maximum surface acceleration at Akita City 115 km from the epicenter was recorded as 0.235 and 0.219 g.

Summary of Data

	Cetin et al. (2016)	Idriss&Boulanger (2010)	Seed et.al. (1984)		Cetin et al. (2016)	Idriss&Boulanger (2010)	Seed et.al. (1984)
Liquefied?	Yes	Yes		D_{50}	0.250 ± 0.050		
Data Class	B			% Fines	1.0 ± 2.0	1.0	
Critical Depth Range	8.2 - 41.0	24.6		% PI			
Depth to GWT (ft)	1.3 ± 0.3	1.3					
σ_v (psf)	2940.0 ± 659.9	2568.9		N	7.8 ± 1.9	7.7	
σ_v' (psf)	1484.4 ± 323.0	1106.9		C_R	0.99	0.95	
a_{max} (g)	0.227 ± 0.068	0.23		C_S	1.00	1.00	
r_d	0.82 ± 0.099	0.94		C_B	1.00	1.00	
CSR	0.239 ± 0.078	0.320		C_E	1.22	1.22	
Equivalent Magnitude	7.7	7.7		C_N	1.19	1.39	
MSF		0.95		$(N_1)_{60}$	11.2 ± 2.7	12.4	
CSRN		0.317					

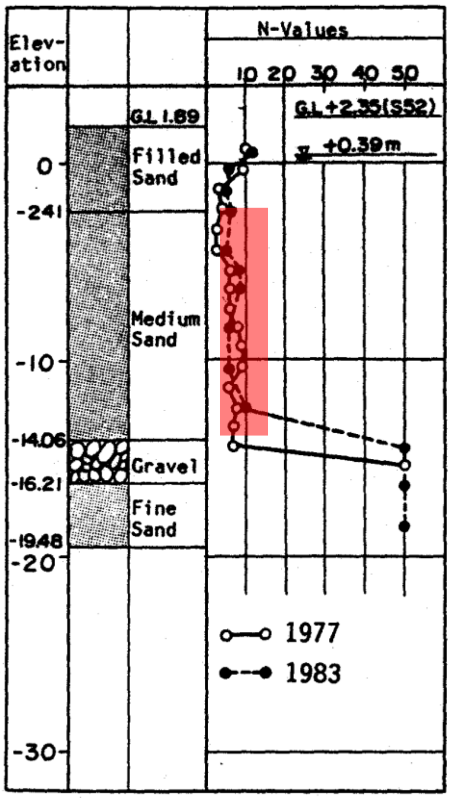

γ_m=110±3 pcf

γ_s=120±3 pcf

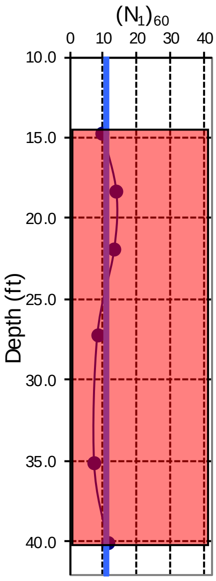

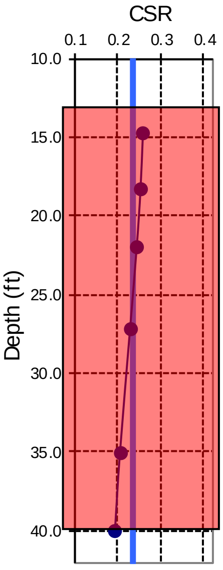

Depth (m)	Depth (ft)	CSR	N	C_N	C_R	$(N1)_{60}$
2.4	7.9	0.256	7.0	2.00	0.82	***13.9***
4.5	14.8	0.260	6.0	1.52	0.90	***10.0***
5.6	18.4	0.255	9.0	1.37	0.94	***14.1***
6.7	22.0	0.246	9.0	1.26	0.97	***13.4***
8.3	27.2	0.231	6.0	1.14	1.00	***8.3***
10.7	35.1	0.208	6.0	1.01	1.00	***7.3***
12.2	40.0	0.197	10	0.94	1.00	***11.5***
					**Mean:**	11.2
					**St. Dev.**	2.7

Case number: 101
Earthquake: 1983 M=7.7 Nihonka-chubu
Magnitude: 7.7 (Mw by USGS, NISEE)
Location: Hakodate
References: Iai. et. al. (1989)

Nature of Failure: -

Comments:

In Table 1 additional investigated sites where strong-motion observation stations exist are tabulated. In Figure 12 location of investigated site and epicenters of the earthquakes are shown. Hakodate site is a non-liquefied site.
In Hakodate maximum accelereation values measured as 48 gals (NS) and 52 gals (EW) geometric mean of which is 0.051 g.
SPT procedures were not clearly documented. However, on page 66 in Iai et. al. for comparison with Seed et. al. 1985, an average Japanese SPT energy ratio of 73% was reported to be used. On the basis of this, with some uncertainty Iai et. al. N values may be adopted as N_{73} values. Note that Seed et. al. (1985) adopted an energy ratio of 73% for liquefied cases.
The rationale behind this choice could be field data which is unknown to us or simply a conservative engineering judgement. Soil profile data is missing just N values.
FC values exist for three depth moreover the values reflects clayey behaviour.
From Technical Report of the Port and Horbour Research Institute Ministry of Transport, Japan No. 511 Marc. 1985 the borehole data could not be obtained.
There is an ambiguity if the site is free field or not in the vicinty of Pacific Ocean.
In addition the location of the site may not be correctly located in Figure 12 since the scale is very small and the coordinates of the borehole are not available.
The location of the station could not be found.
By taking into account the above mentioned reasons the site is rejected.

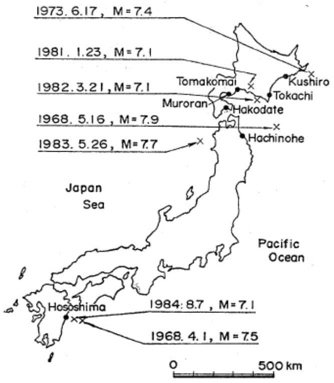

Fig. 12. Locations of other investigated ports and the epicenters of the earthquakes

Table 1. Strong-motion observation stations and earthquakes

Investigated Sites	Max. Accel. (Gal) NS	EW	UD	Accelerograph	Epicentral Distance (km)	Magnitude	Date of Earthquake
Hachinohe	233	181	119	SMAC-B 2	178	7.9	May 16, 1968**
Hakodate	48*	52*	36*	ERS	211	7.7	May 26, 1983***
Hososhima	182	242	48	SMAC-B 2	89	7.5	April 1, 1968
Hososhima	190	268	102	SMAC-B 2	47	7.1	Aug. 7, 1984
Kushiro	166	120	51	SMAC-B 2	127	7.4	June 17, 1973****
Muroran	204	135	70	SMAC-B 2	280	7.9	May 16, 1968**
Tokachi	113*	168*	46*	ERS	64	7.1	March 21, 1982*****
Tomakomai	161	115	20	SMAC-B 2	50	7.1	Jan. 23, 1981

* SMAC-B 2 Equivalent (i.e. modified, by digital filtering technique, into the maximum acceleration which would be obtained by the SMAC-B 2 accelerograph)
** 1968 Tokachi-Oki Earthquake
*** 1983 Nihonkai-Chubu Earthquake
**** 1973 Nemuro-Hanto-Oki Earthquake
***** 1982 Urakawa-Oki Earthquake

Summary of Data

	Cetin et al. (2016)	Idriss&Boulanger (2010)	Seed et.al. (1984)		Cetin et al. (2016)	Idriss&Boulanger (2010)	Seed et.al. (1984)
Liquefied?	No	No		D_{50}	- ± -	-	
Data Class	A	-		% Fines	66.0 ± 45.3	66.0	
Critical Depth Range	8.2 - 26.2	14.1		% PI	-	-	
Depth to GWT (ft)	5.2 ± 3.0	5.2					
σ_v (psf)	2066.9 ± 363.0	1691.7		N	3.7 ± 1.5	2.6	
σ_v' (psf)	1319.7 ± 258.1	1127.8		C_R	0.93	0.95	
a_{max} (g)	0.051 ± 0.003	0.052		C_S	1.00	1.00	
r_d	0.917 ± 0.073	0.970		C_B	1.00	1.00	
CSR	0.048 ± 0.008	0.049		C_E	1.22	1.22	
Equivalent Magnitude	7.7	7.7		C_N	1.27	1.41	
MSF		0.95		$(N_1)_{60}$	5.2 ± 2.1	4.2	
CSRN		0.05					

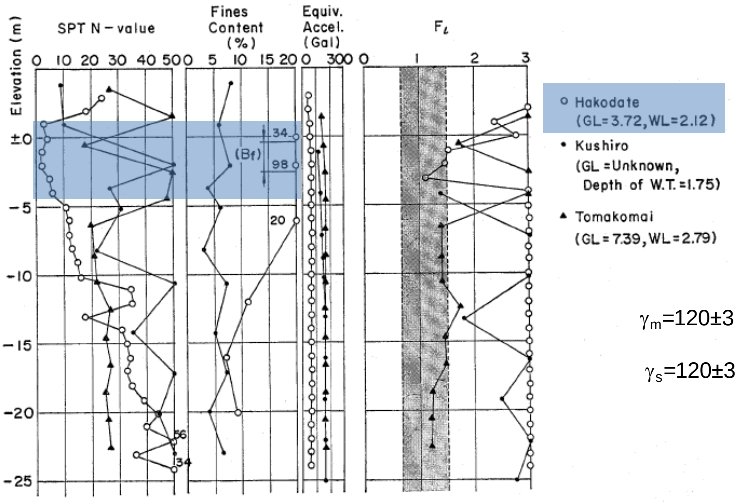

γ_m=120±3 pcf

γ_s=120±3 pcf

(b) Hakodate, Kushiro, and Tomakomai (Nonliquefied)

Fig. 13. Ground conditions and resulted liquefaction potentials at other strong-motion observation stations

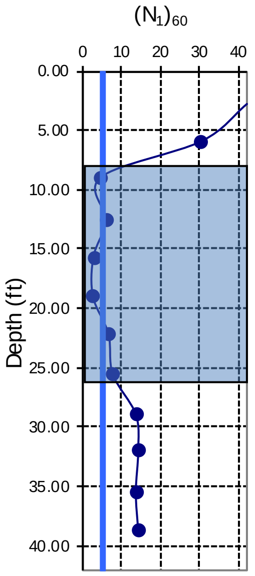

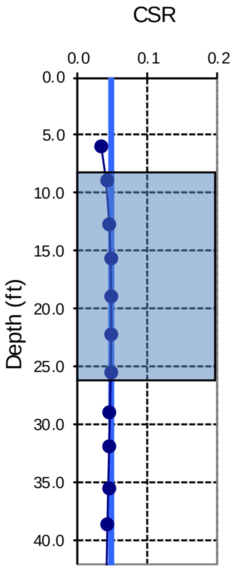

Depth (m)	Depth (ft)	CSR	N	C_N	C_R	$(N1)_{60}$	FC
0.86	2.81	NA	24	2.00	0.72	42.3	-
1.81	5.94	0.035	18	1.78	0.79	30.6	-
2.74	8.98	0.041	3	1.58	0.83	***4.8***	***-***
3.85	12.63	0.045	4	1.42	0.88	***6.1***	***34***
4.78	15.69	0.047	2	1.31	0.91	***2.9***	***-***
5.79	19.00	0.048	2	1.22	0.94	***2.8***	***98***
6.75	22.16	0.048	5	1.15	0.97	***6.8***	***-***
7.78	25.54	0.047	6	1.08	1.00	***7.9***	***-***
8.81	28.91	0.046	11	1.03	1.00	13.8	-
9.74	31.97	0.045	12	0.99	1.00	14.4	20
10.81	35.46	0.044	12	0.94	1.00	13.8	-
11.79	38.67	0.043	13	0.91	1.00	14.4	-
12.80	42.00	0.042	15	0.88	1.00	16.0	-
13.92	45.67	0.041	16	0.85	1.00	16.5	-
14.78	48.48	0.041	35	0.82	1.00	35.1	-
15.83	51.94	0.040	35	0.80	1.00	34.0	-
					**Mean:**	**5.2**	**66.0**
					**St. Dev.**	**2.1**	**45.3**

Case number: 102
Earthquake: 1983 Nihonkai-Chubu
Magnitude: 7.7 (Mw by USGS, NISEE)
Location: Nakajima No. 1 (5)
References: Iai. et. al. (1989)

Nature of Failure: Liquefied and nonliquefied areas are defined by the boundary lines between artifically filled areas and the areas of the Quaternary sediments which are shown in photos provided by Iai et. al. (1989)

Comments:
Tsuchida et. al (1985) shows the liquefied and non liquefied sites on Akita Port in Figure 8.
Nakajima No. 1 (5) is 1.03 km away from strong ground motion station.
SMAC_B2 type accelerograph measured an acceleration value of 190 and 205 Gals in NS and EW directions. "These two values are used for computing the rock outcrop motion. Then this rock outcrop motion is used as input for the earthquake response analysis at several sites." (Kurata et. al. 1983)
In Figure 8 three digit numbers shows the maximum acceleration values obtained from ground response analysis. However this procedure is done for one component which is 209 Gals (EW) In order ro take into account multi-directional effects site response by Iai et.al. was scaled, maximum acceleration values of 209 and 199 Gals are obtained.
Geometric mean of 209 and 199 Gals is equal to 0.208 g is assigned to site.
SPT procedures were not clearly documented. However, on page 66 in Iai et. al. for comparison with Seed et. al. 1985, an average Japanese SPT energy ratio of 73% was reported to be used. On the basis of this, with some uncertainty Iai et. al. N values may be adopted as N73 values. Note that Seed et. al. (1985) adopted an energy ratio of 73% for liquefied cases.
The rationale behind this choice could be field data which is unknown to us or simply a conservative engineering judgement.
From Technical Report of the Port and Horbour Research Institute Ministry of Transport, Japan No. 511 Marc. 1985 the borehole data is obtained.
Site response by Iai et.al. Exists.
There is an ambiguity if the site is free field or not in the vicinty of Akita Port Channel which is 10 m in depth.
From Figure 8 the site is 22 m away from channel.
Note that the location of the site may not be correctly located in Figure 8 since the scale is very small and the coordinates of the borehole are not available.
Additionally Quay walls are believed to be located 22 m away from site.
Presence of Quay walls and due to proximity to the Akita Port Channel which 10 m free field stress boundary conditions may not be fulfilled. Hence cases within 20 m to the Quay walls and channel are eliminated.
There exist two possibly liqueifed layer.

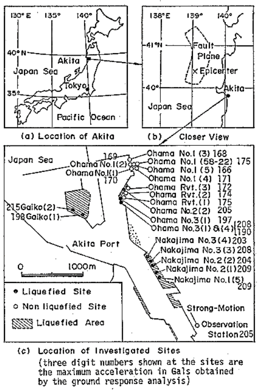

Fig. 8. Investigated sites in Akita Port

Summary of Data

	Cetin et al. (2016)	Idriss&Boulanger (2010)	Seed et.al. (1984)		Cetin et al. (2016)	Idriss&Boulanger (2010)	Seed et.al. (1984)
Liquefied?	Yes	Yes		D_{50}	- ± -	-	
Data Class	B	-		% Fines	14.6 ± 17.4	8.0	
Critical Depth Range	6.6 - 42.7	21.2		% PI	-	-	
Depth to GWT (ft)	5.8 ± 0.3	4.8					
σ_v (psf)	2895.0 ± 724.2	2589.8		N	6.5 ± 2.2	7.3	
σ_v' (psf)	1719.9 ± 351.8	1545.5		C_R	0.99	0.95	
a_{max} (g)	0.208 ± 0.031	0.205		C_S	1.00	1.00	
r_d	0.790 ± 0.099	0.950		C_B	1.00	1.00	
CSR	0.180 ± 0.036	0.210		C_E	1.22	1.22	
Equivalent Magnitude	7.7	7.7		C_N	1.11	1.18	
MSF		0.95		$(N_1)_{60}$	8.7 ± 3.0	9.9	
CSRN		0.22					

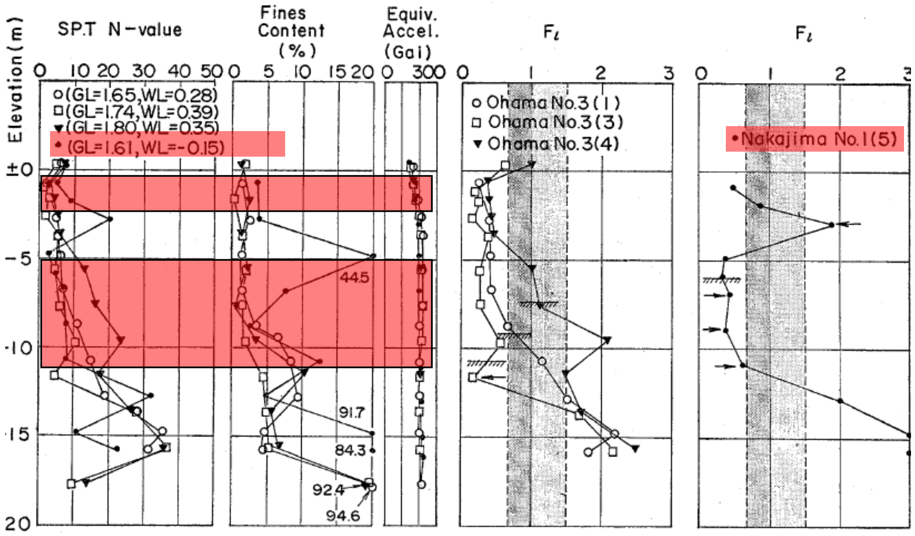

(b) Ohama No. 3 and Nakajima No. 1 (Liquefied)

γ_m=110±3 pcf

γ_s=120±3 pcf

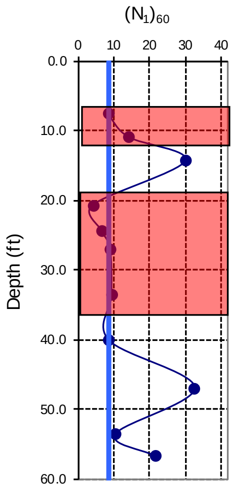

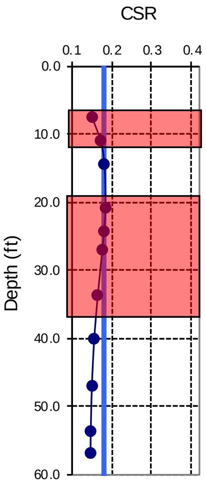

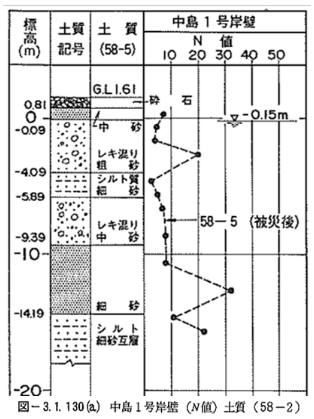

sand	0.8-1.61
sand-gravel	1.61-5.7
silt-sand	5.7-7.5
sand-gravel	7.5-11.0
sand	11.0-15.8
silt-sand	15.8-20.0

Depth (m)	Depth (ft)	CSR	N	C_N	C_R	$(N1)_{60}$	FC	
1.3	4.4	NA	8.0	2.00	0.76	***14.7***	***3.0***	sand
2.3	7.6	0.150	5.0	1.69	0.81	***8.4***	***4.0***	sand-gravel
3.3	11.0	0.171	9.0	1.51	0.86	***14.2***	***-***	sand-gravel
4.4	14.4	0.181	20.0	1.37	0.90	***30.0***	***4.0***	sand-gravel
6.3	20.8	0.184	3.0	1.19	0.96	***4.2***	***45.0***	silt-sand
7.4	24.4	0.180	5.0	1.11	0.99	***6.7***	***-***	silt-sand
8.2	27.0	0.176	7.0	1.07	1.00	***9.1***	***8.0***	silt-sand
10.3	33.7	0.165	8.0	0.97	1.00	***9.5***	***3.0***	sand-gravel
12.2	40.2	0.156	8.0	0.90	1.00	***8.8***	***13.0***	sand
14.3	47.0	0.150	32.0	0.84	1.00	***32.7***	***5.0***	sand
16.4	53.7	0.147	11.0	0.79	1.00	***10.6***	***91.7***	silt-sand
17.3	56.8	0.147	23.0	0.77	1.00	***21.5***	***84.3***	silt-sand
					**Mean:**	**8.7**	**14.6**	
					**St. Dev.**	**3.0**	**17.4**	

**Case number:**	103
**Earthquake:**	1983 Nihonkai-Chubu
**Magnitude:**	7.7 (Mw by USGS, NISEE)
**Location:**	Nakajima No. 2 (1)
**References:**	Iai. et. al. (1989)
**Nature of Failure:**	Liquefied and nonliquefied areas are defined by the boundary lines between artifically filled areas and the areas of the Quaternary sediments which are shown in photos provided by Iai et. al. (1989)
**Comments:**	Tsuchida et. al (1985) shows the liquefied and non liquefied sites on Akita Port in Figure 8.<br>Nakajima No. 2 (1) is 1.12 km away from strong ground motion station.<br>SMAC_B2 type accelerograph measured an acceleration value of 190 and 205 Gals<br>in NS and EW directions. "These two values are used for computing the rock outcrop motion.<br>Then this rock outcrop motion is used as input for the earthquake<br>response analysis at several sites." (Kurata et. Al. 1983)<br>In Figure 8 three digit numbers shows the maximum acceleration<br>values obtained from ground response analysis. However this<br>procedure is done for one component which is 209 Gals (EW) In order ro take into account multi-directional<br>effects site response by Iai et.al. was scaled, maximum acceleration values of 209 and 199 Gals are obtained.<br>Geometric mean of 209 and 199 Gals is equal to 0.208 g is assigned to site.<br>SPT procedures were not clearly documented. However, on page 66<br>in Iai et. al. for comparison with Seed et. al. 1985,<br>an average Japanese SPT energy ratio of 73% was reported<br>to be used. On the basis of this, with some uncertainty Iai et. al.<br>N values may be adopted as N73 values. Note that Seed et. al. (1985)<br>adopted an energy ratio of 73% for liquefied cases.<br>The rationale behind this choice could be field data which is unknown to us or simply<br>a conservative engineering judgement. Site response by Iai et.al. exists.<br>From Technical Report of the Port and Horbour Research Institute Ministry of Transport,<br>Japan No. 511 Marc. 1985 the borehole data is obtained.<br>There is an ambiguity if the site is free field or not in the vicinty<br>of Akita Port Channel which is 10 m in depth.<br>From Figure 8 the site is 32 m away from channel.<br>Note that the location of the site may not be correctly located in Figure 8<br>since the scale is very small and the coordinates of the borehole are not available.<br>Additionally Quay walls are believed to be located 32 m away from site.<br>Presence of Quay walls and due to proximity to the Akita Port Channel which 10 m free<br>field stress boundary conditions may not be fulfilled. Hence cases within 20 m to the<br>Quay walls and channel are eliminated.<br>In Iai et.al. first SPT-N value is missing hence It is taken from<br>Technical Report of the Port and Horbour Research Institute Ministry of Transport.

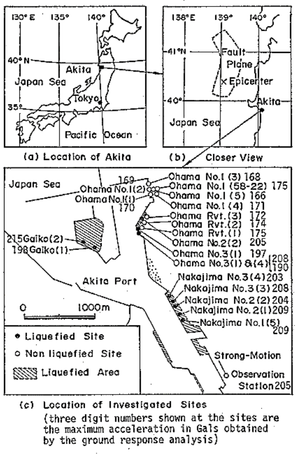

Fig. 8. Investigated sites in Akita Port

**Summary of Data**							
	Cetin et al. (2016)	Idriss&Boulanger (2010)	Seed et.al. (1984)		Cetin et al. (2016)	Idriss&Boulanger (2010)	Seed et.al. (1984)
Liquefied?	Yes	Yes		D_{50}	- ± -	-	
Data Class	A	-		% Fines	2.0 ± 1.0	3.0	
Critical Depth Range	6.6 - 29.5	23.4		% PI	-	-	
Depth to GWT (ft)	4.8 ± 0.3	4.8					
σ_v (psf)	2231.8 ± 480.3	2840.4		N	4.7 ± 1.4	10.4	
σ_v' (psf)	1402.7 ± 243.9	1691.7		C_R	0.94	0.95	
a_{max} (g)	0.207 ± 0.031	0.205		C_S	1.00	1.00	
r_d	0.904 ± 0.076	0.940		C_B	1.00	1.00	
CSR	0.193 ± 0.034	0.213		C_E	1.22	1.22	
Equivalent Magnitude	7.7	7.7		C_N	1.23	1.12	
MSF		0.95		$(N_1)_{60}$	6.6 ± 2.0	13.5	
CSRN		0.22					

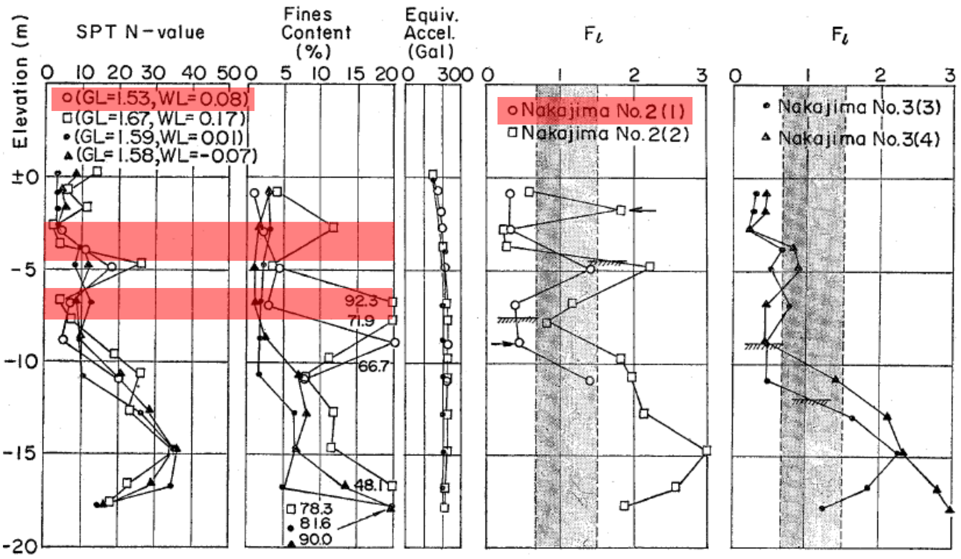

γ_m=120±3 pcf

γ_s=125±3 pcf

(c) Nakajima No.2 and No.3 (Liquefied)

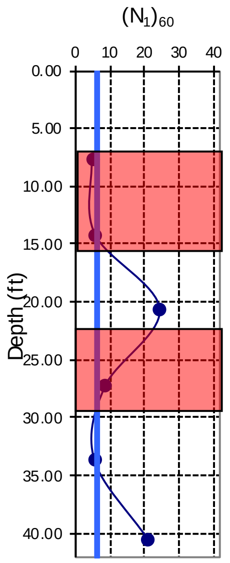

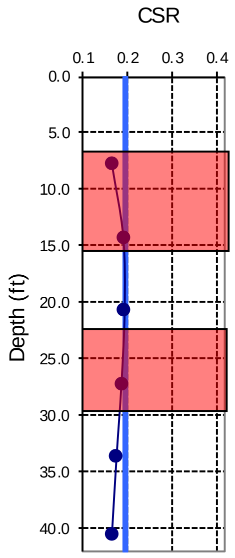

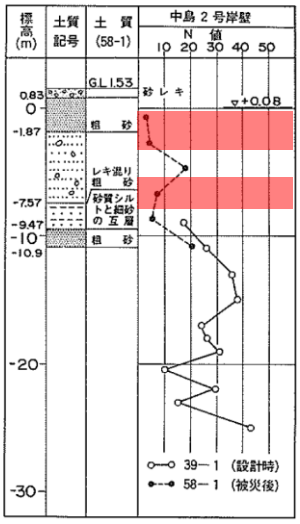

図−3.1.129 中島2号岸壁（N値）土質（58−1）

0-0.7	
0.7-3.40	sand
3.40-9.10	sand gravel
9.10-11.00	silt sand
11.00-12.43	sand

Depth (m)	Depth (ft)	CSR	N	C_N	C_R	$(N1)_{60}$	FC	
2.36	7.74	0.163	3	1.67	0.82	***5.0***	***1.0***	sand
4.32	14.18	0.190	4	1.35	0.90	***5.9***	***2.0***	sand gravel
6.31	20.69	0.193	18	1.16	0.96	*24.4*	*4.0*	sand gravel
8.32	27.30	0.186	7	1.03	1.00	***8.8***	***3.0***	sand gravel
10.25	33.64	0.175	5	0.94	1.00	*5.7*	*66.7*	silt sand
12.35	40.53	0.164	20	0.87	1.00	*21.1*	*8.0*	sand
					**Mean:**	6.6	2.0	
					**St. Dev.**	2.0	1.0	

**Case number:**	104
**Earthquake:**	1983 Nihonkai-Chubu
**Magnitude:**	7.7 (Mw by USGS, NISEE)
**Location:**	Nakajima No. 2 (2)
**References:**	Iai. et. al. (1989)
**Nature of Failure:**	Liquefied and nonliquefied areas are defined by the boundary lines between artifically filled areas and the areas of the Quaternary sediments which are shown in photos provided by Iai et. al. (1989)
**Comments:**	Tsuchida et. al (1985) shows the liquefied and non liquefied sites on Akita Port in Figure 8. Nakajima No. 2 (2) is 1.24 km away from strong ground motion station. SMAC_B2 type accelerograph measured an acceleration value of 190 and 205 Gals in NS and EW directions. "These two values are used for computing the rock outcrop motion. Then this rock outcrop motion is used as input for the earthquake response analysis at several sites." (Kurata et. Al. 1983) In Figure 8 three digit numbers shows the maximum acceleration values obtained from ground response analysis. However this procedure is done for one component which is 204 Gals (EW) In order ro take into account multi-directional effects site response by Iai et.al. was scaled, maximum acceleration values of 204 and 194 Gals are obtained. Geometric mean of 204 and 194 Gals is equal to 0.203 g is assigned to site. SPT procedures were not clearly documented. However, on page 66 in Iai et. al. for comparison with Seed et. al. 1985, an average Japanese SPT energy ratio of 73% was reported to be used. On the basis of this, with some uncertainty Iai et. al. N values may be adopted as N_{73} values. Note that Seed et. al. (1985) adopted an energy ratio of 73% for liquefied cases. The rationale behind this choice could be field data which is unknown to us or simply From Technical Report of the Port and Horbour Research Institute Ministry of Transport, Japan No. 511 Marc. 1985 the borehole data is obtained. Site response by Iai et.al. exists. There is an ambiguity if the site is free field or not in the vicinty of Akita Port Channel which is 10 m in depth. From Figure 8 the site is 37 m away from channel. Note that the location of the site may not be correctly located in Figure 8 since the scale is very small and the coordinates of the borehole are not available. Additionally Quay walls are believed to be located 37 m away from site. Presence of Quay walls and due to proximity to the Akita Port Channel which 10 m free field stress boundary conditions may not be fulfilled. Hence cases within 20 m to the Quay walls and channel are eliminated.

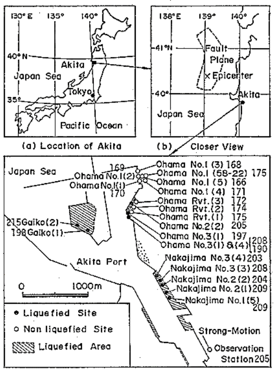

(c) Location of Investigated Sites
(three digit numbers shown at the sites are the maximum acceleration in Gals obtained by the ground response analysis)

Fig. 8. Investigated sites in Akita Port

Summary of Data

	Cetin et al. (2016)	Idriss&Boulanger (2010)	Seed et.al. (1984)		Cetin et al. (2016)	Idriss&Boulanger (2010)	Seed et.al. (1984)
Liquefied?	Yes	Yes		D_{50}	- ± -	-	
Data Class	A	-		% Fines	8.0 ± 5.7	7.0	
Critical Depth Range	6.6 - 18.7	12.4		% PI	-	-	
Depth to GWT (ft)	4.9 ± 0.3	4.9					
σ_v (psf)	1515.7 ± 244.3	1482.9		N	5.9 ± 4.0	6.0	
σ_v' (psf)	1034.6 ± 121.2	1002.5		C_R	0.88	0.85	
a_{max} (g)	0.203 ± 0.030	0.205		C_S	1.00	1.00	
r_d	0.961 ± 0.056	0.980		C_B	1.00	1.00	
CSR	0.186 ± 0.031	0.191		C_E	1.22	1.22	
Equivalent Magnitude	7.7	7.7		C_N	1.43	1.50	
MSF		1.0		$(N_1)_{60}$	9.0 ± 6.2	9.3	
CSRN		0.2					

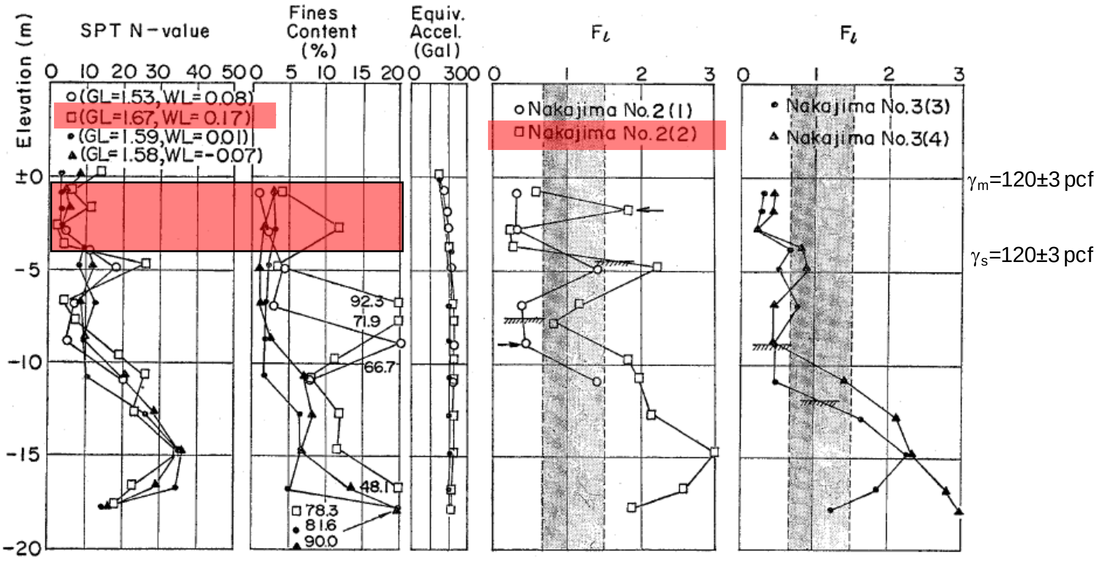

(c) Nakajima No. 2 and No. 3 (Liquefied)

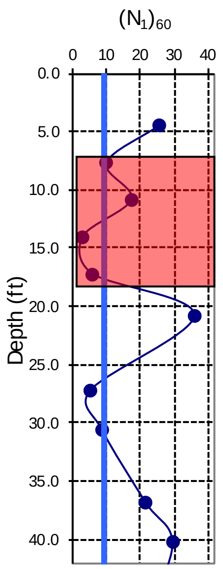

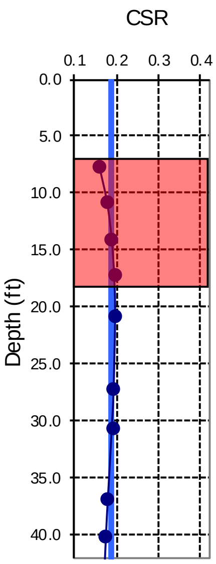

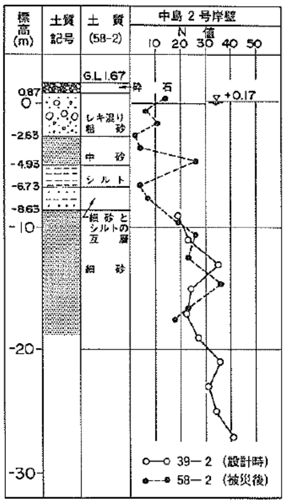

図－3.1.130(b) 中島1号岸壁 (N値) 土質 (58－2)

0.8-4.3	sand gravel
4.3-6.6	sand
6.6-8.4	silt
8.4-10.3	silt sand
10.3-...	sand

Depth (m)	Depth (ft)	CSR	N	C_N	C_R	$(N1)_{60}$	FC	
1.4	4.5	NA	14	1.98	0.76	*25.7*	-	sand gravel
2.3	7.7	0.159	6	1.68	0.82	***10.0***	***4.0***	sand gravel
3.3	10.8	0.179	11	1.51	0.86	***17.3***	**-**	sand gravel
4.3	14.0	0.190	2	1.38	0.90	***3.0***	***12.0***	sand gravel
5.2	17.2	0.196	4	1.28	0.93	***5.8***	**-**	sand
6.3	20.8	0.198	26	1.19	0.96	*36.0*	*3.0*	sand
8.3	27.3	0.195	4	1.06	1.00	*5.2*	*20.0*	silt
9.3	30.6	0.190	7	1.01	1.00	*8.6*	*20.0*	silt sand
11.2	36.9	0.181	19	0.93	1.00	*21.6*	*11.0*	sand
12.3	40.2	0.176	27	0.90	1.00	*29.5*	*8.0*	sand
14.3	46.9	0.168	24	0.84	1.00	*24.5*	*12.0*	sand
16.5	54.0	0.163	35	0.79	1.00	*33.5*	*12.0*	sand
18.2	59.8	0.162	23	0.75	1.00	*21.0*	*20.0*	sand
19.2	63.1	0.161	18	0.73	1.00	*16.0*	-	sand
					**Mean:**	9.0	8.0	
					**St. Dev.**	6.2	5.7	

**Case number:**	105
**Earthquake:**	1983 Nihonkai-Chubu
**Magnitude:**	7.7 (Mw by USGS, NISEE)
**Location:**	Nakajima No. 3 (3)
**References:**	Iai. et. al. (1989)
**Nature of Failure:**	Liquefied and nonliquefied areas are defined by the boundary lines between artifically filled areas and the areas of the Quaternary sediments which are shown in photos provided by Iai et. al. (1989)
**Comments:**	Tsuchida et. al (1985) shows the liquefied and non liquefied sites on Akita Port in Figure 8. Nakajima No. 3 (3) is 1.3 km away from strong ground motion station. SMAC_B2 type accelerograph measured an acceleration value of 190 and 205 Gals in NS and EW directions. "These two values are used for computing the rock outcrop motion. Then this rock outcrop motion is used as input for the earthquake response analysis at several sites." (Kurata et. Al. 1983) In Figure 8 three digit numbers shows the maximum acceleration values obtained from ground response analysis. However this procedure is done for one component which is 208 Gals (EW) In order ro take into account multi-directional effects site response by Iai et.al. was scaled, maximum acceleration values of 208 and 198 Gals are obtained. Geometric mean of 208 and 198 Gals is equal to 0.207 g is assigned to site. SPT procedures were not clearly documented. However, on page 66 in Iai et. al. for comparison with Seed et. al. 1985, an average Japanese SPT energy ratio of 73% was reported to be used. On the basis of this, with some uncertainty Iai et. al. N values may be adopted as N_{73} values. Note that Seed et. al. (1985) adopted an energy ratio of 73% for liquefied cases. The rationale behind this choice could be field data which is unknown to us or simply a conservative engineering judgement. From Technical Report of the Port and Horbour Research Institute Ministry of Transport, Japan No. 511 Marc. 1985 the borehole data is obtained. Site response by Iai et.al. exists. There is an ambiguity if the site is free field or not in the vicinty of Akita Port Channel which is 10 m in depth. From Figure 8 the site is 47 m away from channel. Note that the location of the site may not be correctly located in Figure 8 since the scale is very small and the coordinates of the borehole are not available. Additionally Quay walls are believed to be located 47 m away from site. Presence of Quay walls and due to proximity to the Akita Port Channel which 10 m free field stress boundary conditions may not be fulfilled. Hence cases within 20 m to the Quay walls and channel are eliminated.

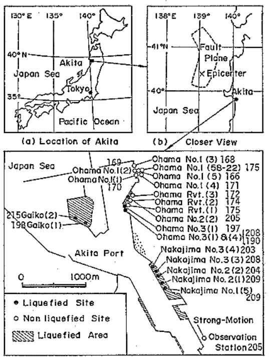

(c) Location of Investigated Sites
(three digit numbers shown at the sites are the maximum acceleration in Gals obtained by the ground response analysis)

Fig. 8. Investigated sites in Akita Port

Summary of Data

	Cetin et al. (2016)	Idriss&Boulanger (2010)	Seed et.al. (1984)		Cetin et al. (2016)	Idriss&Boulanger (2010)	Seed et.al. (1984)
Liquefied?	Yes	Yes		D_{50}	- ± -	-	
Data Class	A	-		% Fines	3.0 ± 2.0	2.0	
Critical Depth Range	3.3 - 12.5	19.8		% PI	-	-	
Depth to GWT (ft)	5.2 ± 0.3	5.2					
σ_v (psf)	814.3 ± 169.4	2401.8		N	3.0 ± 0.1	7.3	
σ_v' (psf)	646.4 ± 76.6	1482.9		C_R	0.82	0.95	
a_{max} (g)	0.207 ± 0.031	0.205		C_S	1.00	1.00	
r_d	0.952 ± 0.037	0.960		C_B	1.00	1.00	
CSR	0.161 ± 0.030	0.205		C_E	1.22	1.22	
Equivalent Magnitude	7.7	7.7		C_N	1.81	1.21	
MSF		0.95		$(N_1)_{60}$	5.4 ± 0.2	10.2	
CSRN		0.21					

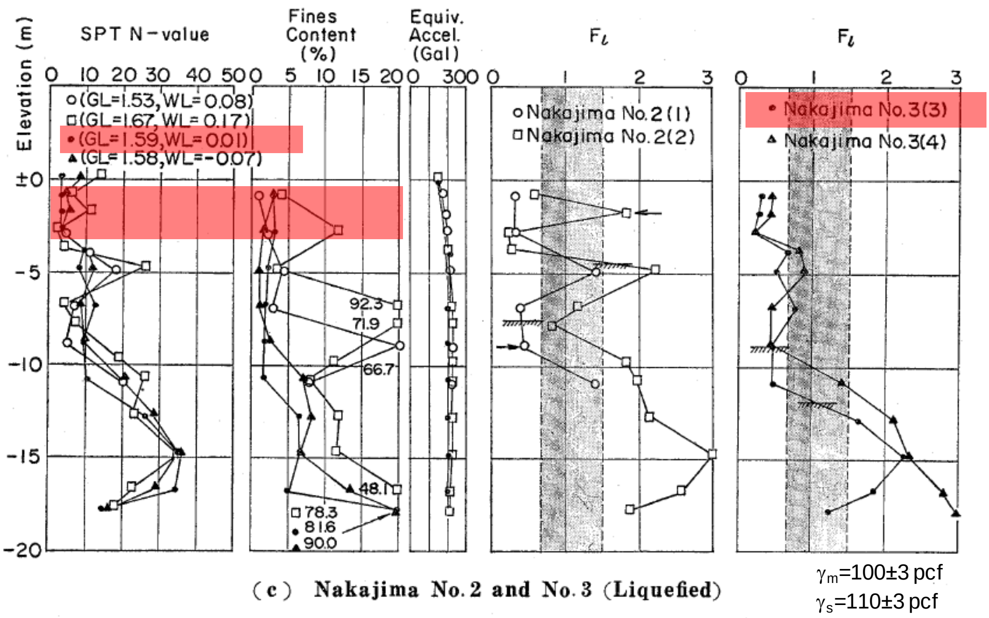

(c) Nakajima No. 2 and No. 3 (Liquefied)

γ_m=100±3 pcf
γ_s=110±3 pcf

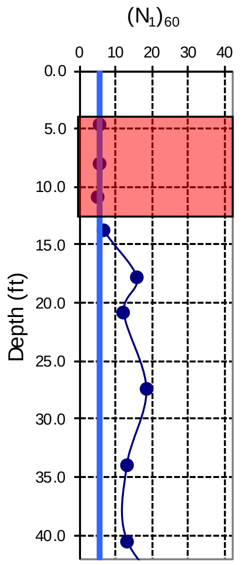

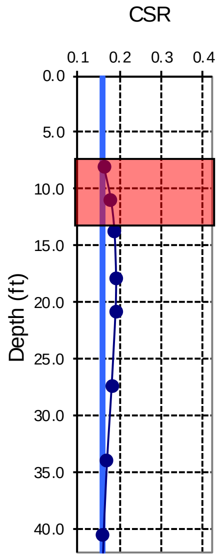

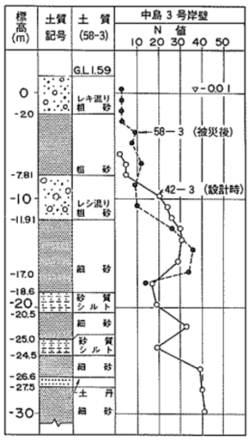

図−3.1.127 中島３号岸壁（N値）土質（58−3）

0-3.59	gravel sand
3.59-3.59	sand
9.4-13.5	gravel sand
13.5-20.19	sand
20.19-22.09	silt
22.09-26.09	sand
26.09-26.59	silt
26.59-28.19	sand
28.19-29.09	silt
29.09-31.59	sand

Depth (m)	Depth (ft)	CSR	N	C_N	C_R	$(N1)_{60}$	FC	
1.4	4.7	NA	3	2.00	0.76	***5.6***	-	gravel sand
2.5	8.1	0.163	3	1.80	0.82	***5.4***	***3.0***	gravel sand
3.3	10.9	0.181	3	1.64	0.86	***5.1***	-	gravel sand
4.2	13.7	0.190	4	1.51	0.89	***6.6***	***3.0***	sand
5.4	17.8	0.194	10	1.37	0.93	***15.6***	-	sand
6.4	20.9	0.193	8	1.29	0.96	***12.1***	***5.0***	sand
8.4	27.5	0.183	13	1.16	1.00	***18.3***	***7.0***	sand
10.4	34.0	0.171	10	1.06	1.00	***12.9***	***9.0***	gravel sand
12.4	40.6	0.162	11	0.98	1.00	***13.1***	***11.0***	gravel sand
14.4	47.1	0.157	27	0.92	1.00	***30.1***	***13.0***	sand
16.3	53.4	0.155	36	0.87	1.00	***38.0***	***15.0***	sand
18.3	60.0	0.154	35	0.82	1.00	***35.0***	***17.0***	sand
19.3	63.4	0.154	15	0.80	1.00	***14.6***	***18.0***	sand
					**Mean:**	**5.4**	**3.0**	
					**St. Dev.**	**0.2**	**2.0**	assigned

Case number: 106
Earthquake: 1983 Nihonkai-Chubu
Magnitude: 7.7 (Mw by USGS, NISEE)
Location: Nakajima No. 3 (4)
References: Iai. et. al. (1989)

Nature of Failure: Liquefied and nonliquefied areas are defined by the boundary lines between artifically filled areas and the areas of the Quaternary sediments which are shown in photos provided by Iai et. al. (1989)

Comments: Tsuchida et. al (1985) shows the liquefied and non liquefied sites on Akita Port in Figure 8. Nakajima No. 3 (4) is 1.40 km awayfrom strong ground motion station. SMAC_B2 type accelerograph measured an acceleration value of 190 and 205 Gals in NS and EW directions. "These two values are used for computing the rock outcrop motion. Then this rock outcrop motion is used as input for the earthquake response analysis at several sites." (Kurata et. Al. 1983) In Figure 8 three digit numbers shows the maximum acceleration values obtained from ground response analysis. However this procedure is done for one component which is 203 Gals (EW) In order ro take into account multi-directional effects site response by Iai et.al. was scaled, maximum acceleration values of 203 and 193 Gals are obtained. Geometric mean of 203 and 193 Gals is equal to 0.202 g is assigned to site. SPT procedures were not clearly documented. However, on page 66 in Iai et. al. for comparison with Seed et. al. 1985, an average Japanese SPT energy ratio of 73% was reported to be used. On the basis of this, with some uncertainty Iai et. al. N values may be adopted as N73 values. Note that Seed et. al. (1985) adopted an energy ratio of 73% for liquefied cases. The rationale behind this choice could be field data which is unknown to us or simply a conservative engineering judgement. From Technical Report of the Port and Horbour Research Institute Ministry of Transport, Japan No. 511 Marc. 1985 the borehole data is obtained. Site response by Iai et.al. exists. There is an ambiguity if the site is free field or not in the vicinty of Akita Port Channel which is 10 m in depth. From Figure 8 the site is 38 m away from channel. Note that the location of the site may not be correctly located in Figure 8 since the scale is very small and the coordinates of the borehole are not available. Additionally Quay walls are believed to be located 38 m away from site. Presence of Quay walls and due to proximity to the Akita Port Channel which 10 m free field stress boundary conditions may not be fulfilled. Hence cases within 20 m to the Quay walls and channel are eliminated.

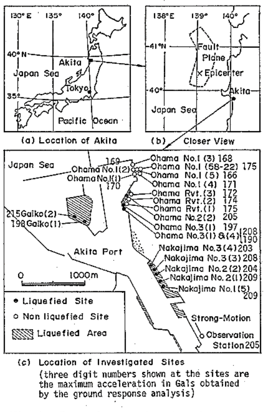

Fig. 8. Investigated sites in Akita Port

Summary of Data

	Cetin et al. (2016)	Idriss&Boulanger (2010)	Seed et.al. (1984)		Cetin et al. (2016)	Idriss&Boulanger (2010)	Seed et.al. (1984)
Liquefied?	Yes	Yes		D_{50}	- ± -	-	
Data Class	A	-		% Fines	2.0 ± 1.0	2.0	
Critical Depth Range	5.9 - 36.7	18.8		% PI	-	-	
Depth to GWT (ft)	5.4 ± 0.3	5.0					
σ_v (psf)	2504.9 ± 618.9	2276.5		N	7.8 ± 3.0	8.0	
σ_v' (psf)	1512.0 ± 300.7	1420.2		C_R	0.96	0.95	
a_{max} (g)	0.202 ± 0.030	0.205		C_S	1.00	1.00	
r_d	0.857 ± 0.087	0.960		C_B	1.00	1.00	
CSR	0.187 ± 0.035	0.206		C_E	1.22	1.22	
Equivalent Magnitude	7.7	7.7		C_N	1.18	1.24	
MSF		0.95		$(N_1)_{60}$	10.8 ± 4.2	11.5	
CSRN		0.21					

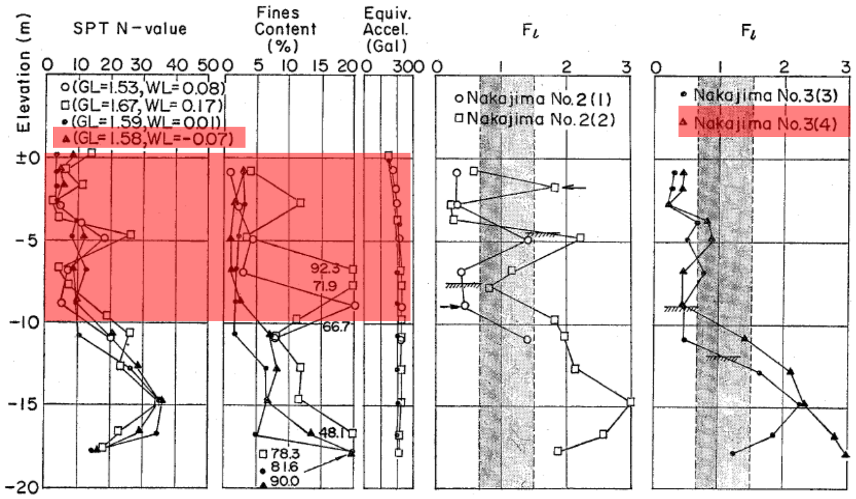

(c) Nakajima No. 2 and No. 3 (Liquefied)

γ_m=110±3pcf
γ_s=120±3 pcf

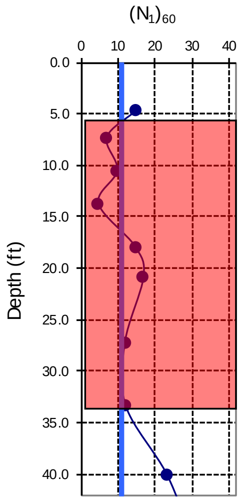

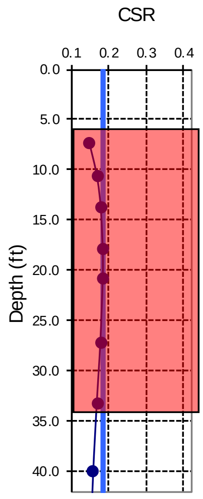

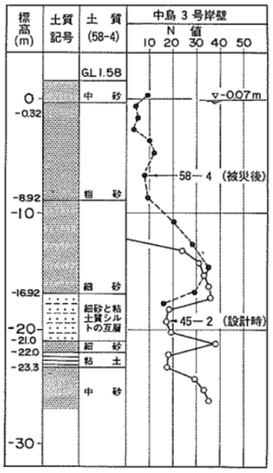

図−3. 1. 128 中島 3 号岸壁 (N値) 土質 (58−4)

0-1.9	medium sand
1.9-10.5	sand
10.5-18.5	sand
18.5-22.58	silt
22.58-23.58	sand
23.58-24.88	clay
24.88-31.58	sand

Depth (m)	Depth (ft)	CSR	N	C_N	C_R	$(N1)_{60}$	FC	
1.4	4.7	NA	8	2.00	0.76	*14.9*	-	medium sand
2.2	7.3	0.149	4	1.73	0.81	***6.8***	***3.0***	sand
3.2	10.5	0.170	6	1.54	0.86	***9.6***	***-***	sand
4.2	13.8	0.181	3	1.40	0.89	***4.6***	***2.0***	sand
5.5	17.9	0.187	10	1.27	0.94	***14.4***	***-***	sand
6.4	20.8	0.187	12	1.19	0.96	***16.7***	***1.0***	sand
8.3	27.2	0.180	9	1.07	1.00	***11.7***	***1.0***	sand
10.2	33.4	0.170	10	0.98	1.00	***11.9***	***3.0***	sand
12.2	40.1	0.160	21	0.90	1.00	*23.1*	*7.0*	sand
14.2	46.5	0.154	29	0.85	1.00	*29.8*	*8.0*	sand
16.3	53.4	0.150	37	0.79	1.00	*35.7*	*7.0*	sand
18.1	59.4	0.149	30	0.76	1.00	*27.6*	*14.0*	sand
19.3	63.3	0.148	17	0.73	1.00	*15.2*	*20.0*	sand
					**Mean:**	**10.8**	**2.0**	
					**St. Dev.**	**4.2**	**1.0**	

Case number: 107
Earthquake: 1983 Nihonkai-Chubu
Magnitude: 7.7 (M)
Location: Noshiro N-7
References: Hamada (1992)

Nature of Failure: Lateral spreading

Comments: The Nihonkai-chubu Earthquake, with a magnitude of 7.7 occurred on May 26, 1983 in the northwest Japan and produced liquefaction sites in the coastal plain of Akita and Aomori Prefectures. About one month later, a magnitude 7.1 earthquake again hit the area
The site is gently sloped (1-3%). A particulary large displacement occurred on the slopes of Maeyama, a sand hill about 10 m higher than the surrounding area. On the northern slope of the hill, displacements began near the top and extended horizontally for more than 300 m in a northerly direction
The maximum surface acceleration at Akita City 115 km from the epicenter was 0.235 and 0.219 g. The PGA at the site, 100 km away from the epicenter was estimated as 0.25 g.

Summary of Data

	Cetin et al. (2016)	Idriss&Boulanger (2010)	Seed et.al. (1984)		Cetin et al. (2016)	Idriss&Boulanger (2010)	Seed et.al. (1984)
Liquefied?	Yes	yes		D_{50}	0.250 ± 0.050		
Data Class	B			% Fines	1.0 ± 2.0	1.0	
Critical Depth Range	6.6 - 16.4	11.5		% PI			
Depth to GWT (ft)	5.7 ± 0.3	5.6					
σ_v (psf)	1320.5 ± 198.4	1148.7		N	9.8 ± 2.1	9.8	
σ_v' (psf)	962.3 ± 98.8	793.6		C_R	0.87	0.85	
a_{max} (g)	0.250 ± 0.075	0.25		C_S	1.00	1.00	
r_d	0.95 ± 0.052	0.98		C_B	1.00	1.00	
CSR	0.212 ± 0.066	0.232		C_E	1.22	1.22	
Equivalent Magnitude	7.7	7.7		C_N	1.48	1.60	
MSF		0.95		$(N_1)_{60}$	15.3 ± 3.3	16.2	
CSRN		0.222					

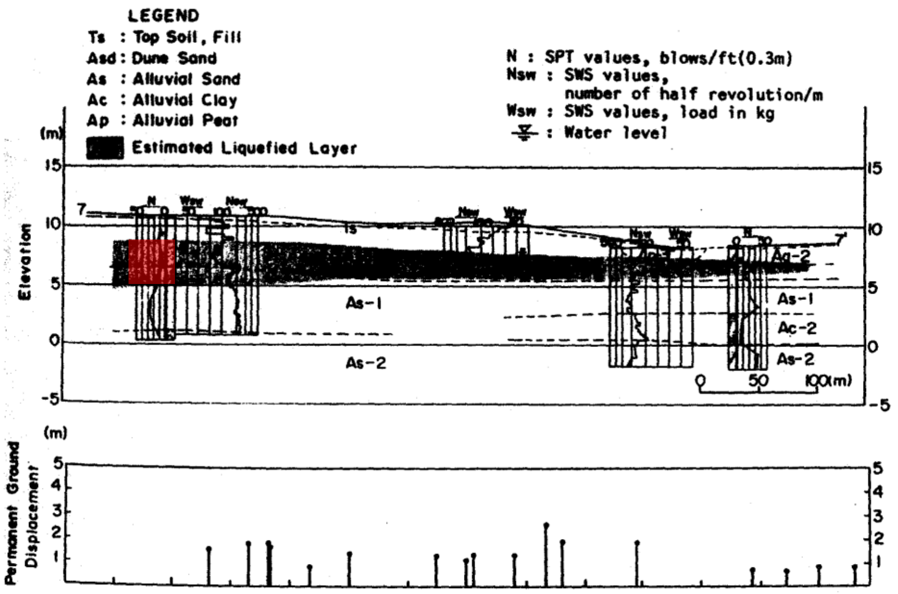

γ_m=110±3 pcf

γ_s=120±3 pcf

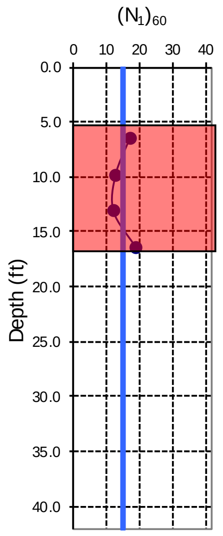

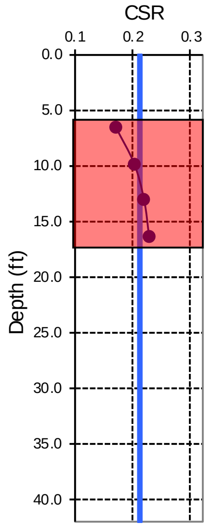

Depth (m)	Depth (ft)	CSR	N	C_N	C_R	$(N1)_{60}$
2.0	6.6	0.171	10.0	1.77	0.80	***17.1***
3.0	9.8	0.202	8.0	1.56	0.85	***12.9***
4.0	13.1	0.219	8.0	1.42	0.89	***12.2***
5.0	16.4	0.228	13.0	1.30	0.92	***19.0***
					**Mean:**	15.3
					**St. Dev.**	3.3

**Case number:**	108
**Earthquake:**	1983 Nihonkai-Chubu
**Magnitude:**	7.7 (Mw by USGS, NISEE)
**Location:**	Ohama No. 2 (2)
**References:**	Iai. et. al. (1989)
**Nature of Failure:**	Liquefied and nonliquefied areas are defined by the boundary lines between artifically filled areas and the areas of the Quaternary sediments which are shown in photos provided by Iai et. al. (1989)

Comments:

Tsuchida et. al (1985) shows the liquefied and non liquefied sites on Akita Port in Figure 8.
Ohama No. 2 (2) is 2.53 km away from strong ground motion station.
SMAC_B2 type accelerograph measured an acceleration value of 190 and 205 Gals in NS and EW directions. "These two values are used for computing the rock outcrop motion. Then this rock outcrop motion is used as input for the earthquake response analysis at several sites." (Kurata et. Al. 1983)
In Figure 8 three digit numbers shows the maximum acceleration values obtained from ground response analysis. However this procedure is done for one component which is 205 Gals (EW) In order ro take into account multi-directional effects site response by Iai et.al. was scaled, maximum acceleration values of 205 and 195 Gals are obtained.
Geometric mean of 205 and 195 Gals is equal to 0.204 g is assigned to site.
SPT procedures were not clearly documented. However, on page 66 in Iai et. al. for comparison with Seed et. al. 1985, an average Japanese SPT energy ratio of 73% was reported to be used. On the basis of this, with some uncertainty Iai et. al. N values may be adopted as N_{73} values. Note that Seed et. al. (1985) adopted an energy ratio of 73% for liquefied cases.
The rationale behind this choice could be field data which is unknown to us or simply
From Technical Report of the Port and Horbour Research Institute Ministry of Transport, Japan No. 511 Marc. 1985 the borehole data is obtained.
Site response by Iai et.al. exists.
There is an ambiguity if the site is free field or not in the vicinty of Akita Port Channel which is 10 m in depth.
From Figure 8 the site is 38 m away from channel.
Note that the location of the site may not be correctly located in Figure 8 since the scale is very small and the coordinates of the borehole are not available.
Additionally Quay walls are believed to be located 38 m away from site.
Presence of Quay walls and due to proximity to the Akita Port Channel which 10 m free field stress boundary conditions may not be fulfilled. Hence cases within 20 m to the Quay walls and channel are eliminated.

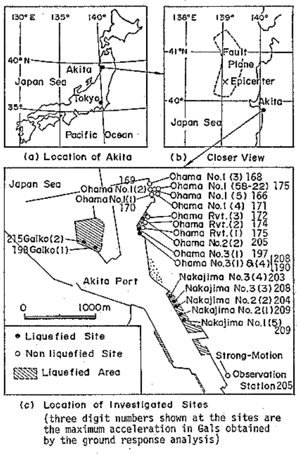

Fig. 8. Investigated sites in Akita Port

Summary of Data

	Cetin et al. (2016)	Idriss&Boulanger (2010)	Seed et.al. (1984)		Cetin et al. (2016)	Idriss&Boulanger (2010)	Seed et.al. (1984)
Liquefied?	Yes	Yes		D_{50}	- ± -	-	
Data Class	A	-		% Fines	1.1 ± 0.5	2.0	
Critical Depth Range	7.2 - 37.1	17.1		% PI	-	-	
Depth to GWT (ft)	2.4 ± 0.3	2.4					
σ_v (psf)	2412.4 ± 550.6	2088.5		N	4.6 ± 2.9	3.3	
σ_v' (psf)	1177.9 ± 244.8	1169.6		C_R	0.97	0.95	
a_{max} (g)	0.204 ± 0.031	0.205		C_S	1.00	1.00	
r_d	0.728 ± 0.090	0.960		C_B	1.00	1.00	
CSR	0.198 ± 0.039	0.229		C_E	1.22	1.22	
Equivalent Magnitude	7.7	7.7		C_N	1.34	1.43	
MSF		0.95		$(N_1)_{60}$	7.3 ± 4.6	5.4	
CSRN		0.23					

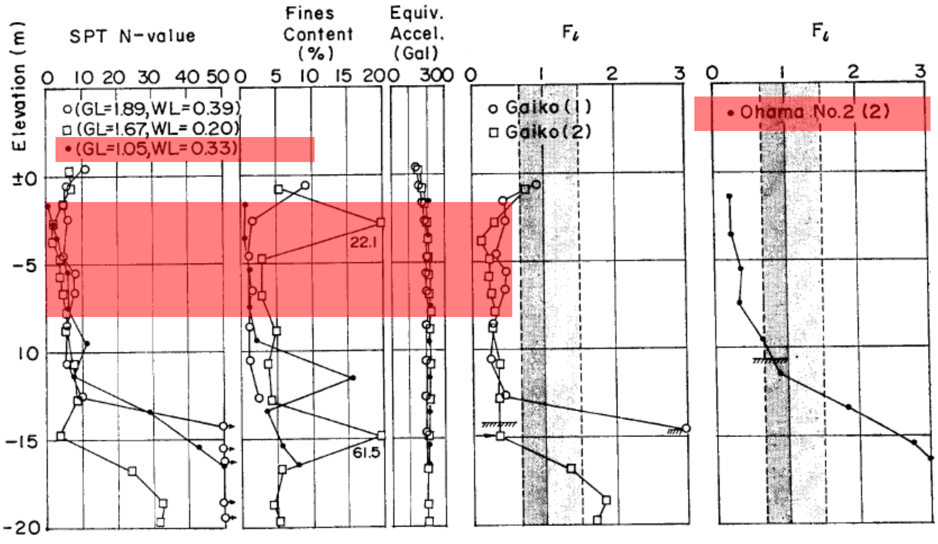

(a) **Gaiko and Ohama No. 2 (Liquefied)**

γ_m=100±3 pcf

γ_s=110±3 pcf

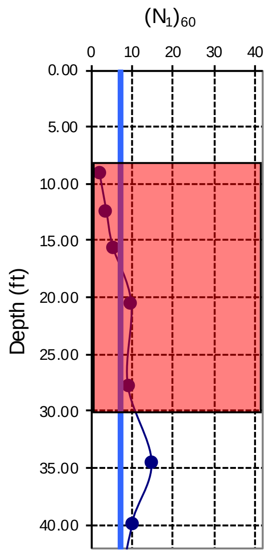

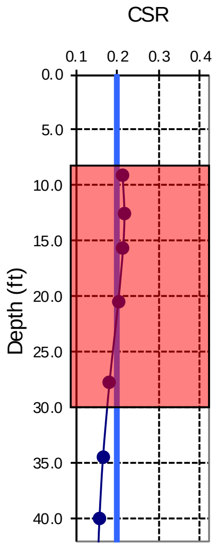

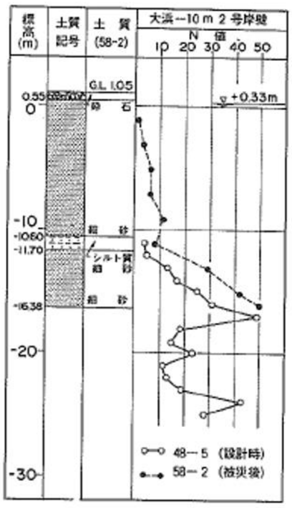

図−3.1.115 大浜−10m 2号岸壁 (N値) 土質 (58−2)

Depth (m)	Depth (ft)	CSR	N	C_N	C_R	$(N1)_{60}$	FC	
2.77	9.09	0.213	1	1.95	0.84	**2.0**	**1.0**	sand
3.80	12.47	0.217	2	1.72	0.88	**3.7**	**-**	sand
4.75	15.58	0.214	3	1.56	0.91	**5.2**	**0.5**	sand
6.25	20.51	0.202	6	1.39	0.96	**9.7**	**1.0**	sand
8.45	27.72	0.182	6	1.21	1.00	**8.8**	**1.0**	sand
10.53	34.55	0.167	11	1.09	1.00	**14.6**	**2.0**	sand
12.17	39.93	0.159	8	1.02	1.00	10.0	16.0	silt
13.55	44.46	0.154	9	0.97	1.00	10.6	4.0	sand
13.95	45.77	0.153	29	0.96	1.00	33.8	6.0	sand
16.55	54.30	0.150	43	0.88	1.00	46.2	8.0	sand
					**Mean:**	**7.3**	**1.1**	
					**St. Dev.**	**4.6**	**0.5**	

Case number: 109

Earthquake: 1983 Nihonkai-Chubu

Magnitude: 7.7 (Mw by USGS, NISEE)

Location: Ohama No. Rvt. (1)

References: Iai. et. al. (1989)

Nature of Failure: Liquefied and nonliquefied areas are defined by the boundary lines between artifically filled areas and the areas of the Quaternary sediments which are shown in photos provided by Iai et. al. (1989)

Comments: Tsuchida et. al (1985) shows the liquefied and non liquefied sites on Akita Port in Figure 8.
Ohama No. Rvt. (1) is 2.43 km away from strong ground motion station.
SMAC_B2 type accelerograph measured an acceleration value of 190 and 205 Gals
in NS and EW directions. "These two values are used for computing the rock outcrop motion.
Then this rock outcrop motion is used as input for the earthquake
response analysis at several sites." (Kurata et. Al. 1983)
In Figure 8 three digit numbers shows the maximum acceleration
values obtained from ground response analysis. However this
procedure is done for one component which is 175 Gals (EW) In order ro take into account multi-directional
effects site response by Iai et.al. was scaled, maximum acceleration values of 175 and 166 Gals are obtained.
Geometric mean of 175 and 166 Gals is equal to 0.174 g is assigned to site.
SPT procedures were not clearly documented. However, on page 66
in Iai et. al. for comparison with Seed et. al. 1985,
an average Japanese SPT energy ratio of 73% was reported
to be used. On the basis of this, with some uncertainty Iai et. al.
N values may be adopted as N_{73} values. Note that Seed et. al. (1985)
adopted an energy ratio of 73% for liquefied cases.
The rationale behind this choice could
be field data which is unknown to us or simply
a conservative engineering judgement. Soil profile data is
missing just N values and FC exist.
From Technical Report of the Port and Horbour Research Institute Ministry of Transport,
Japan No. 511 Marc. 1985 the borehole data could not be obtained.
Site response by Iai et.al. exists however soil profile not known.
There is an ambiguity if the site is free field or not in the vicinty
of Akita Port Channel which is 10 m in depth.
From Figure 8 the site is 76 m away from channel.
Note that the location of the site may not be correctly located in Figure 8
since the scale is very small and the coordinates of the borehole are not available.
Additionally Quay walls are believed to be located 76 m away from site.
Presence of Quay walls and due to proximity to the Akita Port Channel which 10 m free
field stress boundary conditions may not be fulfilled. Hence cases within 20 m to the
Quay walls and channel are eliminated.

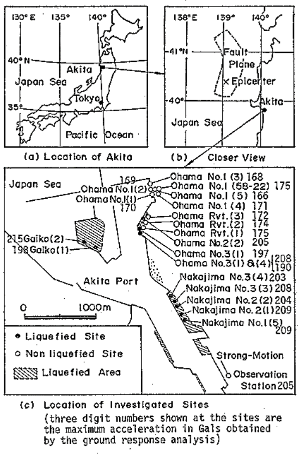

Fig. 8. Investigated sites in Akita Port

Summary of Data

	Cetin et al. (2016)	Idriss&Boulanger (2010)	Seed et.al. (1984)		Cetin et al. (2016)	Idriss&Boulanger (2010)	Seed et.al. (1984)
Liquefied?	No	No		D_{50}	- ± -	-	
Data Class	B	-		% Fines	3.0 ± 1.0	2.0	
Critical Depth Range	11.6 - 24.8	14.9		% PI		-	
Depth to GWT (ft)	4.8 ± 3.0	4.8					
σ_v (psf)	2251.1 ± 278.5	1796.1		N	17.2 ± 2.1	15.8	
σ_v' (psf)	1412.3 ± 224.5	1148.7		C_R	0.94	0.95	
a_{max} (g)	0.174 ± 0.026	0.205		C_S	1.00	1.00	
r_d	0.973 ± 0.076	0.970		C_B	1.00	1.00	
CSR	0.175 ± 0.038	0.200		C_E	1.22	1.22	
Equivalent Magnitude	7.7	7.7		C_N	1.22	1.28	
MSF		0.95		$(N_1)_{60}$	24.0 ± 2.9	23.5	
CSRN		0.19					

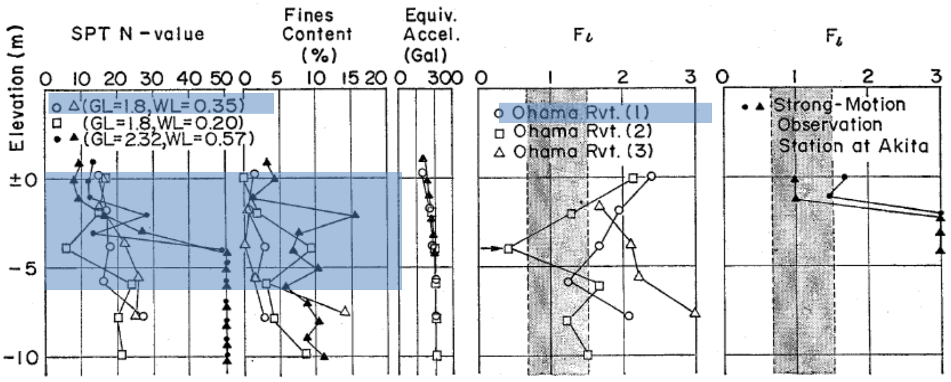

(e) **Ohama No. 2 Former Revetment and the strong-motion observation station (Nonliquefied)**

Fig. 9. Ground conditions and resulted liquefaction potentials at Akita Port during 1983 Nihonkai-Chubu Earthquake

γ_m=120±3 pcf

γ_s=125±3 pcf

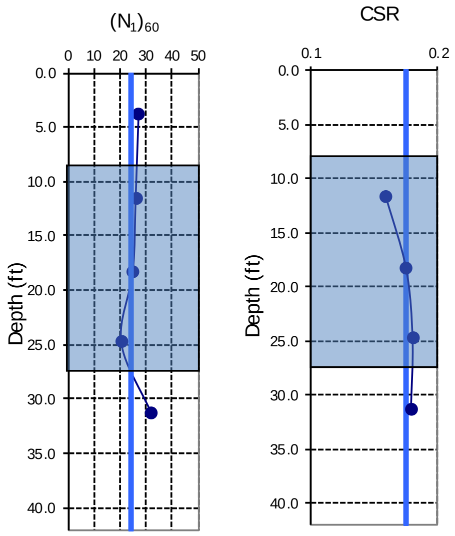

Depth (m)	Depth (ft)	CSR	N	C_N	C_R	$(N1)_{60}$	FC
1.18	3.9	NA	15	2.00	0.75	27.3	3.0
3.54	11.6	0.160	17	1.46	0.87	***26.2***	***2.0***
5.58	18.3	0.176	18	1.22	0.94	***25.1***	***4.0***
7.56	24.8	0.181	16	1.08	0.99	***20.8***	***3.0***
9.53	31.3	0.179	27	0.97	1.00	32.0	4.0
					**Mean:**	**24.0**	**3.0**
					**St. Dev.**	**2.9**	**1.0**

**Case number:**	110
**Earthquake:**	1983 Nihonkai-Chubu
**Magnitude:**	7.7 (M)
**Location:**	Nakazata Town (Takeda Elementary School)
**References:**	Yasuda and Tohno (1988)
**Nature of Failure:**	Sand boils, settled structures.
**Comments:**	The Nihonkai-chubu Earthquake, with a magnitude of 7.7 occurred on May 26, 1983 in the northwest Japan and produced liquefaction sites in the coastal plain of Akita and Aomori Prefectures. About one month later, a magnitude 7.1 earthquake again hit the area and caused liquefaction at the Takeda Elementary School in Nakazato Town. The school was built on the old bed of the Iwaki River. During the main shock on May 26, the ground beneath the schoolhouses (two-story wood structures) and spewed out sand and water then was submerged. The aftershock of June 21 again caused sand and water to swep out from the ground. According to some teachers, the area where sand volcanoes were observed almost coincided with the area where sand volcaones had been caused by the ,main shock.<br>The maximum surface acceleration was assumed to be 0.278 and 0.109 g for the main and after shock respectively based on the values recorded at the Tsugaru-ohashi Bridge about 6 km from the school.

Summary of Data

	Cetin et al. (2016)	Idriss&Boulanger (2010)	Seed et.al. (1984)		Cetin et al. (2016)	Idriss&Boulanger (2010)	Seed et.al. (1984)
Liquefied?	Yes	Yes		D_{50}	0.240 ± 0.050		
Data Class	B			% Fines	0.0 ± 2.0	0.0	
Critical Depth Range	8.2 - 21.3	14.1		% PI			
Depth to GWT (ft)	1.1 ± 0.4	1.3					
s_v (psf)	1680.6 ± 254.9	1691.7		N	7.9 ± 0.9	7.4	
s_v' (psf)	831.0 ± 123.3	877.2		C_R	0.90	0.95	
a_{max} (g)	0.278 ± 0.083	0.28		C_S	1.00	1.00	
r_d	0.85 ± 0.064	0.97		C_B	1.00	1.00	
CSR	0.312 ± 0.097	0.397		C_E	1.22	1.22	
Equivalent Magnitude	7.7	7.7		C_N	1.60	1.56	
MSF		0.95		$(N_1)_{60}$	13.8 ± 1.5	13.3	
CSRN		0.381					

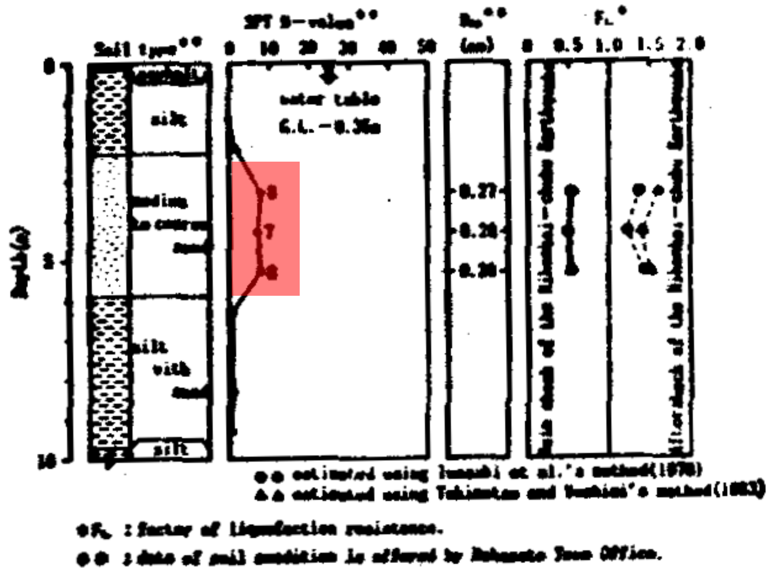

γ_m=100±3 pcf

γ_s=115±3 pcf

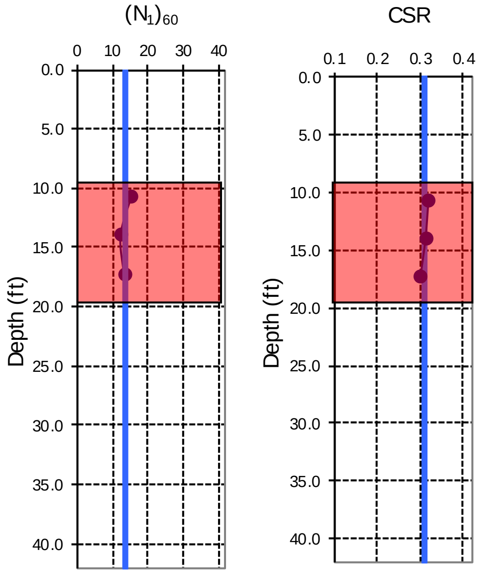

Depth (m)	Depth (ft)	CSR	N	C_N	C_R	$(N1)_{60}$
3.3	10.7	0.322	8.0	1.85	0.86	***15.5***
4.3	13.9	0.315	7.0	1.64	0.90	***12.5***
5.3	17.2	0.303	8.0	1.48	0.93	***13.4***
					**Mean:**	13.8
					**St. Dev.**	1.5

**Case number**	111
**Earthquake:**	1987 Elmore Ranch
**Magnitude:**	6.2 (Mw)
**Location:**	Radio Tower B1
**References:**	James Gerard Bierschwale (1984), Andrus and Stokoe (1997)<br>Bennett et al. (1984) and Fear et al. (1995)
**Nature of Failure:**	No surface evidence of liquefaction
**Comments:**	The Imperial Valley lies in the southern part of the Salton Basin, which was formed by northward extension of the crustal rifting that opened the Gulf of California. The basin is underlain by as much as 20,000 ft of Tertiary and Quaternary marine, continental, and lacustrine sediment. Holocene sediment is as thick as 330 ft.<br>The principal rivers are the Alamo and New Rivers along which many sand boils were developed on fluvial deposits.<br>A: very poorly sorted, soft brown clay and silt-flood plain deposit<br>B: brown, loose to med.dense silt to fine sand- sediment at base is well sorted very fine sand fining upwards to poorly sorted silt-channel deposit<br>C: very stiff brown clayey silt to clay-lacustrine<br>D: med dense sand<br>PGA was recorded at the surface and at 6.7 m depth of Wildlife site as 0.126 and 0.077g respectively. The epicentral distance is ~25 km sligthly further than Wildlife site.The earthquake is very poorly monitored. Only one seismogram is available. Andrus and Stoke (NCEER, 97) predicted the PGA at the site as 0.11g. A PGA of 0.09 g was adopted for these studies.<br>Energy ratio ~68 %

Summary of Data

	Cetin et al. (2016)	Idriss&Boulanger (2010)	Seed et.al. (1984)		Cetin et al. (2016)	Idriss&Boulanger (2010)	Seed et.al. (1984)
Liquefied?	No	No		D_{50}	0.074 ± 0.037		
Data Class	B			% Fines	43.5 ± 29.0	64.0	
Critical Depth Range	9.8 - 18.0	11.2		% PI			
Depth to GWT (ft)	6.6	6.9					
σ_v (psf)	1505.1 ± 160.1	1294.9		N	4.5 ± 3.4	2.0	
σ_v' (psf)	1044.5 ± 79.5	1044.3		C_R	0.86	0.85	
a_{max} (g)	0.090 ± 0.014	0.09		C_S	1.00	1.00	
r_d	0.97 ± 0.032	0.96		C_B	1.00	1.00	
CSR	0.082 ± 0.013	0.069		C_E	1.13	1.13	
Equivalent Magnitude	6.2	6.2		C_N	1.42	1.49	
MSF		1.40		$(N_1)_{60}$	6.2 ± 4.7	2.9	
CSRN		0.046					

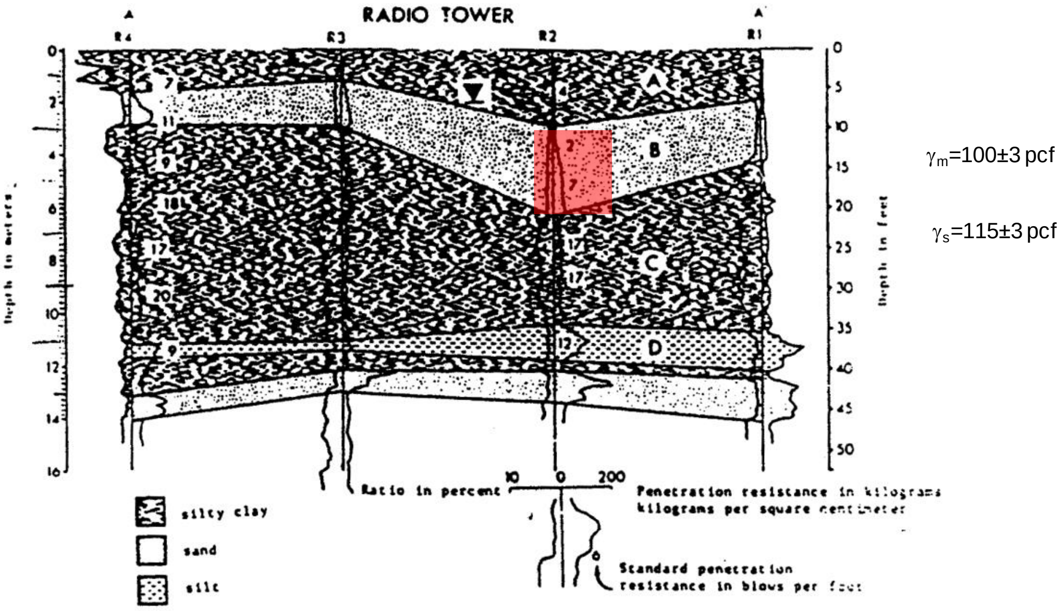

γ_m=100±3 pcf

γ_s=115±3 pcf

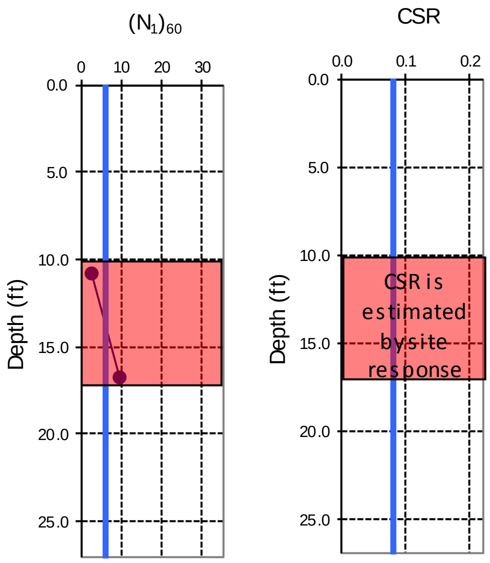

Depth (m)	Depth (ft)	CSR	N	C_N	C_R	$(N1)_{60}$
3.3	10.8		2	1.55	0.82	***2.9***
5.1	16.7		7	1.33	0.89	***9.5***
					**Mean:**	6.2
					**St. Dev.**	4.7

Depth (m)	FC	sand	silt	clay	D50
3.3	***64.0***	***36.0***	***59.0***	***5.0***	***0.047***
5.1	***23.0***	***77.0***	***22.0***	***1.0***	***0.100***
23.15	*18.0*	*82.0*	*13.0*	*5.0*	*0.125*
	43.5	56.5	40.5	3.0	0.074
	29.0	29.0	26.2	2.8	0.037

**Case number:**	112
**Earthquake:**	1987 Elmore Ranch
**Magnitude:**	6.2 (Mw)
**Location:**	Wildlife B
**References:**	Bennett et al. (1984) and Fear et al. (1995)<br>James Gerard Bierschwale. (1984)<br>Youd, T.L., Wieczorek G. F., (1984), "Liquefaction during the 1981 and Previous Earthquakes Near Westmorland, CA" Open File Rep. 84-680
**Nature of Failure:**	Site has not liquefied. Neither pore pressure ratios exceeding 1 nor surface evidence of liquefaction.
**Comments:**	The Imperial Valley lies in the southern part of the Salton Basin, which was formed by northward extension of the crustal rifting that opened the Gulf of California. The basin is underlain by as much as 20,000 ft of Tertiary and Quaternary marine, continental, and lacustrine sediment. Holocene sediment is as thick as 330 ft.<br>The site is very closely monitored. Strong ground motion seismometers are available at the surface and at 6.7 m depth. Piezometers were installed almost at every meter depth along the potentially liquefiable layer of thickness 4 m.<br>A: very loose and very soft interbedded micaceous sandy silt, silt and clayey silts (0-2.5 m) flood plain deposits<br>B: very loose to loose moderately sorted sandy silt (2.5-6.8m); point bar deposits<br>C: silty clay (medium to very stiff) and clayey silt (medium to stiff); lacustrine deposit.<br>PGA was recorded at the surface and at 6.7 m depth as 0.126 and 0.077g. respectively. The epicentral distance is 22.9 km.<br>Energy ratio ~68 %

**Summary of Data**	Cetin et al. (2016)	Idriss&Boulanger (2010)	Seed et.al. (1984)		Cetin et al. (2016)	Idriss&Boulanger (2010)	Seed et.al. (1984)
Liquefied?	No	No		D_{50}	0.109 ± 0.038		
Data Class	A			% Fines	26.2 ± 7.6	30.0	
Critical Depth Range	9.0 - 22.0	15.1		% PI			
Depth to GWT (ft)	3.0	3.9					
σ_v (psf)	1800.0 ± 262.9	1817.0		N	7.9 ± 3.5	7.1	
σ_v' (psf)	1020.0 ± 131.5	1127.8		C_R	0.88	0.95	
a_{max} (g)	0.130 ± 0.020	0.13		C_S	1.00	1.00	
r_d	0.75 ± 0.035	0.93		C_B	1.00	1.00	
CSR	0.112 ± 0.018	0.138		C_E	1.13	1.13	
Equivalent Magnitude	6.2	6.2		C_N	1.44	1.36	
MSF		1.40		$(N_1)_{60}$	11.3 ± 5.0	10.3	
CSRN		0.092					

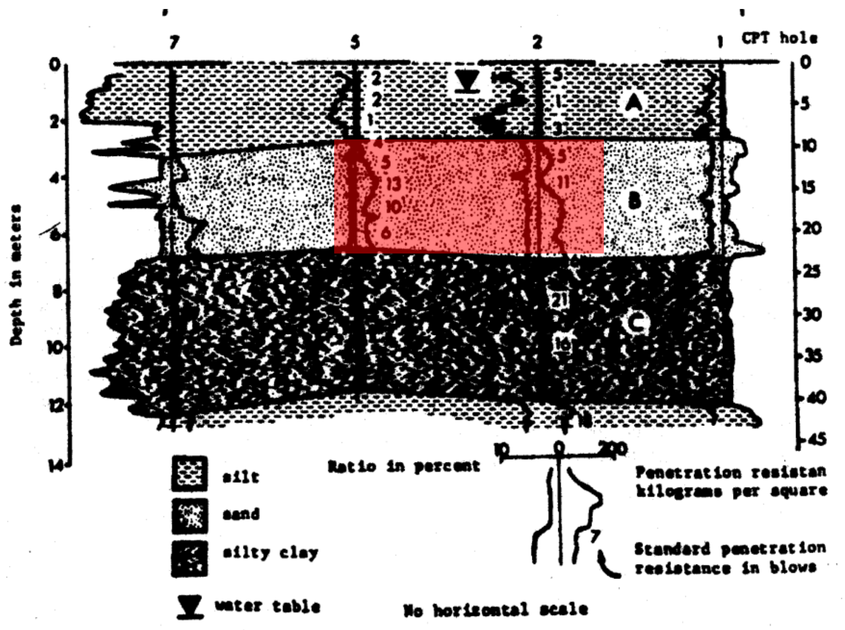

γ_m=100±3 pcf

γ_s=120±3 pcf

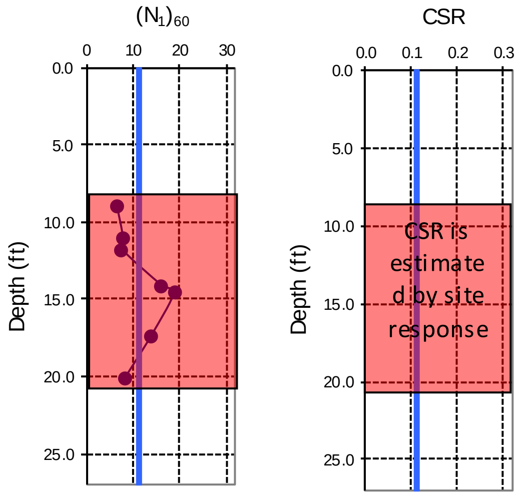

Depth (m)	Depth (ft)	CSR	N	C_N	C_R	$(N1)_{60}$	
2.75	9.0		4	1.81	0.79	***6.5***	Hole-5
3.35	11.0		5	1.67	0.82	***7.8***	Hole-2
3.6	11.8		5	1.62	0.83	***7.6***	Hole-5
4.3	14.1		11	1.50	0.86	***16.2***	Hole-2
4.45	14.6		13	1.48	0.87	***18.9***	Hole-5
5.3	17.4		10	1.37	0.90	***14.0***	Hole-5
6.15	20.2		6	1.28	0.93	***8.1***	Hole-5
					**Mean:**	11.3	
					**St. Dev.**	5.0	

Depth (ft)	FC	Sand	Silt	Clay	D50	
10.5	***34.0***	***66.0***	***34.0***	***0.0***	***0.176***	Hole-2
11.2	***17.0***	***83.0***	***17.0***	***0.0***	***0.096***	Hole-2
13.5	***27.0***	***73.0***	***26.0***	***1.0***	***0.090***	Hole-2
14.2	***20.0***	***80.0***	***20.0***	***0.0***	***0.100***	Hole-2
18.3	***33.0***	***67.0***	***29.0***	***4.0***	***0.082***	Hole-2
22.8	*18.0*	*82.0*	*17.0*	*1.0*	*0.102*	Hole-2
**Mean:**	26.2	73.8	25.2	1.0	0.109	
**St. Dev.**	7.6	7.6	6.8	1.7	0.038	

**Case number:**	113
**Earthquake:**	Superstition Hills
**Magnitude:**	6.54 (Mw)
**Location:**	Heber Road A1
**References:**	Youd et al. (1983), "Liquefaction sites, Imperial Valley, CA<br>Andrus and Stokoe, 1997
**Nature of Failure:**	No surface manifestation of liquefaction
**Comments:**	The Imperial Valley lies in the southern part of the Salton Basin, which was formed by northward extension of the crustal rifting that opened the Gulf of California. The basin is underlain by as much as 20,000 ft of Tertiary and Quaternary marine, continental, and lacustrine sediment. Holocene sediment is as thick as 330 ft.<br>The principal rivers are the Alamo and New Rivers along which many sand boils were developed on fluvial deposits.<br>Heber Road site is ~60 km to the epicenter. Due to the lack of significant surface trace of the fault rupture, it is difficult to estimate the closest distance to the fault rupture. A soil PGA of 0.36g was recorded at El Centro array site # 5, which is ~15 km north of Heber Road site. Based on attenuation relationships, site response analyses and available recordings, a PGA in the range of 0.12-0.16 was estimated. Based on site response analyses results, a PGA value of 0.16 g was adopted.<br>ASTM D1586-67 procedures were followed for SPT tests. 10" outside diameter hollow-stem auger was used with Safe-T- Driver and safety hammer for the drillings in 1979 and 1981. Donut Hammer with rope cathead was used for the drillings in in 1982. Energy ratios are adopted as 68 and 66 % for safety and donut hammers respectively.

Summary of Data

	Cetin et al. (2016)	Idriss&Boulanger (2010)	Seed et.al. (1984)		Cetin et al. (2016)	Idriss&Boulanger (2010)	Seed et.al. (1984)
Liquefied?	No	No		D_{50}	0.111 ± 0.007		
Data Class	A			% Fines	13.0 ± 3.9	12.0	
Critical Depth Range	5.9 - 16.4	11.2		% PI			
Depth to GWT (ft)	5.9	6.9					
σ_v (psf)	1246.7 ± 220.1	1294.9		N	32.9 ± 2.6	30.4	
σ_v' (psf)	919.2 ± 112.6	1044.3		C_R	0.82	0.85	
a_{max} (g)	0.156 ± 0.023	0.20		C_S	1.00	1.00	
r_d	0.96 ± 0.022	0.96		C_B	1.00	1.00	
CSR	0.132 ± 0.022	0.154		C_E	1.13	1.13	
Equivalent Magnitude	6.54	6.54		C_N	1.52	1.30	
MSF		1.29		$(N_1)_{60}$	46.5 ± 3.7	37.8	
CSRN		0.113					

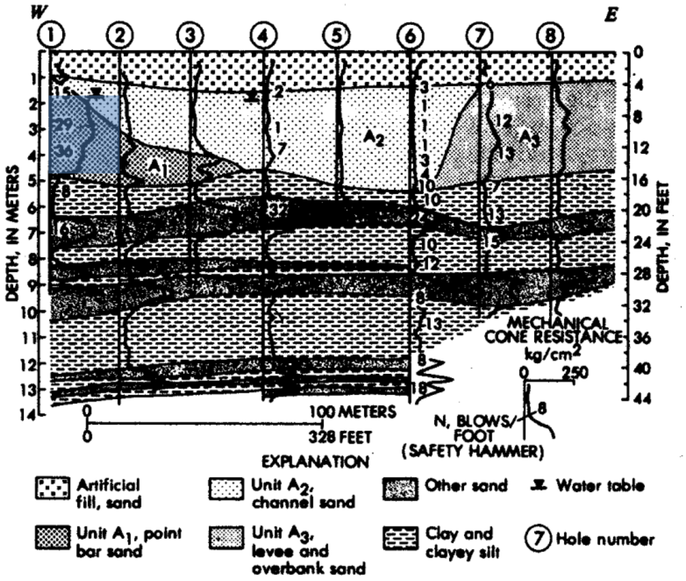

γ_m=100±3 pcf

γ_s=125±3 pcf

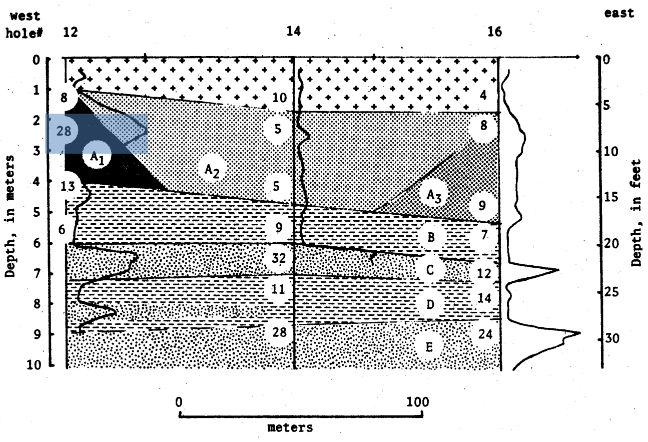

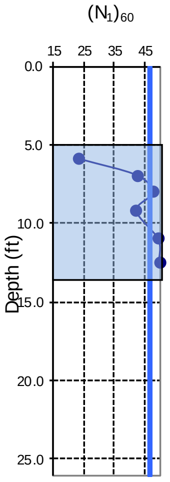

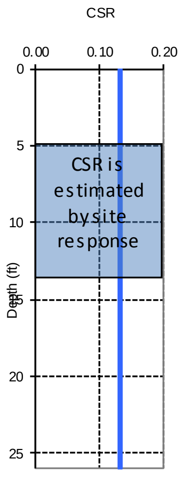

Depth (m)	Depth (ft)	CSR	N	C_N	C_R	$(N1)_{60}$	
1.8	5.9		15	1.89	0.73	23.4	BH-1
2.12	7.0		28	1.80	0.75	***42.7***	BH-12
2.45	8.0		32	1.71	0.77	***47.8***	BH-1 Donut
2.8	9.2		29	1.63	0.79	***42.4***	BH-1 Donut
3.35	11.0		35	1.53	0.82	***49.7***	BH-1
3.8	12.5		36	1.45	0.84	***49.9***	BH-1
					**Mean:**	46.5	
					**St. Dev.**	3.7	

**Case number:**	114
**Earthquake:**	Superstition Hills
**Magnitude:**	6.54 (Mw)
**Location:**	Heber Road A2
**References:**	Youd et al. (1982), "Liquefaction sites, Imperial Valley, CA<br>Andrus and Stokoe, 1997
**Nature of Failure:**	No surface manifestation of liquefaction
**Comments:**	The Imperial Valley lies in the southern part of the Salton Basin, which was formed by northward extension of the crustal rifting that opened the Gulf of California. The basin is underlain by as much as 20,000 ft of Tertiary and Quaternary marine, continental, and lacustrine sediment. Holocene sediment is as thick as 330 ft.<br>The principal rivers are the Alamo and New Rivers along which many sand boils were developed on fluvial deposits.<br>Heber Road site ~60 km to the epicenter. Due to the lack of significant surface trace of the fault rupture, it is difficult to estimate the closest distance to the fault rupture. A soil PGA of 0.36g was recorded at El Centro array site # 5, which is ~15 kms north of Heber Road site. Based on attenuation relationships, site response analyses and available recordings, a PGA in the range of 0.12-0.16 was estimated. Site response analysis results suggests a PGA value of 0.15 g.<br>ASTM D1586-67 procedures were followed for SPT tests. 10" outside diameter hollow-stem auger was used with Safe-T- Driver and safety hammer for the drillings in 1979 and 1981. Donut Hammer with rope cathead was used for the drillings in in 1982. Energy ratios are adopted as 68 and 66 % for safety and donut hammers respectively.

**Summary of Data**	Cetin et al. (2016)	Idriss&Boulanger (2010)	Seed et.al. (1984)		Cetin et al. (2016)	Idriss&Boulanger (2010)	Seed et.al. (1984)
Liquefied?	No	No		D_{50}	0.116 ± 0.013		
Data Class	A			% Fines	20.9 ± 5.0	18.0	
Critical Depth Range	6.0 - 15.1	12.1		% PI			
Depth to GWT (ft)	5.9	5.9					
σ_v (psf)	1103.0 ± 167.6	1420.2		N	2.4 ± 1.5	2.0	
σ_v' (psf)	812.3 ± 76.9	1044.3		C_R	0.81	0.85	
a_{max} (g)	0.150 ± 0.023	0.15		C_S	1.00	1.00	
r_d	0.95 ± 0.024	0.96		C_B	1.00	1.00	
CSR	0.126 ± 0.021	0.127		C_E	1.13	1.13	
Equivalent Magnitude	6.54	6.54		C_N	1.61	1.51	
MSF		1.29		$(N_1)_{60}$	3.5 ± 2.2	2.9	
CSRN		0.093					

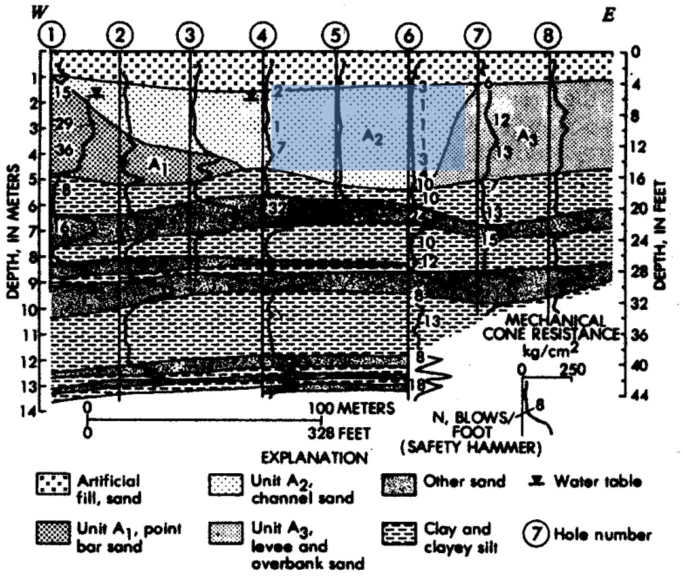

γ_m=100±3 pcf

γ_s=110±3 pcf

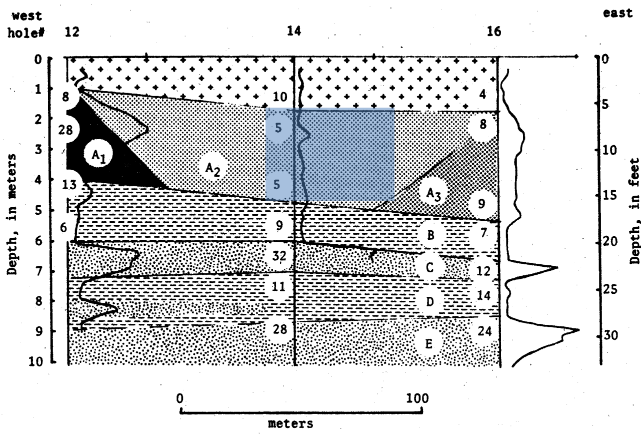

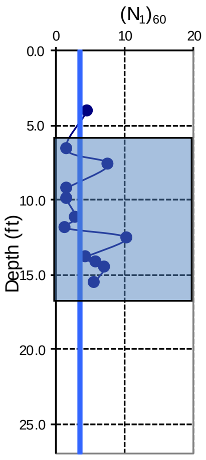

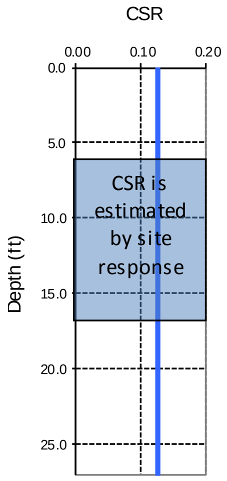

Depth (m)	Depth (ft)	CSR	N	C_N	C_R	$(N1)_{60}$	
1.2	3.9		3	2.00	0.68	4.6	BH-6
2	6.6		1	1.84	0.74	***1.6***	BH-6
2.3	7.5		5	1.78	0.76	7.7	BH-14
2.8	9.2		1	1.68	0.79	***1.5***	BH-6
3	9.8		1	1.65	0.80	***1.5***	BH-4
3.4	11.2		2	1.59	0.82	***3.0***	BH-5 Donut
3.6	11.8		1	1.56	0.83	***1.5***	BH-6
3.8	12.5		7	1.53	0.84	10.2	BH-4
4.2	13.8		3	1.48	0.86	***4.3***	BH-6
4.3	14.1		4	1.47	0.86	***5.7***	BH-5 Donut
4.4	14.4		5	1.46	0.87	***7.2***	BH-14
4.7	15.4		4	1.42	0.88	***5.7***	BH-6
					**Mean:**	3.5	
					**St. Dev.**	2.2	

**Case number:**	115
**Earthquake:**	Superstition Hills
**Magnitude:**	6.54 (Mw)
**Location:**	Heber Road A3
**References:**	Youd et al. (1982), "Liquefaction sites, Imperial Valley, CA
**Nature of Failure:**	No surface manifestation of liquefaction
**Comments:**	The Imperial Valley lies in the southern part of the Salton Basin, which was formed by northward extension of the crustal rifting that opened the Gulf of California. The basin is underlain by as much as 20,000 ft of Tertiary and Quaternary marine, continental, and lacustrine sediment. Holocene sediment is as thick as 330 ft.<br>The principal rivers are the Alamo and New Rivers along which many sand boils were developed on fluvial deposits.<br>Heber Road site ~60 km to the epicenter. Due to the lack of significant surface trace of the fault rupture, it is difficult to estimate the closest distance to the fault rupture. A soil PGA of 0.36 g was recorded at El Centro array site # 5, which is ~15 kms north of Heber Road site. Based on attenuation relationships, site response analyses and available recordings, a PGA in the range of 0.12-0.16 g was estimated. Site response analysis results suggest a PGA value of 0.13 g.<br>ASTM D1586-67 procedures were followed for SPT tests. 10" outside diameter hollow-stem auger was used with Safe-T- Driver and safety hammer for the drillings in 1979 and 1981. Donut Hammer with rope cathead was used for the drillings in in 1982. Energy ratios are adopted as 68 and 66 % for safety and donut hammers respectively.

Summary of Data

	Cetin et al. (2016)	Idriss&Boulanger (2010)	Seed et.al. (1984)		Cetin et al. (2016)	Idriss&Boulanger (2010)	Seed et.al. (1984)
Liquefied?	No	No		D_{50}	0.094 ± 0.004		
Data Class	A			% Fines	25.3 ± 5.2	25.0	
Critical Depth Range	5.9 - 16.1	13.1		% PI			
Depth to GWT (ft)	5.9	5.9					
σ_v (psf)	1200.8 ± 204.8	1649.9		N	13.0 ± 4.2	13.0	
σ_v' (psf)	883.5 ± 101.2	1169.6		C_R	0.82	0.85	
a_{max} (g)	0.130 ± 0.020	0.13		C_S	1.00	1.00	
r_d	0.95 ± 0.025	0.95		C_B	1.00	1.00	
CSR	0.109 ± 0.018	0.115		C_E	1.13	1.13	
Equivalent Magnitude	6.54	6.54		C_N	1.55	1.29	
MSF		1.29		$(N_1)_{60}$	18.6 ± 6.0	16.2	
CSRN		0.082					

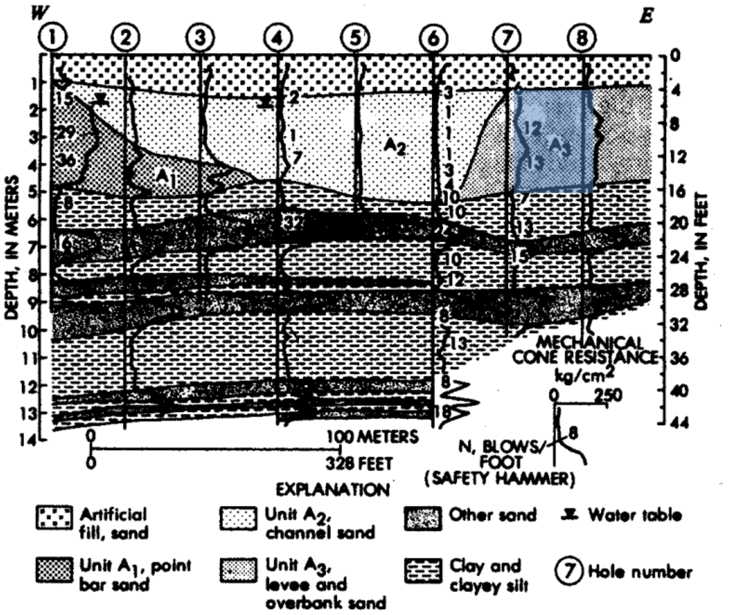

γ_m=100±3 pcf

γ_s=120±3 pcf

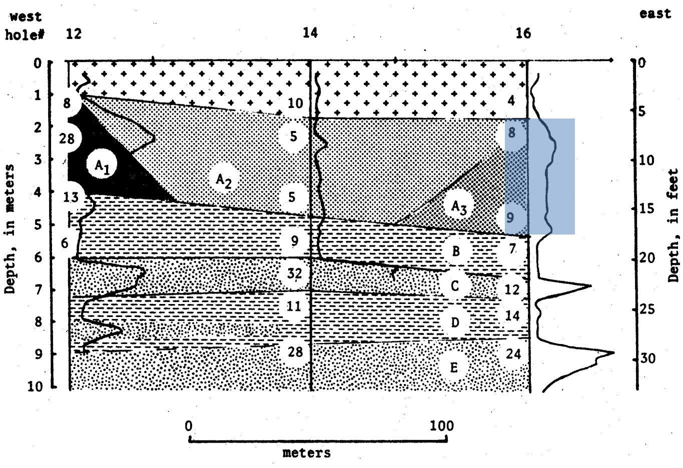

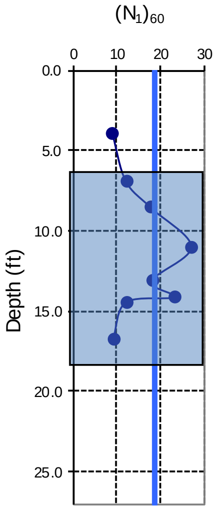

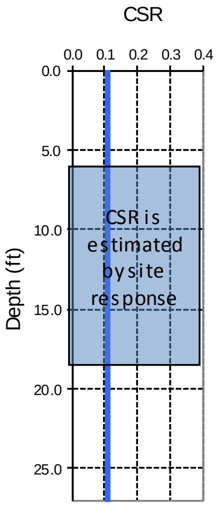

Depth (m)	Depth (ft)	CSR	N	C_N	C_R	$(N1)_{60}$	
1.2	3.9		6	2.00	0.68	9.2	BH-7
2.1	6.9		8	1.81	0.75	***12.3***	BH-16
2.6	8.5		12	1.69	0.78	***17.9***	BH-7
3.35	11.0		19	1.55	0.82	***27.4***	BH-8
4	13.1		13	1.45	0.85	***18.2***	BH-7
4.3	14.1		17	1.41	0.86	***23.5***	BH-8
4.4	14.4		9	1.40	0.87	***12.4***	BH-16
5.1	16.7		7	1.32	0.89	9.4	BH-7
					**Mean:**	18.6	
					**St. Dev.**	6.0	

**Case number:**	116
**Earthquake:**	1987 Superstition Hills
**Magnitude:**	6.54 (Mw)
**Location:**	Kornbloom B
**References:**	Bennett et al. (1984) and Fear et al. (1995)<br>James Gerard Bierschwale. (1984)<br>Youd, T.L., Wieczorek G. F., (1984), "Liquefaction during the 1981 and<br>Previous Earthquakes Near Westmorland, CA" Open File Rep. 84-680
**Nature of Failure:**	No surface evidence of liquefaction
**Comments:**	Imperial Valley lies in the southern part of the Salton Basin, which was<br>formed by northward extension of the crustal rifting that opened the Gulf of<br>California. The basin is underlain by as much as 20,000 ft of Tertiary and<br>Quaternary marine, continental, and lacustrine sediment. Holocene sediment<br>is as thick as 330 ft.<br>The principal rivers are the Alamo and New Rivers along which many sand boils<br>were developed on fluvial deposits.<br>A: silty clay, stiff-lacustrine and/or flood plain deposit<br>B: silty and sandy silt-fluvial and perhaps fluvial deltaic deposition<br>C: silty clay to clay, stiff-lacustrine and/or flood plain deposit<br>D: interbedded silt and sand-loose to medium dense<br>E: Uniform sand with some silt-medium dense to dense<br>F: interbedded silt and sand - loose to medium dense.<br>D-F :fluvial and perhaps fluvial-deltaic deposition<br>PGA was recorded at the Kornbloom road as 0.14 g (temp. seismograph)<br>Site response analysis results suggest a slightly higher PGA of<br>0.17 g<br>Energy ratio ~68 %

Summary of Data

	Cetin et al. (2016)	Idriss&Boulanger (2010)	Seed et.al. (1984)		Cetin et al. (2016)	Idriss&Boulanger (2010)	Seed et.al. (1984)
Liquefied?	No	No		D_{50}	0.045 ± 0.008		
Data Class	A			% Fines	83.0 ± 9.6	92.0	
Critical Depth Range	9.0 - 17.5	14.1		% PI			
Depth to GWT (ft)	9.0	8.9					
σ_v (psf)	1410.0 ± 172.7	1608.2		N	5.4 ± 3.1	5.0	
σ_v' (psf)	1144.8 ± 88.1	1294.9		C_R	0.85	0.85	
a_{max} (g)	0.174 ± 0.026	0.17		C_S	1.00	1.00	
r_d	0.94 ± 0.030	0.95		C_B	1.00	1.00	
CSR	0.131 ± 0.021	0.132		C_E	1.13	1.13	
Equivalent Magnitude	6.54	6.54		C_N	1.36	1.29	
MSF		1.29		$(N_1)_{60}$	7.1 ± 4.1	6.2	
CSRN		0.098					

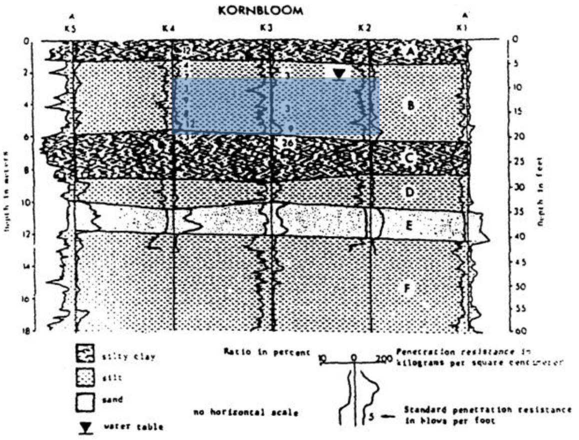

γ_m=100±3 pcf

γ_s=120±3 pcf

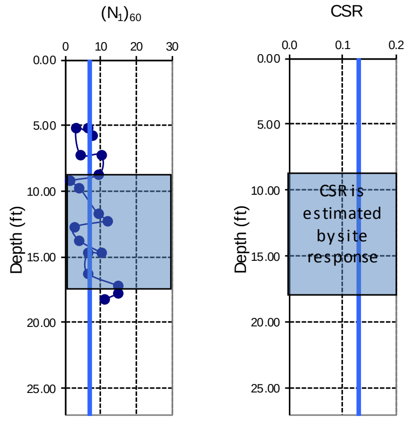

Depth (m)	Depth (ft)	CSR	N	C_N	C_R	$(N1)_{60}$		FC	sand	silt	clay	D50	WN	WL	PI	LI
1.60	5.25		4	2.00	0.71	6.5	K-4	88.0	12.0	65.0	23.0	0.019	26.0	29.0	11.0	0.7
1.75	5.75		5	1.92	0.72	7.9	K-4 b	84.0	16.0	74.0	10.0	0.038				
1.60	5.25		2	2.00	0.71	***3.2***	SK4-5	-	-	-	-	-				
2.21	7.25		3	1.71	0.76	***4.4***	K-3	***80.0***	***21.0***	***73.0***	***7.0***	***0.044***				
2.21	7.25		7	1.71	0.76	***10.2***	K-4	***74.0***	***26.0***	***68.0***	***6.0***	***0.052***				
2.67	8.75		7	1.56	0.78	***9.7***	K-4 b	***86.0***	***14.0***	***83.0***	***3.0***	***0.049***				
2.82	9.25		1	1.52	0.79	***1.4***	SK4-5	-	-	-	-	-				
2.97	9.75		3	1.50	0.80	***4.1***	K-4	***62.0***	***38.0***	***53.0***	***9.0***	***0.056***	***28.0***			
3.58	11.75		7	1.41	0.83	***9.3***	K-4 b	***88.0***	***12.0***	***79.0***	***9.0***	***0.042***				
3.73	12.25		9	1.40	0.84	***11.9***	K-4	***96.0***	***4.0***	***83.0***	***13.0***	***0.028***	***30.0***			
3.89	12.75		2	1.38	0.85	***2.6***	SK4-5	-	-	-	-	-				
4.19	13.75		3	1.34	0.86	***3.9***	K-3	***91.0***	***9.0***	***79.0***	***12.0***	***0.039***				
4.50	14.75		8	1.31	0.87	***10.4***	K-4	***83.0***	***17.0***	***74.0***	***9.0***	***0.047***	***29.0***			
4.50	14.75		5	1.31	0.87	***6.5***	K-4 b	***81.0***	***19.0***	***71.0***	***10.0***	***0.042***				
4.95	16.25		5	1.27	0.89	***6.4***	SK4-5	-	-	-	-	-				
5.26	17.25		12	1.24	0.90	***15.2***	K-4	***89.0***	***11.0***	***83.0***	***6.0***	***0.048***				
5.41	17.75		12	1.23	0.90	15.1	K-4 b	44.0	56.0	40.0	4.0	0.700				
5.56	18.25		9	1.22	0.91	11.3	K-3	30.0	70.0	29.0	1.0	0.082				
					**Mean:**	7.1		83.0	17.1	74.6	8.4	0.045				
					**St. Dev.**	4.1		9.6	9.7	9.3	3.0	0.008				

**Case number:**	117
**Earthquake:**	Superstition Hills
**Magnitude:**	6.7 (Mw)
**Location:**	McKim Ranch A
**References:**	Bennett et al. (1984) and Fear et al. (1995)<br>James Gerard Bierschwale (1984), Andrus and Stokoe, (1997)
**Nature of Failure:**	No surface manifastation of liquefaction
**Comments:**	Imperial Valley lies in the southern part of the Salton Basin, which was formed by northward extension of the crustal rifting that opened the Gulf of California. The basin is underlain by as much as 20,000 ft of Tertiary and Quaternary marine, continental, and lacustrine sediment. Holocene sediment is as thick as 330 ft.<br>The principal rivers are the Alamo and New Rivers along which many sand boils were developed on fluvial deposits.<br>Located between the Alamo River and the Redwood Canal.<br>A: very fine sand (fining upwards)-loose sand that filled the relic channel.<br>B: stiff silty clay<br>Radio Tower Site is ~ 40 km away from the epicenter of the earthquake. The recorded PGA at the El Centro differential array site # 5 (0.36 g) suggest a<br>a high PGA. Contrary to this site response analyses results suggest a<br>a lower PGA (0.16 g)<br>Energy ratio ~68 %

**Summary of Data**	Cetin et al. (2016)	Idriss&Boulanger (2010)	Seed et.al. (1984)		Cetin et al. (2016)	Idriss&Boulanger (2010)	Seed et.al. (1984)
Liquefied?	No	No		D_{50}	0.106 ± 0.005		
Data Class	B			% Fines	19.8 ± 3.5	31.0	
Critical Depth Range	5.0 - 13.0	6.9		% PI			
Depth to GWT (ft)	5.0	4.9					
σ_v (psf)	1030.0 ± 161.2	793.6		N	5.4 ± 2.6	3.0	
σ_v' (psf)	780.4 ± 80.7	668.3		C_R	0.79	0.80	
a_{max} (g)	0.160 ± 0.048	0.16		C_S	1.00	1.00	
r_d	0.95 ± 0.025	0.98		C_B	1.00	1.00	
CSR	0.130 ± 0.040	0.121		C_E	1.13	1.13	
Equivalent Magnitude	6.5	6.5		C_N	1.65	1.70	
MSF		1.29		$(N_1)_{60}$	7.9 ± 3.8	4.6	
CSRN		0.085					

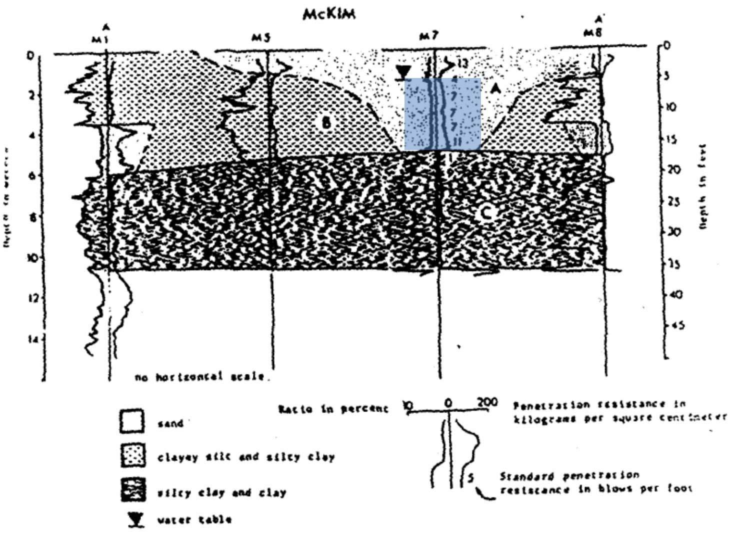

γ_m=110±3 pcf

γ_s=120±3 pcf

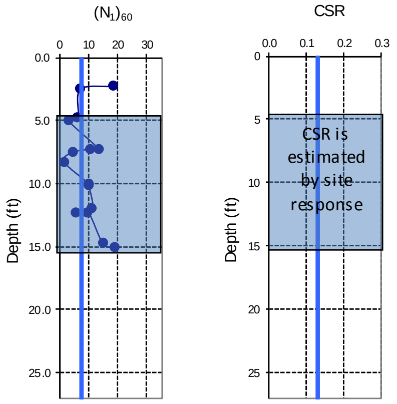

Depth (m)	Depth (ft)	CSR	N	C_N	C_R	$(N1)_{60}$	
0.69	2.3		13	2.00	0.62	18.3	M-7
0.76	2.5		5	2.00	0.63	7.2	SM-7
1.45	4.8		4	2.00	0.70	*6.3*	*M-7*
1.52	5.0		2	1.96	0.71	*3.1*	*SM-7*
2.21	7.3		9	1.76	0.76	*13.6*	*M-2*
2.21	7.3		7	1.76	0.76	*10.6*	*M-7*
2.29	7.5		3	1.75	0.76	*4.5*	*SM-7*
2.51	8.3		1	1.69	0.78	*1.5*	*M-3*
3.05	10.0		7	1.59	0.81	*10.2*	*SM-7*
3.09	10.2		7	1.58	0.81	*10.1*	*M-7*
3.66	12.0		8	1.49	0.84	*11.3*	*SM-7*
3.73	12.3		4	1.48	0.84	*5.6*	*M-3*
3.73	12.3		7	1.48	0.84	*9.8*	*M-7*
4.50	14.8		11	1.38	0.87	15.0	M-7
4.57	15.0		14	1.37	0.87	19.0	SM-7
					**Mean:**	7.9	
					**St. Dev.**	3.8	

Depth (ft)	FC	sand	silt	clay	D50	
2.3	31.0	69.0	18.0	13.0	0.1	M-7
2.5	-	-	-	-	-	SM-7
4.8	*-*	*-*	*-*	*-*	*-*	*M-7*
5.0	*-*	*-*	*-*	*-*	*-*	*SM-7*
7.3	*-*	*-*	*-*	*-*	*-*	*M-2*
7.3	*24.0*	*76.0*	*14.0*	*10.0*	*0.1*	*M-7*
7.5	*-*	*-*	*-*	*-*	*-*	*SM-7*
8.3	*-*	*-*	*-*	*-*	*-*	*M-3*
9.4	*21.0*	*79.0*	*11.0*	*10.0*	*0.1*	*M-7*
10.0	*-*	*-*	*-*	*-*	*-*	*SM-7*
10.2	*16.0*	*84.0*	*9.0*	*7.0*	*0.1*	*M-7*
12.0	*-*	*-*	*-*	*-*	*-*	*SM-7*
12.3	*-*	*-*	*-*	*-*	*-*	*M-3*
12.3	*18.0*	*82.0*	*11.0*	*7.0*	*0.1*	*M-7*
14.8	22.0	78.0	15.0	7.0	0.1	M-7
15.0	-	-	-	-	-	SM-7
	19.8	80.3	11.3	8.5	0.106	
	3.5	3.5	2.1	1.7	0.005	

**Case number:**	118
**Earthquake:**	1987 Superstition Hills
**Magnitude:**	6.54 (Mw)
**Location:**	Radio Tower B1
**References:**	James Gerard Bierschwale. (1984)<br>Bennett et al. (1984) and Fear et al. (1995)
**Nature of Failure:**	No surface evidence of liquefaction
**Comments:**	The Imperial Valley lies in the southern part of the Salton Basin, which was formed by northward extension of the crustal rifting that opened the Gulf of California. The basin is underlain by as much ad 20,000 ft of Tertiary and Quaternary marine, continental, and lacustrine sediment. Holocene sediment is as thick as 330 ft.<br>The principal rivers are the Alamo and New Rivers along which many sand boils were developed on fluvial deposits.<br>A: very poorly sorted, soft brown clay and silt-flood plain deposit<br>B: brown, loose to med.dense silt to fine sand- sediment at base is well sorted very fine sand fining upwards to poorly sorted silt-channel deposit<br>C: very stiff brown clayey silt to clay-lacustrine<br>D: med dense sand<br>PGA was recorded at the surface and at 6.7 m depth of Wildlife site as 0.18 and 0.17g respectively. The epicentral distance is ~30.2 km same as Wildlife site. Andrus and Stoke (NCEER, 97) predicted the PGA at the site as 0.2 g same as the PGA at Wildlife site. Site response analyses results also verify that<br>Energy ratio ~68 %

Summary of Data

	Cetin et al. (2016)	Idriss&Boulanger (2010)	Seed et.al. (1984)		Cetin et al. (2016)	Idriss&Boulanger (2010)	Seed et.al. (1984)
Liquefied?	No	No		D_{50}	0.074 ± 0.037		
Data Class	B			% Fines	43.5 ± 29.0	64.0	
Critical Depth Range	9.8 - 18.0	11.2		% PI			
Depth to GWT (ft)	6.6	6.9					
σ_v (psf)	1505.1 ± 160.1	1294.9		N	4.5 ± 3.4	2.0	
σ_v' (psf)	1044.5 ± 79.5	1044.3		C_R	0.86	0.85	
a_{max} (g)	0.200 ± 0.030	0.20		C_S	1.00	1.00	
r_d	0.94 ± 0.032	0.96		C_B	1.00	1.00	
CSR	0.176 ± 0.028	0.154		C_E	1.13	1.13	
Equivalent Magnitude	6.54	6.54		C_N	1.42	1.49	
MSF		1.29		$(N_1)_{60}$	6.2 ± 4.7	2.9	
CSRN		0.113					

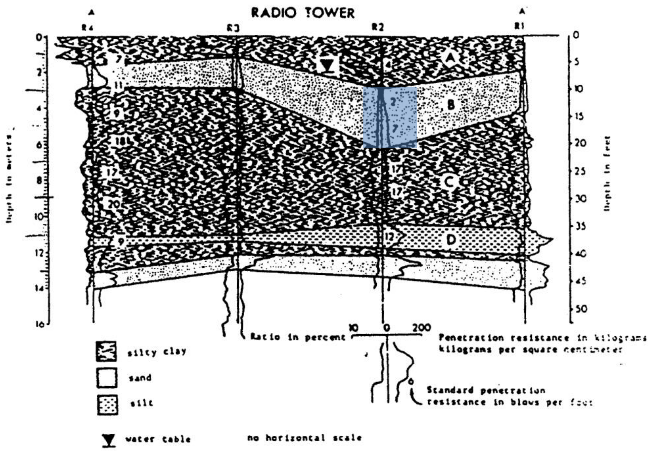

γ_m=100±3 pcf

γ_s=115±3 pcf

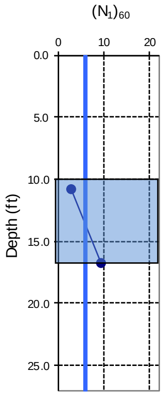

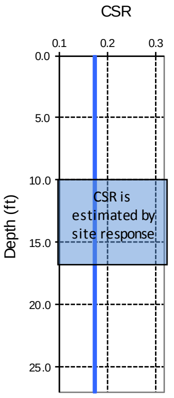

Depth (m)	Depth (ft)	CSR	N	C_N	C_R	$(N1)_{60}$
3.3	10.8		2	1.55	0.82	***2.9***
5.1	16.7		7	1.33	0.89	***9.5***
					**Mean:**	6.2
					**St. Dev.**	4.7

Depth (m)	FC	sand	silt	clay	D_{50}
3.3	***64.0***	***36.0***	***59.0***	***5.0***	***0.047***
5.1	***23.0***	***77.0***	***22.0***	***1.0***	***0.100***
23.15	*18.0*	*82.0*	*13.0*	*5.0*	*0.125*
	43.5	56.5	40.5	3.0	0.074
	29.0	29.0	26.2	2.8	0.037

**Case number:**	119
**Earthquake:**	Superstition Hills
**Magnitude:**	6.54 (Mw)
**Location:**	Radio Tower B2
**References:**	James Gerard Bierschwale. (1984)<br>Bennett et al. (1984) and Fear et al. (1995)
**Nature of Failure:**	No surface manifastation of liquefaction
**Comments:**	Imperial Valley lies in the southern part of the Salton Basin, which was formed by northward extension of the crustal rifting that opened the Gulf of California. The basin is underlain by as much as 20,000 ft of Tertiary and Quaternary marine, continental, and lacustrine sediment. Holocene sediment is as thick as 330 ft.<br>The principal rivers are the Alamo and New Rivers along which many sand boils were developed on fluvial deposits.<br>A: very poorly sorted, soft brown clay and silt-flood plain deposit<br>B: brown, loose to med.dense silt to fine sand- sediment at base is well sorted very fine sand fining upwards to poorly sorted silt-channel deposit<br>C: very stiff brown clayey silt to clay-lacustrine<br>D: med dense sand<br>PGA was recorded at the surface and at 6.7 m depth of Wildlife site as 0.18 and 0.17g respectively. The epicentral distance is ~30.2 km same as Wildlife site.<br>Andrus and Stoke (NCEER, 97) predicted the PGA at the site as 0.2 g same as the PGA at Wildlife site. Site response results suggest a PGA of 0.18g<br>Energy ratio ~68 %

**Summary of Data**	Cetin et al. (2016)	Idriss&Boulanger (2010)	Seed et.al. (1984)		Cetin et al. (2016)	Idriss&Boulanger (2010)	Seed et.al. (1984)
Liquefied?	No	No		D_{50}	0.100 ± 0.050		
Data Class	B			% Fines	18.0 ± 2.0	30.0	
Critical Depth Range	6.6 - 9.8	7.5		% PI			
Depth to GWT (ft)	6.6	6.9					
σ_v (psf)	852.8 ± 67.4	835.4		N	11.3 ± 1.4	11.0	
σ_v' (psf)	750.5 ± 39.0	793.6		C_R	0.77	0.80	
a_{max} (g)	0.180 ± 0.027	0.18		C_S	1.00	1.00	
r_d	0.99 ± 0.020	0.98		C_B	1.00	1.00	
CSR	0.132 ± 0.021	0.119		C_E	1.13	1.13	
Equivalent Magnitude	6.54	6.54		C_N	1.68	1.53	
MSF		1.29		$(N_1)_{60}$	16.6 ± 2.0	15.2	
CSRN		0.084					

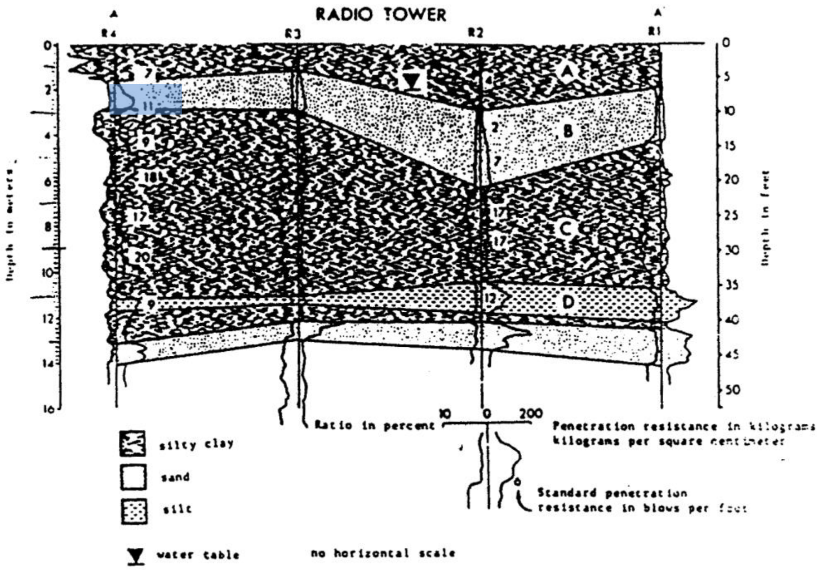

γ_m=100±3 pcf

γ_s=120±3 pcf

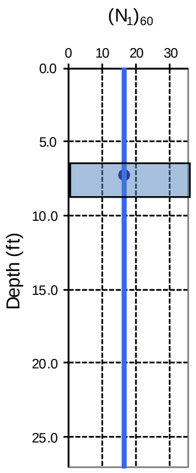

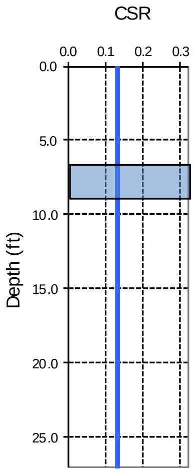

CSR is estimated by site response

Depth (m)	Depth (ft)	CSR	N	C_N	C_R	$(N1)_{60}$	FC	silt	clay	D50
2.3	7.3		11	1.74	0.76	***16.6***	***18.0***	***17.0***	***1.0***	***0.1***
					**Mean:**	16.6				
					**St. Dev.**	2.0	assigned			

**Case number:**	120
**Earthquake:**	Superstition Hills
**Magnitude:**	6.54 (Mw)
**Location:**	River Park A
**References:**	Youd et al. (1983), "Liquefaction sites, Imperial Valley, CA
**Nature of Failure:**	No surface manifastation of liquefaction
**Comments:**	Imperial Valley lies in the southern part of the Salton Basin, which was formed by northward extension of the crustal rifting that opened the Gulf of California. The basin is underlain by as much as 20,000 ft of Tertiary and Quaternary marine, continental, and lacustrine sediment. Holocene sediment is as thick as 330 ft.<br>The principal rivers are the Alamo and New Rivers along which many sand boils were developed on fluvial deposits.<br>River Park Site is ~30 km from the epicenter of the earthquake. PGA's values of 0.46 and 0.16 g were recorded at Parachute (15 kms southwest) and Brawley (~8 kms east) stations, respectively. Site response analysis result suggest a PGA value of 0.19 g.<br>ASTM D1586-67 procedures were followed for SPT tests. 10" outside diameter hollow-stem auger was used with Safe-T Driver and safety hammer.<br>Energy ratio ~68 %<br>SPT blowcounts were taken in 1979 and 1981.

Summary of Data

	Cetin et al. (2016)	Idriss&Boulanger (2010)	Seed et.al. (1984)		Cetin et al. (2016)	Idriss&Boulanger (2010)	Seed et.al. (1984)
Liquefied?	No	No		D_{50}	0.010 ± 0.050		
Data Class	B			% Fines	91.0 ± 2.0	80.0	
Critical Depth Range	1.0 - 5.9	5.9		% PI			
Depth to GWT (ft)	1.0	1.0					
σ_v (psf)	369.8 ± 90.3	731.0		N	2.7 ± 2.3	3.0	
σ_v' (psf)	216.8 ± 42.7	417.7		C_R	0.66	0.80	
a_{max} (g)	0.190 ± 0.029	0.19		C_S	1.00	1.00	
r_d	0.99 ± 0.010	0.98		C_B	1.00	1.00	
CSR	0.209 ± 0.038	0.210		C_E	1.13	1.13	
Equivalent Magnitude	6.54	6.54		C_N	2.00	1.70	
MSF		1.29		$(N_1)_{60}$	4.0 ± 3.4	4.6	
CSRN		0.149					

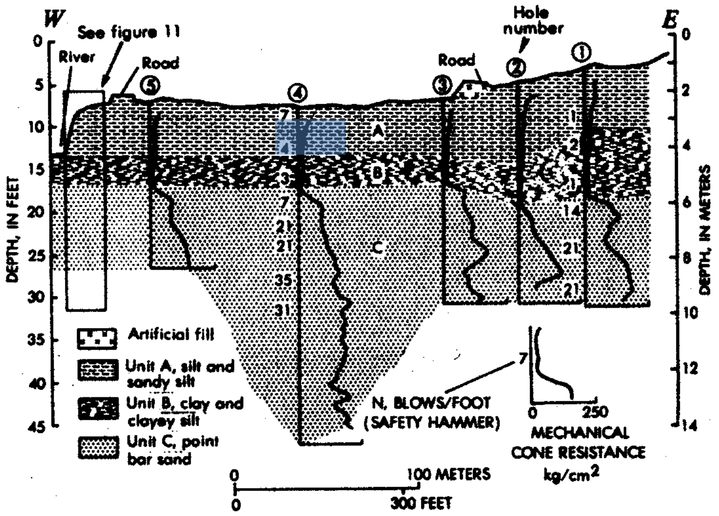

γ_m=100±3 pcf

γ_s=110±3 pcf

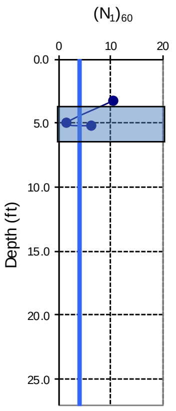

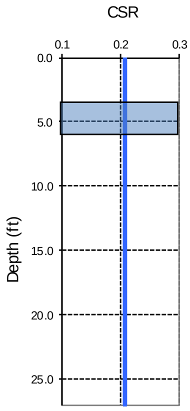

CSR is estimated by site response

Depth (m)	Depth (ft)	CSR	N	C_N	C_R	$(N1)_{60}$	
1	3.3		7	2.00	0.66	10.4	BH-4 (1979)
1.52	5.0		1	2.00	0.71	***1.6***	BH-15 (1981)
1.6	5.2		4	2.00	0.71	***6.5***	BH-4 (1979)
					**Mean:**	4.0	
					**St. Dev.**	3.4	

FC	sand	silt	clay	D50
-	-	-	-	0.071
91.0	*9.0*	*74.0*	*17.0*	*0.010*
-	*-*	*-*	*-*	*-*
91.0	9.0	74.0	17.0	0.010
2.0	-	-	-	0.050

**Case number:**	121
**Earthquake:**	Superstition Hills
**Magnitude:**	6.54 (Mw)
**Location:**	River Park C
**References:**	Youd et al. (1982), "Liquefaction sites, Imperial Valley, CA
**Nature of Failure:**	No surface manifastation of liquefaction
**Comments:**	The Imperial Valley lies in the southern part of the Salton Basin, which was formed by northward extension of the crustal rifting that opened the Gulf of California. The basin is underlain by as much as 20,000 ft of Tertiary and Quaternary marine, continental, and lacustrine sediment. Holocene sediment is as thick as 330 ft.<br>The principal rivers are the Alamo and New Rivers along which many sand boils were developed on fluvial deposits.<br>River Park Site is ~30 km from the epicenter of the earthquake. PGA's values of 0.46 and 0.16 g were recorded at Parachute (15 kms southwest) and Brawley (~8 kms east) stations, respectively. Site response analysis result suggest a PGA value of 0.19 g.<br>ASTM D1586-67 procedures were followed for SPT tests. 10" outside diameter hollow-stem auger was used with Safe-T Driver and safety hammer.<br>Energy ratio ~68 %<br>SPT blowcounts were taken in 1979 and 1981.

Summary of Data

	Cetin et al. (2016)	Idriss&Boulanger (2010)	Seed et.al. (1984)		Cetin et al. (2016)	Idriss&Boulanger (2010)	Seed et.al. (1984)
Liquefied?	No	No		D_{50}	0.142 ± 0.020		
Data Class	A			% Fines	13.2 ± 4.4	18.0	
Critical Depth Range	11.0 - 17.0	14.1		% PI			
Depth to GWT (ft)	1.0	1.0					
σ_v (psf)	1595.0 ± 121.6	1733.5		N	12.4 ± 4.9	11.0	
σ_v' (psf)	783.8 ± 67.6	939.8		C_R	0.86	0.85	
a_{max} (g)	0.190 ± 0.029	0.19		C_S	1.00	1.00	
r_d	0.93 ± 0.025	0.95		C_B	1.00	1.00	
CSR	0.234 ± 0.037	0.218		C_E	1.13	1.13	
Equivalent Magnitude	6.54	6.54		C_N	1.64	1.44	
MSF		1.29		$(N_1)_{60}$	19.9 ± 7.8	15.2	
CSRN		0.154					

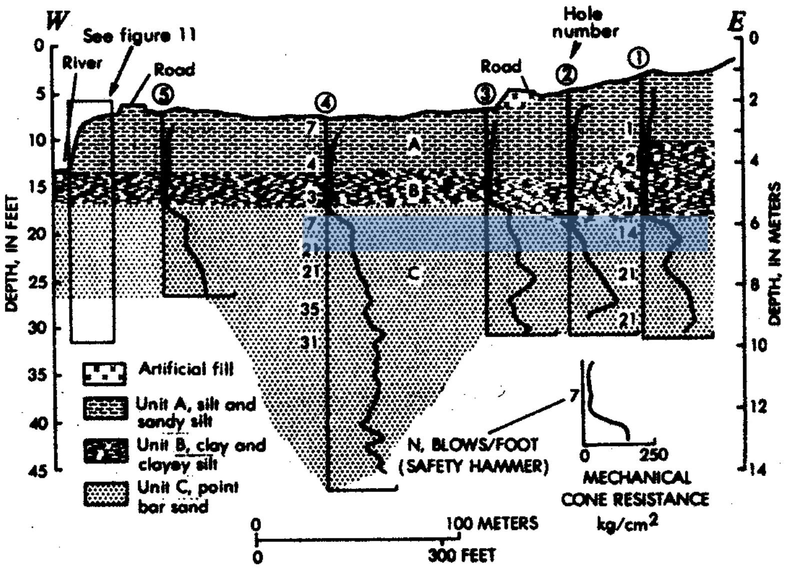

γ_m=100±3 pcf

γ_s=115±3 pcf

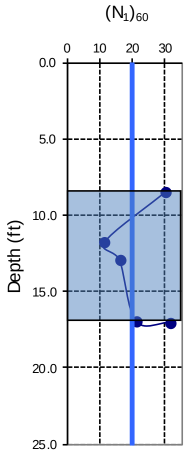

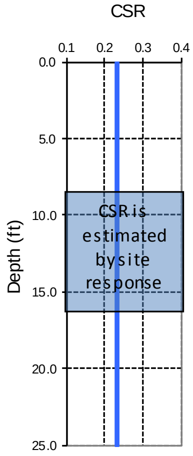

Depth (m)	Depth (ft)	CSR	N	C_N	C_R	$(N1)_{60}$	
2.6	8.5		17	2.00	0.78	***30.1***	BH-13
3.6	11.8		7	1.78	0.83	***11.8***	BH-4
3.96	13.0		10	1.70	0.85	***16.4***	BH-6
5.18	17.0		14	1.50	0.90	***21.3***	BH-1
5.2	17.1		21	1.50	0.90	32.0	BH-4
					**Mean:**	19.9	
					**St. Dev.**	7.8	

Depth (m)	FC	sand	silt	clay	D50	
**3.3**	**12**	**88**	**9**	**3**	**0.110**	BH-4
**3.5**	**13**	**87**	**11**	**2**	**0.120**	BH-4
**3.9**	**11**	**88**	**9**	**2**	**0.135**	BH-4
**4.4**	**11**	**89**	**10**	**1**	**0.140**	BH-4
**4.6**	**10**	**90**	**9**	**1**	**0.145**	BH-4
**5.1**	**10**	**90**	**10**	**0**	**0.145**	BH-4
**5.2**	**9**	**91**	**9**	**0**	**0.180**	BH-1
**5.2**	**14**	**86**	**14**	**0**	**0.170**	BH-1
**5.3**	**13**	**87**	**10**	**3**	**0.145**	BH-4
**5.7**	**18**	**82**	**14**	**4**	**0.140**	BH-4
**6.0**	**24**	**76**	**20**	**4**	**0.135**	BH-4
6.4	7	93	7	0	0.115	BH-1
7.8	6	94	6	0	0.145	BH-1
**Mean:**	13.2	86.7	11.4	1.8	0.142	
**St. Dev.**	4.4	4.3	3.4	1.5	0.020	

**Case number:**	122
**Earthquake:**	1987 Superstition Hills
**Magnitude:**	6.54 (Mw)
**Location:**	Wildlife B
**References:**	Bennett et al. (1984) and Fear et al. (1995)<br>James Gerard Bierschwale. (1984)<br>Youd, T.L., Wieczorek G. F., (1984), "Liquefaction during the 1981 and Previous Earthquakes Near Westmorland, CA" Open File Rep. 84-680
**Nature of Failure:**	Site has liquefied. Pore pressure ratios exceeded 1
**Comments:**	Imperial Valley lies in the southern part of the Salton Basin, which was formed by northward extension of the crustal rifting that opened the Gulf of California. The basin is underlain by as much ad 20,000 ft of Tertiary and Quaternary marine, continental, and lacustrine sediment. Holocene sediment is as thick as 330 ft.<br>The site is very closely monitored. Strong ground motion seismometers were installed at the surface and at 6.7 m depth. Piezometers were installed almost at every meter along the potentially liquefiable layer of thickness 4 m.<br>A: very loose and very soft interbedded micaceous sandy silt, silt and clayey silts (0-2.5 m) flood plain deposits<br>B: very loose to loose moderately sorted sandy silt (2.5-6.8m); point bar deposits<br>C: silty clay (medium to very stiff) and clayey silt (medium to stiff); lacustrine deposit.<br>PGA was recorded at the surface and at 6.7 m depth as 0.2 and 0.17g. respectively. The epicentral distance is 30.2 km.<br>Site response analyses results estimate PGA in two direction as 0.18 g and 0.23 g geometric mean of which is 0.205 g. Energy ratio ~68 %

**Summary of Data**	Cetin et al. (2016)	Idriss&Boulanger (2010)	Seed et.al. (1984)		Cetin et al. (2016)	Idriss&Boulanger (2010)	Seed et.al. (1984)
Liquefied?	Yes	Yes		D_{50}	0.109 ± 0.038		
Data Class	A			% Fines	26.2 ± 7.6	30.0	
Critical Depth Range	9.0 - 22.0	15.1		% PI			
Depth to GWT (ft)	3.0	3.9					
σ_v (psf)	1800.0 ± 262.9	1817.0		N	7.9 ± 3.5	7.1	
σ_v' (psf)	1020.0 ± 131.5	1127.8		C_R	0.88	0.95	
a_{max} (g)	0.205 ± 0.031	0.21		C_S	1.00	1.00	
r_d	0.82 ± 0.035	0.94		C_B	1.00	1.00	
CSR	0.192 ± 0.031	0.216		C_E	1.13	1.13	
Equivalent Magnitude	6.54	6.54		C_N	1.44	1.36	
MSF		1.29		$(N_1)_{60}$	11.3 ± 5.0	10.3	
CSRN		0.157					

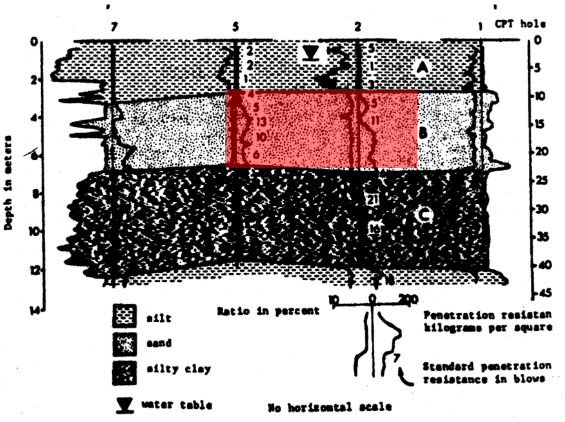

γ_m=100±3 pcf

γ_s=120±3 pcf

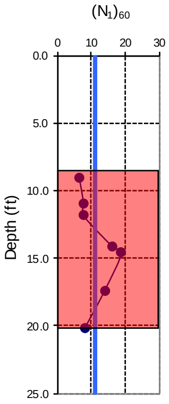

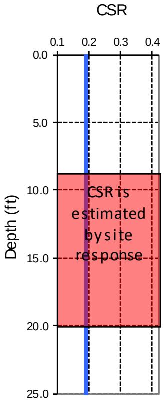

Depth (m)	Depth (ft)	CSR	N	C_N	C_R	$(N1)_{60}$	
2.75	9.0		4	1.81	0.79	***6.5***	Hole-5
3.35	11.0		5	1.67	0.82	***7.8***	Hole-2
3.6	11.8		5	1.62	0.83	***7.6***	Hole-5
4.3	14.1		11	1.50	0.86	***16.2***	Hole-2
4.45	14.6		13	1.48	0.87	***18.9***	Hole-5
5.3	17.4		10	1.37	0.90	***14.0***	Hole-5
6.15	20.2		6	1.28	0.93	***8.1***	Hole-5
					**Mean:**	11.3	
					**St. Dev.**	5.0	

Depth (ft)	FC	Sand	Silt	Clay	D50	
10.5	***34.0***	***66.0***	***34.0***	***0.0***	***0.2***	Hole-2
11.2	***17.0***	***83.0***	***17.0***	***0.0***	***0.1***	Hole-2
13.5	***27.0***	***73.0***	***26.0***	***1.0***	***0.1***	Hole-2
14.2	***20.0***	***80.0***	***20.0***	***0.0***	***0.1***	Hole-2
18.3	***33.0***	***67.0***	***29.0***	***4.0***	***0.1***	Hole-2
22.8	*18.0*	*82.0*	*17.0*	*1.0*	*0.1*	Hole-2
**Mean:**	26.2	73.8	25.2	1.0	0.109	
**St. Dev.**	7.6	7.6	6.8	1.7	0.038	

**Case number:**	123
**Earthquake:**	Loma Prieta
**Magnitude:**	6.93 (Mw) Pacific Earthquake Engineering Research Center: NGA Database
**Location:**	Alameda Bay Farm Island (Dike Location)
**References:**	Mitchell et al. (1994), "Insitu test results from four Loma Prieta Earthquake liquefaction sites: SPT, CPT, DMT and Shear Wave Velocity<br>Kayen et al. (1998), "Soil Liquefaction in the East Bay during the earthquake
**Nature of Failure:**	No surface manifestation of liquefaction.<br>Site was improved by vibraflotation
**Comments:**	Located immediately north of Oakland International Airport<br>Most of the western part of Bay Farm Island consists of sandy hydraulic fill, underlain by bay mud and deeper stiffer soil.<br>Considerable liquefaction occurred at the northwest corner and at points along the west edge of the fill. Soil liquefaction in the northern part of the island was largely confined to artificially fill land. Numerous sand boils~ 3m in diameter erupted in Harbor Bay Plaza area.<br>Fill in the perimeter dike area was densified by vibroflotation.<br>Densification prevented soil liquefaction of the western-perimeter dike.<br>Complex stratigraphy of thin (<1m thick) interbeds of sand and finer material<br>A layer of noncohesive sand and silty sand at 2-3 m. Below this layer to 6.5 m is a set of interlayered deposits.<br>PGA is recorded as 0.27 & 0.21g at the Alameda Naval Air Station seismogram which is located on a 152 m deep soil site.<br>Measurements performed after the earthquake.<br>Depth to water table is given as 2-3 m in Kayen et al (1998). However in Mitchell et al it was given as 5.5 m. P-wave plots suggests a water depth of 5.5 m.

Summary of Data

	Cetin et al. (2016)	Idriss&Boulanger (2010)	Seed et.al. (1984)		Cetin et al. (2016)	Idriss&Boulanger (2010)	Seed et.al. (1984)
Liquefied?	No	No	No	D_{50}	0.280 ± 0.050		? ? ?
Data Class	B			% Fines	7.0 ± 2.0	7.0	sand sand
Critical Depth Range	19.7 - 23.0	21.3	5.30 6.70 m	% PI			
Depth to GWT (ft)	9.8	9.8	3 m				
σ_v (psf)	2616.5 ± 82.1	2610.7	0.95 1.21 kg/cm²	N	37.1 ± 1.7	37.0	37 35
σ_v' (psf)	1899.9 ± 59.4	1900.6	0.72 0.84 kg/cm²	C_R	0.94	0.95	↑
a_{max} (g)	0.240 ± 0.036	0.24	0.270	C_S	1.30	1.30	0.900
r_d	0.99 ± 0.087	0.92	0.95 0.93	C_B	1.00	1.00	
CSR	0.212 ± 0.037	0.198	0.219 0.236	C_E	0.92	0.92	↓
Equivalent Magnitude	6.93	6.93		C_N	1.06	1.03	1.16 1.09
MSF		1.16		$(N_1)_{60}$	43.8 ± 2.0	43.3	42.2 37.5
CSRN		0.165	0.207 0.223				

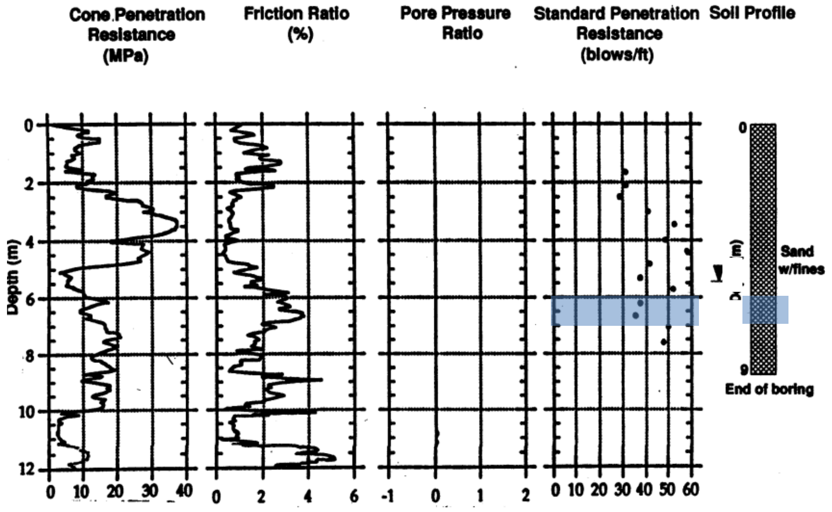

γ_m=120±3 pcf

γ_s=125±3 pcf

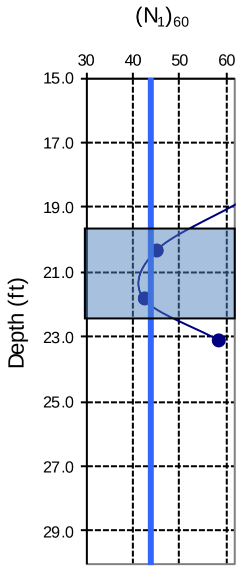

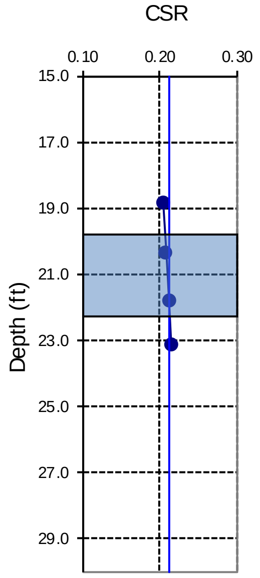

Depth (m)	Depth (ft)	CSR	N	C_N	C_R	$(N1)_{60}$
5.75	18.9	0.204	52	1.10	0.92	62.5
6.2	20.3	0.209	38	1.07	0.93	***45.2***
6.65	21.8	0.213	36	1.05	0.94	***42.4***
7.05	23.1	0.217	50	1.03	0.95	58.3
					**Mean:**	43.8
					**St. Dev.**	2.0

**Case number:**	124
**Earthquake:**	Loma Prieta
**Magnitude:**	6.93 (Mw) Pacific Earthquake Engineering Research Center: NGA Database
**Location:**	Farris Farm Site
**References:**	Holzer et al. (1994), "Observed and predicted ground deformation-Miller Farm lateral spread, Watsonville, California
**Nature of Failure:**	Lateral spread near the Pajaro River. South of the river ~0.2 m high head scarp which could be followed for 1.7 km. Maximum horizontal displacement of ~30 cm just west of the study area. Sand boils along the area affected by lateral spreading. Eyewitnesses reported that sediment-laden water flowed gently from vents on the lateral spread during and until 20 minuntes after the earthquake.
**Comments:**	Located at the south side of the Pajara River at Clint Miller Farms near Watsonville, CA.<br>Composed of younger and older holocene fluvial deposits<br>The upper 4-6 m of the younger flood-plain deposits is silty and contains only a few thin sand layers. In the depth interval from 4.6-9.1, sand percentages range 67-100. This layer includes most of the liquefied layer. Average fines content of the sandy layers if 15%, ranging from 22 % at Miller Farm to 8% at Farris Farm.<br>The depth to the water table was ~4.6 m 10 days after the main shock.<br>PGA is recorded in a four-story building 1km west of Miller Farm as 0.39 g<br>In Open File Report 95-663, Holzer et al., the PGA was given as 0.53g.<br>Watsonville region was shaken at MMI scale of 8.(~ 0.25-0.5 g ?)<br>The earthquake specific attenuation-relationship suggests for alluvium sites at 4.6 miles (closest distance to the fault) ~0.5-0.65 g<br>PGA was recorded as 0.36 g at Watsonville. Based on site response analyses results a PGA of 0.37 g was adopted for the analyses.<br>Strong ground motion lasted ~ 6 seconds.<br>SPT performed after the earthquake

**Summary of Data**	Cetin et al. (2016)	Idriss&Boulanger (2010)	Seed et.al. (1984)		Cetin et al. (2016)	Idriss&Boulanger (2010)	Seed et.al. (1984)
Liquefied?	Yes	Yes		D_{50}	0.200 ± 0.050		
Data Class	B			% Fines	8.0 ± 2.0	8.0	
Critical Depth Range	16.4 - 23.0	19.7		% PI			
Depth to GWT (ft)	14.8	14.8		D N fines			
σ_v (psf)	2066.9 ± 139.4	2213.9			9.0 ± 1.7	9.0	
σ_v' (psf)	1759.8 ± 79.4	1921.5		C_R	0.92	0.95	
a_{max} (g)	0.370 ± 0.056	0.37		C_S	1.00	1.00	
r_d	0.95 ± 0.020	0.93		C_B	1.00	1.00	
CSR	0.268 ± 0.041	0.259		C_E	1.13	1.13	
Equivalent Magnitude	6.93	6.93		C_N	1.10	1.05	
MSF		1.16		$(N_1)_{60}$	10.3 ± 2.0	10.2	
CSRN		0.221					

γ_m=100±3 pcf

γ_s=120±3 pcf

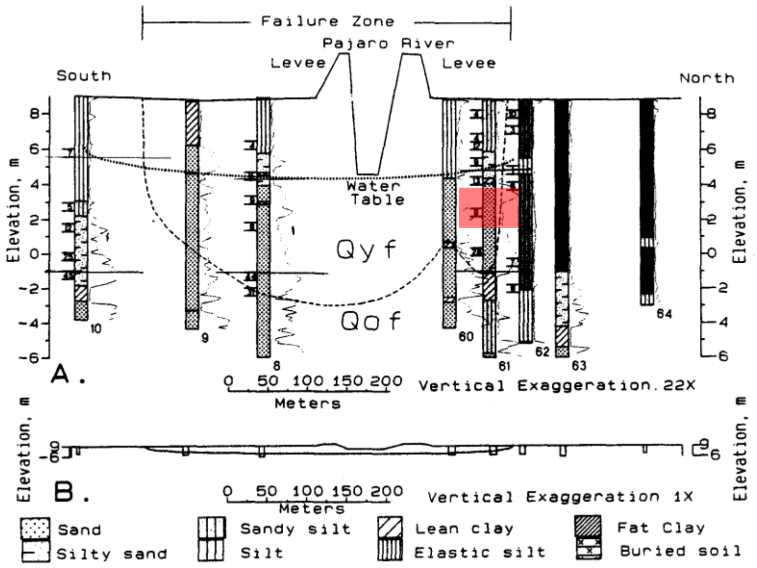

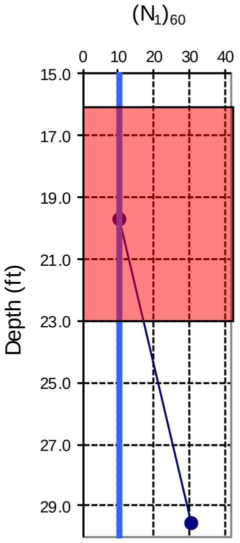

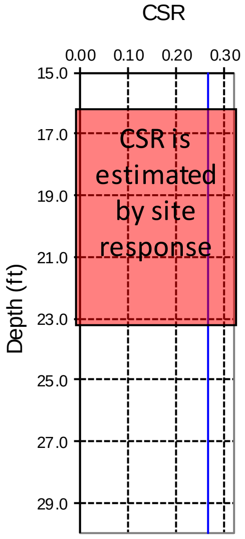

Depth (m)	Depth (ft)	CSR	N	C_N	C_R	$(N1)_{60}$	
6	19.7		9	1.10	0.92	***10.3***	
9	29.5		28	0.95	1.00	30.3	
					**Mean:**	10.3	
					**St. Dev.**	2.0	assigned

**Case number:**	125
**Earthquake:**	Loma Prieta
**Magnitude:**	6.93 (Mw) Pacific Earthquake Engineering Research Center: NGA Database
**Location:**	Port of Richmond (Hall Avenue)
**References:**	Mitchell et al. (1994), "Insitu test results from four Loma Prieta Earthquake liquefaction sites: SPT, CPT, DMT and Shear Wave Velocity<br>Kayen et al. (1998), "Soil Liquefaction in the East Bay during the earthquake
**Nature of Failure:**	No surface evidence of liquefaction was observed at Hall Avenue. However, between depths of about 1.5-3.5 m, the sampler sank under its own weight in a layer of very soft silty clay.
**Comments:**	The site is close to POR 2-3-4 (~ 1 km) by the Hall Street<br>The site represents the most distant point, approximately 85 km north of the rupture zone<br>The liquefaction zone overlies deposits of soft bay mud, underlain by deeper deposits of stiffer, overconsolidated noncohesive and cohesive soils<br>Up to 0.8 m depth the soil is characterized by oxidized, tan-brown crusted silty sand. 0.8-1.8 m oxidized tan-brown silty sand. 1.8-4.1 m oxidized tan-brown sandy clay to reduced sandy clay.4.1-7.8 m, is a hydraulic fill layer of olive-gray fine-silty sand containing shell fragments.<br>The thick deposits amplified the level of ground motion at the site<br>PGA was estimated as 0.18 g by performing a site response analysis (SHAKE) using the input motion from Richmond City Hall (PGA=0.13g) by Kayen et al.<br>Based on site response analyses results, a PGA of 0.14 was adopted.<br>Depth to water table in Kayen et al. (1998) was reported as 2.5 m contrary to 3.5 m reported in Mitchell et al. (1994)

**Summary of Data**	Cetin et al. (2016)	Idriss&Boulanger (2010)	Seed et.al. (1984)		Cetin et al. (2016)	Idriss&Boulanger (2010)	Seed et.al. (1984)
Liquefied?	No	No		D_{50}	0.090 ± 0.050		
Data Class	A			% Fines	30.0 ± 2.0	30.0	
Critical Depth Range	18.9 - 11.5	15.1		% PI			
Depth to GWT (ft)	11.5	11.5					
σ_v (psf)	1572.8 ± 146.1	1566.4		N	4.6 ± 3.2	4.6	
σ_v' (psf)	1342.4 ± 75.5	1336.7		C_R	0.88	0.95	
a_{max}	0.140 ± 0.021	0.14		C_S	1.10	1.10	
r_d	0.72 ± 0.013	0.95		C_B	1.00	1.00	
CSR	0.077 ± 0.012	0.102		C_E	0.92	0.92	
Equivalent Magnitude	6.93	6.93		C_N	1.26	1.28	
MSF		1.16		$(N_1)_{60}$	5.1 ± 3.6	5.7	
CSRN		0.084					

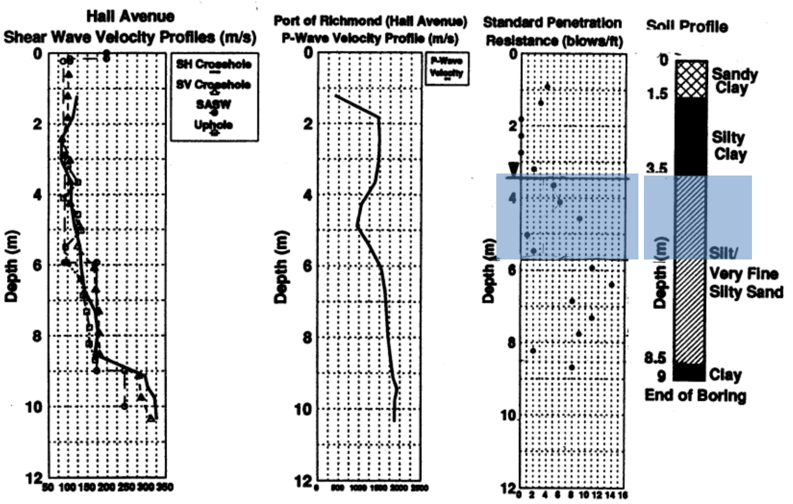

γ_m=100±3 pcf

γ_s=115±3 pcf

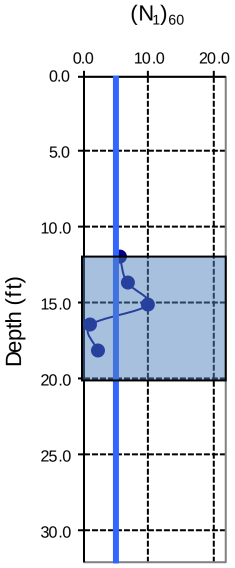

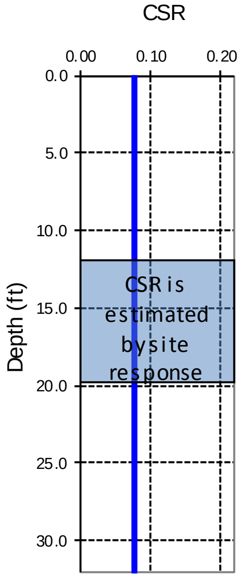

Depth (m)	Depth (ft)	CSR	N	C_N	C_R	$(N1)_{60}$
3.65	12.0		5	1.34	0.84	***5.7***
4.15	13.6		6	1.30	0.86	***6.7***
4.6	15.1		9	1.26	0.88	***10.0***
5	16.4		1	1.23	0.89	***1.1***
5.5	18.0		2	1.19	0.91	***2.2***
					**Mean:**	5.1
					**St. Dev.**	3.6

**Case number:**	126
**Earthquake:**	Loma Prieta
**Magnitude:**	6.93 (Mw) Pacific Earthquake Engineering Research Center: NGA Database
**Location:**	MBARI Facilities Building 3 EB-1
**References:**	Boulanger et al. (1996), "Liquefaction at Moass Landing During Loma Prieta Earthquake"
**Nature of Failure:**	Extensive ground deformations were observed along Sandholdt Road, including the portion alongside the MBARI facilities. Cracks parallel to the front line. Away from Sandholdt Road, surface evidence of liquefaction appeared to occur in localized areas.
**Comments:**	Located on Monterey Bay about midway between the cities of Santa Cruz and Monterey, CA.<br>The MBARI facilities include three main buildings and a pier located along Sandholt Road on the Moss Landing spit. Historical shoreline suggest that the shoreline in the vicinity of the site has fluctuated significantly. Shallower soil strata may have been deposited recently.<br>The area is underlain by Holocene deposits consisting of relatively thick sands offshore and estuarine and fluvial deposits onshore, up to about 60-m deep.<br>The Moss Landing spit is underlain by littoral soils comprising eolian, fluvial, estuarine, and beach deposits of gravel, sand, silt, and clay deposited in a shoreline environment within the zone of tidal fluctuation. Pleistocene and miocene deposits extend to a depth of ~2000 m<br>The site is underlain by an 8-12 m thick layer of poorly graded sand with interlayers of gravelly sand and sandy gravel in its lower portion and occasional thin layers of clayey silt throughout.<br>PGA was recorded in a four-story building 8km from rupture plane as 0.39 g (alluvial site). Also San Juan Bautista seismographic station, ~20 km from the rupture plane recorded 0.15 g (stiff alluvium). The Moss Landing area is located ~21 km from the rupture plane. Due to deep alluvium which is soft near the surface, PGA at the site was predicted in the range of 0.2-0.3g<br>Based on site response analyses results, a PGA value of 0.24 g was adopted.<br>EB-1 and EB-5 are ~50ft apart<br>Difficult to choose a representative SPT value<br>SPT performed after the earthquake

Summary of Data

	Cetin et al. (2016)	Idriss&Boulanger (2010)	Seed et.al. (1984)		Cetin et al. (2016)	Idriss&Boulanger (2010)	Seed et.al. (1984)
Liquefied?	No	No		D_{50}	0.600 ± 0.050		
Data Class	B			% Fines	1.0 ± 2.0	1.0	
Critical Depth Range	6.6 - 9.8	6.6		% PI			
Depth to GWT (ft)	6.6	6.6					
σ_v (psf)	926.8 ± 71.4	731.0		N	18.5 ± 1.6	18.0	
σ_v' (psf)	824.5 ± 42.3	731.0		C_R	0.77	0.80	
a_{max} (g)	0.240 ± 0.036	0.28		C_S	1.00	1.00	
r_d	0.99 ± 0.007	0.99		C_B	1.00	1.00	
CSR	0.174 ± 0.027	0.179		C_E	1.00	1.00	
Equivalent Magnitude	6.93	6.93		C_N	1.60	1.57	
MSF		1.16		$(N_1)_{60}$	22.9 ± 2.0	22.6	
CSRN		0.140					

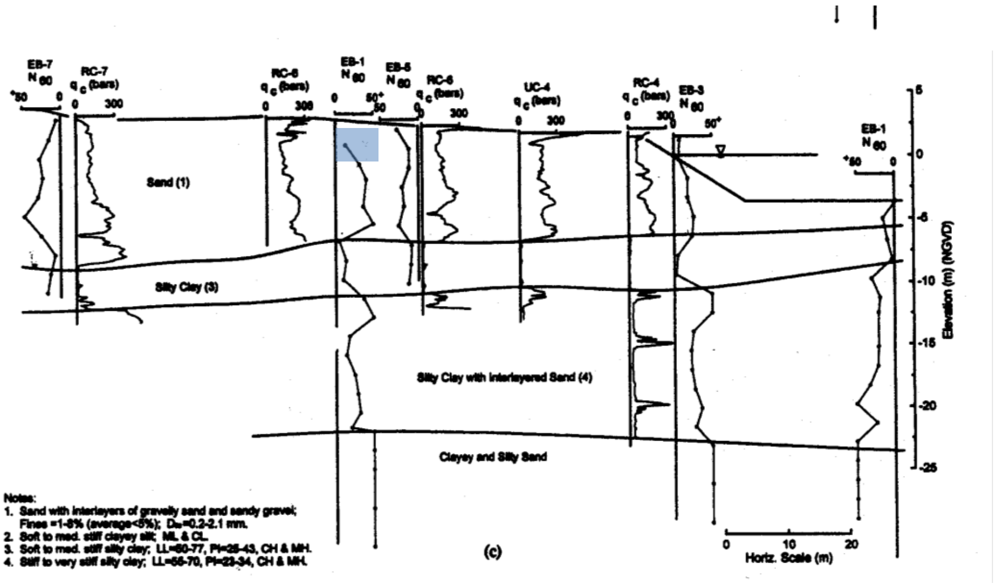

γ_m=110±3 pcf

γ_s=125±3 pcf

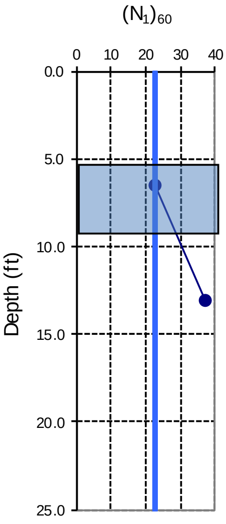

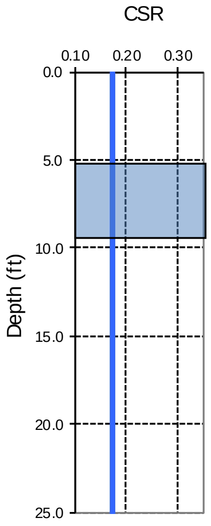

CSR is estimated by site response

Depth (m)	Depth (ft)	CSR	N	C_N	C_R	$(N1)_{60}$
2	6.6		18	1.71	0.74	***22.9***
4	13.1		32	1.37	0.85	37.2
					**Mean:**	22.9
					**St. Dev.**	2.0

assigned

**Case number:**	127
**Earthquake:**	Loma Prieta
**Magnitude:**	6.93 (Mw) Pacific Earthquake Engineering Research Center: NGA Database
**Location:**	MBARI Facilities Building 3 EB-5
**References:**	Boulanger et al. (1996), "Liquefaction at Moss Landing During Loma Prieta Earthquake"
**Nature of Failure:**	Extensive ground deformations were observed along Sandholdt Road, including the portion alongside the MBARI facilities. Cracks parallel to the front line. Away from Sandholdt Road, surface evidence of liquefaction appeared to occur in localized areas.
**Comments:**	Located on Monterey Bay about midway between the cities of Santa Cruz and Monterey, CA.<br>The MBARI facilities include three main buildings and a pier located along Sandholt Road on the Moss Landing spit. Historical shoreline suggest that the shoreline in the vicinity of the site has fluctuated significantly. Shallower soil strata may have been deposited recently.<br>The area is underlain by Holocene deposits consisting of relatively thick sands offshore and estuarine and fluvial deposits onshore, up to about 60-m deep.<br>The Moss Landing spit is underlain by littoral soils comprising eolian, fluvial, estuarine, and beach deposits of gravel, sand, silt, and clay deposited in a shoreline environment within the zone of tidal fluctuation. Pleistocene and miocene deposits extend to a depth of ~2000 m<br>The site is underlain by an 8-12 m thick layer of poorly graded sand with interlayers of gravelly sand and sandy gravel in its lower portion and occasional thin layers of clayey silt throughout.<br>PGA is recorded in a four-story building 8km from rupture plane as 0.39 g which is underlain by alluvium. Also San Juan Bautista seismogram, ~20 km from the rupture plane recorded 0.15 g (stiff alluvium). The Moss Landing area is located ~21 km from the rupture plane. Due to deep alluvium which is soft near the surface, PGA at the site is predicted between 0.2-0.3 g.<br>Based on site response analyses results, PGA is adopted as 0.27 g, slightly higher than the value adopted by authors of the source document (0.25 g)<br>EB-1 and EB-5 are ~50ft apart.<br>SPT performed before the earthquake.

**Summary of Data**	Cetin et al. (2016)	Idriss&Boulanger (2010)	Seed et.al. (1984)		Cetin et al. (2016)	Idriss&Boulanger (2010)	Seed et.al. (1984)
Liquefied?	No	No		D_{50}	0.600 ± 0.050		
Data Class	A			% Fines	1.0 ± 2.0	1.0	
Critical Depth Range	5.9 - 21.0	11.2		% PI			
Depth to GWT (ft)	5.9	5.9					
σ_v (psf)	1592.8 ± 315.8	1315.8		N	15.2 ± 2.8	12.0	
σ_v' (psf)	1122.0 ± 160.7	981.6		C_R	0.86	0.85	
a_{max} (g)	0.270 ± 0.041	0.28		C_S	1.00	1.00	
r_d	0.99 ± 0.007	0.97		C_B	1.00	1.00	
CSR	0.247 ± 0.040	0.235		C_E	1.00	1.00	
Equivalent Magnitude	6.93	6.93		C_N	1.37	1.46	
MSF		1.16		$(N_1)_{60}$	17.8 ± 3.3	14.9	
CSRN		0.187					

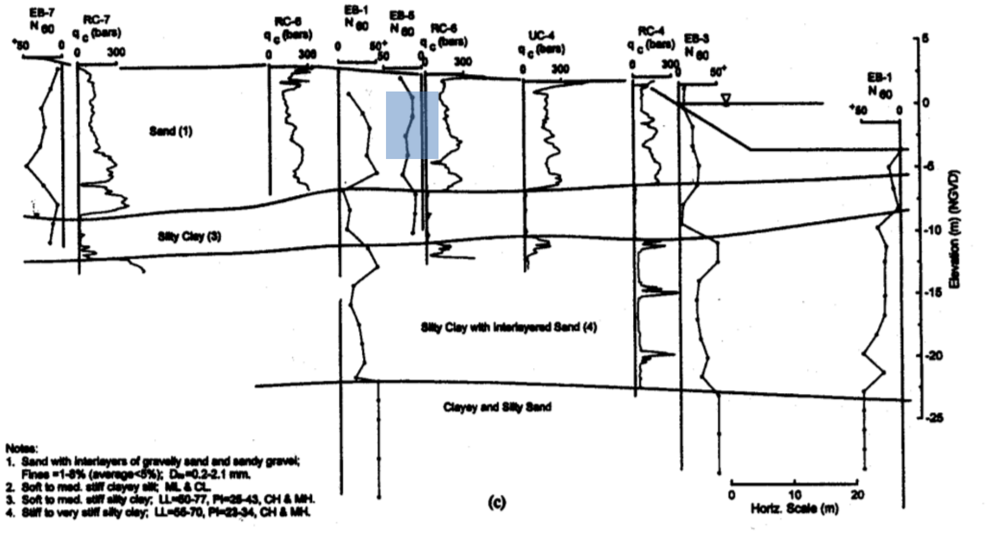

(c)

γ_m=110±3 pcf

γ_s=125±3 pcf

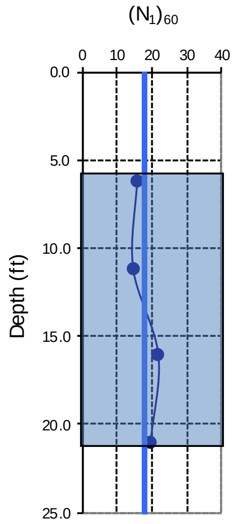

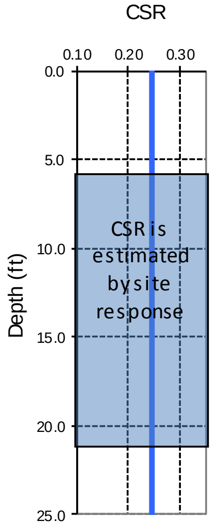

Depth (m)	Depth (ft)	CSR	N	C_N	C_R	$(N1)_{60}$
1.9	6.2		12	1.78	0.73	***15.7***
3.4	11.2		12	1.47	0.82	***14.5***
4.9	16.1		19	1.28	0.89	***21.6***
6.4	21.0		18	1.15	0.94	***19.4***
					**Mean:**	17.8
					**St. Dev.**	3.3

**Case number:**	128
**Earthquake:**	Loma Prieta
**Magnitude:**	6.93 (Mw) Pacific Earthquake Engineering Research Center: NGA Database
**Location:**	Miller Farm CMF 10
**References:**	Bennett and Tinsley, 1995, "Open File Report 95-663"
**Nature of Failure:**	Lateral spread, slide, and sand boils.
**Comments:**	Located at the east of Watsonville, Monterey.<br>Both SPT and CPT test were performed. Surface samples<br>were taken. Grain size characteristices were given in detail.<br>Usually SPT's were made 3-5 ft away from the CPT boreholes to obtain<br>samples. SPT procedures followed the guidelines outlined in D1586-67 (ASTM).<br>Hollow-stem augers (10" outside diameter, 4" inside diameter with split liners)<br>140 lb "inhole sampling hammer dropped at 30" height by "Safe-T-Driver" hoist<br>The overall efficiency of the hammer-hoist system is 68 %.<br>Undisturbed samples were taken using thin-walled Shelby tubes.<br>PGA is recorded in a four-story building 1km west of Miller Farm as 0.39 g.<br>In Open File Report 95-663, Holzer et al., the PGA was given as 0.53g.<br>Watsonville region was shaken at MMI scale of 8.(~ 0.25-0.50 g ?).<br>The earthquake specific attenuation-relationship suggests for alluvium sites<br>at 4.6 miles (closest distance to the fault) ~0.50-0.65 g<br>A PGA value of 0.36g was recorded at Watsonville.<br>Based on site response analyses results, a PGA value of 0.41 is adopted.<br>Strong ground motion lasted ~ 6 seconds.<br>CMF3-5-8-10 are ~ 1000 ft apart.<br>SPT performed after the earthquake.

Summary of Data

	Cetin et al. (2016)	Idriss&Boulanger (2010)	Seed et.al. (1984)		Cetin et al. (2016)	Idriss&Boulanger (2010)	Seed et.al. (1984)
Liquefied?	No	No		D_{50}	0.134 ± 0.026		
Data Class	B			% Fines	20.0 ± 2.0	20.0	
Critical Depth Range	23.0 - 32.8	27.6		% PI			
Depth to GWT (ft)	9.8	9.8					
σ_v (psf)	3338.3 ± 214.2	3299.9		N	18.3 ± 3.2	19.0	
σ_v' (psf)	2212.3 ± 121.3	2193.0		C_R	0.99	0.95	
a_{max} (g)	0.410 ± 0.062	0.39		C_S	1.00	1.00	
r_d	0.88 ± 0.024	0.89		C_B	1.00	1.00	
CSR	0.354 ± 0.054	0.338		C_E	1.13	1.13	
Equivalent Magnitude	6.93	6.93		C_N	0.98	0.99	
MSF		1.16		$(N_1)_{60}$	20.1 ± 3.5	20.2	
CSRN		0.292					

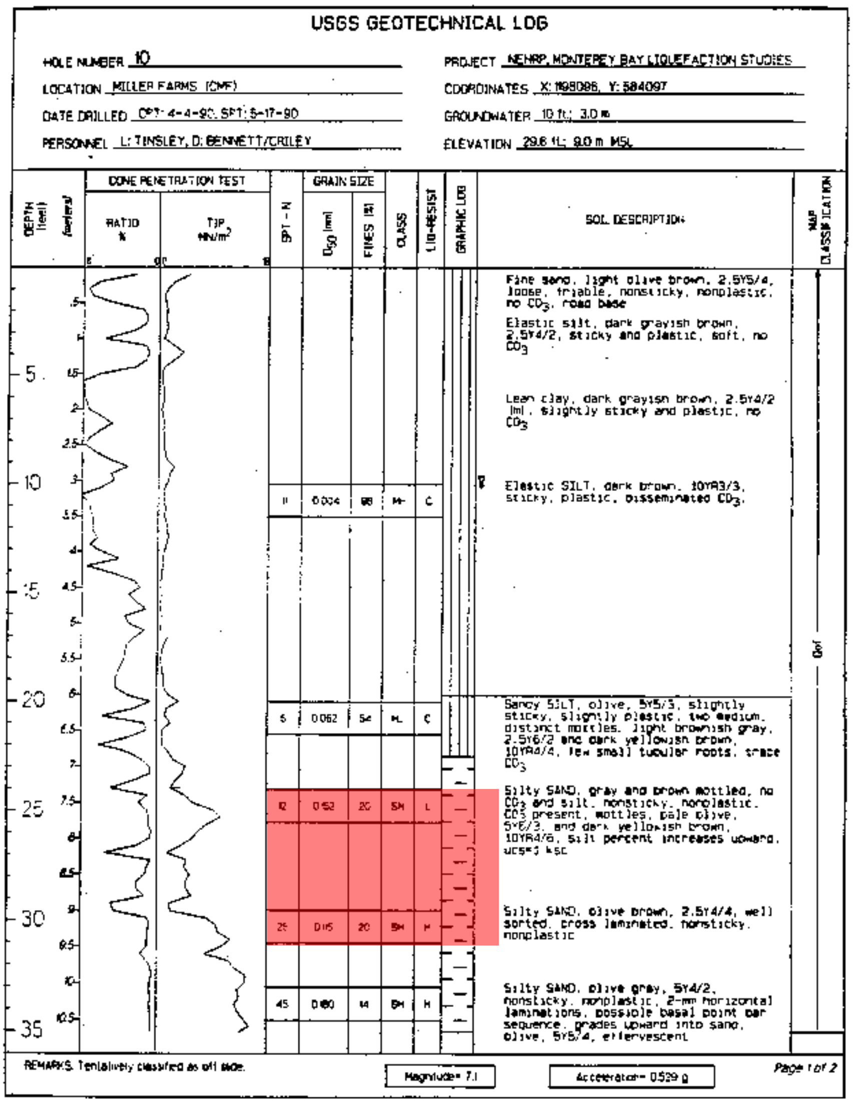

γ_m=110±3 pcf

γ_s=125±3 pcf

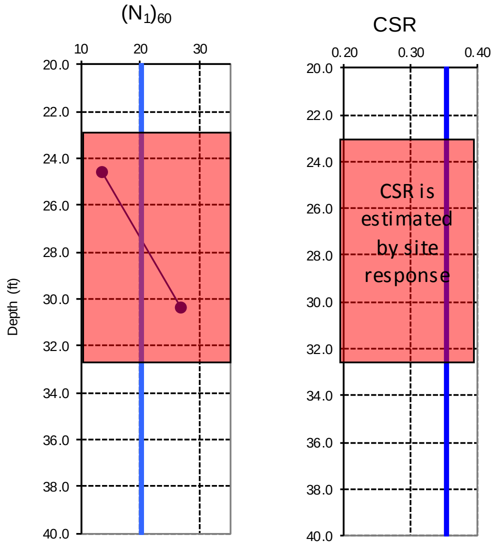

Depth (m)	Depth (ft)	CSR	N	C_N	C_R	$(N_1)_{60}$	FC %	D50
7.5	24.6		12	1.03	0.97	***13.5***	***20.0***	***0.152***
9.25	30.3		25	0.95	1.00	***26.8***	***20.0***	***0.115***
					**Mean:**	20.1	20.0	0.134
					**St. Dev.**	3.5	2.0	0.026

**Case number:**	129
**Earthquake:**	Loma Prieta
**Magnitude:**	6.93 (Mw) Pacific Earthquake Engineering Research Center: NGA Database
**Location:**	Miller Farm CMF 3
**References:**	Bennett and Tinsley, 1995, "Open File Report 95-663."
**Nature of Failure:**	Lateral spread, slide, and sand boils.
**Comments:**	Located at the east of Watsonville, Monterey.<br>Both SPT and CPT test were performed. Surface samples<br>were taken. Grain size characteristices were given in detail.<br>Usually SPT's were made 3-5 ft away from the CPT boreholes to obtain<br>samples. SPT procedures followed the guidelines outlined in D1586-67 (ASTM).<br>Hollow-stem augers (10" outside diameter, 4" inside diameter with split liners)<br>140 lb "inhole sampling hammer dropped at 30" height by "Safe-T-Driver" hoist.<br>The overall efficiency of the hammer-hoist system is 68 %.<br>Undisturbed samples were taken using thin-walled Shelby tubes.<br>PGA is recorded in a four-story building 1km west of Miller Farm as 0.39 g<br>In Open File Report 95-663, Holzer et al., the PGA was given as 0.53 g.<br>Watsonville region was shaken at MMI scale of 8.(~ 0.25-0.50 g ?).<br>The earthquake specific attenuation-relationship suggests for alluvium sites<br>at 4.6 miles (closest distance to the fault) ~0.50-0.65 g.<br>A PGA value of 0.36 g was recorded at Watsonville<br>Based on site response analyses results, a PGA value of 0.46 is adopted.<br>Strong ground motion lasted ~ 6 seconds.<br>CMF3-5-8-10 are ~ 1000 ft apart.<br>The SPT blowcount 26 at depth ~8m was eliminated due to high CPT tip<br>resistance at that depth. Unlikely to be liquefied?<br>SPT performed after the earthquake.

Summary of Data

	Cetin et al. (2016)	Idriss&Boulanger (2010)	Seed et.al. (1984)		Cetin et al. (2016)	Idriss&Boulanger (2010)	Seed et.al. (1984)
Liquefied?	Yes	Yes		D_{50}	0.120 ± 0.034		
Data Class	A			% Fines	27.3 ± 11.9	32.0	
Critical Depth Range	18.9 - 24.6	20.3		% PI			
Depth to GWT (ft)	18.7	16.1					
σ_v (psf)	2436.4 ± 132.5	2380.9		N	10.3 ± 3.7	9.2	
σ_v' (psf)	2247.1 ± 83.8	2109.4		C_R	0.94	0.95	
a_{max} (g)	0.460 ± 0.069	0.39		C_S	1.00	1.00	
r_d	0.83 ± 0.019	0.92		C_B	1.00	1.00	
CSR	0.263 ± 0.041	0.252		C_E	1.13	1.13	
Equivalent Magnitude	6.93	6.93		C_N	0.97	1.00	
MSF		1.16		$(N_1)_{60}$	10.7 ± 3.8	9.9	
CSRN		0.219					

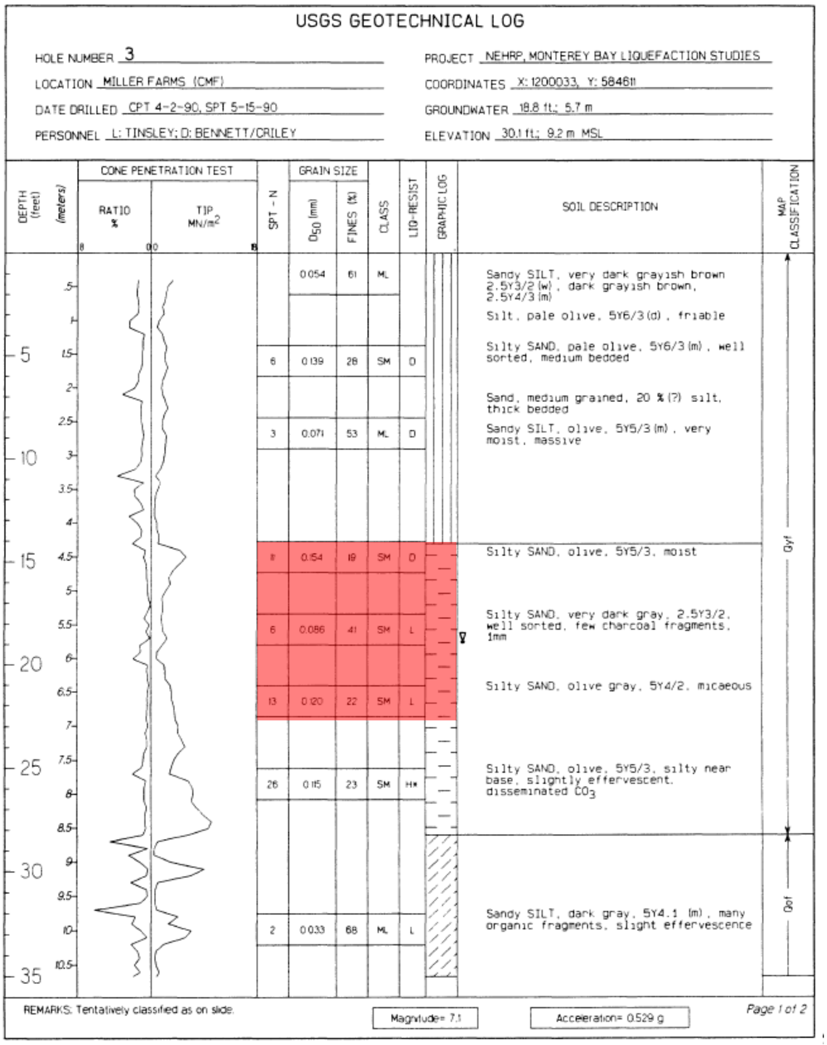

γ_m=120±3 pcf

γ_s=125±3 pcf

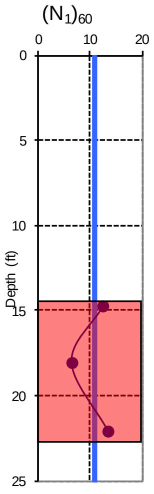

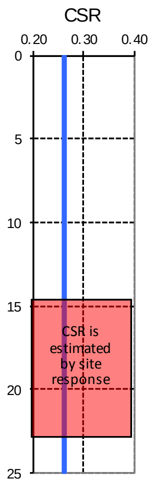

Depth (m)	Depth (ft)	CSR	N	C_N	C_R	$(N1)_{60}$	FC %	D_{50} (mm)
4.5	14.8		11	1.14	0.87	***12.4***	***19.0***	***0.154***
5.5	18.0		6	1.03	0.91	***6.4***	***41.0***	***0.086***
6.75	22.1		13	0.96	0.95	***13.5***	***22.0***	***0.120***
					**Mean:**	10.7	27.3	0.120
					**St. Dev.**	3.8	11.9	0.034

**Case number:**	130
**Earthquake:**	Loma Prieta
**Magnitude:**	6.93 (Mw) Pacific Earthquake Engineering Research Center: NGA Database
**Location:**	Miller Farm CMF 5
**References:**	Bennett and Tinsley, 1995, "Open File Report 95-663"
**Nature of Failure:**	Lateral spread, slide, and sand boils.
**Comments:**	Located at the east of Watsonville, Monterey.<br>Both SPT and CPT test were performed. Surface samples<br>were taken. Grain size characteristices were given in detail.<br>Usually SPT's were made 3-5 ft away from the CPT boreholes to obtain<br>samples. SPT procedures followed the guidelines outlined in D1586-67 (ASTM).<br>Hollow-stem augers (10" outside diameter, 4" inside diameter with split liners)<br>140 lb "inhole sampling hammer dropped at 30" height by "Safe-T-Driver" hoist.<br>The overall efficiency of the hammer-hoist system is 68 %.<br>Undisturbed samples were taken using thin-walled Shelby tubes.<br>PGA is recorded in a four-story building 1km west of Miller Farm as 0.39 g.<br>In Open File Report 95-663, Holzer et al., the PGA was given as 0.53 g.<br>Watsonville region was shaken at MMI scale of 8.(~ 0.25-0.50 g ?).<br>The earthquake specific attenuation-relationship suggests for alluvium sites<br>at 4.6 miles (closest distance to the fault) ~0.50-0.65 g.<br>A PGA value of 0.36 g was recorded at Watsonville<br>Based on site response analyses results, a PGA value of 0.41g is adopted<br>Strong ground motion lasted ~ 6 seconds.<br>CMF3-5-8-10 are ~ 1000 ft apart.<br>SPT performed after the earthquake.

Summary of Data

	Cetin et al. (2016)	Idriss&Boulanger (2010)	Seed et.al. (1984)		Cetin et al. (2016)	Idriss&Boulanger (2010)	Seed et.al. (1984)
Liquefied?	Yes	Yes		D_{50}	0.185 ± 0.050		
Data Class	B			% Fines	13.0 ± 2.0	13.0	
Critical Depth Range	18.0 - 27.9	23.0		% PI			
Depth to GWT (ft)	15.4	15.4					
σ_v (psf)	2793.6 ± 211.4	2715.1		N	20.0 ± 1.9	20.0	
σ_v' (psf)	2322.8 ± 116.6	2255.6		C_R	0.95	0.95	
a_{max} (g)	0.410 ± 0.062	0.39		C_S	1.00	1.00	
r_d	0.90 ± 0.016	0.91		C_B	1.00	1.00	
CSR	0.290 ± 0.044	0.280		C_E	1.13	1.13	
Equivalent Magnitude	6.9	6.9		C_N	0.95	0.97	
MSF		1.16		$(N_1)_{60}$	20.6 ± 2.0	20.9	
CSRN		0.243					

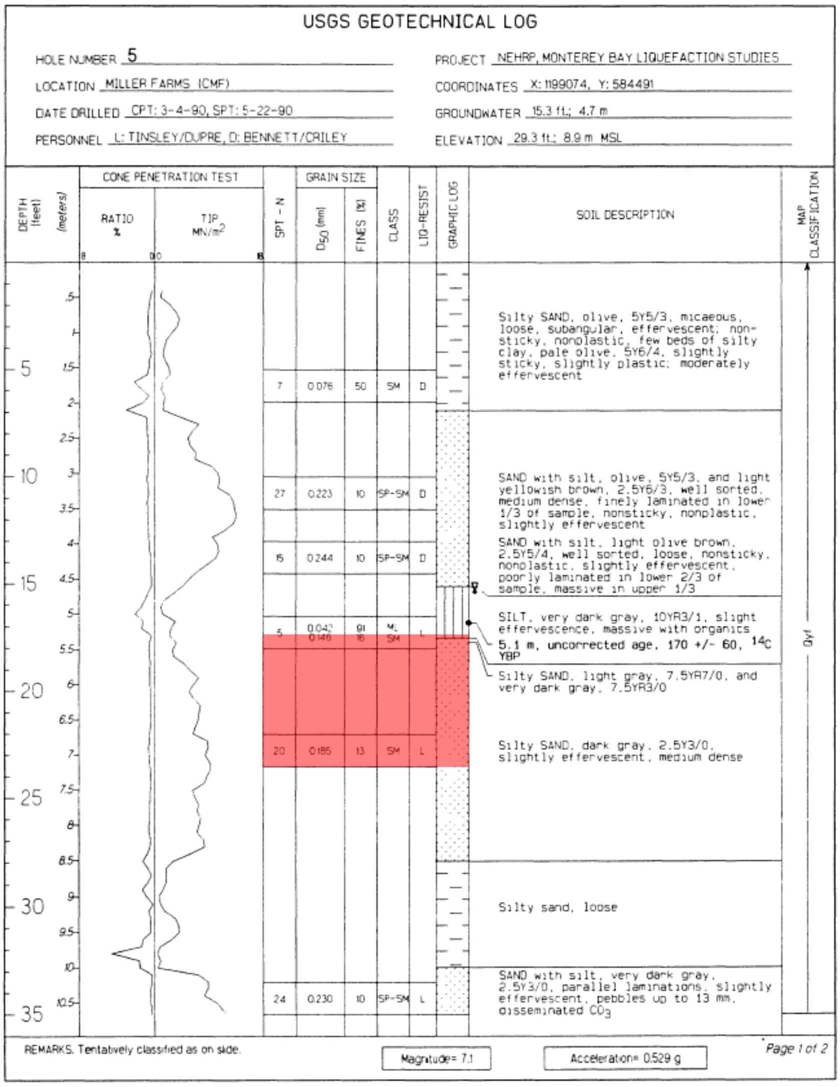

γ_m = 120±3 pcf

γ_s = 125±3 pcf

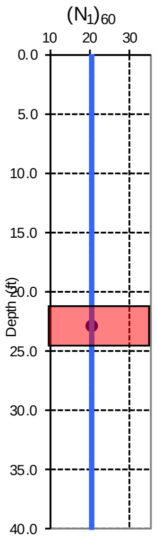

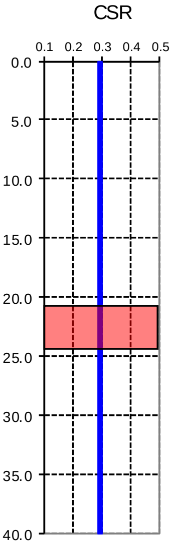

CSR is estimated by site response

Depth (m)	Depth (ft)	CSR	N	C_N	C_R	$(N1)_{60}$
7	23.0		20	0.95	0.95	***20.6***
					**Mean:**	20.6
					**St. Dev.**	2.0

FC %	D_{50} (mm)
13	***0.185***
16	0.146
13.0	0.185
2.0	0.050

**Case number:**	131
**Earthquake:**	Loma Prieta
**Magnitude:**	6.93 (Mw) Pacific Earthquake Engineering Research Center: NGA Database
**Location:**	Miller Farm CMF 8
**References:**	Bennett and Tinsley, 1995, "Open File Report 95-663"
**Nature of Failure:**	Lateral spread, slide, and sand boils.
**Comments:**	Located at the east of Watsonville, Monterey.<br>Both SPT and CPT test were performed. Surface samples<br>were taken. Grain size characteristices were given in detail.<br>Usually SPT's were made 3-5 ft away from the CPT boreholes to obtain<br>samples. SPT procedures followed the guidelines outlined in D1586-67 (ASTM).<br>Hollow-stem augers (10" outside diameter, 4" inside diameter with split liners)<br>140 lb "inhole sampling hammer dropped at 30" height by "Safe-T-Driver" hoist.<br>The overall efficiency of the hammer-hoist system is 68 %.<br>Undisturbed samples were taken using thin-walled Shelby tubes.<br>PGA is recorded in a four-story building 1km west of Miller Farm as 0.39 g<br>In Open File Report 95-663, Holzer et al., the PGA was given as 0.53 g.<br>Watsonville region was shaken at MMI scale of 8 (~ 0.25-0.50 g ?).<br>The earthquake specific attenuation-relationship suggests for alluvium sites<br>at 4.6 miles (closest distance to the fault) ~0.50-0.65 g.<br>A PGA value of 0.36 g was recorded at Watsonville<br>Based on site response analyses results, a PGA value of 0.46 g is adopted.<br>Strong ground motion lasted ~ 6 seconds.<br>CMF3-5-8-10 are ~ 1000 ft apart.<br>SPT performed after the earthquake.

Summary of Data

	Cetin et al. (2016)	Idriss&Boulanger (2010)	Seed et.al. (1984)		Cetin et al. (2016)	Idriss&Boulanger (2010)	Seed et.al. (1984)
Liquefied?	Yes	Yes		D_{50}	0.203 ± 0.085		
Data Class	B			% Fines	15.5 ± 2.1	25.0	
Critical Depth Range	16.4 - 26.2	19.7		% PI			
Depth to GWT (ft)	16.1	14.4					
σ_v (psf)	2237.5 ± 203.4	2318.3		N	9.0 ± 0.9	8.8	
σ_v' (psf)	1910.0 ± 108.0	1984.1		C_R	0.94	0.95	
a_{max} (g)	0.460 ± 0.069	0.39		C_S	1.00	1.00	
r_d	0.73 ± 0.013	0.93		C_B	1.00	1.00	
CSR	0.250 ± 0.040	0.274		C_E	1.13	1.13	
Equivalent Magnitude	6.93	6.93		C_N	1.05	1.03	
MSF		1.16		$(N_1)_{60}$	10.0 ± 1.0	9.8	
CSRN		0.234					

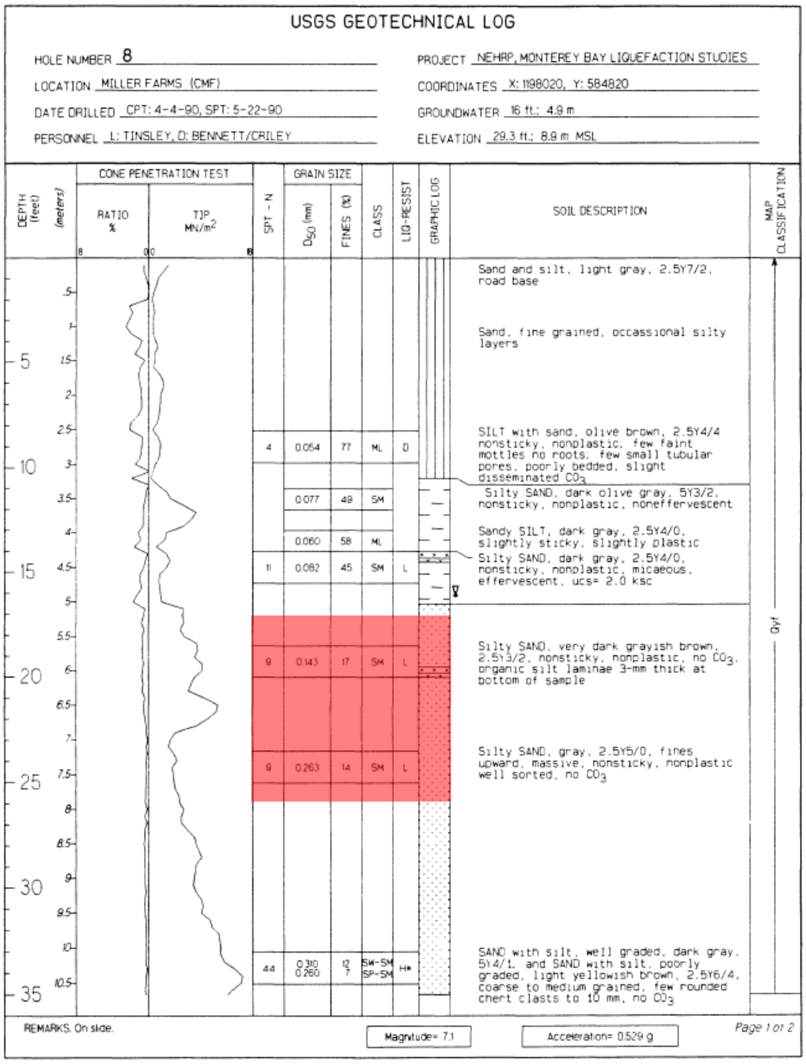

γ_m= 100 ± 3 pcf

γ_s= 120 ± 3 pcf

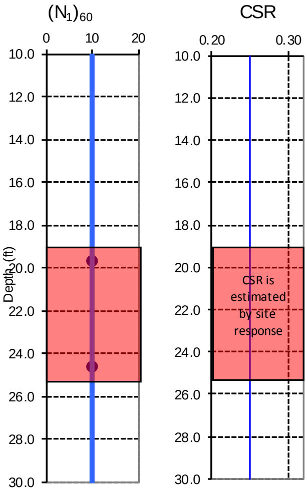

Depth (m)	Depth (ft)	CSR	N	C_N	C_R	$(N1)_{60}$	FC	D50
6	19.7		9	1.08	0.92	***10.2***	***17.0***	***0.143***
7.5	24.6		9	1.00	0.97	***9.9***	***14.0***	***0.263***
					**Mean:**	10.0	15.5	0.203
					**St. Dev.**	1.0	2.1	0.085

**Case number:**	132
**Earthquake:**	Loma Prieta
**Magnitude:**	6.93 (Mw) Pacific Earthquake Engineering Research Center: NGA Database
**Location:**	Port of Oakland (POO7-2)
**References:**	Mitchell et al. (1994), "Insitu test results from four Loma Prieta Earthquake liquefaction sites: SPT, CPT, DMT and Shear Wave Velocity<br>Kayen et al. (1998), "Soil Liquefaction in the East Bay during the earthquake
**Nature of Failure:**	Significant settlements (max ~ 30 cm.) occurred over most of the fill.<br>Significant lateral spreading along most of the fill, causing longitudinal fissures.<br>A 10 m diameter. 0.3 m high sand boil was observed in the paved container south of the C.F.S. Building near Berth 38.
**Comments:**	Located immediately south of the San Francisco - Oakland Bay Bridge<br>Most of the surface fills at the Port of Oakland were hydraulically placed to sea level, above which a combination of hydraulic fill and dumped fill was placed. A relatively thick asphalt was laid on top of the unimproved fill.<br>Underlain by 180 m of stiffer Pleistocene glacial and interglacial deposits.<br>Upper 3-4 m, surface layer of high penetration resistance; below this layer lies a looser deposits of marine sand. Liquefaction appears to have occurred in the depth range of 4-8 m.<br>PGA is recorded as 0.29 & 0.27g at the Oakland Outer Harbor seismogram which is located on a 155 m deep soil site.<br>PGA is estimated as 0.22 g based on site response analyses results.<br>Measurements performed after the earthquake.<br>The POO7-2 and POO7-3 are approximately 15 m apart.

**Summary of Data**	Cetin et al. (2016)	Idriss&Boulanger (2010)	Seed et.al. (1984)		Cetin et al. (2016)	Idriss&Boulanger (2010)	Seed et.al. (1984)
Liquefied?	Yes	Yes	yes	D_{50}	0.300 ± 0.050		? ? ?
Data Class	A			% Fines	3.0 ± 2.0	3.0	sand sand sand
Critical Depth Range	18.0 - 22.3	20.7	3 5.50 8.75	% PI			
Depth to GWT (ft)	9.8	9.8	3 m				
σ_v (psf)	2472.9 ± 98.6	2527.1	0.54 0.99 1.58kg/cm²	N	12.7 ± 3.1	14.4	10 10 12
σ_v' (psf)	1828.1 ± 64.1	1858.8	0.54 0.74 1.00kg/cm²	C_R	0.93	0.95	
a_{max}	0.220 ± 0.033	0.28	0.285	C_S	1.10	1.10	0.900
r_d	0.86 ± 0.018	0.92	0.97 0.95 0.91	C_B	1.00	1.00	
CSR	0.165 ± 0.025	0.229	0.18 0.234 0.266	C_E	0.92	0.92	
Equivalent Magnitude	6.93	6.93		C_N	1.08	1.11	1.3 1.15 1.0
MSF		1.16		$(N_1)_{60}$	12.8 ± 3.1	15.4	11.7 10.4 10.8
CSRN		0.194	0.17 0.221 0.251				

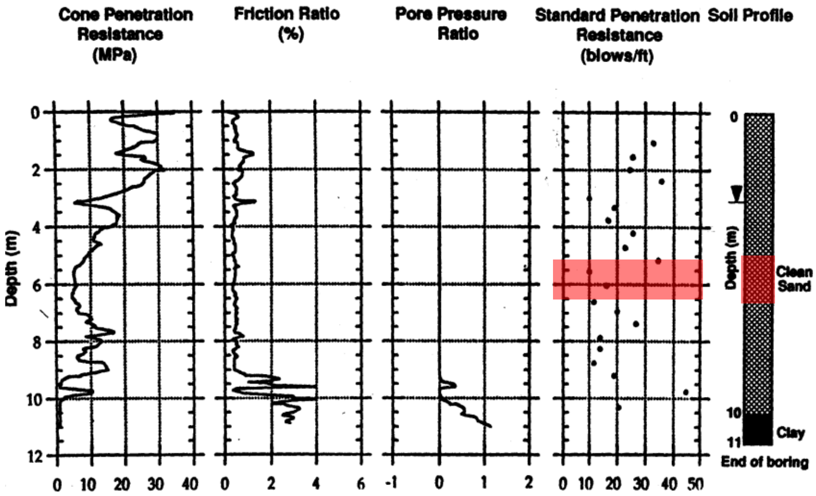

γ_m=120±3 pcf

γ_s=125±3 pcf

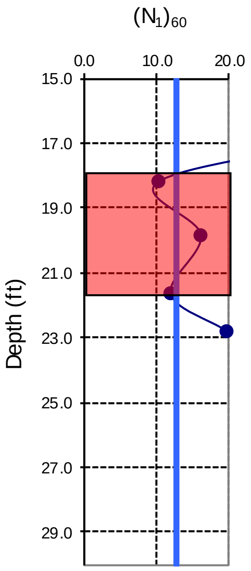

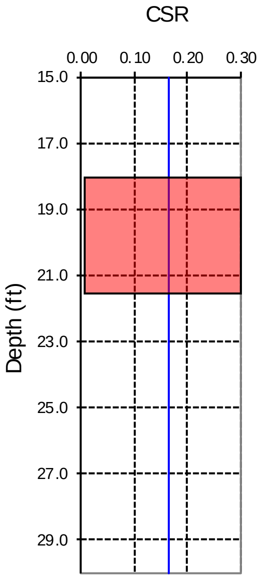

CSR is estimated by site response

Depth (m)	Depth (ft)	CSR	N	C_N	C_R	$(N1)_{60}$
5.15	16.9		35	1.14	0.90	36.1
5.55	18.2		10	1.11	0.91	***10.2***
6.05	19.8		16	1.08	0.93	***16.2***
6.6	21.7		12	1.05	0.94	***12.0***
6.95	22.8		20	1.03	0.95	19.8
					**Mean:**	12.8
					**St. Dev.**	3.1

**Case number:**	133
**Earthquake:**	Loma Prieta
**Magnitude:**	6.93 (Mw) Pacific Earthquake Engineering Research Center: NGA Database
**Location:**	Port of Oakland (POO7-3)
**References:**	Mitchell et al. (1994), "Insitu test results from four Loma Prieta Earthquake<br>liquefaction sites: SPT, CPT, DMT and Shear Wave Velocity<br>Kayen et al. (1998), "Soil Liquefaction in the East Bay during the earthquake
**Nature of Failure:**	Significant settlements (max ~ 30 cm.) occurred over most of the fill.<br>Significant lateral spreading along most of the fill, causing longitudinal fissures<br>A 10 m diameter. 0.3 m high sand boil was observed in the paved container<br>south of the C.F.S. Building near Berth 38.<br>Muddy sand unit at depths 5-7 m
**Comments:**	Located immediately south of the San Francisco - Oakland Bay Bridge.<br>Most of the surface fills at the Port of Oakland were hydraulically placed to<br>sea level, above which a combination of hydraulic fill and dumped fill was<br>placed. A relatively thick asphalt was laid on top of the unimproved fill<br>Underlain by 180 m of stiffer Pleistocene glacial and interglacial deposits<br>Upper 3-4 m, surface layer of high penetration resistance; Below this layer lies<br>a looser deposits of marine sand.<br>Borehole is characterized by high N values (~21) for the entire submerged<br>soil profile, except for a muddy-sand unit between 5-7m depth. Liquefied?<br>PGA is recorded as 0.29 & 0.27g at the Oakland Outer Harbor seismogram<br>which is located on a 155 m deep soil site.<br>PGA is estimated as 0.22 g based on site response analyses results.<br>Measurements performed after the earthquake.<br>The POO7-2 and POO7-3 are approximately 15 m apart.<br>The SPT blowcount (~22) at depth ~6.6 m does not appear on the borelog<br>excel spreadsheet given in Appedix of Mitchell et al. Eliminated for the<br>analysis.

Summary of Data

	Cetin et al. (2016)	Idriss&Boulanger (2010)	Seed et.al. (1984)		Cetin et al. (2016)	Idriss&Boulanger (2010)	Seed et.al. (1984)
Liquefied?	Yes	marginal	Yes	D_{50}	0.320 ± 0.050		? ? ?
Data Class	A			% Fines	5.0 ± 2.0	3.0	sand, sand/sand &fines
Critical Depth Range	16.4 - 23.0	20.7	6.2 7.0 m	% PI			
Depth to GWT (ft)	9.8	9.8	3 m				
σ_v (psf)	2411.4 ± 142.9	2527.1	1.12 1.26 kg/cm²	N	16.2 ± 6.4	16.0	10.0 15.5
σ_v' (psf)	1797.2 ± 82.0	1858.8	0.80 0.86 kg/cm²	C_R	0.92	0.95	
a_{max}	0.220 ± 0.033	0.28	0.285	C_S	1.10	1.10	0.900
r_d	0.83 ± 0.018	0.92	0.94 0.93	C_B	1.00	1.00	
CSR	0.164 ± 0.024	0.229	0.244 0.252	C_E	0.92	0.92	
Equivalent Magnitude	6.93	6.93		C_N	1.09	1.10	1.11 1.08
MSF		1.16		$(N_1)_{60}$	16.4 ± 6.5	17.0	10.0 15.5
CSRN		0.194	0.23 0.238				

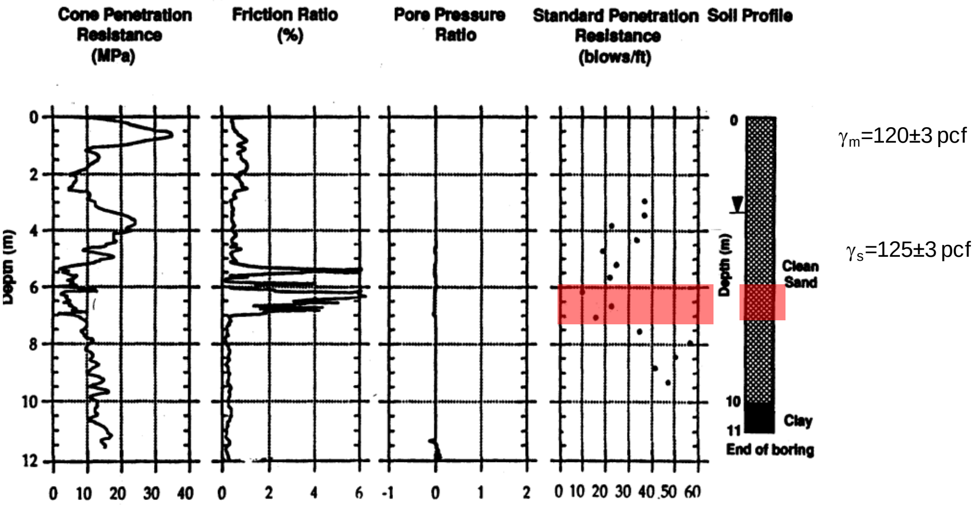

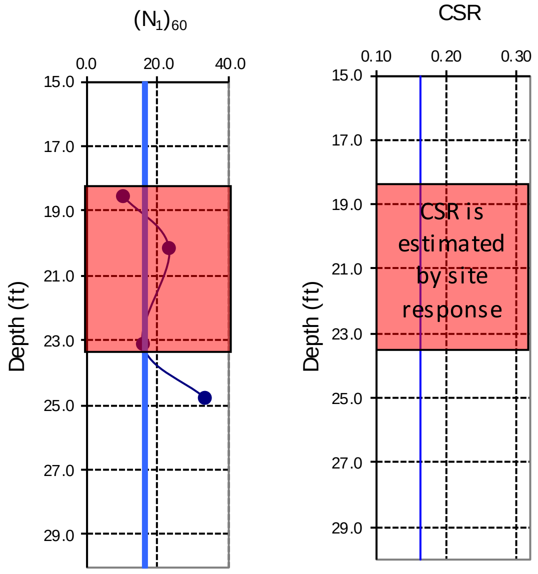

Depth (m)	Depth (ft)	CSR	N	C_N	C_R	$(N1)_{60}$
5.65	18.5		10	1.11	0.91	**10.2**
6.15	20.2		23	1.08	0.93	***23.2***
7.05	23.1		16	1.03	0.95	***15.8***
7.55	24.8		34	1.00	0.97	33.2
					**Mean:**	16.4
					**St. Dev.**	6.5

**Case number:**	134
**Earthquake:**	Loma Prieta
**Magnitude:**	6.93 (Mw) Pacific Earthquake Engineering Research Center: NGA Database
**Location:**	Port of Richmond (POR-2&3&4)
**References:**	Mitchell et al. (1994), "Insitu test results from four Loma Prieta Earthquake liquefaction sites: SPT, CPT, DMT and Shear Wave Velocity<br>Kayen et al. (1998), "Soil Liquefaction in the East Bay during the earthquake
**Nature of Failure:**	Four large and a dozen smaller sand boils vented fine sand and silty sand form the underlying fill. Seepage continued for as long as 24 hours.<br>Ground settlement and minor lateral spreading both aprroximately 2-8 cm.
**Comments:**	The liquefied area is a zone approximately 250 ft wide and 1000 ft long at the foot of Harbor Way Road.<br>The site represents the most distant point, approximately 85 km north of the rupture zone.<br>The liquefaction zone overlies deposits of soft bay mud, underlain by deeper deposits of stiffer, overconsolidated noncohesive and cohesive soils.<br>Up to 0.8 m depth the soil is characterized by oxidized, tan-brown crusted silty sand. 0.8-1.8 m oxidized tan-brown silty sand. 1.8-4.1 m oxidized tan-brown sandy clay to reduced sandy clay.4.1-7.8 m, is a hydraulic fill layer of olive-gray fine-silty sand containing shell fragments.<br>The thick deposits amplified the level of ground motion at the site.<br>At adjacent sites (POR-3 & 4) SPT, CPT and shear wave velocity tests were performed. POR-2 &3&4 are aprroximately 10-15m apart. Correlated?<br>PGA is estimated as 0.18 g by performing a site response analysis (SHAKE) using the input motion from Richmond City Hall (PGA=0.13g ~2km) by Kayen et al. Based on site response analyses results performed by using the recording scaled to the geometric mean of the two orthonormal components. PGA is estimated as 0.15.<br>The SPT blowcount ~11 is eliminated due to unrepresentative nature of it.<br>Depth to water table in Kayen et al. (1998) is reported as 2.5 m contrary to 3.5 m reported in Mitchell et al. (1994).

**Summary of Data**	Cetin et al. (2016)	Idriss&Boulanger (2010)	Seed et.al. (1984)		Cetin et al. (2016)	Idriss&Boulanger (2010)	Seed et.al. (1984)
Liquefied?	Yes	Yes	Yes	D_{50}	0.090 ± 0.050		? ?
Data Class	A			% Fines	50.0 ± 2.0	50.0	sand wt. fines sand
Critical Depth Range	19.0 - 13.1	19.4	5.25 7.25	% PI			
Depth to GWT (ft)	11.5	11.5	2.5				
σ_v (psf)	1652.0 ± 114.0	2025.9	0.95 1.35 kg/cm^2	N	3.2 ± 1.0	4.3	2 4
σ_v' (psf)	1366.2 ± 63.2	1524.6	0.67 0.85 kg/cm^3	C_R	0.89	0.95	↑
a_{max}	0.150 ± 0.023	0.18	0.160	C_S	1.10	1.10	0.900
r_d	0.78 ± 0.017	0.93	0.95 0.93	C_B	1.00	1.00	
CSR	0.090 ± 0.014	0.142	0.135 0.148	C_E	0.92	0.92	↓
Equivalent Magnitude	6.93	6.93		C_N	1.24	1.27	1.2 1.08
MSF		1.16	1.06	$(N_1)_{60}$	3.5 ± 1.1	5.1	2.2 3.9
CSRN		0.119	0.127 0.14				

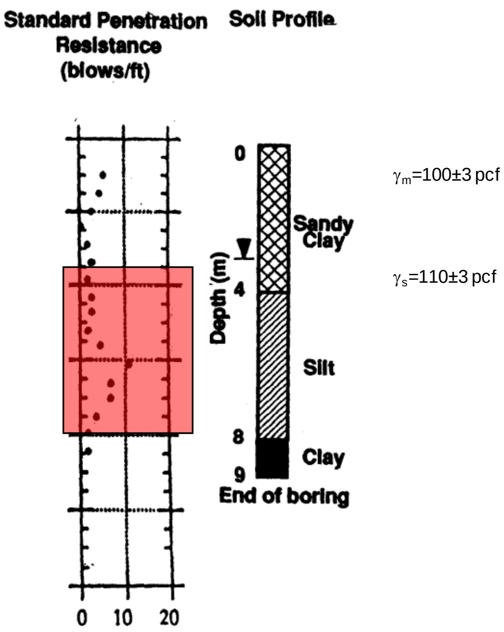

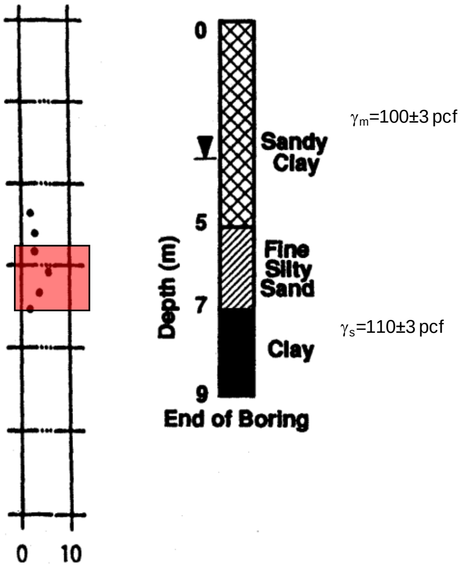

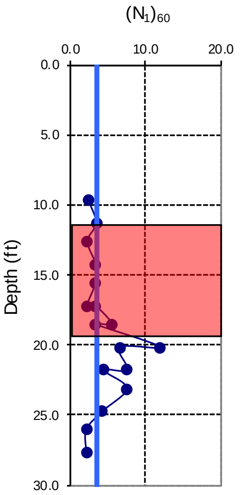

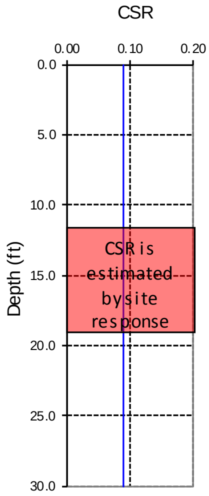

Depth (m)	Depth (ft)	CSR	N	C_N	C_R	$(N1)_{60}$		
2.95	9.7		2	1.48	0.80	2.4	POR-2	sandy clay
3.45	11.3		3	1.37	0.83	3.4	POR-2	sandy clay
3.85	12.6		2	1.33	0.84	2.3	POR-2	sandy clay
4.35	14.3		3	1.29	0.87	***3.4***	POR-2	
4.75	15.6		3	1.26	0.88	***3.3***	POR-2	
5.25	17.2		2	1.22	0.90	***2.2***	POR-2	
5.25	17.2		3	1.22	0.90	***3.3***	POR-3&4	
5.65	18.5		5	1.19	0.91	***5.5***	POR-2	
5.65	18.5		3	1.19	0.91	***3.3***	POR-3&4	
6.15	20.2		11	1.16	0.93	12.0	POR-2	
6.15	20.2		6	1.16	0.93	6.5	POR-3&4	
6.65	21.8		7	1.14	0.94	7.6	POR-2	
6.65	21.8		4	1.14	0.94	4.3	POR-3&4	
7.05	23.1		7	1.11	0.95	7.5	POR-2	
7.55	24.8		4	1.09	0.97	4.3	POR-2	
7.95	26.1		2	1.07	0.98	2.1	POR-2	
8.45	27.7		2	1.05	0.99	2.1	POR-2	
					**Mean:**	3.5		
					**St. Dev.**	1.1		

**Case number:**	135
**Earthquake:**	Loma Prieta
**Magnitude:**	6.93 (Mw) Pacific Earthquake Engineering Research Center: NGA Database
**Location:**	Sandholdt Road UC-B10
**References:**	Boulanger et al. (1996), "Liquefaction at Moss Landing During Loma Prieta Earthquake"
**Nature of Failure:**	Extensive ground deformations were observed along Sandholdt Road, including the portion alongside the MBARI facilities. Cracks parallel to the front line. Away from Sandholdt Road, surface evidence of liquefaction appeared to occur in localized areas.
**Comments:**	Located on Monterey Bay about midway between the cities of Santa Cruz and Monterey, CA.<br>The MBARI facilities include three main buildings and a pier located along Sandholt Road on the Moss Landing spit. Historical shoreline suggest that the shoreline in the vicinity of the site has fluctuated significantly. Shallower soil strata may have been deposited recently.<br>The area is underlain by Holocene deposits consisting of relatively thick sands offshore and estuarine and fluvial deposits onshore, up to about 60-m deep.<br>The Moss Landing spit is underlain by littoral soils comprising eolian, fluvial, estuarine, and beach deposits of gravel, sand, silt, and clay deposited in a shoreline environment within the zone of tidal fluctuation. Pleistocene and miocene deposits extend to a depth of ~2000 m<br>The site is underlain by an 8-12 m thick layer of poorly graded sand with interlayers of gravelly sand and sandy gravel in its lower portion and occasional thin layers of clayey silt throughout.<br>PGA is recorded in a four-story building 8km from rupture plane as 0.39 g which is underlain by alluvium. Also San Juan Bautista seismogram, ~20 km from the rupture plane recorded 0.15 g (stiff alluvium). The Moss Landing area is located ~21 km from the rupture plane. Due to deep alluvium which is soft near the surface, PGA at the site is predicted in the range of 0.2-0.3 g.<br>PGA is chosen for the analysis as 0.26 g almost identical with the value chosen by the authors of the source document (0.25 g)<br>~30 ft away from 30 degree 15' slope.<br>SPT performed after the earthquake.

Summary of Data

	Cetin et al. (2016)	Idriss&Boulanger (2010)	Seed et.al. (1984)		Cetin et al. (2016)	Idriss&Boulanger (2010)	Seed et.al. (1984)
Liquefied?	Yes	Yes		D_{50}	0.800 ± 0.050		
Data Class	A			% Fines	2.0 ± 2.0	2.0	
Critical Depth Range	8.0 - 13.0	9.8		% PI			
Depth to GWT (ft)	5.6	5.9					
σ_v (psf)	1203.9 ± 102.5	1148.7		N	9.2 ± 3.8	9.5	
σ_v' (psf)	898.8 ± 55.2	898.1		C_R	0.81	0.85	
a_{max} (g)	0.260 ± 0.039	0.28		C_S	1.00	1.00	
r_d	0.99 ± 0.008	0.97		C_B	1.00	1.00	
CSR	0.224 ± 0.035	0.226		C_E	1.25	1.25	
Equivalent Magnitude	6.93	6.93		C_N	1.53	1.52	
MSF		1.16		$(N_1)_{60}$	14.3 ± 5.9	15.3	
CSRN		0.177					

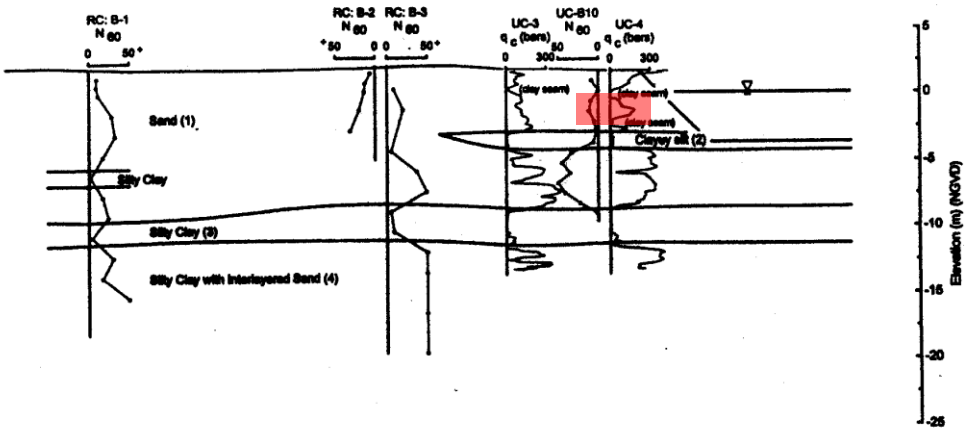

γ_m=110±3 pcf

γ_s=120±3 pcf

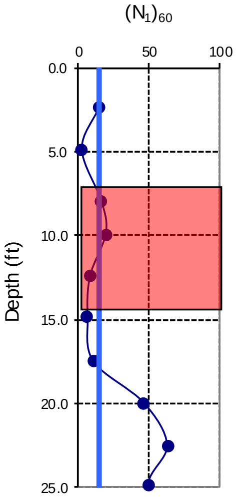

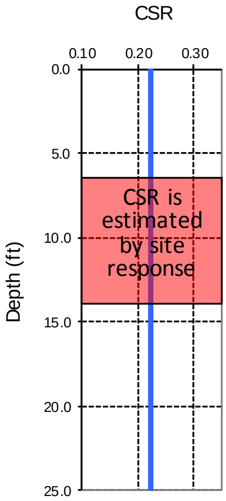

Depth (m)	Depth (ft)	CSR	N	C_N	C_R	$(N1)_{60}$
0.7	2.4		9	2.00	0.63	*14.1*
1.5	4.9		1	1.99	0.70	*1.7*
2.4	8.0		10	1.67	0.77	***16.1***
3.0	9.9		12	1.56	0.80	***18.9***
3.8	12.4		5	1.45	0.84	***7.6***
4.5	14.9		4	1.36	0.87	*5.9*
5.3	17.5		7	1.28	0.90	*10.1*
6.1	19.9		33	1.21	0.93	*46.3*
6.9	22.5		46	1.15	0.95	*63.0*
7.6	24.8		37	1.11	0.97	*49.6*
8.4	27.4		50	1.06	0.99	*65.6*
9.1	29.8		42	1.03	1.00	*53.9*
9.9	32.4		24	0.99	1.00	*29.7*
10.6	34.8		3	0.96	1.00	*3.6*
					**Mean:**	14.2
					**St. Dev.**	5.9

**Case number:**	136
**Earthquake:**	Loma Prieta
**Magnitude:**	6.93 (Mw) Pacific Earthquake Engineering Research Center: NGA Database
**Location:**	San Francisco Oakland Bay Bridge (SFOBB-1&2)
**References:**	Mitchell et al. (1994), "Insitu test results from four Loma Prieta Earthquake liquefaction sites: SPT, CPT, DMT and Shear Wave Velocity<br>Kayen et al. (1998), "Soil Liquefaction in the East Bay during the earthquake
**Nature of Failure:**	Significant settlements (max ~ 40 cm.) occurred over most of the mole.<br>In some places differential settlements produced hummocky surface with permanent waves as large as 15 cm in amplitude.<br>Significant lateral spreading along most of the fill, causing longitudinal fissures.<br>Many of the fissures exuded fine to silty sand. Numerous sand boils erupted along the median strip of the roadway. Fissure width openings ~7-8 cm and exhibited maximum differential vertical offset of 3-5 cm.
**Comments:**	The San Francisco- Oakland Bay Bridge mole (peninsula-approach fill) is located at immediate south of Emeryville.<br>SFOBB-1 was drilled at the easternmost end of the Toll Plaza Parking lot.<br>No piezocone pore-pressure generation during cone penetration until bay mud was reached at 15 m depth.<br>0-2.8 m depth, moderately dense surface crust; 2.8-4.5 m interbedded sequence of cohesionless silty sand and cohesive clay. Between 4.5 to 7.5 m lies cohesionless material with low q_c values ~5MPa. Drill rod and hammer sank under their own weight at 6.6 m depth.<br>PGA is recorded as 0.29 & 0.27 at the Oakland Outer Harbor seismogram which is located on a 155 m deep soil site.<br>PGA is estimated as 0.25 g based on site response analyses results.<br>The average of recorded and site response based estimated PGA values was adopted.<br>Depth to water table in Kayen et al. (1998) is reported as 2.0 m contrary to 3.0 m reported in Mitchell et al. (1994).<br>Measurements performed after the earthquake.<br>The measurements could be correlated with SFOBB-2.

Summary of Data

	Cetin et al. (2016)	Idriss&Boulanger (2010)	Seed et.al. (1984)		Cetin et al. (2016)	Idriss&Boulanger (2010)	Seed et.al. (1984)
Liquefied?	Yes	Yes	Yes	D_{50}	0.280 ± 0.050		?
Data Class	A			% Fines	8.0 ± 2.0	8.0	sand sand
Critical Depth Range	18.0 - 23.0	20.7	6.7m 6.1m	% PI			
Depth to GWT (ft)	9.8	9.8	2.0 m				
σ_v (psf)	2513.9 ± 111.4	2464.5	1.21 1.10 kg/cm^2	N	7.5 ± 2.0	7.5	1 5
σ_v' (psf)	1848.6 ± 69.5	1796.1	0.74 0.69 kg/cm^2	C_R	0.93	0.95	↑
a_{max}	0.270 ± 0.041	0.27	0.285	C_S	1.20	1.20	0.900
r_d	0.79 ± 0.013	0.92	0.93 0.94	C_B	1.00	1.00	
CSR	0.190 ± 0.029	0.222	0.283 0.278	C_E	0.92	0.92	↓
Equivalent Magnitude	6.93	6.93		C_N	1.07	1.10	1.15 1.18
MSF		1.16		$(N_1)_{60}$	8.2 ± 2.2	8.6	1 5.3
CSRN		0.189	0.267 0.262				

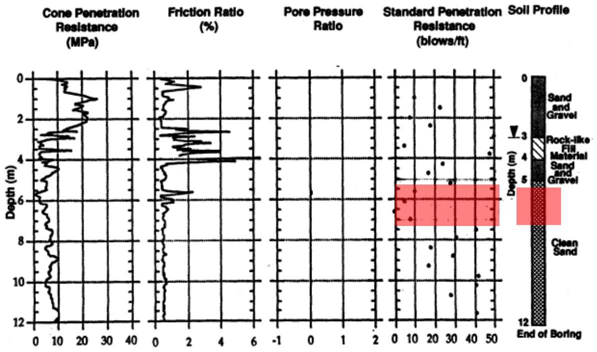

γ_m=120±3 pcf

γ_s=130±3 pcf

γ_s=115±3 pcf

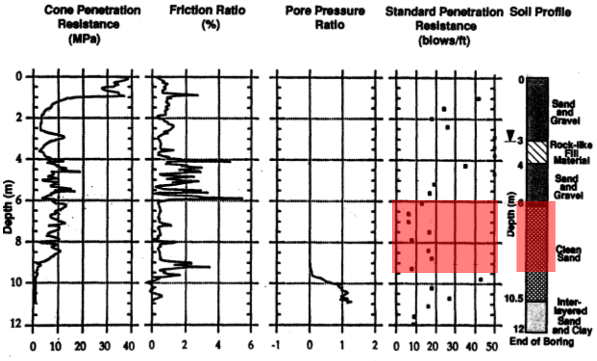

γ_m=120±3 pcf

γ_s=130±3 pcf

γ_s=125±3 pcf

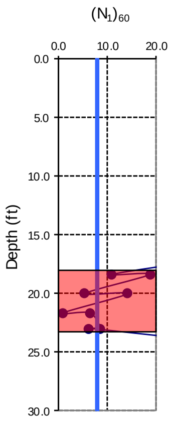

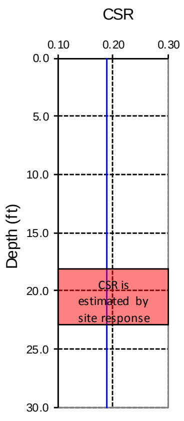

Depth (m)	Depth (ft)	CSR	N	C_N	C_R	$(N1)_{60}$		
5.2	17.1		28	1.14	0.90	31.5	SFOBB-1	
5.6	18.4		10	1.11	0.91	***11.1***	SFOBB-1	
5.6	18.4		17	1.11	0.91	18.9	SFOBB-2	Gravel
6.1	20.0		5	1.08	0.93	5.5	SFOBB-1	Fines?
6.1	20.0		13	1.08	0.93	14.3	SFOBB-2	Gravel?
6.6	21.7		1	1.05	0.94	1.1	SFOBB-1	Eliminate
6.6	21.7		6	1.05	0.94	***6.5***	SFOBB-2	
7	23.0		8	1.03	0.95	***8.6***	SFOBB-1	
7	23.0		6	1.03	0.95	***6.5***	SFOBB-2	
7.5	24.6		41	1.00	0.97	43.7	SFOBB-1	
					**Mean:**	8.2		
					**St. Dev.**	2.2		

**Case number:**	137
**Earthquake:**	Loma Prieta
**Magnitude:**	6.93 (Mw) Pacific Earthquake Engineering Research Center: NGA Database
**Location:**	Moss Landing State Beach UC-B1
**References:**	Boulanger et al. (1996), "Liquefaction at Moass Landing During Loma
**Nature of Failure:**	Liquefaction-induced lateral spreading caused extensive damage to the access road. The tide gate was destroyed due to liquefaction flow slide. Road surface slumping about 1.3 m. Lateral spreading were roughly 0.3-0.6 m horizontal with vertical offset up to 0.3 m. Sand boils and ground cracks on the north side of the access road.
**Comments:**	Located on Monterey Bay about midway between the cities of Santa Cruz and Monterey, CA.<br>The area is underlain by Holocene deposits consisting of relatively thick sands offshore and estuarine and fluvial deposits onshore, up to about 60-m deep.<br>The Moss Landing spit is underlain by littoral soils comprising eolian, fluvial, estuarine, and beach deposits of gravel, sand, silt, and clay deposited in a shoreline environment within the zone of tidal fluctuation. Pleistocene and miocene deposits extend to a depth of ~2000 m.<br>The upper 8-10 m of soils encountered were poorly graded, clean sands to gravelly sands having no more than 3 % fines. Underlying the sands was a complex sequence of interlayered clay, sand, and gravelly sand.<br>PGA is recorded in a four-story building 8km from rupture plane as 0.39 g which is underlain by alluvium. Also San Juan Bautista seismogram, ~20 km from the rupture plane recorded 0.15 g (stiff alluvium). The Moss Landing area is located ~21 km from the rupture plane. Due to deep alluvium which is soft near the surface, PGA at the site is predicted between 0.2-0.3g<br>PGA adopted for the analysis based on site response analyses results (~0.29 g) is slightly higher than the value suggested by the authors of the source document.<br>SPT performed after the earthquake.

**Summary of Data**	Cetin et al. (2016)	Idriss&Boulanger (2010)	Seed et.al. (1984)		Cetin et al. (2016)	Idriss&Boulanger (2010)	Seed et.al. (1984)
Liquefied?	Yes	Yes		D_{50}	0.260 ± 0.050		
Data Class	A			% Fines	1.7 ± 0.6	1.0	
Critical Depth Range	5.9 - 12.0	11.2		% PI			
Depth to GWT (ft)	5.9	5.9					
σ_v (psf)	1030.5 ± 128.6	1274.0		N	5.1 ± 0.9	6.3	
σ_v' (psf)	840.4 ± 68.1	960.7		C_R	0.79	0.85	
a_{max} (g)	0.290 ± 0.044	0.28		C_S	1.00	1.00	
r_d	0.95 ± 0.010	0.97		C_B	1.00	1.00	
CSR	0.240 ± 0.035	0.234		C_E	1.25	1.25	
Equivalent Magnitude	6.93	6.93		C_N	1.59	1.53	
MSF		1.16		$(N_1)_{60}$	7.9 ± 1.4	10.3	
CSRN		0.187					

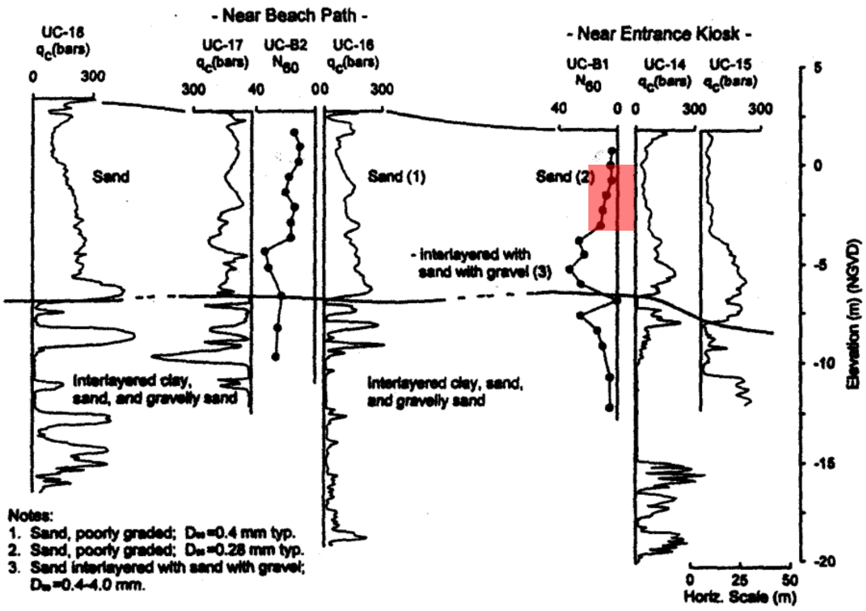

γ_m=110±3 pcf

γ_s=125±3 pcf

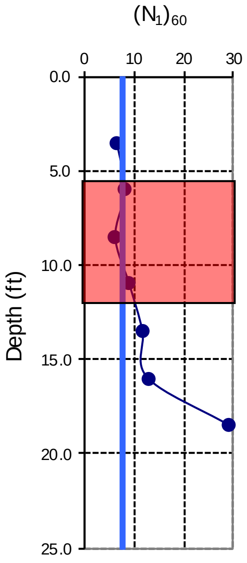

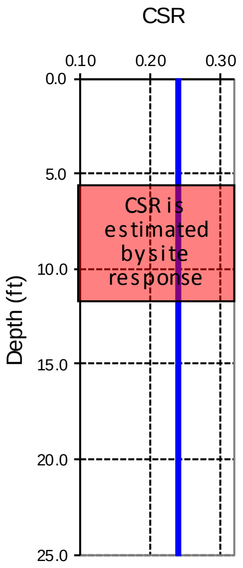

Depth (m)	Depth (ft)	CSR	N	C_N	C_R	$(N1)_{60}$	FC
1.1	3.5		4	2.00	0.66	6.6	0.0
1.8	6.0		5	1.80	0.73	***8.2***	***2.0***
2.6	8.5		4	1.61	0.78	***6.3***	***2.0***
3.4	11.0		6	1.48	0.82	***9.1***	***1.0***
4.1	13.5		8	1.37	0.86	11.7	1.0
4.9	16.0		9	1.29	0.89	12.8	1.0
5.6	18.5		21	1.21	0.91	29.1	1.0
					**Mean:**	7.9	1.7
					**St. Dev.**	1.4	0.6

**Case number:**	138
**Earthquake:**	Loma Prieta
**Magnitude:**	6.93 (Mw) Pacific Earthquake Engineering Research Center: NGA Database
**Location:**	Moss Landing State Beach UC-B2
**References:**	Boulanger et al. (1996), "Liquefaction at Moss Landing During Loma Prieta Earthquake"
**Nature of Failure:**	Liquefaction-induced lateral spreading caused extensive damage to the access road. The tide gate was destroyed due to liquefaction flow slide. Road surface slumping about 1.3 m. Lateral spreading were roughly 0.3-0.6 m horizontal with vertical offset up to 0.3 m. Sand boils and ground cracks on the north side of the access road.
**Comments:**	Located on Monterey Bay about midway between the cities of Santa Cruz and Monterey, CA.<br>The area is underlain by Holocene deposits consisting of relatively thick sands offshore and estuarine and fluvial deposits onshore, up to about 60-m deep.<br>The Moss Landing spit is underlain by littoral soils comprising eolian, fluvial, estuarine, and beach deposits of gravel, sand, silt, and clay deposited in a shoreline environment within the zone of tidal fluctuation. Pleistocene and miocene deposits extend to a depth of ~2000 m.<br>The upper 8-10 m of soils encountered were poorly graded, clean sands to gravelly sands having no more than 3 % fines. Underlying the sands was a complex sequence of interlayered clay, sand, and gravelly sand.<br>PGA is recorded in a four-story building 8km from rupture plane as 0.39 g which is underlain by alluvium. Also San Juan Bautista seismogram, ~20 km from the rupture plane recorded 0.15 g (stiff alluvium). The Moss Landing area is located ~21 km from the rupture plane. Due to deep alluvium which is soft near the surface, PGA at the site is predicted between 0.2-0.3g<br>Based on site response analyses results, a PGA value of 0.24 g is adopted which is slightly lower than the value suggested by the authors of the source document (0.25 g).<br>SPT performed after the earthquake.

**Summary of Data**	Cetin et al. (2016)	Idriss&Boulanger (2010)	Seed et.al. (1984)		Cetin et al. (2016)	Idriss&Boulanger (2010)	Seed et.al. (1984)
Liquefied?	Yes	Yes		D_{50}	0.400 ± 0.050		
Data Class	A			% Fines	1.0 ± 2.0	1.0	
Critical Depth Range	9.0 - 22.0	16.1		% PI			
Depth to GWT (ft)	9.0	8.5					
σ_v (psf)	1892.5 ± 272.9	1879.7		N	13.3 ± 1.8	12.8	
σ_v' (psf)	1486.9 ± 140.7	1399.3		C_R	0.88	0.95	
a_{max} (g)	0.240 ± 0.036	0.28		C_S	1.00	1.00	
r_d	0.99 ± 0.013	0.95		C_B	1.00	1.00	
CSR	0.210 ± 0.031	0.229		C_E	1.25	1.25	
Equivalent Magnitude	6.93	6.93		C_N	1.19	1.20	
MSF		1.16		$(N_1)_{60}$	17.4 ± 2.4	18.4	
CSRN		0.188					

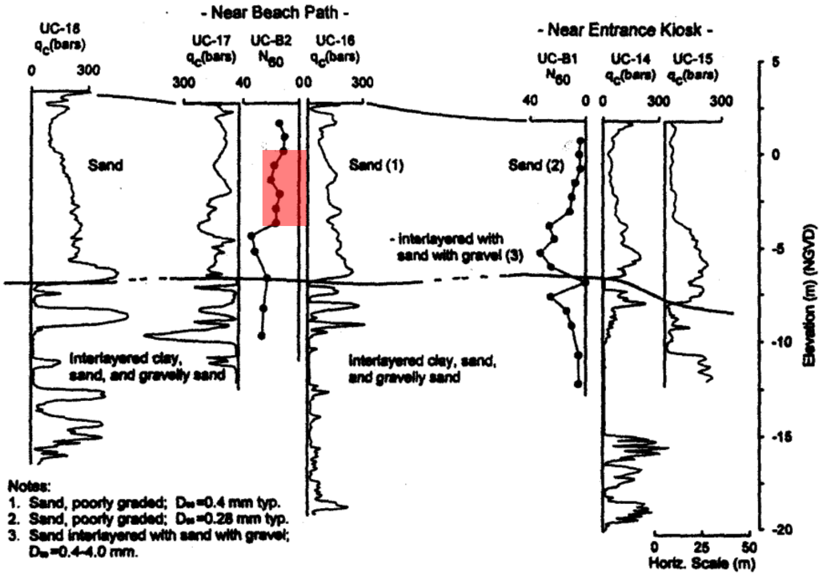

γ_m=120±3 pcf

γ_s=125±3 pcf

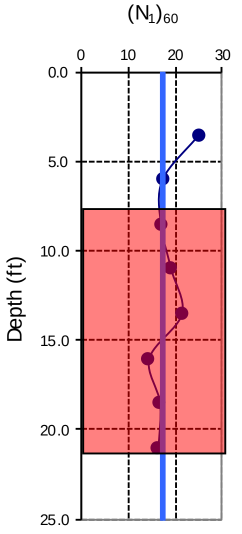

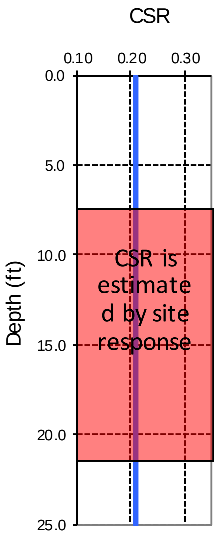

Depth (m)	Depth (ft)	CSR	N	C_N	C_R	$(N1)_{60}$
1.1	3.5		15	2.00	0.66	24.9
1.8	6.0		11	1.71	0.73	17.2
2.6	8.5		12	1.44	0.78	***16.8***
3.4	11.0		14	1.33	0.82	***19.0***
4.1	13.5		16	1.25	0.86	***21.3***
4.9	16.0		11	1.18	0.89	***14.4***
5.6	18.5		13	1.12	0.91	***16.7***
6.4	21.0		13	1.08	0.94	***16.4***
					**Mean:**	17.4
					**St. Dev.**	2.4

**Case number:**	139
**Earthquake:**	Loma Prieta
**Magnitude:**	6.93 (Mw) Pacific Earthquake Engineering Research Center: NGA Database
**Location:**	Treasure Island Naval Station
**References:**	De Alba et al., Youd and Shakal (1994), "Deep Instrumentation Array at the Treasure Island Naval Station"<br>Rollins and Mchood (1994), "Ground Response on Treasure Island"
**Nature of Failure:**	No surface evidence of liquefaction; however significant change in frequency content of the ground motion after 15 s. compared to the recorded motion at 322 ft.
**Comments:**	Treasure Island is a 400-acre manmade island immediately northwest of the rock outcrop on Yerba Buena Island in San Francisco Bay. The original surface soils included a shallow-water sandbar or spit extending northwestward from Yerba Buena Island, and soft silty clay known as bay mud surrounding the sandbar to the north and east. Approximately 65% of the island was built on the sandbar and the rest on the bay mud. Treasure Island was constructed by hydraulic and clamshell dredging. A perimeter rock dike was built in two to four stages on a bed of coarse sand placed over bay mud. The structure is essentially an upstream-constructed hydraulic fill.<br>The soils on Treasure Island may be grouped into four broad categories: fill, native shoal sand, recent bay sedimentary deposits, and older bay sedimentary deposits. Both the fill and native shoal sand consists predominantly of sand and varying amounts of clay, silt and gravel; however, the fill is somewhat looser.<br>The depth and thickness of soil layers vary significantly around the island. The thickness of the fill and native shoal sand ranges from 10.7 m at the south end to 15.2 m in the north. The depth of recent bay sedimentary deposits ranges from 10.7 m in the south. Bedrock elevation has been confirmed at a depth of 85 m.<br>PGA is recorded on Yerba Buena Island as 0.06 and 0.03 g (rock outcrop) and 0.16 and 0.11 g onTreasure Island.<br>A PGA value of 0.18 g is adopted based on site response analyses results.<br>Depth to water table is reported as 1.5 m.

**Summary of Data**	Cetin et al. (2016)	Idriss&Boulanger (2010)	Seed et.al. (1984)		Cetin et al. (2016)	Idriss&Boulanger (2010)	Seed et.al. (1984)
Liquefied?	Yes	Yes		D_{50}	0.170 ± 0.050		
Data Class	A			% Fines	20.0 ± 2.0	20.0	
Critical Depth Range	29.5 - 4.9	21.3		% PI			
Depth to GWT (ft)	4.9	4.9					
σ_v (psf)	1907.0 ± 473.3	2422.7		N	5.1 ± 3.0	4.3	
σ_v' (psf)	1139.3 ± 219.5	1399.3		C_R	0.90	0.95	
a_{max}	0.180 ± 0.027	0.16		C_S	1.10	1.10	
r_d	0.88 ± 0.012	0.92		C_B	1.00	1.00	
CSR	0.160 ± 0.028	0.165		C_E	1.13	1.13	
Equivalent Magnitude	6.93	6.93		C_N	1.36	1.24	
MSF		1.16		$(N_1)_{60}$	7.7 ± 4.6	6.4	
CSRN		0.137					

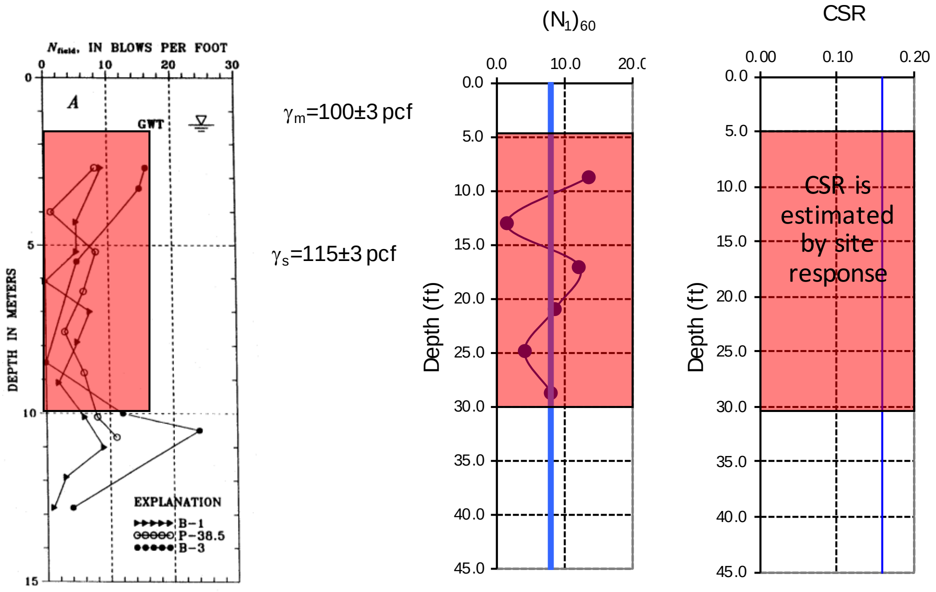

P-38.5

Depth (m)	Depth (ft)	CSR	N	C_N	C_R	$(N1)_{60}$
2.7	8.9		8	1.74	0.79	***13.6***
4	13.1		1	1.51	0.85	***1.6***
5.2	17.1		8	1.37	0.90	***12.2***
6.4	21.0		6	1.26	0.94	***8.8***
7.6	24.9		3	1.17	0.97	***4.2***
8.8	28.9		6	1.10	1.00	***8.2***
					**Mean:**	8.1
					**St. Dev.**	4.6

B-1

Depth (m)	Depth (ft)	CSR	N	C_N	C_R	$(N1)_{60}$
2.7	8.9		9	1.74	0.79	***15.3***
4.2	13.8		5	1.49	0.86	***8.0***
5.2	17.1		5	1.37	0.90	***7.7***
6	19.7		0	1.29	0.92	***0.0***
7	23.0		7	1.21	0.95	***10.1***
7.9	25.9		5	1.15	0.98	***7.0***
9.1	29.9		2	1.08	1.00	***2.7***
					**Mean:**	7.3
					**St. Dev.**	5.0

**Case number:**	140
**Earthquake:**	Loma Prieta
**Magnitude:**	6.93 (Mw) Pacific Earthquake Engineering Research Center: NGA Database
**Location:**	Woodward Marine UC-B4
**References:**	Boulanger et al. (1996), "Liquefaction at Moss Landing During Loma Prieta Earthquake"
**Nature of Failure:**	Liquefaction-induced ground spreading was evident across much of the site. Along the shoreline, jetty piles were displaced horizontally, and the boat ramp and adjacent asphalt pavement experienced cracking and settlement. Several cracks up to 5 cm wide. The oil and gas tanks settled and tilted 1-6°. Gray coarse to medium grained sand was vented through some of the cracks. Sand dikes were traced from the ground surface to a depth of about 0.8 m through a gravelly sand and gray, medium-grained sand to a gray silty sand.
**Comments:**	Located on Salomon Way off Sandholdt Road near the northern end of the Moss Landing spit and along the South Harbor.<br>The Woodward Marine facilities include a boat launch ramp, fuel service dock and oil and gas storage tanks.<br>The Moss Landing spit is underlain by littoral soils comprising eolian, fluvial, estuarine, and beach deposits of gravel, sand, silt, and clay deposited in a shoreline environment within the zone of tidal fluctuation. Pleistocene and miocene deposits extend to a depth of ~2000 m.<br>A ~1m thick layer of sandy fill blankets the site. By the shoreline, the fill is underlain by a roughly 2.2 m thick layer of sand and silty sand. By the fuel tanks, the fill is underlain by a 1-2 m thick layer of soft silty sand, sandy silt and silt. This soft layer of silty sand to silt is underlain by a thin zone of sand which appears similar to the sand encountered at similar depths near the shoreline. These materials are underlain by a ~ 2m thick layer of denser sand. Beneath this layer, there is a layer of high plasticity clay with interlayers of dense silty sand and sand.<br>PGA is recorded in a four-story building 8 km from rupture plane as 0.39 g which is underlain by alluvium. Also Sab Juan Bautista seismogram, ~20 km from the rupture plane recorded 0.15 g (stiff alluvium). The Moss Landing area is located ~21 km from the rupture plane. Due to deep alluvium which is soft near the surface, PGA at the site is predicted between 0.2-0.3 g.<br>PGA is chosen for the analysis as 0.25 g same as the value chosen by the authors of the source document. Also site response analyses results supported this conclusion.<br>SPT performed after the earthquake.

**Summary of Data**	Cetin et al. (2016)	Idriss&Boulanger (2010)	Seed et.al. (1984)		Cetin et al. (2016)	Idriss&Boulanger (2010)	Seed et.al. (1984)
Liquefied?	Yes	Yes		D_{50}	0.100 ± 0.050		
Data Class	A			% Fines	35.0 ± 2.0	35.0	
Critical Depth Range	3.3 - 8.2	5.9		% PI			
Depth to GWT (ft)	3.3	3.3					
σ_v (psf)	656.2 ± 99.2	668.3		N	5.8 ± 0.2	6.7	
σ_v' (psf)	502.6 ± 51.3	522.1		C_R	0.72	0.80	
a_{max} (g)	0.250 ± 0.038	0.28		C_S	1.00	1.00	
r_d	0.99 ± 0.005	0.99		C_B	1.00	1.00	
CSR	0.200 ± 0.034	0.233		C_E	1.00	1.00	
Equivalent Magnitude	6.93	6.93		C_N	2.00	1.70	
MSF		1.16		$(N_1)_{60}$	8.3 ± 0.3	9.1	
CSRN		0.183					

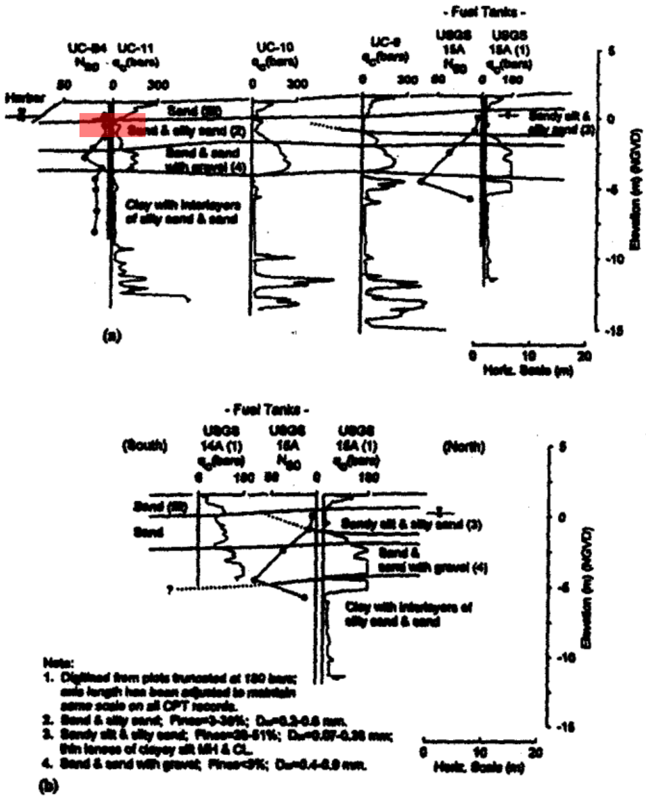

γ_m=110±3 pcf

γ_s=120±3 pcf

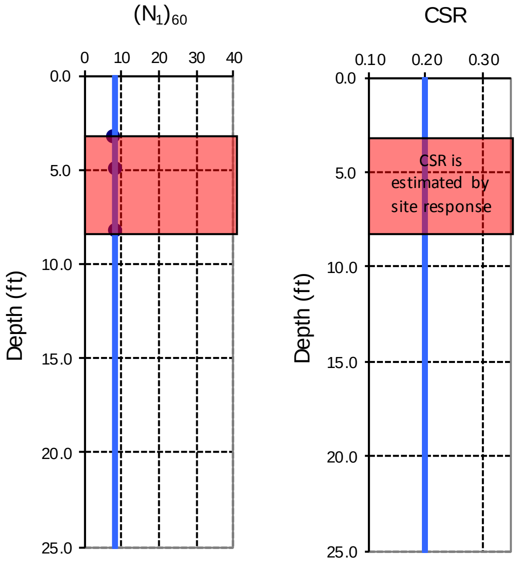

Depth (m)	Depth (ft)	CSR	N	C_N	C_R	$(N1)_{60}$
1	3.3		6	2.00	0.66	***7.9***
1.5	4.9		6	2.00	0.70	***8.4***
2.5	8.2		6	1.81	0.77	***8.4***
					**Mean:**	8.3
					**St. Dev.**	0.3

Case number: 141

Earthquake: Loma Prieta

Magnitude: 6.93 (Mw) Pacific Earthquake Engineering Research Center: NGA Database

Location: General Fish

References: Boulanger et al. (1995), Boulanger et al. (1997)

Nature of Failure:

"The Site of General Fish Company experienced minor lateral movements. Ground cracks and some cracks in the buildings (up to 8 cm) were observed. A significant ground crack was observed at the location of EB-5 borehole. Lateral movement of the site was tottally estimated as 5-7.5 cm." (Barminski 1993)

Comments:

It is stated that the site is somewhere near to existing metal building.
In original document it is stated that "the General Fish Company building were constructed of wood but the details are not known". Boulanger et.al. (1995)
The site consists of 8-12 m of poorly graded sand (FC=5%; 1-8%, D_{50}=0.20-2.1 mm) with some interlayers.
For SI-2 inclinometer D_{50}=0.40-0.70 mm. Sand layer is underlained by clay layer of low plasticity (ML and CL).
The magnitude of the Loma Prieta is given by USGS equal to 6.9. The acceleration value is recommended by Boulanger (1995) 0.20-0.30 g. In Cetin 2014 it is taken as 0.25 g. However in Boulanger 2010 study it is taken as 0.28 g.
In Boulanger et.al (1997) it is stated that "a peak horizantal acceleration of about 0.25 g is a reasonable estimate for evaluation the effects of soil behavior during the 1989 Loma Prieta earthquake at Moss Landing .In Cetin 2014 a_{max} is taken as 0.25 g.
"The SPT were performed by Rutherford and Chekene (1988,1993) by Taber Consultans using a Concore rig. A fish tail bit was used for drilling. SPT testes were performed by using a safety hammer, a cathead and rope system with 2 wraps of the rope around the cathead and a 5-cm-outside diameter split spoon sampler with room for liners. A few samples were obtained using a California split spoon sampler (6.3-cm outside-diameter). Energy measurements for this rig and the procedures while drilling at shallow depth in soft clay on another profect indicated an average energy ratio of 65%.Given the differences in energy ratios that may arise from different operators and soil conditions it seems reasonable to conclude that the blow counts reported by R&C (1988,1993) can be essentially taken as N_{60} values." Boulanger et.al. (1995).
In Boulanger et.al (1995) borehole x-axis was named as $N_{1,60}$, In Boulanger et.al (1997) x-axis named as N_{60}. In this study these values are taken as N_{60} values. GWT is taken as 1.7 m.
There exist ambiguity in Boulanger et.al (1995)
1. FC data is missing. The data is given in the output section on a graph however since the ground level information is missing it is unclear in which depth these fines content can be assigned.
In the text in Boulanger et.al (1995) it is stated that "The site consists of 8-12 m of poorly graded sand (FC=5%; 1-8%, D50=0.20-2.1 mm) with some interlayers. In this study FC=5% is taken for all depths.
Sea depth is 3 m. B1 is located 28 m away from sea, B2 is located 69 m away from sea and B3 is located 71 m away from sea. Thus, the site is a free field.

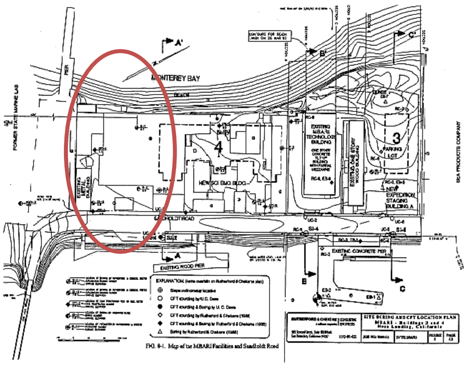

Summary of Data

	Cetin et al. (2016)	Idriss&Boulanger (2010)	Seed et.al. (1984)		Cetin et al. (2016)	Idriss&Boulanger (2010)	Seed et.al. (1984)
Liquefied?	No	No		D_{50}	0.550 ± 0.050	-	
Data Class	B	-		% Fines	5.0 ± 2.0	5.0	
Critical Depth Range	5.0 - 8.3	8.2		% PI	-	-	
Depth to GWT (ft)	5.5	4.6					
σ_v (psf)	749.0 ± 70.8	939.8		N	11.5 ± 2.3	16.9	
σ_v' (psf)	676.2 ± 38.6	731.0		C_R	0.74	0.80	
a_{max} (g)	0.250 ± 0.075	0.280		C_S	1.00	1.00	
r_d	0.99 ± 0.007	0.980		C_B	1.00	1.00	
CSR	0.179 ± 0.054	0.232		C_E	1.00	1.00	
Equivalent Magnitude	6.93	6.93		C_N	1.77	1.58	
MSF		1.16		$(N_1)_{60}$	15.1 ± 3.0	21.4	
CSRN		0.18					

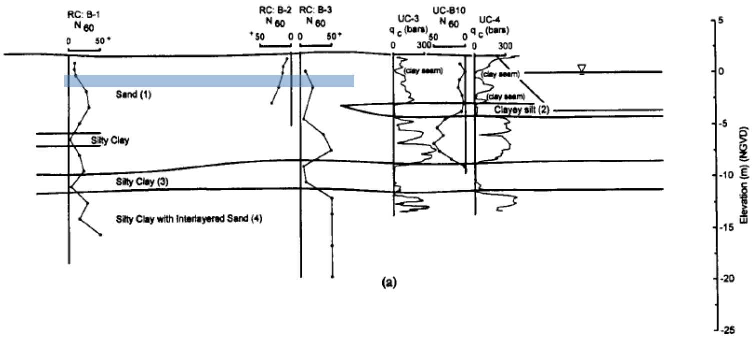

γ_m=110±3 pcf

γ_s=125±3 pcf

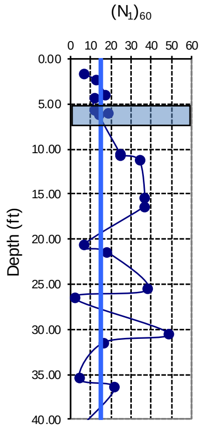

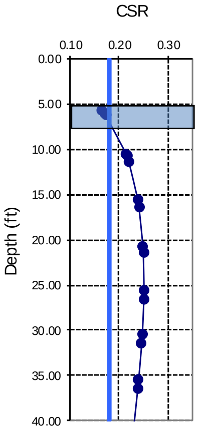

Depth (m)	Depth (ft)	CSR	N	C_N	C_R	$(N1)_{60}$	FC	Borehole	Soil Type
0.5	1.64	NA	6	2.00	0.60	7.2	5.0	**B2**	Sand(1)
0.7	2.33	NA	10	2.00	0.63	12.5	5.0	**B1**	Sand(1)
1.2	4.04	NA	13	2.00	0.68	***17.7***	***5.0***	***B2***	Sand(1)
1.3	4.33	NA	9	2.00	0.69	***12.4***	***5.0***	***B1***	Sand(1)
1.7	5.71	0.165	9	1.85	0.72	***12.0***	***5.0***	***B3***	Sand(1)
1.8	6.04	0.170	14	1.82	0.73	***18.6***	***5.0***	***B2***	Sand(1)
1.9	6.20	0.173	11	1.81	0.73	***14.6***	***5.0***	***B1***	Sand(1)
3.2	10.50	0.215	20	1.52	0.81	24.7	5.0	**B2**	Sand(1)
3.3	10.70	0.216	20	1.51	0.82	24.6	5.0	**B3**	Sand(1)
3.4	11.29	0.220	28	1.48	0.83	34.2	5.0	**B1**	Sand(1)
4.7	15.49	0.238	32	1.31	0.88	37.0	5.0	**B2**	Sand(1)
5.0	16.37	0.241	32	1.28	0.89	36.6	5.0	**B1**	Sand(1)
6.3	20.70	0.249	6	1.17	0.93	6.5	5.0	**B3**	Sand(1)
6.5	21.42	0.250	17	1.15	0.94	18.4	5.0	**B1**	Sand(1)
7.8	25.59	0.251	37	1.07	0.97	38.4	5.0	**B3**	Sand(1)
8.1	26.51	0.251	2	1.05	0.98	2.1	5.0	**B1**	Silty Clay
9.3	30.48	0.248	49	0.99	1.00	48.4	5.0	**B3**	Sand(1)
9.6	31.50	0.247	17	0.97	1.00	16.6	5.0	**B1**	Sand(1)
10.8	35.50	0.240	5	0.92	1.00	4.6	5.0	**B3**	Silty Clay (3)
11.1	36.48	0.239	24	0.91	1.00	21.9	5.0	**B1**	Sand(1)
12.3	40.35	0.231	9	0.87	1.00	7.8	5.0	**B3**	Silty Clay (3)
12.6	41.47	0.229	4	0.86	1.00	3.4	5.0	**B1**	Silty Clay (3)
13.9	45.60	0.221	50	0.82	1.00	41.2	5.0	**B3**	Silty Clay with interlayered Sand(4)
14.2	46.59	0.219	30	0.82	1.00	24.5	5.0	**B1**	Silty Clay with interlayered Sand(4)
15.8	51.67	0.212	18	0.78	1.00	14.0	5.0	**B1**	Silty Clay with interlayered Sand(4)
17.3	56.63	0.207	50	0.75	1.00	37.3	5.0	**B1**	Silty Clay with interlayered Sand(4)
					**Mean:**	15.1	5.0		
					**St. Dev.**	3.0	2.0		

**Case number:**	142
**Earthquake:**	Loma Prieta
**Magnitude:**	6.93 (Mw) Pacific Earthquake Engineering Research Center: NGA Database
**Location:**	Marine Laboratory B-1
**References:**	Boulanger et al. (1996), "Liquefaction at Moass Landing During Loma Prieta Earthquake"
**Nature of Failure:**	Liquefaction-induced lateral spreading and differential settlement. Buildings spread 1-1.3 m at the ground surface. Fine grained clayey silt boil in the voleyball court near the southeaast of the site.
**Comments:**	Located on the Moss Landing spit, just south of the timber bridge. The facilities consisted of one- and two-story, woodframe structures. and oil and gas storage tanks. The Moss Landing spit is underlain by littoral soils comprising eolian, fluvial, estuarine, and beach deposits of gravel, sand, silt, and clay deposited in a shoreline environment within the zone of tidal fluctuation. Pleistocene and miocene deposits extend to a depth of ~2000 m The clayey silt stratum beneath the east side is 1.5-3 m thick, and is interbedded between dune and beach sands. PGA was recorded in a four-story building 8 km from rupture plane as 0.39 g (alluvial site). Also San Juan Bautista seismographic station, ~20 km from the rupture plane recorded 0.15 g (stiff alluvium). The Moss Landing area is located ~21 km from the rupture plane. Due to deep alluvium which is soft near the surface, PGA at the site is predicted between 0.2-0.3g Based on site response analyses results, a PGA value of 0.24 g was adopted for these studies SPT performed after the earthquake

Summary of Data

	Cetin et al. (2016)	Idriss&Boulanger (2010)	Seed et.al. (1984)		Cetin et al. (2016)	Idriss&Boulanger (2010)	Seed et.al. (1984)
Liquefied?	Yes	Yes		D_{50}	0.800 ± 0.050		
Data Class	A			% Fines	3.0 ± 2.0	3.0	
Critical Depth Range	7.9 - 18.0	15.1		% PI			
Depth to GWT (ft)	7.9	7.9					
σ_v (psf)	1501.8 ± 213.8	1817.0		N	10.3 ± 1.0	11.0	
σ_v' (psf)	1184.5 ± 110.7	1357.6		C_R	0.85	0.95	
a_{max} (g)	0.240 ± 0.036	0.28		C_S	1.00	1.00	
r_d	0.99 ± 0.011	0.95		C_B	1.00	1.00	
CSR	0.196 ± 0.031	0.230		C_E	1.00	1.00	
Equivalent Magnitude	6.93	6.93		C_N	1.34	1.25	
MSF		1.16		$(N_1)_{60}$	11.7 ± 1.1	13.1	
CSRN		0.189					

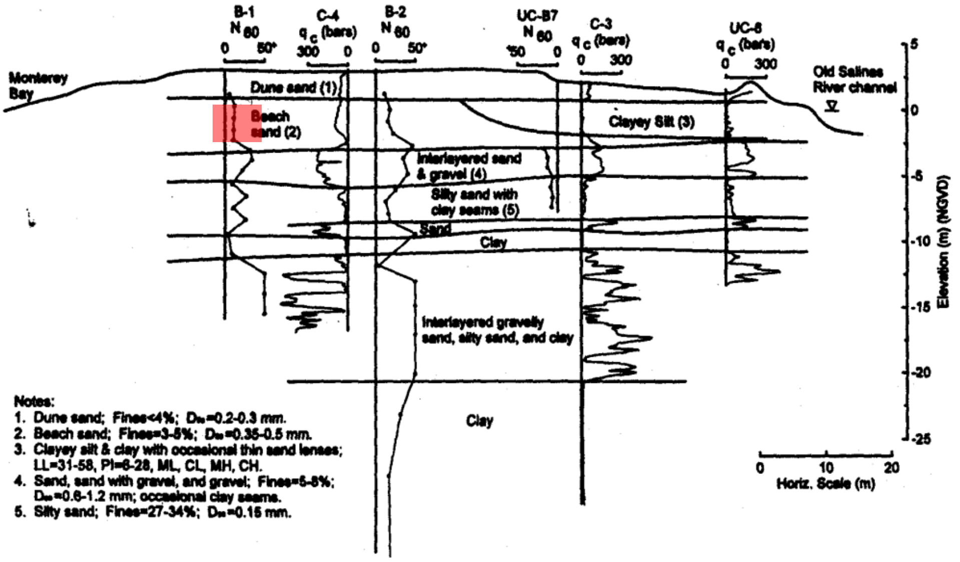

γ_m=110±3 pcf

γ_s=125±3 pcf

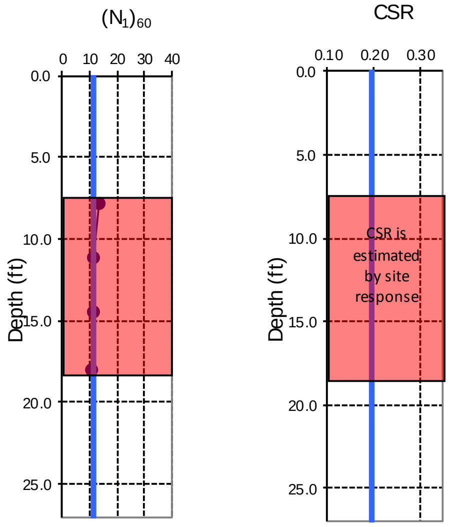

Depth (m)	Depth (ft)	CSR	N	C_N	C_R	$(N1)_{60}$
2.4	7.9		11	1.56	0.77	***13.2***
3.4	11.2		10	1.41	0.82	***11.6***
4.4	14.4		10	1.29	0.87	***11.2***
5.5	18.0		10	1.19	0.91	***10.8***
					**Mean:**	11.7
					**St. Dev.**	1.1

**Case number:**	143
**Earthquake:**	Loma Prieta
**Magnitude:**	6.93 (Mw) Pacific Earthquake Engineering Research Center: NGA Database
**Location:**	Marine Laboratory B-2
**References:**	Boulanger et al. (1996), "Liquefaction at Moss Landing During Loma Prieta Earthquake"
**Nature of Failure:**	Liquefaction-induced lateral spreading and differential settlement.<br>Buildings spread 1-1.3 m at the ground surface. Fine grained clayey silt boil in the volleyball court near the southeaast of the site.
**Comments:**	Located on the Moss Landing spit, just south of the timber bridge.<br>The facilities consisted of one- and two-story, woodframe structures.<br>and oil and gas storage tanks.<br>The Moss Landing spit is underlain by littoral soils comprising eolian, fluvial, estuarine, and beach deposits of gravel, sand, silt, and clay deposited in a shoreline environment within the zone of tidal fluctuation. Pleistocene and miocene deposits extend to a depth of ~2000 m<br>The clayey silt stratum beneath the east side is 1.5-3 m thick, and is interbedded between dune and beach sands.<br>PGA is recorded in a four-story building 8km from rupture plane as 0.39 g which is underlain by alluvium. Also San Juan Bautista seismogram, ~20 km from the rupture plane recorded 0.15 g (stiff alluvium). The Moss Landing area is located ~21 km from the rupture plane. Due to deep alluvium which is soft near the surface, PGA at the site is predicted between 0.2-0.3g<br>Based on site response analyses results, a PGA value of 0.26 g is adopted for this study (a PGA of 0.25 is suggested in the source document).<br>UC-B1 and UC-B2 are ~100 ft apart.<br>SPT performed after the earthquake.

Summary of Data

	Cetin et al. (2016)	Idriss&Boulanger (2010)	Seed et.al. (1984)		Cetin et al. (2016)	Idriss&Boulanger (2010)	Seed et.al. (1984)
Liquefied?	Yes	Yes		D_{50}	0.500 ± 0.050		
Data Class	B			% Fines	3.0 ± 2.0	3.0	
Critical Depth Range	10.0 - 13.0	11.5		% PI			
Depth to GWT (ft)	8.2	8.2					
σ_v (psf)	1314.5 ± 68.0	1357.6		N	13.0 ± 1.7	13.0	
σ_v' (psf)	1108.7 ± 43.4	1148.7		C_R	0.83	0.85	
a_{max} (g)	0.260 ± 0.039	0.28		C_S	1.00	1.00	
r_d	0.99 ± 0.008	0.97		C_B	1.00	1.00	
CSR	0.198 ± 0.030	0.207		C_E	1.00	1.00	
Equivalent Magnitude	6.93	6.93		C_N	1.38	1.35	
MSF		1.16		$(N_1)_{60}$	14.9 ± 2.0	14.9	
CSRN		0.167					

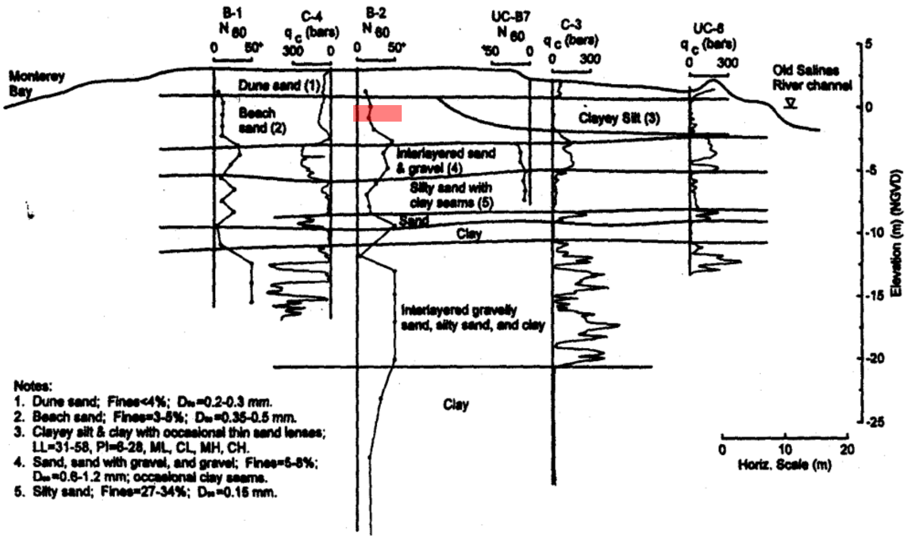

γ_m=110±3 pcf

γ_s=125±3 pcf

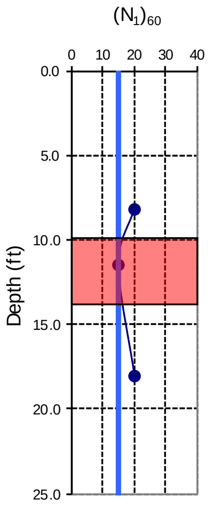

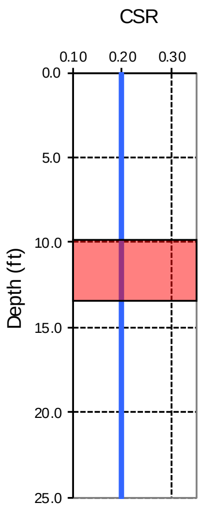

CSR is estimated by site response

Depth (m)	Depth (ft)	CSR	N	C_N	C_R	$(N1)_{60}$
2.5	8.2		17	1.53	0.77	20.2
3.5	11.5		13	1.38	0.83	***14.9***
5.5	18.0		19	1.18	0.91	20.4
					**Mean:**	14.9
					**St. Dev.**	2.0

**Case number:**	144
**Earthquake:**	Loma Prieta
**Magnitude:**	6.93 (Mw) Pacific Earthquake Engineering Research Center: NGA Database
**Location:**	Marine Laboratory F1-F7
**References:**	Boulanger et al. (1995), "Liquefaction at Moss Landing During Loma Prieta Earthquake"
**Nature of Failure:**	"The MLML facilities damaged beyond repair by liquefaction-induced lateral spreading and differential settlement of the site.<br>The survey data shows that the building spread 1 to 1.3 m at the ground surface.<br>Severe cracking occured in the foundation and slab." Boulanger et.al. (1995)
**Comments:**	MLML consist of two building and a west wing surrounding a courtuyard.<br>The northern building is one and two storey wood frame structure founded on spread footings.<br>The southern one and the west wing were one storey wood frame<br>and shear wall structure with concrete slab-on-grade floors. The eastern part of<br>southern building was founded on 0.4 m diameter cast in place concrete piers up<br>to 5.5 m deep. Field investigations were perfomed by WCC (1990) and<br>UC Davis. Additional data were obtained from Fugro (1980).<br>B1,B2,B3 belongs to Woodward-Cylde Consultants (1990)<br>There exist two additional cases: Marine Lab B1 and B2.<br>SPT were performed using a CME automatic trip hammer and a safety hammer<br>with a cathead and rope system. The sampler was a standard 5 cm outside<br>diameter split spoon with room for liners. Sample taken with the liners omitted.<br>Energy hammer ratios were estimated as 63% and 75% for<br>the safety hammer and automatic trip hammer systems.<br>Fugro's boreholes located 80 m away from the site.<br>"SPT hammers were of two types: a rope around the cathead "donut" hammer<br>provided by drillers (total weigth of 225 ponds)i and a "free-fall", mechanical<br>trip hammer (total weigth of 210 pounds) manufactured by Pilcon Engineering.<br>Sampling spoon were newly constructed following ASTM-guidlines with the exception<br>that space for liners was provided, but liners were not used. " (Fugro Inc.)<br>In Boulanger et. al. 1995 UCB-6-12 and F1-6 were used.However UCB 6-12<br>boreholes were unavailable. From Boulanger et. al. 1995 it can be inferred that<br>these boreholes are CPT logs.<br>Therefore F1-7 logs are used in Marine Laboratory F1-F7 site.

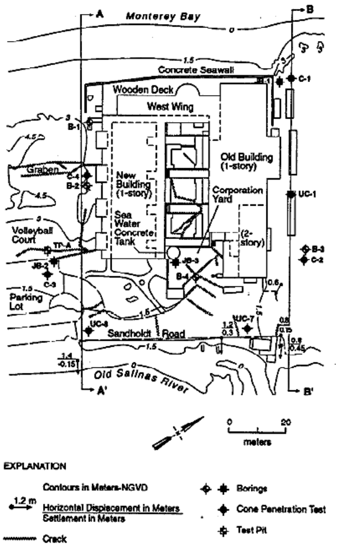

FIG. 9-1. Map of the Moss Landing Marine Laboratory (MLML) Site

Summary of Data

	Cetin et al. (2016)	Idriss&Boulanger (2010)	Seed et.al. (1984)		Cetin et al. (2016)	Idriss&Boulanger (2010)	Seed et.al. (1984)
Liquefied?	Yes	Yes		D_{50}	0.425 ± 0.075	-	
Data Class	B	-		% Fines	3.0 ± 1.4	3.0	
Critical Depth Range	11.5 - 18.5	17.4		% PI	-	-	
Depth to GWT (ft)	5.0	4.9					
σ_v (psf)	1850.0 ± 149.6	2130.3		N	13.8 ± 4.7	12.0	
σ_v' (psf)	1226.0 ± 80.8	1336.7		C_R	0.87	0.95	
a_{max} (g)	0.250 ± 0.075	0.280		C_S	1.00	1.00	
r_d	0.99 ± 0.007	0.940		C_B	1.00	1.00	
CSR	0.243 ± 0.073	0.270		C_E	1.25	1.25	
Equivalent Magnitude	6.93	6.9		C_N	1.31	1.23	
MSF		1.16		$(N_1)_{60}$	19.8 ± 6.7	17.6	
CSRN		0.22					

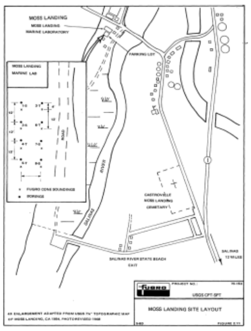

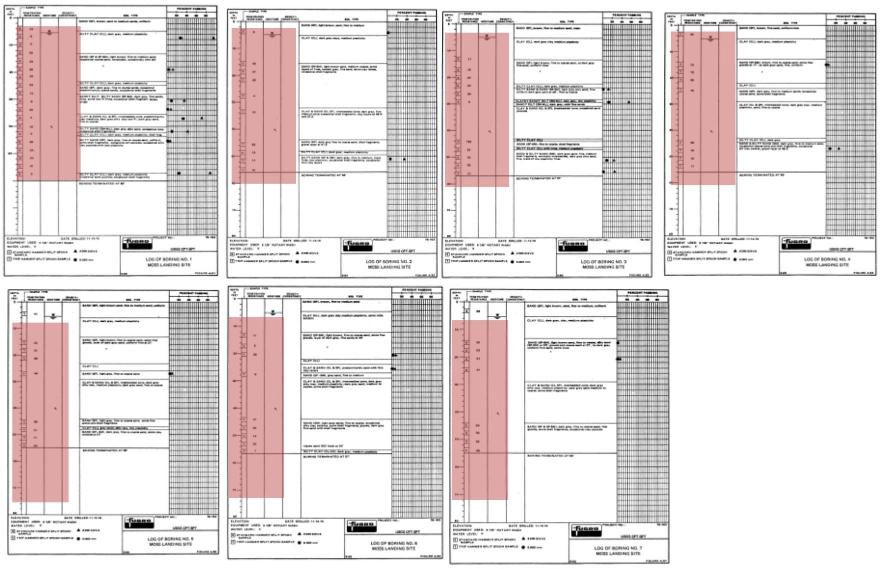

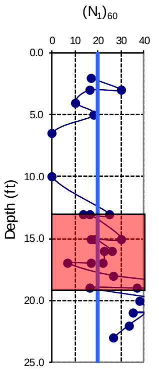

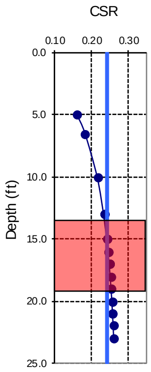

Depth (m)	Depth (ft)	CSR	N	C_N	C_R	$(N1)_{60}$	FC	Borehole	Soil Type	Hammer Type
0.61	2.0	NA	13	2.00	0.61	*16.7*	-	S-1	sand	S
0.91	3.0	NA	22	2.00	0.65	*30.0*	-	S-7	sand	S
0.91	3.0	NA	10	2.00	0.65	*16.2*	-	T-4	sand	T
1.22	4.0	NA	6	2.00	0.68	*10.2*	2	T-2	sand	T
1.52	5.0	0.162	11	1.88	0.71	*18.2*	-	T-5	sand	T
1.98	6.5	0.184	0	1.75	0.74	*0.0*	-	S-1	silty clay	S
3.05	10.0	0.217	0	1.52	0.81	*0.0*	-	S-1	silty clay	S
3.96	13.0	0.234	20	1.39	0.85	*24.7*	12	S-1	silty clay	S
3.96	13.0	0.234	11	1.39	0.85	***13.6***	**-**	S-3	sand	S
3.96	13.0	0.234	11	1.39	0.85	***16.2***	**-**	T-6	sand	T
4.57	15.0	0.243	25	1.31	0.87	***30.2***	***2.0***	S-7	sand	S
4.57	15.0	0.243	12	1.31	0.87	***17.2***	***4.0***	T-4	sand	T
4.57	15.0	0.243	13	1.31	0.87	***18.7***	**-**	T-5	sand	T
4.88	16.0	0.246	22	1.28	0.89	***26.2***	**-**	S-1	sand	S
4.88	16.0	0.246	16	1.28	0.89	***22.7***	**-**	T-2	sand	T
5.18	17.0	0.249	19	1.25	0.90	***22.4***	**-**	S-3	sand	S
5.18	17.0	0.249	12	1.25	0.90	***16.8***	**-**	T-4	sand	T
5.18	17.0	0.249	5	1.25	0.90	***7.0***	**-**	T-6	sand	T
5.49	18.0	0.252	23	1.22	0.91	***26.8***	**-**	S-7	sand	S
5.79	19.0	0.255	65	1.20	0.92	*75.0*	-	S-1	sand	S
5.79	19.0	0.255	27	1.20	0.92	*37.1*	-	T-2	sand	T
5.79	19.0	0.255	12	1.20	0.92	*16.5*	-	T-5	sand	T
6.10	20.0	0.257	44	1.17	0.93	*50.2*	-	S-3	sand	S
6.10	20.0	0.257	28	1.17	0.93	*38.1*	8	T-6	sand	T
6.40	21.0	0.259	42	1.15	0.94	*47.5*	-	S-1	sand	S
6.40	21.0	0.259	57	1.15	0.94	*64.4*	3	S-7	sand	S
6.40	21.0	0.259	26	1.15	0.94	*35.0*	-	T-4	sand	T
6.40	21.0	0.259	30	1.15	0.94	*40.4*	-	T-5	sand	T
6.71	22.0	0.260	25	1.13	0.95	*33.3*	-	T-2	sand	T
7.01	23.0	0.262	24	1.11	0.95	*26.6*	-	S-3	silty clay	S
					**Mean:**	19.8	3.0			
					**St. Dev.**	6.7	1.4			

**Case number:**	145
**Earthquake:**	Loma Prieta
**Magnitude:**	6.93 (Mw) Pacific Earthquake Engineering Research Center: NGA Database
**Location:**	MBARI N0.4-B4B5EB2EB3
**References:**	Boulanger et al. (1995), "Liquefaction at Moass Landing During Loma Prieta Earthquake"
**Nature of Failure:**	"Ground cracks were mapped at a few spots toward the eastern side of the current site of Building 4 and the lot on its south side" Boulanger et. al. 1997

Comments:

The MBARI facilities include three main buildings and a pier located along Sandholt Road on the Moss Landing spit.
Construction of No.4 building started in 1994.
B4, B5, EB2, EB3 is used for the liquefaction analysis by Boulanger et. al. 1995.
However in Cetin 2014 B5 and EB3 was used due to fact that B4 borehole data was unavailable and from EB-3 borehole data consists of silty clay layers.
The site consists of 8-12 m of poorly graded sand (FC=5%; 1-8%, D50=0.20-2.1 mm) with some interlayers.
For SI-2 inlinometer D50=0.40-0.70 mm. Sand layer is underlained by clay layer of low plasticity (ML and CL).
The magnitude of the Loma Prieta is given by USGS equal to 6.9. The acceleration value is recommended by Boulanger (1995) 0.20-0.30 g. In Cetin 2014 it is taken as 0.25 g. However in Boulanger 2010 study it is taken as 0.28 g.
"The SPT were performed by Rutherford and Chekene (1988,1993) by Taber Consultans using a Concore rig. A fish tail bit was used for drilling. SPT testes were performed by using a safety hammer, a cathead and rope system with 2 wraps of the rope around the cathead and a 5-cm-outside diameter split spoon sampler with room for liners. A few samples were obtained using a California split spoon sampler (6.3-cm outside-diameter). Energy measurements for this rig and the procedures while drilling at shallow depth in soft clay on another profectindicated an average anergy ratio of 65%.Given the differences in energy ratios that may arise from different opertaors and soil conditionsi it seems reasonable to conclude that the blow counts reported by R&C (1988,1993) can be essentially taken as N60 values." Boulanger et.al. (1995)
From B5 water table is located at 2.3 m depth and from EB3 it is located at 1.6 m. An average value of 1.95 m is assigned as ground water table for the site. FC data is missing.
The FC data is given in the output section in Boulanger 1995 on a graph however since the ground level information is missing it is unclear in which depth these fines content can be assigned.
In the text in Boulanger et.al (1995) it is stated that "The site consists of 8-12 m of poorly graded sand (FC=5%; 1-8%, D50=0.20-2.1 mm) with some interlayers. In this study FC=5% is taken for all depths.
Sea depth is 3 m. B4 is located 59 m away from sea, EB3 is located 33 m away from sea and EB2 is located 84 m away from sea. Thus, the site is a free field.

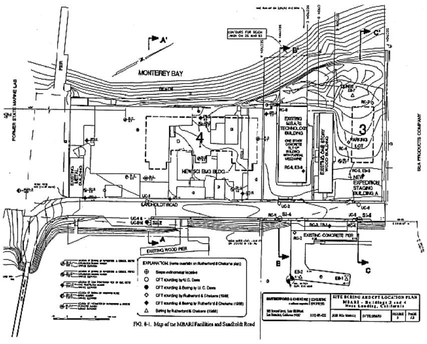

Summary of Data

	Cetin et al. (2016)	Idriss&Boulanger (2010)	Seed et.al. (1984)		Cetin et al. (2016)	Idriss&Boulanger (2010)	Seed et.al. (1984)
Liquefied?	No	No		D_{50}	0.550 ± 0.150	-	
Data Class	B	-		% Fines	5.0 ± 2.0	5.00	
Critical Depth Range	7.9 - 25.3	11.15		% PI	-	-	
Depth to GWT (ft)	6.4	6.23					
s_v (psf)	2039.0 ± 364.1	1294.9		N	23.6 ± 5.5	18.00	
s_v' (psf)	1405.3 ± 206.5	1002.5		C_R	0.89	0.85	
a_{max} (g)	0.250 ± 0.075	0.28		C_S	1.00	1.00	
r_d	0.98 ± 0.007	0.97		C_B	1.00	1.00	
CSR	0.230 ± 0.072	0.23		C_E	1.00	1.00	
Equivalent Magnitude	6.93	6.93		C_N	1.23	1.38	
MSF		1.16		$(N_1)_{60}$	25.8 ± 6.0	21.20	
CSRN		0.18					

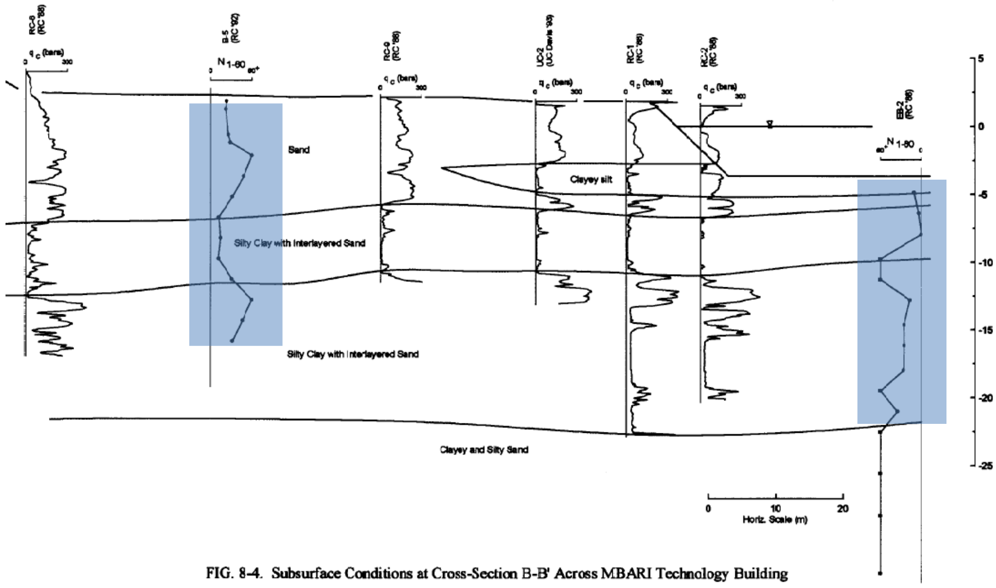

γ_m=120±3 pcf

γ_s=125±3 pcf

FIG. 8-4. Subsurface Conditions at Cross-Section B-B' Across MBARI Technology Building

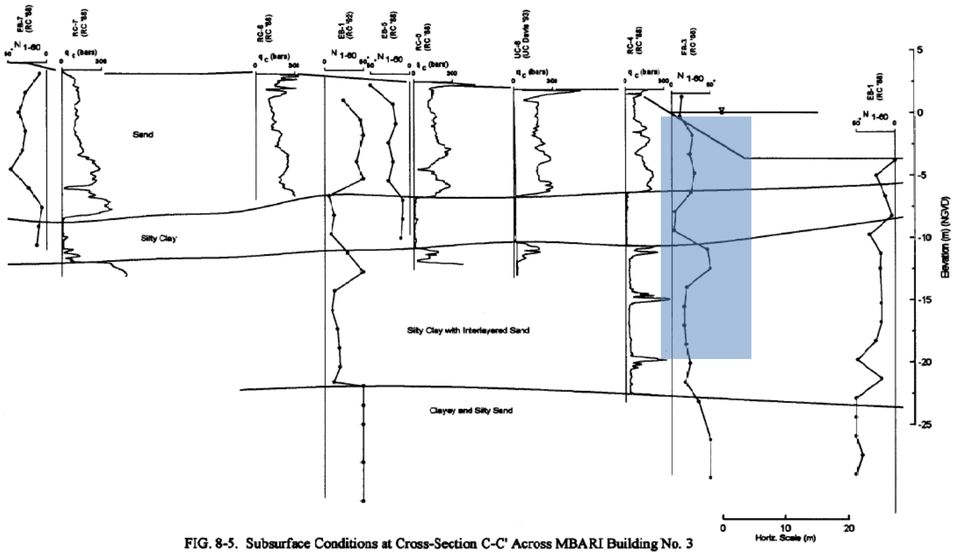

FIG. 8-5. Subsurface Conditions at Cross-Section C-C' Across MBARI Building No. 3

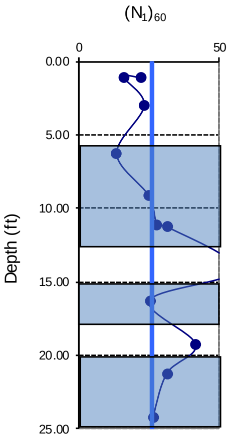

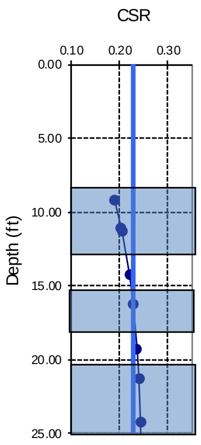

Depth (m)	Depth (ft)	CSR	N	C_N	C_R	$(N1)_{60}$	FC	Borehole	Soil Type
0.34	1.12	NA	19	2.00	0.58	*21.9*	*5.0*	B5	sand
0.34	1.12	NA	14	2.00	0.58	*16.2*	*5.0*	EB3	sand
0.92	3.02	NA	18	2.00	0.65	*23.4*	*5.0*	B5	sand
1.92	6.30	NA	11	1.67	0.74	***13.5***	***5.0***	EB3	sand
2.79	9.15	0.190	21	1.50	0.79	***24.9***	***5.0***	B5	sand
3.39	11.12	0.205	24	1.41	0.82	***27.8***	***5.0***	B5	sand
3.43	11.25	0.206	27	1.40	0.82	***31.3***	***5.0***	EB3	sand
4.33	14.21	0.221	50	1.30	0.86	*56.1*	*5.0*	B5	sand
4.96	16.27	0.229	23	1.24	0.89	***25.3***	***5.0***	EB3	sand
5.87	19.26	0.237	39	1.16	0.92	*41.6*	*5.0*	B5	sand
6.47	21.23	0.241	30	1.12	0.94	***31.4***	***5.0***	EB3	sand
7.38	24.21	0.244	26	1.06	0.96	***26.6***	***5.0***	B5	sand
7.99	26.21	0.245	25	1.03	0.98	*25.1*	*5.0*	EB3	silty clay
8.9	29.20	0.245	9	0.98	1.00	*8.8*	*5.0*	B5	sand
9.54	31.30	0.243	5	0.95	1.00	*4.8*	*5.0*	EB3	silty clay
10.38	34.06	0.241	12	0.92	1.00	*11.0*	*5.0*	B5	silty clay2
11.06	36.29	0.238	3	0.90	1.00	*2.7*	*5.0*	EB3	silty clay
11.93	39.14	0.233	9	0.87	1.00	*7.8*	*5.0*	B5	silty clay2
12.58	41.27	0.230	46	0.85	1.00	*38.9*	*5.0*	EB3	silty clay2
13.43	44.06	0.225	25	0.82	1.00	*20.6*	*5.0*	B5	silty clay2
14.08	46.19	0.222	49	0.81	1.00	*39.5*	*5.0*	EB3	silty clay2
14.96	49.08	0.217	50	0.78	1.00	*39.2*	*5.0*	B5	silty clay3
15.58	51.12	0.215	20	0.77	1.00	*15.4*	*5.0*	EB3	silty clay2
16.49	54.10	0.211	38	0.75	1.00	*28.5*	*5.0*	B5	silty clay3
17.16	56.30	0.209	16	0.74	1.00	*11.8*	*5.0*	EB3	silty clay2
17.98	58.99	0.207	26	0.72	1.00	*18.8*	*5.0*	B5	silty clay3
18.66	61.22	0.206	17	0.71	1.00	*12.1*	*5.0*	EB3	silty clay2
20.17	66.17	0.204	19	0.68	1.00	*13.0*	*5.0*	EB3	silty clay2
21.68	71.13	0.204	23	0.66	1.00	*15.2*	*5.0*	EB3	silty clay2
23.21	76.15	0.203	17	0.64	1.00	*10.9*	*5.0*	EB3	silty clay2
24.77	81.27	0.203	35	0.62	1.00	*21.8*	*5.0*	EB3	silty clay2
					**Mean:**	25.8	5.0		
					**St. Dev.**	6.0	2.0		

**Case number:**	146
**Earthquake:**	Loma Prieta
**Magnitude:**	6.93 (Mw) Pacific Earthquake Engineering Research Center: NGA Database
**Location:**	Miller Farm Site
**References:**	Holzer et al. (1994), "Observed and predicted ground deformation-Miller Farm lateral spread, Watsonville, California."
**Nature of Failure:**	Lateral spread near the Pajaro River. South of the river ~0.2 m high head scarp which could be followed for 1.7 km. Maximum horizontal displacement of ~30 cm just west of the study area. Sand boils along the area affected by lateral spreading. Eyewitnesses reported that sediment-laden water flowed gently from vents on the lateral spread during and until 20 minuntes after the earthquake.
**Comments:**	Located at the south side of the Pajara River at Clint Miller Farms near Watsonville, CA.<br>Composed of younger and older holocene fluvial deposits.<br>The upper 4-6 m of the younger flood-plain deposits is silty and contains only a few thin sand layers. In the depth interval from 4.6-9.1, sand percentages range from 67-100. This layer includes most of the liquefied layer. Average fines content of the sandy layers if 15%, ranging from 22 % at Miller Farm to 8% at Farris Farm.<br>The depth to the water table was 4.6 m 10 days after the main shock.<br>PGA is recorded in a four-story building 1km west of Miller Farm as 0.39 g.<br>In Open File Report 95-663, Holzer et al., the PGA was given as 0.53 g.<br>Watsonville region was shaken at MMI scale of 8.(~ 0.25-0.50 g ?)<br>The earthquake specific attenuation-relationship suggests for alluvium sites at 4.6 miles (closest distance to the fault) ~0.50-0.65 g.<br>A PGA value of 0.36 g was recorded at Watsonville.<br>Based on site response analyses results, a PGA value of 0.42 is adopted.<br>Strong ground motion lasted ~ 6 seconds.<br>SPT performed after the earthquake.

Summary of Data

	Cetin et al. (2016)	Idriss&Boulanger (2010)	Seed et.al. (1984)		Cetin et al. (2016)	Idriss&Boulanger (2010)	Seed et.al. (1984)
Liquefied?	Yes			D_{50}	0.160 ± 0.050		
Data Class	A			% Fines	22.0 ± 2.0		
Critical Depth Range	13.1 - 26.2			% PI			
Depth to GWT (ft)	13.1						
σ_v (psf)	2263.8 ± 277.0			N	8.0 ± 3.4		
σ_v' (psf)	1854.3 ± 144.8			C_R	0.92		
a_{max} (g)	0.420 ± 0.063			C_S	1.00		
r_d	0.90 ± 0.017			C_B	1.00		
CSR	0.300 ± 0.048			C_E	1.13		
Equivalent Magnitude	6.9			C_N	1.07		
MSF				$(N_1)_{60}$	8.9 ± 3.8		
CSRN							

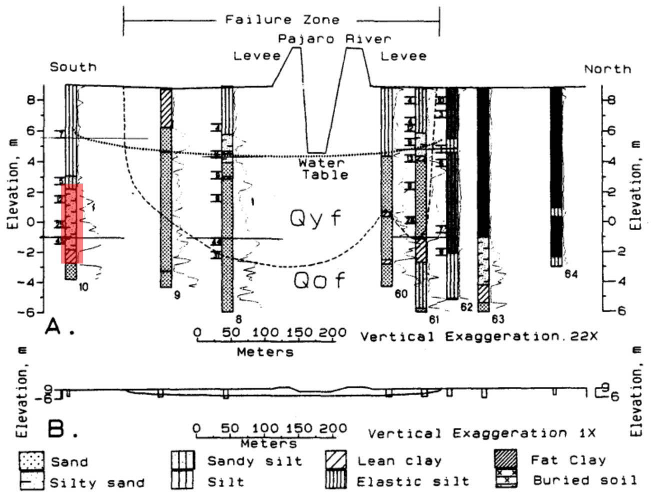

γ_m=110±3 pcf

γ_s=125±3 pcf

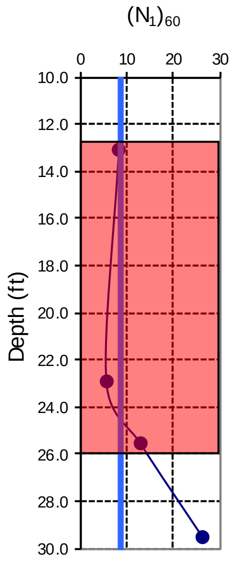

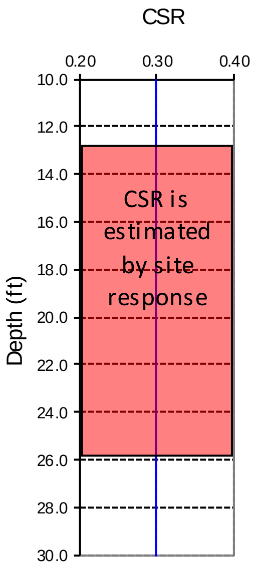

Depth (m)	Depth (ft)	CSR	N	C_N	C_R	$(N1)_{60}$	
4	13.1		7	1.21	0.85	***8.2***	
7	23.0		5	1.01	0.95	***5.5***	HOLZER
7.8	25.6		12	0.98	0.97	***12.9***	HOLZER
9	29.5		25	0.93	1.00	26.2	HOLZER
					**Mean:**	8.9	
					**St. Dev.**	3.8	

**Case number:**	147
**Earthquake:**	1990 Luzon
**Magnitude:**	7.7 (M_w)
**Location:**	Cereenan St. B-12
**References:**	Wakamatsu (1992)
**Nature of Failure:**	No surface evidence of liquefaction
**Comments:**	The Luzon, Philippines Earthquake of July 16, 1990 occurred as result of relative movement between the Eurasian and Philippine Sea plate. A fine soil layer with SPT-values between 10 and 40, exists near the ground surface or several meters below it. Under this layer, there exists a sand layer containing silt and clayey layer. The ground surface is composed of sandy soil, which is thought to be fill, as well as natural levee and or point-bar deposits, or clayey soil from back marsh or swamp deposits. The thickness of the liquefied layer is on the order of 5-10 m. In addition the unliquefied layer above it is thin, being less than 3 m deep.<br>The SPT energy used in Philippines is about half of the Japanese energy values. PGA was estimated as 0.2 g in the source document. PGA contour maps suggests a value in between 0.2-0.3g.

**Summary of Data**	Cetin et al. (2016)	Idriss&Boulanger (2010)	Seed et.al. (1984)		Cetin et al. (2016)	Idriss&Boulanger (2010)	Seed et.al. (1984)
Liquefied?	No	No		D_{50}	0.200 ± 0.050		
Data Class	B			% Fines	19.0 ± 2.0	19.0	
Critical Depth Range	7.9 - 24.6	16.4		% PI	5		
Depth to GWT (ft)	7.5 ± 0.3	7.5					
σ_v (psf)	1916.8 ± 350.3	1963.2		N	34.8 ± 7.0	34.7	
σ_v' (psf)	1374.3 ± 178.5	1420.2		C_R	0.92	0.95	
a_{max} (g)	0.250 ± 0.075	0.25		C_S	1.00	1.00	
r_d	0.99 ± 0.069	0.97		C_B	1.00	1.00	
CSR	0.225 ± 0.070	0.218		C_E	0.65	0.65	
Equivalent Magnitude	7.7	7.7		C_N	1.24	1.16	
MSF		0.95		$(N_1)_{60}$	25.8 ± 5.2	24.9	
CSRN		0.213					

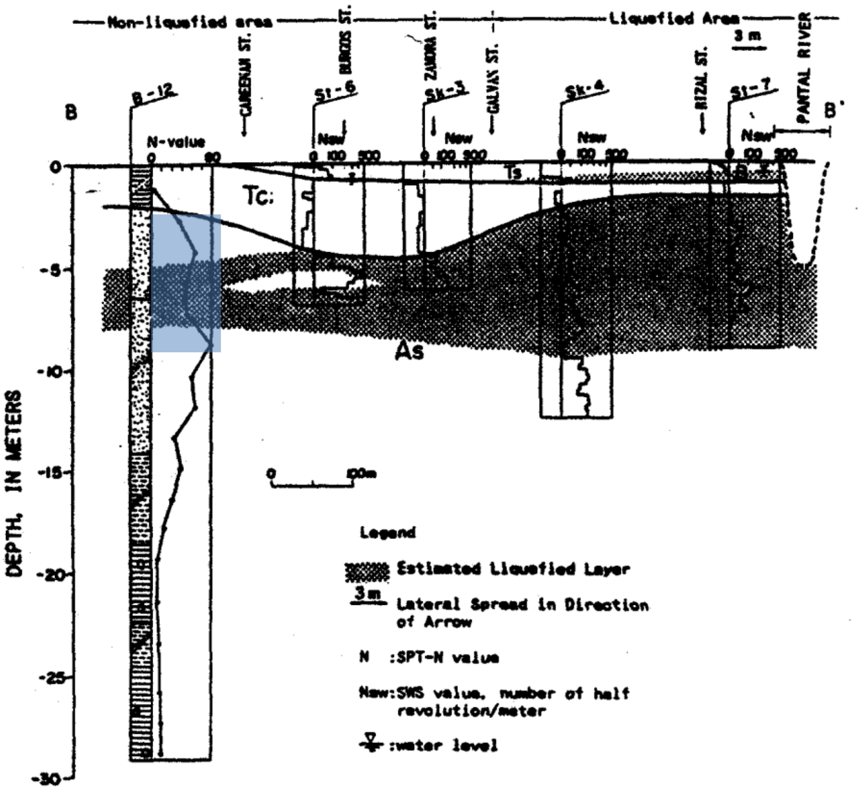

γ_m=110±3 pcf

γ_s=125±3 pcf

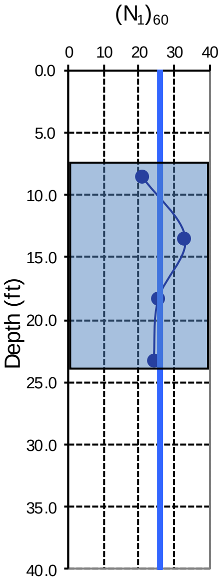

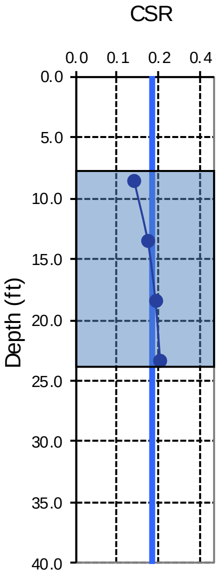

Depth (m)	Depth (ft)	CSR	N	C_N	C_R	$(N1)_{60}$
2.6	8.5	0.173	25.0	1.54	0.83	***20.7***
4.1	13.5	0.211	43.0	1.33	0.89	***33.1***
5.6	18.4	0.233	35.0	1.18	0.94	***25.3***
7.1	23.3	0.246	35.0	1.08	0.98	***24.1***
					**Mean:**	25.8
					**St. Dev.**	5.2

**Case number:**	148
**Earthquake:**	1990 Luzon
**Magnitude:**	7.7 (M_w)
**Location:**	Perez Blv. B-11
**References:**	Wakamatsu (1992)
**Nature of Failure:**	Cracks, lateral spread and tilting of bulidings
**Comments:**	The Luzon, Philippines Earthquake of July 16, 1990 occurred as result of relative movement between the Eurasian and Philippine Sea plate. A fine soil layer with SPT-values between 10 and 40, exists near the ground surface or several meters below it. Under this layer, there exists a sand layer containing silt and clayey layer. The ground surface is composed of sandy soil, which is thought to be fill, as well as natural levee and or point-bar deposits, or clayey soil from back marsh or swamp deposits. The thickness of the liquefied layer is on the order of 5-10 m. In addition the unliquefied layer above it is thin, being less than 3 m deep.<br>The SPT energy used in Philippines is about half of the Japanese energy values.<br>PGA was estimated as 0.2 g in the source document. PGA contour maps suggests a value in between 0.2-0.3g.

Summary of Data

	Cetin et al. (2016)	Idriss&Boulanger (2010)	Seed et.al. (1984)		Cetin et al. (2016)	Idriss&Boulanger (2010)	Seed et.al. (1984)
Liquefied?	Yes	Yes		D_{50}	0.200 ± 0.050		
Data Class	B			% Fines	19.0 ± 2.0	19.0	
Critical Depth Range	13.1 - 34.4	23.6		% PI	5		
Depth to GWT (ft)	7.5 ± 0.3	7.5					
σ_v (psf)	2784.6 ± 447.6	2903.1		N	19.9 ± 4.0	19.9	
σ_v' (psf)	1771.2 ± 229.2	1879.7		C_R	0.98	0.95	
a_{max} (g)	0.250 ± 0.075	0.25		C_S	1.00	1.00	
r_d	0.89 ± 0.096	0.94		C_B	1.00	1.00	
CSR	0.227 ± 0.073	0.236		C_E	0.65	0.65	
Equivalent Magnitude	7.7	7.7		C_N	1.09	1.06	
MSF		0.95		$(N_1)_{60}$	13.9 ± 2.8	13.0	
CSRN		0.245					

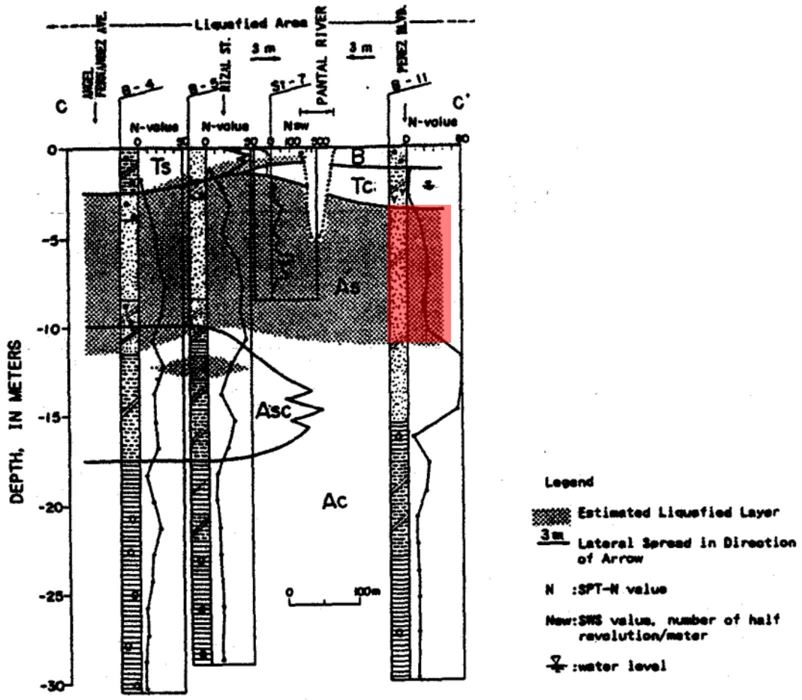

γ_m=100±3 pcf

γ_s=125±3 pcf

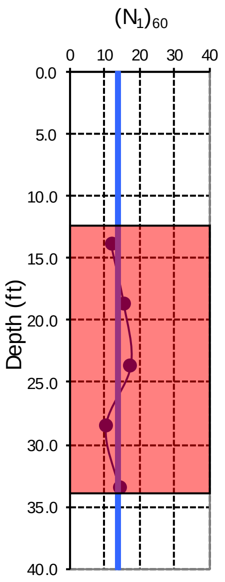

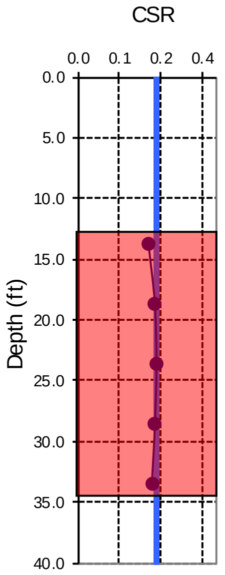

Depth (m)	Depth (ft)	CSR	N	C_N	C_R	$(N1)_{60}$
4.2	13.8	0.209	15.0	1.36	0.89	***11.9***
5.7	18.7	0.223	21.0	1.21	0.94	***15.5***
7.2	23.6	0.227	25.0	1.10	0.98	***17.5***
8.7	28.5	0.223	16.0	1.01	1.00	***10.5***
10.2	33.5	0.216	23.0	0.94	1.00	***14.1***
					**Mean:**	13.9
					**St. Dev.**	2.8

**Case number:**	149
**Earthquake:**	1993 Kushiro-Oki
**Magnitude:**	7.8 (Mjma)
**Location:**	Kushiro Port Strong Motion Recording Station
**References:**	Iai et al (1994)
**Nature of Failure:**	Liquefaction of the backfill sand of quay walls; displacements of quay walls. Sudden change in the response of ground after 30 seconds.
**Comments:**	On January 15, 1993, an earthquake of magnitude 7.8 in JMA scale hit a northern island of Japan called Hokkaido. The epicenter was located 15 km south of Kushiro City. Kushiro Port was shaken with a peak horizontal acceleration of 0.47 and 0.34g. The earthquake caused liquefaction of untreated backfill sand at the port, resulting in damage to many of the quay walls. The extent of the damage was generally moderate; the displacements of the quay walls were less than about 5% of the water depth in front of the quay walls. In contrast, the quay walls having the backfill sand compacted as measures against liquefaction survived the earthquake without damage.<br>Kushiro Port has been developed at the estuary of the Kushiro River. Most of the port area has been reclaimed from the sea by filling the sand dredged from the nearby sea. The natural ground along the former beach line is of sand dune origin being dense with SPT N values of about 50. The fill above the original ground gradually increases its thickness from none at the former beach line to about 10 m at the southern end of the piers in the West Port District. SPT was conducted before the earthquake.

Summary of Data

	Cetin et al. (2016)	Idriss&Boulanger (2010)	Seed et.al. (1984)		Cetin et al. (2016)	Idriss&Boulanger (2010)	Seed et.al. (1984)
Liquefied?	Yes	Yes		D_{50}	0.350 ± 0.050		
Data Class	A			% Fines	5.0 ± 2.0	5.0	
Critical Depth Range	5.2 - 18.4	12.5		% PI			
Depth to GWT (ft)	6.6 ± 0.3	6.6					
σ_v (psf)	1378.0 ± 274.6	1357.6		N	16.9 ± 2.0	17.4	
σ_v' (psf)	1050.4 ± 139.9	981.6		C_R	0.87	0.85	
a_{max} (g)	0.400 ± 0.060	0.47		C_S	1.00	1.00	
r_d	0.99 ± 0.053	0.98		C_B	1.00	1.00	
CSR	0.338 ± 0.059	0.410		C_E	1.22	1.30	
Equivalent Magnitude	7.6	7.6		C_N	1.42	1.35	
MSF		0.97		$(N_1)_{60}$	25.4 ± 3.0	25.9	
CSRN		0.383					

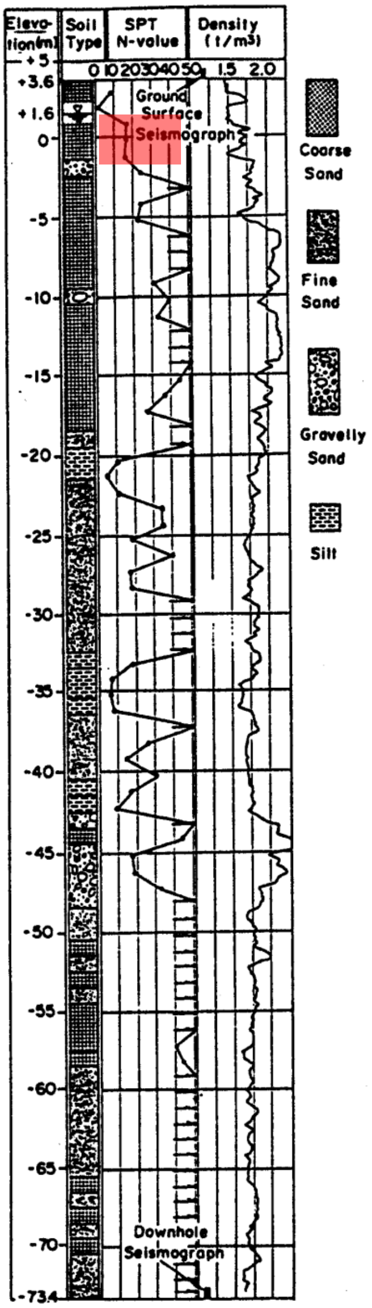

γ_m=110±3 pcf

γ_s=125±3 pcf

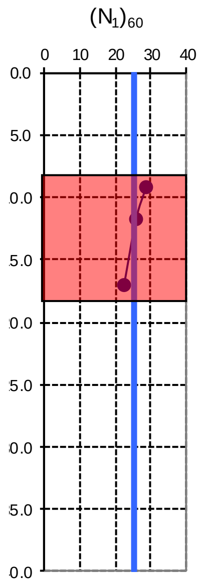

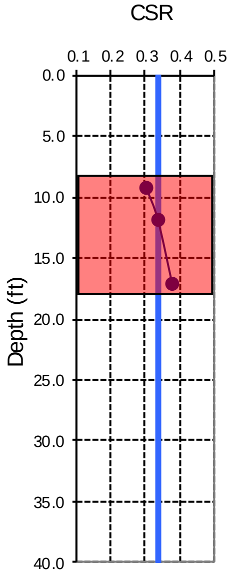

Depth (m)	Depth (ft)	CSR	N	C_N	C_R	$(N1)_{60}$
2.8	9.2	0.306	18.0	1.55	0.84	***28.3***
3.6	11.8	0.338	17.0	1.42	0.87	***25.6***
5.2	17.1	0.378	16.0	1.24	0.93	***22.4***
					**Mean:**	25.4
					**St. Dev.**	3.0

**Case number:**	150
**Earthquake:**	1993 Kushiro-Oki
**Magnitude:**	7.8 (Mjma)
**Location:**	Quay Wall Site A
**References:**	Iai et al (1994)
**Nature of Failure:**	Settlement and lateral movement of quay wall couple of meters.
**Comments:**	On January 15, 1993, an earthquake of magnitude 7.8 in JMA scale hit a northern island of Japan called Hokkaido. The epicenter was located 15 km south of Kushiro City. Kushiro Port was shaken with a peak horizontal acceleration of 0.47 and 0.34g. The earthquake caused liquefaction of untreated backfill sand at the port, resulting in damage to many of the quay walls. The extent of the damage was generally moderate; the displacements of the quay walls were less than about 5% of the water depth in front of the quay walls. In contrast, the quay walls having the backfill sand compacted as measures against liquefaction survived the earthquake without damage. Kushiro Port is developed at the estuary of the Kushiro River. Most of the port area has been reclaimed from the sea by filling the sand dredged from the nearby sea. The natural ground along the former beach line is of sand dune origin being dense with SPT N values of about 50. The fill above the original ground gradually increases its thickness from none at the former beach line to about 10 m at the southern end of the piers in the West Port District. SPT was conducted before the earthquake.

Summary of Data

	Cetin et al. (2016)	Idriss&Boulanger (2010)	Seed et.al. (1984)		Cetin et al. (2016)	Idriss&Boulanger (2010)	Seed et.al. (1984)
Liquefied?	Yes	Yes		D_{50}	0.340 ± 0.050		
Data Class	A			% Fines	2.0 ± 2.0	2.0	
Critical Depth Range	13.1 - 21.3	17.1		% PI			
Depth to GWT (ft)	6.6 ± 0.3	6.6					
σ_v (psf)	2021.8 ± 175.1	2088.5		N	11.7 ± 2.9	11.7	
σ_v' (psf)	1356.5 ± 94.3	1420.2		C_R	0.93	0.95	
a_{max} (g)	0.400 ± 0.060	0.40		C_S	1.00	1.00	
r_d	0.96 ± 0.073	0.96		C_B	1.00	1.00	
CSR	0.372 ± 0.063	0.366		C_E	1.22	1.22	
Equivalent Magnitude	7.6	7.6		C_N	1.25	1.21	
MSF		0.97		$(N_1)_{60}$	16.5 ± 4.1	16.4	
CSRN		0.359					

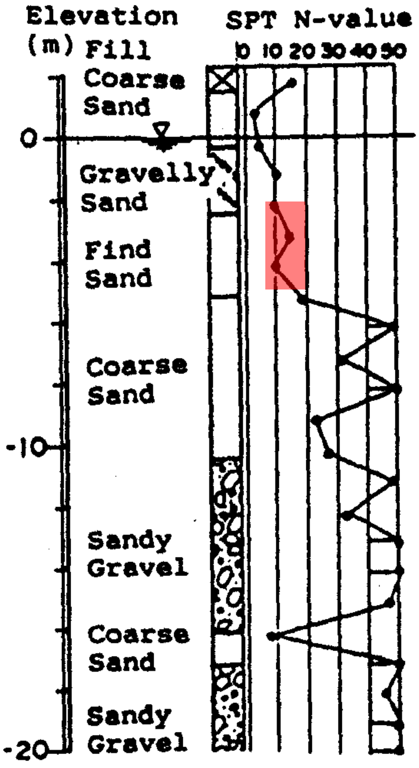

γ_m=105±3 pcf

γ_s=125±3 pcf

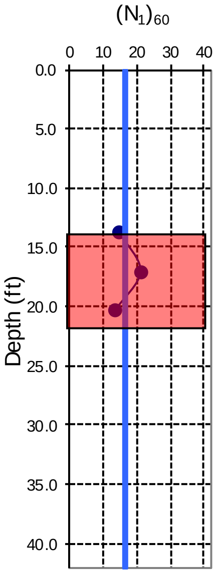

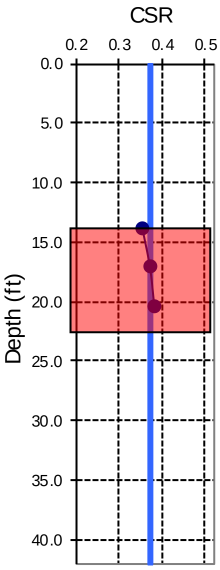

Depth (m)	Depth (ft)	CSR	N	C_N	C_R	$(N1)_{60}$
4.2	13.8	0.353	10.0	1.36	0.89	***14.8***
5.2	17.1	0.372	15.0	1.25	0.93	***21.2***
6.2	20.3	0.382	10.0	1.17	0.96	***13.6***
					**Mean:**	16.5
					**St. Dev.**	4.1

**Case number:**	151
**Earthquake:**	1993 Kushiro-Oki
**Magnitude:**	7.8 (Mjma)
**Location:**	Quay Wall Site D
**References:**	Iai et al (1994)
**Nature of Failure:**	No surface evidence of liquefaction
**Comments:**	On January 15, 1993, an earthquake of magnitude 7.8 in JMA scale hit a northern island of Japan called Hokkaido. The epicenter was located 15 km south of Kushiro City. Kushiro Port was shaken with a peak horizontal acceleration of 0.47 and 0.34g. The earthquake caused liquefaction of untreated backfill sand at the port, resulting in damage to many of the quay walls. The extent of the damage was generally moderate; the displacements of the quay walls were less than about 5% of the water depth in front of the quay walls. In contrast, the quay walls having the backfill sand compacted as measures against liquefaction survived the earthquake without damage.<br>Kushiro Port was developed at the estuary of the Kushiro River. Most of the port area has been reclaimed from the sea by filling the sand dredged from the nearby sea. The natural ground along the former beach line is of sand dune origin being dense with SPT N values of about 50. The fill above the original ground gradually increases its thickness from none at the former beach line to about 10 m at the southern end of the piers in the West Port District. SPT was conducted before the earthquake. The Site had been improved by sand compaction piles.

Summary of Data

	Cetin et al. (2016)	Idriss&Boulanger (2010)	Seed et.al. (1984)		Cetin et al. (2016)	Idriss&Boulanger (2010)	Seed et.al. (1984)
Liquefied?	No	No		D_{50}	0.340 ± 0.050		
Data Class	B			% Fines	0.0 ± 2.0	0.0	
Critical Depth Range	24.6 - 45.9	35.4		% PI			
Depth to GWT (ft)	5.2 ± 0.3	5.2					
σ_v (psf)	4382.4 ± 453.6	4344.2		N	26.7 ± 3.1	26.8	
σ_v' (psf)	2509.2 ± 241.2	2464.5		C_R	1.00	1.00	
a_{max} (g)	0.400 ± 0.060	0.40		C_S	1.00	1.00	
r_d	0.88 ± 0.134	0.89		C_B	1.00	1.00	
CSR	0.398 ± 0.086	0.408		C_E	1.22	1.22	
Equivalent Magnitude	7.6	7.6		C_N	0.92	0.95	
MSF		0.97		$(N_1)_{60}$	29.8 ± 3.5	30.9	
CSRN		0.434					

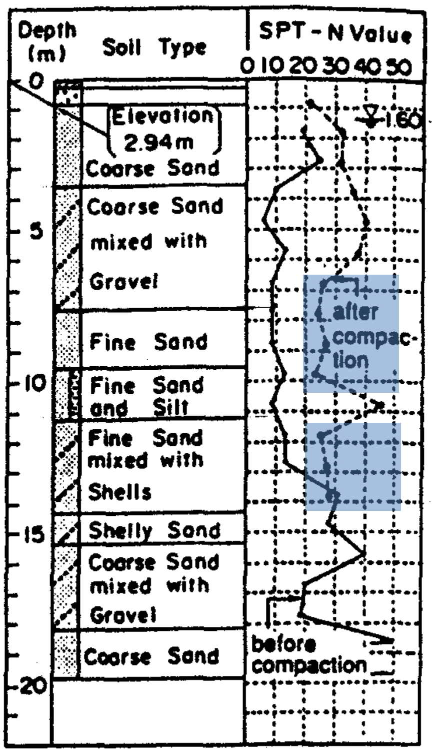

γ_m=120±3 pcf

γ_s=125±3 pcf

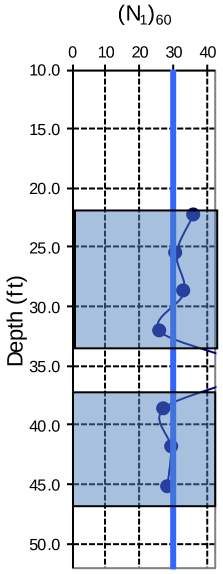

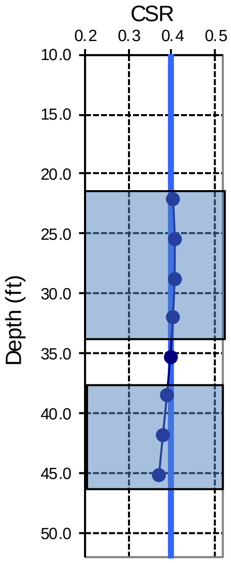

Depth (m)	Depth (ft)	CSR	N	C_N	C_R	$(N1)_{60}$	
6.8	22.1	0.405	27.0	1.12	0.97	***35.7***	
7.8	25.4	0.408	24.0	1.06	0.99	***30.7***	
8.8	28.7	0.408	27.0	1.00	1.00	***33.0***	
9.8	32.0	0.404	22.0	0.96	1.00	***25.7***	
10.8	35.3	0.398	46.0	0.92	1.00	51.4	gravel?
11.8	38.5	0.390	25.0	0.88	1.00	***26.9***	
12.8	41.8	0.381	28.0	0.85	1.00	***29.0***	
13.8	45.1	0.372	28.0	0.82	1.00	***28.0***	
					**Mean:**	29.8	
					**St. Dev.**	3.5	

**Case number:**	152
**Earthquake:**	1994 Northridge Earthquake
**Magnitude:**	6.7 (M_w)
**Location:**	Balboa Blv. Unit C
**References:**	Bennett et al. (1998)
**Nature of Failure:**	Cracks and lateral spread
**Comments:**	The Northridge Earthquake of January 17, 1994 caused widespread ground deformations on the gently sloping alluvial fan surface of the San Fernando Valley. The San Fernando Valley is a structural valley in southern California, which is filled with up to 4500 m of Tertiary sedimentary rock and alluvial sediment. Holocene age alluvial gravels, sands, and finer sediment with an aggregate thickness ranging from ~8-12 m immediately underlie the gently sloping valley floor.The alluvium was deposited by sediment-laden floodwaters that flowed out of the surrounding mountain canyons.<br>The Balboa Boulevard site is on the northern margin of the San Fernando Valley where a 5 km long belt defined by a complex pattern of ground cracks formed in the Granada and Mission Hills. Ground cracks in most of the belt developed in Holocene alluvium on a gentle south-sloping surface. The site is underlain by Holocene silty sand to lean clay that ranges in thickness from 8-10 m.<br>The uppermost unit, A, is less than 1 m thick and consists of fill and reworked sandy silt and lean clay with sand. The upper part of the primary Holocene section, unit B, consists of sheet flood and debris flow deposits and the lower part unit C, consists of fluvial deposits. The locations of maximum compression and extension correlate closely with where the water table falls below the base of the Holocene fluvial unit C. The part of unit C below water table has an average fines content of 52% and clay content of 18 %. Only 8 SPTs in unit C are below water table. Unit D is much coarser grained but is still very silty. Average fines content is 48%. 44 SPT's in unit D.<br>PGA was recorded as 0.83 and 0.47g 2.3 km east of the study site at Los Angles Department of Water and Power Rinaldi Receiving station. Other nearby recorded PGA's were as high as 0.94 g; contoured regional maps of peak acceleration imply a value of 0.85 g. Site response analyses results suggests a value of 0.7 g.

Summary of Data

	Cetin et al. (2016)	Idriss&Boulanger (2010)	Seed et.al. (1984)		Cetin et al. (2016)	Idriss&Boulanger (2010)	Seed et.al. (1984)
Liquefied?	Yes	Yes		D_{50}	0.099 ± 0.040		
Data Class	B			% Fines	48.0 ± 15.0	50.0	
Critical Depth Range	27.1 - 32.0	27.9		% PI	11		
Depth to GWT (ft)	23.6 ± 0.4	23.6					
σ_v (psf)	3336.6 ± 126.0	3258.1		N	19.9 ± 4.2	13.6	
σ_v' (psf)	2968.1 ± 91.3	2986.6		C_R	1.00	1.00	
a_{max} (g)	0.693 ± 0.104	0.84		C_S	1.00	1.00	
r_d	0.71 ± 0.005	0.87		C_B	1.00	1.00	
CSR	0.357 ± 0.054	0.428		C_E	1.13	1.13	
Equivalent Magnitude	6.7	6.7		C_N	0.84	0.86	
MSF		1.24		$(N_1)_{60}$	19.0 ± 4.0	13.1	
CSRN		0.362					

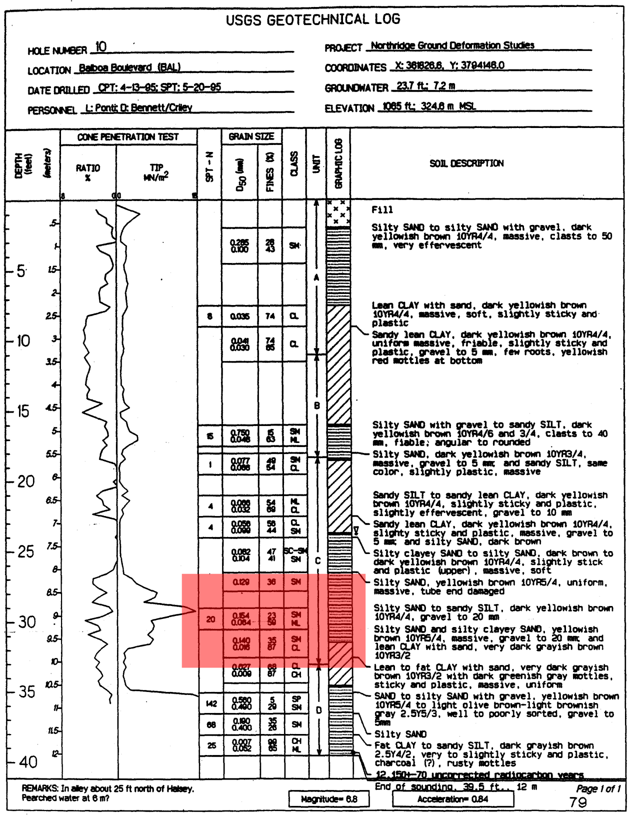

γ_m=110±3 pcf

γ_s=125±3 pcf

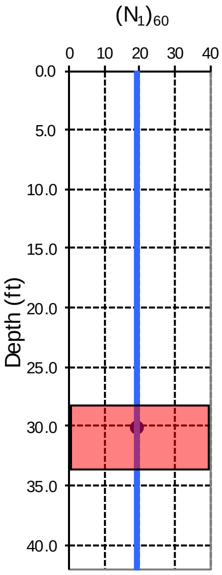

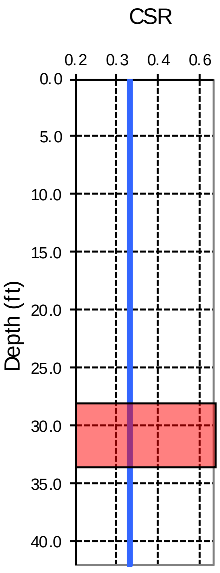

CSR is estimated by site response

Depth (m)	Depth (ft)	CSR	N	C_N	C_R	$(N1)_{60}$
9.2	30.2		20.0	0.84	1.00	***19.0***
					**Mean:**	19.0
					**St. Dev.**	4.0

given in the source document

**Case number:**	153
**Earthquake:**	1994 Northridge Earthquake
**Magnitude:**	6.7 (Mw)
**Location:**	Potrero Canyon Unit C
**References:**	Bennett et al. (1998)
**Nature of Failure:**	Cracks and lateral spread
**Comments:**	The Northridge Earthquake of January 17, 1994 caused widespread ground deformations on the gently sloping alluvial fan surface of the San Fernando Valley. The San Fernando Valley is a structural valley in southern California, which is filled with up to 4500 m of Tertiary sedimentary rock and alluvial sediment. Holocene age alluvial gravels, sands, and finer sediment with an aggregate thickness ranging from ~8-12 m immediately underlie the gently sloping valley floor.The alluvium was deposited by sediment-laden floodwaters that flowed out of the surrounding mountain canyons.<br>Potrero Canyon is a narrow 5 km long E-W valley incised into Pliocene sedimentary rock on the north flank of the Santa Susana Mountains. Altough it is about 22 km NNW of the epicenter of the Northridge earthquake, it is near the up dip projection of the seismogenic rupture surface. Ground failure in Potrero Canyon consisted of two zones of ground cracks along the north and south edges of the valley. Cracks formed in alluvium at or near the contact between the valley fill and the Pliocene bedrock forming the adjacent valley walls. Horizontal displacements across ground cracks were at least 10 cm.<br>The Valley is underlain by fine grained Holocene sediment, units A and C, most of which is below the water table. Depth to groundwater was about 2.5 m. The uppermost unit, A, is a homogeneous lean clay to silt with an average thickness of 2.7 m. The remainder of Holocene section, unit C, consists of lean clay to sandy silt with lenses of silt and silty sand, C1. The Holocene sediment rests on dense silty sand, which may either Pleistocene or part of Pliocene Pico Formation, unit D. The average fines and clay contents for unit C are 57 and 11 % respectively.<br>PGA was recorded as 0.43 and 0.37g 3.8 km east of the study site at NWS. Contoured regional maps of peak acceleration imply a value of 0.35 g

**Summary of Data**	Cetin et al. (2016)	Idriss&Boulanger (2010)	Seed et.al. (1984)		Cetin et al. (2016)	Idriss&Boulanger (2010)	Seed et.al. (1984)
Liquefied?	Yes	Yes		D_{50}	0.102 ± 0.011		
Data Class	B			% Fines	44.5 ± 2.1	64.0	
Critical Depth Range	19.7 - 23.0	23.3		% PI	5		
Depth to GWT (ft)	10.8 ± 0.4	6.6					
σ_v (psf)	2503.3 ± 82.1	2903.1		N	9.5 ± 0.6	7.4	
σ_v' (psf)	1848.2 ± 59.1	1837.9		C_R	0.94	0.95	
a_{max} (g)	0.399 ± 0.120	0.43		C_S	1.00	1.00	
r_d	0.79 ± 0.087	0.91		C_B	1.00	1.00	
CSR	0.279 ± 0.089	0.320		C_E	1.13	1.13	
Equivalent Magnitude	6.7	6.7		C_N	1.07	1.07	
MSF		1.24		$(N_1)_{60}$	10.8 ± 0.7	8.5	
CSRN		0.254					

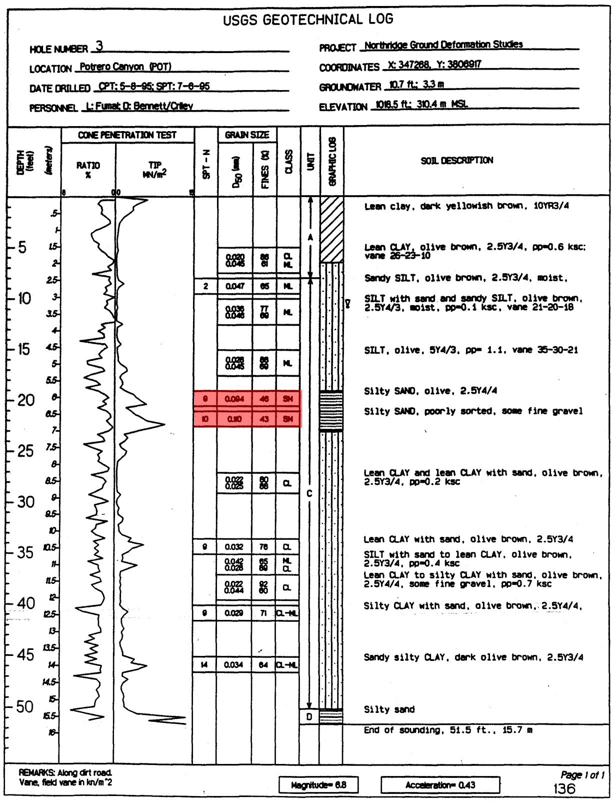

γ_m=110±3 pcf

γ_s=125±3 pcf

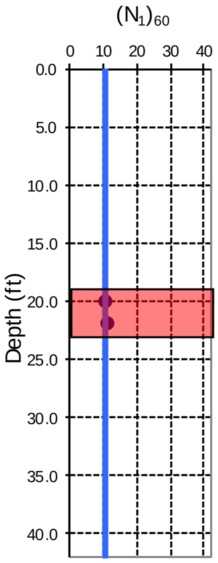

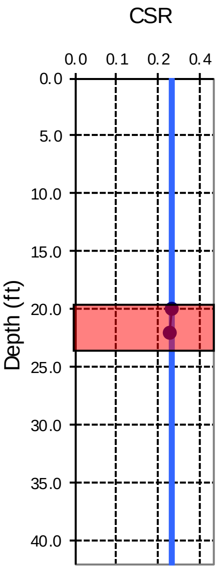

Depth (m)	Depth (ft)	CSR	N	C_N	C_R	$(N1)_{60}$	FC (%)	D50 (mm)
6.1	20.0	0.279	9.0	1.09	0.93	***10.4***	***46.0***	***0.094***
6.7	22.0	0.278	10.0	1.06	0.95	***11.3***	***43.0***	***0.110***
					**Mean:**	10.8	44.5	0.102
					**St. Dev.**	0.7	2.1	0.011

**Case number:**	154
**Earthquake:**	1994 Northridge Earthquake
**Magnitude:**	6.7 (Mw)
**Location:**	Wynne Ave. Unit C1
**References:**	Bennett et al. (1998)
**Nature of Failure:**	Cracks and lateral spread
**Comments:**	The Northridge Earthquake of January 17, 1994 caused widespread ground deformations on the gently sloping alluvial fan surface of the San Fernando Valley. The San Fernando Valley is a structural valley in southern California, which is filled with up to 4500 m of Tertiary sedimentary rock and alluvial sediment. Holocene age alluvial gravels, sands, and finer sediment with an aggregate thickness ranging from ~8-12 m immediately underlie the gently sloping valley floor.The alluvium was deposited by sediment-laden floodwaters that flowed out of the surrounding mountain canyons.<br>The Wynne Ave. site is located couple of kms north east from the epicenter.<br>Ground failure at the Wynne Ave. site consisted of an 150 m long by 12 m wide down dropped block. Vertical offset across cracks ranged from 100-200 mm.<br>Groundwater beneath the failure zone was was at a depth of about 4.3 m.<br>The site is underlain by 2 m of silty sand and sandy lean clay, unit A, which in turn is underlain by 12-14 m of Holocene sandy silt to lean clay, unit B and C.<br>Unit B consists of multiple fining upward sequences of overbank and sheetflood deposits that were deposited in a flood basin between major distributary flood channels. Unit C, which is texturally comparable to B, is distinguished by the presence of lenticular sand layers. Immediately beneath the failure, two sand layers are intercalated in unit C at depths of 6 and 10 m. The shallower sand layer, C1, is about 2 m thick. Deeper sand layer C2, is 1.5 thick. Both layers have average fines and clay content of 38 and 11 %.<br>PGA was recorded as 0.47 and 0.36g 1.7 km south-east of the study site at NRG.<br>Contoured regional maps of peak acceleration imply a value of 0.50 g. Site response based estimation of PGA is 0.54 g

**Summary of Data**	Cetin et al. (2016)	Idriss&Boulanger (2010)	Seed et.al. (1984)		Cetin et al. (2016)	Idriss&Boulanger (2010)	Seed et.al. (1984)
Liquefied?	Yes	Yes		D_{50}	0.106 ± 0.037		
Data Class	A			% Fines	42.4 ± 8.9	33.0	
Critical Depth Range	18.9 - 22.1	22.0		% PI			
Depth to GWT (ft)	13.7 ± 1.5	14.1					
σ_v (psf)	2358.1 ± 85.5	2694.2		N	10.3 ± 1.5	11.0	
σ_v' (psf)	1931.6 ± 93.1	2193.0		C_R	0.93	0.95	
a_{max} (g)	0.540 ± 0.081	0.51		C_S	1.00	1.00	
r_d	0.86 ± 0.040	0.91		C_B	1.00	1.00	
CSR	0.350 ± 0.061	0.390		C_E	1.13	1.13	
Equivalent Magnitude	6.7	6.7		C_N	1.05	0.98	
MSF		1.24		$(N_1)_{60}$	11.4 ± 1.7	11.6	
CSRN		0.317					

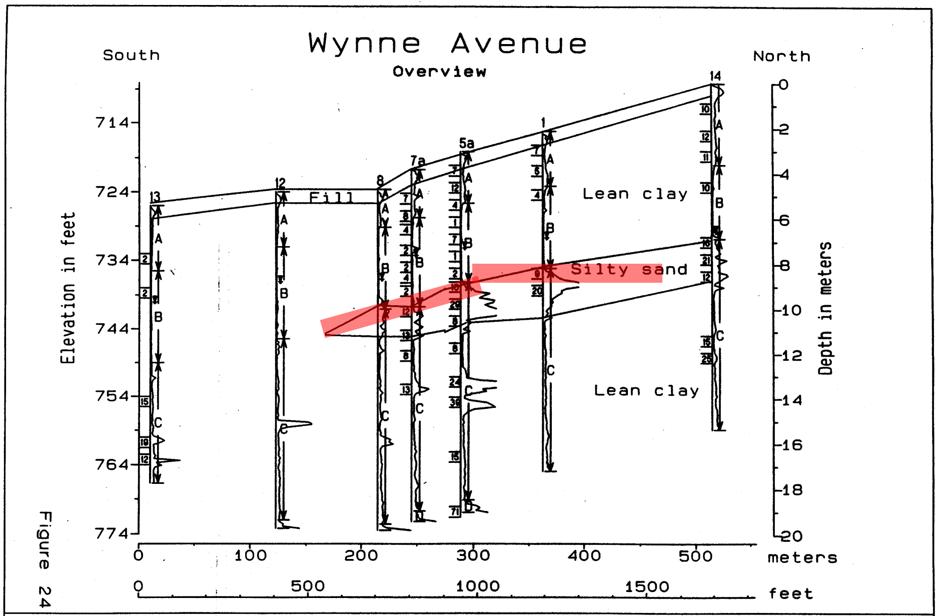

Figure 24

NORTHRIDGE GROUND DEFORMATION STUDIES

33

γ_m=110±3 pcf

γ_s=125±3 pcf

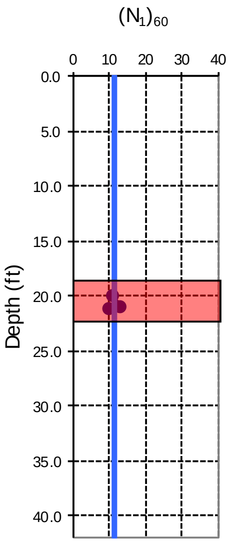

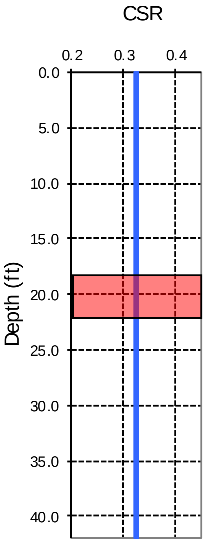

	D_{50} (mm)	FC	
BH-5a	***0.160***	***31.0***	SM
BH-7a	***0.098***	***43.0***	SM
BH-7a	***0.068***	***54.0***	SM??
BH-1a	***0.079***	***47.0***	SM
BH-1a	***0.123***	***37.0***	SM
	0.106	42.4	
	0.037	8.9	

CSR is estimated by site response

Depth (m)	Depth (ft)	CSR	N	C_N	C_R	$(N1)_{60}$	
6.1	20.0		10.0	1.06	0.93	***11.1***	BH-5a
6.4	21.0		12.0	1.04	0.94	***13.2***	BH-7a
6.5	21.2		9.0	1.04	0.94	***9.9***	BH-1a
					**Mean:**	11.4	
					**St. Dev.**	1.7	

454545

**Case number:**	155
**Earthquake:**	1995 Hyogoken-Nambu
**Magnitude:**	7.2 (M_L)
**Location:**	Ashiyama A (Mountain Sand 1)
**References:**	Shibata et al.(1996)
**Nature of Failure:**	From the cracks in roads, building foundations, and around utility poles, massive amounts of sand were ejected. Many two-story houses tilted and settled differentially. However, this site is located in the non-liquefied zone.
**Comments:**	On January 17, 1995, an earthquake of magnitude 7.2 (Richter scale) struck the southern part of Hyogo Prefecture. Sand boils and liquefied, ejected soil were observed art numerous sites, mainly in the coastal areas of Hyogo and Osaka Prefectures. In particular, massive liquefaction was observed on man-made islands and inland areas with high ground water tables.<br>The reclaimed island known as Ashiyahama was reclamed by Hyogo Prefecture using mainly Masado, weathered and decomposed low plasticity, low cohesion, high permeability, erodible granite. The reclamation of Ashiyahama began in 1969 and completed in 1975. An alluvial clay layer of 3-5 m in thickness was deposited just beneath the old sea level, and the total thickness of the reclamation is 13-15 m. The fill material used was mainly debris from mountainside construction sites, but also a marine sand layer having an N value of ~10 exists about 5 m below the ground surface. The erupted sand has the characteristics of marine sand layer.<br>In the vicinity of the site, PGA was recorded as 0.3-0.4g at liquefied sites and 0.6-0.8 g at non-liquefied sites. The PGA value of 0.326g, was recorded at Uozakihama, which is 2-3 km south-west of Ashiyahama. A PGA value of 0.4 g was adopted. The average Japanese SPT energy ratio of 73% is used.

Summary of Data

	Cetin et al. (2016)	Idriss&Boulanger (2010)	Seed et.al. (1984)		Cetin et al. (2016)	Idriss&Boulanger (2010)	Seed et.al. (1984)
Liquefied?	No	No		D_{50}	0.126 ± 0.050		
Data Class	B			% Fines	18.0 ± 2.0	18.0	
Critical Depth Range	11.5 - 22.6	17.1		% PI			
Depth to GWT (ft)	11.5 ± 0.3	11.5					
σ_v (psf)	1960.3 ± 235.6	2025.9		N	16.6 ± 5.3	16.6	
σ_v' (psf)	1612.3 ± 123.3	1670.8		C_R	0.93	0.95	
a_{max} (g)	0.400 ± 0.080	0.40		C_S	1.00	1.00	
r_d	0.94 ± 0.072	0.94		C_B	1.00	1.00	
CSR	0.297 ± 0.065	0.295		C_E	1.22	1.22	
Equivalent Magnitude	6.9	6.9		C_N	1.15	1.10	
MSF		1.17		$(N_1)_{60}$	21.5 ± 6.8	21.1	
CSRN		0.243					

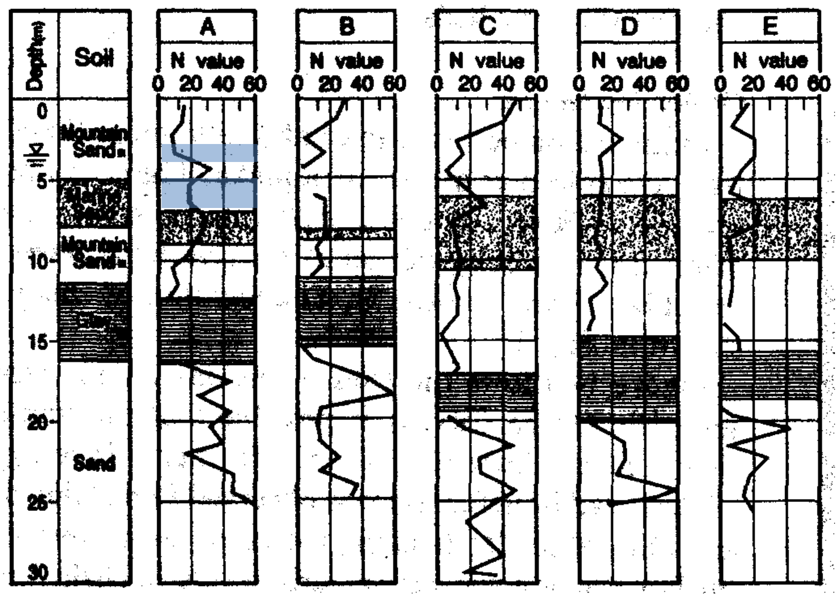

γ_m=110±3 pcf

γ_s=125±3 pcf

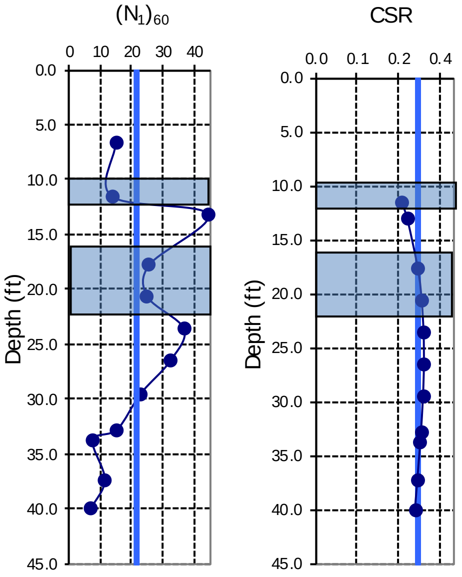

Depth (m)	Depth (ft)	CSR	N	C_N	C_R	$(N1)_{60}$	
2.0	6.6	NA	9.0	1.71	0.80	15.0	
3.5	11.5	0.252	10.0	1.29	0.87	***13.7***	
4.0	13.1	0.269	33.0	1.24	0.89	44.3	Gravel ?
5.4	17.7	0.300	20.0	1.13	0.93	***25.7***	
6.3	20.7	0.311	20.0	1.07	0.96	***25.0***	
7.2	23.6	0.316	30.0	1.02	0.98	36.7	
8.1	26.6	0.317	27.0	0.98	1.00	32.2	
9.0	29.5	0.315	20.0	0.94	1.00	22.9	
10.0	32.8	0.308	14.0	0.90	1.00	15.4	
10.3	33.8	0.306	7.0	0.89	1.00	7.6	
11.4	37.4	0.297	11.0	0.86	1.00	11.5	
12.2	40.0	0.290	7.0	0.83	1.00	7.1	
					**Mean:**	21.5	
					**St. Dev.**	6.8	

**Case number:**	156
**Earthquake:**	1995 Hyogoken-Nambu
**Magnitude:**	7.2 (M_L)
**Location:**	Ashiyama A (Marine Sand)
**References:**	Shibata et al.(1996)
**Nature of Failure:**	From the cracks in roads, building foundations, and around utility poles, massive amounts of sand were ejected. Many two-story houses tilted and settled differentially. However, this site is located in the non-liquefied zone.
**Comments:**	On January 17, 1995, an earthquake of magnitude 7.2 (Richter scale) struck the southern part of Hyogo Prefecture. Sand boils and liquefied, ejected soil were observed at numerous sites, mainly in the coastal areas of Hyogo and Osaka Prefectures. In particular, massive liquefaction was observed on man-made islands and inland areas with high ground water tables.<br>The island known as Ashiyahama was reclamed by Hyogo Prefecture using mainly Masado, weathered and decomposed low plasticity, low cohesion, high permeability, erodible granite. The reclamation of Ashiyahama began in 1969 and completed in 1975. An alluvial clay layer of 3-5 m in thickness was deposited just beneath the old sea level, and the total thickness of the reclamation is 13-15 m. The fill material used was mainly debris from mountainside construction sites, but also marine sand layer having an N value of ~10 exists about 5 m below the ground surface. The erupted sand has the characteristics of marine sand layer.<br>In the vicinity of the site, PGA was recorded as 0.3-0.4 g at liquefied sites and 0.6-0.8 g at non-liquefied sites. The PGA value of 0.326g, was recorded at Uozakihama, which is 2-3 km south-west of Ashiyahama. A PGA value of 0.4 g was adopted.The average Japanese SPT energy ratio of 73% is used.

Summary of Data

	Cetin et al. (2016)	Idriss&Boulanger (2010)	Seed et.al. (1984)		Cetin et al. (2016)	Idriss&Boulanger (2010)	Seed et.al. (1984)
Liquefied?	No			D_{50}	0.185 ± 0.050		
Data Class	B			% Fines	2.0 ± 2.0		
Critical Depth Range	22.6 - 29.5			% PI			
Depth to GWT (ft)	11.5 ± 0.3						
σ_v (psf)	3088.1 ± 154.0			N	25.5 ± 5.8		
σ_v' (psf)	2177.1 ± 92.1			C_R	1.00		
a_{max} (g)	0.400 ± 0.080			C_S	1.00		
r_d	0.86 ± 0.104			C_B	1.00		
CSR	0.317 ± 0.074			C_E	1.22		
Equivalent Magnitude	6.9			C_N	0.99		
MSF				$(N_1)_{60}$	30.6 ± 7.0		
CSRN							

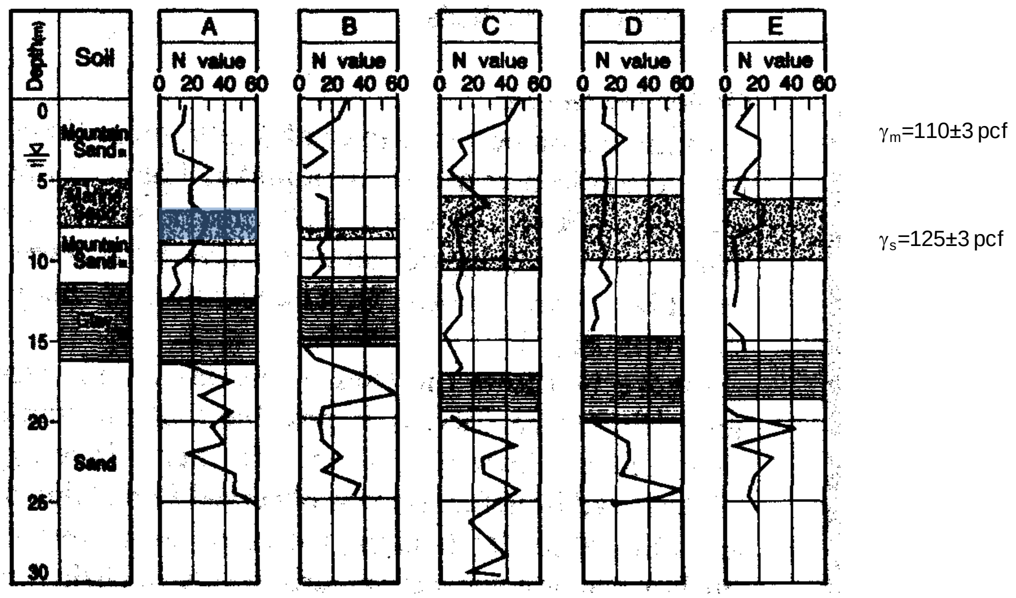

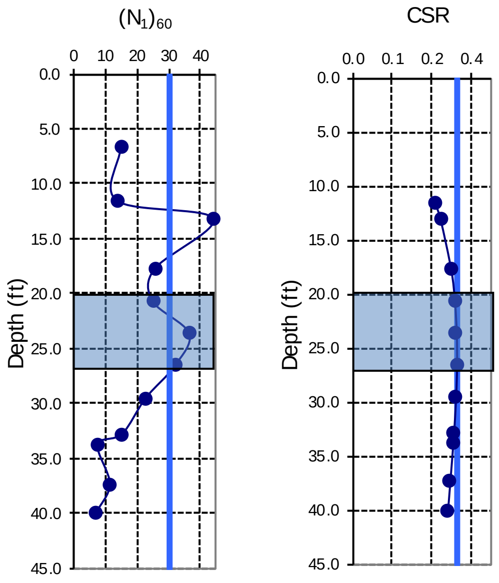

Depth (m)	Depth (ft)	CSR	N	C_N	C_R	$(N1)_{60}$	
2.0	6.6	NA	9.0	1.71	0.80	15.0	
3.5	11.5	0.252	10.0	1.29	0.87	13.7	
4.0	13.1	0.269	33.0	1.24	0.89	44.3	Gravel ?
5.4	17.7	0.300	20.0	1.13	0.93	25.7	
6.3	20.7	0.311	20.0	1.07	0.96	25.0	
7.2	23.6	0.316	30.0	1.02	0.98	***36.7***	
8.1	26.6	0.317	27.0	0.98	1.00	***32.2***	
9.0	29.5	0.315	20.0	0.94	1.00	***22.9***	
10.0	32.8	0.308	14.0	0.90	1.00	15.4	
10.3	33.8	0.306	7.0	0.89	1.00	7.6	
11.4	37.4	0.297	11.0	0.86	1.00	11.5	
12.2	40.0	0.290	7.0	0.83	1.00	7.1	
					**Mean:**	30.6	
					**St. Dev.**	7.0	

**Case number:**	157
**Earthquake:**	1995 Hyogoken-Nambu
**Magnitude:**	7.2 (ML)
**Location:**	Ashiyama C&D&E (Mountain Sand 2)
**References:**	Shibata et al.(1996)
**Nature of Failure:**	From the cracks in roads, building foundations, and around utility poles, massive amounts of sand were ejected. Many two-story houses tilted and settled differentially.
**Comments:**	On January 17, 1995, an earthquake of magnitude 7.2 (Richter scale) struck the southern part of Hyogo Prefecture. Sand boils and liquefied, ejected soil were observed art numerous sites, mainly in the coastal areas of Hyogo and Osaka Prefectures. In particular, massive liquefaction was observed on man-made islands and inland areas with high ground water tables.<br>The island known as Ashiyahama was reclaimed by Hyogo Prefecture using mainly Masado, weathered and decomposed low plasticity, low cohesion, high permeability, erodible granite. The reclamation of Ashiyahama began in 1969 and completed in 1975. An alluvial clay layer of 3-5 m in thickness was deposited just beneath the old sea level, and the total thickness of the reclamation is 13-15 m. The fill material used was mainly debris from mountainside construction sites, but also a merine sand layer having an N value of ~10 exists about 5 m below the ground surface. The erupted sand has the characteristics of marine sand layer.<br>In the vicinity of the site, PGA was recorded as 0.3-0.4g at liquefied sites and 0.6-0.8 g at non-liquefied sites. The PGA value of 0.326g,recorded at Uozakihama, which is 2-3 km south-west of Ashiyahama. A PGA value of 0.40 g was adopted. The average Japanese SPT energy ratio of 73% is used.

Summary of Data

	Cetin et al. (2016)	Idriss&Boulanger (2010)	Seed et.al. (1984)		Cetin et al. (2016)	Idriss&Boulanger (2010)	Seed et.al. (1984)
Liquefied?	Yes			D_{50}	0.126 ± 0.050		
Data Class	B			% Fines	18.0 ± 2.0		
Critical Depth Range	36.1 - 49.2			% PI			
Depth to GWT (ft)	11.5 ± 0.2						
σ_v (psf)	5159.1 ± 291.0			N	5.7 ± 2.7		
σ_v' (psf)	3214.2 ± 169.5			C_R	1.00		
a_{max} (g)	0.400 ± 0.080			C_S	1.00		
r_d	0.59 ± 0.149			C_B	1.00		
CSR	0.246 ± 0.079			C_E	1.22		
Equivalent Magnitude	6.9			C_N	0.81		
MSF				$(N_1)_{60}$	5.6 ± 2.7		
CSRN							

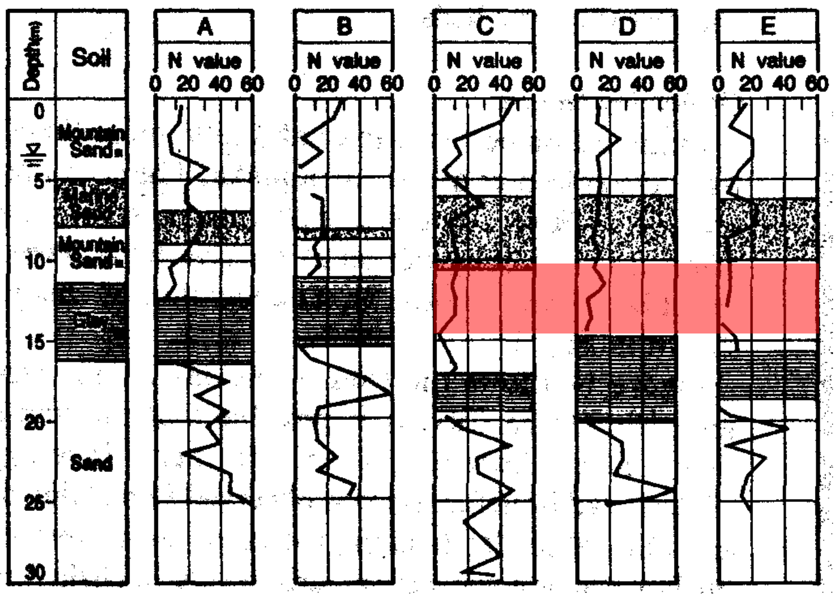

γ_m=110±3 pcf

γ_s=125±3 pcf

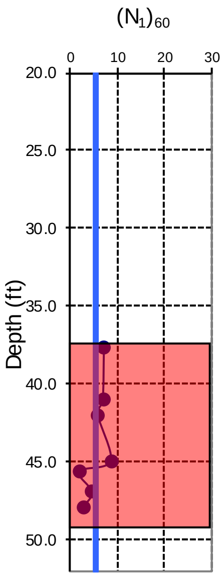

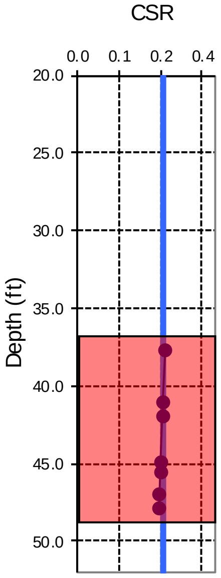

Depth (m)	Depth (ft)	CSR	N	C_N	C_R	$(N1)_{60}$		
11.5	37.7	0.257	7.0	0.85	1.00	***7.3***	Site D	
12.5	41.0	0.249	7.0	0.82	1.00	***7.0***	Site E	
12.8	42.0	0.247	6.0	0.82	1.00	***6.0***	Site E	
13.7	44.9	0.242	9.0	0.79	1.00	***8.7***	Site D	
13.9	45.6	0.241	2.0	0.79	1.00	***1.9***	Site E	
14.3	46.9	0.239	5.0	0.78	1.00	4.7	Site D	clay?
14.6	47.9	0.238	3.0	0.77	1.00	***2.8***	Site C	
					**Mean:**	5.6		
					**St. Dev.**	2.7		

**Case number:**	158
**Earthquake:**	1995 Hyogoken-Nambu
**Magnitude:**	7.2 (M_L)
**Location:**	Ashiyama C&D&E (Marine Sand)
**References:**	Shibata et al.(1996)
**Nature of Failure:**	From the cracks in roads, building foundations, and around utility poles, massive amounts of sand were ejected. Many two-story houses tilted and settled differentially.
**Comments:**	On January 17, 1995, an earthquake of magnitude 7.2 (Richter scale) struck the southern part of Hyogo Prefecture. Sand boils and liquefied, ejected soil were observed at numerous sites, mainly in the coastal areas of Hyogo and Osaka Prefectures. In particular, massive liquefaction was observed on man-made islands and inland areas with high ground water tables.<br>The reclaimed island known as Ashiyahama was reclamed by Hyogo Prefecture using mainly Masado, weathered and decomposed low plasticity, low cohesion, high permeability, erodible granite. The reclamation of Ashiyahama began in 1969 and completed in 1975. An alluvial clay layer of 3-5 m in thickness was deposited just beneath the old sea level, and the total thickness of the reclamation is 13-15 m. The fill material used was mainly debris from mountainside construction sites, but also a marine sand layer having an N value of ~10 exists about 5 m below the ground surface. The erupted sand has the characteristics of marine sand layer.<br>In the vicinity of the site, PGA was recorded as 0.3-0.4g at liquefied sites and 0.6-0.8 g at non-liquefied sites. The PGA value of 0.326g, was recorded at Uozakihama, which is 2-3 km south-west of Ashiyahama. A PGA value of 0.4 g was adopted. The average Japanese SPT energy ratio of 73% is used.

Summary of Data

	Cetin et al. (2016)	Idriss&Boulanger (2010)	Seed et.al. (1984)		Cetin et al. (2016)	Idriss&Boulanger (2010)	Seed et.al. (1984)
Liquefied?	Yes	Yes		D_{50}	0.185 ± 0.050		
Data Class	B			% Fines	2.0 ± 2.0	2.0	
Critical Depth Range	24.6 - 32.8	28.9		% PI			
Depth to GWT (ft)	11.5 ± 0.2	11.5					
σ_v (psf)	3416.2 ± 181.8	3467.0		N	10.9 ± 2.6	10.9	
σ_v' (psf)	2341.4 ± 106.0	2401.8		C_R	1.00	1.00	
a_{max} (g)	0.400 ± 0.080	0.40		C_S	1.00	1.00	
r_d	0.74 ± 0.113	0.88		C_B	1.00	1.00	
CSR	0.281 ± 0.071	0.331		C_E	1.22	1.22	
Equivalent Magnitude	6.9	6.9		C_N	0.95	0.94	
MSF		1.17		$(N_1)_{60}$	12.6 ± 3.0	12.5	
CSRN		0.286					

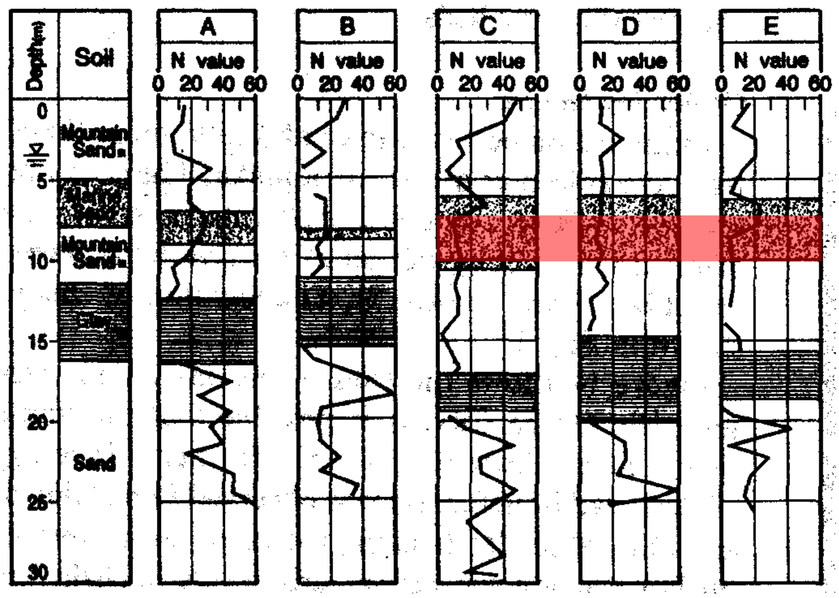

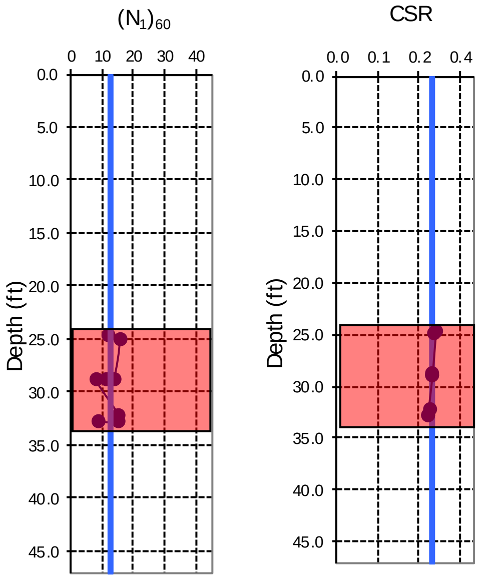

Depth (m)	Depth (ft)	CSR	N	C_N	C_R	$(N1)_{60}$	
7.5	24.6	0.289	10.0	1.01	0.99	***12.1***	Site C
7.6	24.9	0.288	13.0	1.00	0.99	***15.7***	Site D
8.8	28.7	0.281	12.0	0.95	1.00	***13.9***	Site C
8.8	28.9	0.281	10.0	0.95	1.00	***11.5***	Site D
8.8	28.9	0.281	7.0	0.95	1.00	***8.1***	Site E
9.8	32.2	0.272	14.0	0.91	1.00	***15.5***	Site D
10.0	32.8	0.270	14.0	0.90	1.00	***15.4***	Site C
10.0	32.8	0.270	8.0	0.90	1.00	***8.8***	Site E
					**Mean:**	12.6	
					**St. Dev.**	3.0	

**Case number:**	159
**Earthquake:**	1995 Hyogoken-Nambu
**Magnitude:**	7.2 (M_L)
**Location:**	Kobe No : 1
**References:**	Kobe City Office, Construction Engineering Research Institute (1999)
**Nature of Failure:**	Non-liquefied
**Comments:**	

Summary of Data

	Cetin et al. (2016)	Idriss&Boulanger (2010)	Seed et.al. (1984)		Cetin et al. (2016)	Idriss&Boulanger (2010)	Seed et.al. (1984)
Liquefied?	No	No		D_{50}	0.000 ± 0.000		
Data Class	B			% Fines	3.5 ± 4.9	3.0	
Critical Depth Range	16.4 - 23.0	19.0		% PI			
Depth to GWT (ft)	7.7 ± 0.3	7.9					
σ_v (psf)	2541.8 ± 153.8	2360.1		N	42.4 ± 2.5	42.1	
σ_v' (psf)	1794.6 ± 91.3	1670.8		C_R	0.95	0.95	
a_{max} (g)	0.400 ± 0.080	0.40		C_S	1.00	1.00	
r_d	0.99 ± 0.082	0.93		C_B	1.00	1.00	
CSR	0.365 ± 0.079	0.345		C_E	1.22	1.22	
Equivalent Magnitude	6.9	6.9		C_N	1.09	1.07	
MSF		1.17		$(N_1)_{60}$	53.2 ± 3.2	52.0	
CSRN		0.275					

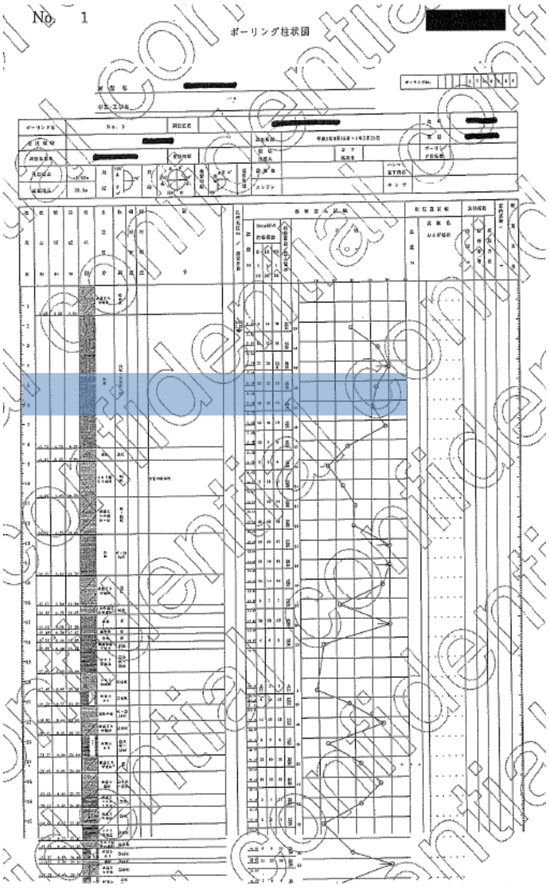

γ_m=120±3 pcf

γ_s=135±3 pcf

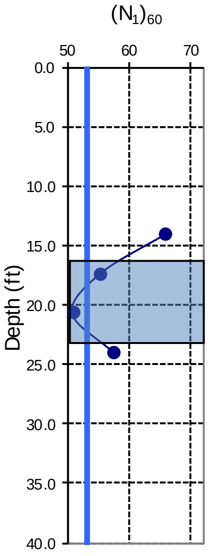

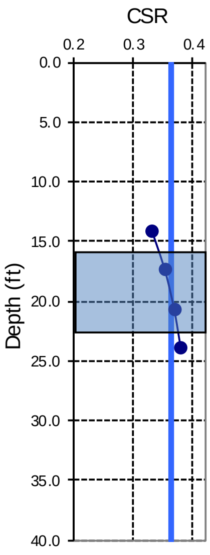

Depth (m)	Depth (ft)	CSR	N	C_N	C_R	$(N1)_{60}$	FC
4.3	14.1	0.333	49.0	1.23	0.90	66.0	
5.3	17.4	0.354	43.0	1.14	0.93	*55.5*	*0.0*
6.3	20.7	0.369	41.0	1.06	0.96	*50.9*	*7.0*
7.3	24.0	0.380	48.0	1.00	0.98	57.6	
					**Mean:**	53.2	3.5
					**St. Dev.**	3.2	4.9

**Case number:**	160
**Earthquake:**	1995 Hypgoken-Nambu
**Magnitude:**	7.2 (M_L)
**Location:**	Kobe No : 2
**References:**	Kobe City Office, Construction Engineering Research Institute (1999)
**Nature of Failure:**	No surface manifestation of liquefaction
**Comments:**	

Summary of Data

	Cetin et al. (2016)	Idriss&Boulanger (2010)	Seed et.al. (1984)		Cetin et al. (2016)	Idriss&Boulanger (2010)	Seed et.al. (1984)
Liquefied?	No	No		D_{50}	0.000 ± 0.000		
Data Class	B			% Fines	14.8 ± 11.5	3.0	
Critical Depth Range	16.4 - 39.4	19.0		% PI			
Depth to GWT (ft)	9.5 ± 0.3	7.9					
σ_v (psf)	3622.5 ± 520.5	2360.1		N	35.2 ± 8.1	42.1	
σ_v' (psf)	2474.0 ± 285.1	1670.8		C_R	1.00	0.95	
a_{max} (g)	0.400 ± 0.080	0.40		C_S	1.00	1.00	
r_d	0.98 ± 0.110	0.93		C_B	1.00	1.00	
CSR	0.373 ± 0.086	0.345		C_E	1.22	1.22	
Equivalent Magnitude	6.9	6.9		C_N	0.92	1.07	
MSF		1.17		$(N_1)_{60}$	39.6 ± 9.1	52.0	
CSRN		0.275					

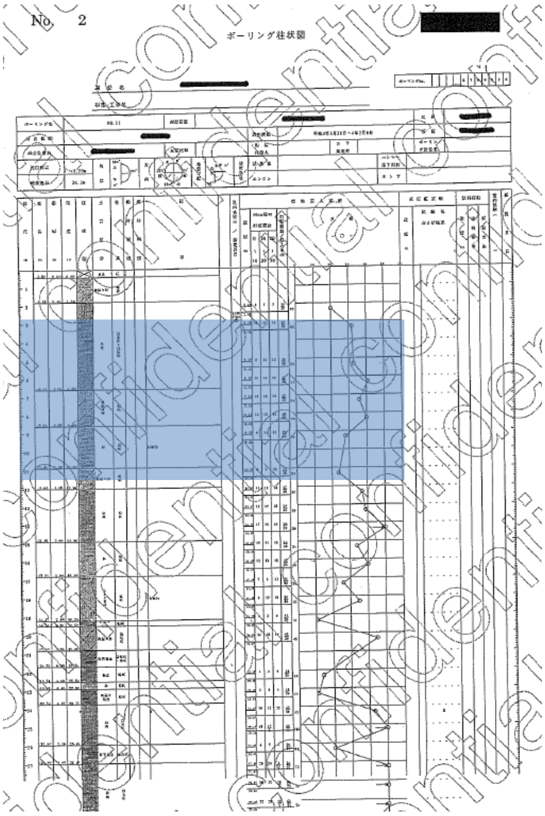

γ_m=120±3 pcf

γ_s=135±3 pcf

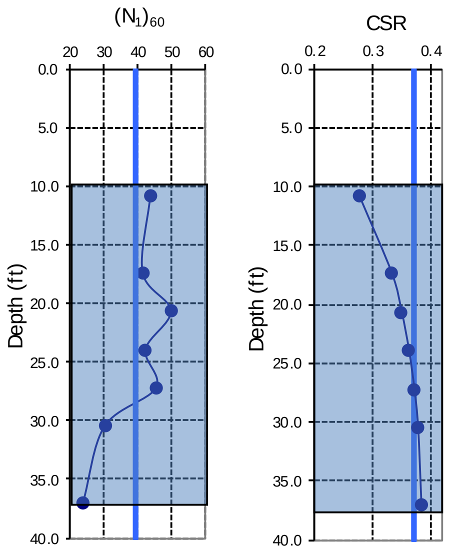

Depth (m)	Depth (ft)	CSR	N	C_N	C_R	$(N1)_{60}$	FC
3.3	10.8	0.277	32.0	1.31	0.86	***43.8***	
5.3	17.4	0.333	33.0	1.11	0.93	***41.5***	***8.0***
6.3	20.7	0.350	41.0	1.04	0.96	***49.8***	***0.0***
7.3	24.0	0.362	36.0	0.98	0.98	***42.4***	***7.0***
8.3	27.2	0.372	40.0	0.93	1.00	***45.4***	***30.0***
9.3	30.5	0.378	28.0	0.89	1.00	***30.4***	***22.0***
11.3	37.1	0.384	24.0	0.82	1.00	***24.0***	***22.0***
					**Mean:**	39.6	14.8
					**St. Dev.**	9.1	11.5

**Case number:**	161
**Earthquake:**	1995 Hypgoken-Nambu
**Magnitude:**	7.2 (M_L)
**Location:**	Kobe No : 3
**References:**	Kobe City Office, Construction Engineering Research Institute (1999)
**Nature of Failure:**	No surface manifestation of liquefaction
**Comments:**	

**Summary of Data**	Cetin et al. (2016)	Idriss&Boulanger (2010)	Seed et.al. (1984)		Cetin et al. (2016)	Idriss&Boulanger (2010)	Seed et.al. (1984)
Liquefied?	No	No		D_{50}	0.000 ± 0.000		
Data Class	B			% Fines	3.3 ± 2.2	3.0	
Critical Depth Range	11.5 - 24.6	19.0		% PI			
Depth to GWT (ft)	8.2 ± 0.3	8.2					
σ_v (psf)	2149.0 ± 298.0	2276.5		N	39.5 ± 5.5	40.0	
σ_v' (psf)	1534.8 ± 163.6	1608.2		C_R	0.94	0.95	
a_{max} (g)	0.400 ± 0.080	0.40		C_S	1.00	1.00	
r_d	0.99 ± 0.076	0.93		C_B	1.00	1.00	
CSR	0.362 ± 0.078	0.344		C_E	1.22	1.22	
Equivalent Magnitude	6.9	6.9		C_N	1.17	1.08	
MSF		1.17		$(N_1)_{60}$	52.8 ± 7.4	49.8	
CSRN		0.271					

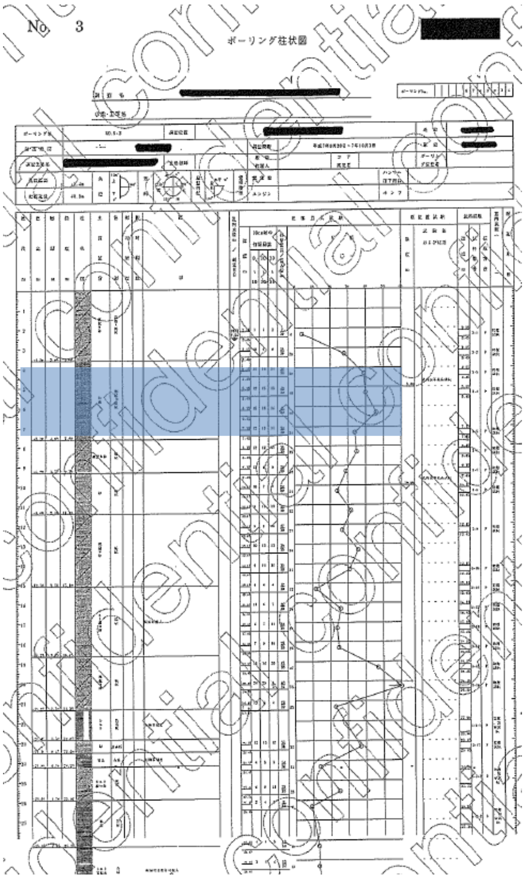

γ_m=100±3 pcf

γ_s=135±3 pcf

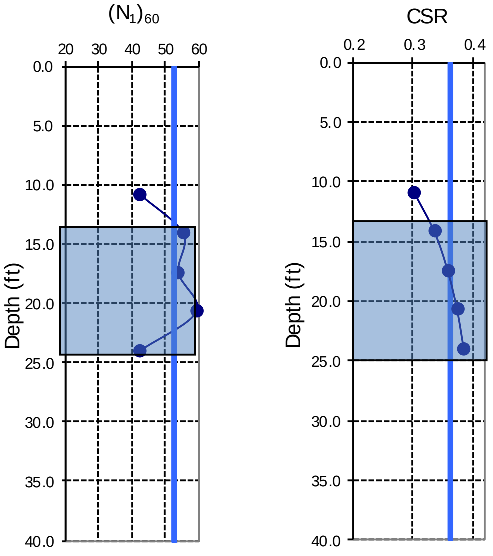

Depth (m)	Depth (ft)	CSR	N	C_N	C_R	$(N1)_{60}$	FC
3.3	10.8	0.301	28.0	1.45	0.86	42.4	
4.3	14.1	0.335	39.0	1.30	0.90	***55.4***	***5.0***
5.3	17.4	0.358	40.0	1.19	0.93	***54.0***	***4.0***
6.3	20.7	0.374	46.0	1.11	0.96	***59.4***	***0.0***
7.3	24.0	0.385	34.0	1.04	0.98	***42.3***	***4.0***
					**Mean:**	52.8	3.3
					**St. Dev.**	7.4	2.2

**Case number:**	162
**Earthquake:**	1995 Hyogoken-Nambu
**Magnitude:**	7.2 (M_L)
**Location:**	Kobe No : 4
**References:**	Kobe City Office, Construction Engineering Research Institute (1999)
**Nature of Failure:**	No surface manifestation of liquefaction
**Comments:**	

Summary of Data

	Cetin et al. (2016)	Idriss&Boulanger (2010)	Seed et.al. (1984)		Cetin et al. (2016)	Idriss&Boulanger (2010)	Seed et.al. (1984)
Liquefied?	No	No		D_{50}	0.000 ± 0.000		
Data Class	B			% Fines	1.3 ± 2.3	1.0	
Critical Depth Range	9.8 - 18.0	14.1		% PI			
Depth to GWT (ft)	6.7 ± 0.3	6.9					
σ_v (psf)	1642.1 ± 173.5	1587.3		N	27.8 ± 3.0	25.8	
σ_v' (psf)	1191.7 ± 91.7	1127.8		C_R	0.90	0.95	
a_{max} (g)	0.400 ± 0.080	0.40		C_S	1.00	1.00	
r_d	0.99 ± 0.061	0.95		C_B	1.00	1.00	
CSR	0.354 ± 0.075	0.350		C_E	1.22	1.22	
Equivalent Magnitude	6.9	6.9		C_N	1.33	1.23	
MSF		1.17		$(N_1)_{60}$	40.4 ± 4.4	36.6	
CSRN		0.272					

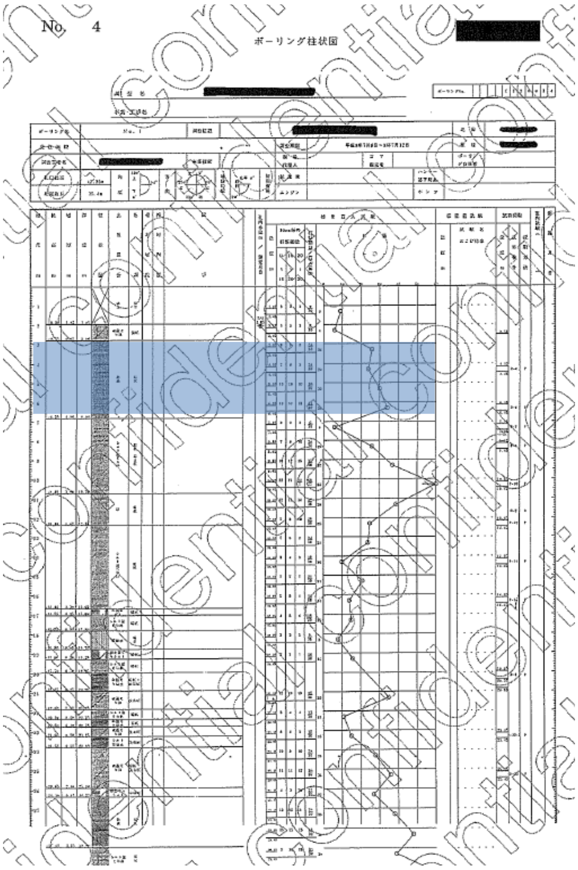

γ_m=110±3 pcf

γ_s=125±3 pcf

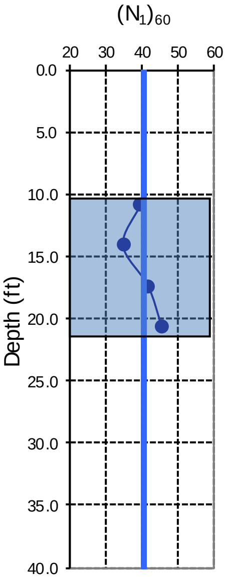

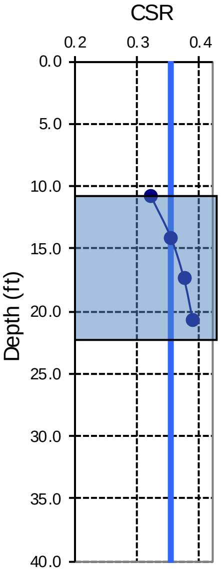

Depth (m)	Depth (ft)	CSR	N	C_N	C_R	$(N1)_{60}$	FC
3.3	10.8	0.324	26.0	1.46	0.86	*39.6*	*0.0*
4.3	14.1	0.355	24.0	1.33	0.90	*34.8*	*4.0*
5.3	17.4	0.375	30.0	1.23	0.93	*41.6*	*0.0*
6.3	20.7	0.389	34.0	1.15	0.96	*45.4*	
					**Mean:**	40.4	1.3
					**St. Dev.**	4.4	2.3

**Case number:**	163
**Earthquake:**	1995 Hyogoken-Nambu
**Magnitude:**	7.2 (M_L)
**Location:**	Kobe No : 5
**References:**	Kobe City Office, Construction Engineering Research Institute (1999)
**Nature of Failure:**	Surface manifestation of liquefaction
**Comments:**	

Summary of Data

	Cetin et al. (2016)	Idriss&Boulanger (2010)	Seed et.al. (1984)		Cetin et al. (2016)	Idriss&Boulanger (2010)	Seed et.al. (1984)
Liquefied?	Yes	Yes		D_{50}	0.000 ± 0.000		
Data Class	B			% Fines	1.3 ± 2.5	1.0	
Critical Depth Range	21.3 - 36.1	29.2		% PI			
Depth to GWT (ft)	9.9 ± 0.3	9.8					
σ_v (psf)	3345.8 ± 302.1	3613.2		N	5.8 ± 1.3	5.4	
σ_v' (psf)	2172.7 ± 156.2	2422.7		C_R	1.00	1.00	
a_{max} (g)	0.350 ± 0.070	0.35		C_S	1.00	1.00	
r_d	0.70 ± 0.113	0.88		C_B	1.00	1.00	
CSR	0.244 ± 0.063	0.298		C_E	1.22	1.22	
Equivalent Magnitude	6.9	6.9		C_N	0.99	0.92	
MSF		1.17		$(N_1)_{60}$	7.0 ± 1.6	6.1	
CSRN		0.258					

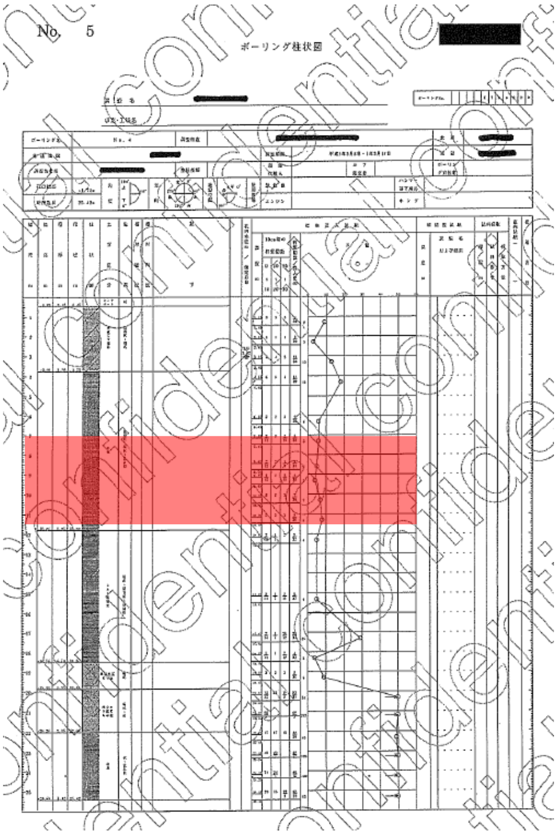

γ_m=110±3 pcf

γ_s=120±3 pcf

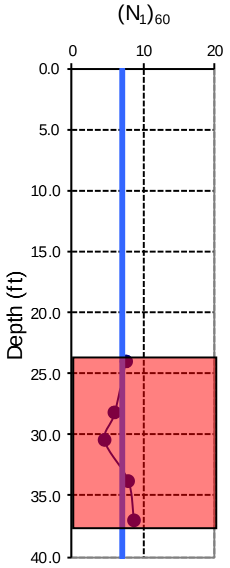

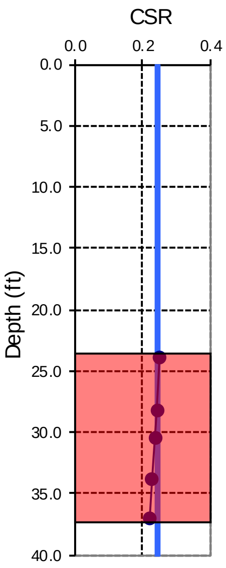

Depth (m)	Depth (ft)	CSR	N	C_N	C_R	$(N1)_{60}$	FC
7.3	24.0	0.254	6.0	1.06	0.98	***7.6***	***0.0***
8.6	28.2	0.245	5.0	0.99	1.00	***6.0***	***0.0***
9.3	30.5	0.239	4.0	0.96	1.00	***4.7***	***0.0***
10.3	33.8	0.231	7.0	0.93	1.00	***7.9***	***5.0***
11.3	37.1	0.223	8.0	0.89	1.00	***8.7***	
					**Mean:**	7.0	1.3
					**St. Dev.**	1.6	2.5

**Case number:**	164
**Earthquake:**	1995 Hyogoken-Nambu
**Magnitude:**	7.2 (M_L)
**Location:**	Kobe No : 6
**References:**	Kobe City Office, Construction Engineering Research Institute (1999)
**Nature of Failure:**	No surface manifestation of liquefaction.
**Comments:**	

**Summary of Data**	Cetin et al. (2016)	Idriss&Boulanger (2010)	Seed et.al. (1984)		Cetin et al. (2016)	Idriss&Boulanger (2010)	Seed et.al. (1984)
Liquefied?	No	No		D_{50}	0.000 ± 0.000		
Data Class	B			% Fines	24.7 ± 9.2	21.0	
Critical Depth Range	14.1 - 24.0	19.4		% PI			
Depth to GWT (ft)	7.5 ± 0.3	7.5					
σ_v (psf)	2340.9 ± 209.2	2234.7		N	16.7 ± 3.0	13.4	
σ_v' (psf)	1624.3 ± 112.0	1503.8		C_R	0.95	0.95	
a_{max} (g)	0.400 ± 0.080	0.40		C_S	1.00	1.00	
r_d	0.89 ± 0.079	0.93		C_B	1.00	1.00	
CSR	0.332 ± 0.073	0.360		C_E	1.22	1.22	
Equivalent Magnitude	6.9	6.9		C_N	1.14	1.15	
MSF		1.17		$(N_1)_{60}$	21.9 ± 3.9	17.8	
CSRN		0.293					

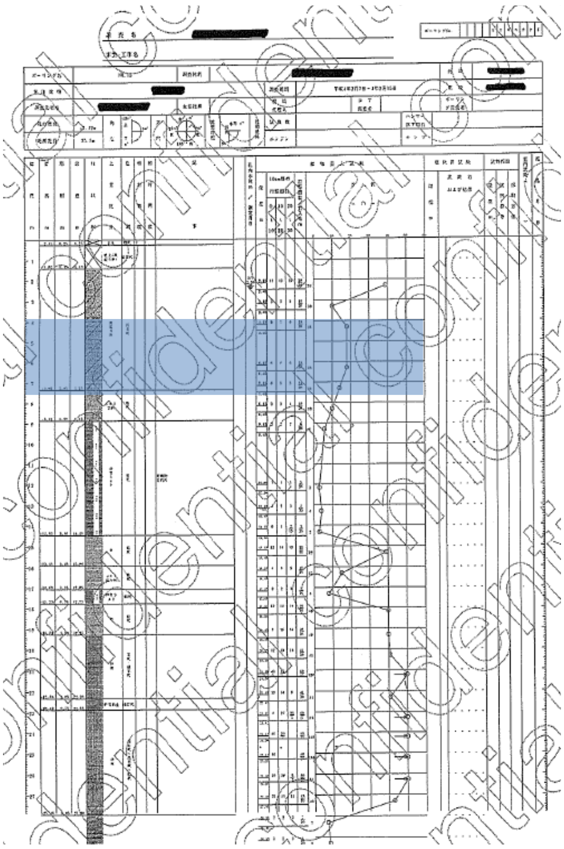

γ_m=120±3 pcf

γ_s=125±3 pcf

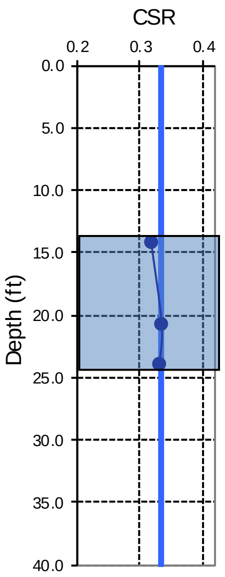

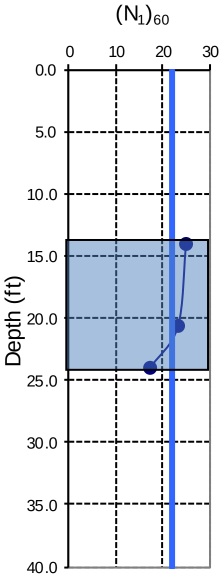

Depth (m)	Depth (ft)	CSR	N	C_N	C_R	$(N1)_{60}$	FC
4.3	14.1	0.318	18.0	1.27	0.90	*24.9*	*30.0*
6.3	20.7	0.333	18.0	1.11	0.96	*23.2*	*14.0*
7.3	24.0	0.330	14.0	1.05	0.98	*17.5*	*30.0*
					**Mean:**	21.9	24.7
					**St. Dev.**	3.9	9.2

**Case number:**	165
**Earthquake:**	1995 Hyogoken-Nambu
**Magnitude:**	7.2 (M_L)
**Location:**	Kobe No : 7
**References:**	Kobe City Office, Construction Engineering Research Institute (1999)
**Nature of Failure:**	Surface manifestation of liquefaction
**Comments:**	

Summary of Data

	Cetin et al. (2016)	Idriss&Boulanger (2010)	Seed et.al. (1984)		Cetin et al. (2016)	Idriss&Boulanger (2010)	Seed et.al. (1984)
Liquefied?	Yes	Yes		D_{50}	0.000 ± 0.000		
Data Class	B			% Fines	0.0 ± 2.0	0.0	
Critical Depth Range	5.9 - 12.5	10.8		% PI			
Depth to GWT (ft)	10.4 ± 0.3	10.5					
σ_v (psf)	998.7 ± 134.9	1294.9		N	15.6 ± 5.5	8.0	
σ_v' (psf)	1072.4 ± 72.1	1253.1		C_R	0.84	0.85	
a_{max} (g)	0.400 ± 0.080	0.40		C_S	1.00	1.00	
r_d	0.99 ± 0.043	0.93		C_B	1.00	1.00	
CSR	0.239 ± 0.052	0.314		C_E	1.22	1.22	
Equivalent Magnitude	6.9	6.9		C_N	1.40	1.32	
MSF		1.17		$(N_1)_{60}$	22.3 ± 7.9	10.9	
CSRN		0.262					

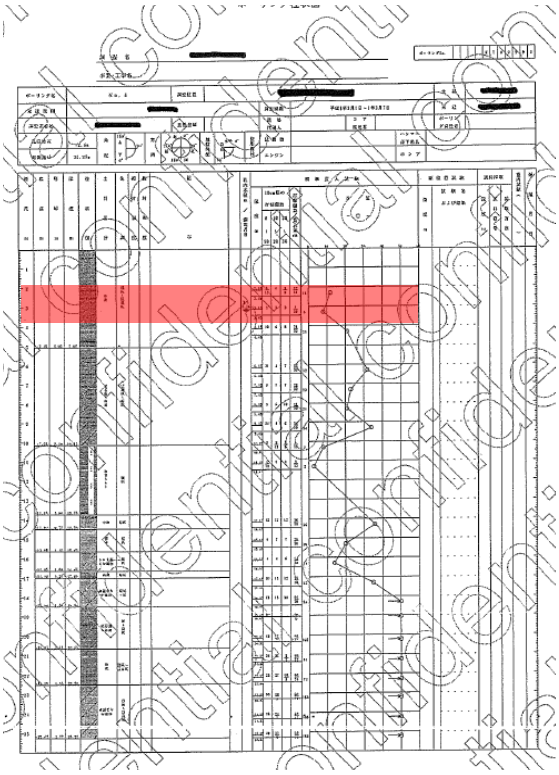

γ_m=110±3 pcf

γ_s=120±3pcf

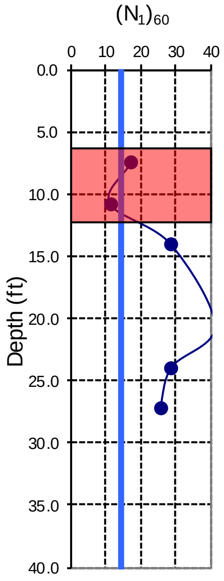

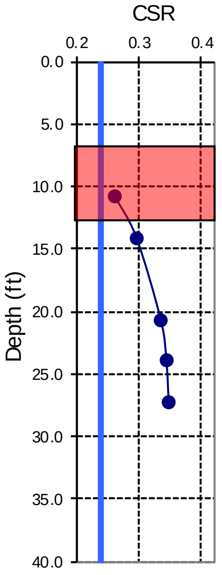

Depth (m)	Depth (ft)	CSR	N	C_N	C_R	$(N1)_{60}$	FC	
2.3	7.5	NA	11.0	1.60	0.81	**17.4**	**0.0**	interpreted
3.3	10.8	0.262	8.0	1.35	0.86	**11.3**	**0.0**	interpreted
4.3	14.1	0.296	21.0	1.25	0.90	28.7	0.0	
6.3	20.7	0.335	32.0	1.10	0.96	41.2	12.0	
7.3	24.0	0.344	23.0	1.05	0.98	28.9	0.0	
8.3	27.2	0.348	21.0	1.00	1.00	25.6	0.0	
					**Mean:**	14.3	0.0	
					**St. Dev.**	4.3	2.0	assigned

**Case number:**	166
**Earthquake:**	1995 Hyogoken-Nambu
**Magnitude:**	7.2 (M_L)
**Location:**	Kobe No : 8
**References:**	Tokimatsu, K. Personal Communication (2000)
**Nature of Failure:**	Surface manifestation of liquefaction
**Comments:**	

Summary of Data

	Cetin et al. (2016)	Idriss&Boulanger (2010)	Seed et.al. (1984)		Cetin et al. (2016)	Idriss&Boulanger (2010)	Seed et.al. (1984)
Liquefied?	Yes	Yes		D_{50}	0.000 ± 0.000		
Data Class	B			% Fines	0.0 ± 2.0	0.0	
Critical Depth Range	13.1 - 19.7	16.4		% PI			
Depth to GWT (ft)	9.7 ± 0.3	9.8					
σ_v (psf)	1808.6 ± 141.4	1775.3		N	17.4 ± 2.0	17.4	
σ_v' (psf)	1388.9 ± 77.9	1357.6		C_R	0.92	0.95	
a_{max} (g)	0.500 ± 0.100	0.50		C_S	1.00	1.00	
r_d	0.96 ± 0.070	0.94		C_B	1.00	1.00	
CSR	0.407 ± 0.087	0.402		C_E	1.22	1.22	
Equivalent Magnitude	6.9	6.9		C_N	1.23	1.20	
MSF		1.17		$(N_1)_{60}$	24.0 ± 2.8	24.1	
CSRN		0.321					

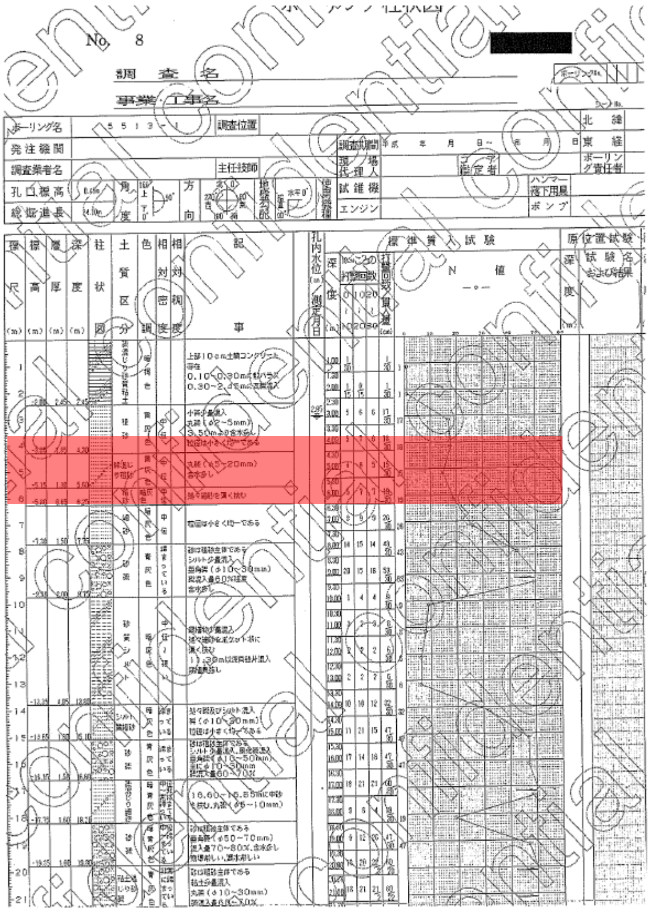

γ_m=100±3 pcf

γ_s=125±3 pcf

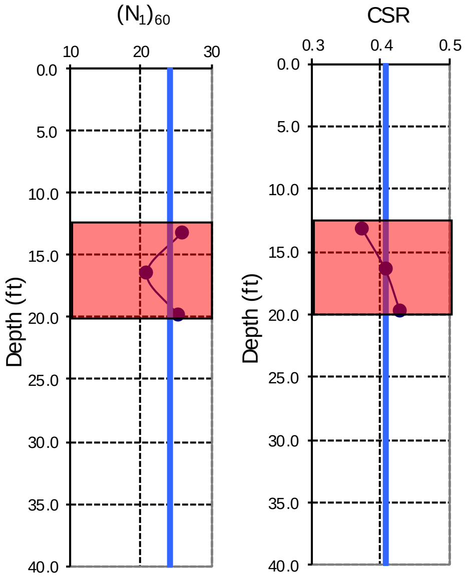

Depth (m)	Depth (ft)	CSR	N	C_N	C_R	$(N1)_{60}$	FC	
4.0	13.1	0.374	18.0	1.34	0.89	***26.0***	***0.0***	
5.0	16.4	0.407	15.0	1.23	0.92	***20.7***	***0.0***	
6.0	19.7	0.427	19.0	1.15	0.95	***25.3***	***0.0***	
					**Mean:**	24.0	0.0	
					**St. Dev.**	2.8	2.0	assigned

**Case number:**	167
**Earthquake:**	1995 Hyogoken-Nambu
**Magnitude:**	7.2 (M_L)
**Location:**	Kobe No : 9
**References:**	Kobe City Office, Construction Engineering Research Institute (1999)
**Nature of Failure:**	No surface manifestation of liquefaction
**Comments:**	

Summary of Data

	Cetin et al. (2016)	Idriss&Boulanger (2010)	Seed et.al. (1984)		Cetin et al. (2016)	Idriss&Boulanger (2010)	Seed et.al. (1984)
Liquefied?	Yes	Yes		D_{50}	0.000 ± 0.000		
Data Class	B			% Fines	2.3 ± 4.0	2.0	
Critical Depth Range	10.8 - 17.4	14.1		% PI			
Depth to GWT (ft)	9.1 ± 0.3	9.2					
σ_v (psf)	1531.5 ± 129.6	1649.9		N	8.6 ± 3.8	8.3	
σ_v' (psf)	1218.3 ± 67.3	1336.7		C_R	0.90	0.95	
a_{max} (g)	0.500 ± 0.100	0.50		C_S	1.00	1.00	
r_d	0.89 ± 0.061	0.95		C_B	1.00	1.00	
CSR	0.364 ± 0.078	0.383		C_E	1.22	1.22	
Equivalent Magnitude	6.9	6.9		C_N	1.32	1.27	
MSF		1.17		$(N_1)_{60}$	12.4 ± 5.5	12.2	
CSRN		0.313					

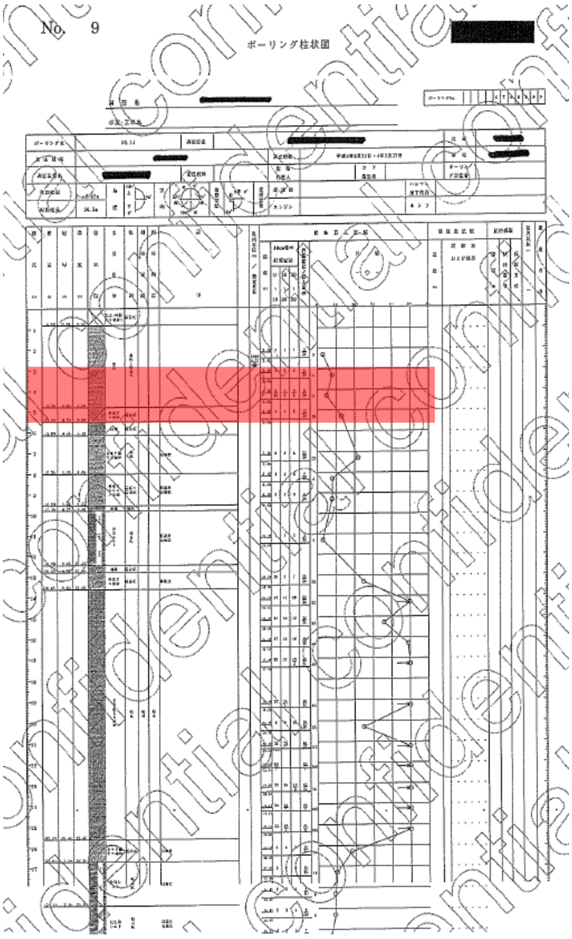

γ_m=105±3 pcf

γ_s=115±3 pcf

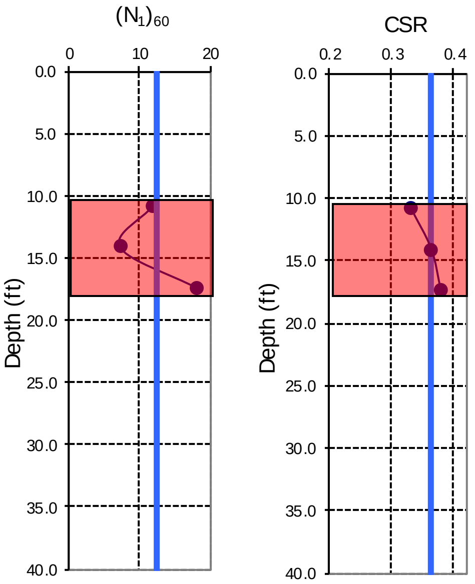

Depth (m)	Depth (ft)	CSR	N	C_N	C_R	$(N1)_{60}$	FC
3.3	10.8	0.332	8.0	1.42	0.86	***11.9***	***7.0***
4.3	14.1	0.364	5.0	1.32	0.90	***7.2***	***0.0***
5.3	17.4	0.378	13.0	1.23	0.93	***18.2***	***0.0***
					**Mean:**	12.4	2.3
					**St. Dev.**	5.5	4.0

**Case number:**	168
**Earthquake:**	1995 Hyogoken-Nambu
**Magnitude:**	7.2 (M_L)
**Location:**	Kobe No : 10
**References:**	Tokimatsu, K. Personal Communication (2000)
**Nature of Failure:**	No surface manifestation of liquefaction
**Comments:**	

Summary of Data

	Cetin et al. (2016)	Idriss&Boulanger (2010)	Seed et.al. (1984)		Cetin et al. (2016)	Idriss&Boulanger (2010)	Seed et.al. (1984)
Liquefied?	No	No		D_{50}	0.000 ± 0.000		
Data Class	B			% Fines	8.8 ± 2.5	9.0	
Critical Depth Range	19.7 - 29.5	24.6		% PI			
Depth to GWT (ft)	14.6 ± 0.3	14.8					
σ_v (psf)	3002.6 ± 211.8	2861.3		N	23.1 ± 3.5	24.1	
σ_v' (psf)	2380.3 ± 116.9	2234.7		C_R	0.99	0.95	
a_{max} (g)	0.600 ± 0.120	0.60		C_S	1.00	1.00	
r_d	0.92 ± 0.099	0.90		C_B	1.00	1.00	
CSR	0.455 ± 0.104	0.450		C_E	1.22	1.22	
Equivalent Magnitude	6.9	6.9		C_N	0.94	0.98	
MSF		1.17		$(N_1)_{60}$	26.2 ± 4.0	27.4	
CSRN		0.388					

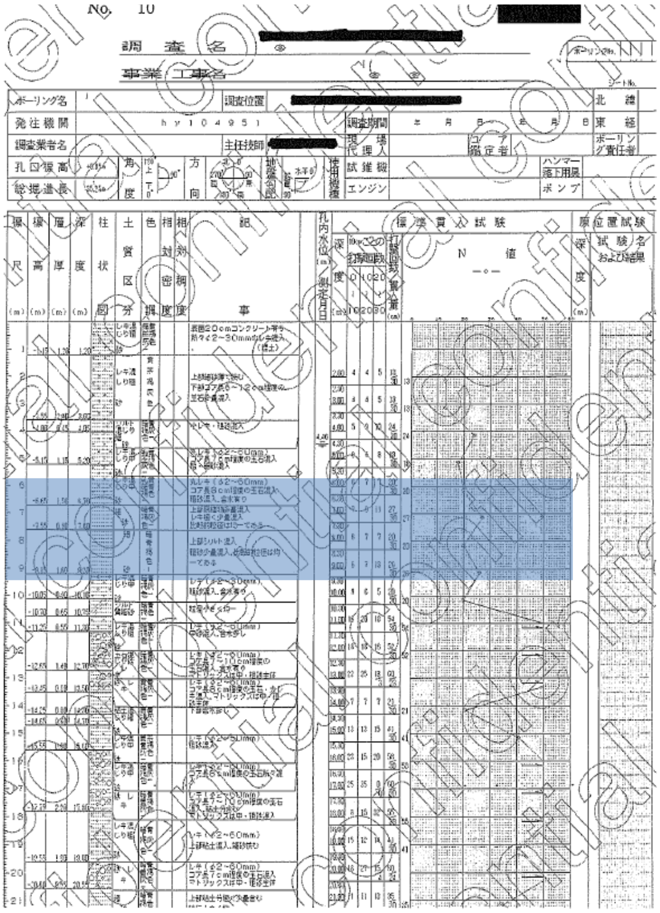
No. 10
調 査 名 [redacted]
事業・工事名
ボーリング名 | 調査位置 [redacted]
発注機関 | 調査期間
調査業者名 | 主任技師 [redacted] | 現場代理人
孔口標高 | 試錐機
総掘進長 | エンジン
標準貫入試験
N値
原位置試験

γ_m=120±3 pcf

γ_s=125±3 pcf

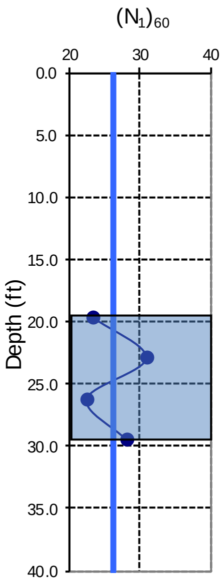

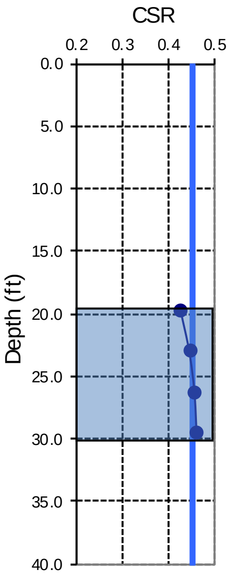

Depth (m)	Depth (ft)	CSR	N	C_N	C_R	$(N1)_{60}$	FC
6.0	19.7	0.428	20.0	1.01	0.95	*23.4*	*5.0*
7.0	23.0	0.448	27.0	0.96	0.98	*30.9*	*10.0*
8.0	26.2	0.460	20.0	0.92	1.00	*22.5*	*10.0*
9.0	29.5	0.465	26.0	0.89	1.00	*28.1*	*10.0*
					**Mean:**	26.2	8.8
					**St. Dev.**	4.0	2.5

**Case number:**	169
**Earthquake:**	1995 Hyogoken-Nambu
**Magnitude:**	7.2 (M_L)
**Location:**	Kobe No : 11
**References:**	Tokimatsu, K. Personal Communication (2000)
**Nature of Failure:**	Liquefied
**Comments:**	

Summary of Data

	Cetin et al. (2016)	Idriss&Boulanger (2010)	Seed et.al. (1984)		Cetin et al. (2016)	Idriss&Boulanger (2010)	Seed et.al. (1984)
Liquefied?	Yes	Yes		D_{50}	0.000 ± 0.000		
Data Class	B			% Fines	5.0 ± 2.0	5.0	
Critical Depth Range	12.3 - 32.0	22.3		% PI			
Depth to GWT (ft)	4.8 ± 0.3	4.9					
σ_v (psf)	2609.9 ± 397.4	2380.9		N	5.6 ± 1.6	5.6	
σ_v' (psf)	1524.9 ± 197.2	1294.9		C_R	0.97	0.95	
a_{max} (g)	0.500 ± 0.100	0.50		C_S	1.00	1.00	
r_d	0.67 ± 0.090	0.91		C_B	1.00	1.00	
CSR	0.374 ± 0.091	0.546		C_E	1.22	1.22	
Equivalent Magnitude	6.9	6.9		C_N	1.18	1.31	
MSF		1.17		$(N_1)_{60}$	7.7 ± 2.2	8.5	
CSRN		0.447					

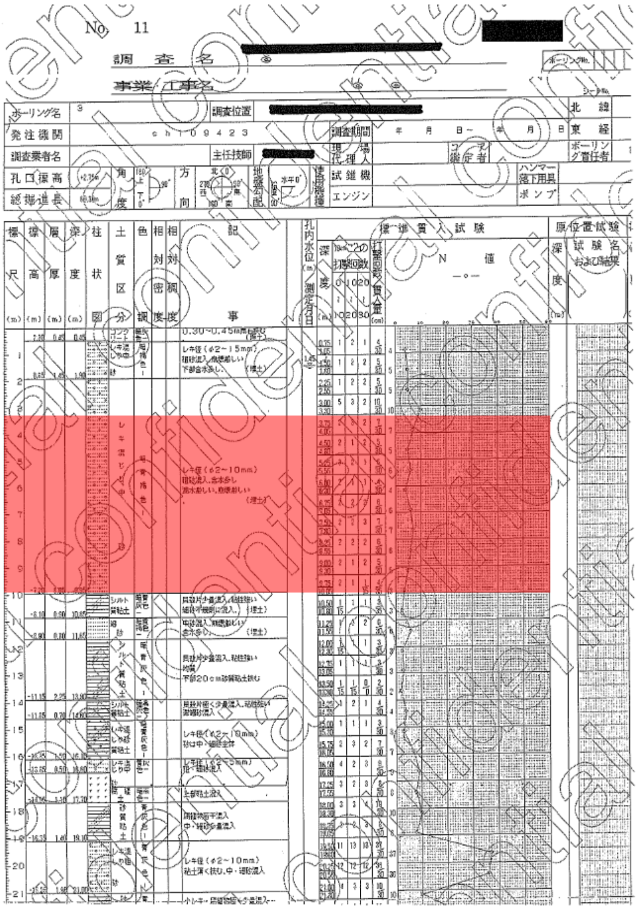

γ_m=110±3 pcf

γ_s=120±3 pcf

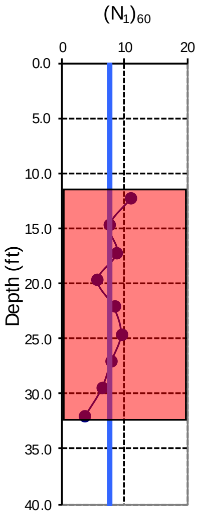

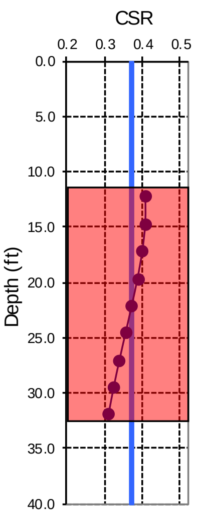

Depth (m)	Depth (ft)	CSR	N	C_N	C_R	$(N1)_{60}$	FC
3.8	12.3	0.410	7.0	1.49	0.88	***11.1***	***5.0***
4.5	14.8	0.410	5.0	1.39	0.90	***7.6***	***5.0***
5.3	17.2	0.402	6.0	1.31	0.93	***8.9***	***5.0***
6.0	19.7	0.389	4.0	1.24	0.95	***5.7***	***5.0***
6.8	22.1	0.374	6.0	1.18	0.97	***8.3***	***5.0***
7.5	24.6	0.357	7.0	1.13	0.99	***9.5***	***5.0***
8.3	27.1	0.341	6.0	1.08	1.00	***7.9***	***5.0***
9.0	29.5	0.325	5.0	1.04	1.00	***6.3***	***5.0***
9.8	32.0	0.311	3.0	1.01	1.00	***3.7***	***5.0***
					**Mean:**	7.7	5.0
					**St. Dev.**	2.2	2.0

assg

**Case number:**	170
**Earthquake:**	1995 Hyogoken-Nambu
**Magnitude:**	7.2 (M_L)
**Location:**	Kobe No : 12
**References:**	Kobe City Office, Construction Engineering Research Institute (1999)
**Nature of Failure:**	Liquefied
**Comments:**	

Summary of Data

	Cetin et al. (2016)	Idriss&Boulanger (2010)	Seed et.al. (1984)		Cetin et al. (2016)	Idriss&Boulanger (2010)	Seed et.al. (1984)
Liquefied?	No	No		D_{50}	0.000 ± 0.000		
Data Class	B			% Fines	14.0 ± 14.0	14.0	
Critical Depth Range	14.1 - 20.7	17.4		% PI			
Depth to GWT (ft)	10.5 ± 0.3	10.5					
σ_v (psf)	1911.1 ± 142.0	1921.5		N	19.6 ± 1.3	18.6	
σ_v' (psf)	1481.2 ± 78.9	1503.8		C_R	0.92	0.95	
a_{max} (g)	0.500 ± 0.100	0.50		C_S	1.00	1.00	
r_d	0.94 ± 0.073	0.94		C_B	1.00	1.00	
CSR	0.396 ± 0.086	0.393		C_E	1.22	1.22	
Equivalent Magnitude	6.9	6.9		C_N	1.20	1.14	
MSF		1.17		$(N_1)_{60}$	26.2 ± 1.7	24.7	
CSRN		0.316					

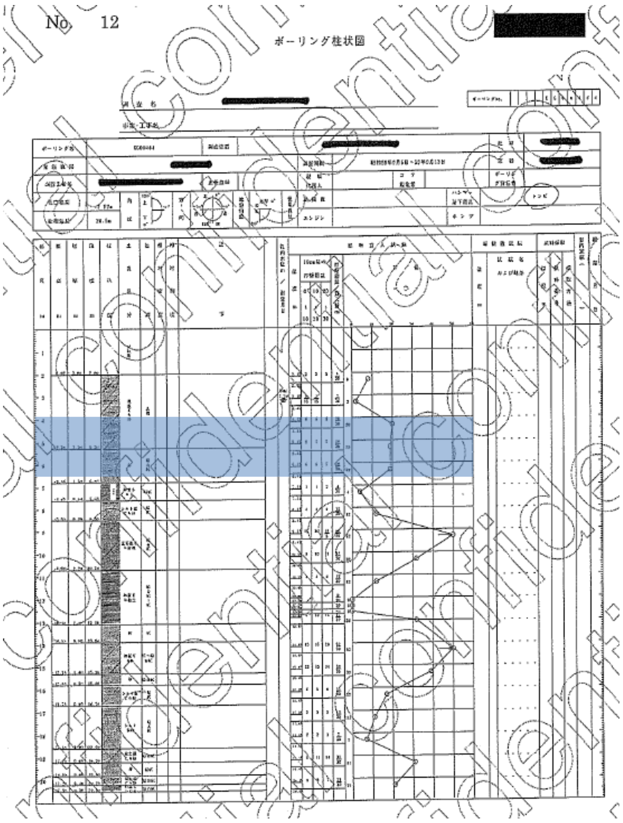

γ_m=100±3 pcf

γ_s=125±3 pcf

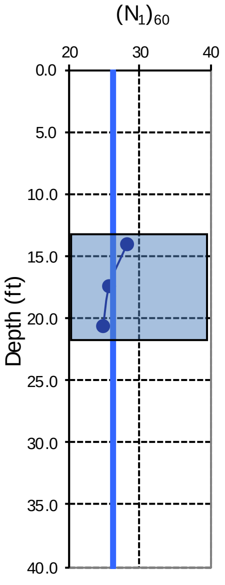

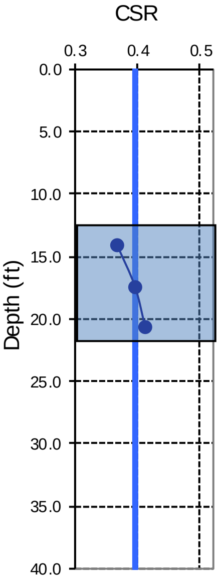

Depth (m)	Depth (ft)	CSR	N	C_N	C_R	$(N1)_{60}$	FC
4.3	14.1	0.368	20.0	1.29	0.90	***28.1***	***4.0***
5.3	17.4	0.396	19.0	1.20	0.93	***25.7***	***30.0***
6.3	20.7	0.413	19.0	1.12	0.96	***24.8***	***8.0***
					**Mean:**	26.2	14.0
					**St. Dev.**	1.7	14.0

**Case number:**	171
**Earthquake:**	1995 Hyogoken-Nambu
**Magnitude:**	7.2 (M_L)
**Location:**	Kobe No : 13
**References:**	Kobe City Office, 1999 (In Japanese), Confidential
**Nature of Failure:**	Liquefied
**Comments:**	

Summary of Data

	Cetin et al. (2016)	Idriss&Boulanger (2010)	Seed et.al. (1984)		Cetin et al. (2016)	Idriss&Boulanger (2010)	Seed et.al. (1984)
Liquefied?	Yes	Yes		D_{50}	0.000 ± 0.000		
Data Class	B			% Fines	15.0 ± 10.0	15.0	
Critical Depth Range	16.4 - 26.2	21.3		% PI			
Depth to GWT (ft)	7.5 ± 0.3	7.5					
σ_v (psf)	2596.8 ± 202.4	2422.7		N	9.3 ± 1.1	9.5	
σ_v' (psf)	1736.9 ± 107.5	1545.5		C_R	0.96	0.95	
a_{max} (g)	0.500 ± 0.100	0.50		C_S	1.00	1.00	
r_d	0.87 ± 0.087	0.92		C_B	1.00	1.00	
CSR	0.421 ± 0.095	0.464		C_E	1.22	1.22	
Equivalent Magnitude	6.9	6.9		C_N	1.10	1.16	
MSF		1.17		$(N_1)_{60}$	12.0 ± 1.4	12.7	
CSRN		0.383					

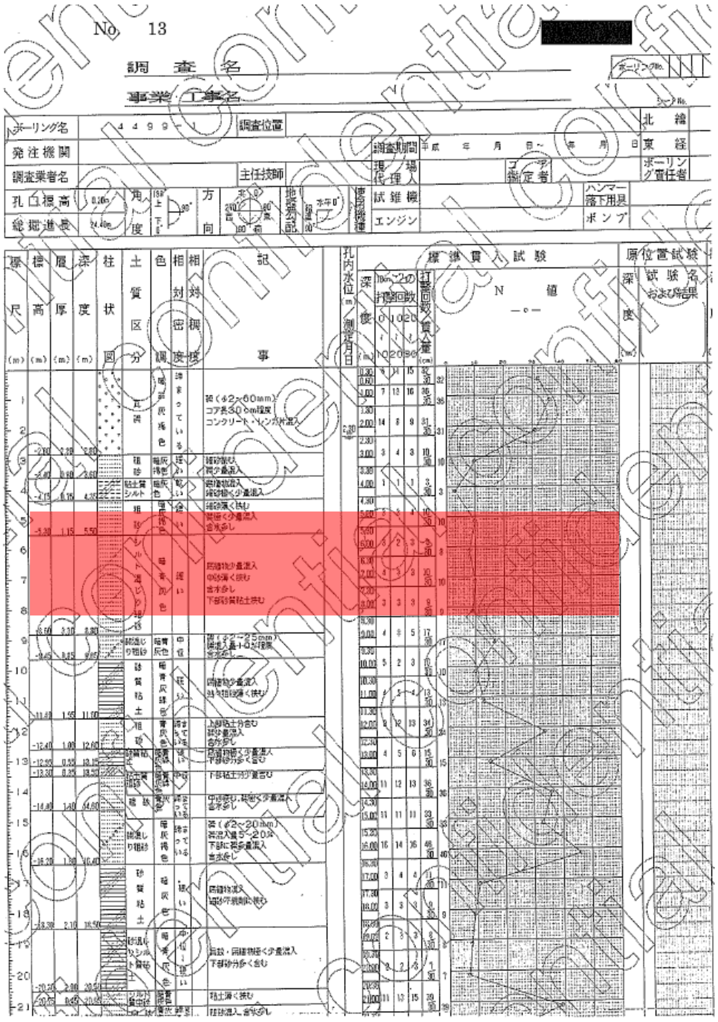

γ_m=125±3 pcf

γ_s=120±3 pcf

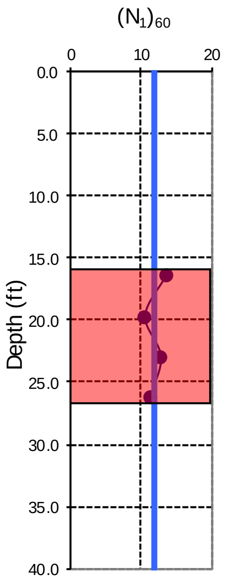

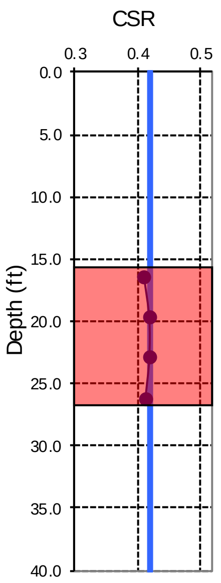

Depth (m)	Depth (ft)	CSR	N	C_N	C_R	$(N1)_{60}$	FC
5.0	16.4	0.411	10.0	1.21	0.92	***13.5***	***0.0***
6.0	19.7	0.420	8.0	1.14	0.95	***10.5***	***20.0***
7.0	23.0	0.420	10.0	1.07	0.98	***12.8***	***20.0***
8.0	26.2	0.413	9.0	1.02	1.00	***11.2***	***20.0***
					**Mean:**	12.0	15.0
					**St. Dev.**	1.4	10.0

**Case number:**	172
**Earthquake:**	1995 Hyogoken-Nambu
**Magnitude:**	7.2 (M_L)
**Location:**	Kobe No : 14
**References:**	Kobe City Office, Construction Engineering Research Institute (1999)
**Nature of Failure:**	Non-Liquefied
**Comments:**	

Summary of Data

	Cetin et al. (2016)	Idriss&Boulanger (2010)	Seed et.al. (1984)		Cetin et al. (2016)	Idriss&Boulanger (2010)	Seed et.al. (1984)
Liquefied?	No	No		D_{50}	0.000 ± 0.000		
Data Class	B			% Fines	18.5 ± 16.3	19.0	
Critical Depth Range	14.1 - 17.4	15.7		% PI			
Depth to GWT (ft)	10.2 ± 0.3	10.2					
σ_v (psf)	1866.8 ± 76.8	1796.1		N	16.0 ± 1.8	15.0	
σ_v' (psf)	1518.8 ± 51.3	1441.1		C_R	0.91	0.95	
a_{max} (g)	0.500 ± 0.100	0.50		C_S	1.00	1.00	
r_d	0.90 ± 0.068	0.95		C_B	1.00	1.00	
CSR	0.360 ± 0.077	0.382		C_E	1.22	1.22	
Equivalent Magnitude	6.9	6.9		C_N	1.18	1.17	
MSF		1.17		$(N_1)_{60}$	21.0 ± 2.3	20.3	
CSRN		0.307					

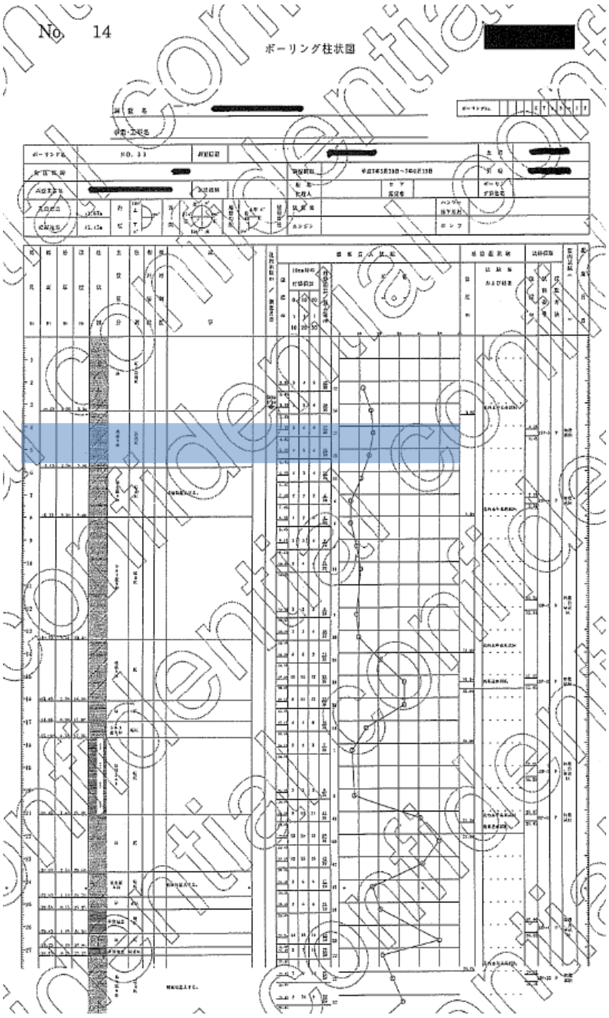

γ_m=115±3 pcf

γ_s=125±3 pcf

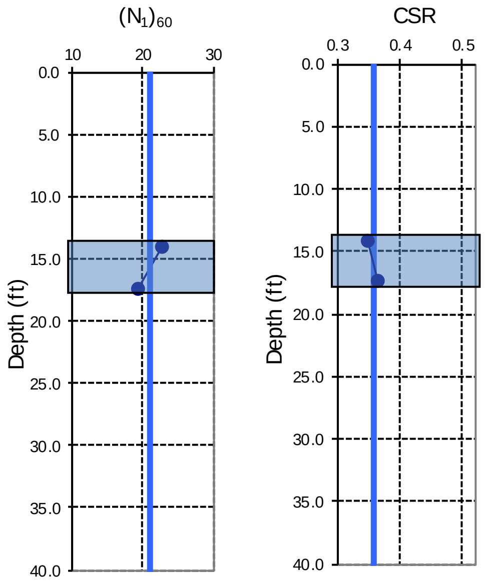

Depth (m)	Depth (ft)	CSR	N	C_N	C_R	$(N1)_{60}$	FC
4.3	14.1	0.350	17.0	1.22	0.90	*22.7*	*7.0*
5.3	17.4	0.366	15.0	1.14	0.93	*19.4*	*30.0*
					**Mean:**	21.0	18.5
					**St. Dev.**	2.3	16.3

**Case number:**	173
**Earthquake:**	1995 Hyogoken-Nambu
**Magnitude:**	7.2 (M_L)
**Location:**	Kobe No : 15
**References:**	Kobe City Office, Construction Engineering Research Institute (1999)
**Nature of Failure:**	Liquefied
**Comments:**	

Summary of Data

	Cetin et al. (2016)	Idriss&Boulanger (2010)	Seed et.al. (1984)		Cetin et al. (2016)	Idriss&Boulanger (2010)	Seed et.al. (1984)
Liquefied?	Yes	Yes		D_{50}	0.000 ± 0.000		
Data Class	B			% Fines	4.7 ± 4.5	5.0	
Critical Depth Range	15.3 - 22.6	18.7		% PI			
Depth to GWT (ft)	12.0 ± 0.3	12.1					
σ_v (psf)	2069.0 ± 159.5	2130.3		N	15.0 ± 3.1	15.1	
σ_v' (psf)	1633.9 ± 88.2	1712.6		C_R	0.94	0.95	
a_{max} (g)	0.500 ± 0.100	0.50		C_S	1.00	1.00	
r_d	0.86 ± 0.079	0.93		C_B	1.00	1.00	
CSR	0.356 ± 0.079	0.375		C_E	1.22	1.22	
Equivalent Magnitude	6.9	6.9		C_N	1.14	1.10	
MSF		1.17		$(N_1)_{60}$	19.6 ± 4.0	19.2	
CSRN		0.312					

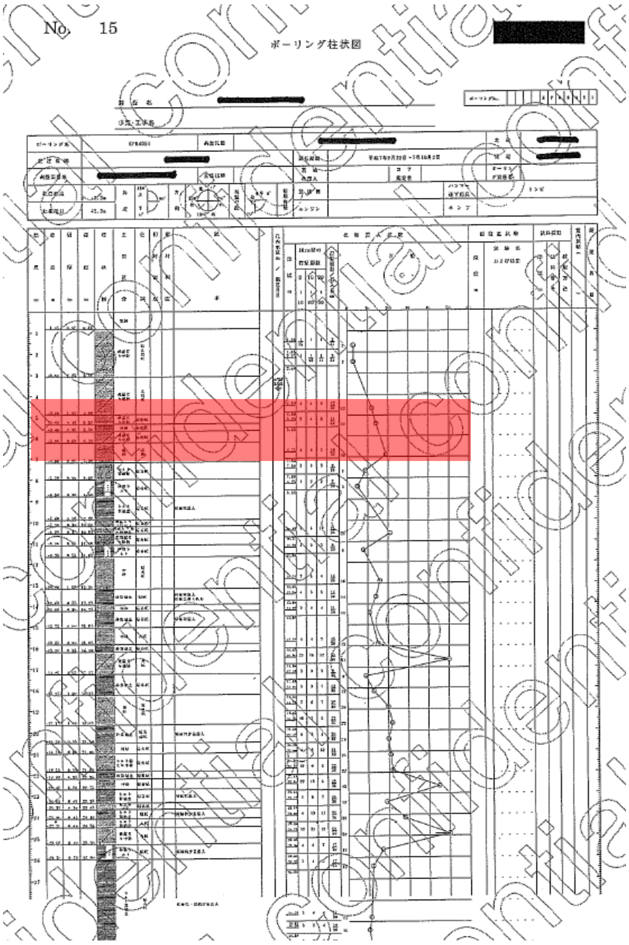

γ_m=100±3 pcf

γ_s=125±3 pcf

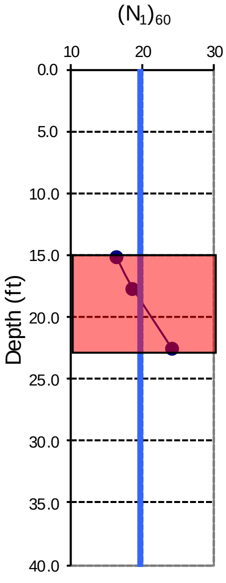

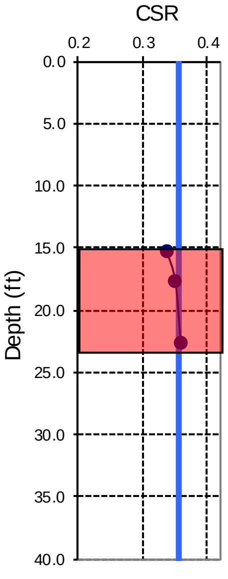

Depth (m)	Depth (ft)	CSR	N	C_N	C_R	$(N1)_{60}$	FC
4.7	15.3	0.337	12.0	1.23	0.91	***16.3***	***5.0***
5.4	17.7	0.351	14.0	1.17	0.93	***18.5***	***0.0***
6.9	22.6	0.360	19.0	1.07	0.97	***24.0***	***9.0***
					**Mean:**	19.6	4.7
					**St. Dev.**	4.0	4.5

**Case number:**	174
**Earthquake:**	1995 Hyogoken-Nambu
**Magnitude:**	7.2 (M_L)
**Location:**	Kobe No : 16
**References:**	Tokimatsu, K. Personal Communication (2000)
**Nature of Failure:**	Nonliquefied
**Comments:**	

Summary of Data

	Cetin et al. (2016)	Idriss&Boulanger (2010)	Seed et.al. (1984)		Cetin et al. (2016)	Idriss&Boulanger (2010)	Seed et.al. (1984)
Liquefied?	No	No		D_{50}	0.000 ± 0.000		
Data Class	B			% Fines	5.0 ± 2.0	5.0	
Critical Depth Range	13.1 - 16.4	14.8		% PI			
Depth to GWT (ft)	8.0 ± 0.3	8.2					
σ_v (psf)	1724.9 ± 75.4	1670.8		N	17.6 ± 1.2	17.5	
σ_v' (psf)	1305.2 ± 48.6	1253.1		C_R	0.90	0.95	
a_{max} (g)	0.600 ± 0.120	0.60		C_S	1.00	1.00	
r_d	0.95 ± 0.064	0.95		C_B	1.00	1.00	
CSR	0.489 ± 0.104	0.495		C_E	1.22	1.22	
Equivalent Magnitude	6.9	6.9		C_N	1.27	1.23	
MSF		1.17		$(N_1)_{60}$	24.6 ± 1.7	25.0	
CSRN		0.390					

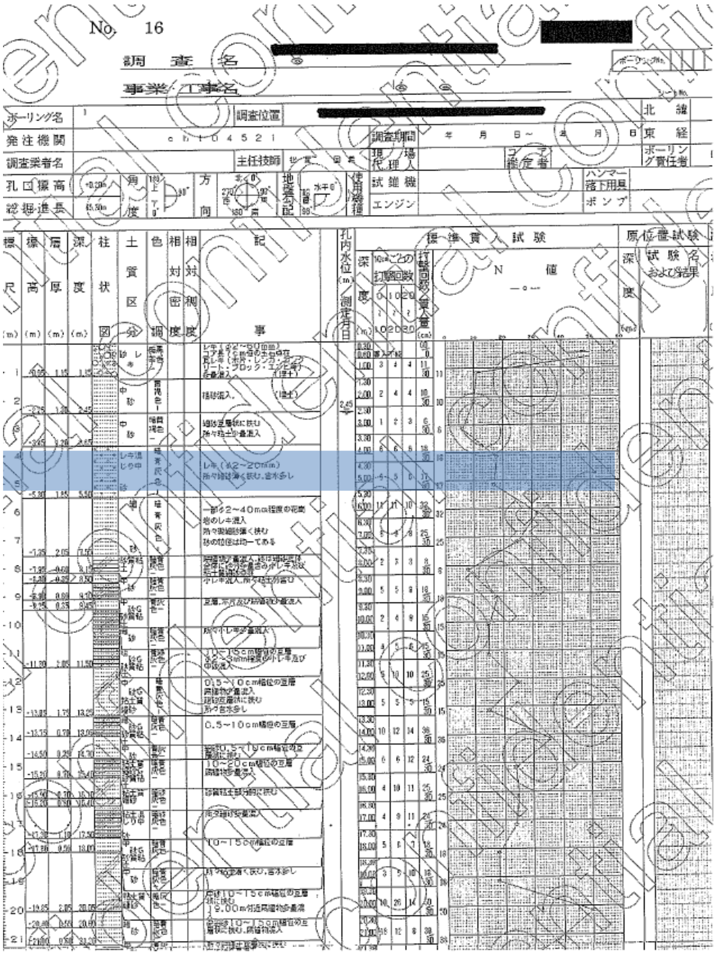

γ_m=110±3 pcf

γ_s=125±3 pcf

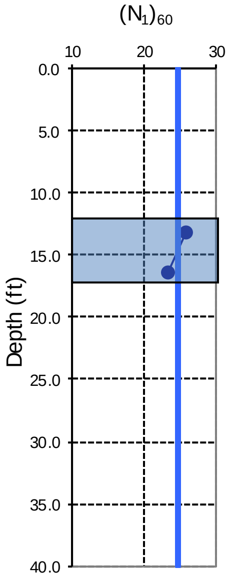

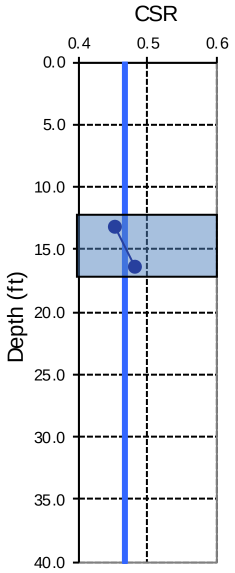

Depth (m)	Depth (ft)	CSR	N	C_N	C_R	$(N1)_{60}$	FC
4.0	13.1	0.472	18.0	1.33	0.89	*25.8*	*5.0*
5.0	16.4	0.502	17.0	1.23	0.92	*23.4*	*5.0*
					**Mean:**	24.6	5.0
					**St. Dev.**	1.7	2.0

assigned

**Case number:**	175
**Earthquake:**	1995 Hyogoken-Nambu
**Magnitude:**	7.2 (M_L)
**Location:**	Kobe No : 17
**References:**	Tokimatsu, K. Personal Communication (2000)
**Nature of Failure:**	Liquefied
**Comments:**	

**Summary of Data**	Cetin et al. (2016)	Idriss&Boulanger (2010)	Seed et.al. (1984)		Cetin et al. (2016)	Idriss&Boulanger (2010)	Seed et.al. (1984)
Liquefied?	Yes	Yes		D_{50}	0.000 ± 0.000		
Data Class	B			% Fines	5.0 ± 2.0	5.0	
Critical Depth Range	9.8 - 19.7	14.8		% PI			
Depth to GWT (ft)	2.5 ± 0.3	2.6					
σ_v (psf)	1747.0 ± 200.4	1670.8		N	13.1 ± 4.4	12.6	
σ_v' (psf)	979.3 ± 102.9	898.1		C_R	0.90	0.95	
a_{max} (g)	0.500 ± 0.100	0.50		C_S	1.00	1.00	
r_d	0.95 ± 0.064	0.95		C_B	1.00	1.00	
CSR	0.551 ± 0.117	0.574		C_E	1.22	1.22	
Equivalent Magnitude	6.9	6.9		C_N	1.47	1.45	
MSF		1.17		$(N_1)_{60}$	21.2 ± 7.1	21.1	
CSRN		0.446					

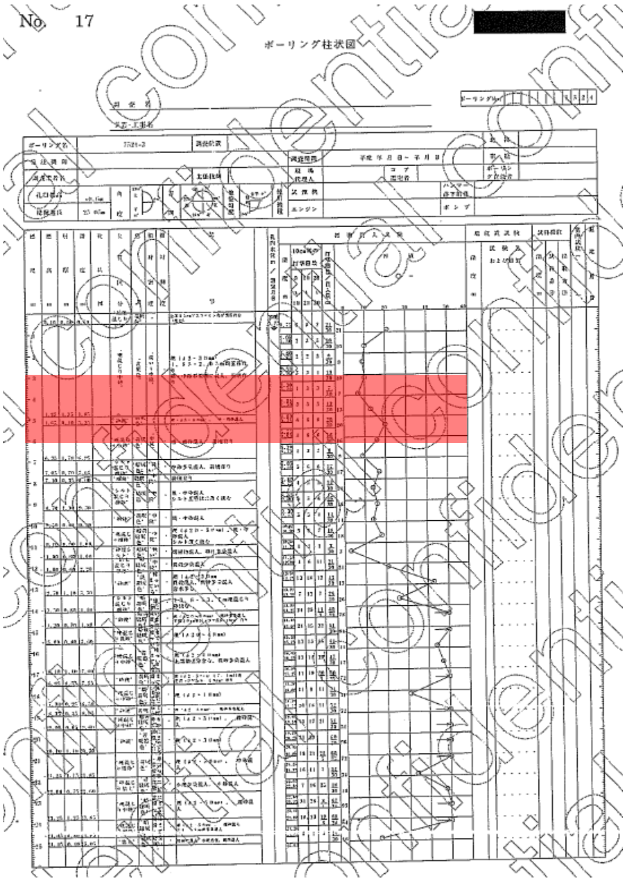

γ_m=110±3 pcf

γ_s=120±3 pcf

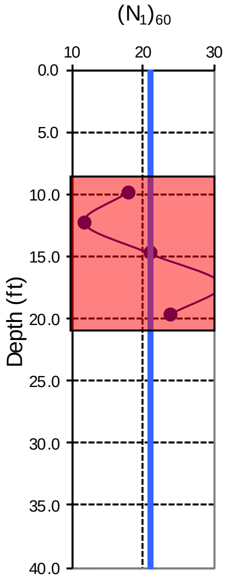

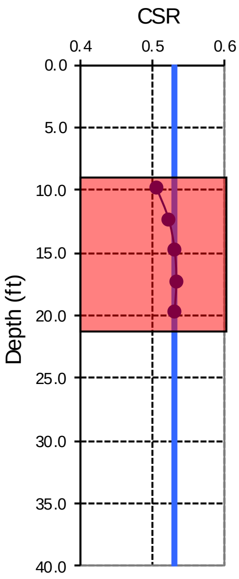

Depth (m)	Depth (ft)	CSR	N	C_N	C_R	$(N1)_{60}$	FC	
3.0	9.8	0.526	10.0	1.74	0.85	***18.0***	***5.0***	
3.8	12.3	0.543	7.0	1.59	0.88	***11.9***	***5.0***	
4.5	14.8	0.551	13.0	1.47	0.90	***21.0***	***5.0***	
5.3	17.2	0.554	20.0	1.37	0.93	***31.1***	***5.0***	
6.0	19.7	0.551	16.0	1.29	0.95	***24.0***	***5.0***	
					**Mean:**	21.2	5.0	
					**St. Dev.**	7.1	2.0	assigned

Case number: 176
Earthquake: 1995 Hyogoken-Nambu
Magnitude: 7.2 (M_L)
Location: Kobe No : 18
References: Tokimatsu, K. Personal Communication (2000)
Nature of Failure: Nonliquefied

Comments:

Summary of Data

	Cetin et al. (2016)	Idriss&Boulanger (2010)	Seed et.al. (1984)		Cetin et al. (2016)	Idriss&Boulanger (2010)	Seed et.al. (1984)
Liquefied?	No	No		D_{50}	0.000 ± 0.000		
Data Class	B			% Fines	0.0 ± 2.0	0.0	
Critical Depth Range	29.5 - 39.4	34.4		% PI			
Depth to GWT (ft)	25.1 ± 0.3	25.3					
σ_v (psf)	4399.6 ± 235.6	4156.2		N	40.5 ± 4.2	40.5	
σ_v' (psf)	3816.1 ± 144.5	3571.4		C_R	1.00	1.00	
a_{max} (g)	0.700 ± 0.140	0.70		C_S	1.00	1.00	
r_d	0.94 ± 0.131	0.85		C_B	1.00	1.00	
CSR	0.493 ± 0.121	0.448		C_E	1.22	1.22	
Equivalent Magnitude	6.9	6.9		C_N	0.74	0.86	
MSF		1.17		$(N_1)_{60}$	36.7 ± 3.8	42.6	
CSRN		0.452					

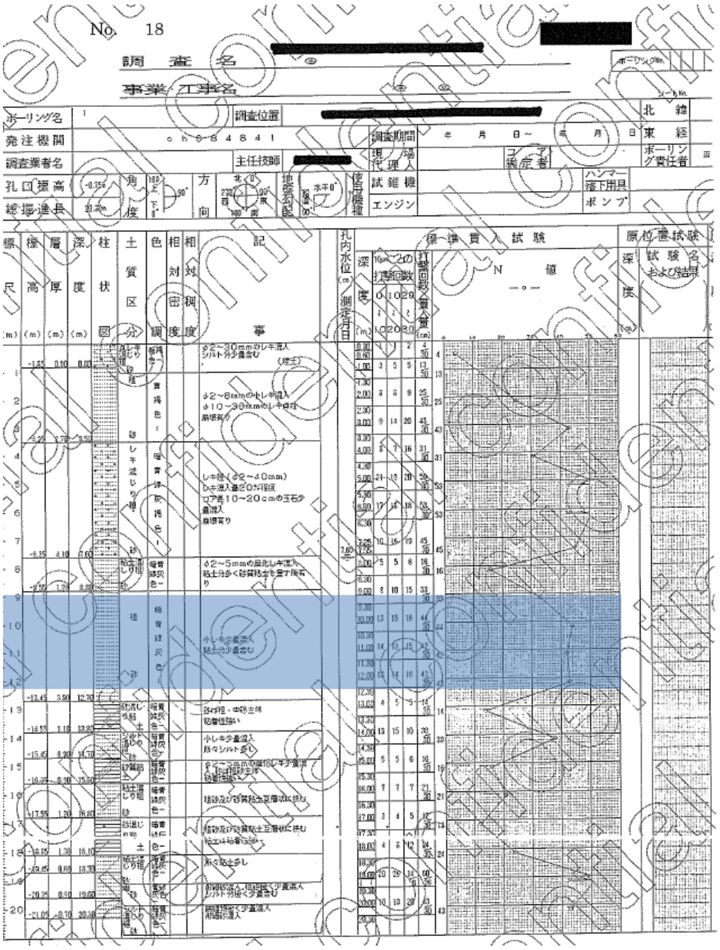

γ_m=125±3 pcf

γ_s=135±3 pcf

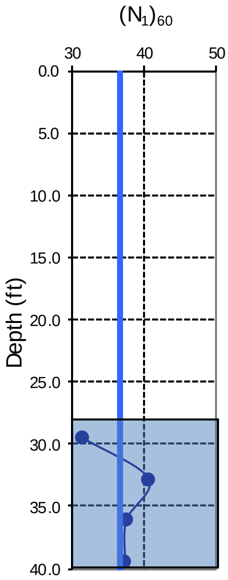

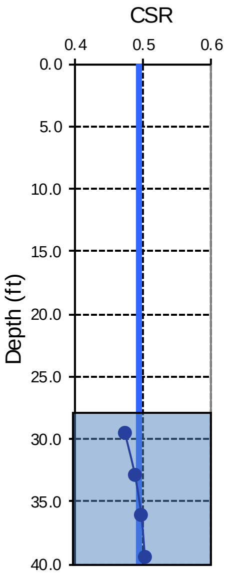

Depth (m)	Depth (ft)	CSR	N	C_N	C_R	$(N1)_{60}$	FC
9.0	29.5	0.473	33.0	0.78	1.00	*31.4*	*0.0*
10.0	32.8	0.488	44.0	0.76	1.00	*40.5*	*0.0*
11.0	36.1	0.498	42.0	0.73	1.00	*37.5*	*0.0*
12.0	39.4	0.503	43.0	0.71	1.00	*37.3*	*0.0*
					**Mean:**	36.7	0.0
					**St. Dev.**	3.8	2.0

**Case number:**	177
**Earthquake:**	1995 Hyogoken-Nambu
**Magnitude:**	7.2 (M_L)
**Location:**	Kobe No : 19
**References:**	Tokimatsu, K. Personal Communication (2000)
**Nature of Failure:**	Nonliquefied
**Comments:**	

**Summary of Data**	Cetin et al. (2016)	Idriss&Boulanger (2010)	Seed et.al. (1984)		Cetin et al. (2016)	Idriss&Boulanger (2010)	Seed et.al. (1984)
Liquefied?	No	No		D_{50}	0.000 ± 0.000		
Data Class	B			% Fines	10.0 ± 2.0	10.0	
Critical Depth Range	23.0 - 26.2	24.6		% PI			
Depth to GWT (ft)	20.0 ± 0.3	20.0					
σ_v (psf)	2975.7 ± 92.0	2861.3		N	19.5 ± 0.9	20.0	
σ_v' (psf)	2689.1 ± 72.5	2589.8		C_R	0.99	0.95	
a_{max} (g)	0.600 ± 0.120	0.60		C_S	1.00	1.00	
r_d	0.95 ± 0.099	0.90		C_B	1.00	1.00	
CSR	0.411 ± 0.093	0.391		C_E	1.22	1.22	
Equivalent Magnitude	6.9	6.9		C_N	0.89	0.92	
MSF		1.17		$(N_1)_{60}$	20.8 ± 1.0	21.3	
CSRN		0.344					

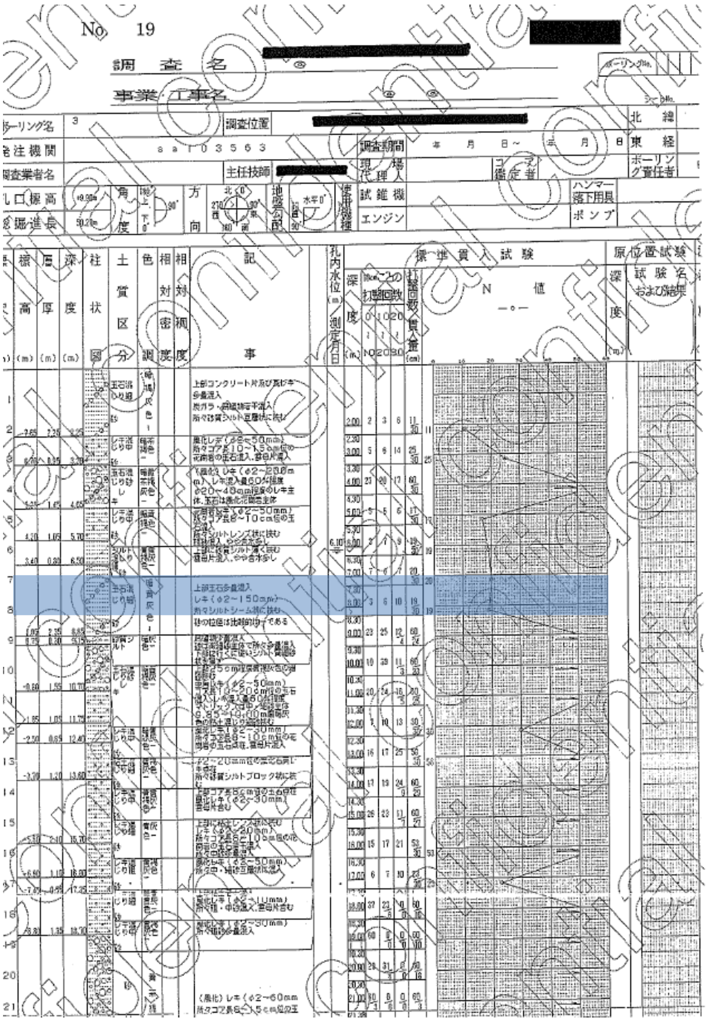

γ_m=120±3 pcf

γ_s=125±3 pcf

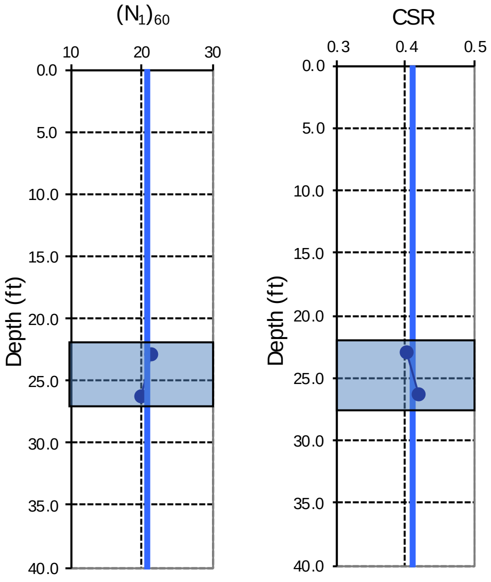

Depth (m)	Depth (ft)	CSR	N	C_N	C_R	$(N1)_{60}$	FC	
7.0	23.0	0.401	20.0	0.90	0.98	*21.5*	*10.0*	
8.0	26.2	0.420	19.0	0.87	1.00	*20.1*	*10.0*	
					**Mean:**	20.8	10.0	
					**St. Dev.**	1.0	2.0	assigned

**Case number:**	178
**Earthquake:**	1995 Hyogoken-Nambu
**Magnitude:**	7.2 (M_L)
**Location:**	Kobe No : 20
**References:**	Tokimatsu, K. Personal Communication (2000)
**Nature of Failure:**	Nonliquefied
**Comments:**	

Summary of Data

	Cetin et al. (2016)	Idriss&Boulanger (2010)	Seed et.al. (1984)		Cetin et al. (2016)	Idriss&Boulanger (2010)	Seed et.al. (1984)
Liquefied?	No	No		D_{50}	0.000 ± 0.000		
Data Class	B			% Fines	0.0 ± 2.0	0.0	
Critical Depth Range	13.1 - 26.2	19.7		% PI			
Depth to GWT (ft)	6.6 ± 0.3	6.6					
σ_v (psf)	2493.4 ± 298.6	2380.9		N	50.4 ± 3.5	50.8	
σ_v' (psf)	1674.5 ± 165.2	1566.4		C_R	0.95	0.95	
a_{max} (g)	0.550 ± 0.110	0.55		C_S	1.00	1.00	
r_d	0.99 ± 0.082	0.93		C_B	1.00	1.00	
CSR	0.530 ± 0.115	0.505		C_E	1.22	1.22	
Equivalent Magnitude	6.9	6.9		C_N	1.12	1.08	
MSF		1.17		$(N_1)_{60}$	65.5 ± 4.5	63.7	
CSRN		0.396					

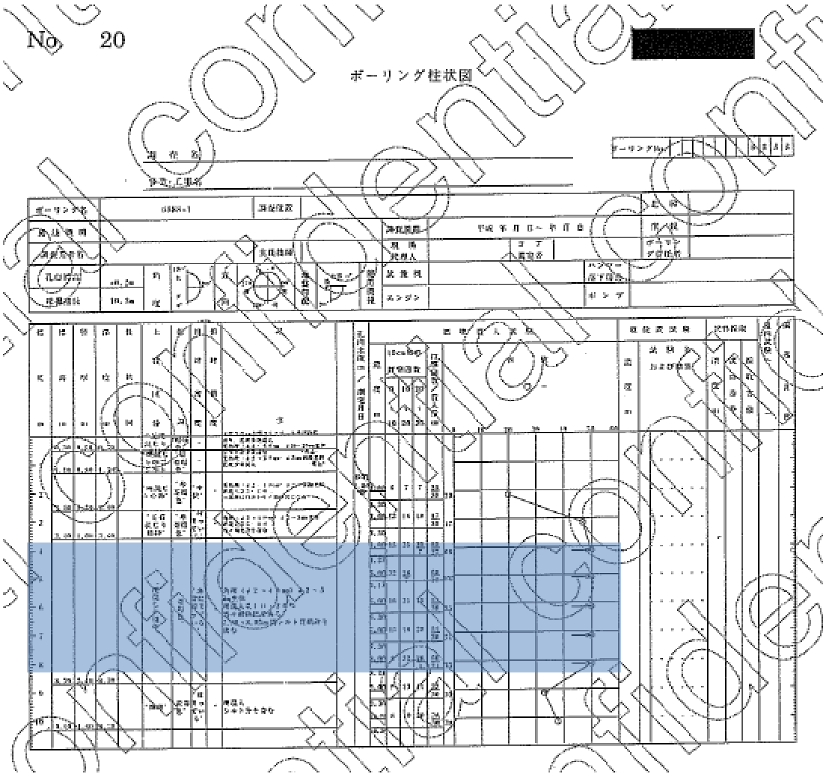

γ_m=110±3 pcf

γ_s=135±3 pcf

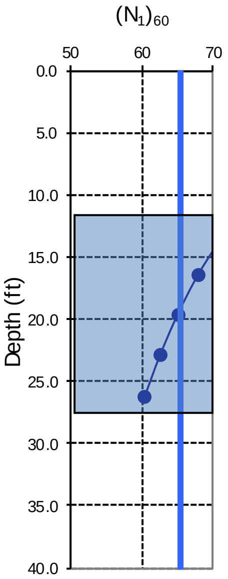

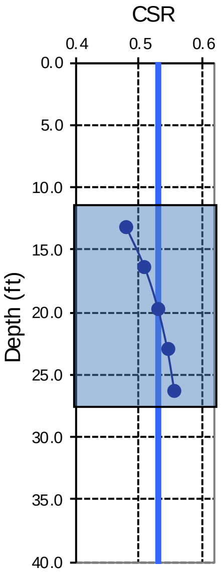

Depth (m)	Depth (ft)	CSR	N	C_N	C_R	$(N1)_{60}$	FC
4.0	13.1	0.479	50.0	1.33	0.89	*71.7*	*0.0*
5.0	16.4	0.509	50.0	1.21	0.92	*68.0*	*0.0*
6.0	19.7	0.530	50.0	1.12	0.95	*65.0*	*0.0*
7.0	23.0	0.545	50.0	1.05	0.98	*62.5*	*0.0*
8.0	26.2	0.556	50.0	0.99	1.00	*60.3*	*0.0*
					**Mean:**	65.5	0.0
					**St. Dev.**	4.5	2.0

**Case number:**	179
**Earthquake:**	1995 Hyogoken-Nambu
**Magnitude:**	7.2 (M_L)
**Location:**	Kobe No : 21
**References:**	Tokimatsu, K. Personal Communication (2000)
**Nature of Failure:**	Nonliquefied
**Comments:**	

Summary of Data

	Cetin et al. (2016)	Idriss&Boulanger (2010)	Seed et.al. (1984)		Cetin et al. (2016)	Idriss&Boulanger (2010)	Seed et.al. (1984)
Liquefied?	No	No		D_{50}	0.000 ± 0.000		
Data Class	B			% Fines	0.0 ± 2.0	0.0	
Critical Depth Range	9.8 - 13.1	11.5		% PI			
Depth to GWT (ft)	5.4 ± 0.3	5.6					
σ_v (psf)	1300.0 ± 73.0	1294.9		N	23.0 ± 1.8	24.4	
σ_v' (psf)	921.3 ± 43.5	919.0		C_R	0.87	0.85	
a_{max} (g)	0.600 ± 0.120	0.60		C_S	1.00	1.00	
r_d	0.99 ± 0.052	0.97		C_B	1.00	1.00	
CSR	0.545 ± 0.114	0.531		C_E	1.22	1.22	
Equivalent Magnitude	6.9	6.9		C_N	1.52	1.32	
MSF		1.17		$(N_1)_{60}$	36.8 ± 2.8	33.5	
CSRN		0.412					

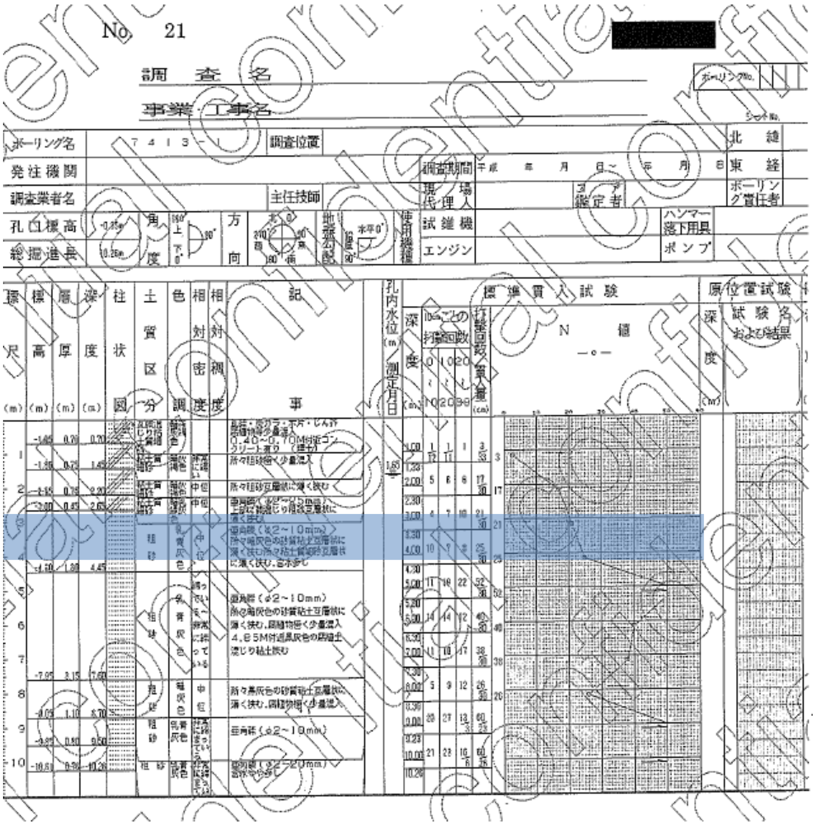

γ_m=100±3 pcf

γ_s=125±3 pcf

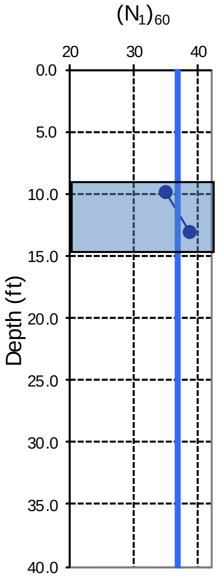

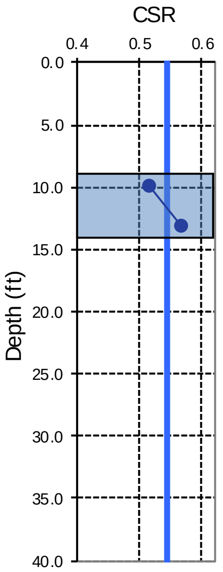

Depth (m)	Depth (ft)	CSR	N	C_N	C_R	$(N1)_{60}$	FC
3.0	9.8	0.518	21.0	1.61	0.85	*34.8*	*0.0*
4.0	13.1	0.567	25.0	1.44	0.89	*38.8*	*0.0*
					**Mean:**	36.8	0.0
					**St. Dev.**	2.8	2.0

**Case number:**	180
**Earthquake:**	1995 Hyogoken-Nambu
**Magnitude:**	7.2 (M_L)
**Location:**	Kobe No : 22
**References:**	Tokimatsu, K. Personal Communication (2000)
**Nature of Failure:**	Nonliquefied
**Comments:**	

**Summary of Data**							
	Cetin et al. (2016)	Idriss&Boulanger (2010)	Seed et.al. (1984)		Cetin et al. (2016)	Idriss&Boulanger (2010)	Seed et.al. (1984)
Liquefied?	No	No		D_{50}	0.000 ± 0.000		
Data Class	B			% Fines	6.0 ± 5.5	6.0	
Critical Depth Range	13.1 - 26.2	19.7		% PI			
Depth to GWT (ft)	7.9 ± 0.3	7.9					
σ_v (psf)	2421.3 ± 276.7	2380.9		N	30.0 ± 9.0	30.8	
σ_v' (psf)	1684.3 ± 144.4	1649.9		C_R	0.95	0.95	
a_{max} (g)	0.600 ± 0.120	0.60		C_S	1.00	1.00	
r_d	0.96 ± 0.082	0.93		C_B	1.00	1.00	
CSR	0.539 ± 0.119	0.524		C_E	1.22	1.22	
Equivalent Magnitude	6.9	6.9		C_N	1.12	1.08	
MSF		1.17		$(N_1)_{60}$	38.9 ± 11.7	38.6	
CSRN		0.416					

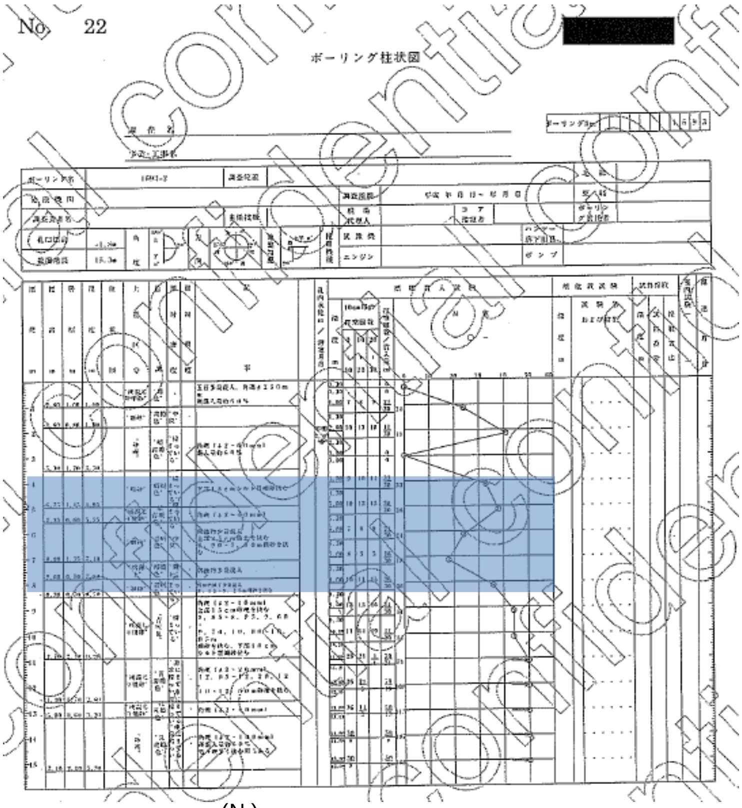

γ_m=120±3 pcf

γ_s=125±3 pcf

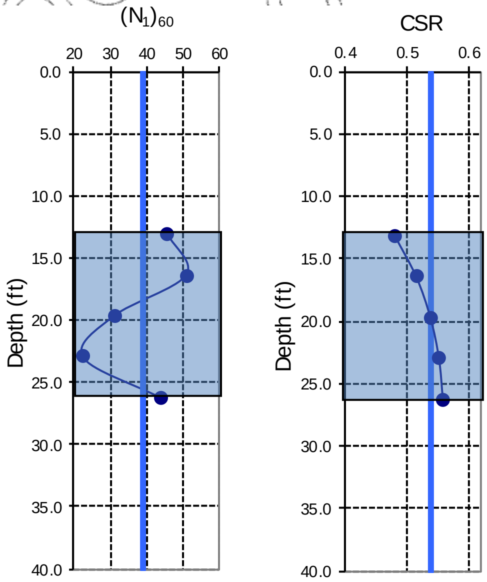

Depth (m)	Depth (ft)	CSR	N	C_N	C_R	$(N1)_{60}$	FC
4.0	13.1	0.482	33.0	1.29	0.89	***45.9***	***0.0***
5.0	16.4	0.516	38.0	1.20	0.92	***50.9***	***0.0***
6.0	19.7	0.539	24.0	1.12	0.95	***31.1***	***10.0***
7.0	23.0	0.553	18.0	1.06	0.98	***22.6***	***10.0***
8.0	26.2	0.559	36.0	1.01	1.00	***44.0***	***10.0***
					**Mean:**	38.9	6.0
					**St. Dev.**	11.7	5.5

**Case number:**	181
**Earthquake:**	1995 Hyogoken-Nambu
**Magnitude:**	7.2 (M_L)
**Location:**	Kobe No : 23
**References:**	Tokimatsu, K. Personal Communication (2000)
**Nature of Failure:**	Nonliquefied
**Comments:**	

**Summary of Data**	Cetin et al. (2016)	Idriss&Boulanger (2010)	Seed et.al. (1984)		Cetin et al. (2016)	Idriss&Boulanger (2010)	Seed et.al. (1984)
Liquefied?	No	No		D_{50}	0.000 ± 0.000		
Data Class	B			% Fines	10.0 ± 2.0	10.0	
Critical Depth Range	13.1 - 19.7	16.4		% PI			
Depth to GWT (ft)	9.8 ± 0.3	9.8					
σ_v (psf)	2001.3 ± 141.2	1921.5		N	18.0 ± 0.8	18.1	
σ_v' (psf)	1591.9 ± 79.0	1503.8		C_R	0.92	0.95	
a_{max} (g)	0.600 ± 0.120	0.60		C_S	1.00	1.00	
r_d	0.97 ± 0.070	0.94		C_B	1.00	1.00	
CSR	0.477 ± 0.102	0.468		C_E	1.22	1.22	
Equivalent Magnitude	6.9	6.9		C_N	1.15	1.15	
MSF		1.17		$(N_1)_{60}$	23.3 ± 1.0	24.0	
CSRN		0.379					

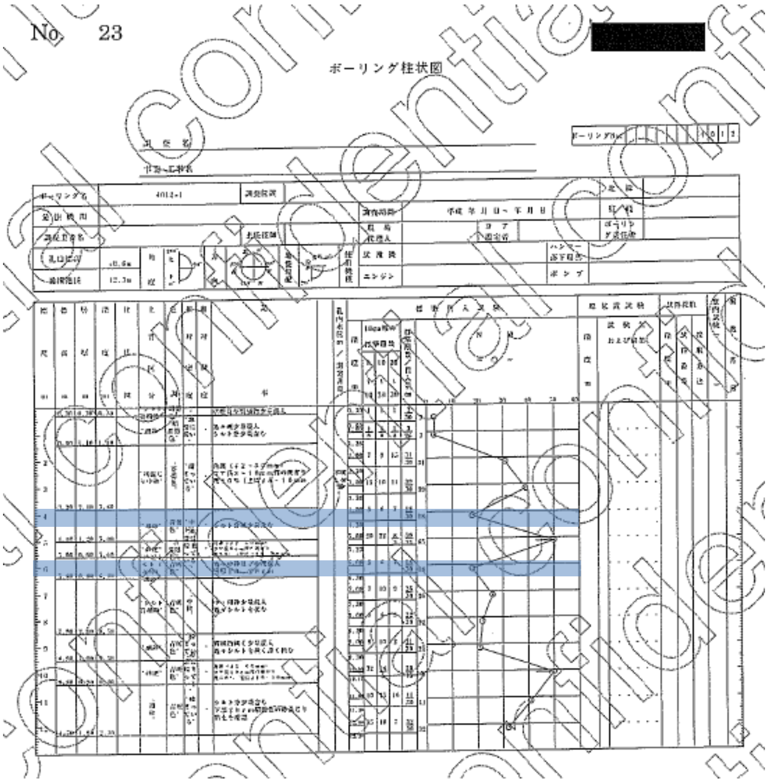

γ_m=120±3 pcf

γ_s=125±3 pcf

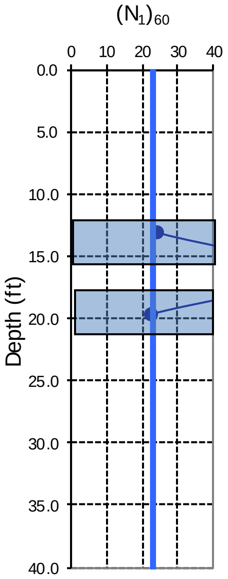

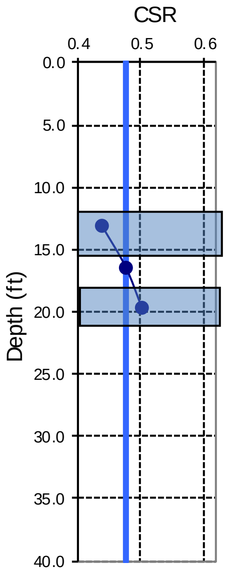

Depth (m)	Depth (ft)	CSR	N	C_N	C_R	$(N1)_{60}$	FC
4.0	13.1	0.440	18.0	1.24	0.89	***24.0***	***10.0***
5.0	16.4	0.477	50.0	1.15	0.92	64.6	0.0
6.0	19.7	0.503	18.0	1.09	0.95	***22.6***	***10.0***
					**Mean:**	23.3	10.0
					**St. Dev.**	1.0	2.0

**Case number:**	182
**Earthquake:**	1995 Hyogoken-Nambu
**Magnitude:**	7.2 (M_L)
**Location:**	Kobe No : 24
**References:**	Tokimatsu, K. Personal Communication (2000)
**Nature of Failure:**	Nonliquefied
**Comments:**	

Summary of Data

	Cetin et al. (2016)	Idriss&Boulanger (2010)	Seed et.al. (1984)		Cetin et al. (2016)	Idriss&Boulanger (2010)	Seed et.al. (1984)
Liquefied?	Yes	Yes		D_{50}	0.000 ± 0.000		
Data Class	B			% Fines	0.0 ± 2.0	0.0	
Critical Depth Range	9.8 - 13.1	11.5		% PI			
Depth to GWT (ft)	7.7 ± 0.3	7.9					
σ_v (psf)	1339.4 ± 70.5	1315.8		N	17.0 ± 1.0	18.0	
σ_v' (psf)	1104.0 ± 44.2	1065.2		C_R	0.87	0.85	
a_{max} (g)	0.500 ± 0.100	0.50		C_S	1.00	1.00	
r_d	0.98 ± 0.052	0.97		C_B	1.00	1.00	
CSR	0.388 ± 0.081	0.383		C_E	1.22	1.22	
Equivalent Magnitude	6.9	6.9		C_N	1.38	1.32	
MSF		1.17		$(N_1)_{60}$	24.8 ± 1.4	24.6	
CSRN		0.297					

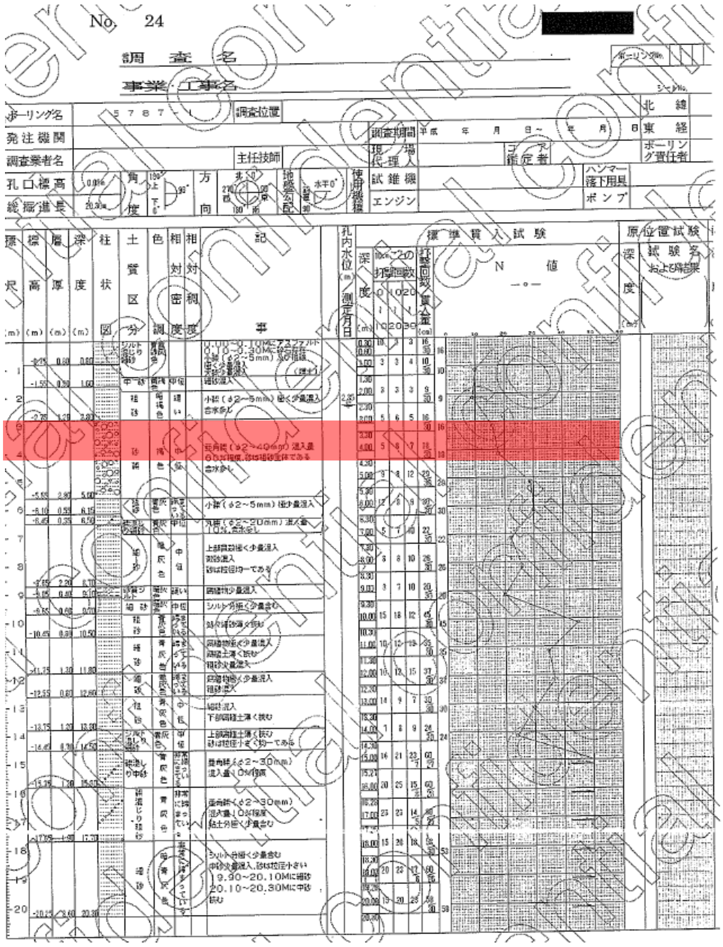

γ_m=115±3 pcf

γ_s=120±3 pcf

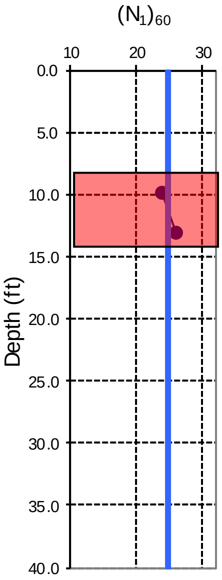

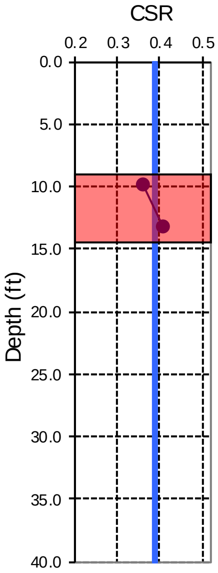

Depth (m)	Depth (ft)	CSR	N	C_N	C_R	$(N1)_{60}$	FC
3.0	9.8	0.363	16.0	1.45	0.85	***23.9***	***0.0***
4.0	13.1	0.408	18.0	1.33	0.89	***25.8***	***0.0***
					**Mean:**	24.8	0.0
					**St. Dev.**	1.4	2.0

**Case number:**	183
**Earthquake:**	1995 Hyogoken-Nambu
**Magnitude:**	7.2 (M_L)
**Location:**	Kobe No : 25
**References:**	Tokimatsu, K. Personal Communication (2000)
**Nature of Failure:**	Nonliquefied
**Comments:**	

Summary of Data

	Cetin et al. (2016)	Idriss&Boulanger (2010)	Seed et.al. (1984)		Cetin et al. (2016)	Idriss&Boulanger (2010)	Seed et.al. (1984)
Liquefied?	No	No		D_{50}	0.000 ± 0.000		
Data Class	B			% Fines	2.5 ± 3.5	3.0	
Critical Depth Range	9.8 - 13.1	11.5		% PI			
Depth to GWT (ft)	7.1 ± 0.3	7.2					
σ_v (psf)	1329.6 ± 72.9	1336.7		N	26.1 ± 0.9	27.5	
σ_v' (psf)	1053.2 ± 44.7	1044.3		C_R	0.87	0.85	
a_{max} (g)	0.700 ± 0.140	0.70		C_S	1.00	1.00	
r_d	0.99 ± 0.052	0.97		C_B	1.00	1.00	
CSR	0.568 ± 0.118	0.555		C_E	1.22	1.22	
Equivalent Magnitude	6.9	6.9		C_N	1.42	1.25	
MSF		1.17		$(N_1)_{60}$	39.0 ± 1.4	35.8	
CSRN		0.431					

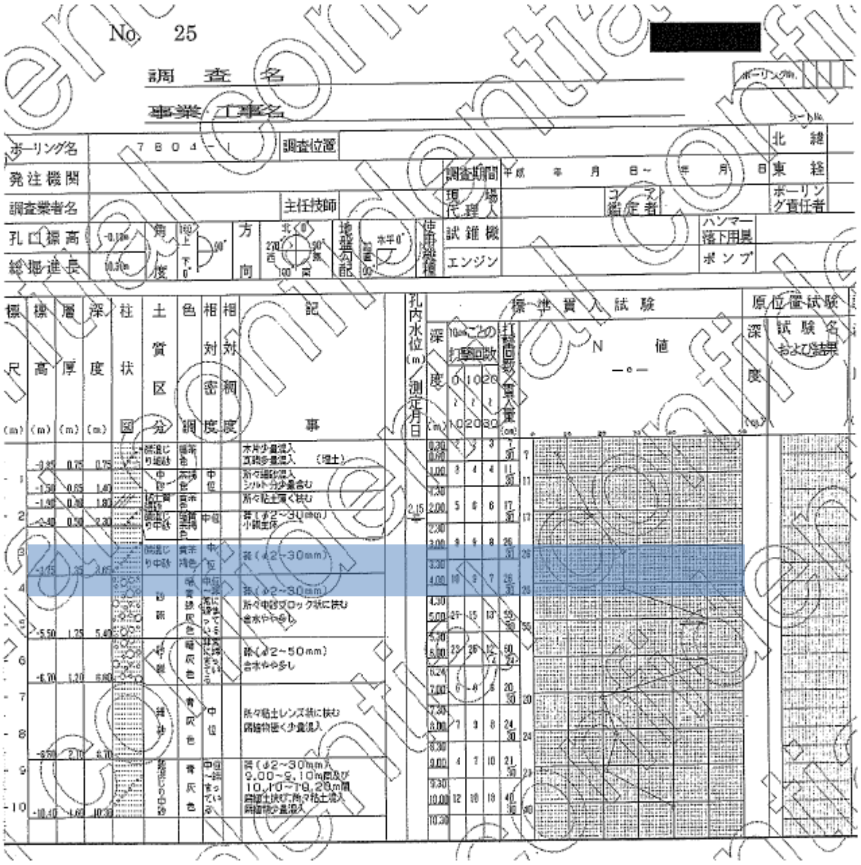

γ_m=110±3 pcf

γ_s=125±3 pcf

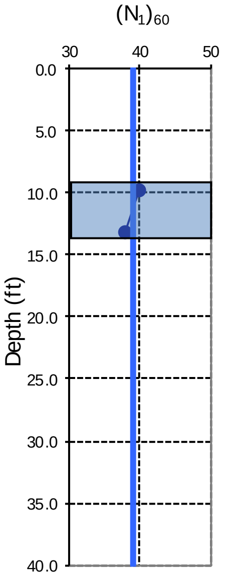

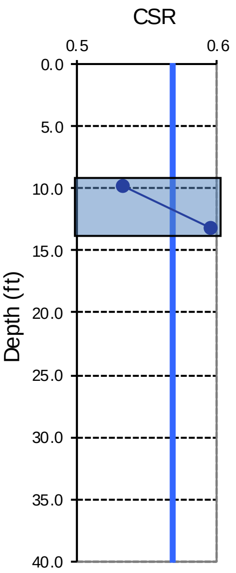

Depth (m)	Depth (ft)	CSR	N	C_N	C_R	$(N1)_{60}$	FC
3.0	9.8	0.534	26.0	1.49	0.85	*40.0*	*5.0*
4.0	13.1	0.596	26.0	1.35	0.89	*38.0*	*0.0*
					**Mean:**	39.0	2.5
					**St. Dev.**	1.4	3.5

**Case number:**	184
**Earthquake:**	1995 Hyogoken-Nambu
**Magnitude:**	7.2 (M_L)
**Location:**	Kobe No : 26
**References:**	Tokimatsu, K. Personal Communication (2000)
**Nature of Failure:**	Non-liquefied
**Comments:**	

**Summary of Data**							
	Cetin et al. (2016)	Idriss&Boulanger (2010)	Seed et.al. (1984)		Cetin et al. (2016)	Idriss&Boulanger (2010)	Seed et.al. (1984)
Liquefied?	No	No		D_{50}	0.000 ± 0.000		
Data Class	B			% Fines	0.0 ± 2.0	0.0	
Critical Depth Range	9.8 - 13.1	11.5		% PI			
Depth to GWT (ft)	3.0 ± 0.3	3.0					
σ_v (psf)	1361.5 ± 73.9	1315.8		N	24.5 ± 3.6	26.0	
σ_v' (psf)	829.3 ± 45.1	772.8		C_R	0.87	0.85	
a_{max} (g)	0.600 ± 0.120	0.60		C_S	1.00	1.00	
r_d	0.99 ± 0.052	0.97		C_B	1.00	1.00	
CSR	0.634 ± 0.132	0.633		C_E	1.22	1.22	
Equivalent Magnitude	6.9	6.9		C_N	1.60	1.37	
MSF		1.17		$(N_1)_{60}$	41.2 ± 6.1	37.0	
CSRN		0.491					

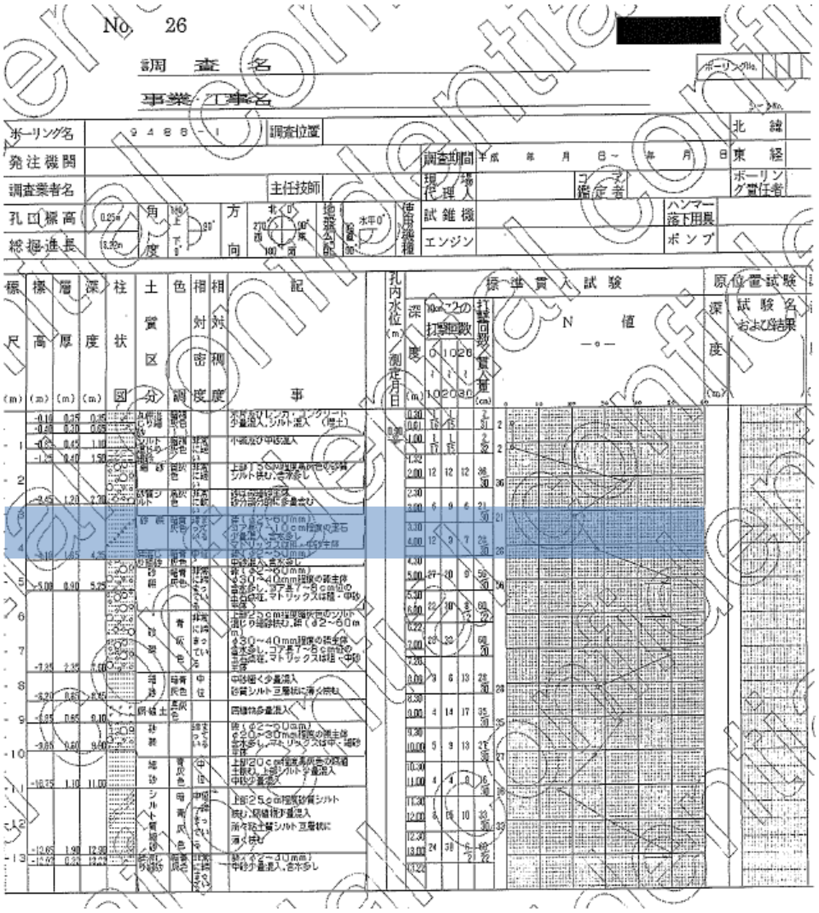

γ_m=100±3 pcf

γ_s=125±3 pcf

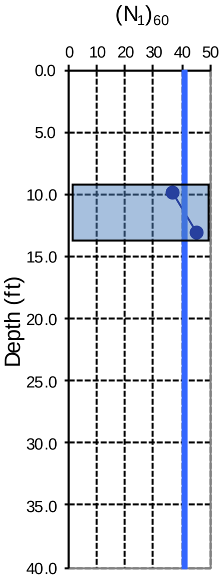

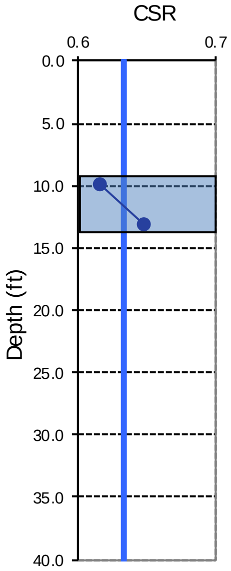

Depth (m)	Depth (ft)	CSR	N	C_N	C_R	$(N1)_{60}$	FC
3.0	9.8	0.616	21.0	1.71	0.85	***36.9***	***0.0***
4.0	13.1	0.648	28.0	1.51	0.89	***45.5***	***0.0***
					**Mean:**	41.2	0.0
					**St. Dev.**	6.1	2.0

**Case number:**	185
**Earthquake:**	1995 Hyogoken-Nambu
**Magnitude:**	7.2 (M_L)
**Location:**	Kobe No : 27
**References:**	Tokimatsu, K. Personal Communication (2000)
**Nature of Failure:**	Non-liquefied
**Comments:**	

Summary of Data

	Cetin et al. (2016)	Idriss&Boulanger (2010)	Seed et.al. (1984)		Cetin et al. (2016)	Idriss&Boulanger (2010)	Seed et.al. (1984)
Liquefied?	No	No		D_{50}	0.000 ± 0.000		
Data Class	B			% Fines	10.0 ± 2.0	10.0	
Critical Depth Range	6.6 - 9.8	8.2		% PI			
Depth to GWT (ft)	3.4 ± 0.3	3.6					
σ_v (psf)	939.1 ± 71.0	898.1		N	27.7 ± 4.1	27.6	
σ_v' (psf)	642.3 ± 40.1	605.7		C_R	0.82	0.85	
a_{max} (g)	0.600 ± 0.120	0.60		C_S	1.00	1.00	
r_d	1.00 ± 0.039	0.98		C_B	1.00	1.00	
CSR	0.569 ± 0.117	0.568		C_E	1.22	1.22	
Equivalent Magnitude	6.9	6.9		C_N	1.82	1.43	
MSF		1.17		$(N_1)_{60}$	50.4 ± 7.5	40.8	
CSRN		0.441					

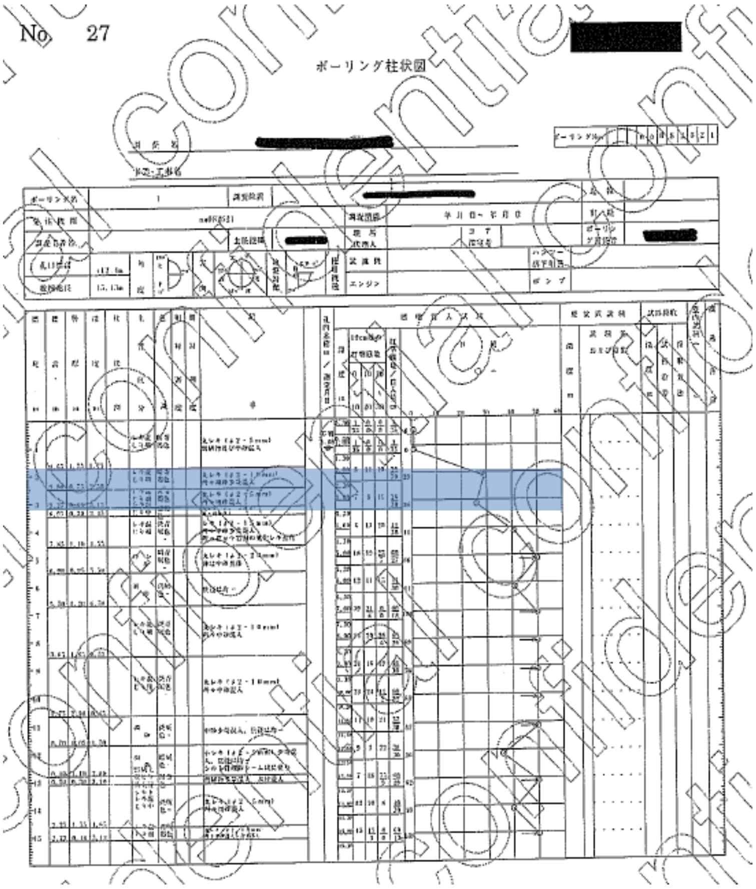

γ_m=100±3 pcf

γ_s=125±3 pcf

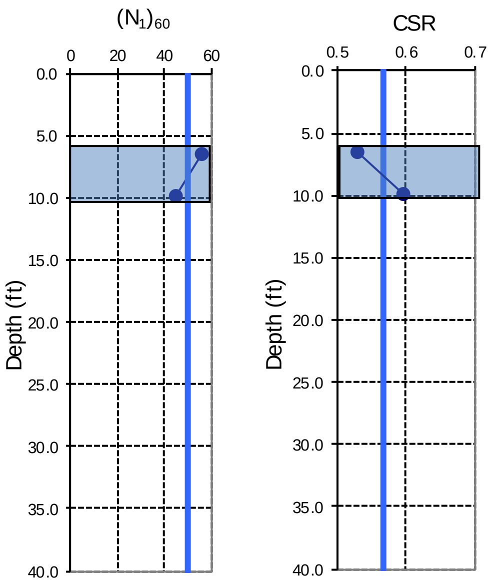

Depth (m)	Depth (ft)	CSR	N	C_N	C_R	$(N1)_{60}$	FC
2.0	6.6	0.530	29.0	1.98	0.80	*55.7*	*10.0*
3.0	9.8	0.597	26.0	1.69	0.85	*45.1*	*10.0*
					**Mean:**	50.4	10.0
					**St. Dev.**	7.5	2.0

**Case number:**	186
**Earthquake:**	1995 Hyogoken-Nambu
**Magnitude:**	7.2 (M_L)
**Location:**	Kobe No : 28
**References:**	Tokimatsu, K. Personal Communication (2000)
**Nature of Failure:**	Non-liquefied
**Comments:**	

**Summary of Data**	Cetin et al. (2016)	Idriss&Boulanger (2010)	Seed et.al. (1984)		Cetin et al. (2016)	Idriss&Boulanger (2010)	Seed et.al. (1984)
Liquefied?	Yes	Yes		D_{50}	0.000 ± 0.000		
Data Class	B			% Fines	8.0 ± 2.0	8.0	
Critical Depth Range	9.8 - 16.4	11.5		% PI			
Depth to GWT (ft)	5.7 ± 0.3	5.9					
σ_v (psf)	1554.3 ± 139.6	1294.9		N	14.3 ± 3.7	14.3	
σ_v' (psf)	1093.7 ± 75.3	919.0		C_R	0.89	0.85	
a_{max} (g)	0.400 ± 0.080	0.40		C_S	1.00	1.00	
r_d	0.96 ± 0.058	0.97		C_B	1.00	1.00	
CSR	0.355 ± 0.075	0.348		C_E	1.22	1.22	
Equivalent Magnitude	6.9	6.9		C_N	1.39	1.42	
MSF		1.17		$(N_1)_{60}$	21.5 ± 5.6	21.1	
CSRN		0.270					

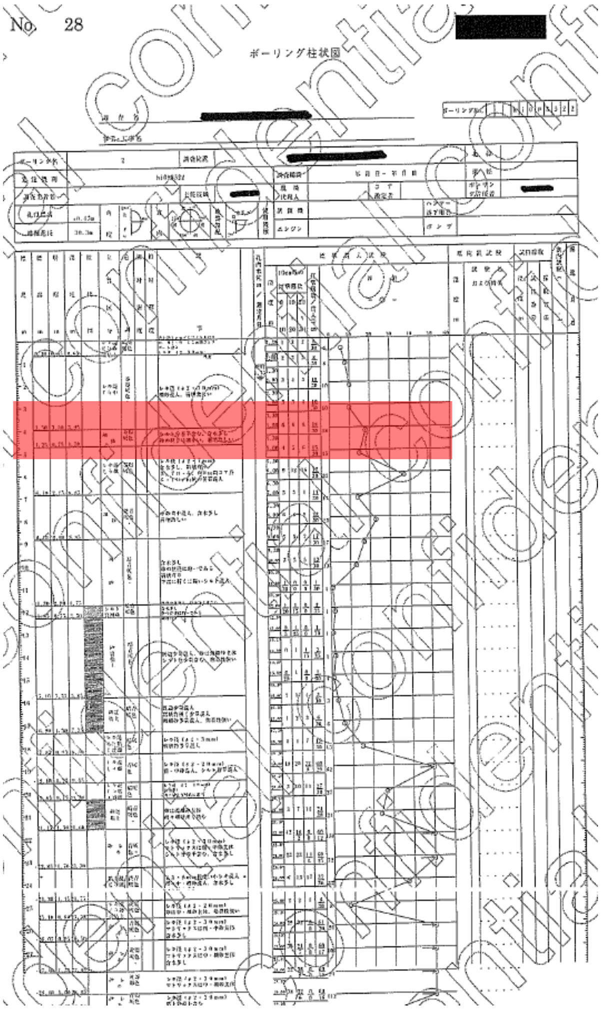

γ_m=110±3 pcf

γ_s=125±3 pcf

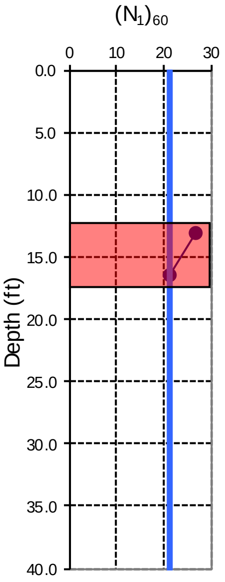

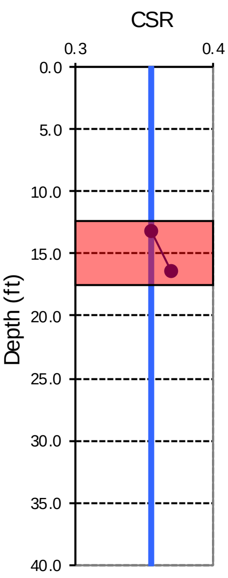

Depth (m)	Depth (ft)	CSR	N	C_N	C_R	$(N1)_{60}$	FC	
3.0	9.8	0.327	10.0	1.54	0.85	***15.9***	***4.0***	interpreted
4.0	13.1	0.355	18.0	1.39	0.89	***27.0***	***10.0***	
5.0	16.4	0.371	15.0	1.28	0.92	***21.5***	***10.0***	
					**Mean:**	21.5	8.0	
					**St. Dev.**	5.6	2.0	

**Case number:**	187
**Earthquake:**	1995 Hyogoken-Nambu
**Magnitude:**	7.2 (M_L)
**Location:**	Kobe No : 29
**References:**	Tokimatsu, K. Personal Communication (2000)
**Nature of Failure:**	Liquefied
**Comments:**	

**Summary of Data**	Cetin et al. (2016)	Idriss&Boulanger (2010)	Seed et.al. (1984)		Cetin et al. (2016)	Idriss&Boulanger (2010)	Seed et.al. (1984)
Liquefied?	Yes	Yes		D_{50}	0.000 ± 0.000		
Data Class	B			% Fines	0.0 ± 2.0	0.0	
Critical Depth Range	9.8 - 14.8	12.5		% PI			
Depth to GWT (ft)	6.6 ± 0.3	6.6					
σ_v (psf)	1439.5 ± 105.9	1399.3		N	12.1 ± 2.2	12.4	
σ_v' (psf)	1081.2 ± 59.4	1023.4		C_R	0.88	0.85	
a_{max} (g)	0.400 ± 0.080	0.40		C_S	1.00	1.00	
r_d	0.98 ± 0.055	0.96		C_B	1.00	1.00	
CSR	0.339 ± 0.071	0.337		C_E	1.22	1.22	
Equivalent Magnitude	6.9	6.9		C_N	1.40	1.39	
MSF		1.17		$(N_1)_{60}$	18.0 ± 3.3	17.9	
CSRN		0.264					

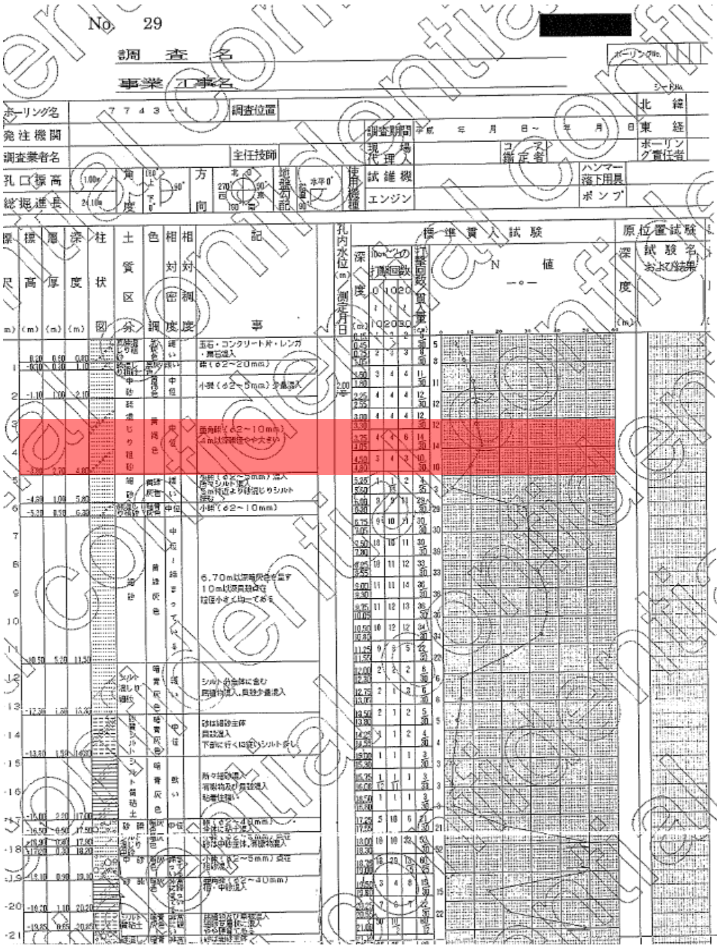

γ_m=110±3 pcf

γ_s=125±3 pcf

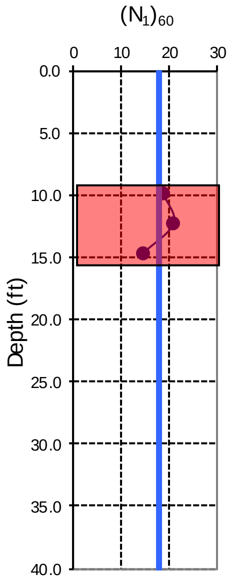

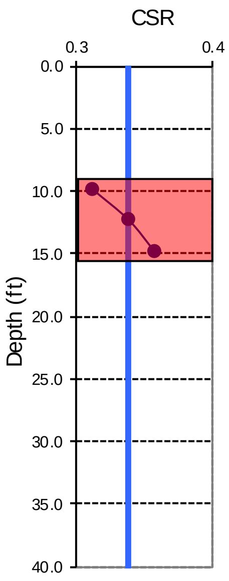

Depth (m)	Depth (ft)	CSR	N	C_N	C_R	$(N1)_{60}$	FC	
3.0	9.8	0.313	12.0	1.51	0.85	***18.7***	***0.0***	
3.8	12.3	0.339	14.0	1.40	0.88	***20.9***	***0.0***	
4.5	14.8	0.357	10.0	1.31	0.90	***14.4***	***0.0***	
					**Mean:**	18.0	0.0	
					**St. Dev.**	3.3	2.0	assigned

**Case number:**	188
**Earthquake:**	1995 Hyogoken-Nambu
**Magnitude:**	7.2 (M_L)
**Location:**	Kobe No : 30
**References:**	Tokimatsu, K. Personal Communication (2000)
**Nature of Failure:**	Non-Liquefied
**Comments:**	

**Summary of Data**	Cetin et al. (2016)	Idriss&Boulanger (2010)	Seed et.al. (1984)		Cetin et al. (2016)	Idriss&Boulanger (2010)	Seed et.al. (1984)
Liquefied?	No	No		D_{50}	0.000 ± 0.000		
Data Class	B			% Fines	10.0 ± 2.0	10.0	
Critical Depth Range	23.0 - 32.8	27.9		% PI			
Depth to GWT (ft)	4.9 ± 0.3	4.9					
σ_v (psf)	3362.9 ± 217.0	3049.3		N	30.6 ± 4.6	30.5	
σ_v' (psf)	1929.8 ± 125.0	1629.1		C_R	1.00	1.00	
a_{max} (g)	0.600 ± 0.120	0.60		C_S	1.00	1.00	
r_d	0.90 ± 0.110	0.88		C_B	1.00	1.00	
CSR	0.611 ± 0.144	0.649		C_E	1.22	1.22	
Equivalent Magnitude	6.9	6.9		C_N	1.05	1.08	
MSF		1.17		$(N_1)_{60}$	39.0 ± 5.8	40.1	
CSRN		0.514					

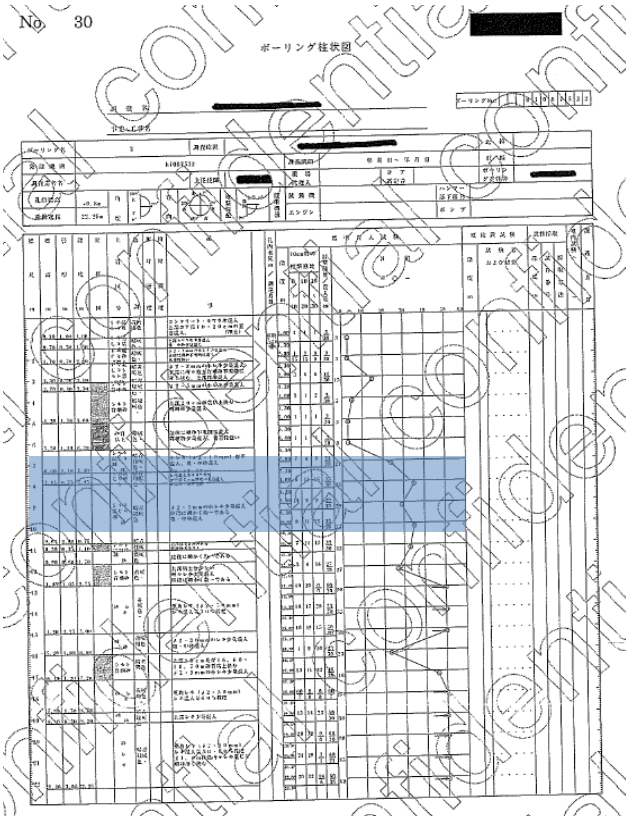

γ_m=100±3 pcf

γ_s=125±3 pcf

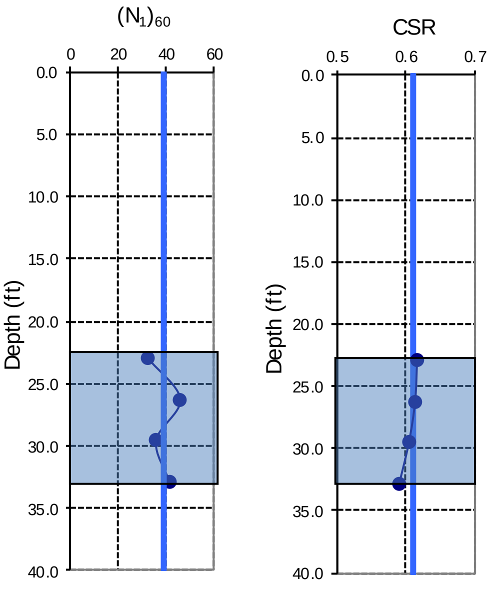

Depth (m)	Depth (ft)	CSR	N	C_N	C_R	$(N1)_{60}$	FC
7.0	23.0	0.618	24.0	1.14	0.98	***32.6***	***10.0***
8.0	26.2	0.615	35.0	1.08	1.00	***45.8***	***10.0***
9.0	29.5	0.606	29.0	1.02	1.00	***36.0***	***10.0***
10.0	32.8	0.592	35.0	0.97	1.00	***41.4***	***10.0***
					**Mean:**	39.0	10.0
					**St. Dev.**	5.8	2.0 assigned

**Case number:**	189
**Earthquake:**	1995 Hyogoken-Nambu
**Magnitude:**	7.2 (M_L)
**Location:**	Kobe No : 31
**References:**	Tokimatsu, K. Personal Communication (2000)
**Nature of Failure:**	Non-liquefied
**Comments:**	

Summary of Data

	Cetin et al. (2016)	Idriss&Boulanger (2010)	Seed et.al. (1984)		Cetin et al. (2016)	Idriss&Boulanger (2010)	Seed et.al. (1984)
Liquefied?	No	No		D_{50}	0.000 ± 0.000		
Data Class	B			% Fines	0.0 ± 2.0	0.0	
Critical Depth Range	9.8 - 16.4	13.1		% PI			
Depth to GWT (ft)	3.9 ± 0.3	3.9					
σ_v (psf)	1542.0 ± 140.2	1524.6		N	35.8 ± 3.7	34.8	
σ_v' (psf)	968.8 ± 75.6	960.7		C_R	0.89	0.95	
a_{max} (g)	0.600 ± 0.120	0.60		C_S	1.00	1.00	
r_d	0.99 ± 0.058	0.96		C_B	1.00	1.00	
CSR	0.614 ± 0.129	0.599		C_E	1.22	1.22	
Equivalent Magnitude	6.9	6.9		C_N	1.48	1.23	
MSF		1.17		$(N_1)_{60}$	57.0 ± 5.9	49.7	
CSRN		0.465					

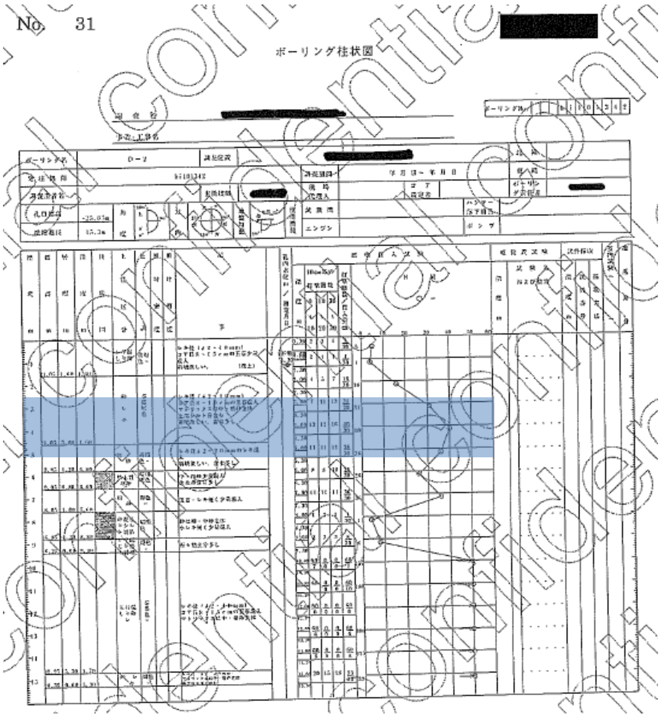

γ_m=100±3 pcf

γ_s=125±3 pcf

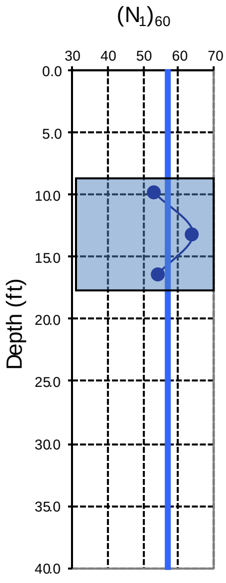

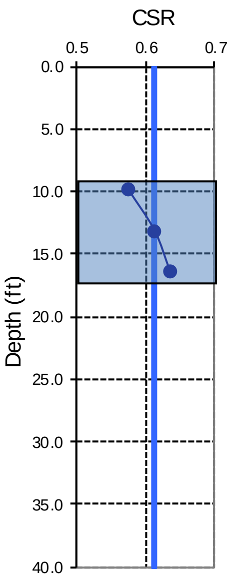

Depth (m)	Depth (ft)	CSR	N	C_N	C_R	$(N1)_{60}$	FC	
3.0	9.8	0.574	31.0	1.66	0.85	***53.2***	***0.0***	
4.0	13.1	0.614	40.0	1.48	0.89	***63.8***	***0.0***	
5.0	16.4	0.637	36.0	1.34	0.92	***54.2***	***0.0***	
					**Mean:**	57.0	0.0	
					**St. Dev.**	5.9	2.0	assigned

**Case number:**	190
**Earthquake:**	1995 Hyogoken-Nambu
**Magnitude:**	7.2 (M_L)
**Location:**	Kobe No : 32
**References:**	Tokimatsu, K. Personal Communication (2000)
**Nature of Failure:**	Non-liquefied
**Comments:**	

Summary of Data

	Cetin et al. (2016)	Idriss&Boulanger (2010)	Seed et.al. (1984)		Cetin et al. (2016)	Idriss&Boulanger (2010)	Seed et.al. (1984)
Liquefied?	No	No		D_{50}	0.000 ± 0.000		
Data Class	B			% Fines	6.3 ± 4.8	6.0	
Critical Depth Range	6.6 - 16.4	11.5		% PI			
Depth to GWT (ft)	4.6 ± 0.3	4.6					
σ_v (psf)	1320.5 ± 206.7	1274.0		N	18.8 ± 2.2	20.1	
σ_v' (psf)	890.6 ± 106.2	856.3		C_R	0.87	0.85	
a_{max} (g)	0.500 ± 0.100	0.50		C_S	1.00	1.00	
r_d	0.98 ± 0.052	0.97		C_B	1.00	1.00	
CSR	0.474 ± 0.100	0.472		C_E	1.22	1.22	
Equivalent Magnitude	6.9	6.9		C_N	1.54	1.40	
MSF		1.17		$(N_1)_{60}$	30.5 ± 3.6	29.1	
CSRN		0.367					

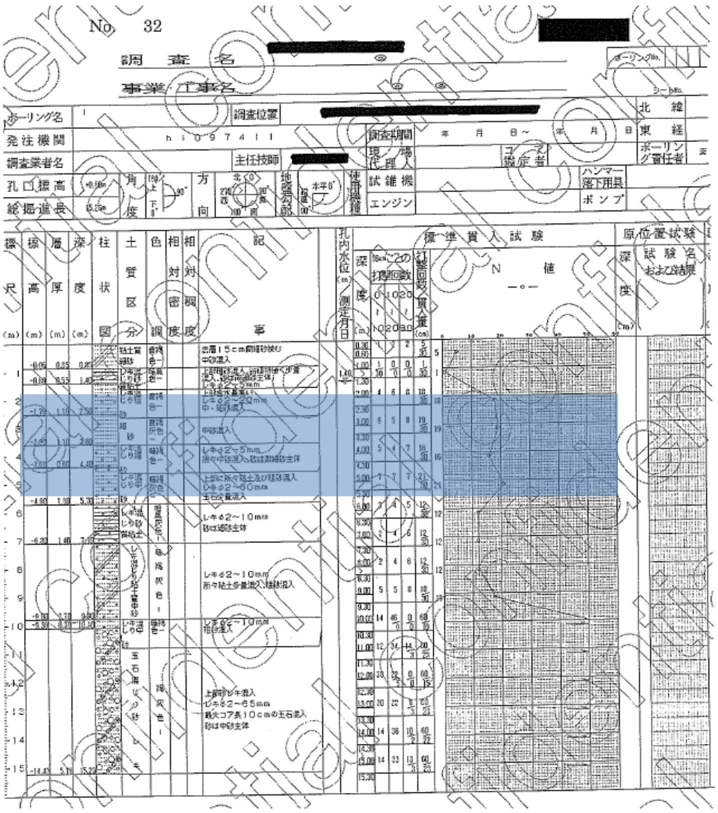

γ_m=100±3 pcf

γ_s=125±3 pcf

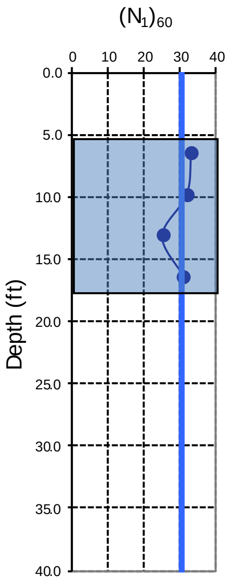

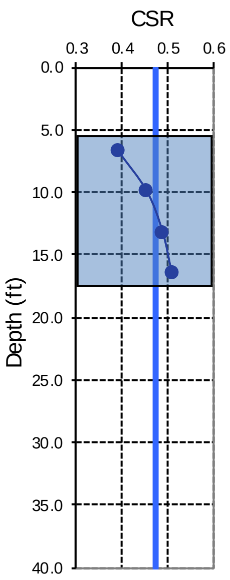

Depth (m)	Depth (ft)	CSR	N	C_N	C_R	$(N1)_{60}$	FC
2.0	6.6	0.391	18.0	1.91	0.80	***33.3***	***0.0***
3.0	9.8	0.454	19.0	1.64	0.85	***32.1***	***10.0***
4.0	13.1	0.489	16.0	1.46	0.89	***25.2***	***10.0***
5.0	16.4	0.509	21.0	1.33	0.92	***31.3***	***5.0***
					**Mean:**	30.5	6.3
					**St. Dev.**	3.6	4.8

**Case number:**	191
**Earthquake:**	1995 Hyogoken-Nambu
**Magnitude:**	7.2 (M_L)
**Location:**	Kobe No : 33
**References:**	Tokimatsu, K. Personal Communication (2000)
**Nature of Failure:**	Non-liquefied
**Comments:**	

**Summary of Data**	Cetin et al. (2016)	Idriss&Boulanger (2010)	Seed et.al. (1984)		Cetin et al. (2016)	Idriss&Boulanger (2010)	Seed et.al. (1984)
Liquefied?	No	No		D_{50}	0.000 ± 0.000		
Data Class	B			% Fines	50.0 ± 2.0	50.0	
Critical Depth Range	23.0 - 29.5	26.2		% PI			
Depth to GWT (ft)	6.6 ± 0.3	6.6					
σ_v (psf)	3116.8 ± 150.4	2965.7		N	21.6 ± 1.6	21.3	
σ_v' (psf)	1888.5 ± 93.2	1733.5		C_R	1.00	1.0	
a_{max} (g)	0.500 ± 0.100	0.50		C_S	1.00	1.00	
r_d	0.90 ± 0.104	0.89		C_B	1.00	1.00	
CSR	0.483 ± 0.112	0.496		C_E	1.22	1.22	
Equivalent Magnitude	6.9	6.9		C_N	1.06	1.1	
MSF		1.17		$(N_1)_{60}$	27.8 ± 2.0	27.9	
CSRN		0.404					

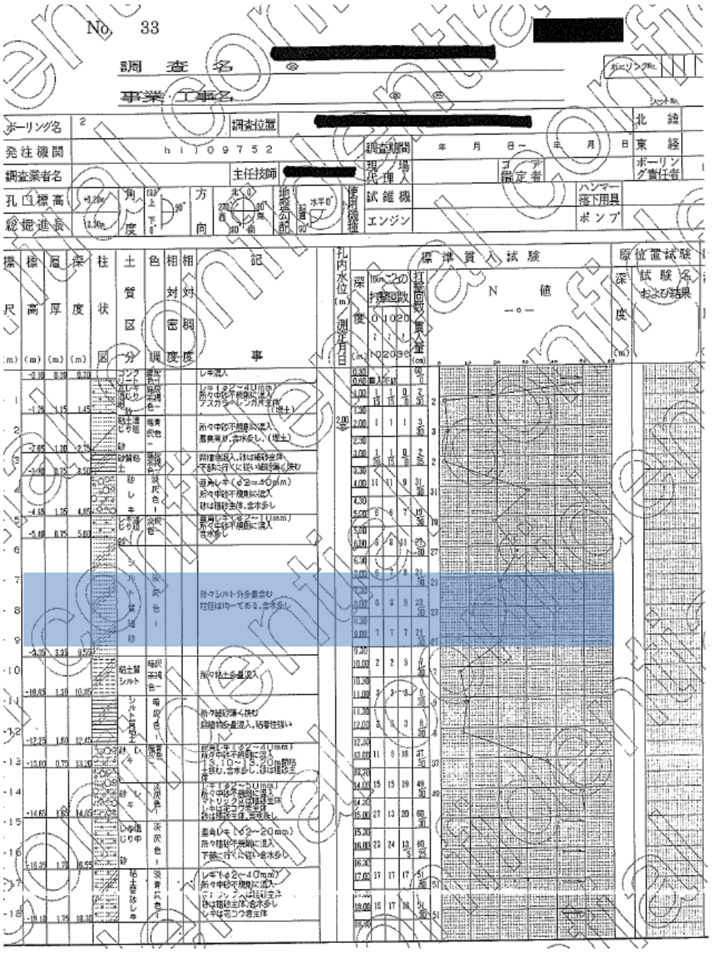

γ_m=100±3 pcf

γ_s=125±3 pcf

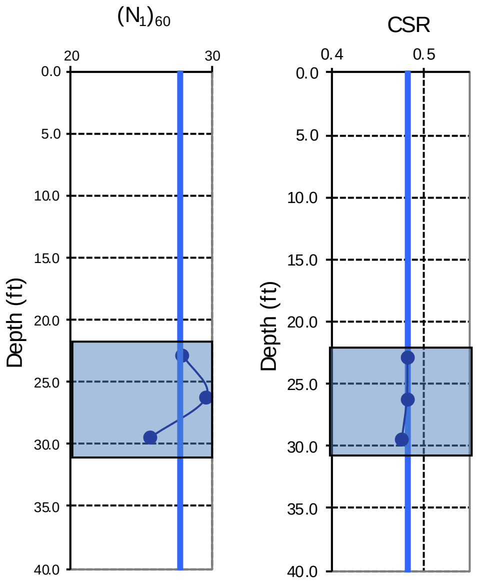

Depth (m)	Depth (ft)	CSR	N	C_N	C_R	$(N1)_{60}$	FC
7.0	23.0	0.484	21.0	1.12	0.98	***28.0***	***50.0***
8.0	26.2	0.483	23.0	1.06	1.00	***29.6***	***50.0***
9.0	29.5	0.477	21.0	1.01	1.00	***25.7***	***50.0***
					**Mean:**	27.8	50.0
					**St. Dev.**	2.0	2.0

**Case number:**	192
**Earthquake:**	1995 Hyogoken-Nambu
**Magnitude:**	7.2 (M_L)
**Location:**	Kobe No : 34
**References:**	Tokimatsu, K. Personal Communication (2000)
**Nature of Failure:**	Liquefied
**Comments:**	

**Summary of Data**	Cetin et al. (2016)	Idriss&Boulanger (2010)	Seed et.al. (1984)		Cetin et al. (2016)	Idriss&Boulanger (2010)	Seed et.al. (1984)
Liquefied?	Yes	Yes		D_{50}	0.000 ± 0.000		
Data Class	B			% Fines	9.3 ± 1.9	9.0	
Critical Depth Range	13.1 - 32.8	23.0		% PI			
Depth to GWT (ft)	5.9 ± 0.3	5.9					
σ_v (psf)	2752.6 ± 413.7	2589.8		N	17.7 ± 2.9	18.3	
σ_v' (psf)	1688.1 ± 212.8	1524.6		C_R	0.98	0.95	
a_{max} (g)	0.400 ± 0.080	0.40		C_S	1.00	1.00	
r_d	0.88 ± 0.093	0.91		C_B	1.00	1.00	
CSR	0.374 ± 0.085	0.403		C_E	1.22	1.22	
Equivalent Magnitude	6.9	6.9		C_N	1.12	1.14	
MSF		1.17		$(N_1)_{60}$	23.5 ± 3.8	24.2	
CSRN		0.326					

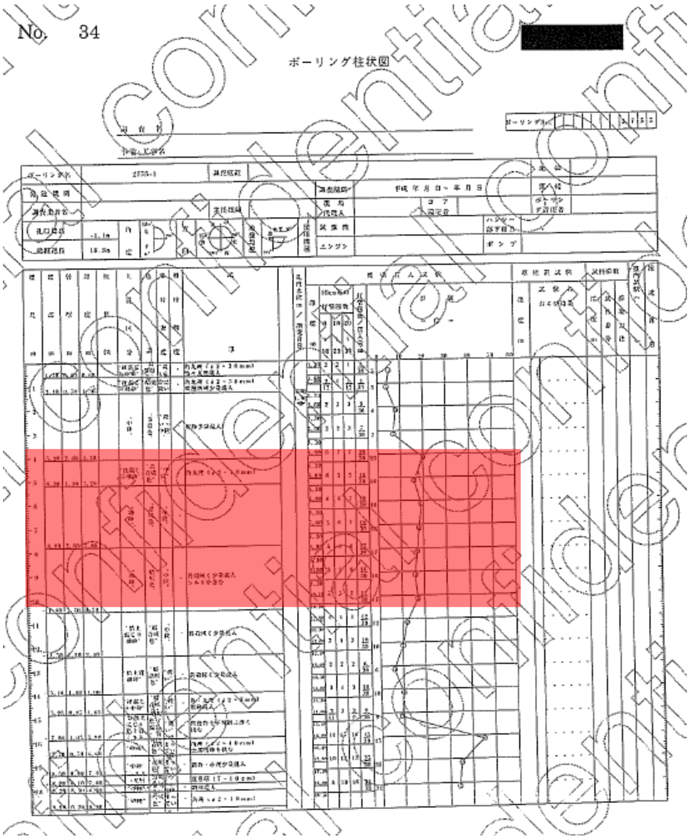

γ_m=105±3 pcf

γ_s=125±3 pc

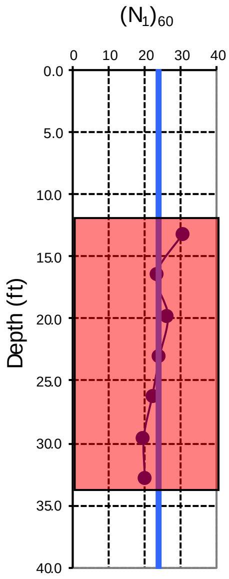

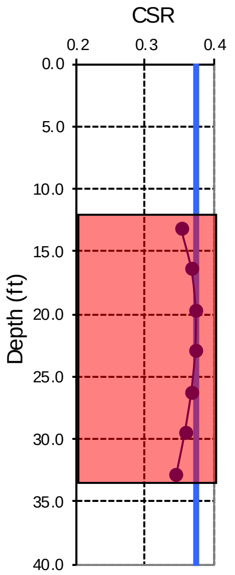

Depth (m)	Depth (ft)	CSR	N	C_N	C_R	$(N1)_{60}$	FC
4.0	13.1	0.354	20.0	1.41	0.89	***30.3***	***5.0***
5.0	16.4	0.369	16.0	1.29	0.92	***23.1***	***10.0***
6.0	19.7	0.375	19.0	1.19	0.95	***26.3***	***10.0***
7.0	23.0	0.374	18.0	1.12	0.98	***24.0***	***10.0***
8.0	26.2	0.368	17.0	1.06	1.00	***21.9***	***10.0***
9.0	29.5	0.359	16.0	1.00	1.00	***19.5***	***10.0***
10.0	32.8	0.347	17.0	0.96	1.00	***19.8***	***10.0***
					**Mean:**	23.5	9.3
					**St. Dev.**	3.8	1.9

**Case number:**	193
**Earthquake:**	1995 Hyogoken-Nambu
**Magnitude:**	7.2 (M_L)
**Location:**	Kobe No : 35
**References:**	Tokimatsu, K. Personal Communication (2000)
**Nature of Failure:**	Liquefied
**Comments:**	

**Summary of Data**	Cetin et al. (2016)	Idriss&Boulanger (2010)	Seed et.al. (1984)		Cetin et al. (2016)	Idriss&Boulanger (2010)	Seed et.al. (1984)
Liquefied?	Yes	Yes		D_{50}	0.000 ± 0.000		
Data Class	B			% Fines	6.3 ± 4.8	6.0	
Critical Depth Range	9.8 - 19.7	14.8		% PI			
Depth to GWT (ft)	6.7 ± 0.3	6.9					
σ_v (psf)	1744.6 ± 207.5	1649.9		N	12.3 ± 1.9	12.3	
σ_v' (psf)	1243.0 ± 108.3	1148.7		C_R	0.90	0.95	
a_{max} (g)	0.500 ± 0.100	0.50		C_S	1.00	1.00	
r_d	0.95 ± 0.064	0.95		C_B	1.00	1.00	
CSR	0.432 ± 0.093	0.445		C_E	1.22	1.22	
Equivalent Magnitude	6.9	6.9		C_N	1.30	1.32	
MSF		1.17		$(N_1)_{60}$	17.7 ± 2.7	18.9	
CSRN		0.352					

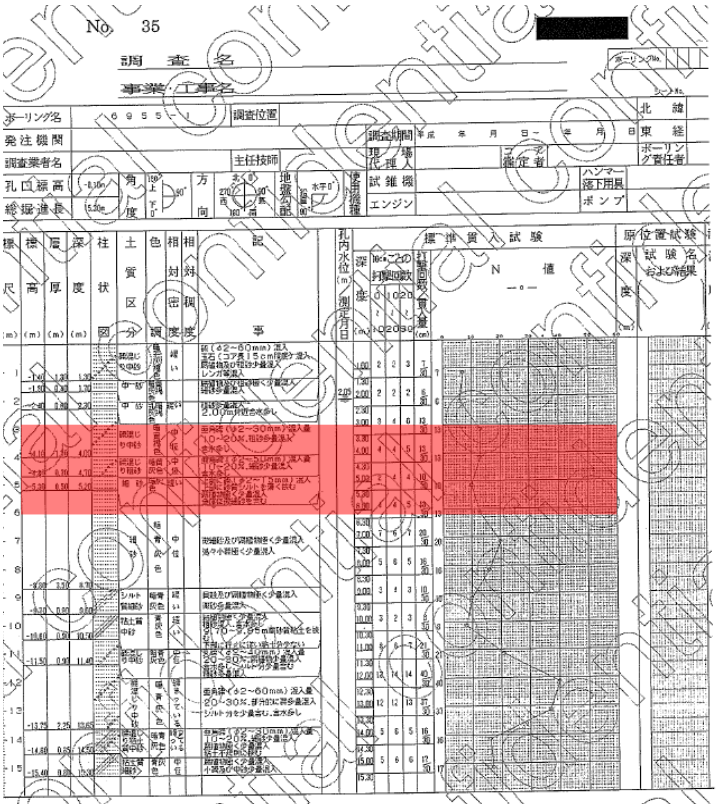

γ_m=110±3 pcf

γ_s=125±3 pcf

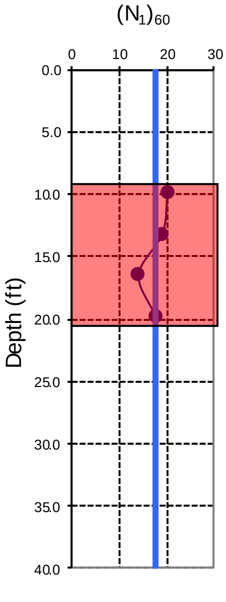

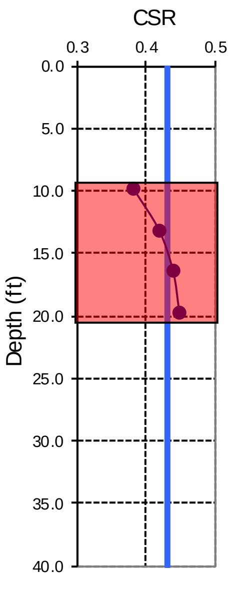

Depth (m)	Depth (ft)	CSR	N	C_N	C_R	$(N1)_{60}$	FC
3.0	9.8	0.382	13.0	1.50	0.85	***20.1***	***5.0***
4.0	13.1	0.419	13.0	1.36	0.89	***19.1***	***0.0***
5.0	16.4	0.440	10.0	1.25	0.92	***14.1***	***10.0***
6.0	19.7	0.450	13.0	1.17	0.95	***17.6***	***10.0***
					**Mean:**	17.7	6.3
					**St. Dev.**	2.7	4.8

**Case number:**	194
**Earthquake:**	1995 Hyogoken-Nambu
**Magnitude:**	7.2 (M_L)
**Location:**	Kobe No : 36
**References:**	Tokimatsu, K. Personal Communication (2000)
**Nature of Failure:**	Non-liquefied
**Comments:**	

**Summary of Data**	Cetin et al. (2016)	Idriss&Boulanger (2010)	Seed et.al. (1984)		Cetin et al. (2016)	Idriss&Boulanger (2010)	Seed et.al. (1984)
Liquefied?	No	No		D_{50}	0.000 ± 0.000		
Data Class	B			% Fines	2.5 ± 3.5	3.0	
Critical Depth Range	9.8 - 13.1	11.5		% PI			
Depth to GWT (ft)	3.1 ± 0.3	3.0					
σ_v (psf)	1358.3 ± 73.8	1274.0		N	20.1 ± 1.1	21.2	
σ_v' (psf)	834.2 ± 44.9	751.9		C_R	0.87	0.85	
a_{max} (g)	0.600 ± 0.120	0.60		C_S	1.00	1.00	
r_d	0.97 ± 0.052	0.97		C_B	1.00	1.00	
CSR	0.617 ± 0.129	0.639		C_E	1.22	1.22	
Equivalent Magnitude	6.9	6.9		C_N	1.59	1.44	
MSF		1.17		$(N_1)_{60}$	33.7 ± 1.8	31.6	
CSRN		0.496					

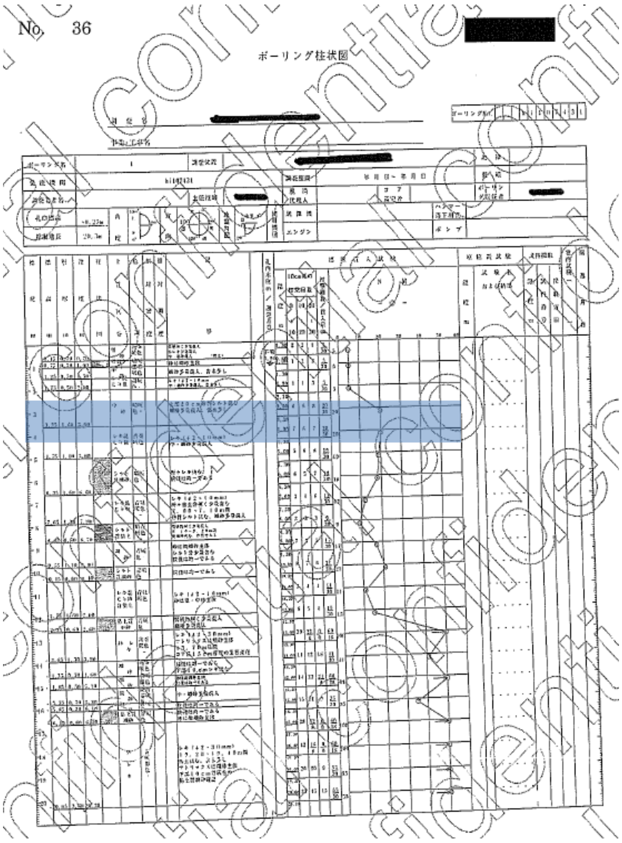

γ_m=100±3 pcf

γ_s=125±3 pcf

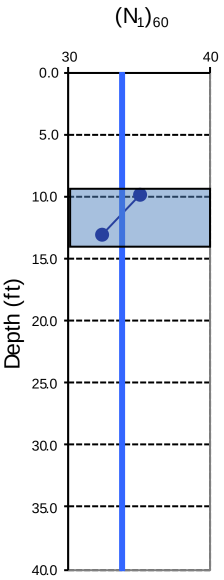

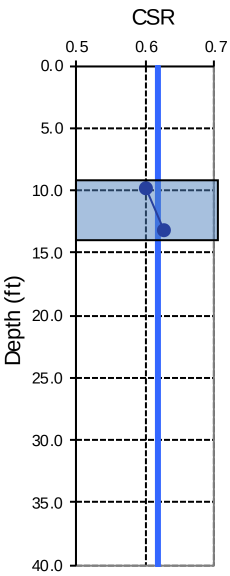

Depth (m)	Depth (ft)	CSR	N	C_N	C_R	$(N1)_{60}$	FC
3.0	9.8	0.601	20.0	1.70	0.85	***35.0***	***5.0***
4.0	13.1	0.628	20.0	1.50	0.89	***32.4***	***0.0***
					**Mean:**	33.7	2.5
					**St. Dev.**	1.8	3.5

**Case number:**	195
**Earthquake:**	1995 Hyogoken-Nambu
**Magnitude:**	7.2 (M_L)
**Location:**	Kobe No : 37
**References:**	Tokimatsu, K. Personal Communication (2000)
**Nature of Failure:**	Liquefied
**Comments:**	

Summary of Data

	Cetin et al. (2016)	Idriss&Boulanger (2010)	Seed et.al. (1984)		Cetin et al. (2016)	Idriss&Boulanger (2010)	Seed et.al. (1984)
Liquefied?	Yes	Yes		D_{50}	0.000 ± 0.000		
Data Class	B			% Fines	0.0 ± 2.0	0.0	
Critical Depth Range	13.1 - 19.7	16.4		% PI			
Depth to GWT (ft)	13.1 ± 0.3	13.1					
σ_v (psf)	1984.9 ± 142.6	1858.8		N	18.4 ± 2.5	15.0	
σ_v' (psf)	1780.2 ± 81.4	1649.9		C_R	0.92	0.95	
a_{max} (g)	0.350 ± 0.070	0.35		C_S	1.00	1.00	
r_d	0.91 ± 0.070	0.94		C_B	1.00	1.00	
CSR	0.232 ± 0.050	0.241		C_E	1.22	1.22	
Equivalent Magnitude	6.9	6.9		C_N	1.09	1.12	
MSF		1.17		$(N_1)_{60}$	22.5 ± 3.1	19.3	
CSRN		0.200					

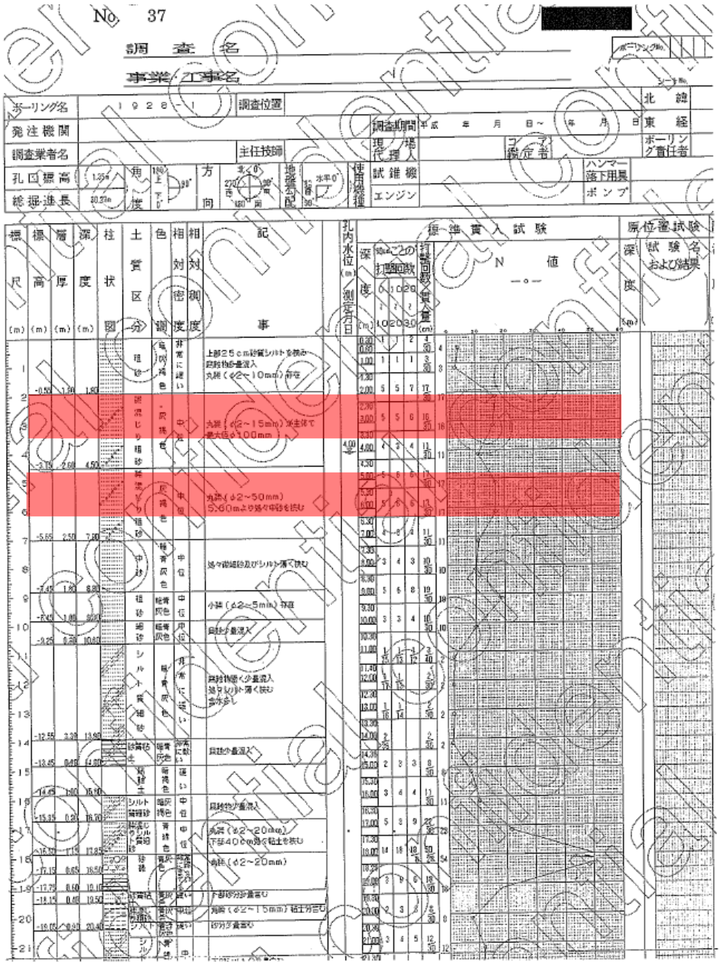

γ_m=120±3 pcf

γ_s=125±3 pcf

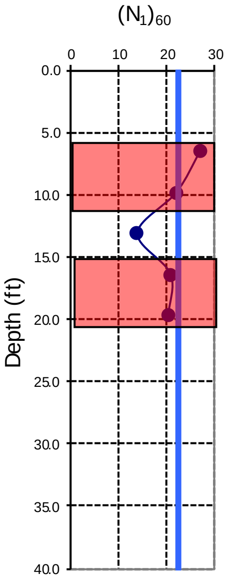

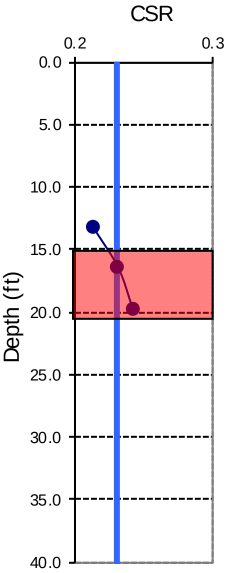

Depth (m)	Depth (ft)	CSR	N	C_N	C_R	$(N1)_{60}$	FC
2.0	6.6	NA	17.0	1.64	0.80	***27.0***	***0.0***
3.0	9.8	NA	16.0	1.34	0.85	***22.1***	***0.0***
4.0	13.1	0.214	11.0	1.16	0.89	*13.8*	*0.0*
5.0	16.4	0.232	17.0	1.09	0.92	***20.8***	***0.0***
6.0	19.7	0.242	17.0	1.03	0.95	***20.3***	***0.0***
					**Mean:**	22.5	0.0
					**St. Dev.**	3.1	2.0

assigned

**Case number:**	196
**Earthquake:**	1995 Hyogoken-Nambu
**Magnitude:**	7.2 (M_L)
**Location:**	Kobe No : 38
**References:**	Tokimatsu, K. Personal Communication (2000)
**Nature of Failure:**	Liquefied
**Comments:**	

Summary of Data

	Cetin et al. (2016)	Idriss&Boulanger (2010)	Seed et.al. (1984)		Cetin et al. (2016)	Idriss&Boulanger (2010)	Seed et.al. (1984)
Liquefied?	Yes	Yes		D_{50}	0.000 ± 0.000		
Data Class	B			% Fines	5.0 ± 2.0	5.0	
Critical Depth Range	19.7 - 32.8	26.2		% PI			
Depth to GWT (ft)	9.8 ± 0.3	9.8					
σ_v (psf)	3182.4 ± 279.4	2986.6		N	15.2 ± 2.1	15.1	
σ_v' (psf)	2158.8 ± 149.3	1963.2		C_R	1.00	1.00	
a_{max} (g)	0.500 ± 0.100	0.50		C_S	1.00	1.00	
r_d	0.85 ± 0.104	0.89		C_B	1.00	1.00	
CSR	0.409 ± 0.096	0.441		C_E	1.22	1.22	
Equivalent Magnitude	6.9	6.9		C_N	0.99	1.03	
MSF		1.17		$(N_1)_{60}$	18.3 ± 2.5	19.1	
CSRN		0.373					

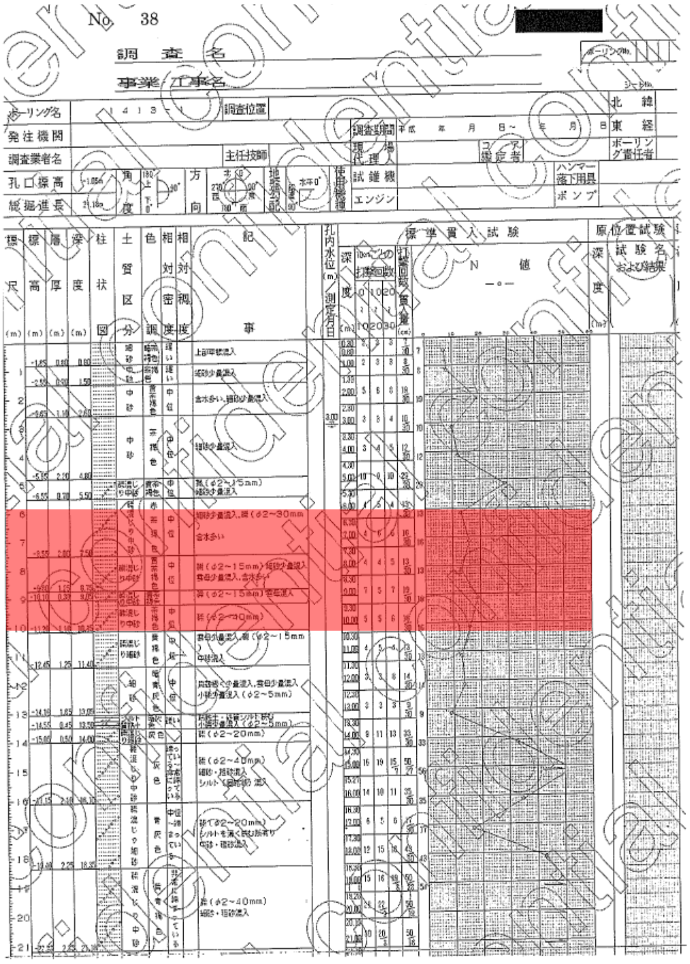

γ_m=115±3 pcf

γ_s=125±3 pcf

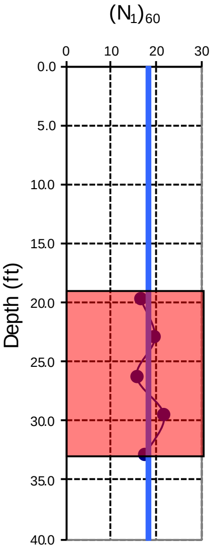

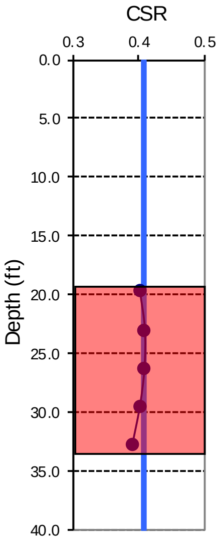

Depth (m)	Depth (ft)	CSR	N	C_N	C_R	$(N1)_{60}$	FC
6.0	19.7	0.403	13.0	1.10	0.95	***16.5***	***5.0***
7.0	23.0	0.409	16.0	1.04	0.98	***19.8***	***5.0***
8.0	26.2	0.409	13.0	0.99	1.00	***15.7***	***5.0***
9.0	29.5	0.403	19.0	0.95	1.00	***21.9***	***5.0***
10.0	32.8	0.393	16.0	0.91	1.00	***17.7***	***5.0***
					**Mean:**	18.3	5.0
					**St. Dev.**	2.5	2.0

assigned

**Case number:**	197
**Earthquake:**	1995 Hyogoken-Nambu
**Magnitude:**	7.2 (M_L)
**Location:**	Kobe No : 39
**References:**	Tokimatsu, K. Personal Communication (2000)
**Nature of Failure:**	Non-liquefied
**Comments:**	

Summary of Data

	Cetin et al. (2016)	Idriss&Boulanger (2010)	Seed et.al. (1984)		Cetin et al. (2016)	Idriss&Boulanger (2010)	Seed et.al. (1984)
Liquefied?	No	No		D_{50}	0.000 ± 0.000		
Data Class	B			% Fines	0.0 ± 2.0	0.0	
Critical Depth Range	13.1 - 16.4	14.8		% PI			
Depth to GWT (ft)	8.5 ± 0.3	8.5					
σ_v (psf)	1865.2 ± 80.5	1754.4		N	47.0 ± 2.8	47.0	
σ_v' (psf)	1476.2 ± 52.8	1378.4		C_R	0.90	0.95	
a_{max} (g)	0.600 ± 0.120	0.60		C_S	1.00	1.00	
r_d	1.00 ± 0.064	0.95		C_B	1.00	1.00	
CSR	0.492 ± 0.104	0.476		C_E	1.22	1.22	
Equivalent Magnitude	6.9	6.9		C_N	1.20	1.12	
MSF		1.17		$(N_1)_{60}$	61.9 ± 3.7	61.0	
CSRN		0.370					

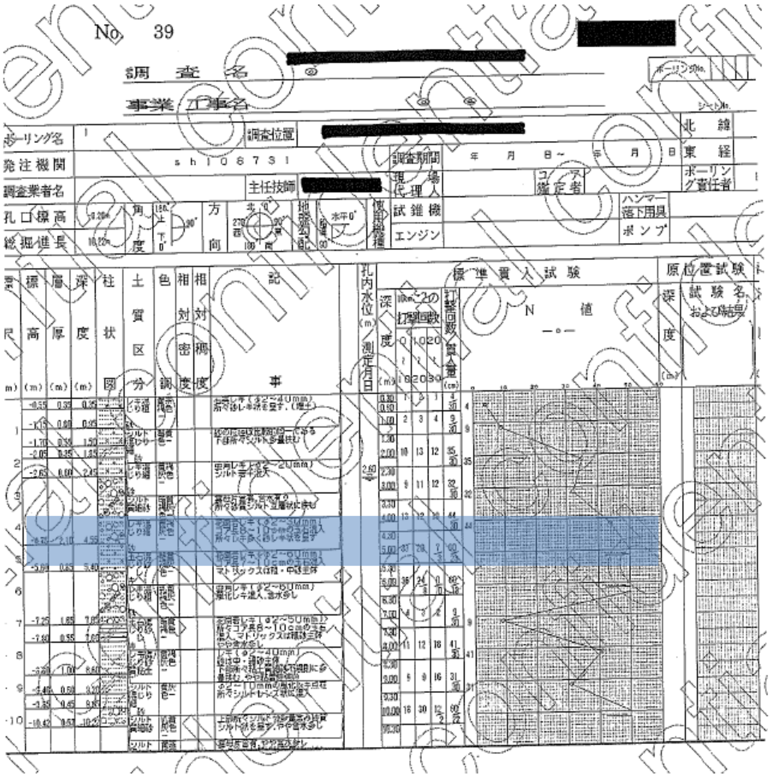

γ_m=120±3 pcf

γ_s=135±3 pcf

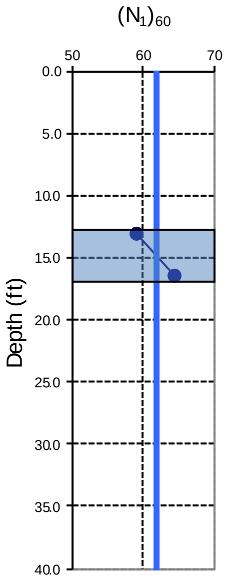

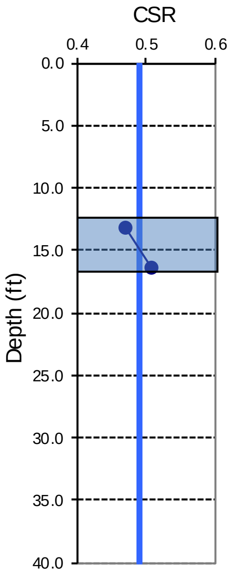

Depth (m)	Depth (ft)	CSR	N	C_N	C_R	$(N1)_{60}$	FC	
4.0	13.1	0.472	44.0	1.25	0.89	*59.3*	*0.0*	
5.0	16.4	0.509	50.0	1.15	0.92	*64.5*	*0.0*	
					**Mean:**	61.9	0.0	
					**St. Dev.**	3.7	2.0	assigned

**Case number:**	198
**Earthquake:**	1995 Hyogoken-Nambu
**Magnitude:**	7.2 (M_L)
**Location:**	Kobe No : 40
**References:**	Tokimatsu, K. Personal Communication (2000)
**Nature of Failure:**	Non-liquefied
**Comments:**	

**Summary of Data**	Cetin et al. (2016)	Idriss&Boulanger (2010)	Seed et.al. (1984)		Cetin et al. (2016)	Idriss&Boulanger (2010)	Seed et.al. (1984)
Liquefied?	No	No		D_{50}	0.000 ± 0.000		
Data Class	B			% Fines	0.0 ± 2.0	0.0	
Critical Depth Range	9.8 - 13.1	11.5		% PI			
Depth to GWT (ft)	9.2 ± 0.3	9.2					
σ_v (psf)	1389.4 ± 74.0	1378.4		N	31.1 ± 7.9	32.5	
σ_v' (psf)	1246.1 ± 47.7	1232.2		C_R	0.87	0.85	
a_{max} (g)	0.600 ± 0.120	0.60		C_S	1.00	1.00	
r_d	1.00 ± 0.052	0.97		C_B	1.00	1.00	
CSR	0.433 ± 0.090	0.421		C_E	1.22	1.22	
Equivalent Magnitude	6.9	6.9		C_N	1.30	1.18	
MSF		1.17		$(N_1)_{60}$	42.8 ± 10.8	39.7	
CSRN		0.326					

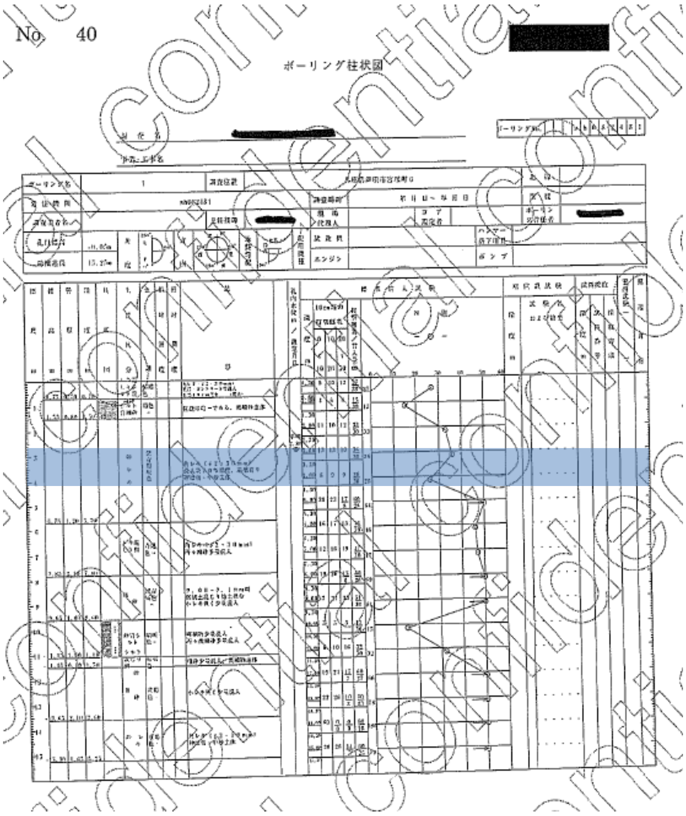

γ_m=120±3 pcf

γ_s=125±3 pcf

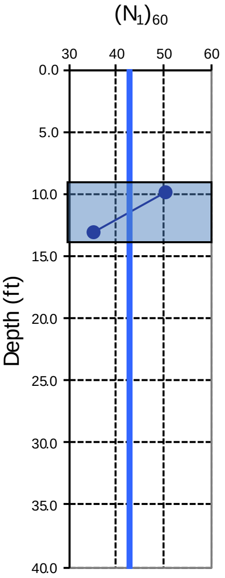

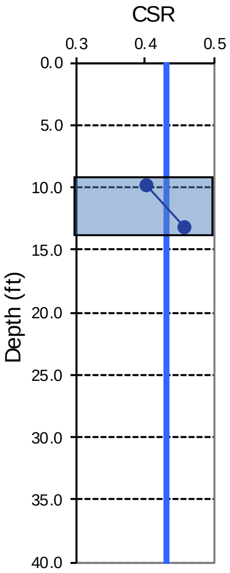

Depth (m)	Depth (ft)	CSR	N	C_N	C_R	$(N1)_{60}$	FC	
3.0	9.8	0.402	36.0	1.36	0.85	***50.4***	***0.0***	
4.0	13.1	0.458	26.0	1.25	0.89	***35.1***	***0.0***	
					**Mean:**	42.8	0.0	
					**St. Dev.**	10.8	2.0	assigned

**Case number:**	199
**Earthquake:**	1995 Hyogoken-Nambu
**Magnitude:**	7.2 (M_L)
**Location:**	Kobe No : 41
**References:**	Tokimatsu, K. Personal Communication (2000)
**Nature of Failure:**	Liquefied
**Comments:**	

Summary of Data

	Cetin et al. (2016)	Idriss&Boulanger (2010)	Seed et.al. (1984)		Cetin et al. (2016)	Idriss&Boulanger (2010)	Seed et.al. (1984)
Liquefied?	Yes	Yes		D_{50}	0.000 ± 0.000		
Data Class	B			% Fines	0.0 ± 2.0	0.0	
Critical Depth Range	7.4 - 19.7	13.5		% PI			
Depth to GWT (ft)	6.6 ± 0.3	6.6					
σ_v (psf)	1492.8 ± 247.8	1482.9		N	9.7 ± 2.0	9.2	
σ_v' (psf)	1057.7 ± 122.2	1044.3		C_R	0.89	0.95	
a_{max} (g)	0.400 ± 0.080	0.40		C_S	1.00	1.00	
r_d	0.96 ± 0.059	0.96		C_B	1.00	1.00	
CSR	0.351 ± 0.076	0.352		C_E	1.22	1.22	
Equivalent Magnitude	6.9	6.9		C_N	1.41	1.41	
MSF		1.17		$(N_1)_{60}$	14.9 ± 3.0	15.0	
CSRN		0.279					

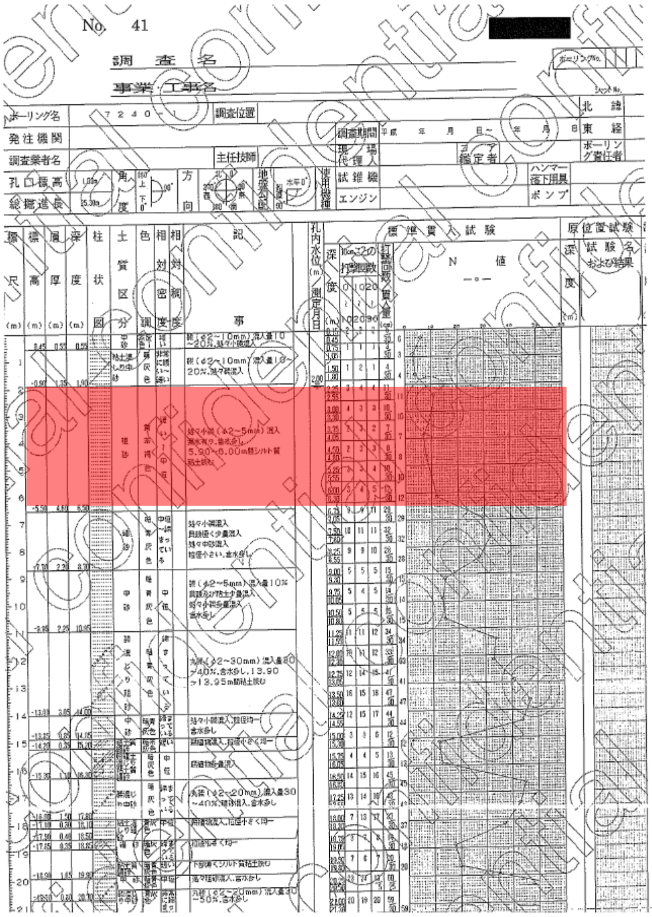

γ_m=100±3 pcf

γ_s=120±3 pcf

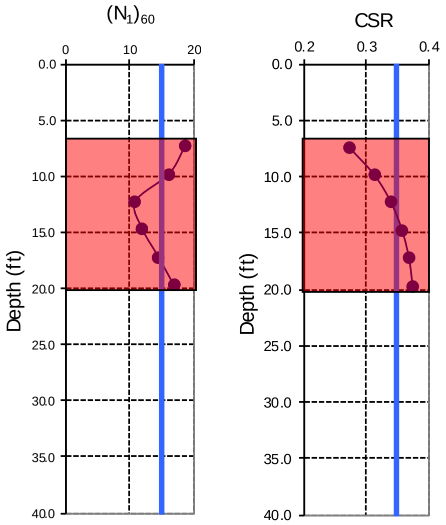

Depth (m)	Depth (ft)	CSR	N	C_N	C_R	$(N1)_{60}$	FC
2.3	7.4	0.274	11.0	1.73	0.81	***18.8***	***0.0***
3.0	9.8	0.314	10.0	1.58	0.85	***16.3***	***0.0***
3.8	12.3	0.341	7.0	1.46	0.88	***10.9***	***0.0***
4.5	14.8	0.358	8.0	1.37	0.90	***12.1***	***0.0***
5.3	17.2	0.369	10.0	1.29	0.93	***14.6***	***0.0***
6.0	19.7	0.375	12.0	1.22	0.95	***17.0***	***0.0***
					**Mean:**	14.9	0.0
					**St. Dev.**	3.0	2.0

**Case number:**	200
**Earthquake:**	1995 Hyogoken-Nambu
**Magnitude:**	7.2 (M_L)
**Location:**	Kobe No : 42
**References:**	Tokimatsu, K. Personal Communication (2000)
**Nature of Failure:**	Liquefied
**Comments:**	

**Summary of Data**	Cetin et al. (2016)	Idriss&Boulanger (2010)	Seed et.al. (1984)		Cetin et al. (2016)	Idriss&Boulanger (2010)	Seed et.al. (1984)
Liquefied?	Yes	Yes		D_{50}	0.000 ± 0.000		
Data Class	B			% Fines	10.0 ± 2.0	10.0	
Critical Depth Range	13.1 - 19.7	16.4		% PI			
Depth to GWT (ft)	3.8 ± 0.3	3.9					
σ_v (psf)	1930.8 ± 137.1	1754.4		N	7.0 ± 0.3	7.0	
σ_v' (psf)	1142.6 ± 76.0	960.7		C_R	0.92	0.95	
a_{max} (g)	0.400 ± 0.080	0.40		C_S	1.00	1.00	
r_d	0.78 ± 0.070	0.94		C_B	1.00	1.00	
CSR	0.343 ± 0.076	0.445		C_E	1.22	1.22	
Equivalent Magnitude	6.9	6.9		C_N	1.36	1.48	
MSF		1.17		$(N_1)_{60}$	10.7 ± 0.5	12.1	
CSRN		0.352					

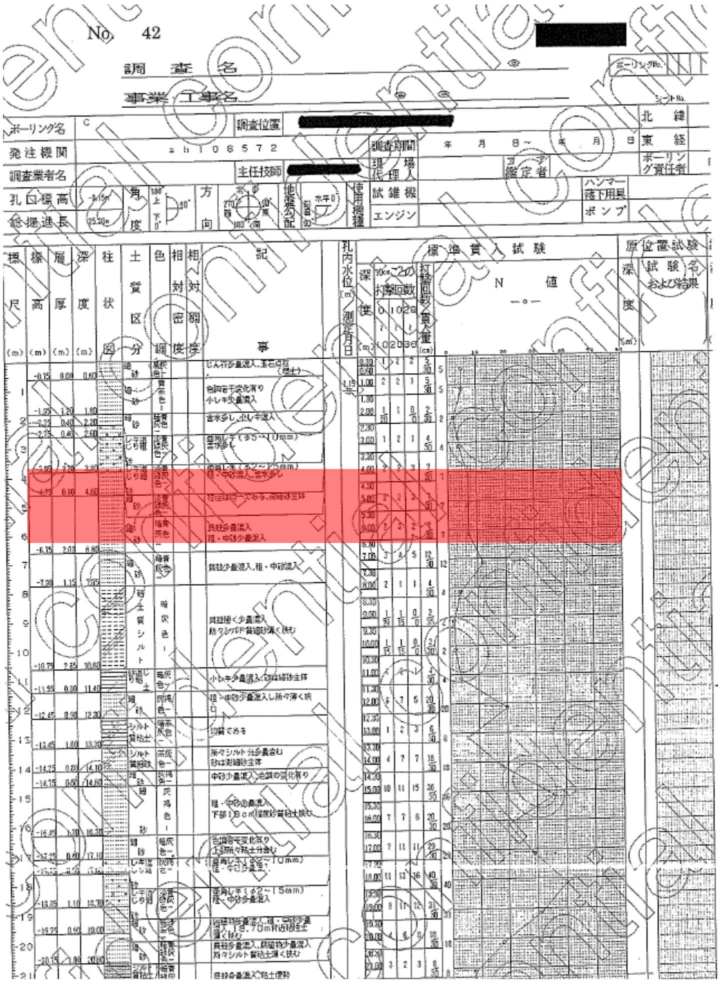

γ_m=110±3 pcf

γ_s=120±3 pcf

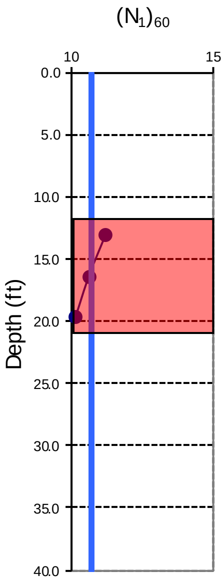

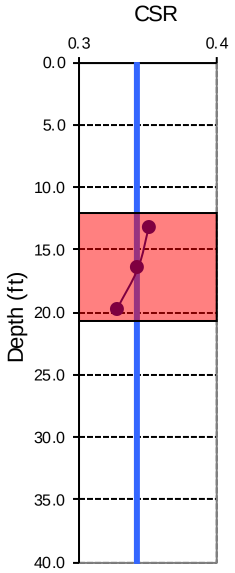

Depth (m)	Depth (ft)	CSR	N	C_N	C_R	$(N1)_{60}$	FC
4.0	13.1	0.351	7.0	1.49	0.89	***11.3***	***10.0***
5.0	16.4	0.343	7.0	1.36	0.92	***10.7***	***10.0***
6.0	19.7	0.329	7.0	1.26	0.95	***10.2***	***10.0***
					**Mean:**	10.7	10.0
					**St. Dev.**	0.5	2.0

**Case number:**	201
**Earthquake:**	1995 Hyogoken-Nambu
**Magnitude:**	7.2 (M_L)
**Location:**	Kobe No : 43
**References:**	Tokimatsu, K. Personal Communication (2000)
**Nature of Failure:**	Liquefied
**Comments:**	

Summary of Data

	Cetin et al. (2016)	Idriss&Boulanger (2010)	Seed et.al. (1984)		Cetin et al. (2016)	Idriss&Boulanger (2010)	Seed et.al. (1984)
Liquefied?	Yes	Yes		D_{50}	0.000 ± 0.000		
Data Class	B			% Fines	20.0 ± 2.0	20.0	
Critical Depth Range	13.8 - 17.1	15.4		% PI			
Depth to GWT (ft)	7.1 ± 0.3	7.2					
σ_v (psf)	1709.3 ± 73.6	1670.8		N	10.0 ± 0.3	10.0	
σ_v' (psf)	1187.3 ± 47.2	1148.7		C_R	0.91	0.95	
a_{max} (g)	0.350 ± 0.070	0.35		C_S	1.00	1.00	
r_d	0.96 ± 0.066	0.95		C_B	1.00	1.00	
CSR	0.314 ± 0.067	0.311		C_E	1.22	1.22	
Equivalent Magnitude	6.9	6.9		C_N	1.34	1.31	
MSF		1.17		$(N_1)_{60}$	14.8 ± 0.5	15.2	
CSRN		0.246					

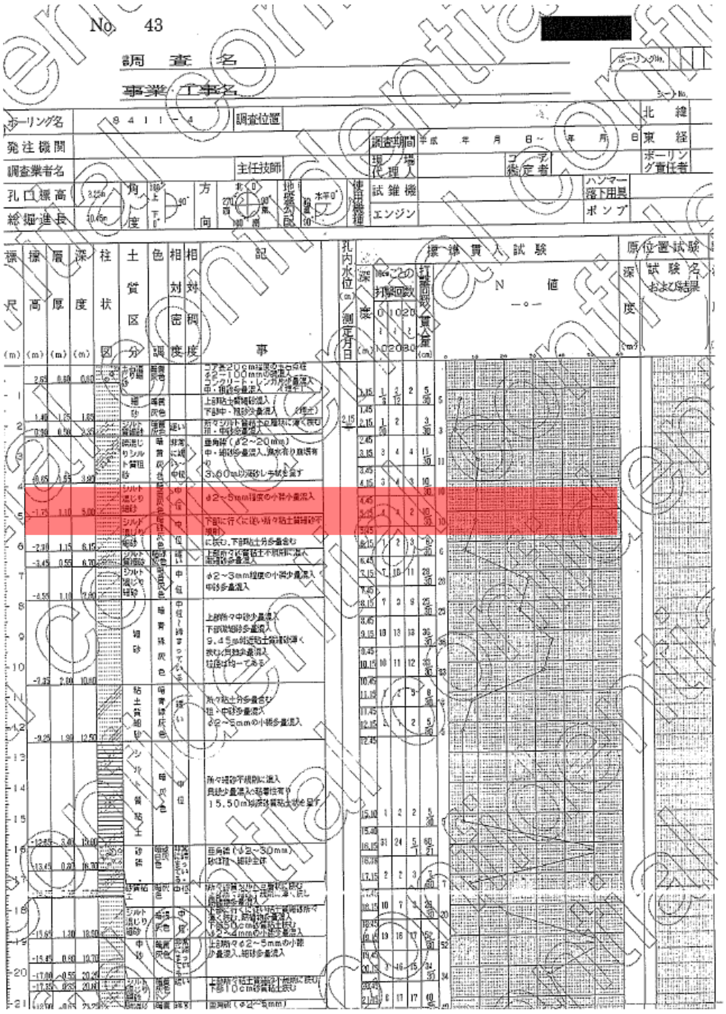

γ_m=100±3 pcf

γ_s=120±3 pcf

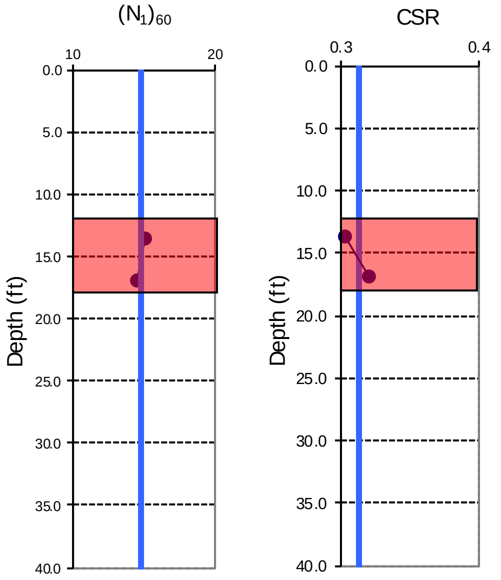

Depth (m)	Depth (ft)	CSR	N	C_N	C_R	$(N1)_{60}$	FC
4.2	13.6	0.303	10.0	1.40	0.89	***15.2***	***20.0***
5.2	16.9	0.321	10.0	1.29	0.93	***14.5***	***20.0***
					**Mean:**	14.8	20.0
					**St. Dev.**	0.5	2.0

assigned

**Case number:**	202
**Earthquake:**	1995 Hyogoken-Nambu
**Magnitude:**	7.2 (M_L)
**Location:**	Kobe No : 44
**References:**	Tokimatsu, K. Personal Communication (2000)
**Nature of Failure:**	Liquefied
**Comments:**	

Summary of Data

	Cetin et al. (2016)	Idriss&Boulanger (2010)	Seed et.al. (1984)		Cetin et al. (2016)	Idriss&Boulanger (2010)	Seed et.al. (1984)
Liquefied?	Yes	Yes		D_{50}	0.000 ± 0.000		
Data Class	B			% Fines	5.0 ± 2.0	5.0	
Critical Depth Range	9.8 - 16.4	13.1		% PI			
Depth to GWT (ft)	5.1 ± 0.3	5.2					
σ_v (psf)	1443.6 ± 123.6	1399.3		N	4.7 ± 1.2	4.4	
σ_v' (psf)	942.0 ± 62.2	898.1		C_R	0.89	0.95	
a_{max} (g)	0.400 ± 0.080	0.40		C_S	1.00	1.00	
r_d	0.90 ± 0.058	0.96		C_B	1.00	1.00	
CSR	0.359 ± 0.076	0.388		C_E	1.22	1.22	
Equivalent Magnitude	6.9	6.9		C_N	1.50	1.61	
MSF		1.17		$(N_1)_{60}$	7.6 ± 1.9	8.3	
CSRN		0.308					

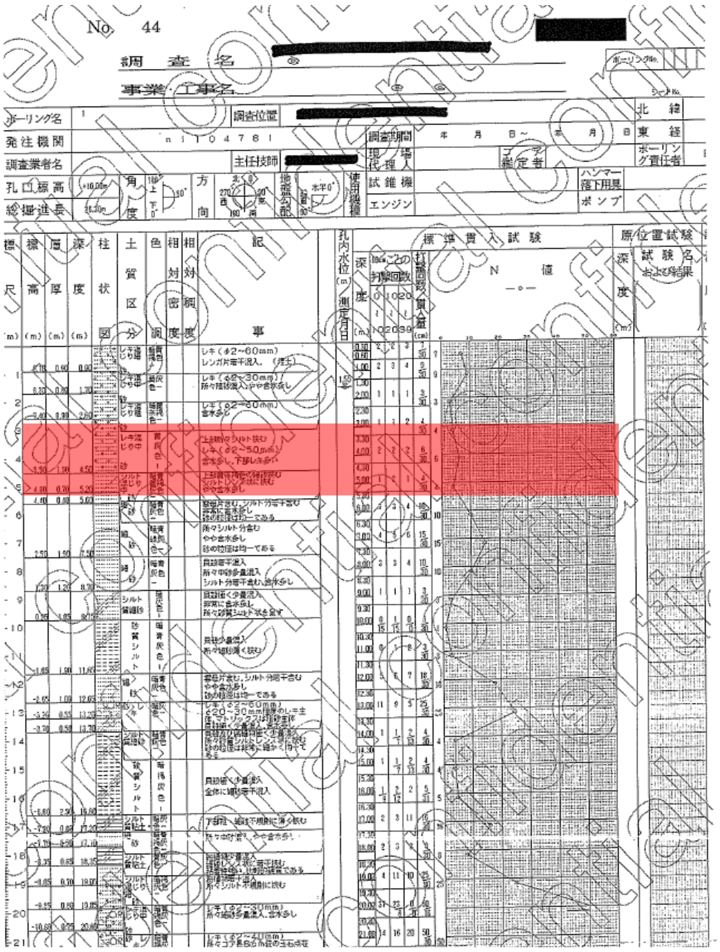

γ_m=110±3 pcf

γ_s=110±3 pcf

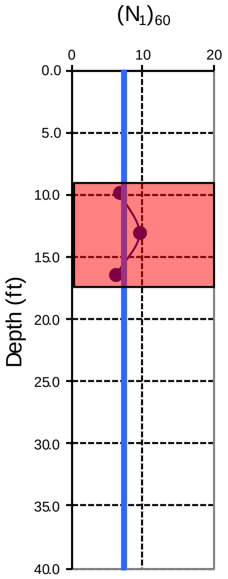

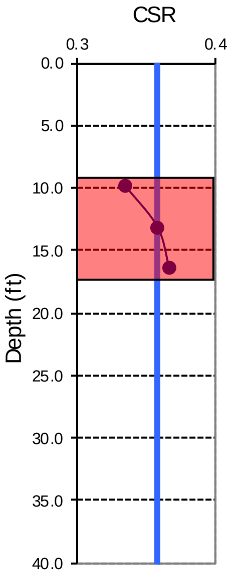

Depth (m)	Depth (ft)	CSR	N	C_N	C_R	$(N1)_{60}$	FC	
3.0	9.8	0.335	4.0	1.64	0.85	***6.8***	***5.0***	
4.0	13.1	0.359	6.0	1.50	0.89	***9.7***	***5.0***	
5.0	16.4	0.368	4.0	1.39	0.92	***6.2***	***5.0***	
					**Mean:**	7.6	5.0	
					**St. Dev.**	1.9	2.0	assigned

**Case number:**	203
**Earthquake:**	1995 Hyogoken-Nambu
**Magnitude:**	7.2 (ML)
**Location:**	Port Island Borehole Array Site
**References:**	Shibata et al.(1996)
**Nature of Failure:**	Densification after earthquake, pore pressure increase, change in frequency content of the strong motion recordings at depths 0 and 16 m. Liquefied.
**Comments:**	On January 17, 1995, an earthquake of magnitude 7.2 (Richter scale) struck the southern part of Hyogo Prefecture. Sand boils and liquefied, ejected soil were observed art numerous sites, mainly in the coastal areas of Hyogo and Osaka Prefectures. In particular, massive liquefaction was observed on man-made islands and in inland areas with high ground water tables.<br>The entire area of the first stage reclaimed land for Port Island and the northern area of Rokko Island were filled mainly with Masado. The first stage reclamation of Port Island was carried out at its northern part of the island from 1966-1980. Rokko Ilsand was constructed from 1972 to 1990. The second stage reclamation of Port Island started in 1986 and would be finished by 1996.<br>Masado is a weathered and decomposed low plasticity, low cohesion, high permeability, erodible granite. It is a sandy soil with much gravel, silt and clay. The mean grain diameter is mostly 0.2-6 mm. The contents of gravel and fines are about 0-65 % and 5-35 % respectively.<br>PGA was recorded as 0.34g at the site. Based on the recordings, it became apparent that the site has liquefied before 6th second. Also the liquefied layer was estimated at depths 0-16 m.<br>The average Japanese SPT energy ratio of 73% is used.

**Summary of Data**	Cetin et al. (2016)	Idriss&Boulanger (2010)	Seed et.al. (1984)		Cetin et al. (2016)	Idriss&Boulanger (2010)	Seed et.al. (1984)
Liquefied?	Yes	Yes		D_{50}	0.400 ± 0.020		
Data Class	B			% Fines	20.0 ± 2.0	20.0	
Critical Depth Range	6.9 - 43.0	25.6		% PI			
Depth to GWT (ft)	7.9 ± 0.3	7.9					
σ_v (psf)	2998.7 ± 754.0	3111.9		N	5.7 ± 1.4	5.7	
σ_v' (psf)	1934.1 ± 381.0	2005.0		C_R	0.99	0.95	
a_{max} (g)	0.340 ± 0.068	0.34		C_S	1.00	1.00	
r_d	0.72 ± 0.100	0.90		C_B	1.00	1.00	
CSR	0.245 ± 0.061	0.307		C_E	1.22	1.22	
Equivalent Magnitude	6.9	6.9		C_N	1.05	1.03	
MSF		1.17		$(N_1)_{60}$	7.1 ± 1.8	6.8	
CSRN		0.260					

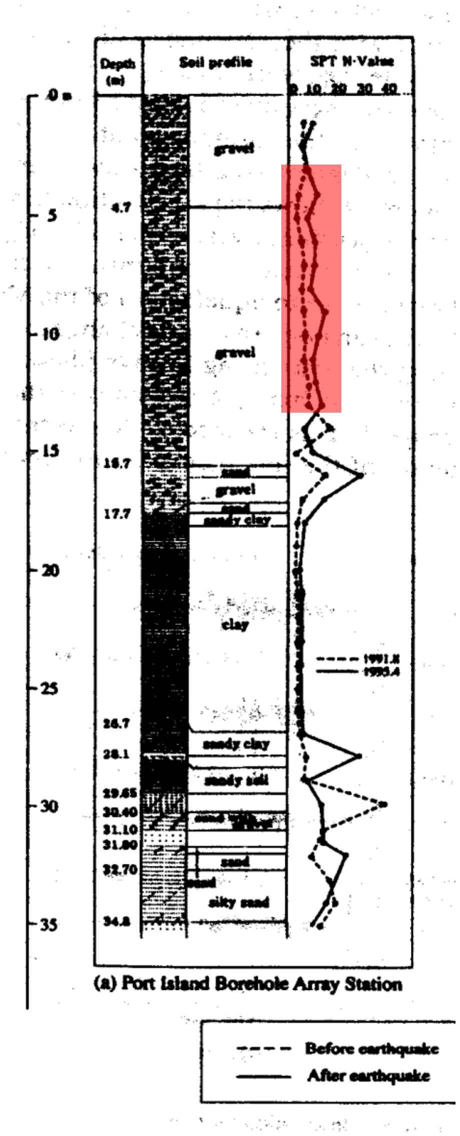

(a) Port Island Borehole Array Station

γ_m=110±3 pcf

γ_s=125±3 pcf

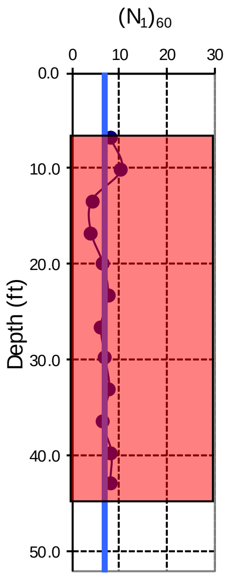

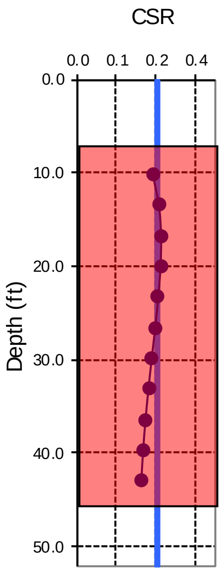

Depth (m)	Depth (ft)	CSR	N	C_N	C_R	$(N1)_{60}$
2.1	6.9	NA	5.0	1.67	0.80	***8.2***
3.1	10.2	0.233	7.0	1.45	0.85	***10.5***
4.1	13.5	0.251	3.0	1.32	0.89	***4.3***
5.1	16.7	0.258	3.0	1.22	0.92	***4.1***
6.1	20.0	0.256	5.0	1.14	0.95	***6.6***
7.1	23.3	0.249	6.0	1.07	0.98	***7.7***
8.1	26.6	0.240	5.0	1.02	1.00	***6.2***
9.1	29.9	0.230	6.0	0.97	1.00	***7.1***
10.1	33.1	0.220	7.0	0.93	1.00	***7.9***
11.1	36.4	0.211	6.0	0.89	1.00	***6.5***
12.1	39.7	0.204	8.0	0.86	1.00	***8.4***
13.1	43.0	0.199	8.0	0.83	1.00	***8.1***
					**Mean:**	7.1
					**St. Dev.**	1.8

**Case number:**	204
**Earthquake:**	1995 Hyogoken-Nambu
**Magnitude:**	7.2 (ML)
**Location:**	Port Island Improved Site (Ikegaya, 80)
**References:**	Yasuda et al.(1996)<br>Ikegaya (1980) in Japanese
**Nature of Failure:**	No subsidence and no damage to structures were observed in the zones densified by rod (vibro) compaction.
**Comments:**	On January 17, 1995, an earthquake of magnitude 7.2 (Richter scale) struck the southern part of Hyogo Prefecture. Sand boils and liquefied, ejected soil were observed at numerous sites, mainly in the coastal areas of Hyogo and Osaka Prefectures. In particular, massive liquefaction was observed on man-made islands and in inland areas with high ground water tables.<br>The entire area of the first stage reclaimed land for Port Island and the northern area of Rokko Island were filled mainly with Masado. The first stage reclamation of Port Island was carried out at its northern part of the island from 1966-1980. Rokko Island was constructed from 1972 to 1990. The second stage reclamation of Port Island started in 1986 and would be finished by 1996.<br>The improved site is located on Port Island.<br>Masado is a weathered and decomposed low plasticity, low cohesion, high permeability, erodible granite. It is a sandy soil with much gravel, silt and clay. The mean grain diameter is mostly 0.2-6 mm. The contents of gravel and fines are about 0-65 % and 5-35 % respectively.<br>PGA was recorded as 0.34g at Port Island. The recorded motion was obtained at a liquefied site. Liquefaction of the site is believed not affected the PGA significantly because the fling was recorded at very early stages of the shaking.<br>The water table is at depth 4-6 m at the island.<br>The average Japanese SPT energy ratio of 73% is used.

**Summary of Data**	Cetin et al. (2016)	Idriss&Boulanger (2010)	Seed et.al. (1984)		Cetin et al. (2016)	Idriss&Boulanger (2010)	Seed et.al. (1984)
Liquefied?	No	No		D_{50}	0.400 ± 0.050		
Data Class	B			% Fines	20.0 ± 2.0	20.0	
Critical Depth Range	16.4 - 39.4	27.9		% PI			
Depth to GWT (ft)	16.4 ± 0.3	16.4					
σ_v (psf)	3239.8 ± 482.2	3320.8		N	20.3 ± 3.8	20.2	
σ_v' (psf)	2523.3 ± 247.4	2610.7		C_R	1.00	1.00	
a_{max} (g)	0.400 ± 0.080	0.40		C_S	1.00	1.00	
r_d	0.87 ± 0.110	0.88		C_B	1.00	1.00	
CSR	0.291 ± 0.071	0.293		C_E	1.22	1.22	
Equivalent Magnitude	6.9	6.9		C_N	0.92	0.92	
MSF		1.17		$(N_1)_{60}$	22.6 ± 4.2	22.7	
CSRN		0.260					

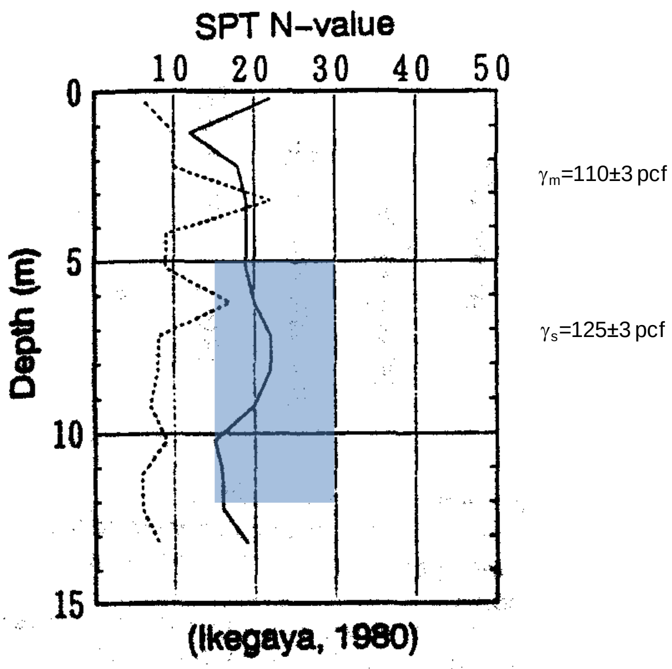

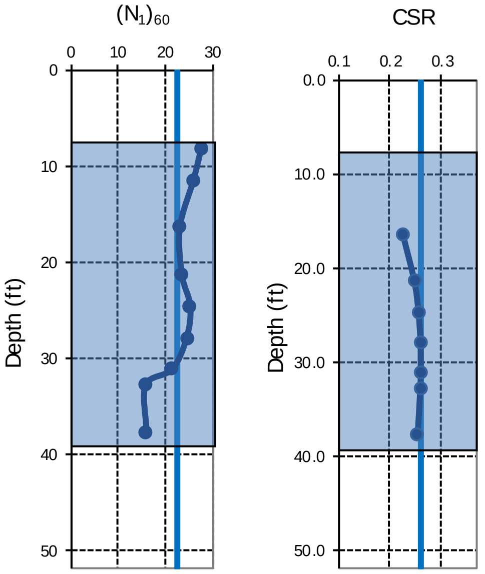

Depth (m)	Depth (ft)	CSR	N	C_N	C_R	$(N1)_{60}$
2.5	8.2	NA	18.0	1.53	0.82	***27.6***
3.5	11.5	NA	19.0	1.29	0.87	***26.0***
5.0	16.4	0.249	19.0	1.08	0.92	***23.1***
6.5	21.3	0.276	20.0	1.00	0.96	***23.5***
7.5	24.6	0.286	22.0	0.96	0.99	***25.3***
8.5	27.9	0.291	22.0	0.92	1.00	***24.5***
9.5	31.2	0.292	20.0	0.88	1.00	***21.4***
10.0	32.8	0.291	15.0	0.86	1.00	***15.8***
11.5	37.7	0.285	16.0	0.82	1.00	***16.0***
					**Mean:**	22.6
					**St. Dev.**	4.2

**Case number:**	205
**Earthquake:**	1995 Hyogoken-Nambu
**Magnitude:**	7.2 (M_L)
**Location:**	Port Island Improved Site (Tanahashi 87)
**References:**	Yasuda et al.(1996)<br>Tanahashi (1978) in Japanese
**Nature of Failure:**	No subsidence and no damage to structures were observed in the zones densified by rod (vibro) compaction.
**Comments:**	On January 17, 1995, an earthquake of magnitude 7.2 (Richter scale) struck the southern part of Hyogo Prefecture. Sand boils and liquefied, ejected soil were observed at numerous sites, mainly in the coastal areas of Hyogo and Osaka Prefectures. In particular, massive liquefaction was observed on man-made islands and in inland areas with high ground water tables.<br>The entire area of the first stage reclaimed land for Port Island and the northern area of Rokko Island were filled mainly with Masado. The first stage reclamation of Port Island was carried out at its northern part of the island from 1966-1980. Rokko Ilsand was constructed from 1972 to 1990. The second stage reclamation of Port Island started in 1986 and would be finished by 1996.<br>The improved site is located on Port Island.<br>Masado is a weathered and decomposed low plasticity, low cohesion, high permeability, erodible granite. It is a sandy soil with much gravel, silt and clay. The mean grain diameter is mostly 0.2-6 mm. The contents of gravel and fines are about 0-65 % and 5-35 % respectively.<br>PGA was recorded as 0.34g at Port Island. The recorded motion was obtained at a liquefied site. Liquefaction of the site is believed not affected the PGA significantly because the fling was recorded at very early stages of the shaking.<br>The water table is at depth 4-6 m at the island.<br>The average Japanese SPT energy ratio of 73% is used.

Summary of Data

	Cetin et al. (2016)	Idriss&Boulanger (2010)	Seed et.al. (1984)		Cetin et al. (2016)	Idriss&Boulanger (2010)	Seed et.al. (1984)
Liquefied?	No	No		D_{50}	0.400 ± 0.050		
Data Class	B			% Fines	20.0 ± 2.0	20.0	
Critical Depth Range	16.4 - 49.2	32.8		% PI			
Depth to GWT (ft)	16.4 ± 0.3	16.4					
σ_v (psf)	3855.0 ± 687.1	3947.3		N	18.2 ± 3.2	18.2	
σ_v' (psf)	2831.4 ± 349.6	2924.0		C_R	1.00	1.00	
a_{max} (g)	0.400 ± 0.080	0.40		C_S	1.00	1.00	
r_d	0.82 ± 0.126	0.86		C_B	1.00	1.00	
CSR	0.291 ± 0.075	0.301		C_E	1.22	1.22	
Equivalent Magnitude	6.9	6.9		C_N	0.86	0.88	
MSF		1.17		$(N_1)_{60}$	19.1 ± 3.4	19.5	
CSRN		0.270					

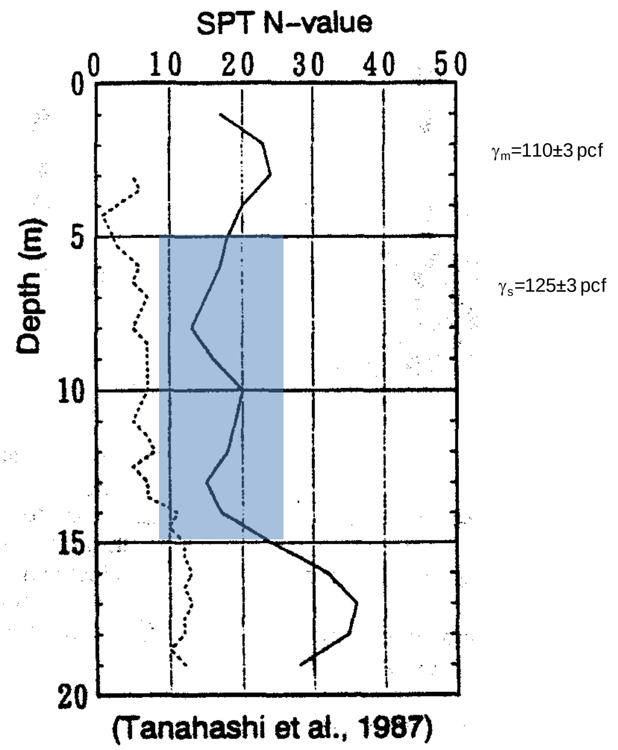

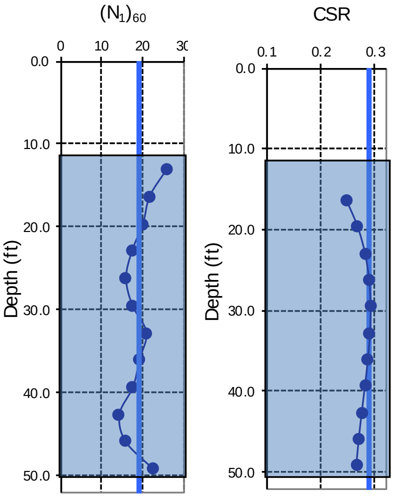

Depth (m)	Depth (ft)	CSR	N	C_N	C_R	$(N1)_{60}$
4.0	13.1	NA	20.0	1.21	0.89	***26.1***
5.0	16.4	0.249	18.0	1.08	0.92	***21.8***
6.0	19.7	0.269	17.0	1.03	0.95	***20.2***
7.0	23.0	0.282	15.0	0.98	0.98	***17.4***
8.0	26.2	0.290	14.0	0.94	1.00	***15.9***
9.0	29.5	0.292	16.0	0.90	1.00	***17.5***
10.0	32.8	0.291	20.0	0.86	1.00	***21.0***
11.0	36.1	0.287	19.0	0.83	1.00	***19.3***
12.0	39.4	0.282	18.0	0.81	1.00	***17.7***
13.0	42.7	0.276	15.0	0.78	1.00	***14.3***
14.0	45.9	0.271	17.0	0.76	1.00	***15.7***
15.0	49.2	0.266	25.0	0.74	1.00	***22.5***
					**Mean:**	19.1
					**St. Dev.**	3.4

**Case number:**	206
**Earthquake:**	1995 Hyogoken-Nambu
**Magnitude:**	7.2 (ML)
**Location:**	Port Island Improved Site (Watanabe 81)
**References:**	Yasuda et al.(1996)<br>Tanahashi (1978) in Japanese
**Nature of Failure:**	No subsidence and no damage to structures were observed in the zones densified by rod (vibro) compaction.
**Comments:**	On January 17, 1995, an earthquake of magnitude 7.2 (Richter scale) struck the southern part of Hyogo Prefecture. Sand boils and liquefied, ejected soil were observed at numerous sites, mainly in the coastal areas of Hyogo and Osaka Prefectures. In particular, massive liquefaction was observed on man-made islands and in inland areas with high ground water tables.<br>The entire area of the first stage reclaimed land for Port Island and the northern area of Rokko Island were filled mainly with Masado. The first stage reclamation of Port Island was carried out at its northern part of the island from 1966-1980. Rokko Island was constructed from 1972 to 1990. The second stage reclamation of Port Island started in 1986 and would be finished by 1996.<br>The improved site is located on Port Island.<br>Masado is a weathered and decomposed low plasticity, low cohesion, high permeability, erodible granite. It is a sandy soil with much gravel, silt and clay. The mean grain diameter is mostly 0.2-6 mm. The contents of gravel and fines are about 0-65 % and 5-35 % respectively.<br>PGA was recorded as 0.34 g at Port Island. The recorded motion was obtained at a liquefied site. Liquefaction of the site is believed not affected the PGA significantly because the fling was recorded at very early stages of the shaking.<br>The water table is at depth 4-6 m at the island.<br>The average Japanese SPT energy ratio of 73% is used.

Summary of Data

	Cetin et al. (2016)	Idriss&Boulanger (2010)	Seed et.al. (1984)		Cetin et al. (2016)	Idriss&Boulanger (2010)	Seed et.al. (1984)
Liquefied?	No	No		D_{50}	0.400 ± 0.050		
Data Class	B			% Fines	20.0 ± 2.0	20.0	
Critical Depth Range	16.4 - 45.9	31.2		% PI			
Depth to GWT (ft)	16.4 ± 0.3	16.4					
σ_v (psf)	3723.8 ± 643.2	3738.5		N	31.0 ± 6.7	30.9	
σ_v' (psf)	2802.5 ± 339.4	2819.5		C_R	1.00	1.00	
a_{max} (g)	0.400 ± 0.080	0.40		C_S	1.00	1.00	
r_d	0.96 ± 0.121	0.87		C_B	1.00	1.00	
CSR	0.332 ± 0.080	0.299		C_E	1.22	1.22	
Equivalent Magnitude	6.9	6.9		C_N	0.87	0.92	
MSF		1.17		$(N_1)_{60}$	32.8 ± 7.1	34.6	
CSRN		0.278					

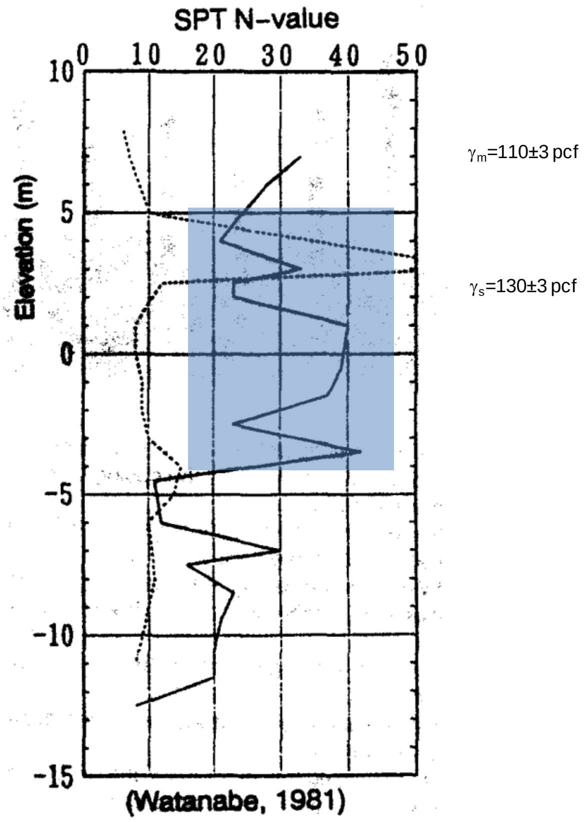

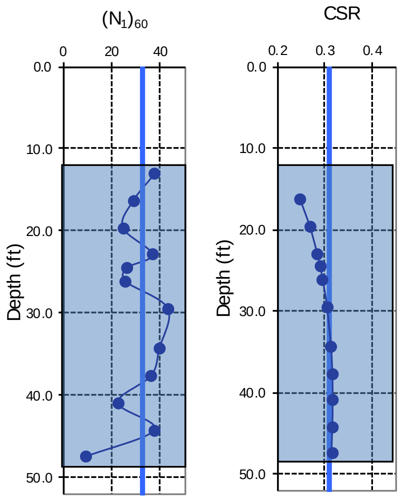

Depth (m)	Depth (ft)	CSR	N	C_N	C_R	$(N1)_{60}$	
4.0	13.1	NA	29.0	1.21	0.89	***37.9***	
5.0	16.4	0.258	24.0	1.08	0.92	***29.1***	
6.0	19.7	0.283	21.0	1.02	0.95	***24.8***	
7.0	23.0	0.302	32.0	0.97	0.98	***36.9***	
7.5	24.6	0.310	23.0	0.95	0.99	***26.2***	
8.0	26.2	0.317	23.0	0.93	1.00	***25.9***	
9.0	29.5	0.328	40.0	0.89	1.00	***43.2***	
10.5	34.4	0.338	39.0	0.84	1.00	***39.7***	
11.5	37.7	0.342	37.0	0.81	1.00	***36.3***	
12.5	41.0	0.343	24.0	0.78	1.00	***22.8***	
13.5	44.3	0.342	41.0	0.76	1.00	***37.8***	
14.5	47.6	0.340	11.0	0.74	1.00	9.8	clay layer
					**Mean:**	32.8	
					**St. Dev.**	7.1	

**Case number:**	207
**Earthquake:**	1995 Hyogoken-Nambu
**Magnitude:**	7.2 (M_L)
**Location:**	Port Island Site I
**References:**	Tokimatsu et al. (1996)
**Nature of Failure:**	Up to 50 cm of settlements; sand boils
**Comments:**	On January 17, 1995, an earthquake of magnitude 7.2 (Richter scale) struck the southern part of Hyogo Prefecture. Sand boils and liquefied, ejected soil were observed at numerous sites, mainly in the coastal areas of Hyogo and Osaka Prefectures. In particular, massive liquefaction was observed on man-made islands and in inland areas with high ground water tables.<br>The entire area of the first stage reclaimed land for Port Island and the northern area of Rokko Island were filled mainly with Masado. The first stage reclamation of Port Island was carried out at its northern part of the island from 1966-1980. Rokko Island was constructed from 1972 to 1990. The second stage reclamation of Port Island started in 1986 and would be finished by 1996.<br>The improved site is located on Port Island.<br>Masado is a weathered and decomposed low plasticity, low cohesion, high permeability, erodible granite. It is a sandy soil with much gravel, silt and clay. The mean grain diameter is mostly 0.2-6 mm. The contents of gravel and fines are about 0-65 % and 5-35 % respectively.<br>PGA was recorded as 0.34g at Port Island. The recorded motion was obtained at a liquefied site. Liquefaction of the site is believed not to affect the PGA because the fling was recorded at very early stages of the shaking.<br>The water table is at depth 4-6 m at the island.<br>The average Japanese SPT energy ratio of 73% is used.

**Summary of Data**	Cetin et al. (2016)	Idriss&Boulanger (2010)	Seed et.al. (1984)		Cetin et al. (2016)	Idriss&Boulanger (2010)	Seed et.al. (1984)
Liquefied?	Yes	Yes		D_{50}	0.400 ± 0.050		
Data Class	B			% Fines	20.0 ± 2.0	20.0	
Critical Depth Range	19.7 - 45.9	32.8		% PI			
Depth to GWT (ft)	9.8 ± 0.3	9.8					
σ_v (psf)	3838.6 ± 530.3	4010.0		N	9.7 ± 1.6	9.7	
σ_v' (psf)	2405.5 ± 263.4	2568.9		C_R	1.00	1.00	
a_{max} (g)	0.340 ± 0.068	0.34		C_S	1.00	1.00	
r_d	0.78 ± 0.126	0.86		C_B	1.00	1.00	
CSR	0.274 ± 0.071	0.295		C_E	1.22	1.22	
Equivalent Magnitude	6.9	6.9		C_N	0.94	0.91	
MSF		1.17		$(N_1)_{60}$	11.1 ± 1.8	10.8	
CSRN		0.258					

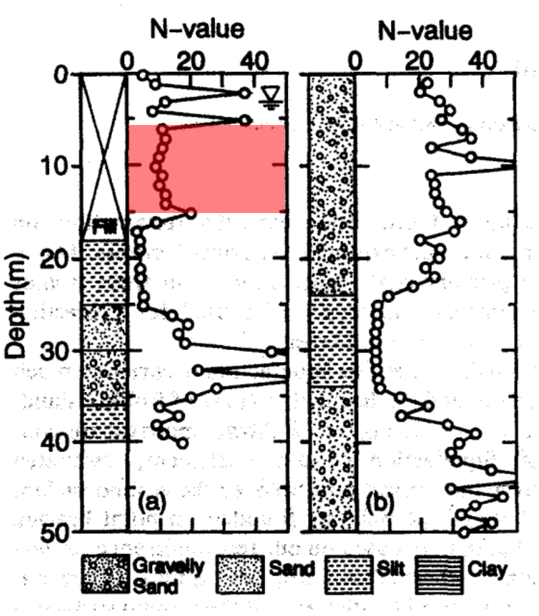

γ_m=110±3 pcf

γ_s=120±3 pcf

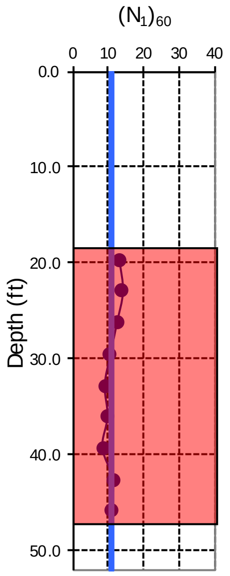

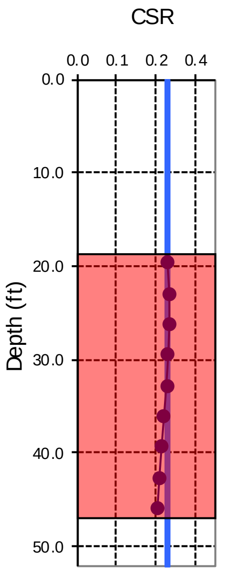

Depth (m)	Depth (ft)	CSR	N	C_N	C_R	$(N1)_{60}$
6.0	19.7	0.277	10.0	1.13	0.95	***13.1***
7.0	23.0	0.283	11.0	1.07	0.98	***14.0***
8.0	26.2	0.283	10.0	1.02	1.00	***12.4***
9.0	29.5	0.280	9.0	0.98	1.00	***10.7***
10.0	32.8	0.274	8.0	0.94	1.00	***9.1***
11.0	36.1	0.267	9.0	0.90	1.00	***9.9***
12.0	39.4	0.259	8.0	0.87	1.00	***8.5***
13.0	42.7	0.252	11.0	0.84	1.00	***11.3***
14.0	45.9	0.246	11.0	0.82	1.00	***10.9***
					**Mean:**	11.1
					**St. Dev.**	1.8

**Case number:**	208
**Earthquake:**	1995 Hyogoken-Nambu
**Magnitude:**	7.2 (M_L)
**Location:**	Rokko Island Building D
**References:**	Tokimatsu et al. (1996)
**Nature of Failure:**	Ground settlement, tilted structures
**Comments:**	On January 17, 1995, an earthquake of magnitude 7.2 (Richter scale) struck the southern part of Hyogo Prefecture. Sand boils and liquefied, ejected soil were observed at numerous sites, mainly in the coastal areas of Hyogo and Osaka Prefectures. In particular, massive liquefaction was observed on man-made islands and in inland areas with high ground water tables.<br>The entire area of the first stage reclaimed land for Port Island and the northern area of Rokko Island were filled mainly with Masado. The first stage reclamation of Port Island was carried out at its northern part of the island from 1966-1980. Rokko Island was constructed from 1972 to 1990. The second stage reclamation of Port Island started in 1986 and would be finished by 1996.<br>The southern part of Rokko Island consist of mainly of crsuhed tuff and sand-stone of the Kobe and Osaka Groups from Rokko Mountains. The soils of southern Rokko Island includes more fines and clay, i.e about 20 % and 10 % compared with about 15 % and 5 % of decomposed granite (Masado).<br>Building D is located in the southern part of Rokko Island.<br>Site has been improved by sand compaction piles.<br>Masado is a weathered and decomposed low plasticity, low cohesion, high permeability, erodible granite. It is a sandy soil with much gravel, silt and clay. The mean grain diameter is mostly 0.2-6 mm. The contents of gravel and fines are about 0-65 % and 5-35 % respectively.<br>PGA was recorded as 0.34g at Port Island. The recorded motion was obtained at a liquefied site. Liquefaction of the site is believed not affected the PGA significantly because the fling was recorded at very early stages of the shaking.<br>Rokko Island is predicted to have experienced the same level of shaking<br>The water table is at depth 4-6 m at the island.<br>The average Japanese SPT energy ratio of 73% is used.

Summary of Data

	Cetin et al. (2016)	Idriss&Boulanger (2010)	Seed et.al. (1984)		Cetin et al. (2016)	Idriss&Boulanger (2010)	Seed et.al. (1984)
Liquefied?	Yes	Yes		D_{50}	0.800 ± 0.050		
Data Class	B			% Fines	25.0 ± 2.0	25.0	
Critical Depth Range	13.1 - 36.1	24.6		% PI			
Depth to GWT (ft)	13.1 ± 0.3	13.1					
σ_v (psf)	2878.9 ± 481.3	2944.8		N	14.8 ± 6.0	14.8	
σ_v' (psf)	2162.4 ± 245.7	2234.7		C_R	0.99	0.95	
a_{max} (g)	0.400 ± 0.080	0.40		C_S	1.00	1.00	
r_d	0.93 ± 0.099	0.90		C_B	1.00	1.00	
CSR	0.322 ± 0.075	0.310		C_E	1.22	1.22	
Equivalent Magnitude	6.9	6.9		C_N	0.99	0.98	
MSF		1.17		$(N_1)_{60}$	17.6 ± 7.1	16.8	
CSRN		0.267					

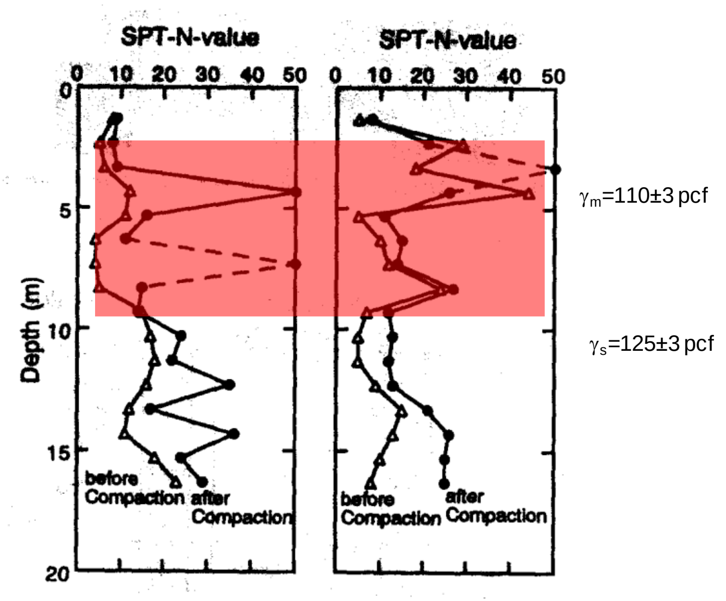

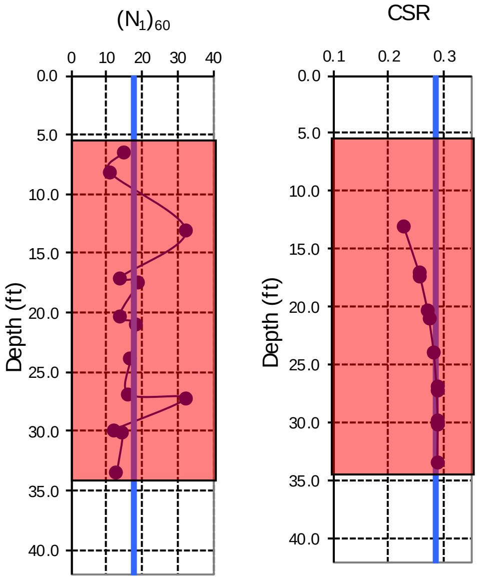

Depth (m)	Depth (ft)	CSR	N	C_N	C_R	$(N1)_{60}$	
2.0	6.6	NA	9.0	1.71	0.80	***15.0***	1
2.5	8.2	NA	7.0	1.53	0.82	***10.7***	2
4.0	13.1	0.255	25.0	1.21	0.89	***32.7***	2
5.2	17.1	0.288	11.0	1.12	0.93	***13.9***	2
5.3	17.4	0.291	15.0	1.11	0.93	***18.9***	1
6.2	20.3	0.307	11.0	1.06	0.96	***13.5***	1
6.4	21.0	0.310	15.0	1.05	0.96	***18.3***	2
7.3	24.0	0.321	14.0	1.00	0.98	***16.7***	2
8.2	26.9	0.327	14.0	0.96	1.00	***16.3***	1
8.3	27.2	0.327	28.0	0.95	1.00	***32.5***	2
9.1	29.9	0.329	11.0	0.92	1.00	***12.3***	2
9.2	30.2	0.330	13.0	0.92	1.00	***14.5***	1
10.2	33.5	0.328	12.0	0.88	1.00	***12.9***	2
					**Mean:**	17.6	
					**St. Dev.**	7.1	

**Case number:**	209
**Earthquake:**	1995 Hyogoken-Nambu
**Magnitude:**	7.2 (M_L)
**Location:**	Rokko Island Site G
**References:**	Tokimatsu et al. (1996)
**Nature of Failure:**	Up to 50 cm of settlements; sand boils
**Comments:**	On January 17, 1995, an earthquake of magnitude 7.2 (Richter scale) struck the southern part of Hyogo Prefecture. Sand boils and liquefied, ejected soil were observed at numerous sites, mainly in the coastal areas of Hyogo and Osaka Prefectures. In particular, massive liquefaction was observed on man-made islands and in inland areas with high ground water tables.<br>The entire area of the first stage reclaimed land for Port Island and the northern area of Rokko Island were filled mainly with Masado. The first stage reclamation of Port Island was carried out at its northern part of the island from 1966-1980. Rokko Island was constructed from 1972 to 1990. The second stage reclamation of Port Island started in 1986 and would be finished by 1996.<br>The southern part of Rokko Island consist of mainly of crsuhed tuff and sand-stone of the Kobe and Osaka Groups from Rokko Mountains. The soils of southern Rokko Island includes more fines and clay, i.e about 20 % and 10 % compared with about 15 % and 5 % of decomposed granite (Masado).<br>Site G is located in the northern part of Rokko Island.<br>Masado is a weathered and decomposed low plasticity, low cohesion, high permeability, erodible granite. It is a sandy soil with much gravel, silt and clay. The mean grain diameter is mostly 0.2-6 mm. The contents of gravel and fines are about 0-65 % and 5-35 % respectively.<br>PGA was recorded as 0.34g at Port Island. The recorded motion was obtained at a liquefied site. Liquefaction of the site is believed not affected the PGA significantly because the fling was recorded at very early stages of the shaking.<br>Rokko Island is predicted to have experienced the same level of shaking<br>The water table is at depth 4-6 m at the island.<br>The average Japanese SPT energy ratio of 73% is used.

**Summary of Data**	Cetin et al. (2016)	Idriss&Boulanger (2010)	Seed et.al. (1984)		Cetin et al. (2016)	Idriss&Boulanger (2010)	Seed et.al. (1984)
Liquefied?	Yes	Yes		D_{50}	0.400 ± 0.050		
Data Class	B			% Fines	20.0 ± 2.0	20.0	
Critical Depth Range	13.1 - 62.3	37.7		% PI			
Depth to GWT (ft)	13.1 ± 0.3	13.1					
σ_v (psf)	4396.3 ± 987.8	4573.9		N	12.0 ± 3.4	12.0	
σ_v' (psf)	2860.9 ± 480.0	3049.3		C_R	1.00	1.00	
a_{max} (g)	0.340 ± 0.068	0.34		C_S	1.00	1.00	
r_d	0.72 ± 0.142	0.83		C_B	1.00	1.00	
CSR	0.245 ± 0.070	0.275		C_E	1.22	1.22	
Equivalent Magnitude	6.9	6.9		C_N	0.86	0.84	
MSF		1.17		$(N_1)_{60}$	12.5 ± 3.6	12.3	
CSRN		0.246					

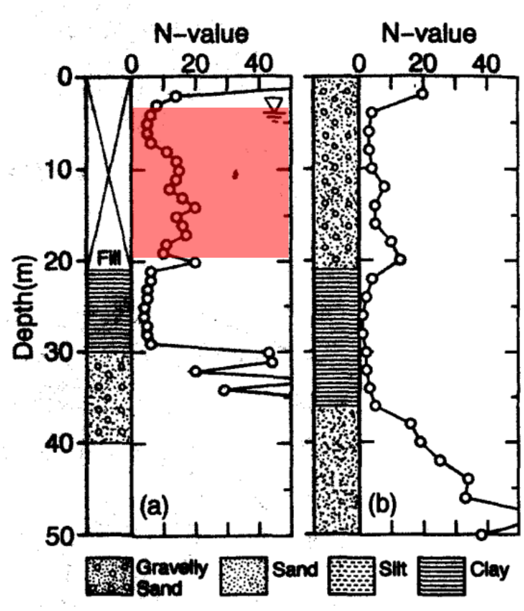

γ_m=110±3 pcf

γ_s=120±3 pcf

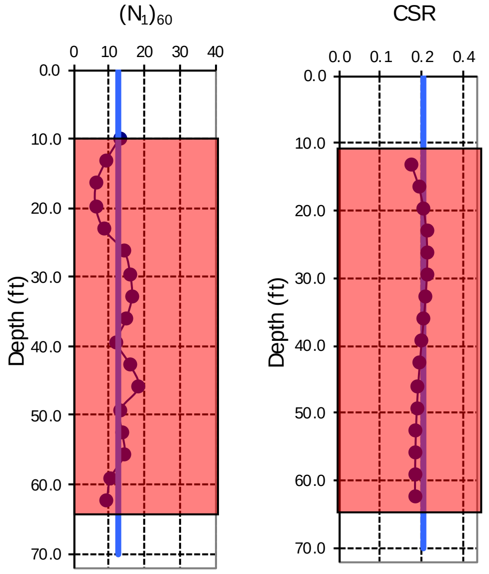

Depth (m)	Depth (ft)	CSR	N	C_N	C_R	$(N1)_{60}$
3.0	9.8	NA	9.0	1.40	0.85	***13.0***
4.0	13.1	0.212	7.0	1.21	0.89	***9.1***
5.0	16.4	0.234	5.0	1.14	0.92	***6.4***
6.0	19.7	0.248	5.0	1.08	0.95	***6.2***
7.0	23.0	0.256	7.0	1.03	0.98	***8.5***
8.0	26.2	0.258	12.0	0.98	1.00	***14.3***
9.0	29.5	0.257	14.0	0.94	1.00	***16.0***
10.0	32.8	0.254	15.0	0.91	1.00	***16.5***
11.0	36.1	0.248	14.0	0.87	1.00	***14.9***
12.0	39.4	0.242	12.0	0.85	1.00	***12.4***
13.0	42.7	0.237	16.0	0.82	1.00	***16.0***
14.0	45.9	0.232	19.0	0.80	1.00	***18.4***
15.0	49.2	0.228	14.0	0.78	1.00	***13.2***
16.0	52.5	0.225	15.0	0.76	1.00	***13.8***
17.0	55.8	0.224	16.0	0.74	1.00	***14.3***
18.0	59.1	0.223	12.0	0.72	1.00	***10.5***
19.0	62.3	0.222	11.0	0.70	1.00	***9.4***
					**Mean:**	12.5
					**St. Dev.**	3.6

**Case number:**	210
**Earthquake:**	1995 Hyogoken-Nambu
**Magnitude:**	7.2 (M_L)
**Location:**	Torishima Dike
**References:**	Matsuo (1996)
**Nature of Failure:**	Failed dike; sand boils near dike.
**Comments:**	On January 17, 1995, an earthquake of magnitude 7.2 (Richter scale) struck the southern part of Hyogo Prefecture. Sand boils and liquefied, ejected soil were observed at numerous sites, mainly in the coastal areas of Hyogo and Osaka Prefectures. In particular, massive liquefaction was observed on man-made islands and in inland areas with high ground water tables.<br>The most severely damaged were the dikes of Yodo-gawa, which, flowing through Osaka is the largest river in Kansai area. A length of 2 km of Torishima Dike, the left levee of Yodo-gawa, was severely damaged. The dike was designed to protect against high sea tide of 5.2 m above mean sea level. The core of the dike was a soil embankment, and the river side surface was protected by concrete parapet wall, the design height of which was 8.1 m from the sea level.<br>The dike is underlain by Holocene deposit with a thickness of 30 m, called the Umeda layer. The deposit consists of, from the top to bottom, the upper sandy layer (As2), clay layer (Ac), and the lower sandy layer (As11). The thickness of Holocene deposit decreases downstream. The As2 layer is also divided by the upper (As2-2) and lower (As2-1) layer. The sand boils observed after the quake are considered to have been ejected from the upper sandy layer.<br>The average of the main grain size and fines content of the As2 layer are 0.2-0.3 mm and 20 % respectively. Cyclic undrain TX compression tests were made on samples from the depth 8.0-8.6 m. Triaxial cyclic stress ratio on frozen samples required to cause 5 % axial starin in 20 cycles is about 0.3.<br>PGA was recorded along Yodo River at many stations. It ranges from 0.15-0.30 g mostly concentrating around 0.22-0.26g. A value of 0.25 g was adopted for the analysis.<br>The borings were obtained in February and March 1995, before the earthquake.<br>The average Japanese SPT energy ratio of 73% is used.

**Summary of Data**	Cetin et al. (2016)	Idriss&Boulanger (2010)	Seed et.al. (1984)		Cetin et al. (2016)	Idriss&Boulanger (2010)	Seed et.al. (1984)
Liquefied?	Yes	Yes		D_{50}	0.200 ± 0.050		
Data Class	B			% Fines	20.0 ± 2.0	20.0	
Critical Depth Range	9.8 - 21.3	15.4		% PI			
Depth to GWT (ft)	0.0 ± 0.3	0.0					
σ_v (psf)	1792.2 ± 225.0	1942.3		N	8.5 ± 1.9	8.5	
σ_v' (psf)	819.7 ± 112.3	960.7		C_R	0.91	0.95	
a_{max} (g)	0.250 ± 0.050	0.25		C_S	1.00	1.00	
r_d	0.81 ± 0.067	0.95		C_B	1.00	1.00	
CSR	0.286 ± 0.063	0.308		C_E	1.22	1.22	
Equivalent Magnitude	6.9	6.9		C_N	1.61	1.42	
MSF		1.17		$(N_1)_{60}$	15.2 ± 3.4	14.0	
CSRN		0.240					

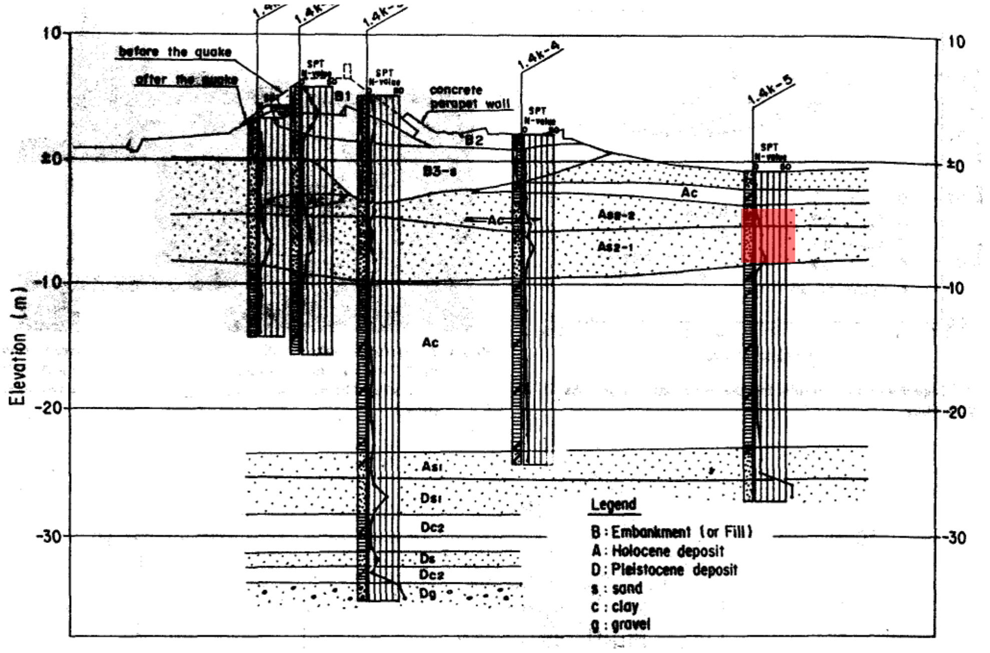

γ_s=110±3 pcf

γ_s=115±3 pcf

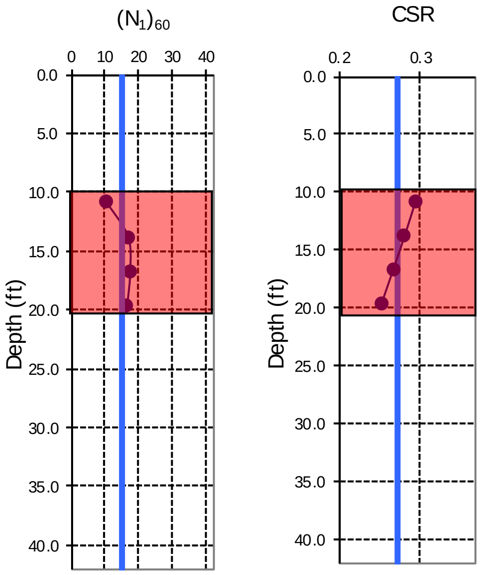

Depth (m)	Depth (ft)	CSR	N	C_N	C_R	$(N1)_{60}$
3.3	10.8	0.313	5.0	1.93	0.86	***10.1***
4.2	13.8	0.297	9.0	1.71	0.89	***16.7***
5.1	16.7	0.279	10.0	1.55	0.92	***17.4***
6.0	19.7	0.262	10.0	1.43	0.95	***16.5***
					**Mean:**	15.2
					**St. Dev.**	3.4
